# The impact of Traditional Chinese Medicine on mouse gut microbiota abundances and interactions based on Granger causality and pathway analysis

**DOI:** 10.3389/fmicb.2022.980082

**Published:** 2022-11-11

**Authors:** Yi Zhang, Dahan Zhang, Xiaogang Bai, Yang Chen, Qinwei Qiu, Xiaoxiao Shang, Yusheng Deng, Hongyan Yang, Xiaodong Fang, Zhimin Yang, Lijuan Han

**Affiliations:** ^1^Department of Scientific Research, Kangmeihuada GeneTech Co., Ltd., (KMHD), Shenzhen, China; ^2^Department of Mathematics of Science College/Hebei Province Key Laboratory of Molecular Chemistry for Drug, Hebei University of Science and Technology, Shijiazhuang, China; ^3^Institute of Genetics and Developmental Biology, Chinese Academy of Sciences, Beijing, China; ^4^State Key Laboratory of Dampness Syndrome of Chinese Medicine, The Second Affiliated Hospital of Guangzhou University of Chinese Medicine, Guangzhou, China

**Keywords:** bioinformatics, Granger causality, Traditional Chinese Medicine, intestinal microbiology, molecular mechanisms

## Abstract

**Objectives:**

The intestinal microbiota is essential in absorbing nutrients and defending against pathogens and is associated with various diseases, including obesity, type 2 diabetes, and hypertension. As an alternative medicine, Traditional Chinese Medicine (TCM) has long been used in disease treatment and healthcare, partly because it may mediate gut microbiota. However, the specific effects of TCM on the abundance and interactions of microbiota remain unknown. Moreover, using TCM ingredients and data detailing changes in the abundance of gut microorganisms, we developed bioinformatic methods that decipher the impact of TCM on microorganism interactions.

**Methods:**

The dynamics of gut microorganisms affected by TCM treatments is explored using a mouse model, which provided the abundance of 70 microorganisms over time. The Granger causality analysis was used to measure microorganism interactions. Novel “serial connection” and “diverging connection” models were used to identify molecular mechanisms underlying the impact of TCM on gut microorganism interactions, based on microorganism proteins, TCM chemical ingredients, and KEGG reaction equations.

**Results:**

*Codonopsis pilosula* (Dangshen), *Cassia twig* (Gui Zhi), *Radices saussureae* (Mu Xiang), and Sijunzi Decoction did not cause an increase in the abundance of harmful microorganisms. Most TCMs decreased the abundance of *Bifidobacterium pseudolongum*, suggesting a *Bifidobacterium pseudolongum* supplement should be used during TCM treatment. The Granger causality analysis indicated that TCM treatment changes more than half the interactions between the 70 microorganisms, and “serial connection” and “diverging connection” models suggested that changes in interactions may be related to the reaction number connecting species proteins and TCM ingredients. From a species diversity perspective, a TCM decoction is better than a single herb for healthcare. The Sijunzi Decoction only significantly increased the abundance of *Bifidobacterium pseudolongum* and did not cause a decrease in the abundance of other species but was found to improve the alpha diversity with the lowest replacement rate.

**Conclusions:**

Because most of the nine TCMs are medicinal and edible plants, we expect the methods and results presented can be used to optimize and integrate microbiota and TCMs into healthcare processes. Moreover, as a control study, these results can be combined with future disease mouse models to link variations in species abundance with particular diseases.

## Introduction

The intestinal microbiota consists of 10^13^-10^14^ microorganisms, with a gene number 100 times that of the human genome (Gill et al., 2006). This microbiota acts as an important ecosystem (Sonnenburg et al., 2005) that plays essential biological roles in maintaining health by potentially protecting the host from pathogen infections (Sorbara and Pamer, 2019) and other diseases (Xu et al., 2015; Liu et al., 2017). Compared with an abundance per sample, a time series of microorganism abundance can characterize dynamic rather than static processes and examine inter-microorganism dependencies between adjacent time points (Mehta et al., 2018), and this scheme is used to study gut microorganisms in infants (Vatanen et al., 2016) and the effects of type 1 diabetes (T1D) (Kostic et al., 2015) and antibiotics on gut microbiota (Yassour et al., 2016).

Most human gut microbiome experiments must be carried out with animal models, mainly mouse models (Nguyen et al., 2015), for ethical and safety reasons. Germ-free (Schaedler et al., 1965), ASF (altered Schaedler flora) (Brand et al., 2015) and HFA (human flora-associated) (Hirayama et al., 1995) are often used in mouse model experiments. The mouse intestinal bacterial collection (miBC) database contains gut microbes from various mice (Lagkouvardos et al., 2016), and the mouse gut microbial biobank (mGMB) contains data covering 126 species represented by 244 strains (Liu et al., 2020). However, only a few projects have systematically constructed an abundance time series of mouse intestinal microorganisms, which limits our understanding of microorganism interactions.

As an important alternative medicine, Traditional Chinese Medicine (TCM) has a long utilization and treatment history over thousands of years and has contributed significantly to human disease prophylaxis and therapy (Qiu, 2007). TCM medicine for COVID-19 has received national clinical approval (Lyu et al., 2021). The World Health Organization has estimated that up to 80% of the global population living in developing countries relies on herbal medicines as a primary source of healthcare (World Health Organization, 2004). Even in developed countries in Europe and North America, herbal medicines are consumed in large quantities, and there is an increasing demand for these complementary and alternative medicines (Ekor, 2014). By studying ancient literature on TCM, Youyou Tu won the Nobel Prize for discovering artemisinin, a drug refined from a TCM, *Artemisia apiacea*, that can be used to treat malaria. Hence, investigating TCM ingredients as potential therapies is a worthwhile venture. TCM can also be used to treat intestinal diseases, perhaps representing the most important potential of TCM in medical therapy (Teschke et al., 2015; Bailly, 2021). Literature has studied the relationship between microbiota and TCM or its ingredients (Gong et al., 2020; Zheng et al., 2020); however, few papers have focused on the association of gut microbiota and TCM for intestinal diseases. In this report, we focus on nine effective TCMs for treating intestinal disease and explore their impact on typical mouse gut microorganisms. As a control, the results can be combined with future disease mouse models to identify variations in microorganism abundance associated with the disease. Moreover, most of the nine TCMs are both medicinal and edible plants; thus, the methods and results herein can be used to optimize and integrate microbiota and the nine TCMs into healthcare and intestinal disease treatments.

The interactions of microorganisms contribute to the stability of the microbiome. The strength of this interaction can be measured by the Granger causality approach, which identifies causality between different time series (Granger, 1969), can be used to construct sensorimotor cortical networks (Brovelli et al., 2004) and gene regulation networks (Finkle et al., 2018) and to connect species in complex ecosystems (Sugihara et al., 2012). A current review provides a comprehensive summary (Shojaie and Fox, 2022). In this paper, the null hypothesis for Granger causality tests is that the time series of one species' abundance (x2) does not cause the other species' time series (x1). We reject the null hypothesis when x2 does not cause x1 if *p* < 0.01 and accept the hypothesis when x2 causes x1 at the level of *p* < 0.01. For the python function “grangercausalitytests,” there are four kinds of tests for the null hypothesis: two tests are based on the *F* distribution, “params_ftest” and “ssr_ftest,” and two are based on the χ^2^ distribution, “ssr_chi2test” and “lrtest.”

To benefit healthcare and intestinal disease TCM treatment, we used the bio-statistic and Granger causality methods to explore the impact of TCM on gut microorganism abundance and interactions using nine TCMs and standard mouse gut microorganism sequencing data. Moreover, by novel serial and diverging connection models, we also deduced possible reaction pathways underlying the impact of the interactions.

## Materials and methods

### Experiment part 1: Preparing water extract from the TCM

The TCMs were obtained from Kangmei Pharmaceutical Industry Co., Ltd, with batch numbers (BN) as follows: *Codonopsis pilosula* (Dangshen, BN:17040212), *Poria cocos* (Fuling, BN:170809401), *Rhizoma dioscoreae* (Shan Yao, BN:170711221), *Radices saussureae* (Mu Xiang, BN:170501921), *Rhizoma Zingiberis* (Ganjiang, BN:170708491), *Cassia twig* (Gui Zhi, BN:170704361), *Magnolia officinalis* (Houpo, BN:170700141), White *Atractylodes* rhizome (Bai Zhu, BN: 170812601), *Poria cocos* (Fuling, BN:170809401), *Liquiritiae glycyrrhizae* (Gan Cao, BN:170808051), *Astragalus mongholicus* (Huangqi, BN:170800541), and *Chinese angelica* (Dang Gui, BN:170708441).

Initially, we weighed seven single dried Chinese herbs and two compounds used in intestinal treatment (Teschke et al., 2015; Bailly, 2021). *Codonopsis pilosula* may affect small intestinal propulsion movement (Li et al., 2004). *Poria cocos* can improve intestinal barrier function and intestinal microbiota (Yu and Liu, 2021). *Rhizoma dioscoreae* (Fu et al., 2008) and *Radices saussureae* (Tang et al., 2010) are medicines used to treat gastrointestinal disorders. *Magnolia officinalis* is a popular TCM with antibiotic and analgesic effects (Lu et al., 2004). *Cassia twig* is also an important alternative medicine that displays anti-inflammatory, antibacterial, antiviral, anti-allergic, and analgesic functions (Ye et al., 2022). The TCMs used herein have been verified by clinical investigation, are herbal slices without any secondary processing (granule, powder, and herbal leaven), and most of them, including *Codonopsis pilosula* (Dangshen), *Poria cocos* (Fuling), *Rhizoma dioscoreae* (Shan Yao), *Rhizoma zingiberis* (Ganjiang), *Liquiritiae glycyrrhizae* (Gan Cao), *Astragalus mongholicus* (Huangqi), and *Chinese angelica* (Dang Gui), are medicinal and edible plants. TCM samples listed in Tables 1, 2 were placed in round-bottom flasks and 10-fold the weight of pure water was added, and these samples were left to soak for 30 min. Samples were then heated in the round-bottom flasks and boiled for over an hour. After cooling, the flasks containing the TCM water extracts were filtered with six-layer gauze, and the nine water extracts were collected. We then added pure water (eight-fold in weight) to the nine remaining TCM residues, and after repeating the boiling, cooling, and filtering process, these nine water extracts were also stored. We combined the water extracts derived from the same TCMs and concentrated the nine water extracts to yield the crude drug concentrations listed in Tables 1, 2. Samples were stored at −20°C.

**Table 1:** Single TCM gavage dose for mouse treatment.

**Single TCM**	**Weight of TCM (g)**	**Concentrated volume of TCM water extract (mL)**	**Crude drug concentration (g/mL)**	**Mouse dose (mg/g)**
*Codonopsis pilosula* (Dangshen)	260	200	1.3	13
*Poria cocos* (Fuling)	260	200	1.3	13
*Rhizoma Dioscoreae* (Shan Yao)	260	200	1.3	13
*Radices saussureae* (Mu Xiang)	130	200	0.65	6.5
*Rhizoma zingiberis* (Ganjiang)	130	200	0.65	6.5
*Cassia twig* (Gui Zhi)	195	200	0.975	9.75
*Magnolia officinalis* (Houpo)	195	200	0.975	9.75

**Table 2:** Compound TCM gavage dose for mouse treatment.

**Compound TCM**	**Composition of TCM**	**Weight of TCM (g)**	**Concentrated volume of TCM water extract (mL)**	**Crude drug concentration (g/mL)**	**Mouse dose (mg/g)**
Sijunzi decoction	*Codonopsis pilosula* (Dangshen)	110	200	2	20
	*White Atractylodes rhizome* (Bai Zhu)	110			
	*Poria cocos* (Fuling)	110			
	*Liquiritia glycyrrhiza* (Gan Cao)	72			
Dang Gui Bu Xue decoction	*Astragalus mongholicus* (Huangqi)	200	200	1.2	12
	*Chinese angelica* (Dang Gui)	40			

### Experiment part 2: Feeding mice and collecting fecal samples

This study was performed with 198 male-specific pathogen-free (SPF) C57BL/6J mice aged 6 weeks. The mice were housed in the SPF animal breeding room of KMHD under License No. SYXK (Yue) 2019-0205, at a temperature of 23–25°C, moisture 54–57% and a 12-h light/dark cycle. This experimental study was approved by the Ethics Committee of KMHD under Approval No. IACUC-0210831-1.

**Figure 1:**
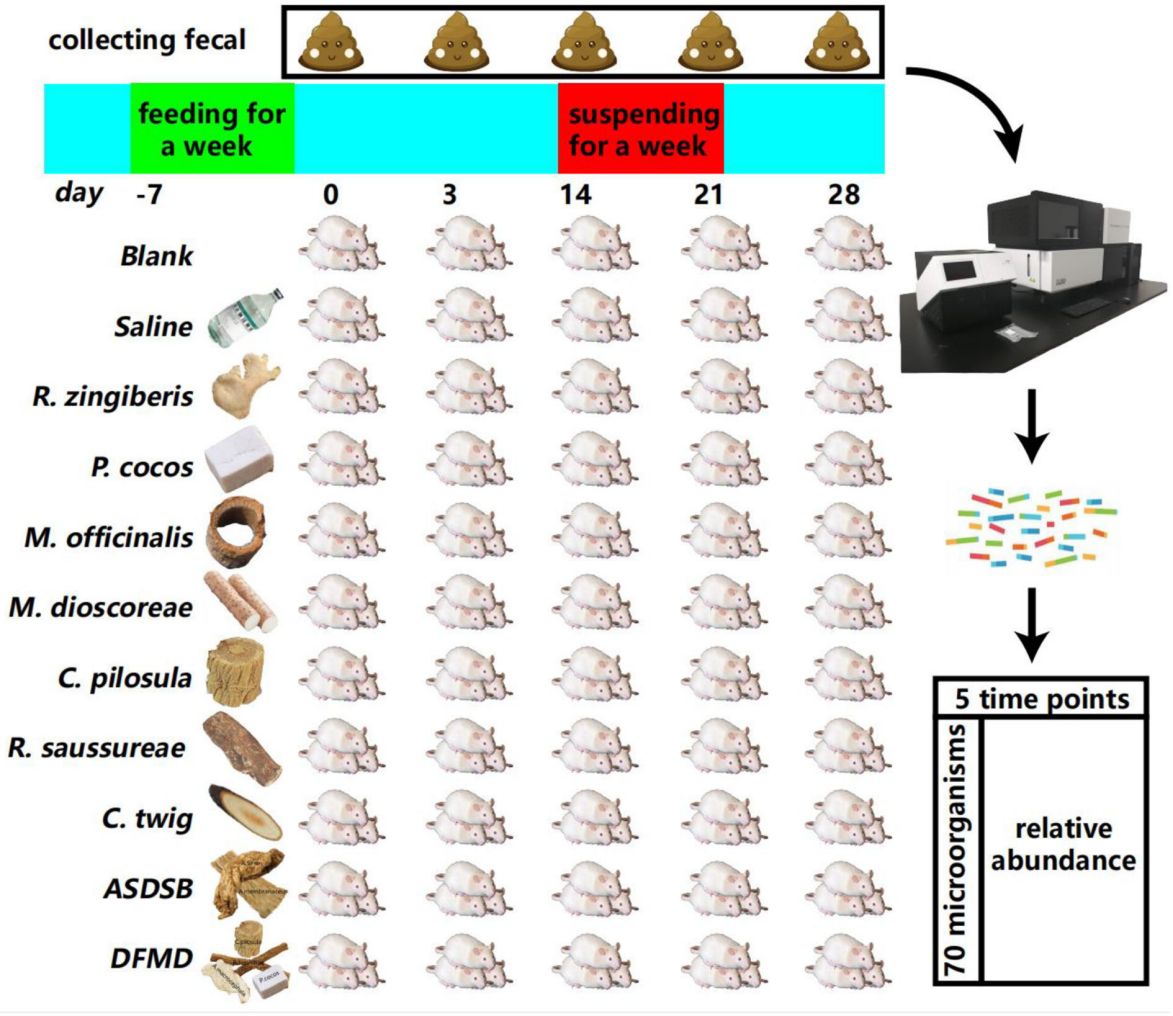
Experimental design and the microorganism relative abundance calculation for 70 species over five time points, where ASDSB represents the Dang Gui Bu Xue Decoction, and DFMD represents the Sijunzi Decoction.

After feeding for a week, 198 mice were randomly grouped into 11 groups, with each group consisting of 18 mice. Among the 11 groups, nine groups were treated with different TCMs by gastric infusion (Figure 1), and the other two groups were the blank control group (blank group) and the physiological saline treatment group (saline group). Mice were given free access to food and drinking water.

Fecal collection process: after alcohol disinfection of each cage, three mice in each cage acted freely and defecated freely. Using sterile forceps, fresh feces were collected, placed into 2EP tubes, and quickly placed at −80°C. The fecal collection was performed before intragastric administration. The intragastric administration was suspended during the 3rd week (days 15 to 21) of the experiment.

### Bioinformatics part 1: Sequencing metagenome and calculating the relative microorganism abundance in a time series

High-quality genomic DNA was extracted from the mouse feces. The DNA that passed quality control was then used to construct a library using the TruSeq DNA HT Sample Prep Kit. Paired-end sequencing (2 × 150 bp) was carried out using the Illumina HiSeq X10 platform. After removing the host (mouse) and low-quality sequences, the relative abundance was calculated using MetaPhlAn 3.0 (Beghini et al., 2021) with default parameter values. Functional profiling was performed using HUMAnN3 (Beghini et al., 2021) in the UniRef90 mode. In Supplementary material 1, *merged_abundance_table_species70.csv*, we list the relative abundance of 70 species at 0, 3, 14, 21, and 28-day time points under the 11 different treatments, where the sum of each column is 100.

### Bioinformatics part 2: Chemical composition of TCMs, metabolites of microorganisms, and KEGG reactions

KEGG (Kyoto Encyclopedia of Genes and Genomes) (http://rest.kegg.jp/list/reaction) includes 11,075 reactions and represents a primary source to relate TCM with microorganisms. Chemical compositions and keywords of chemical reactions related to *Cassia twig* (Gui Zhi) and Sijunzi Decoction are listed in Supplementary material 2. Among the 70 microorganisms we annotated from mice gut microbiota, 28 microorganisms are present in KEGG (Supplementary Table 1 in Supplementary material 3), and their proteins and metabolites can also be obtained from KEGG. We retrieved reactions involving each TCM composition or microorganism metabolite from KEGG. Thus, a TCM or microorganism can be related to several hundred reactions. If a TCM and a microorganism share some reactions, they may naturally interact through these shared reactions. Similarly, the intersection of reactions between a TCM and two microorganisms may indicate where the TCM affects microorganism interactions.

## Results and discussion

### Beneficial impact of TCM on microorganism abundance

Most microorganisms with increasing abundance following treatment with the nine TCMs have positive health functions for humans. There were 11 TCM-free samples on day 0. After *N* (0, 1) normalizing each column of *merged_abundance_table_species70.csv*, from the 11 TCM-free samples, we estimated the mean abundance values and variances for all 70 microorganisms. Then, we calculated the mean relative abundance for each microorganism species under each TCM treatment on days 3, 14, 21, and 28. If the mean is in the 95% confidence interval, treatment does not significantly change the abundance of the species at the *p*1 < 0.05 level according to the two-tail test. A mean located on the left side of the 95% confidence interval indicates that treatment significantly decreases the abundance of a species, whereas a mean on the right side of the confidence interval indicates that treatment increases the abundance of a species, with *p*2 < 0.025 using the one-tail test.

Regarding setting *p*2 < 0.005 with the one-tail test, no microorganisms treated with the nine TCMs showed a decrease; however, setting *p*2 < 0.05 with the one-tail test revealed that Dang Gui Bu Xue Decoction, *Cassia twig* (Gui Zhi), and Sijunzi Decoction did not cause a significant decrease in the abundance of all microorganisms, whereas the other TCMs caused a reduction in the abundance of *Bifidobacterium pseudolongum*. This observation suggests that people taking TCM for healthcare should also take probiotics (including *Bifidobacterium pseudolongum*) to neutralize the mild adverse effects because this species is enriched with specific enzymes that degrade complex plant carbohydrates and host glycans (Xiao et al., 2021).

**Figure 2:**
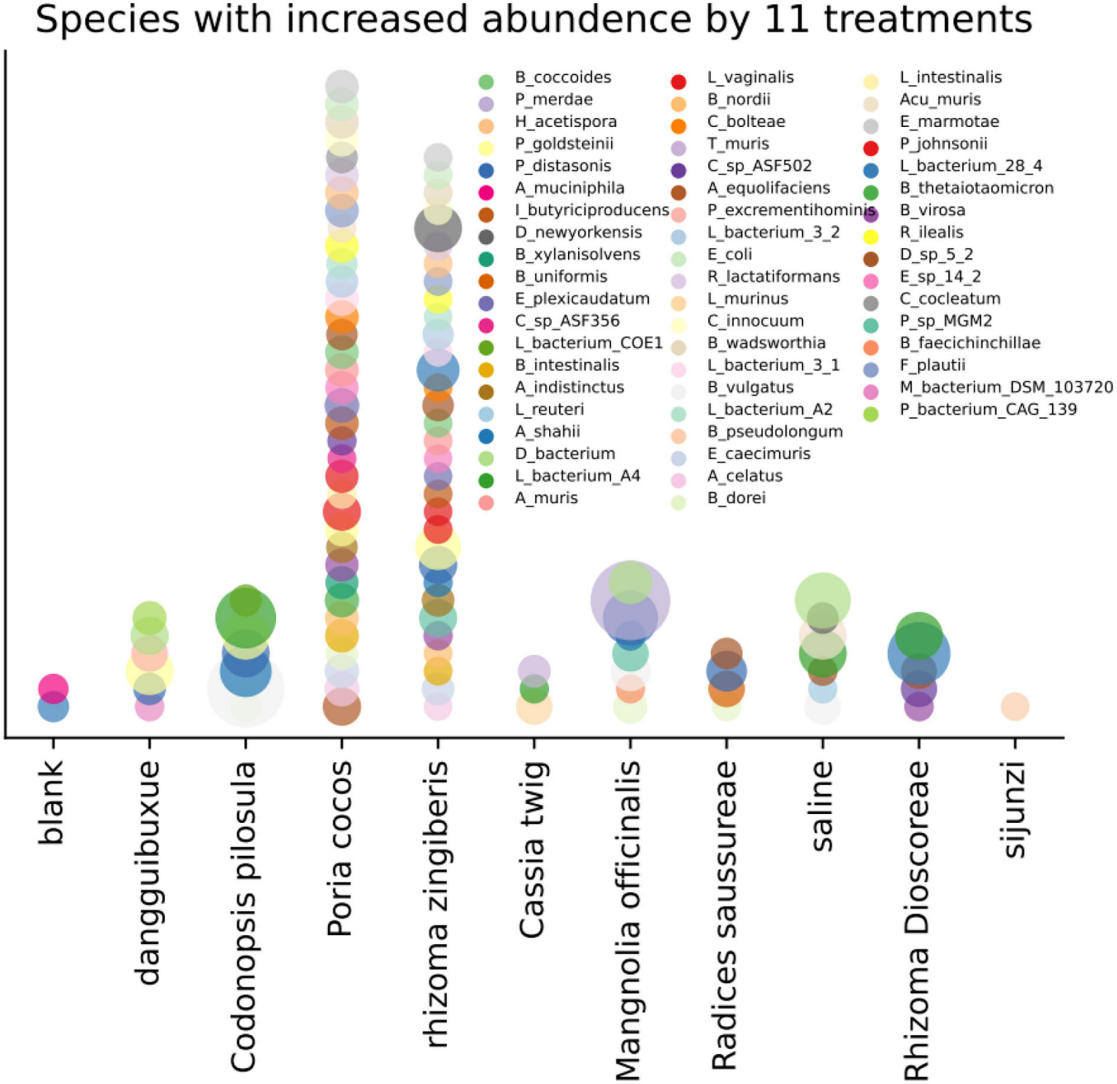
Increase in species abundance by the 11 treatments, where *p*2 < 0.025 with the one-tail test.

In Figure 2 and Supplementary Table 2 in Supplementary material 3, we show species whose abundance increased under the nine treatments with a *p*2 < 0.025 using the one-tail test. Many microorganisms have positive (or conditionally positive) benefits to human health, including *Lactobacillus_johnsonii* (Lim et al., 2017), *Parasutterella_excrementihominis* (Fart et al., 2020), *Bacteroides_vulgatus* (You et al., 2019), *Parabacteroides_distasonis* (Wang et al., 2019), *Parabacteroides_goldsteinii* (Wu et al., 2018), and *Lachnospiraceae_bacterium_A4*, which increase the short-chain fatty acids (SCFA) content in the caecum, *Enterorhabdus_caecimuris* (Fang et al., 2022), which aids the production of tryptophan metabolism in the gut, and *Bacteroides_thetaiotaomicron* (Delday et al., 2019) and *Dubosiella_newyorkensis*, which improve various health indicators of a fatty mouse. *Lactobacillus_murinus* (Perelmuter et al., 2008), *Bacteroides_uniformis* (Zhao et al., 2016), *Adlercreutzia_equolifaciens* (Zhu et al., 2015), and *Bacteroides_intestinalis* are helpful for treating type 2 diabetes mellitus.

A few microorganisms with adverse conditional functions toward human health were also identified to increase in abundance following TCM treatments (Figure 2, Supplementary Table 2 in Supplementary material 3). For example, *Desulfovibrionaceae_bacterium*, which produces H_2_S, can harm intestinal epithelial cells, and *Clostridium_bolteae* (Cai et al., 2020), *Flavonifractor_plautii* (Wang et al., 2021), and *Bilophila_wadsworthia* also produce H_2_S and are associated with appendicitis and some other local inflammation. *Lachnospiraceae_bacterium_28_4* and *Romboutsia_ilealis* was also found and may contribute to the development of diabetes in obese mice (Kameyama and Itoh, 2014; Rodrigues et al., 2021).

Most species identified to increase in abundance when exposed to the nine TCMs were positive or conditionally positive. This observation indicates that the nine TCMs, which have similar functions according to TCM theory (Wang and Guo, 2015), i.e., tonifying the spleen, regulating the flow of vital energy, and removing obstructions, have largely a consistent impact on gut microorganisms.

### Biomarker characterization of the TCM treatments

**Figure 3:**
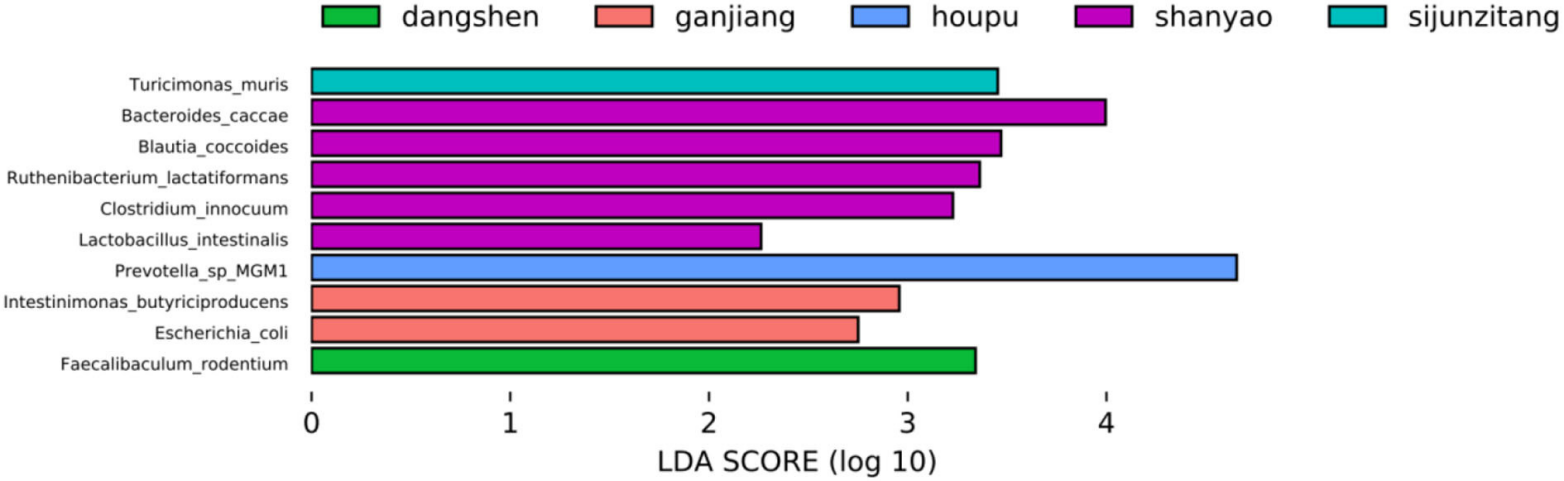
LEfSe analysis identifies microorganism biomarkers that show distinctions among the nine TCM treatments.

The LDA Effect Size (LEfSe) (Segata et al., 2011) is used to discover biomarkers that show a difference between two or more biological conditions or intervention groups. LEfSe is used to identify both statistical significance and biological relevance. LEfSe was applied to our data matrix (Supplementary material 1, *merged_abundance_table_species70.csv*), and the results are presented in Figure 3. *Rhizoma dioscoreae* (Shan Yao) was found to have five biomarkers: *Bacteroides_caccae, Blautia_coccoides, Ruthenibacterium_lactatiformans, Clostridium_innocuum*, and *Lactobacillus_intestinalis*. *Rhizoma zingiberis* (Ganjiang) was found to have *Intestinimonas_butyriciproducens* and *Escherichia_coli* as biomarkers. *Codonopsis pilosula* (Dangshen), Sijunzi, and *Magnolia officinalis* (Houpo) were found to have only one biomarker. Compared with Figure 2, the abundances of these biomarkers did not increase in their corresponding TCM treatment, except for *Rhizoma zingiberis* (Ganjiang), where the abundance of the two biomarkers increased.

### TCM can change the strength of the inter-microorganism interaction

**Figure 4:**
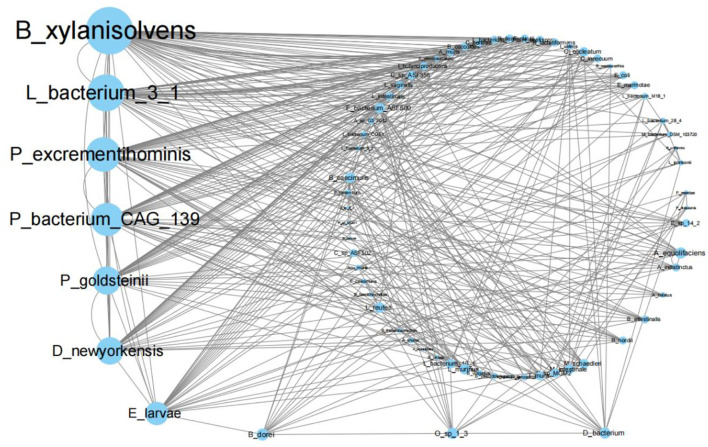
Granger causality network for mouse gut microorganisms treated by the Sijunzi Decoction with *p* < 10^−4^.

Taking the python package statsmodels.tsa.stattools, importing the grangercausalitytests function and using gc_res = grangercausalitytests [DATA, (1)], the Granger causality *p*-values of two species were calculated based on their abundance time series included in variable DATA. We used the likelihood ratio test (“lrtest”), a χ^2^ distribution test, to calculate the *p*-values of the Granger causality. We found many possible causality relationships under the 11 treatments when *p* < 10^−4^. For example, we showed that the Granger causality network for mouse gut microorganisms treated by Sijunzi Decoction has a *p* < 10^−4^ (Figure 4). Supplementary Figures 1–10 of Supplementary material 4 show the Granger causality network for the other 10 networks. In summary, blank control, Dang Gui Bu Xue Decoction, *Codonopsis pilosula* (Dangshen), *Poria cocos* (Fuling), *Rhizoma zingiberis* (Ganjiang), *Cassia twig* (Gui Zhi), *Magnolia officinalis* (Houpo), *Radices saussureae* (Mu Xiang), saline control, *Rhizoma dioscoreae* (Shan Yao), and the Sijunzi Decoction have 372, 414, 426, 428, 467, 588, 443, 543, 431, 410 and 392 pairs of significantly correlated species, respectively.

**Figure 5:**
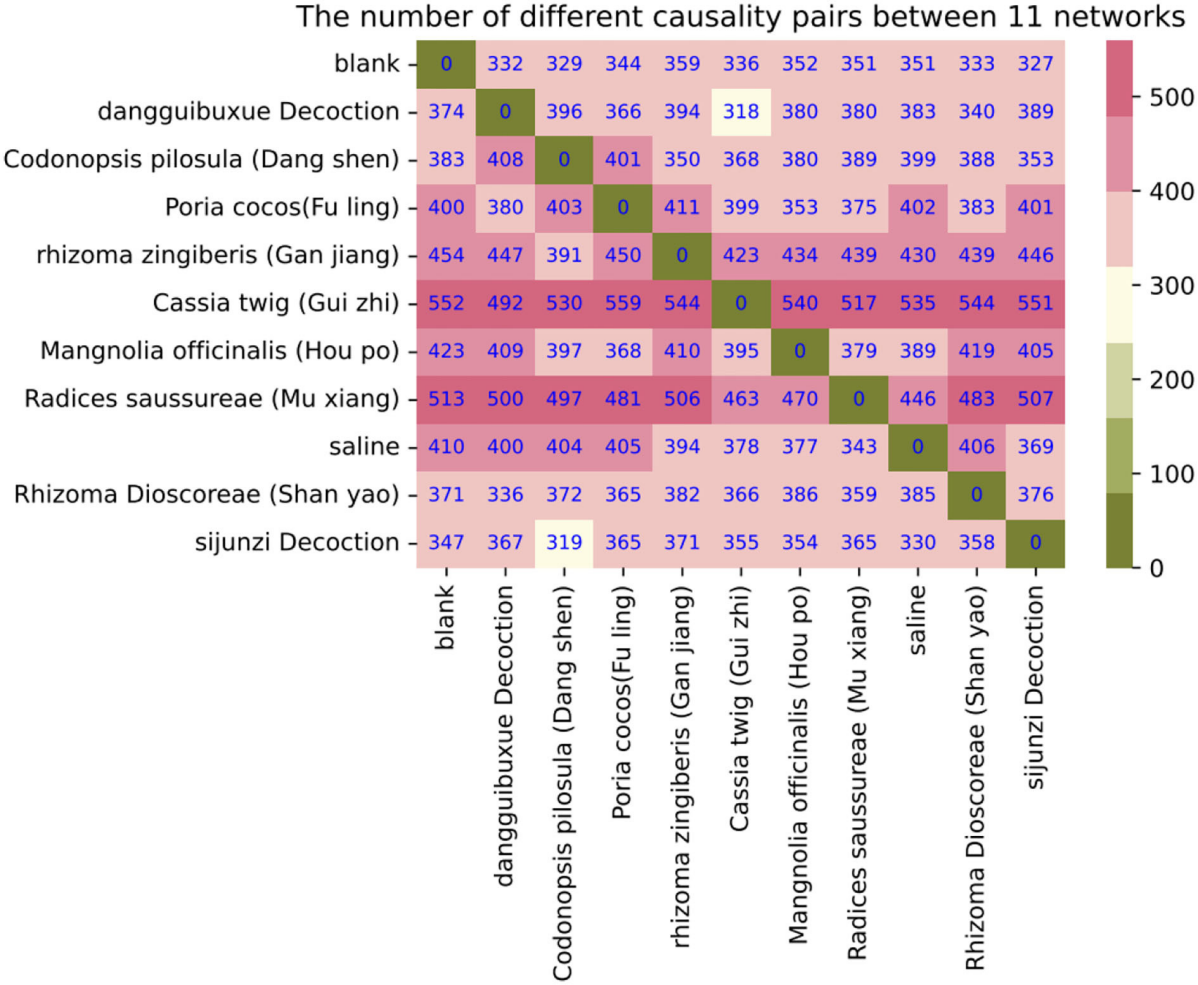
The number of different causality pairs among the 11 Granger causality networks.

Compared with the blank control group, the newly added causality pairs included 374, 383, 400, 454, 552, 423, 513, 410, 371, and 347 for the last 10 treatments, and 332, 329, 344, 359, 336, 352, 351, 351, 333, and 327 causality pairs vanished. In Figure 5, we also show the number of different causality pairs identified between 11 Granger causality networks with *p* < 10^−4^. Thus, correlations between the 70 species were found to change considerably by TCMs.

After setting lrtest to *p* < 0.00000001, a stricter cutoff, for blank control, Dang Gui Bu Xue Decoction, *Codonopsis pilosula* (Dangshen), *Poria cocos* (Fuling), *Rhizoma zingiberis* (Ganjiang), *Cassia twig* (Gui Zhi), *Magnolia officinalis* (Houpo), *Radices saussureae* (Mu Xiang), saline control, *Rhizoma dioscoreae* (Shan Yao), and Sijunzi Decoction, there remained 36, 30, 44, 45, 73, 22, 58, 61, 31, 64, and 45 pairs of significantly correlated species, respectively. Compared with the blank group, the number of newly added causality pairs was 29, 44, 45, 73, 22, 57, 61, 31, 63, and 45 for the last 10 treatments, and 35, 36, 36, 36, 36, 35, 36, 36, 35, and 36 causality pairs vanished, respectively. These results further confirmed that TCMs greatly changed the species correlations.

Moreover, the biomarkers shown in Figure 3 are not among the first 10 highest degree species in all Granger causality networks, including Figure 4 and Supplementary Figures 1–10 of Supplementary material 4.

### Alpha and beta diversity

**Figure 6:**
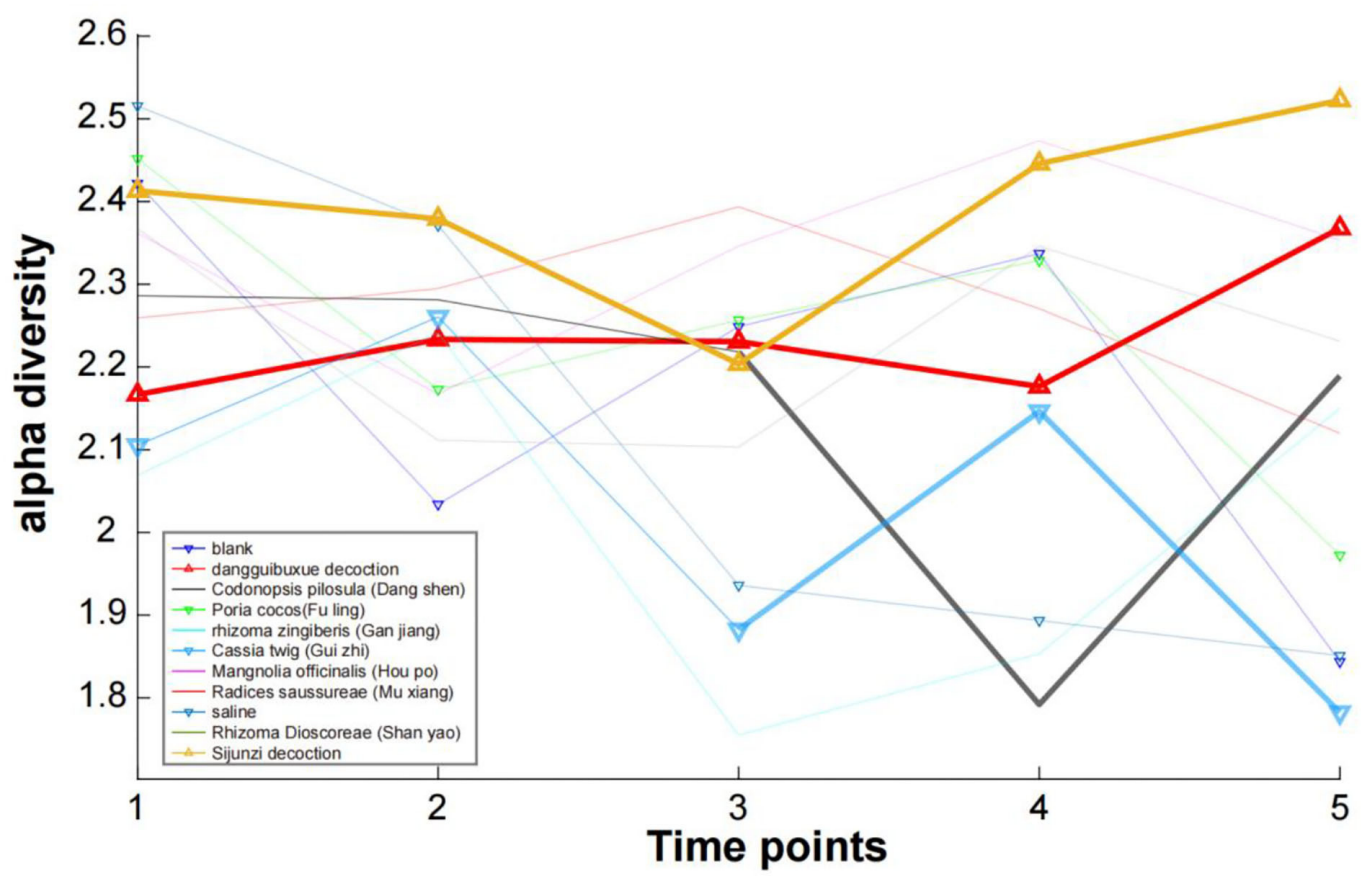
The alpha diversity Shannon index for each sample of each treatment.

A diversity index for a dataset is a quantity reflecting how many different species exist and how evenly individuals are distributed among those species. The diversity index increases with increasing species number and increasing evenness. Alpha diversity measures, in one sample, the number of different species and their different abundance, whereas beta diversity focuses on a group of samples and compares species compositions between different samples. For each sample of each treatment in our study, an alpha diversity index was calculated (Figure 6). Here, we found that for two decoction TCMs (Sijunzi Decoction and Dang Gui Bu Xue Decoction), the alpha diversity value increased slightly when comparing their start and end diversity values. In contrast, for the saline control, *Poria cocos* (Fuling) and *Cassia twig* (Gui Zhi), a decrease in the alpha diversity values were observed because their diversity at point “5” was significantly lower than that at point “1”. The remaining TCMs showed no significant change in their alpha diversity. From the perspective of species diversity, TCM decoctions were found to have a greater effect when compared with single herbs.

As shown in Supplementary material 5, we also calculated the Simpson and Invsimpson index, and found that three indexes were consistent and closely related, i.e., the Shannon index can describe the diversity of each sample.

Beta diversity measures differences between the species composition of samples, with larger beta diversity values indicating a larger replacement rate (Anderson et al., 2010). We calculated the beta diversity for the 11 treatments, with a beta diversity of 0.2222222 determined for the blank control group. The beta diversity of the Dang Gui Bu Xue Decoction treatment group was 0.2441315, whereas the value for the *Codonopsis pilosula* (Dangshen) treatment group was 0.2443439. A value of 0.2672811 was determined for *Poria cocos* (Fuling) treatment, 0.3333333 for *Rhizoma zingiberis* (Ganjiang) treatment, 0.2918660 for *Cassia twig* (Gui Zhi) treatment, 0.3302752 for *Magnolia officinalis* (Houpo) treatment, 0.3594470 for *Radices saussureae* (Mu Xiang) treatment, 0.2127660 for the saline control treatment, 0.4285714 for *Rhizoma dioscoreae* (Shan Yao) treatment and 0.1842105 for Sijunzi Decoction treatment. The Sijunzi Decoction, saline, and blank treatments have the lowest replacement rates, whereas Ganjiang, Houpo, Mu Xiang and Shan Yao have the highest replacement rates. Interestingly, the Sijunzi Decoction was found to only increase the abundance of *Bifidobacterium_pseudolongum* significantly, with no decrease in any other species abundance. Thus, this treatment improved the alpha diversity with the lowest replacement rate.

### Exploring the mechanisms of TCM that affect the microorganism correlation strength by contrast analysis

Two TCMs (*Cassia twig* and Sijunzi Decoction) and two microorganisms (*Adlercreutzia equolifaciens* and *Bifidobacterium pseudolongum)* were used as examples to clarify the novel mechanism exploration method as follows. First, the two microorganisms present very different correlation strengths under the two TCM treatments. Second, specific reactions involving the chemical compositions of the two TCMs and metabolisms of the two microorganisms were retrieved. Finally, by using novel serial and diverging connection reaction path models, contrast analysis was performed to deduce the possible mechanism of TCM that changes the microorganism correlation strength.

The interaction between *Adlercreutzia equolifaciens* and *Bifidobacterium pseudolongum* presents very different correlations: treatment with *Cassia twig* (Gui Zhi) gave a Granger causality lrtest *p*-value of 0.000064934, whereas treatment with the Sijunzi Decoction yielded a Granger causality lrtest *p*-value of 0.93943. We typically study three models to explore the relationship among the three objects (a TCM and two microorganisms): serial connection, diverging connection, and converging connection. Here, the “converging connection” is not applicable because microorganisms cannot have an impact on the TCM.

For convenience, in the following study, in Supplementary material 2 we have listed the chemical compositions of *Cassia twig* (Gui Zhi) and Sijunzi Decoction, respectively, and the metabolites of *Adlercreutzia_equolifaciens* and *Bifidobacterium_pseudolongum* are available from KEGG (data not shown due to size). “Serial connection” means three equations are involved in *Adlercreutzia_equolifaciens* → *Bifidobacterium_pseudolongum* → chemical composition of TCM, or *Bifidobacterium_pseudolongum* → *Adlercreutzia_equolifaciens* → chemical composition of TCM, where the TCM may increase the inter-species correlation by driving the chemical reaction based on Le Chatelier's principle/the equilibrium law.

For *Cassia twig*, taking the following as an example: {rn:R00237} acetyl-CoA + pyruvate ↔ (3*S*)-citramalyl-CoA (*A. equolifaciens*) → {rn:R02955} acetate + (3*S*)-citramalyl-CoA (*A. equolifaciens*) ↔ Acetyl-CoA (*B. pseudolongum*) + Citramalate → {rn:R10474} Acetyl-CoA (*B. pseudolongum*) + cinnamyl alcohol (*Cassia twig*) ↔ CoA + Cinnamyl acetate. Clearly, there are three equations involved in *A. equolifaciens* → *B. pseudolongum* → *Cassia twig*. Here, the metabolite “(3*S*)-citramalyl-CoA” of *A. equolifaciens* helps to produce the metabolite “acetyl-CoA” of *B. pseudolongum*, and the chemical composition of “cinnamyl alcohol” from *Cassia twig* can consume “acetyl-CoA” of *B. pseudolongum*. Hence, this serial connection may increase the correlation between *A. equolifaciens* and *B. pseudolongum*.

In Supplementary material, more information is provided on the novel serial connection models: in Supplementary material 6, we list 2,775 such serial connections: *A. equolifaciens* → *B. pseudolongum* → *Cassia twig*. In Supplementary material 7, 1,352 serial connections are listed: *B. pseudolongum* → *A. equolifaciens* → *Cassia twig*. In Supplementary material 8, 1,419 such serial connections are listed: *A. equolifaciens* → *B. pseudolongum* → Sijunzi Decoction, and in Supplementary material 9, we have 390 serial connections: *B. pseudolongum* → *A. equolifaciens* → Sijunzi Decoction. Clearly, for *Adlercreutzia_equolifaciens* and *Bifidobacterium_pseudolongum*, the number of serial connections driven by *Cassia twig* (2,775 + 1,352 = 4,127) is much larger than that driven by the Sijunzi Decoction (1,419 + 390 = 1,809). This observation may partially explain the stronger Granger causality of *A. equolifaciens* and *B. pseudolongum* under *Cassia twig* than that under the Sijunzi Decoction.

The “diverging connection” indicated that the compositions of TCM may react with both metabolites of *A. equolifaciens and B. pseudolongum* through the same or different reaction equations. As shown in Supplementary material 10, *Cassia twig* (Gui Zhi) connects with both *A. equolifaciens* and *B. pseudolongum* through 75 shared chemical reactions, whereas *Cassia twig* (Gui Zhi) has five specific equations with *A. equolifaciens* and 15 specific equations with *B. pseudolongum*. Data in Supplementary material 10 also shows that the Sijunzi Decoction connects with *A. equolifaciens* and *B. pseudolongum* through 11 shared chemical reactions, without any specific reactions. The more shared chemical reactions may explain, in part, the higher correlation between *A. equolifaciens* and *B. pseudolongum* under *Cassia twig* treatment than that found under Sijunzi Decoction treatment.

## Conclusion

Mouse gut experiments and sequencing technology afforded a systematic analysis of the abundance of gut microorganisms over time with and without TCM treatments. The study also provided an opportunity to explore how TCM affects microorganism abundance and their inter-species correlations. Ingredient and reaction pathway analysis was used to explain the impact of TCM on gut microorganisms, and we focused our analysis on two TCMs, C*assia twig* (Gui Zhi) and Sijunzi Decoction. Moreover, among the 70 microorganisms annotated in our mice model, only 28 microorganisms were annotated in KEGG. Given more detailed data, the novel serial and diverging connection models should provide additional insights into how TCM affects microorganism abundance and inter-species correlations.

## Data availability statement

The datasets presented in this study can be found in the Fecal Metagenomic sequencing reads from CNGB Nucleotide Sequence Archive under accession number CNP0002573.

## Ethics statement

The animal study was reviewed and approved by the Ethics Committee of KMHD on the use of animal subjects under Approval No. IACUC-0210831-1. Written informed consent was obtained from the owners for the participation of their animals in this study.

## Author contributions

LH, XF, HY, and YZ designed the project. YC, XS, YD, QQ, and DZ conducted the experiments. DZ, YZ, and XB analyzed the data. DZ and XB developed the algorithm and software. YZ, DZ, and LH wrote and revised the paper. All authors contributed to the article and approved the submitted version.

## Funding

This paper was supported by Science Technology and Innovation Committee of Shenzhen Municipality under Grant No. JCYJ20160331190123578 and the Natural Science Foundation of Hebei Province of China under Grant No. A2019208336.

## Conflict of interest

Authors YZ, DZ, XB, and XF were employed by Kangmeihuada GeneTech Co., Ltd. The remaining authors declare that the research was conducted in the absence of any commercial or financial relationships that could be construed as a potential conflict of interest.

## Publisher's note

All claims expressed in this article are solely those of the authors and do not necessarily represent those of their affiliated organizations, or those of the publisher, the editors and the reviewers. Any product that may be evaluated in this article, or claim that may be made by its manufacturer, is not guaranteed or endorsed by the publisher.
