# Supplementary material for: The impact of Traditional Chinese Medicine on mouse gut microbiota abundances and interactions based on Granger causality and pathway analysis
Source: Front Microbiol. 2022 Nov 11;13:980082. doi: 10.3389/fmicb.2022.980082 (PMC9692106; doi:10.3389/fmicb.2022.980082)
Supplement: Supplementary file 1 [file Table_1.DOCX]

**Chemical compositions of *cassia twig* (Gui zhi)**

D-limonene ^[1]^

alpha-cubebene ^[1]^

τ-ylangene ^[1]^

cadin-1,3,5-triene ^[1]^

aromadendrene ^[1]^

4,5,9,10-dehydration isolongifolene ^[1]^

copaene^[1]^

(S)-1-methyl-4-(5-methyl-1-methylene-4-vinyl)-cyclohexene ^[1]^

styrene^[1]^

2-β-pinene^[1]^

β-elemene^[1]^

β-fanesene ^[1]^

β-selinene ^[1]^

α-fanesene ^[1]^

megastigma-2,7(E),9-triene ^[1]^

α-curcumene ^[1]^

β-cadinene ^[1]^

α-bisabolene ^[1]^

α-bergamotene ^[1]^

δ-cadinene ^[1]^

camphene ^[1]^

β-pinene ^[1]^

β-Myrcene ^[1]^

capaene ^[1]^

Di-epi-α-cedrene ^[1]^

(-)-isocaryophyllene ^[1]^

γ-muurolene ^[1]^

α-muurolene ^[1]^

β-bisabolene ^[1]^

calamenene ^[1]^

o-menthene-2-ene ^[1]^

isoermophilene ^[1]^

cadinene ^[1]^

4-acetyl-1-methyl-1-cyclohexene ^[1]^

α-cedrene ^[1]^

γ-cadinene ^[1]^

calacorene ^[1]^

calamene ^[1]^

bicyclo [4.2.0] octa-1,3,5-triene ^[1]^

α-pinene^[1]^

1,7,7-trimethyl-bicyclo [2.2.1] hept-2-ene ^[1]^

2,6-dimethyl-6-(4-methyl-3-pentenyl)-bicyclo [3.1.1] -hept-2-ene ^[1]^

1-methyl-4-(5-methyl-1-methylene-4-hexenyl)-cyclohexene ^[1]^

2,6,6,9-tetramethyltricyclo [5.4.0.02.8] undec-9-ene ^[1]^

caryophyllene ^[1]^

1,6-dimethyl-4-(1-methylethyl) ^[1]^

cadala-1(10),3,8-triene ^[1]^

1,3-cyclohexadiene,5-(1,5-dimethyl-4-hexenyl)-2-methyl-[S-(R*,S*)]^[1]^

neoclovene ^[1]^

elixene ^[1]^

eremophilene ^[1]^

gamma.-terpinene ^[1]^

β-muurolene^[1]^

α-cadinene ^[1]^

β-guaiene ^[1]^

(-)-calamencne ^[1]^

(+)-limonene ^[1]^

L-alloaromadendrene ^[1]^

1-methyl-4-(1,5-dimethyl-1,4-hexylene) cyclohexene-1 ^[1]^

β-cedrene ^[1]^

3,7-dimethyl-E-1,3,6-octatriene ^[1]^

1,3,8-p-Menthatriene ^[1]^

hexamethyl-1,3,5-cyclononatriene ^[1]^

9-octadecenal^[1]^

β-methoxystyrene ^[1]^

cyclosativene ^[1]^

α-santalene ^[1]^

β-santalene ^[1]^

β-caryophyllene ^[1]^

(Z)-β-farnesene ^[1]^

α-humulene ^[1]^

α-amorphene ^[1]^

γ-curcumene ^[1]^

P-cadinene ^[1]^

(+)-spathulene ^[1]^

terpineol ^[1]^

paeonol ^[1]^

globulol ^[1]^

cubenol ^[1]^

α-muurolol ^[1]^

α-cadinol ^[1]^

α-bisabolol ^[1]^

β-bisabolol ^[1]^

spathalenol ^[1,3]^

4-methyl-1-(1,5-dimethyl-4-vinyl)-3-hexen-1-ol ^[1]^

3,4-dimethyl-cyclohexanol ^[1]^

myrtenol ^[1]^

benzenpropanol ^[1]^

nerolidol ^[1]^

farnesol ^[1]^

palustrol ^[1]^

β-elemol ^[1]^

α-cedrol ^[1]^

guaiol ^[1]^

2-ethoxy-1-propanol ^[1]^

phebethyl alcohol ^[1]^

4-terpineol ^[1]^

tan.-muurolol ^[1]^

cinnamyl alcohol ^[1]^

menthol ^[1]^

spathulenol ^[1]^

tau.-cadinol ^[1]^

trans-borneol ^[1]^

styryl alcohol ^[1]^

4-methyl-2-(1,5-dimethyl-4-hexenyl)-3-cyclohexen-1-ol^[1]^

epicedrol ^[1]^

L-4-terpineol L-4-^[1]^

elemol ^[1]^

β-linalool ^[1]^

cedren-13-ol ^[1]^

2-octen-1-ol ^[1]^

linderol ^[1]^

hexadecanol ^[1]^

τ-muurolol ^[1]^

torreyol ^[1]^

benzenepropanal ^[1,3]^

benzalmalonic dioldehyde ^[1]^

cinnamaldehyde ^[1,2,3]^

cinnamaldehyde,o-methoxy ^[1]^

tetradecanal ^[1]^

13-tetradecenal^[1]^

benzylidene malona ldehyde ^[1]^

myristic aldehyde ^[1]^

2-phenyl-2-propenal ^[1]^

benzeneacetaldehyde ^[1]^

hexanal ^[1]^

salicylal ^[1]^

o-anisaldehyde ^[1]^

octanal ^[1]^

benzaldehyde ^[1,3]^

(Z)-cis-cinnamic aldehyde ^[1]^

(Z)-o-methoxycinnamaldehyde ^[1]^

(E)-o-methoxycinnamaldehyde ^[1]^

citral ^[1]^

3-hydroxy-benzaldehyde ^[1]^

3-phenyl-2-propenal ^[1,3]^

3-(-2-methoxyphenyl)-2-propenal^[1,3]^

octade canal ^[1]^

m-methoxycinnamaldehyde ^[1]^

4-hydroxy-benzaldehyde ^[1]^

hyacinthin ^[1]^

2-anisaldehyde ^[1]^

(E,E)-farnesal ^[1]^

benzyl propionate ^[1]^

(E)-methylcinnamate ^[1]^

2,5-dimethyl-phenyl butyric acid methyl ester ^[1]^

lepidozenol Lepidozenol ^[1]^

1,2-benzenedicarboxylic acid,dibutyl ester ^[1]^

phenethyl alcohol,formate ^[1]^

benzoicacid,ethyl ester ^[1]^

bronyl acetate ^[1]^

cinnamic acid,methyl ester ^[1]^

cinnamyl alcohol,acetate ^[1]^

cinnamic acid,ethyl ester ^[1]^

benzoic acid,phenethyl ester ^[1]^

dibutyl phthalate ^[1]^

cinnamyl acetate ^[1]^

methqxy cinnamylacetate ^[1]^

trans-cinnamyl acetate ^[1]^

2-prepen-1-ol-3-phenyl-acetate ^[1]^

benzoic acid,phenylmethyl ester ^[1]^

benzoic-2-phenylethyl ester ^[1]^

1,2-benznendicarboxylic acid-bis(2-methoxyethyl) ester ^[1]^

5-oxopentanonate benzyl ^[1]^

o-hydroxycinnamic acid lactone ^[1]^

4-(4-methylphenyl)-4-pentenoi acid ethyl ester ^[1]^

farnesyl acetate ^[1]^

benzyl benzoate ^[1]^

phenylethyl benzoate ^[1]^

methyl palmitate ^[1]^

methyl petroselinate ^[1]^

methyl stearate ^[1]^

acetophenone ^[1]^

artemisia ketone ^[1]^

methyl heptenone ^[1]^

propiophenone ^[1]^

trans-chalcone ^[1]^

1,4-diphenyl-1,4-butanedione ^[1]^

1,4-diphenyl-butanedione ^[3]^

2-methoxyphenylacetone ^[1]^

2’-hydroxyacetophenone ^[1]^

3-methylacetophenone ^[1]^

1-phenyl-1,2-propanedione ^[1]^

1,10,14-trimethyl-2-pentade canone ^[1]^

2-ethyl-2-phenyl-1,3-dioxan-4,6-dion ^[1]^

ethanone,2’-hydroxy-1-phenyl ^[1]^

cyclohexanone,5-methyl-2-(1-methylethyl) ^[1]^

6-methy,5-hepten-2-one ^[1]^

6,10,14-trimethyl-2-pentadecanone ^[1]^

myristic acid ^[1]^

arachidic acid ^[1]^

(E)-9-octadecenoic acid ^[1]^

trans-cinnamic acid ^[1]^

tran-2-phenylcyclopropane-1-caboxylic acid ^[1]^

benzoic acid ^[1]^

hydro cinnamic acid ^[1]^

hexadecanoic acid ^[1]^

stearic acid ^[1]^

palmitic acid ^[1]^

3-phenylmethyl-2-propenoic acid ^[1]^

2-propenoic acid,3-(2-hydroxyphenyl) ^[1]^

9,12-octadecadienoic acid ^[1]^

tetradecanoic acid ^[1]^

2-phenylethyl ester,benzoic acid ^[1]^

pentadecanoic acid ^[1]^

oleic acid ^[1]^

cinnamic acid^[2,3]^

protocatechuic aldehyde^[2,4]^

protocatechuic acid^[2,4]^

coumarin^[2,3]^

harpagoside^[2]^

dihydrocinnacasside^[2]^

3,4,5-trimethoxypheny-1-β-D-glucopyranoside ^[2]^

O-coumaric acid glucoside^[2]^

β-sitosterol ^[3]^

(+)-syringaresinol ^[4]^

(+)-lyoniresinol ^[4]^

spicatolignan B^[4]^

(-)-secoisolariciresinol^[4]^

syringaldehyde^[3,4]^

vanillic acid^[4]^

ethyl protocatechuate^[4]^

syringic acid^[4]^

ethyl gallate^[4]^

cinnamyl alcohol^[2]^

2-(3',4'-dihydroxyphenyl)-1,3-pepperring-5-aldehyde ^[4]^

2-methoxy cinnamic acid^[3]^

ovafolinin B^[4]^

references:

1. Feng Xu , De-Jian W , Nan Z . Review on Chemical Components of Rimulus cinnamon Essential Oil[J]. Natural Product Research & Development, 2017.
2. Xi-Zhe L,Hai-Yan H,Wei Z,Yu-Chen Z,Zhong Y. Isolation and Identification of cassia twig Compounds and Their Neuroprotective Effects [J]. Journal of Shenyang Pharmaceutical University,2016,33(01):14-19.
3. Ping L , Li-Ping Z . Studies on Chemical Constituents and Cardiovascular Pharmacological Action of Cinnamomum cassia [J]. Liaoning Journal of Traditional Chinese Medicine, 2012.
4. Lingli Zhu, Zhifu Ai, Li Xu, Yongliang Jin, Chunling Liu, Feng Liu, Hua Liu. Isolation and Identification of Chemical Constituents from Ramulus Cinnamomi [J/OL]. Chin J Exp Tradit Med Form:1-5[2019-01-07].https://doi.org/10.13422/j.cnki.syfjx.20190712.

**Keywords for seeking chemical reactions related with** ***cassia twig* (Gui zhi) from KEGG:**

"Limonene","cubebene","ylangene","cadin","aromadendrene","isolongifolene","tetradecanal","benzylbenzoate",

"copaene","cyclohexene","styrene","pinene","elemene","fanesene","selinene","tetradecenal","acetophenone",

"vanillc acid","megastigma","curcumene","cadinene","bisabolene","bergamotene","camphene",

"benzeneacetaldehyde","octadecadienoic acid","Myrcene","capaene","cedrene","isocaryophyllene","muurolene",

"calamenene","hexanal","salicylal","tetradecanoic acid","menthene","isoermophilene","calacorene",

"calamene","bicyclo","tetramethyltricyclo","hyacinthin","coumarin","caryophyllene","methylethyl",

"cyclohexadiene","neoclovene","elixene","anisaldehyde","anisaldehyde","glucopyranoside","eremophilene",

"terpinene","guaiene","calamencne","alloaromadendrene","octanal","benzaldehyde","stearicacid","octatriene",

"methoxystyrene","octadecenal","cyclosativene","cinnamaldehyde","citral","farnesal","2-hydroxyphenyl", "santalene","farnesene","humulene","amorphene","spathulene","terpineol","hydroxybenzaldehyde",

"myristic acid","paeonol","globulol","cubenol","muurolol",

"cadinol","bisabolol","spathalenol","octadecanal","cyclohexanone","syringic acid","cyclohexanol","myrtenol",

"benzenpropanol","nerolidol","farnesol","palustrol","elemol","phenyl acetate","beta-sitosterol","cedrol","guaiol",

"propanol","cinnamyl alcohol","menthol","benzenepropanal","cinnamyl acetate","hexadecanoic acid",

"spathulenol","borneol","epicedrol","linalool","linderol","hexadecanol","torreyol","octadecenoic acid","secoisolariciresinol"

**Chemical compositions of Sijunzi Decoction**

ginsenoside ^[1,2,3]^

Glycyrrhizic acid ^[1,2,3,4]^

flavonoid^[1,3]^

Liquiritigenin ^[1,4]^

coumarin ^[1]^

Atractylenolide ^[2,3,4]^

panaxan ^[3]^

atractylodes macrocephalaon polysaccharide ^[3]^

pachymaran ^[3]^

Atractylon ^[3]^

β-Eucalyptus alcohol ^[3]^

gallic acid ^[3,4]^

Benzoic acid ^[3,4]^

Ferulic acid ^[3,4]^

Liquiritin ^[4]^

Lobetyolin ^[4]^

Calycosin ^[4]^

Isoliquiritigenin ^[4]^

Formononetin ^[4]^

Glycyrrhetinic acid ^[1,4]^

references:

1. Ru-Xia S,Wei-Dong X,Xiao-Qian X,Yue P. Analysis of the major metabolites of Sijunzi decoction in rat serum based on HPLC-MS [J]. Journal of Jiangsu University( Medicine Edition),2018,28(06):529-533.
2. Can-Can W,Li-Rong D,Min Z,Zhi-Bo G,Li Z,Xiu-Mei L. Simultaneous determination of seven chemical constituents in Sijunzi Decoction by UPLC [J]. Journal of Shenyang Pharmaceutical University,2018,35(08):639-645.
3. Hua L,Yan W,Qi-Wei L,Yu-Tong S,Qi Y,Li-Wei L. Discussion on the Main Effective Components and Systematic Biology of Sijunzi Decoction for Supplementing Spleen and Benefiting Qi [J]. Acta Chinese Medicine and Pharmacology,2017,45(06):96-99.
4. Zhang Y, Huang X, Xie Y, et al. Application of ultra-high performance liquid chromatography (UPLC) with photodiode array detector for the simultaneous determination of 12 chemical components of traditional Chinese Si-Jun-Zi-Tang formula[J]. Journal of Medicinal Plants Research, 2011, 5(10): 1955-1961.

**Keywords for seeking chemical reactions related with Sijunzi *Decoction* from KEGG:**

"ginsenoside", "Glycyrrhizic acid", "flavonoid", "Liquiritigenin", "Inositol phosphate", "coumarin", "Atractylenolide", "panaxan", "pachymaran", "Atractylon", "Eucalyptus alcohol","gallic acid","Benzoic acid", "Ferulic acid", "Liquiritin", "Lobetyolin", "Calycosin","Isoliquiritigenin", "Formononetin", "Glycyrrhetinic acid"
