# Supplementary material for: The impact of Traditional Chinese Medicine on mouse gut microbiota abundances and interactions based on Granger causality and pathway analysis
Source: Front Microbiol. 2022 Nov 11;13:980082. doi: 10.3389/fmicb.2022.980082 (PMC9692106; doi:10.3389/fmicb.2022.980082)

Table S1: 28 Species annotated in our paper and their corresponding items in KEGG.

| Species among 70 annotated | corresponding name in KEGG |
| --- | --- |
| Bifidobacterium pseudolongum | Bifidobacterium pseudolongum PV8-2 |
| Adlercreutzia_equolifaciens | Adlercreutzia equolifaciens DSM 19450 |
| Bacteroides_caccae | Bacteroides caccae ATCC 43185 |
| Bacteroides_caecimuris | Bacteroides caecimuris I48 |
| Bacteroides_intestinalis | Bacteroides intestinalis APC919/174 |
| Bacteroides_nordii | Bacteroides nordii FDAARGOS_1461 |
| Bacteroides_thetaiotaomicron | Bacteroides thetaiotaomicron VPI-5482 |
| Bacteroides_uniformis | Bacteroides uniformis NBRC 113350 |
| Bacteroides_vulgatus | Phocaeicola vulgatus ATCC 8482 |
| Bacteroides_xylanisolvens | Bacteroides xylanisolvens XB1A |
| Muribaculum_intestinale | Muribaculum intestinale YL27 |
| Butyricimonas_virosa | Butyricimonas virosa FDAARGOS_1229 |
| Alistipes_indistinctus | Alistipes indistinctus 2BBH45 |
| Alistipes_shahii | Alistipes shahii WAL 8301 |
| Parabacteroides_distasonis | Parabacteroides distasonis ATCC 8503 |
| Parabacteroides_goldsteinii | Parabacteroides goldsteinii BFG-241 |
| Parabacteroides_johnsonii | Parabacteroides johnsonii FDAARGOS_1580 |
| Parabacteroides_merdae | Parabacteroides merdae CL06T03C08 |
| Lactobacillus_johnsonii | Lactobacillus johnsonii NCC 533 |
| Lactobacillus_reuteri | Lactobacillus reuteri DSM 20016 |
| Intestinimonas_butyriciproducens | Intestinimonas butyriciproducens AF211 |
| Flavonifractor_plautii | Flavonifractor plautii YL31 |
| Clostridium_innocuum | Clostridium innocuum LC-LUMC-CI-001 |
| Erysipelothrix_larvae | Erysipelothrix larvae LV19 |
| Faecalibaculum_rodentium | Faecalibaculum rodentium ALO17 |
| Escherichia_coli | Escherichia coli K-12 MG1655 |
| Escherichia_marmotae | Escherichia marmotae HT073016 |
| Akkermansia_muciniphila | Akkermansia muciniphila ATCC BAA-835 |

Table S2 The species which abundance are increased by 11 treatments where p<0.05

| blank | ['L_bacterium_28_4', 'A_muciniphila'] |
| --- | --- |
| DangguiBuxue Decoction | M_bacterium_DSM_103720  P_distasonis  P_goldsteinii  P_excrementihominis  D_bacterium  P_bacterium_CAG_139 |
| *Codonopsis pilosula* (Dang shen) | B_dorei  B_vulgatus  A_shahii  P_distasonis  P_goldsteinii  L_bacterium_A4  L_bacterium_COE1 |
| *Poria cocos*(Fu ling) | R_lactatiformans  R_ilealis  P_johnsonii  P_goldsteinii  L_vaginalis  L_intestinalis  L_bacterium_A2  L_bacterium_3_2  L_bacterium_3_1  I_butyriciproducens  H_acetispora  F_plautii  E_sp_14_2  E_plexicaudatum  E_marmotae  E_coli  E_caecimuris  D_sp_5_2  C_sp_ASF502  C_sp_ASF356  C_innocuum  C_cocleatum  C_bolteae  B_xylanisolvens  B_wadsworthia  B_virosa  B_thetaiotaomicron  B_nordii  B_intestinalis  B_dorei  B_coccoides  Acu_muris  A_muris  A_indistinctus  A_equolifaciens  A_celatus |
| *rhizoma zingiberis* (Gan jiang) | A_celatus  E_caecimuris  B_intestinalis  B_nordii  B_virosa  P_sp_MGM2  A_indistinctus  A_shahii  P_distasonis  P_goldsteinii  P_johnsonii  L_vaginalis  I_butyriciproducens  E_plexicaudatum  E_sp_14_2  A_muris  B_coccoides  D_sp_5_2  C_bolteae  L_bacterium_28_4  L_bacterium_3_1  L_bacterium_3_2  L_bacterium_A2  R_ilealis  F_plautii  H_acetispora  R_lactatiformans  D_newyorkensis  C_innocuum  B_wadsworthia  E_coli  E_marmotae |
| *Cassia twig* (Gui zhi) | L_murinus  L_bacterium_A4  T_muris |
| *Mangnolia officinalis* (Hou po) | B_dorei  B_faecichinchillae  B_vulgatus  P_sp_MGM2  A_shahii  P_distasonis  P_merdae  D_bacterium |
| *Radices saussureae* (Mu xiang) | B_dorei  B_uniformis  P_distasonis  D_sp_5_2 |
| saline | B_vulgatus  L_reuteri  D_sp_5_2  L_bacterium_A4  Acu_muris  D_newyorkensis  D_bacterium |
| *Rhizoma Dioscoreae* (Shan yao) | B_virosa  C_sp_ASF502  D_sp_5_2  L_bacterium_28_4  L_bacterium_A4 |
| Sijunzi Decoction | B_pseudolongum |

**Invsimpson index for alpha diversity index**

| 7.700824 4.81645 5.842102 6.979517 4.279513 |
| --- |
| 6.017031 5.715991 5.969135 5.837191 6.045832 |
| 6.548915 6.035612 5.710265 4.011883 5.366101 |
| 7.965627 5.796925 6.684149 6.824323 4.460092 |
| 5.508136 6.464553 2.667523 4.419043 6.207651 |
| 5.491767 6.213575 4.065987 5.677088 4.163429 |
| 6.858053 6.57359 6.638729 7.314943 6.466898 |
| 6.352312 6.787451 7.207396 6.89669 5.773753 |
| 8.894207 6.007613 3.86633 3.23251 4.118428 |
| 6.010573 4.821782 5.171265 6.435095 6.481111 |
| 7.627127 6.145556 4.811807 7.842954 7.873881 |

**Simpson index for alpha diversity index**

| 0.8701438 0.7923782 0.8288287 0.8567236 0.7663285 |
| --- |
| 0.8338051 0.8250522 0.8324715 0.8286847 0.8345968 |
| 0.847303 0.8343167 0.8248768 0.7507405 0.813645 |
| 0.8744606 0.8274947 0.8503923 0.8534653 0.7757894 |
| 0.8184504 0.8453103 0.6251204 0.7737066 0.8389085 |
| 0.8179092 0.839062 0.7540573 0.8238534 0.7598133 |
| 0.854186 0.8478761 0.8493688 0.8632935 0.8453663 |
| 0.842577 0.8526693 0.8612536 0.8550029 0.8268024 |
| 0.8875673 0.8335445 0.7413568 0.6906428 0.7571889 |
| 0.8336265 0.7926078 0.8066237 0.8446021 0.8457055 |
| 0.868889 0.8372808 0.7921779 0.872497 0.8729978 |

The correlation between Alpha diversity Shannon, simpson and invsimpson index


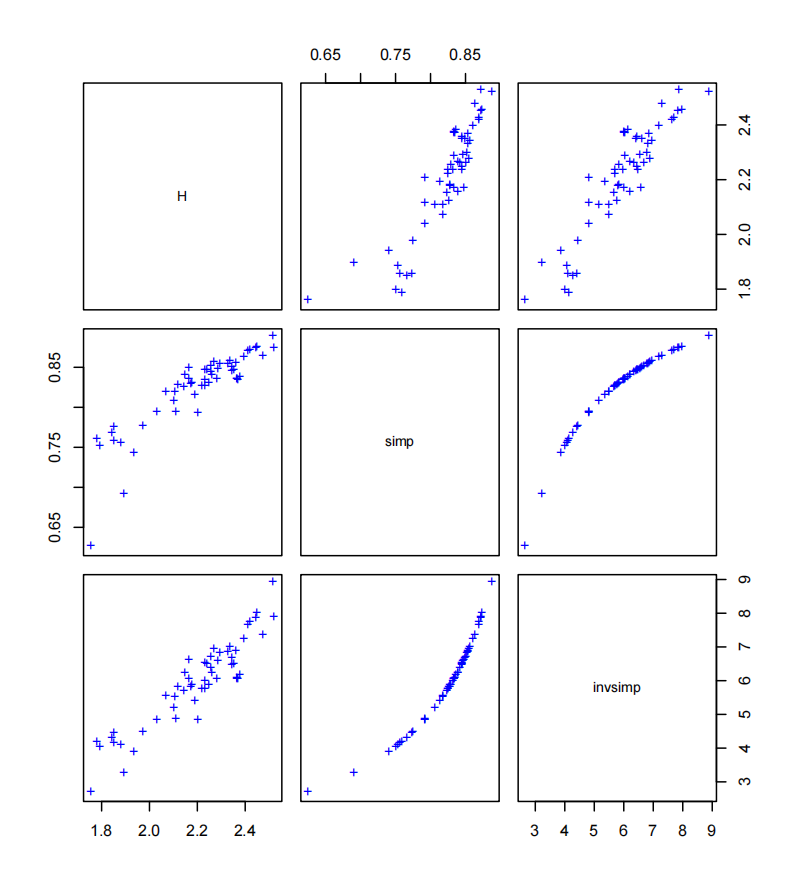

Supplement: Supplementary file 2 [file Table_2.DOC]
