# Supplementary material for: The impact of Traditional Chinese Medicine on mouse gut microbiota abundances and interactions based on Granger causality and pathway analysis
Source: Front Microbiol. 2022 Nov 11;13:980082. doi: 10.3389/fmicb.2022.980082 (PMC9692106; doi:10.3389/fmicb.2022.980082)
Supplement: Supplementary file 3 [file Table_3.doc]

rn:R01703 { Hexadecanoic acid } <-- Hydrogen peroxide -- rn:R00069 <-- 4-Imidazolone-5-propanoate --> rn:R02288

rn:R01703 { Hexadecanoic acid } <-- Hydrogen peroxide -- rn:R00069 <-- 4-Imidazolone-5-propanoate --> rn:R02914

rn:R01703 { Hexadecanoic acid } <-- Hydrogen peroxide -- rn:R00069 <-- 4-Imidazolone-5-propanoate --> rn:R04282

rn:R01703 { Hexadecanoic acid } <-- Hydrogen peroxide -- rn:R00069 <-- 4-Imidazolone-5-propanoate --> rn:R04283

rn:R05136 { Salicylaldehyde } <-- Pyruvate -- rn:R00210 <-- Acetyl-CoA --> rn:R00693

rn:R05136 { Salicylaldehyde } <-- Pyruvate -- rn:R00210 <-- Acetyl-CoA --> rn:R02152

rn:R05136 { Salicylaldehyde } <-- Pyruvate -- rn:R00210 <-- Acetyl-CoA --> rn:R02911

rn:R05136 { Salicylaldehyde } <-- Pyruvate -- rn:R00210 <-- Acetyl-CoA --> rn:R02955

rn:R05136 { Salicylaldehyde } <-- Pyruvate -- rn:R00210 <-- Acetyl-CoA --> rn:R03153

rn:R05136 { Salicylaldehyde } <-- Pyruvate -- rn:R00210 <-- Acetyl-CoA --> rn:R03903

rn:R05136 { Salicylaldehyde } <-- Pyruvate -- rn:R00210 <-- Acetyl-CoA --> rn:R04950

rn:R05136 { Salicylaldehyde } <-- Pyruvate -- rn:R00210 <-- Acetyl-CoA --> rn:R05509

rn:R05136 { Salicylaldehyde } <-- Pyruvate -- rn:R00210 <-- Acetyl-CoA --> rn:R07253

rn:R05136 { Salicylaldehyde } <-- Pyruvate -- rn:R00210 <-- Acetyl-CoA --> rn:R07937

rn:R05136 { Salicylaldehyde } <-- Pyruvate -- rn:R00210 <-- Acetyl-CoA --> rn:R07953

rn:R05136 { Salicylaldehyde } <-- Pyruvate -- rn:R00210 <-- Acetyl-CoA --> rn:R08036

rn:R05136 { Salicylaldehyde } <-- Pyruvate -- rn:R00210 <-- Acetyl-CoA --> rn:R08870

rn:R05136 { Salicylaldehyde } <-- Pyruvate -- rn:R00210 <-- Acetyl-CoA --> rn:R08871

rn:R05136 { Salicylaldehyde } <-- Pyruvate -- rn:R00210 <-- Acetyl-CoA --> rn:R08938

rn:R05136 { Salicylaldehyde } <-- Pyruvate -- rn:R00210 <-- Acetyl-CoA --> rn:R10500

rn:R05136 { Salicylaldehyde } <-- Pyruvate -- rn:R00210 <-- Acetyl-CoA --> rn:R10600

rn:R05136 { Salicylaldehyde } <-- Pyruvate -- rn:R00210 <-- Acetyl-CoA --> rn:R10745

rn:R05136 { Salicylaldehyde } <-- Pyruvate -- rn:R00210 <-- Acetyl-CoA --> rn:R10746

rn:R05136 { Salicylaldehyde } <-- Pyruvate -- rn:R00210 <-- Acetyl-CoA --> rn:R10893

rn:R05136 { Salicylaldehyde } <-- Pyruvate -- rn:R00210 <-- Acetyl-CoA --> rn:R11124

rn:R05136 { Salicylaldehyde } <-- Pyruvate -- rn:R00210 <-- Acetyl-CoA --> rn:R11125

rn:R05136 { Salicylaldehyde } <-- Pyruvate -- rn:R00210 <-- Acetyl-CoA --> rn:R11479

rn:R05136 { Salicylaldehyde } <-- Pyruvate -- rn:R00210 <-- Acetyl-CoA --> rn:R11708

rn:R05136 { Salicylaldehyde } <-- Pyruvate -- rn:R00210 <-- Acetyl-CoA --> rn:R11902

rn:R01703 { Hexadecanoic acid } <-- Hydrogen peroxide -- rn:R00274 <-- Glutathione disulfide --> rn:R01111

rn:R01703 { Hexadecanoic acid } <-- Hydrogen peroxide -- rn:R00274 <-- Glutathione disulfide --> rn:R03915

rn:R01703 { Hexadecanoic acid } <-- Hydrogen peroxide -- rn:R00274 <-- Glutathione disulfide --> rn:R03984

rn:R01703 { Hexadecanoic acid } <-- Hydrogen peroxide -- rn:R00274 <-- Glutathione disulfide --> rn:R04039

rn:R01703 { Hexadecanoic acid } <-- Hydrogen peroxide -- rn:R00274 <-- Glutathione disulfide --> rn:R05267

rn:R01703 { Hexadecanoic acid } <-- Hydrogen peroxide -- rn:R00274 <-- Glutathione disulfide --> rn:R05717

rn:R02234 { Cyclohexanone } <-- Acceptor -- rn:R00283 <-- Hydroxylamine --> rn:R01485

rn:R03212 { 3-Hydroxycyclohexanone } <-- Acceptor -- rn:R00283 <-- Hydroxylamine --> rn:R01485

rn:R02234 { Cyclohexanone } <-- Acceptor -- rn:R00283 <-- Hydroxylamine --> rn:R10164

rn:R03212 { 3-Hydroxycyclohexanone } <-- Acceptor -- rn:R00283 <-- Hydroxylamine --> rn:R10164

rn:R02234 { Cyclohexanone } <-- Acceptor -- rn:R00283 <-- Hydroxylamine --> rn:R10165

rn:R03212 { 3-Hydroxycyclohexanone } <-- Acceptor -- rn:R00283 <-- Hydroxylamine --> rn:R10165

rn:R02234 { Cyclohexanone } <-- Acceptor -- rn:R00283 <-- Hydroxylamine --> rn:R10230

rn:R03212 { 3-Hydroxycyclohexanone } <-- Acceptor -- rn:R00283 <-- Hydroxylamine --> rn:R10230

rn:R02234 { Cyclohexanone } <-- Acceptor -- rn:R00283 <-- Hydroxylamine --> rn:R10913

rn:R03212 { 3-Hydroxycyclohexanone } <-- Acceptor -- rn:R00283 <-- Hydroxylamine --> rn:R10913

rn:R02234 { Cyclohexanone } <-- Acceptor -- rn:R00284 <-- Hydroxylamine --> rn:R01485

rn:R03212 { 3-Hydroxycyclohexanone } <-- Acceptor -- rn:R00284 <-- Hydroxylamine --> rn:R01485

rn:R02234 { Cyclohexanone } <-- Acceptor -- rn:R00284 <-- Hydroxylamine --> rn:R10164

rn:R03212 { 3-Hydroxycyclohexanone } <-- Acceptor -- rn:R00284 <-- Hydroxylamine --> rn:R10164

rn:R02234 { Cyclohexanone } <-- Acceptor -- rn:R00284 <-- Hydroxylamine --> rn:R10165

rn:R03212 { 3-Hydroxycyclohexanone } <-- Acceptor -- rn:R00284 <-- Hydroxylamine --> rn:R10165

rn:R02234 { Cyclohexanone } <-- Acceptor -- rn:R00284 <-- Hydroxylamine --> rn:R10230

rn:R03212 { 3-Hydroxycyclohexanone } <-- Acceptor -- rn:R00284 <-- Hydroxylamine --> rn:R10230

rn:R02234 { Cyclohexanone } <-- Acceptor -- rn:R00284 <-- Hydroxylamine --> rn:R10913

rn:R03212 { 3-Hydroxycyclohexanone } <-- Acceptor -- rn:R00284 <-- Hydroxylamine --> rn:R10913

rn:R02468 { (-)-Limonene } <-- [Reduced NADPH---hemoprotein reductase] -- rn:R00311 <-- Fe2+ --> rn:R00310

rn:R02469 { (-)-Limonene } <-- [Reduced NADPH---hemoprotein reductase] -- rn:R00311 <-- Fe2+ --> rn:R00310

rn:R02470 { (-)-Limonene } <-- [Reduced NADPH---hemoprotein reductase] -- rn:R00311 <-- Fe2+ --> rn:R00310

rn:R04366 { Linalool } <-- [Reduced NADPH---hemoprotein reductase] -- rn:R00311 <-- Fe2+ --> rn:R00310

rn:R06119 { d-Limonene } <-- [Reduced NADPH---hemoprotein reductase] -- rn:R00311 <-- Fe2+ --> rn:R00310

rn:R09451 { Hexadecanoic acid } <-- [Reduced NADPH---hemoprotein reductase] -- rn:R00311 <-- Fe2+ --> rn:R00310

rn:R09452 { (9Z)-Octadecenoic acid } <-- [Reduced NADPH---hemoprotein reductase] -- rn:R00311 <-- Fe2+ --> rn:R00310

rn:R09922 { (+)-Linalool } <-- [Reduced NADPH---hemoprotein reductase] -- rn:R00311 <-- Fe2+ --> rn:R00310

rn:R09923 { (-)-Linalool } <-- [Reduced NADPH---hemoprotein reductase] -- rn:R00311 <-- Fe2+ --> rn:R00310

rn:R09925 { Linalool } <-- [Reduced NADPH---hemoprotein reductase] -- rn:R00311 <-- Fe2+ --> rn:R00310

rn:R09934 { Humulene } <-- [Reduced NADPH---hemoprotein reductase] -- rn:R00311 <-- Fe2+ --> rn:R00310

rn:R10562 { (E,E)-Geranyllinalool } <-- [Reduced NADPH---hemoprotein reductase] -- rn:R00311 <-- Fe2+ --> rn:R00310

rn:R11055 { Myrcene } <-- [Reduced NADPH---hemoprotein reductase] -- rn:R00311 <-- Fe2+ --> rn:R00310

rn:R02468 { (-)-Limonene } <-- [Reduced NADPH---hemoprotein reductase] -- rn:R00311 <-- Biliverdin --> rn:R05817

rn:R02469 { (-)-Limonene } <-- [Reduced NADPH---hemoprotein reductase] -- rn:R00311 <-- Biliverdin --> rn:R05817

rn:R02470 { (-)-Limonene } <-- [Reduced NADPH---hemoprotein reductase] -- rn:R00311 <-- Biliverdin --> rn:R05817

rn:R04366 { Linalool } <-- [Reduced NADPH---hemoprotein reductase] -- rn:R00311 <-- Biliverdin --> rn:R05817

rn:R06119 { d-Limonene } <-- [Reduced NADPH---hemoprotein reductase] -- rn:R00311 <-- Biliverdin --> rn:R05817

rn:R09451 { Hexadecanoic acid } <-- [Reduced NADPH---hemoprotein reductase] -- rn:R00311 <-- Biliverdin --> rn:R05817

rn:R09452 { (9Z)-Octadecenoic acid } <-- [Reduced NADPH---hemoprotein reductase] -- rn:R00311 <-- Biliverdin --> rn:R05817

rn:R09922 { (+)-Linalool } <-- [Reduced NADPH---hemoprotein reductase] -- rn:R00311 <-- Biliverdin --> rn:R05817

rn:R09923 { (-)-Linalool } <-- [Reduced NADPH---hemoprotein reductase] -- rn:R00311 <-- Biliverdin --> rn:R05817

rn:R09925 { Linalool } <-- [Reduced NADPH---hemoprotein reductase] -- rn:R00311 <-- Biliverdin --> rn:R05817

rn:R09934 { Humulene } <-- [Reduced NADPH---hemoprotein reductase] -- rn:R00311 <-- Biliverdin --> rn:R05817

rn:R10562 { (E,E)-Geranyllinalool } <-- [Reduced NADPH---hemoprotein reductase] -- rn:R00311 <-- Biliverdin --> rn:R05817

rn:R11055 { Myrcene } <-- [Reduced NADPH---hemoprotein reductase] -- rn:R00311 <-- Biliverdin --> rn:R05817

rn:R02468 { (-)-Limonene } <-- [Reduced NADPH---hemoprotein reductase] -- rn:R00311 <-- Biliverdin --> rn:R05818

rn:R02469 { (-)-Limonene } <-- [Reduced NADPH---hemoprotein reductase] -- rn:R00311 <-- Biliverdin --> rn:R05818

rn:R02470 { (-)-Limonene } <-- [Reduced NADPH---hemoprotein reductase] -- rn:R00311 <-- Biliverdin --> rn:R05818

rn:R04366 { Linalool } <-- [Reduced NADPH---hemoprotein reductase] -- rn:R00311 <-- Biliverdin --> rn:R05818

rn:R06119 { d-Limonene } <-- [Reduced NADPH---hemoprotein reductase] -- rn:R00311 <-- Biliverdin --> rn:R05818

rn:R09451 { Hexadecanoic acid } <-- [Reduced NADPH---hemoprotein reductase] -- rn:R00311 <-- Biliverdin --> rn:R05818

rn:R09452 { (9Z)-Octadecenoic acid } <-- [Reduced NADPH---hemoprotein reductase] -- rn:R00311 <-- Biliverdin --> rn:R05818

rn:R09922 { (+)-Linalool } <-- [Reduced NADPH---hemoprotein reductase] -- rn:R00311 <-- Biliverdin --> rn:R05818

rn:R09923 { (-)-Linalool } <-- [Reduced NADPH---hemoprotein reductase] -- rn:R00311 <-- Biliverdin --> rn:R05818

rn:R09925 { Linalool } <-- [Reduced NADPH---hemoprotein reductase] -- rn:R00311 <-- Biliverdin --> rn:R05818

rn:R09934 { Humulene } <-- [Reduced NADPH---hemoprotein reductase] -- rn:R00311 <-- Biliverdin --> rn:R05818

rn:R10562 { (E,E)-Geranyllinalool } <-- [Reduced NADPH---hemoprotein reductase] -- rn:R00311 <-- Biliverdin --> rn:R05818

rn:R11055 { Myrcene } <-- [Reduced NADPH---hemoprotein reductase] -- rn:R00311 <-- Biliverdin --> rn:R05818

rn:R02468 { (-)-Limonene } <-- [Reduced NADPH---hemoprotein reductase] -- rn:R00311 <-- [Oxidized NADPH---hemoprotein reductase] --> rn:R08551

rn:R02469 { (-)-Limonene } <-- [Reduced NADPH---hemoprotein reductase] -- rn:R00311 <-- [Oxidized NADPH---hemoprotein reductase] --> rn:R08551

rn:R02470 { (-)-Limonene } <-- [Reduced NADPH---hemoprotein reductase] -- rn:R00311 <-- [Oxidized NADPH---hemoprotein reductase] --> rn:R08551

rn:R04366 { Linalool } <-- [Reduced NADPH---hemoprotein reductase] -- rn:R00311 <-- [Oxidized NADPH---hemoprotein reductase] --> rn:R08551

rn:R06119 { d-Limonene } <-- [Reduced NADPH---hemoprotein reductase] -- rn:R00311 <-- [Oxidized NADPH---hemoprotein reductase] --> rn:R08551

rn:R09451 { Hexadecanoic acid } <-- [Reduced NADPH---hemoprotein reductase] -- rn:R00311 <-- [Oxidized NADPH---hemoprotein reductase] --> rn:R08551

rn:R09452 { (9Z)-Octadecenoic acid } <-- [Reduced NADPH---hemoprotein reductase] -- rn:R00311 <-- [Oxidized NADPH---hemoprotein reductase] --> rn:R08551

rn:R09922 { (+)-Linalool } <-- [Reduced NADPH---hemoprotein reductase] -- rn:R00311 <-- [Oxidized NADPH---hemoprotein reductase] --> rn:R08551

rn:R09923 { (-)-Linalool } <-- [Reduced NADPH---hemoprotein reductase] -- rn:R00311 <-- [Oxidized NADPH---hemoprotein reductase] --> rn:R08551

rn:R09925 { Linalool } <-- [Reduced NADPH---hemoprotein reductase] -- rn:R00311 <-- [Oxidized NADPH---hemoprotein reductase] --> rn:R08551

rn:R09934 { Humulene } <-- [Reduced NADPH---hemoprotein reductase] -- rn:R00311 <-- [Oxidized NADPH---hemoprotein reductase] --> rn:R08551

rn:R10562 { (E,E)-Geranyllinalool } <-- [Reduced NADPH---hemoprotein reductase] -- rn:R00311 <-- [Oxidized NADPH---hemoprotein reductase] --> rn:R08551

rn:R11055 { Myrcene } <-- [Reduced NADPH---hemoprotein reductase] -- rn:R00311 <-- [Oxidized NADPH---hemoprotein reductase] --> rn:R08551

rn:R02468 { (-)-Limonene } <-- [Reduced NADPH---hemoprotein reductase] -- rn:R00311 <-- Fe2+ --> rn:R09541

rn:R02469 { (-)-Limonene } <-- [Reduced NADPH---hemoprotein reductase] -- rn:R00311 <-- Fe2+ --> rn:R09541

rn:R02470 { (-)-Limonene } <-- [Reduced NADPH---hemoprotein reductase] -- rn:R00311 <-- Fe2+ --> rn:R09541

rn:R04366 { Linalool } <-- [Reduced NADPH---hemoprotein reductase] -- rn:R00311 <-- Fe2+ --> rn:R09541

rn:R06119 { d-Limonene } <-- [Reduced NADPH---hemoprotein reductase] -- rn:R00311 <-- Fe2+ --> rn:R09541

rn:R09451 { Hexadecanoic acid } <-- [Reduced NADPH---hemoprotein reductase] -- rn:R00311 <-- Fe2+ --> rn:R09541

rn:R09452 { (9Z)-Octadecenoic acid } <-- [Reduced NADPH---hemoprotein reductase] -- rn:R00311 <-- Fe2+ --> rn:R09541

rn:R09922 { (+)-Linalool } <-- [Reduced NADPH---hemoprotein reductase] -- rn:R00311 <-- Fe2+ --> rn:R09541

rn:R09923 { (-)-Linalool } <-- [Reduced NADPH---hemoprotein reductase] -- rn:R00311 <-- Fe2+ --> rn:R09541

rn:R09925 { Linalool } <-- [Reduced NADPH---hemoprotein reductase] -- rn:R00311 <-- Fe2+ --> rn:R09541

rn:R09934 { Humulene } <-- [Reduced NADPH---hemoprotein reductase] -- rn:R00311 <-- Fe2+ --> rn:R09541

rn:R10562 { (E,E)-Geranyllinalool } <-- [Reduced NADPH---hemoprotein reductase] -- rn:R00311 <-- Fe2+ --> rn:R09541

rn:R11055 { Myrcene } <-- [Reduced NADPH---hemoprotein reductase] -- rn:R00311 <-- Fe2+ --> rn:R09541

rn:R02468 { (-)-Limonene } <-- [Reduced NADPH---hemoprotein reductase] -- rn:R00311 <-- CO --> rn:R11168

rn:R02469 { (-)-Limonene } <-- [Reduced NADPH---hemoprotein reductase] -- rn:R00311 <-- CO --> rn:R11168

rn:R02470 { (-)-Limonene } <-- [Reduced NADPH---hemoprotein reductase] -- rn:R00311 <-- CO --> rn:R11168

rn:R04366 { Linalool } <-- [Reduced NADPH---hemoprotein reductase] -- rn:R00311 <-- CO --> rn:R11168

rn:R06119 { d-Limonene } <-- [Reduced NADPH---hemoprotein reductase] -- rn:R00311 <-- CO --> rn:R11168

rn:R09451 { Hexadecanoic acid } <-- [Reduced NADPH---hemoprotein reductase] -- rn:R00311 <-- CO --> rn:R11168

rn:R09452 { (9Z)-Octadecenoic acid } <-- [Reduced NADPH---hemoprotein reductase] -- rn:R00311 <-- CO --> rn:R11168

rn:R09922 { (+)-Linalool } <-- [Reduced NADPH---hemoprotein reductase] -- rn:R00311 <-- CO --> rn:R11168

rn:R09923 { (-)-Linalool } <-- [Reduced NADPH---hemoprotein reductase] -- rn:R00311 <-- CO --> rn:R11168

rn:R09925 { Linalool } <-- [Reduced NADPH---hemoprotein reductase] -- rn:R00311 <-- CO --> rn:R11168

rn:R09934 { Humulene } <-- [Reduced NADPH---hemoprotein reductase] -- rn:R00311 <-- CO --> rn:R11168

rn:R10562 { (E,E)-Geranyllinalool } <-- [Reduced NADPH---hemoprotein reductase] -- rn:R00311 <-- CO --> rn:R11168

rn:R11055 { Myrcene } <-- [Reduced NADPH---hemoprotein reductase] -- rn:R00311 <-- CO --> rn:R11168

rn:R02234 { Cyclohexanone } <-- Acceptor -- rn:R00544 <-- Carboxylate --> rn:R09930

rn:R03212 { 3-Hydroxycyclohexanone } <-- Acceptor -- rn:R00544 <-- Carboxylate --> rn:R09930

rn:R02234 { Cyclohexanone } <-- Acceptor -- rn:R00639 <-- Aldehyde --> rn:R00634

rn:R03212 { 3-Hydroxycyclohexanone } <-- Acceptor -- rn:R00639 <-- Aldehyde --> rn:R00634

rn:R02234 { Cyclohexanone } <-- Acceptor -- rn:R00639 <-- Aldehyde --> rn:R10911

rn:R03212 { 3-Hydroxycyclohexanone } <-- Acceptor -- rn:R00639 <-- Aldehyde --> rn:R10911

rn:R02234 { Cyclohexanone } <-- Acceptor -- rn:R00639 <-- Aldehyde --> rn:R10912

rn:R03212 { 3-Hydroxycyclohexanone } <-- Acceptor -- rn:R00639 <-- Aldehyde --> rn:R10912

rn:R01703 { Hexadecanoic acid } <-- Hydrogen peroxide -- rn:R00644 <-- Dehydroascorbate --> rn:R01108

rn:R01703 { Hexadecanoic acid } <-- Hydrogen peroxide -- rn:R00644 <-- Dehydroascorbate --> rn:R04785

rn:R01703 { Hexadecanoic acid } <-- Hydrogen peroxide -- rn:R00644 <-- Dehydroascorbate --> rn:R08358

rn:R11068 { 3-[(1R,2S,5R,6S)-5-Hydroxy-7-oxabicyclo[4.1.0]heptan-2-yl]-2-oxopropanoate } <-- L-Phenylalanine -- rn:R00686 <-- D-Phenylalanine --> rn:R01374

rn:R11068 { 3-[(1R,2S,5R,6S)-5-Hydroxy-7-oxabicyclo[4.1.0]heptan-2-yl]-2-oxopropanoate } <-- L-Phenylalanine -- rn:R00686 <-- AMP --> rn:R01490

rn:R11068 { 3-[(1R,2S,5R,6S)-5-Hydroxy-7-oxabicyclo[4.1.0]heptan-2-yl]-2-oxopropanoate } <-- L-Phenylalanine -- rn:R00686 <-- D-Phenylalanine --> rn:R01582

rn:R11068 { 3-[(1R,2S,5R,6S)-5-Hydroxy-7-oxabicyclo[4.1.0]heptan-2-yl]-2-oxopropanoate } <-- L-Phenylalanine -- rn:R00686 <-- D-Phenylalanine --> rn:R03903

rn:R11068 { 3-[(1R,2S,5R,6S)-5-Hydroxy-7-oxabicyclo[4.1.0]heptan-2-yl]-2-oxopropanoate } <-- L-Phenylalanine -- rn:R00686 <-- AMP --> rn:R05717

rn:R11068 { 3-[(1R,2S,5R,6S)-5-Hydroxy-7-oxabicyclo[4.1.0]heptan-2-yl]-2-oxopropanoate } <-- L-Phenylalanine -- rn:R00686 <-- AMP --> rn:R08743

rn:R11068 { 3-[(1R,2S,5R,6S)-5-Hydroxy-7-oxabicyclo[4.1.0]heptan-2-yl]-2-oxopropanoate } <-- L-Phenylalanine -- rn:R00686 <-- AMP --> rn:R11679

rn:R11068 { 3-[(1R,2S,5R,6S)-5-Hydroxy-7-oxabicyclo[4.1.0]heptan-2-yl]-2-oxopropanoate } <-- L-Phenylalanine -- rn:R00688 <-- Phenylpyruvate --> rn:R00695

rn:R11068 { 3-[(1R,2S,5R,6S)-5-Hydroxy-7-oxabicyclo[4.1.0]heptan-2-yl]-2-oxopropanoate } <-- L-Phenylalanine -- rn:R00688 <-- Phenylpyruvate --> rn:R01372

rn:R11068 { 3-[(1R,2S,5R,6S)-5-Hydroxy-7-oxabicyclo[4.1.0]heptan-2-yl]-2-oxopropanoate } <-- L-Phenylalanine -- rn:R00688 <-- Phenylpyruvate --> rn:R01375

rn:R11068 { 3-[(1R,2S,5R,6S)-5-Hydroxy-7-oxabicyclo[4.1.0]heptan-2-yl]-2-oxopropanoate } <-- L-Phenylalanine -- rn:R00688 <-- Phenylpyruvate --> rn:R01376

rn:R11068 { 3-[(1R,2S,5R,6S)-5-Hydroxy-7-oxabicyclo[4.1.0]heptan-2-yl]-2-oxopropanoate } <-- L-Phenylalanine -- rn:R00688 <-- Phenylpyruvate --> rn:R01377

rn:R11068 { 3-[(1R,2S,5R,6S)-5-Hydroxy-7-oxabicyclo[4.1.0]heptan-2-yl]-2-oxopropanoate } <-- L-Phenylalanine -- rn:R00688 <-- Phenylpyruvate --> rn:R01378

rn:R11068 { 3-[(1R,2S,5R,6S)-5-Hydroxy-7-oxabicyclo[4.1.0]heptan-2-yl]-2-oxopropanoate } <-- L-Phenylalanine -- rn:R00688 <-- Phenylpyruvate --> rn:R10431

rn:R11068 { 3-[(1R,2S,5R,6S)-5-Hydroxy-7-oxabicyclo[4.1.0]heptan-2-yl]-2-oxopropanoate } <-- L-Phenylalanine -- rn:R00688 <-- Phenylpyruvate --> rn:R10500

rn:R11068 { 3-[(1R,2S,5R,6S)-5-Hydroxy-7-oxabicyclo[4.1.0]heptan-2-yl]-2-oxopropanoate } <-- L-Phenylalanine -- rn:R00689 <-- Hydrogen peroxide --> rn:R00069

rn:R11068 { 3-[(1R,2S,5R,6S)-5-Hydroxy-7-oxabicyclo[4.1.0]heptan-2-yl]-2-oxopropanoate } <-- L-Phenylalanine -- rn:R00689 <-- Hydrogen peroxide --> rn:R00113

rn:R11068 { 3-[(1R,2S,5R,6S)-5-Hydroxy-7-oxabicyclo[4.1.0]heptan-2-yl]-2-oxopropanoate } <-- L-Phenylalanine -- rn:R00689 <-- Hydrogen peroxide --> rn:R00274

rn:R11068 { 3-[(1R,2S,5R,6S)-5-Hydroxy-7-oxabicyclo[4.1.0]heptan-2-yl]-2-oxopropanoate } <-- L-Phenylalanine -- rn:R00689 <-- Hydrogen peroxide --> rn:R00644

rn:R11068 { 3-[(1R,2S,5R,6S)-5-Hydroxy-7-oxabicyclo[4.1.0]heptan-2-yl]-2-oxopropanoate } <-- L-Phenylalanine -- rn:R00689 <-- Phenylpyruvate --> rn:R00695

rn:R11068 { 3-[(1R,2S,5R,6S)-5-Hydroxy-7-oxabicyclo[4.1.0]heptan-2-yl]-2-oxopropanoate } <-- L-Phenylalanine -- rn:R00689 <-- Phenylpyruvate --> rn:R01372

rn:R11068 { 3-[(1R,2S,5R,6S)-5-Hydroxy-7-oxabicyclo[4.1.0]heptan-2-yl]-2-oxopropanoate } <-- L-Phenylalanine -- rn:R00689 <-- Phenylpyruvate --> rn:R01375

rn:R11068 { 3-[(1R,2S,5R,6S)-5-Hydroxy-7-oxabicyclo[4.1.0]heptan-2-yl]-2-oxopropanoate } <-- L-Phenylalanine -- rn:R00689 <-- Phenylpyruvate --> rn:R01376

rn:R11068 { 3-[(1R,2S,5R,6S)-5-Hydroxy-7-oxabicyclo[4.1.0]heptan-2-yl]-2-oxopropanoate } <-- L-Phenylalanine -- rn:R00689 <-- Phenylpyruvate --> rn:R01377

rn:R11068 { 3-[(1R,2S,5R,6S)-5-Hydroxy-7-oxabicyclo[4.1.0]heptan-2-yl]-2-oxopropanoate } <-- L-Phenylalanine -- rn:R00689 <-- Phenylpyruvate --> rn:R01378

rn:R11068 { 3-[(1R,2S,5R,6S)-5-Hydroxy-7-oxabicyclo[4.1.0]heptan-2-yl]-2-oxopropanoate } <-- L-Phenylalanine -- rn:R00689 <-- Hydrogen peroxide --> rn:R02657

rn:R11068 { 3-[(1R,2S,5R,6S)-5-Hydroxy-7-oxabicyclo[4.1.0]heptan-2-yl]-2-oxopropanoate } <-- L-Phenylalanine -- rn:R00689 <-- Hydrogen peroxide --> rn:R03208

rn:R11068 { 3-[(1R,2S,5R,6S)-5-Hydroxy-7-oxabicyclo[4.1.0]heptan-2-yl]-2-oxopropanoate } <-- L-Phenylalanine -- rn:R00689 <-- Hydrogen peroxide --> rn:R03953

rn:R11068 { 3-[(1R,2S,5R,6S)-5-Hydroxy-7-oxabicyclo[4.1.0]heptan-2-yl]-2-oxopropanoate } <-- L-Phenylalanine -- rn:R00689 <-- Hydrogen peroxide --> rn:R08863

rn:R11068 { 3-[(1R,2S,5R,6S)-5-Hydroxy-7-oxabicyclo[4.1.0]heptan-2-yl]-2-oxopropanoate } <-- L-Phenylalanine -- rn:R00689 <-- Phenylpyruvate --> rn:R10431

rn:R11068 { 3-[(1R,2S,5R,6S)-5-Hydroxy-7-oxabicyclo[4.1.0]heptan-2-yl]-2-oxopropanoate } <-- L-Phenylalanine -- rn:R00689 <-- Phenylpyruvate --> rn:R10500

rn:R11068 { 3-[(1R,2S,5R,6S)-5-Hydroxy-7-oxabicyclo[4.1.0]heptan-2-yl]-2-oxopropanoate } <-- L-Phenylalanine -- rn:R00689 <-- Hydrogen peroxide --> rn:R11503

rn:R11068 { 3-[(1R,2S,5R,6S)-5-Hydroxy-7-oxabicyclo[4.1.0]heptan-2-yl]-2-oxopropanoate } <-- L-Phenylalanine -- rn:R00689 <-- Hydrogen peroxide --> rn:R11522

rn:R11068 { 3-[(1R,2S,5R,6S)-5-Hydroxy-7-oxabicyclo[4.1.0]heptan-2-yl]-2-oxopropanoate } <-- L-Phenylalanine -- rn:R00690 <-- 2-Phenylacetamide --> rn:R02540

rn:R05136 { Salicylaldehyde } <-- Pyruvate -- rn:R00692 <-- Phenylpyruvate --> rn:R00695

rn:R11068 { 3-[(1R,2S,5R,6S)-5-Hydroxy-7-oxabicyclo[4.1.0]heptan-2-yl]-2-oxopropanoate } <-- L-Phenylalanine -- rn:R00692 <-- Phenylpyruvate --> rn:R00695

rn:R05136 { Salicylaldehyde } <-- Pyruvate -- rn:R00692 <-- Phenylpyruvate --> rn:R01372

rn:R11068 { 3-[(1R,2S,5R,6S)-5-Hydroxy-7-oxabicyclo[4.1.0]heptan-2-yl]-2-oxopropanoate } <-- L-Phenylalanine -- rn:R00692 <-- Phenylpyruvate --> rn:R01372

rn:R05136 { Salicylaldehyde } <-- Pyruvate -- rn:R00692 <-- Phenylpyruvate --> rn:R01375

rn:R11068 { 3-[(1R,2S,5R,6S)-5-Hydroxy-7-oxabicyclo[4.1.0]heptan-2-yl]-2-oxopropanoate } <-- L-Phenylalanine -- rn:R00692 <-- Phenylpyruvate --> rn:R01375

rn:R05136 { Salicylaldehyde } <-- Pyruvate -- rn:R00692 <-- Phenylpyruvate --> rn:R01376

rn:R11068 { 3-[(1R,2S,5R,6S)-5-Hydroxy-7-oxabicyclo[4.1.0]heptan-2-yl]-2-oxopropanoate } <-- L-Phenylalanine -- rn:R00692 <-- Phenylpyruvate --> rn:R01376

rn:R05136 { Salicylaldehyde } <-- Pyruvate -- rn:R00692 <-- Phenylpyruvate --> rn:R01377

rn:R11068 { 3-[(1R,2S,5R,6S)-5-Hydroxy-7-oxabicyclo[4.1.0]heptan-2-yl]-2-oxopropanoate } <-- L-Phenylalanine -- rn:R00692 <-- Phenylpyruvate --> rn:R01377

rn:R05136 { Salicylaldehyde } <-- Pyruvate -- rn:R00692 <-- Phenylpyruvate --> rn:R01378

rn:R11068 { 3-[(1R,2S,5R,6S)-5-Hydroxy-7-oxabicyclo[4.1.0]heptan-2-yl]-2-oxopropanoate } <-- L-Phenylalanine -- rn:R00692 <-- Phenylpyruvate --> rn:R01378

rn:R05136 { Salicylaldehyde } <-- Pyruvate -- rn:R00692 <-- L-Alanine --> rn:R04187

rn:R11068 { 3-[(1R,2S,5R,6S)-5-Hydroxy-7-oxabicyclo[4.1.0]heptan-2-yl]-2-oxopropanoate } <-- L-Phenylalanine -- rn:R00692 <-- L-Alanine --> rn:R04187

rn:R05136 { Salicylaldehyde } <-- Pyruvate -- rn:R00692 <-- L-Alanine --> rn:R08872

rn:R11068 { 3-[(1R,2S,5R,6S)-5-Hydroxy-7-oxabicyclo[4.1.0]heptan-2-yl]-2-oxopropanoate } <-- L-Phenylalanine -- rn:R00692 <-- L-Alanine --> rn:R08872

rn:R05136 { Salicylaldehyde } <-- Pyruvate -- rn:R00692 <-- Phenylpyruvate --> rn:R10431

rn:R11068 { 3-[(1R,2S,5R,6S)-5-Hydroxy-7-oxabicyclo[4.1.0]heptan-2-yl]-2-oxopropanoate } <-- L-Phenylalanine -- rn:R00692 <-- Phenylpyruvate --> rn:R10431

rn:R05136 { Salicylaldehyde } <-- Pyruvate -- rn:R00692 <-- Phenylpyruvate --> rn:R10500

rn:R11068 { 3-[(1R,2S,5R,6S)-5-Hydroxy-7-oxabicyclo[4.1.0]heptan-2-yl]-2-oxopropanoate } <-- L-Phenylalanine -- rn:R00692 <-- Phenylpyruvate --> rn:R10500

rn:R11068 { 3-[(1R,2S,5R,6S)-5-Hydroxy-7-oxabicyclo[4.1.0]heptan-2-yl]-2-oxopropanoate } <-- L-Phenylalanine -- rn:R00694 <-- L-Glutamate --> rn:R00114

rn:R11068 { 3-[(1R,2S,5R,6S)-5-Hydroxy-7-oxabicyclo[4.1.0]heptan-2-yl]-2-oxopropanoate } <-- L-Phenylalanine -- rn:R00694 <-- L-Glutamate --> rn:R00248

rn:R11068 { 3-[(1R,2S,5R,6S)-5-Hydroxy-7-oxabicyclo[4.1.0]heptan-2-yl]-2-oxopropanoate } <-- L-Phenylalanine -- rn:R00694 <-- L-Glutamate --> rn:R00254

rn:R11068 { 3-[(1R,2S,5R,6S)-5-Hydroxy-7-oxabicyclo[4.1.0]heptan-2-yl]-2-oxopropanoate } <-- L-Phenylalanine -- rn:R00694 <-- Phenylpyruvate --> rn:R00695

rn:R11068 { 3-[(1R,2S,5R,6S)-5-Hydroxy-7-oxabicyclo[4.1.0]heptan-2-yl]-2-oxopropanoate } <-- L-Phenylalanine -- rn:R00694 <-- L-Glutamate --> rn:R00894

rn:R11068 { 3-[(1R,2S,5R,6S)-5-Hydroxy-7-oxabicyclo[4.1.0]heptan-2-yl]-2-oxopropanoate } <-- L-Phenylalanine -- rn:R00694 <-- Phenylpyruvate --> rn:R01372

rn:R11068 { 3-[(1R,2S,5R,6S)-5-Hydroxy-7-oxabicyclo[4.1.0]heptan-2-yl]-2-oxopropanoate } <-- L-Phenylalanine -- rn:R00694 <-- Phenylpyruvate --> rn:R01375

rn:R11068 { 3-[(1R,2S,5R,6S)-5-Hydroxy-7-oxabicyclo[4.1.0]heptan-2-yl]-2-oxopropanoate } <-- L-Phenylalanine -- rn:R00694 <-- Phenylpyruvate --> rn:R01376

rn:R11068 { 3-[(1R,2S,5R,6S)-5-Hydroxy-7-oxabicyclo[4.1.0]heptan-2-yl]-2-oxopropanoate } <-- L-Phenylalanine -- rn:R00694 <-- Phenylpyruvate --> rn:R01377

rn:R11068 { 3-[(1R,2S,5R,6S)-5-Hydroxy-7-oxabicyclo[4.1.0]heptan-2-yl]-2-oxopropanoate } <-- L-Phenylalanine -- rn:R00694 <-- Phenylpyruvate --> rn:R01378

rn:R11068 { 3-[(1R,2S,5R,6S)-5-Hydroxy-7-oxabicyclo[4.1.0]heptan-2-yl]-2-oxopropanoate } <-- L-Phenylalanine -- rn:R00694 <-- L-Glutamate --> rn:R02287

rn:R11068 { 3-[(1R,2S,5R,6S)-5-Hydroxy-7-oxabicyclo[4.1.0]heptan-2-yl]-2-oxopropanoate } <-- L-Phenylalanine -- rn:R00694 <-- L-Glutamate --> rn:R03189

rn:R11068 { 3-[(1R,2S,5R,6S)-5-Hydroxy-7-oxabicyclo[4.1.0]heptan-2-yl]-2-oxopropanoate } <-- L-Phenylalanine -- rn:R00694 <-- L-Glutamate --> rn:R03970

rn:R11068 { 3-[(1R,2S,5R,6S)-5-Hydroxy-7-oxabicyclo[4.1.0]heptan-2-yl]-2-oxopropanoate } <-- L-Phenylalanine -- rn:R00694 <-- L-Glutamate --> rn:R03971

rn:R11068 { 3-[(1R,2S,5R,6S)-5-Hydroxy-7-oxabicyclo[4.1.0]heptan-2-yl]-2-oxopropanoate } <-- L-Phenylalanine -- rn:R00694 <-- L-Glutamate --> rn:R04051

rn:R11068 { 3-[(1R,2S,5R,6S)-5-Hydroxy-7-oxabicyclo[4.1.0]heptan-2-yl]-2-oxopropanoate } <-- L-Phenylalanine -- rn:R00694 <-- L-Glutamate --> rn:R04776

rn:R11068 { 3-[(1R,2S,5R,6S)-5-Hydroxy-7-oxabicyclo[4.1.0]heptan-2-yl]-2-oxopropanoate } <-- L-Phenylalanine -- rn:R00694 <-- L-Glutamate --> rn:R07643

rn:R11068 { 3-[(1R,2S,5R,6S)-5-Hydroxy-7-oxabicyclo[4.1.0]heptan-2-yl]-2-oxopropanoate } <-- L-Phenylalanine -- rn:R00694 <-- Phenylpyruvate --> rn:R10431

rn:R11068 { 3-[(1R,2S,5R,6S)-5-Hydroxy-7-oxabicyclo[4.1.0]heptan-2-yl]-2-oxopropanoate } <-- L-Phenylalanine -- rn:R00694 <-- Phenylpyruvate --> rn:R10500

rn:R11068 { 3-[(1R,2S,5R,6S)-5-Hydroxy-7-oxabicyclo[4.1.0]heptan-2-yl]-2-oxopropanoate } <-- L-Phenylalanine -- rn:R00697 <-- trans-Cinnamate --> rn:R02253

rn:R11068 { 3-[(1R,2S,5R,6S)-5-Hydroxy-7-oxabicyclo[4.1.0]heptan-2-yl]-2-oxopropanoate } <-- L-Phenylalanine -- rn:R00697 <-- trans-Cinnamate --> rn:R02254

rn:R11068 { 3-[(1R,2S,5R,6S)-5-Hydroxy-7-oxabicyclo[4.1.0]heptan-2-yl]-2-oxopropanoate } <-- L-Phenylalanine -- rn:R00697 <-- trans-Cinnamate --> rn:R02255

rn:R11068 { 3-[(1R,2S,5R,6S)-5-Hydroxy-7-oxabicyclo[4.1.0]heptan-2-yl]-2-oxopropanoate } <-- L-Phenylalanine -- rn:R00697 <-- trans-Cinnamate --> rn:R02256

rn:R11068 { 3-[(1R,2S,5R,6S)-5-Hydroxy-7-oxabicyclo[4.1.0]heptan-2-yl]-2-oxopropanoate } <-- L-Phenylalanine -- rn:R00697 <-- trans-Cinnamate --> rn:R06745

rn:R11068 { 3-[(1R,2S,5R,6S)-5-Hydroxy-7-oxabicyclo[4.1.0]heptan-2-yl]-2-oxopropanoate } <-- L-Phenylalanine -- rn:R00697 <-- trans-Cinnamate --> rn:R06781

rn:R11068 { 3-[(1R,2S,5R,6S)-5-Hydroxy-7-oxabicyclo[4.1.0]heptan-2-yl]-2-oxopropanoate } <-- L-Phenylalanine -- rn:R00697 <-- trans-Cinnamate --> rn:R06783

rn:R11068 { 3-[(1R,2S,5R,6S)-5-Hydroxy-7-oxabicyclo[4.1.0]heptan-2-yl]-2-oxopropanoate } <-- L-Phenylalanine -- rn:R00697 <-- trans-Cinnamate --> rn:R08422

rn:R11068 { 3-[(1R,2S,5R,6S)-5-Hydroxy-7-oxabicyclo[4.1.0]heptan-2-yl]-2-oxopropanoate } <-- L-Phenylalanine -- rn:R00697 <-- trans-Cinnamate --> rn:R08423

rn:R11068 { 3-[(1R,2S,5R,6S)-5-Hydroxy-7-oxabicyclo[4.1.0]heptan-2-yl]-2-oxopropanoate } <-- L-Phenylalanine -- rn:R00697 <-- trans-Cinnamate --> rn:R11070

rn:R11068 { 3-[(1R,2S,5R,6S)-5-Hydroxy-7-oxabicyclo[4.1.0]heptan-2-yl]-2-oxopropanoate } <-- L-Phenylalanine -- rn:R00698 <-- 2-Phenylacetamide --> rn:R02540

rn:R11068 { 3-[(1R,2S,5R,6S)-5-Hydroxy-7-oxabicyclo[4.1.0]heptan-2-yl]-2-oxopropanoate } <-- L-Phenylalanine -- rn:R00699 <-- Phenethylamine --> rn:R02612

rn:R11068 { 3-[(1R,2S,5R,6S)-5-Hydroxy-7-oxabicyclo[4.1.0]heptan-2-yl]-2-oxopropanoate } <-- L-Phenylalanine -- rn:R00699 <-- Phenethylamine --> rn:R02613

rn:R02468 { (-)-Limonene } <-- [Reduced NADPH---hemoprotein reductase] -- rn:R00730 <-- N-Hydroxy-L-tyrosine --> rn:R04460

rn:R02469 { (-)-Limonene } <-- [Reduced NADPH---hemoprotein reductase] -- rn:R00730 <-- N-Hydroxy-L-tyrosine --> rn:R04460

rn:R02470 { (-)-Limonene } <-- [Reduced NADPH---hemoprotein reductase] -- rn:R00730 <-- N-Hydroxy-L-tyrosine --> rn:R04460

rn:R04366 { Linalool } <-- [Reduced NADPH---hemoprotein reductase] -- rn:R00730 <-- N-Hydroxy-L-tyrosine --> rn:R04460

rn:R06119 { d-Limonene } <-- [Reduced NADPH---hemoprotein reductase] -- rn:R00730 <-- N-Hydroxy-L-tyrosine --> rn:R04460

rn:R09451 { Hexadecanoic acid } <-- [Reduced NADPH---hemoprotein reductase] -- rn:R00730 <-- N-Hydroxy-L-tyrosine --> rn:R04460

rn:R09452 { (9Z)-Octadecenoic acid } <-- [Reduced NADPH---hemoprotein reductase] -- rn:R00730 <-- N-Hydroxy-L-tyrosine --> rn:R04460

rn:R09922 { (+)-Linalool } <-- [Reduced NADPH---hemoprotein reductase] -- rn:R00730 <-- N-Hydroxy-L-tyrosine --> rn:R04460

rn:R09923 { (-)-Linalool } <-- [Reduced NADPH---hemoprotein reductase] -- rn:R00730 <-- N-Hydroxy-L-tyrosine --> rn:R04460

rn:R09925 { Linalool } <-- [Reduced NADPH---hemoprotein reductase] -- rn:R00730 <-- N-Hydroxy-L-tyrosine --> rn:R04460

rn:R09934 { Humulene } <-- [Reduced NADPH---hemoprotein reductase] -- rn:R00730 <-- N-Hydroxy-L-tyrosine --> rn:R04460

rn:R10562 { (E,E)-Geranyllinalool } <-- [Reduced NADPH---hemoprotein reductase] -- rn:R00730 <-- N-Hydroxy-L-tyrosine --> rn:R04460

rn:R11055 { Myrcene } <-- [Reduced NADPH---hemoprotein reductase] -- rn:R00730 <-- N-Hydroxy-L-tyrosine --> rn:R04460

rn:R02468 { (-)-Limonene } <-- [Reduced NADPH---hemoprotein reductase] -- rn:R00730 <-- [Oxidized NADPH---hemoprotein reductase] --> rn:R08551

rn:R02469 { (-)-Limonene } <-- [Reduced NADPH---hemoprotein reductase] -- rn:R00730 <-- [Oxidized NADPH---hemoprotein reductase] --> rn:R08551

rn:R02470 { (-)-Limonene } <-- [Reduced NADPH---hemoprotein reductase] -- rn:R00730 <-- [Oxidized NADPH---hemoprotein reductase] --> rn:R08551

rn:R04366 { Linalool } <-- [Reduced NADPH---hemoprotein reductase] -- rn:R00730 <-- [Oxidized NADPH---hemoprotein reductase] --> rn:R08551

rn:R06119 { d-Limonene } <-- [Reduced NADPH---hemoprotein reductase] -- rn:R00730 <-- [Oxidized NADPH---hemoprotein reductase] --> rn:R08551

rn:R09451 { Hexadecanoic acid } <-- [Reduced NADPH---hemoprotein reductase] -- rn:R00730 <-- [Oxidized NADPH---hemoprotein reductase] --> rn:R08551

rn:R09452 { (9Z)-Octadecenoic acid } <-- [Reduced NADPH---hemoprotein reductase] -- rn:R00730 <-- [Oxidized NADPH---hemoprotein reductase] --> rn:R08551

rn:R09922 { (+)-Linalool } <-- [Reduced NADPH---hemoprotein reductase] -- rn:R00730 <-- [Oxidized NADPH---hemoprotein reductase] --> rn:R08551

rn:R09923 { (-)-Linalool } <-- [Reduced NADPH---hemoprotein reductase] -- rn:R00730 <-- [Oxidized NADPH---hemoprotein reductase] --> rn:R08551

rn:R09925 { Linalool } <-- [Reduced NADPH---hemoprotein reductase] -- rn:R00730 <-- [Oxidized NADPH---hemoprotein reductase] --> rn:R08551

rn:R09934 { Humulene } <-- [Reduced NADPH---hemoprotein reductase] -- rn:R00730 <-- [Oxidized NADPH---hemoprotein reductase] --> rn:R08551

rn:R10562 { (E,E)-Geranyllinalool } <-- [Reduced NADPH---hemoprotein reductase] -- rn:R00730 <-- [Oxidized NADPH---hemoprotein reductase] --> rn:R08551

rn:R11055 { Myrcene } <-- [Reduced NADPH---hemoprotein reductase] -- rn:R00730 <-- [Oxidized NADPH---hemoprotein reductase] --> rn:R08551

rn:R02781 { 2,4,6/3,5-Pentahydroxycyclohexanone } <-- L-Glutamine -- rn:R00986 <-- L-Glutamate --> rn:R00114

rn:R02781 { 2,4,6/3,5-Pentahydroxycyclohexanone } <-- L-Glutamine -- rn:R00986 <-- Pyruvate --> rn:R00210

rn:R02781 { 2,4,6/3,5-Pentahydroxycyclohexanone } <-- L-Glutamine -- rn:R00986 <-- L-Glutamate --> rn:R00248

rn:R02781 { 2,4,6/3,5-Pentahydroxycyclohexanone } <-- L-Glutamine -- rn:R00986 <-- L-Glutamate --> rn:R00254

rn:R02781 { 2,4,6/3,5-Pentahydroxycyclohexanone } <-- L-Glutamine -- rn:R00986 <-- Pyruvate --> rn:R00692

rn:R02781 { 2,4,6/3,5-Pentahydroxycyclohexanone } <-- L-Glutamine -- rn:R00986 <-- Pyruvate --> rn:R00750

rn:R02781 { 2,4,6/3,5-Pentahydroxycyclohexanone } <-- L-Glutamine -- rn:R00986 <-- Anthranilate --> rn:R00823

rn:R02781 { 2,4,6/3,5-Pentahydroxycyclohexanone } <-- L-Glutamine -- rn:R00986 <-- Anthranilate --> rn:R00825

rn:R02781 { 2,4,6/3,5-Pentahydroxycyclohexanone } <-- L-Glutamine -- rn:R00986 <-- L-Glutamate --> rn:R00894

rn:R02781 { 2,4,6/3,5-Pentahydroxycyclohexanone } <-- L-Glutamine -- rn:R00986 <-- Anthranilate --> rn:R00980

rn:R02781 { 2,4,6/3,5-Pentahydroxycyclohexanone } <-- L-Glutamine -- rn:R00986 <-- Anthranilate --> rn:R00982

rn:R02781 { 2,4,6/3,5-Pentahydroxycyclohexanone } <-- L-Glutamine -- rn:R00986 <-- Anthranilate --> rn:R00984

rn:R02781 { 2,4,6/3,5-Pentahydroxycyclohexanone } <-- L-Glutamine -- rn:R00986 <-- Anthranilate --> rn:R00989

rn:R02781 { 2,4,6/3,5-Pentahydroxycyclohexanone } <-- L-Glutamine -- rn:R00986 <-- Anthranilate --> rn:R00990

rn:R02781 { 2,4,6/3,5-Pentahydroxycyclohexanone } <-- L-Glutamine -- rn:R00986 <-- Anthranilate --> rn:R00991

rn:R02781 { 2,4,6/3,5-Pentahydroxycyclohexanone } <-- L-Glutamine -- rn:R00986 <-- Pyruvate --> rn:R01147

rn:R02781 { 2,4,6/3,5-Pentahydroxycyclohexanone } <-- L-Glutamine -- rn:R00986 <-- Pyruvate --> rn:R01302

rn:R02781 { 2,4,6/3,5-Pentahydroxycyclohexanone } <-- L-Glutamine -- rn:R00986 <-- Pyruvate --> rn:R01647

rn:R02781 { 2,4,6/3,5-Pentahydroxycyclohexanone } <-- L-Glutamine -- rn:R00986 <-- Pyruvate --> rn:R01712

rn:R02781 { 2,4,6/3,5-Pentahydroxycyclohexanone } <-- L-Glutamine -- rn:R00986 <-- Anthranilate --> rn:R01816

rn:R02781 { 2,4,6/3,5-Pentahydroxycyclohexanone } <-- L-Glutamine -- rn:R00986 <-- Pyruvate --> rn:R02050

rn:R02781 { 2,4,6/3,5-Pentahydroxycyclohexanone } <-- L-Glutamine -- rn:R00986 <-- Pyruvate --> rn:R02271

rn:R02781 { 2,4,6/3,5-Pentahydroxycyclohexanone } <-- L-Glutamine -- rn:R00986 <-- L-Glutamate --> rn:R02287

rn:R02781 { 2,4,6/3,5-Pentahydroxycyclohexanone } <-- L-Glutamine -- rn:R00986 <-- Anthranilate --> rn:R02453

rn:R02781 { 2,4,6/3,5-Pentahydroxycyclohexanone } <-- L-Glutamine -- rn:R00986 <-- L-Glutamate --> rn:R03189

rn:R02781 { 2,4,6/3,5-Pentahydroxycyclohexanone } <-- L-Glutamine -- rn:R00986 <-- L-Glutamate --> rn:R03970

rn:R02781 { 2,4,6/3,5-Pentahydroxycyclohexanone } <-- L-Glutamine -- rn:R00986 <-- L-Glutamate --> rn:R03971

rn:R02781 { 2,4,6/3,5-Pentahydroxycyclohexanone } <-- L-Glutamine -- rn:R00986 <-- L-Glutamate --> rn:R04051

rn:R02781 { 2,4,6/3,5-Pentahydroxycyclohexanone } <-- L-Glutamine -- rn:R00986 <-- Pyruvate --> rn:R04152

rn:R02781 { 2,4,6/3,5-Pentahydroxycyclohexanone } <-- L-Glutamine -- rn:R00986 <-- L-Glutamate --> rn:R04776

rn:R02781 { 2,4,6/3,5-Pentahydroxycyclohexanone } <-- L-Glutamine -- rn:R00986 <-- L-Glutamate --> rn:R07643

rn:R02781 { 2,4,6/3,5-Pentahydroxycyclohexanone } <-- L-Glutamine -- rn:R00986 <-- Anthranilate --> rn:R08473

rn:R02781 { 2,4,6/3,5-Pentahydroxycyclohexanone } <-- L-Glutamine -- rn:R00986 <-- Anthranilate --> rn:R08477

rn:R02781 { 2,4,6/3,5-Pentahydroxycyclohexanone } <-- L-Glutamine -- rn:R00986 <-- Pyruvate --> rn:R09088

rn:R02781 { 2,4,6/3,5-Pentahydroxycyclohexanone } <-- L-Glutamine -- rn:R00986 <-- Anthranilate --> rn:R09517

rn:R02781 { 2,4,6/3,5-Pentahydroxycyclohexanone } <-- L-Glutamine -- rn:R00986 <-- Pyruvate --> rn:R10180

rn:R02781 { 2,4,6/3,5-Pentahydroxycyclohexanone } <-- L-Glutamine -- rn:R00986 <-- Anthranilate --> rn:R10451

rn:R02781 { 2,4,6/3,5-Pentahydroxycyclohexanone } <-- L-Glutamine -- rn:R00986 <-- Anthranilate --> rn:R10495

rn:R02781 { 2,4,6/3,5-Pentahydroxycyclohexanone } <-- L-Glutamine -- rn:R00986 <-- Anthranilate --> rn:R12035

rn:R05136 { Salicylaldehyde } <-- Pyruvate -- rn:R01147 <-- Pyridoxal phosphate --> rn:R00173

rn:R05136 { Salicylaldehyde } <-- Pyruvate -- rn:R01147 <-- D-Alanine --> rn:R02718

rn:R05136 { Salicylaldehyde } <-- Pyruvate -- rn:R01147 <-- D-Alanine --> rn:R04369

rn:R05537 { 3-(2-Hydroxyphenyl)propanoate } <-- FAD -- rn:R01279 <-- FADH2 --> rn:R03978

rn:R05537 { 3-(2-Hydroxyphenyl)propanoate } <-- FAD -- rn:R01279 <-- FADH2 --> rn:R05488

rn:R05537 { 3-(2-Hydroxyphenyl)propanoate } <-- FAD -- rn:R01279 <-- FADH2 --> rn:R09517

rn:R05537 { 3-(2-Hydroxyphenyl)propanoate } <-- FAD -- rn:R01279 <-- FADH2 --> rn:R11653

rn:R05537 { 3-(2-Hydroxyphenyl)propanoate } <-- FAD -- rn:R01279 <-- FADH2 --> rn:R12021

rn:R05537 { 3-(2-Hydroxyphenyl)propanoate } <-- FAD -- rn:R01279 <-- FADH2 --> rn:R12023

rn:R05537 { 3-(2-Hydroxyphenyl)propanoate } <-- FAD -- rn:R01279 <-- FADH2 --> rn:R12027

rn:R05537 { 3-(2-Hydroxyphenyl)propanoate } <-- FAD -- rn:R01279 <-- FADH2 --> rn:R12030

rn:R02468 { (-)-Limonene } <-- [Reduced NADPH---hemoprotein reductase] -- rn:R01295 <-- 4-Hydroxybenzoate --> rn:R01238

rn:R02469 { (-)-Limonene } <-- [Reduced NADPH---hemoprotein reductase] -- rn:R01295 <-- 4-Hydroxybenzoate --> rn:R01238

rn:R02470 { (-)-Limonene } <-- [Reduced NADPH---hemoprotein reductase] -- rn:R01295 <-- 4-Hydroxybenzoate --> rn:R01238

rn:R04366 { Linalool } <-- [Reduced NADPH---hemoprotein reductase] -- rn:R01295 <-- 4-Hydroxybenzoate --> rn:R01238

rn:R06119 { d-Limonene } <-- [Reduced NADPH---hemoprotein reductase] -- rn:R01295 <-- 4-Hydroxybenzoate --> rn:R01238

rn:R09451 { Hexadecanoic acid } <-- [Reduced NADPH---hemoprotein reductase] -- rn:R01295 <-- 4-Hydroxybenzoate --> rn:R01238

rn:R09452 { (9Z)-Octadecenoic acid } <-- [Reduced NADPH---hemoprotein reductase] -- rn:R01295 <-- 4-Hydroxybenzoate --> rn:R01238

rn:R09922 { (+)-Linalool } <-- [Reduced NADPH---hemoprotein reductase] -- rn:R01295 <-- 4-Hydroxybenzoate --> rn:R01238

rn:R09923 { (-)-Linalool } <-- [Reduced NADPH---hemoprotein reductase] -- rn:R01295 <-- 4-Hydroxybenzoate --> rn:R01238

rn:R09925 { Linalool } <-- [Reduced NADPH---hemoprotein reductase] -- rn:R01295 <-- 4-Hydroxybenzoate --> rn:R01238

rn:R09934 { Humulene } <-- [Reduced NADPH---hemoprotein reductase] -- rn:R01295 <-- 4-Hydroxybenzoate --> rn:R01238

rn:R10562 { (E,E)-Geranyllinalool } <-- [Reduced NADPH---hemoprotein reductase] -- rn:R01295 <-- 4-Hydroxybenzoate --> rn:R01238

rn:R11055 { Myrcene } <-- [Reduced NADPH---hemoprotein reductase] -- rn:R01295 <-- 4-Hydroxybenzoate --> rn:R01238

rn:R02468 { (-)-Limonene } <-- [Reduced NADPH---hemoprotein reductase] -- rn:R01295 <-- 4-Hydroxybenzoate --> rn:R01296

rn:R02469 { (-)-Limonene } <-- [Reduced NADPH---hemoprotein reductase] -- rn:R01295 <-- 4-Hydroxybenzoate --> rn:R01296

rn:R02470 { (-)-Limonene } <-- [Reduced NADPH---hemoprotein reductase] -- rn:R01295 <-- 4-Hydroxybenzoate --> rn:R01296

rn:R04366 { Linalool } <-- [Reduced NADPH---hemoprotein reductase] -- rn:R01295 <-- 4-Hydroxybenzoate --> rn:R01296

rn:R06119 { d-Limonene } <-- [Reduced NADPH---hemoprotein reductase] -- rn:R01295 <-- 4-Hydroxybenzoate --> rn:R01296

rn:R09451 { Hexadecanoic acid } <-- [Reduced NADPH---hemoprotein reductase] -- rn:R01295 <-- 4-Hydroxybenzoate --> rn:R01296

rn:R09452 { (9Z)-Octadecenoic acid } <-- [Reduced NADPH---hemoprotein reductase] -- rn:R01295 <-- 4-Hydroxybenzoate --> rn:R01296

rn:R09922 { (+)-Linalool } <-- [Reduced NADPH---hemoprotein reductase] -- rn:R01295 <-- 4-Hydroxybenzoate --> rn:R01296

rn:R09923 { (-)-Linalool } <-- [Reduced NADPH---hemoprotein reductase] -- rn:R01295 <-- 4-Hydroxybenzoate --> rn:R01296

rn:R09925 { Linalool } <-- [Reduced NADPH---hemoprotein reductase] -- rn:R01295 <-- 4-Hydroxybenzoate --> rn:R01296

rn:R09934 { Humulene } <-- [Reduced NADPH---hemoprotein reductase] -- rn:R01295 <-- 4-Hydroxybenzoate --> rn:R01296

rn:R10562 { (E,E)-Geranyllinalool } <-- [Reduced NADPH---hemoprotein reductase] -- rn:R01295 <-- 4-Hydroxybenzoate --> rn:R01296

rn:R11055 { Myrcene } <-- [Reduced NADPH---hemoprotein reductase] -- rn:R01295 <-- 4-Hydroxybenzoate --> rn:R01296

rn:R02468 { (-)-Limonene } <-- [Reduced NADPH---hemoprotein reductase] -- rn:R01295 <-- 4-Hydroxybenzoate --> rn:R01297

rn:R02469 { (-)-Limonene } <-- [Reduced NADPH---hemoprotein reductase] -- rn:R01295 <-- 4-Hydroxybenzoate --> rn:R01297

rn:R02470 { (-)-Limonene } <-- [Reduced NADPH---hemoprotein reductase] -- rn:R01295 <-- 4-Hydroxybenzoate --> rn:R01297

rn:R04366 { Linalool } <-- [Reduced NADPH---hemoprotein reductase] -- rn:R01295 <-- 4-Hydroxybenzoate --> rn:R01297

rn:R06119 { d-Limonene } <-- [Reduced NADPH---hemoprotein reductase] -- rn:R01295 <-- 4-Hydroxybenzoate --> rn:R01297

rn:R09451 { Hexadecanoic acid } <-- [Reduced NADPH---hemoprotein reductase] -- rn:R01295 <-- 4-Hydroxybenzoate --> rn:R01297

rn:R09452 { (9Z)-Octadecenoic acid } <-- [Reduced NADPH---hemoprotein reductase] -- rn:R01295 <-- 4-Hydroxybenzoate --> rn:R01297

rn:R09922 { (+)-Linalool } <-- [Reduced NADPH---hemoprotein reductase] -- rn:R01295 <-- 4-Hydroxybenzoate --> rn:R01297

rn:R09923 { (-)-Linalool } <-- [Reduced NADPH---hemoprotein reductase] -- rn:R01295 <-- 4-Hydroxybenzoate --> rn:R01297

rn:R09925 { Linalool } <-- [Reduced NADPH---hemoprotein reductase] -- rn:R01295 <-- 4-Hydroxybenzoate --> rn:R01297

rn:R09934 { Humulene } <-- [Reduced NADPH---hemoprotein reductase] -- rn:R01295 <-- 4-Hydroxybenzoate --> rn:R01297

rn:R10562 { (E,E)-Geranyllinalool } <-- [Reduced NADPH---hemoprotein reductase] -- rn:R01295 <-- 4-Hydroxybenzoate --> rn:R01297

rn:R11055 { Myrcene } <-- [Reduced NADPH---hemoprotein reductase] -- rn:R01295 <-- 4-Hydroxybenzoate --> rn:R01297

rn:R02468 { (-)-Limonene } <-- [Reduced NADPH---hemoprotein reductase] -- rn:R01295 <-- 4-Hydroxybenzoate --> rn:R01298

rn:R02469 { (-)-Limonene } <-- [Reduced NADPH---hemoprotein reductase] -- rn:R01295 <-- 4-Hydroxybenzoate --> rn:R01298

rn:R02470 { (-)-Limonene } <-- [Reduced NADPH---hemoprotein reductase] -- rn:R01295 <-- 4-Hydroxybenzoate --> rn:R01298

rn:R04366 { Linalool } <-- [Reduced NADPH---hemoprotein reductase] -- rn:R01295 <-- 4-Hydroxybenzoate --> rn:R01298

rn:R06119 { d-Limonene } <-- [Reduced NADPH---hemoprotein reductase] -- rn:R01295 <-- 4-Hydroxybenzoate --> rn:R01298

rn:R09451 { Hexadecanoic acid } <-- [Reduced NADPH---hemoprotein reductase] -- rn:R01295 <-- 4-Hydroxybenzoate --> rn:R01298

rn:R09452 { (9Z)-Octadecenoic acid } <-- [Reduced NADPH---hemoprotein reductase] -- rn:R01295 <-- 4-Hydroxybenzoate --> rn:R01298

rn:R09922 { (+)-Linalool } <-- [Reduced NADPH---hemoprotein reductase] -- rn:R01295 <-- 4-Hydroxybenzoate --> rn:R01298

rn:R09923 { (-)-Linalool } <-- [Reduced NADPH---hemoprotein reductase] -- rn:R01295 <-- 4-Hydroxybenzoate --> rn:R01298

rn:R09925 { Linalool } <-- [Reduced NADPH---hemoprotein reductase] -- rn:R01295 <-- 4-Hydroxybenzoate --> rn:R01298

rn:R09934 { Humulene } <-- [Reduced NADPH---hemoprotein reductase] -- rn:R01295 <-- 4-Hydroxybenzoate --> rn:R01298

rn:R10562 { (E,E)-Geranyllinalool } <-- [Reduced NADPH---hemoprotein reductase] -- rn:R01295 <-- 4-Hydroxybenzoate --> rn:R01298

rn:R11055 { Myrcene } <-- [Reduced NADPH---hemoprotein reductase] -- rn:R01295 <-- 4-Hydroxybenzoate --> rn:R01298

rn:R02468 { (-)-Limonene } <-- [Reduced NADPH---hemoprotein reductase] -- rn:R01295 <-- 4-Hydroxybenzoate --> rn:R01299

rn:R02469 { (-)-Limonene } <-- [Reduced NADPH---hemoprotein reductase] -- rn:R01295 <-- 4-Hydroxybenzoate --> rn:R01299

rn:R02470 { (-)-Limonene } <-- [Reduced NADPH---hemoprotein reductase] -- rn:R01295 <-- 4-Hydroxybenzoate --> rn:R01299

rn:R04366 { Linalool } <-- [Reduced NADPH---hemoprotein reductase] -- rn:R01295 <-- 4-Hydroxybenzoate --> rn:R01299

rn:R06119 { d-Limonene } <-- [Reduced NADPH---hemoprotein reductase] -- rn:R01295 <-- 4-Hydroxybenzoate --> rn:R01299

rn:R09451 { Hexadecanoic acid } <-- [Reduced NADPH---hemoprotein reductase] -- rn:R01295 <-- 4-Hydroxybenzoate --> rn:R01299

rn:R09452 { (9Z)-Octadecenoic acid } <-- [Reduced NADPH---hemoprotein reductase] -- rn:R01295 <-- 4-Hydroxybenzoate --> rn:R01299

rn:R09922 { (+)-Linalool } <-- [Reduced NADPH---hemoprotein reductase] -- rn:R01295 <-- 4-Hydroxybenzoate --> rn:R01299

rn:R09923 { (-)-Linalool } <-- [Reduced NADPH---hemoprotein reductase] -- rn:R01295 <-- 4-Hydroxybenzoate --> rn:R01299

rn:R09925 { Linalool } <-- [Reduced NADPH---hemoprotein reductase] -- rn:R01295 <-- 4-Hydroxybenzoate --> rn:R01299

rn:R09934 { Humulene } <-- [Reduced NADPH---hemoprotein reductase] -- rn:R01295 <-- 4-Hydroxybenzoate --> rn:R01299

rn:R10562 { (E,E)-Geranyllinalool } <-- [Reduced NADPH---hemoprotein reductase] -- rn:R01295 <-- 4-Hydroxybenzoate --> rn:R01299

rn:R11055 { Myrcene } <-- [Reduced NADPH---hemoprotein reductase] -- rn:R01295 <-- 4-Hydroxybenzoate --> rn:R01299

rn:R02468 { (-)-Limonene } <-- [Reduced NADPH---hemoprotein reductase] -- rn:R01295 <-- 4-Hydroxybenzoate --> rn:R01300

rn:R02469 { (-)-Limonene } <-- [Reduced NADPH---hemoprotein reductase] -- rn:R01295 <-- 4-Hydroxybenzoate --> rn:R01300

rn:R02470 { (-)-Limonene } <-- [Reduced NADPH---hemoprotein reductase] -- rn:R01295 <-- 4-Hydroxybenzoate --> rn:R01300

rn:R04366 { Linalool } <-- [Reduced NADPH---hemoprotein reductase] -- rn:R01295 <-- 4-Hydroxybenzoate --> rn:R01300

rn:R06119 { d-Limonene } <-- [Reduced NADPH---hemoprotein reductase] -- rn:R01295 <-- 4-Hydroxybenzoate --> rn:R01300

rn:R09451 { Hexadecanoic acid } <-- [Reduced NADPH---hemoprotein reductase] -- rn:R01295 <-- 4-Hydroxybenzoate --> rn:R01300

rn:R09452 { (9Z)-Octadecenoic acid } <-- [Reduced NADPH---hemoprotein reductase] -- rn:R01295 <-- 4-Hydroxybenzoate --> rn:R01300

rn:R09922 { (+)-Linalool } <-- [Reduced NADPH---hemoprotein reductase] -- rn:R01295 <-- 4-Hydroxybenzoate --> rn:R01300

rn:R09923 { (-)-Linalool } <-- [Reduced NADPH---hemoprotein reductase] -- rn:R01295 <-- 4-Hydroxybenzoate --> rn:R01300

rn:R09925 { Linalool } <-- [Reduced NADPH---hemoprotein reductase] -- rn:R01295 <-- 4-Hydroxybenzoate --> rn:R01300

rn:R09934 { Humulene } <-- [Reduced NADPH---hemoprotein reductase] -- rn:R01295 <-- 4-Hydroxybenzoate --> rn:R01300

rn:R10562 { (E,E)-Geranyllinalool } <-- [Reduced NADPH---hemoprotein reductase] -- rn:R01295 <-- 4-Hydroxybenzoate --> rn:R01300

rn:R11055 { Myrcene } <-- [Reduced NADPH---hemoprotein reductase] -- rn:R01295 <-- 4-Hydroxybenzoate --> rn:R01300

rn:R02468 { (-)-Limonene } <-- [Reduced NADPH---hemoprotein reductase] -- rn:R01295 <-- 4-Hydroxybenzoate --> rn:R01302

rn:R02469 { (-)-Limonene } <-- [Reduced NADPH---hemoprotein reductase] -- rn:R01295 <-- 4-Hydroxybenzoate --> rn:R01302

rn:R02470 { (-)-Limonene } <-- [Reduced NADPH---hemoprotein reductase] -- rn:R01295 <-- 4-Hydroxybenzoate --> rn:R01302

rn:R04366 { Linalool } <-- [Reduced NADPH---hemoprotein reductase] -- rn:R01295 <-- 4-Hydroxybenzoate --> rn:R01302

rn:R06119 { d-Limonene } <-- [Reduced NADPH---hemoprotein reductase] -- rn:R01295 <-- 4-Hydroxybenzoate --> rn:R01302

rn:R09451 { Hexadecanoic acid } <-- [Reduced NADPH---hemoprotein reductase] -- rn:R01295 <-- 4-Hydroxybenzoate --> rn:R01302

rn:R09452 { (9Z)-Octadecenoic acid } <-- [Reduced NADPH---hemoprotein reductase] -- rn:R01295 <-- 4-Hydroxybenzoate --> rn:R01302

rn:R09922 { (+)-Linalool } <-- [Reduced NADPH---hemoprotein reductase] -- rn:R01295 <-- 4-Hydroxybenzoate --> rn:R01302

rn:R09923 { (-)-Linalool } <-- [Reduced NADPH---hemoprotein reductase] -- rn:R01295 <-- 4-Hydroxybenzoate --> rn:R01302

rn:R09925 { Linalool } <-- [Reduced NADPH---hemoprotein reductase] -- rn:R01295 <-- 4-Hydroxybenzoate --> rn:R01302

rn:R09934 { Humulene } <-- [Reduced NADPH---hemoprotein reductase] -- rn:R01295 <-- 4-Hydroxybenzoate --> rn:R01302

rn:R10562 { (E,E)-Geranyllinalool } <-- [Reduced NADPH---hemoprotein reductase] -- rn:R01295 <-- 4-Hydroxybenzoate --> rn:R01302

rn:R11055 { Myrcene } <-- [Reduced NADPH---hemoprotein reductase] -- rn:R01295 <-- 4-Hydroxybenzoate --> rn:R01302

rn:R02468 { (-)-Limonene } <-- [Reduced NADPH---hemoprotein reductase] -- rn:R01295 <-- 4-Hydroxybenzoate --> rn:R01303

rn:R02469 { (-)-Limonene } <-- [Reduced NADPH---hemoprotein reductase] -- rn:R01295 <-- 4-Hydroxybenzoate --> rn:R01303

rn:R02470 { (-)-Limonene } <-- [Reduced NADPH---hemoprotein reductase] -- rn:R01295 <-- 4-Hydroxybenzoate --> rn:R01303

rn:R04366 { Linalool } <-- [Reduced NADPH---hemoprotein reductase] -- rn:R01295 <-- 4-Hydroxybenzoate --> rn:R01303

rn:R06119 { d-Limonene } <-- [Reduced NADPH---hemoprotein reductase] -- rn:R01295 <-- 4-Hydroxybenzoate --> rn:R01303

rn:R09451 { Hexadecanoic acid } <-- [Reduced NADPH---hemoprotein reductase] -- rn:R01295 <-- 4-Hydroxybenzoate --> rn:R01303

rn:R09452 { (9Z)-Octadecenoic acid } <-- [Reduced NADPH---hemoprotein reductase] -- rn:R01295 <-- 4-Hydroxybenzoate --> rn:R01303

rn:R09922 { (+)-Linalool } <-- [Reduced NADPH---hemoprotein reductase] -- rn:R01295 <-- 4-Hydroxybenzoate --> rn:R01303

rn:R09923 { (-)-Linalool } <-- [Reduced NADPH---hemoprotein reductase] -- rn:R01295 <-- 4-Hydroxybenzoate --> rn:R01303

rn:R09925 { Linalool } <-- [Reduced NADPH---hemoprotein reductase] -- rn:R01295 <-- 4-Hydroxybenzoate --> rn:R01303

rn:R09934 { Humulene } <-- [Reduced NADPH---hemoprotein reductase] -- rn:R01295 <-- 4-Hydroxybenzoate --> rn:R01303

rn:R10562 { (E,E)-Geranyllinalool } <-- [Reduced NADPH---hemoprotein reductase] -- rn:R01295 <-- 4-Hydroxybenzoate --> rn:R01303

rn:R11055 { Myrcene } <-- [Reduced NADPH---hemoprotein reductase] -- rn:R01295 <-- 4-Hydroxybenzoate --> rn:R01303

rn:R02468 { (-)-Limonene } <-- [Reduced NADPH---hemoprotein reductase] -- rn:R01295 <-- 4-Hydroxybenzoate --> rn:R01304

rn:R02469 { (-)-Limonene } <-- [Reduced NADPH---hemoprotein reductase] -- rn:R01295 <-- 4-Hydroxybenzoate --> rn:R01304

rn:R02470 { (-)-Limonene } <-- [Reduced NADPH---hemoprotein reductase] -- rn:R01295 <-- 4-Hydroxybenzoate --> rn:R01304

rn:R04366 { Linalool } <-- [Reduced NADPH---hemoprotein reductase] -- rn:R01295 <-- 4-Hydroxybenzoate --> rn:R01304

rn:R06119 { d-Limonene } <-- [Reduced NADPH---hemoprotein reductase] -- rn:R01295 <-- 4-Hydroxybenzoate --> rn:R01304

rn:R09451 { Hexadecanoic acid } <-- [Reduced NADPH---hemoprotein reductase] -- rn:R01295 <-- 4-Hydroxybenzoate --> rn:R01304

rn:R09452 { (9Z)-Octadecenoic acid } <-- [Reduced NADPH---hemoprotein reductase] -- rn:R01295 <-- 4-Hydroxybenzoate --> rn:R01304

rn:R09922 { (+)-Linalool } <-- [Reduced NADPH---hemoprotein reductase] -- rn:R01295 <-- 4-Hydroxybenzoate --> rn:R01304

rn:R09923 { (-)-Linalool } <-- [Reduced NADPH---hemoprotein reductase] -- rn:R01295 <-- 4-Hydroxybenzoate --> rn:R01304

rn:R09925 { Linalool } <-- [Reduced NADPH---hemoprotein reductase] -- rn:R01295 <-- 4-Hydroxybenzoate --> rn:R01304

rn:R09934 { Humulene } <-- [Reduced NADPH---hemoprotein reductase] -- rn:R01295 <-- 4-Hydroxybenzoate --> rn:R01304

rn:R10562 { (E,E)-Geranyllinalool } <-- [Reduced NADPH---hemoprotein reductase] -- rn:R01295 <-- 4-Hydroxybenzoate --> rn:R01304

rn:R11055 { Myrcene } <-- [Reduced NADPH---hemoprotein reductase] -- rn:R01295 <-- 4-Hydroxybenzoate --> rn:R01304

rn:R02468 { (-)-Limonene } <-- [Reduced NADPH---hemoprotein reductase] -- rn:R01295 <-- 4-Hydroxybenzoate --> rn:R01308

rn:R02469 { (-)-Limonene } <-- [Reduced NADPH---hemoprotein reductase] -- rn:R01295 <-- 4-Hydroxybenzoate --> rn:R01308

rn:R02470 { (-)-Limonene } <-- [Reduced NADPH---hemoprotein reductase] -- rn:R01295 <-- 4-Hydroxybenzoate --> rn:R01308

rn:R04366 { Linalool } <-- [Reduced NADPH---hemoprotein reductase] -- rn:R01295 <-- 4-Hydroxybenzoate --> rn:R01308

rn:R06119 { d-Limonene } <-- [Reduced NADPH---hemoprotein reductase] -- rn:R01295 <-- 4-Hydroxybenzoate --> rn:R01308

rn:R09451 { Hexadecanoic acid } <-- [Reduced NADPH---hemoprotein reductase] -- rn:R01295 <-- 4-Hydroxybenzoate --> rn:R01308

rn:R09452 { (9Z)-Octadecenoic acid } <-- [Reduced NADPH---hemoprotein reductase] -- rn:R01295 <-- 4-Hydroxybenzoate --> rn:R01308

rn:R09922 { (+)-Linalool } <-- [Reduced NADPH---hemoprotein reductase] -- rn:R01295 <-- 4-Hydroxybenzoate --> rn:R01308

rn:R09923 { (-)-Linalool } <-- [Reduced NADPH---hemoprotein reductase] -- rn:R01295 <-- 4-Hydroxybenzoate --> rn:R01308

rn:R09925 { Linalool } <-- [Reduced NADPH---hemoprotein reductase] -- rn:R01295 <-- 4-Hydroxybenzoate --> rn:R01308

rn:R09934 { Humulene } <-- [Reduced NADPH---hemoprotein reductase] -- rn:R01295 <-- 4-Hydroxybenzoate --> rn:R01308

rn:R10562 { (E,E)-Geranyllinalool } <-- [Reduced NADPH---hemoprotein reductase] -- rn:R01295 <-- 4-Hydroxybenzoate --> rn:R01308

rn:R11055 { Myrcene } <-- [Reduced NADPH---hemoprotein reductase] -- rn:R01295 <-- 4-Hydroxybenzoate --> rn:R01308

rn:R02468 { (-)-Limonene } <-- [Reduced NADPH---hemoprotein reductase] -- rn:R01295 <-- 4-Hydroxybenzoate --> rn:R05000

rn:R02469 { (-)-Limonene } <-- [Reduced NADPH---hemoprotein reductase] -- rn:R01295 <-- 4-Hydroxybenzoate --> rn:R05000

rn:R02470 { (-)-Limonene } <-- [Reduced NADPH---hemoprotein reductase] -- rn:R01295 <-- 4-Hydroxybenzoate --> rn:R05000

rn:R04366 { Linalool } <-- [Reduced NADPH---hemoprotein reductase] -- rn:R01295 <-- 4-Hydroxybenzoate --> rn:R05000

rn:R06119 { d-Limonene } <-- [Reduced NADPH---hemoprotein reductase] -- rn:R01295 <-- 4-Hydroxybenzoate --> rn:R05000

rn:R09451 { Hexadecanoic acid } <-- [Reduced NADPH---hemoprotein reductase] -- rn:R01295 <-- 4-Hydroxybenzoate --> rn:R05000

rn:R09452 { (9Z)-Octadecenoic acid } <-- [Reduced NADPH---hemoprotein reductase] -- rn:R01295 <-- 4-Hydroxybenzoate --> rn:R05000

rn:R09922 { (+)-Linalool } <-- [Reduced NADPH---hemoprotein reductase] -- rn:R01295 <-- 4-Hydroxybenzoate --> rn:R05000

rn:R09923 { (-)-Linalool } <-- [Reduced NADPH---hemoprotein reductase] -- rn:R01295 <-- 4-Hydroxybenzoate --> rn:R05000

rn:R09925 { Linalool } <-- [Reduced NADPH---hemoprotein reductase] -- rn:R01295 <-- 4-Hydroxybenzoate --> rn:R05000

rn:R09934 { Humulene } <-- [Reduced NADPH---hemoprotein reductase] -- rn:R01295 <-- 4-Hydroxybenzoate --> rn:R05000

rn:R10562 { (E,E)-Geranyllinalool } <-- [Reduced NADPH---hemoprotein reductase] -- rn:R01295 <-- 4-Hydroxybenzoate --> rn:R05000

rn:R11055 { Myrcene } <-- [Reduced NADPH---hemoprotein reductase] -- rn:R01295 <-- 4-Hydroxybenzoate --> rn:R05000

rn:R02468 { (-)-Limonene } <-- [Reduced NADPH---hemoprotein reductase] -- rn:R01295 <-- 4-Hydroxybenzoate --> rn:R05615

rn:R02469 { (-)-Limonene } <-- [Reduced NADPH---hemoprotein reductase] -- rn:R01295 <-- 4-Hydroxybenzoate --> rn:R05615

rn:R02470 { (-)-Limonene } <-- [Reduced NADPH---hemoprotein reductase] -- rn:R01295 <-- 4-Hydroxybenzoate --> rn:R05615

rn:R04366 { Linalool } <-- [Reduced NADPH---hemoprotein reductase] -- rn:R01295 <-- 4-Hydroxybenzoate --> rn:R05615

rn:R06119 { d-Limonene } <-- [Reduced NADPH---hemoprotein reductase] -- rn:R01295 <-- 4-Hydroxybenzoate --> rn:R05615

rn:R09451 { Hexadecanoic acid } <-- [Reduced NADPH---hemoprotein reductase] -- rn:R01295 <-- 4-Hydroxybenzoate --> rn:R05615

rn:R09452 { (9Z)-Octadecenoic acid } <-- [Reduced NADPH---hemoprotein reductase] -- rn:R01295 <-- 4-Hydroxybenzoate --> rn:R05615

rn:R09922 { (+)-Linalool } <-- [Reduced NADPH---hemoprotein reductase] -- rn:R01295 <-- 4-Hydroxybenzoate --> rn:R05615

rn:R09923 { (-)-Linalool } <-- [Reduced NADPH---hemoprotein reductase] -- rn:R01295 <-- 4-Hydroxybenzoate --> rn:R05615

rn:R09925 { Linalool } <-- [Reduced NADPH---hemoprotein reductase] -- rn:R01295 <-- 4-Hydroxybenzoate --> rn:R05615

rn:R09934 { Humulene } <-- [Reduced NADPH---hemoprotein reductase] -- rn:R01295 <-- 4-Hydroxybenzoate --> rn:R05615

rn:R10562 { (E,E)-Geranyllinalool } <-- [Reduced NADPH---hemoprotein reductase] -- rn:R01295 <-- 4-Hydroxybenzoate --> rn:R05615

rn:R11055 { Myrcene } <-- [Reduced NADPH---hemoprotein reductase] -- rn:R01295 <-- 4-Hydroxybenzoate --> rn:R05615

rn:R02468 { (-)-Limonene } <-- [Reduced NADPH---hemoprotein reductase] -- rn:R01295 <-- 4-Hydroxybenzoate --> rn:R05616

rn:R02469 { (-)-Limonene } <-- [Reduced NADPH---hemoprotein reductase] -- rn:R01295 <-- 4-Hydroxybenzoate --> rn:R05616

rn:R02470 { (-)-Limonene } <-- [Reduced NADPH---hemoprotein reductase] -- rn:R01295 <-- 4-Hydroxybenzoate --> rn:R05616

rn:R04366 { Linalool } <-- [Reduced NADPH---hemoprotein reductase] -- rn:R01295 <-- 4-Hydroxybenzoate --> rn:R05616

rn:R06119 { d-Limonene } <-- [Reduced NADPH---hemoprotein reductase] -- rn:R01295 <-- 4-Hydroxybenzoate --> rn:R05616

rn:R09451 { Hexadecanoic acid } <-- [Reduced NADPH---hemoprotein reductase] -- rn:R01295 <-- 4-Hydroxybenzoate --> rn:R05616

rn:R09452 { (9Z)-Octadecenoic acid } <-- [Reduced NADPH---hemoprotein reductase] -- rn:R01295 <-- 4-Hydroxybenzoate --> rn:R05616

rn:R09922 { (+)-Linalool } <-- [Reduced NADPH---hemoprotein reductase] -- rn:R01295 <-- 4-Hydroxybenzoate --> rn:R05616

rn:R09923 { (-)-Linalool } <-- [Reduced NADPH---hemoprotein reductase] -- rn:R01295 <-- 4-Hydroxybenzoate --> rn:R05616

rn:R09925 { Linalool } <-- [Reduced NADPH---hemoprotein reductase] -- rn:R01295 <-- 4-Hydroxybenzoate --> rn:R05616

rn:R09934 { Humulene } <-- [Reduced NADPH---hemoprotein reductase] -- rn:R01295 <-- 4-Hydroxybenzoate --> rn:R05616

rn:R10562 { (E,E)-Geranyllinalool } <-- [Reduced NADPH---hemoprotein reductase] -- rn:R01295 <-- 4-Hydroxybenzoate --> rn:R05616

rn:R11055 { Myrcene } <-- [Reduced NADPH---hemoprotein reductase] -- rn:R01295 <-- 4-Hydroxybenzoate --> rn:R05616

rn:R02468 { (-)-Limonene } <-- [Reduced NADPH---hemoprotein reductase] -- rn:R01295 <-- 4-Hydroxybenzoate --> rn:R07273

rn:R02469 { (-)-Limonene } <-- [Reduced NADPH---hemoprotein reductase] -- rn:R01295 <-- 4-Hydroxybenzoate --> rn:R07273

rn:R02470 { (-)-Limonene } <-- [Reduced NADPH---hemoprotein reductase] -- rn:R01295 <-- 4-Hydroxybenzoate --> rn:R07273

rn:R04366 { Linalool } <-- [Reduced NADPH---hemoprotein reductase] -- rn:R01295 <-- 4-Hydroxybenzoate --> rn:R07273

rn:R06119 { d-Limonene } <-- [Reduced NADPH---hemoprotein reductase] -- rn:R01295 <-- 4-Hydroxybenzoate --> rn:R07273

rn:R09451 { Hexadecanoic acid } <-- [Reduced NADPH---hemoprotein reductase] -- rn:R01295 <-- 4-Hydroxybenzoate --> rn:R07273

rn:R09452 { (9Z)-Octadecenoic acid } <-- [Reduced NADPH---hemoprotein reductase] -- rn:R01295 <-- 4-Hydroxybenzoate --> rn:R07273

rn:R09922 { (+)-Linalool } <-- [Reduced NADPH---hemoprotein reductase] -- rn:R01295 <-- 4-Hydroxybenzoate --> rn:R07273

rn:R09923 { (-)-Linalool } <-- [Reduced NADPH---hemoprotein reductase] -- rn:R01295 <-- 4-Hydroxybenzoate --> rn:R07273

rn:R09925 { Linalool } <-- [Reduced NADPH---hemoprotein reductase] -- rn:R01295 <-- 4-Hydroxybenzoate --> rn:R07273

rn:R09934 { Humulene } <-- [Reduced NADPH---hemoprotein reductase] -- rn:R01295 <-- 4-Hydroxybenzoate --> rn:R07273

rn:R10562 { (E,E)-Geranyllinalool } <-- [Reduced NADPH---hemoprotein reductase] -- rn:R01295 <-- 4-Hydroxybenzoate --> rn:R07273

rn:R11055 { Myrcene } <-- [Reduced NADPH---hemoprotein reductase] -- rn:R01295 <-- 4-Hydroxybenzoate --> rn:R07273

rn:R02468 { (-)-Limonene } <-- [Reduced NADPH---hemoprotein reductase] -- rn:R01295 <-- [Oxidized NADPH---hemoprotein reductase] --> rn:R08551

rn:R02469 { (-)-Limonene } <-- [Reduced NADPH---hemoprotein reductase] -- rn:R01295 <-- [Oxidized NADPH---hemoprotein reductase] --> rn:R08551

rn:R02470 { (-)-Limonene } <-- [Reduced NADPH---hemoprotein reductase] -- rn:R01295 <-- [Oxidized NADPH---hemoprotein reductase] --> rn:R08551

rn:R04366 { Linalool } <-- [Reduced NADPH---hemoprotein reductase] -- rn:R01295 <-- [Oxidized NADPH---hemoprotein reductase] --> rn:R08551

rn:R06119 { d-Limonene } <-- [Reduced NADPH---hemoprotein reductase] -- rn:R01295 <-- [Oxidized NADPH---hemoprotein reductase] --> rn:R08551

rn:R09451 { Hexadecanoic acid } <-- [Reduced NADPH---hemoprotein reductase] -- rn:R01295 <-- [Oxidized NADPH---hemoprotein reductase] --> rn:R08551

rn:R09452 { (9Z)-Octadecenoic acid } <-- [Reduced NADPH---hemoprotein reductase] -- rn:R01295 <-- [Oxidized NADPH---hemoprotein reductase] --> rn:R08551

rn:R09922 { (+)-Linalool } <-- [Reduced NADPH---hemoprotein reductase] -- rn:R01295 <-- [Oxidized NADPH---hemoprotein reductase] --> rn:R08551

rn:R09923 { (-)-Linalool } <-- [Reduced NADPH---hemoprotein reductase] -- rn:R01295 <-- [Oxidized NADPH---hemoprotein reductase] --> rn:R08551

rn:R09925 { Linalool } <-- [Reduced NADPH---hemoprotein reductase] -- rn:R01295 <-- [Oxidized NADPH---hemoprotein reductase] --> rn:R08551

rn:R09934 { Humulene } <-- [Reduced NADPH---hemoprotein reductase] -- rn:R01295 <-- [Oxidized NADPH---hemoprotein reductase] --> rn:R08551

rn:R10562 { (E,E)-Geranyllinalool } <-- [Reduced NADPH---hemoprotein reductase] -- rn:R01295 <-- [Oxidized NADPH---hemoprotein reductase] --> rn:R08551

rn:R11055 { Myrcene } <-- [Reduced NADPH---hemoprotein reductase] -- rn:R01295 <-- [Oxidized NADPH---hemoprotein reductase] --> rn:R08551

rn:R02468 { (-)-Limonene } <-- [Reduced NADPH---hemoprotein reductase] -- rn:R01295 <-- 4-Hydroxybenzoate --> rn:R09040

rn:R02469 { (-)-Limonene } <-- [Reduced NADPH---hemoprotein reductase] -- rn:R01295 <-- 4-Hydroxybenzoate --> rn:R09040

rn:R02470 { (-)-Limonene } <-- [Reduced NADPH---hemoprotein reductase] -- rn:R01295 <-- 4-Hydroxybenzoate --> rn:R09040

rn:R04366 { Linalool } <-- [Reduced NADPH---hemoprotein reductase] -- rn:R01295 <-- 4-Hydroxybenzoate --> rn:R09040

rn:R06119 { d-Limonene } <-- [Reduced NADPH---hemoprotein reductase] -- rn:R01295 <-- 4-Hydroxybenzoate --> rn:R09040

rn:R09451 { Hexadecanoic acid } <-- [Reduced NADPH---hemoprotein reductase] -- rn:R01295 <-- 4-Hydroxybenzoate --> rn:R09040

rn:R09452 { (9Z)-Octadecenoic acid } <-- [Reduced NADPH---hemoprotein reductase] -- rn:R01295 <-- 4-Hydroxybenzoate --> rn:R09040

rn:R09922 { (+)-Linalool } <-- [Reduced NADPH---hemoprotein reductase] -- rn:R01295 <-- 4-Hydroxybenzoate --> rn:R09040

rn:R09923 { (-)-Linalool } <-- [Reduced NADPH---hemoprotein reductase] -- rn:R01295 <-- 4-Hydroxybenzoate --> rn:R09040

rn:R09925 { Linalool } <-- [Reduced NADPH---hemoprotein reductase] -- rn:R01295 <-- 4-Hydroxybenzoate --> rn:R09040

rn:R09934 { Humulene } <-- [Reduced NADPH---hemoprotein reductase] -- rn:R01295 <-- 4-Hydroxybenzoate --> rn:R09040

rn:R10562 { (E,E)-Geranyllinalool } <-- [Reduced NADPH---hemoprotein reductase] -- rn:R01295 <-- 4-Hydroxybenzoate --> rn:R09040

rn:R11055 { Myrcene } <-- [Reduced NADPH---hemoprotein reductase] -- rn:R01295 <-- 4-Hydroxybenzoate --> rn:R09040

rn:R02468 { (-)-Limonene } <-- [Reduced NADPH---hemoprotein reductase] -- rn:R01295 <-- 4-Hydroxybenzoate --> rn:R11102

rn:R02469 { (-)-Limonene } <-- [Reduced NADPH---hemoprotein reductase] -- rn:R01295 <-- 4-Hydroxybenzoate --> rn:R11102

rn:R02470 { (-)-Limonene } <-- [Reduced NADPH---hemoprotein reductase] -- rn:R01295 <-- 4-Hydroxybenzoate --> rn:R11102

rn:R04366 { Linalool } <-- [Reduced NADPH---hemoprotein reductase] -- rn:R01295 <-- 4-Hydroxybenzoate --> rn:R11102

rn:R06119 { d-Limonene } <-- [Reduced NADPH---hemoprotein reductase] -- rn:R01295 <-- 4-Hydroxybenzoate --> rn:R11102

rn:R09451 { Hexadecanoic acid } <-- [Reduced NADPH---hemoprotein reductase] -- rn:R01295 <-- 4-Hydroxybenzoate --> rn:R11102

rn:R09452 { (9Z)-Octadecenoic acid } <-- [Reduced NADPH---hemoprotein reductase] -- rn:R01295 <-- 4-Hydroxybenzoate --> rn:R11102

rn:R09922 { (+)-Linalool } <-- [Reduced NADPH---hemoprotein reductase] -- rn:R01295 <-- 4-Hydroxybenzoate --> rn:R11102

rn:R09923 { (-)-Linalool } <-- [Reduced NADPH---hemoprotein reductase] -- rn:R01295 <-- 4-Hydroxybenzoate --> rn:R11102

rn:R09925 { Linalool } <-- [Reduced NADPH---hemoprotein reductase] -- rn:R01295 <-- 4-Hydroxybenzoate --> rn:R11102

rn:R09934 { Humulene } <-- [Reduced NADPH---hemoprotein reductase] -- rn:R01295 <-- 4-Hydroxybenzoate --> rn:R11102

rn:R10562 { (E,E)-Geranyllinalool } <-- [Reduced NADPH---hemoprotein reductase] -- rn:R01295 <-- 4-Hydroxybenzoate --> rn:R11102

rn:R11055 { Myrcene } <-- [Reduced NADPH---hemoprotein reductase] -- rn:R01295 <-- 4-Hydroxybenzoate --> rn:R11102

rn:R02468 { (-)-Limonene } <-- [Reduced NADPH---hemoprotein reductase] -- rn:R01295 <-- 4-Hydroxybenzoate --> rn:R11608

rn:R02469 { (-)-Limonene } <-- [Reduced NADPH---hemoprotein reductase] -- rn:R01295 <-- 4-Hydroxybenzoate --> rn:R11608

rn:R02470 { (-)-Limonene } <-- [Reduced NADPH---hemoprotein reductase] -- rn:R01295 <-- 4-Hydroxybenzoate --> rn:R11608

rn:R04366 { Linalool } <-- [Reduced NADPH---hemoprotein reductase] -- rn:R01295 <-- 4-Hydroxybenzoate --> rn:R11608

rn:R06119 { d-Limonene } <-- [Reduced NADPH---hemoprotein reductase] -- rn:R01295 <-- 4-Hydroxybenzoate --> rn:R11608

rn:R09451 { Hexadecanoic acid } <-- [Reduced NADPH---hemoprotein reductase] -- rn:R01295 <-- 4-Hydroxybenzoate --> rn:R11608

rn:R09452 { (9Z)-Octadecenoic acid } <-- [Reduced NADPH---hemoprotein reductase] -- rn:R01295 <-- 4-Hydroxybenzoate --> rn:R11608

rn:R09922 { (+)-Linalool } <-- [Reduced NADPH---hemoprotein reductase] -- rn:R01295 <-- 4-Hydroxybenzoate --> rn:R11608

rn:R09923 { (-)-Linalool } <-- [Reduced NADPH---hemoprotein reductase] -- rn:R01295 <-- 4-Hydroxybenzoate --> rn:R11608

rn:R09925 { Linalool } <-- [Reduced NADPH---hemoprotein reductase] -- rn:R01295 <-- 4-Hydroxybenzoate --> rn:R11608

rn:R09934 { Humulene } <-- [Reduced NADPH---hemoprotein reductase] -- rn:R01295 <-- 4-Hydroxybenzoate --> rn:R11608

rn:R10562 { (E,E)-Geranyllinalool } <-- [Reduced NADPH---hemoprotein reductase] -- rn:R01295 <-- 4-Hydroxybenzoate --> rn:R11608

rn:R11055 { Myrcene } <-- [Reduced NADPH---hemoprotein reductase] -- rn:R01295 <-- 4-Hydroxybenzoate --> rn:R11608

rn:R02468 { (-)-Limonene } <-- [Reduced NADPH---hemoprotein reductase] -- rn:R01295 <-- 4-Hydroxybenzoate --> rn:R11872

rn:R02469 { (-)-Limonene } <-- [Reduced NADPH---hemoprotein reductase] -- rn:R01295 <-- 4-Hydroxybenzoate --> rn:R11872

rn:R02470 { (-)-Limonene } <-- [Reduced NADPH---hemoprotein reductase] -- rn:R01295 <-- 4-Hydroxybenzoate --> rn:R11872

rn:R04366 { Linalool } <-- [Reduced NADPH---hemoprotein reductase] -- rn:R01295 <-- 4-Hydroxybenzoate --> rn:R11872

rn:R06119 { d-Limonene } <-- [Reduced NADPH---hemoprotein reductase] -- rn:R01295 <-- 4-Hydroxybenzoate --> rn:R11872

rn:R09451 { Hexadecanoic acid } <-- [Reduced NADPH---hemoprotein reductase] -- rn:R01295 <-- 4-Hydroxybenzoate --> rn:R11872

rn:R09452 { (9Z)-Octadecenoic acid } <-- [Reduced NADPH---hemoprotein reductase] -- rn:R01295 <-- 4-Hydroxybenzoate --> rn:R11872

rn:R09922 { (+)-Linalool } <-- [Reduced NADPH---hemoprotein reductase] -- rn:R01295 <-- 4-Hydroxybenzoate --> rn:R11872

rn:R09923 { (-)-Linalool } <-- [Reduced NADPH---hemoprotein reductase] -- rn:R01295 <-- 4-Hydroxybenzoate --> rn:R11872

rn:R09925 { Linalool } <-- [Reduced NADPH---hemoprotein reductase] -- rn:R01295 <-- 4-Hydroxybenzoate --> rn:R11872

rn:R09934 { Humulene } <-- [Reduced NADPH---hemoprotein reductase] -- rn:R01295 <-- 4-Hydroxybenzoate --> rn:R11872

rn:R10562 { (E,E)-Geranyllinalool } <-- [Reduced NADPH---hemoprotein reductase] -- rn:R01295 <-- 4-Hydroxybenzoate --> rn:R11872

rn:R11055 { Myrcene } <-- [Reduced NADPH---hemoprotein reductase] -- rn:R01295 <-- 4-Hydroxybenzoate --> rn:R11872

rn:R02468 { (-)-Limonene } <-- [Reduced NADPH---hemoprotein reductase] -- rn:R01295 <-- 4-Hydroxybenzoate --> rn:R12013

rn:R02469 { (-)-Limonene } <-- [Reduced NADPH---hemoprotein reductase] -- rn:R01295 <-- 4-Hydroxybenzoate --> rn:R12013

rn:R02470 { (-)-Limonene } <-- [Reduced NADPH---hemoprotein reductase] -- rn:R01295 <-- 4-Hydroxybenzoate --> rn:R12013

rn:R04366 { Linalool } <-- [Reduced NADPH---hemoprotein reductase] -- rn:R01295 <-- 4-Hydroxybenzoate --> rn:R12013

rn:R06119 { d-Limonene } <-- [Reduced NADPH---hemoprotein reductase] -- rn:R01295 <-- 4-Hydroxybenzoate --> rn:R12013

rn:R09451 { Hexadecanoic acid } <-- [Reduced NADPH---hemoprotein reductase] -- rn:R01295 <-- 4-Hydroxybenzoate --> rn:R12013

rn:R09452 { (9Z)-Octadecenoic acid } <-- [Reduced NADPH---hemoprotein reductase] -- rn:R01295 <-- 4-Hydroxybenzoate --> rn:R12013

rn:R09922 { (+)-Linalool } <-- [Reduced NADPH---hemoprotein reductase] -- rn:R01295 <-- 4-Hydroxybenzoate --> rn:R12013

rn:R09923 { (-)-Linalool } <-- [Reduced NADPH---hemoprotein reductase] -- rn:R01295 <-- 4-Hydroxybenzoate --> rn:R12013

rn:R09925 { Linalool } <-- [Reduced NADPH---hemoprotein reductase] -- rn:R01295 <-- 4-Hydroxybenzoate --> rn:R12013

rn:R09934 { Humulene } <-- [Reduced NADPH---hemoprotein reductase] -- rn:R01295 <-- 4-Hydroxybenzoate --> rn:R12013

rn:R10562 { (E,E)-Geranyllinalool } <-- [Reduced NADPH---hemoprotein reductase] -- rn:R01295 <-- 4-Hydroxybenzoate --> rn:R12013

rn:R11055 { Myrcene } <-- [Reduced NADPH---hemoprotein reductase] -- rn:R01295 <-- 4-Hydroxybenzoate --> rn:R12013

rn:R02468 { (-)-Limonene } <-- [Reduced NADPH---hemoprotein reductase] -- rn:R01295 <-- 4-Hydroxybenzoate --> rn:R12015

rn:R02469 { (-)-Limonene } <-- [Reduced NADPH---hemoprotein reductase] -- rn:R01295 <-- 4-Hydroxybenzoate --> rn:R12015

rn:R02470 { (-)-Limonene } <-- [Reduced NADPH---hemoprotein reductase] -- rn:R01295 <-- 4-Hydroxybenzoate --> rn:R12015

rn:R04366 { Linalool } <-- [Reduced NADPH---hemoprotein reductase] -- rn:R01295 <-- 4-Hydroxybenzoate --> rn:R12015

rn:R06119 { d-Limonene } <-- [Reduced NADPH---hemoprotein reductase] -- rn:R01295 <-- 4-Hydroxybenzoate --> rn:R12015

rn:R09451 { Hexadecanoic acid } <-- [Reduced NADPH---hemoprotein reductase] -- rn:R01295 <-- 4-Hydroxybenzoate --> rn:R12015

rn:R09452 { (9Z)-Octadecenoic acid } <-- [Reduced NADPH---hemoprotein reductase] -- rn:R01295 <-- 4-Hydroxybenzoate --> rn:R12015

rn:R09922 { (+)-Linalool } <-- [Reduced NADPH---hemoprotein reductase] -- rn:R01295 <-- 4-Hydroxybenzoate --> rn:R12015

rn:R09923 { (-)-Linalool } <-- [Reduced NADPH---hemoprotein reductase] -- rn:R01295 <-- 4-Hydroxybenzoate --> rn:R12015

rn:R09925 { Linalool } <-- [Reduced NADPH---hemoprotein reductase] -- rn:R01295 <-- 4-Hydroxybenzoate --> rn:R12015

rn:R09934 { Humulene } <-- [Reduced NADPH---hemoprotein reductase] -- rn:R01295 <-- 4-Hydroxybenzoate --> rn:R12015

rn:R10562 { (E,E)-Geranyllinalool } <-- [Reduced NADPH---hemoprotein reductase] -- rn:R01295 <-- 4-Hydroxybenzoate --> rn:R12015

rn:R11055 { Myrcene } <-- [Reduced NADPH---hemoprotein reductase] -- rn:R01295 <-- 4-Hydroxybenzoate --> rn:R12015

rn:R05136 { Salicylaldehyde } <-- Pyruvate -- rn:R01302 <-- Chorismate --> rn:R00986

rn:R05136 { Salicylaldehyde } <-- Pyruvate -- rn:R01302 <-- Chorismate --> rn:R01715

rn:R05136 { Salicylaldehyde } <-- Pyruvate -- rn:R01302 <-- Chorismate --> rn:R01717

rn:R05136 { Salicylaldehyde } <-- Pyruvate -- rn:R01302 <-- Chorismate --> rn:R06603

rn:R05136 { Salicylaldehyde } <-- Pyruvate -- rn:R01302 <-- Chorismate --> rn:R10583

rn:R05136 { Salicylaldehyde } <-- Pyruvate -- rn:R01302 <-- Chorismate --> rn:R10666

rn:R02234 { Cyclohexanone } <-- Acceptor -- rn:R01303 <-- 4-Hydroxybenzaldehyde --> rn:R01293

rn:R03212 { 3-Hydroxycyclohexanone } <-- Acceptor -- rn:R01303 <-- 4-Hydroxybenzaldehyde --> rn:R01293

rn:R02234 { Cyclohexanone } <-- Acceptor -- rn:R01303 <-- 4-Hydroxybenzaldehyde --> rn:R01294

rn:R03212 { 3-Hydroxycyclohexanone } <-- Acceptor -- rn:R01303 <-- 4-Hydroxybenzaldehyde --> rn:R01294

rn:R02179 { (-)-Menthol } <-- UDP-glucose -- rn:R01304 <-- 4-(beta-D-Glucosyloxy)benzoate --> rn:R09039

rn:R03548 { 7,8-Dihydroxycoumarin } <-- UDP-glucose -- rn:R01304 <-- 4-(beta-D-Glucosyloxy)benzoate --> rn:R09039

rn:R02468 { (-)-Limonene } <-- [Reduced NADPH---hemoprotein reductase] -- rn:R01348 <-- [Oxidized NADPH---hemoprotein reductase] --> rn:R08551

rn:R02469 { (-)-Limonene } <-- [Reduced NADPH---hemoprotein reductase] -- rn:R01348 <-- [Oxidized NADPH---hemoprotein reductase] --> rn:R08551

rn:R02470 { (-)-Limonene } <-- [Reduced NADPH---hemoprotein reductase] -- rn:R01348 <-- [Oxidized NADPH---hemoprotein reductase] --> rn:R08551

rn:R04366 { Linalool } <-- [Reduced NADPH---hemoprotein reductase] -- rn:R01348 <-- [Oxidized NADPH---hemoprotein reductase] --> rn:R08551

rn:R06119 { d-Limonene } <-- [Reduced NADPH---hemoprotein reductase] -- rn:R01348 <-- [Oxidized NADPH---hemoprotein reductase] --> rn:R08551

rn:R09451 { Hexadecanoic acid } <-- [Reduced NADPH---hemoprotein reductase] -- rn:R01348 <-- [Oxidized NADPH---hemoprotein reductase] --> rn:R08551

rn:R09452 { (9Z)-Octadecenoic acid } <-- [Reduced NADPH---hemoprotein reductase] -- rn:R01348 <-- [Oxidized NADPH---hemoprotein reductase] --> rn:R08551

rn:R09922 { (+)-Linalool } <-- [Reduced NADPH---hemoprotein reductase] -- rn:R01348 <-- [Oxidized NADPH---hemoprotein reductase] --> rn:R08551

rn:R09923 { (-)-Linalool } <-- [Reduced NADPH---hemoprotein reductase] -- rn:R01348 <-- [Oxidized NADPH---hemoprotein reductase] --> rn:R08551

rn:R09925 { Linalool } <-- [Reduced NADPH---hemoprotein reductase] -- rn:R01348 <-- [Oxidized NADPH---hemoprotein reductase] --> rn:R08551

rn:R09934 { Humulene } <-- [Reduced NADPH---hemoprotein reductase] -- rn:R01348 <-- [Oxidized NADPH---hemoprotein reductase] --> rn:R08551

rn:R10562 { (E,E)-Geranyllinalool } <-- [Reduced NADPH---hemoprotein reductase] -- rn:R01348 <-- [Oxidized NADPH---hemoprotein reductase] --> rn:R08551

rn:R11055 { Myrcene } <-- [Reduced NADPH---hemoprotein reductase] -- rn:R01348 <-- [Oxidized NADPH---hemoprotein reductase] --> rn:R08551

rn:R02781 { 2,4,6/3,5-Pentahydroxycyclohexanone } <-- L-Glutamine -- rn:R01375 <-- L-Phenylalanine --> rn:R00686

rn:R02781 { 2,4,6/3,5-Pentahydroxycyclohexanone } <-- L-Glutamine -- rn:R01375 <-- L-Phenylalanine --> rn:R00688

rn:R02781 { 2,4,6/3,5-Pentahydroxycyclohexanone } <-- L-Glutamine -- rn:R01375 <-- L-Phenylalanine --> rn:R00689

rn:R02781 { 2,4,6/3,5-Pentahydroxycyclohexanone } <-- L-Glutamine -- rn:R01375 <-- L-Phenylalanine --> rn:R00690

rn:R02781 { 2,4,6/3,5-Pentahydroxycyclohexanone } <-- L-Glutamine -- rn:R01375 <-- L-Phenylalanine --> rn:R00692

rn:R02781 { 2,4,6/3,5-Pentahydroxycyclohexanone } <-- L-Glutamine -- rn:R01375 <-- L-Phenylalanine --> rn:R00693

rn:R02781 { 2,4,6/3,5-Pentahydroxycyclohexanone } <-- L-Glutamine -- rn:R01375 <-- L-Phenylalanine --> rn:R00694

rn:R02781 { 2,4,6/3,5-Pentahydroxycyclohexanone } <-- L-Glutamine -- rn:R01375 <-- L-Phenylalanine --> rn:R00697

rn:R02781 { 2,4,6/3,5-Pentahydroxycyclohexanone } <-- L-Glutamine -- rn:R01375 <-- L-Phenylalanine --> rn:R00698

rn:R02781 { 2,4,6/3,5-Pentahydroxycyclohexanone } <-- L-Glutamine -- rn:R01375 <-- L-Phenylalanine --> rn:R00699

rn:R02781 { 2,4,6/3,5-Pentahydroxycyclohexanone } <-- L-Glutamine -- rn:R01375 <-- L-Phenylalanine --> rn:R08463

rn:R02781 { 2,4,6/3,5-Pentahydroxycyclohexanone } <-- L-Glutamine -- rn:R01375 <-- L-Phenylalanine --> rn:R08652

rn:R02781 { 2,4,6/3,5-Pentahydroxycyclohexanone } <-- L-Glutamine -- rn:R01375 <-- L-Phenylalanine --> rn:R08690

rn:R02781 { 2,4,6/3,5-Pentahydroxycyclohexanone } <-- L-Glutamine -- rn:R01375 <-- L-Phenylalanine --> rn:R09578

rn:R02781 { 2,4,6/3,5-Pentahydroxycyclohexanone } <-- L-Glutamine -- rn:R01375 <-- L-Phenylalanine --> rn:R09579

rn:R02781 { 2,4,6/3,5-Pentahydroxycyclohexanone } <-- L-Glutamine -- rn:R01375 <-- L-Phenylalanine --> rn:R10495

rn:R02781 { 2,4,6/3,5-Pentahydroxycyclohexanone } <-- L-Glutamine -- rn:R01375 <-- L-Phenylalanine --> rn:R10499

rn:R02781 { 2,4,6/3,5-Pentahydroxycyclohexanone } <-- L-Glutamine -- rn:R01375 <-- L-Phenylalanine --> rn:R11068

rn:R02781 { 2,4,6/3,5-Pentahydroxycyclohexanone } <-- L-Glutamine -- rn:R01375 <-- L-Phenylalanine --> rn:R11918

rn:R01419 { Benzaldehyde } <-- Benzaldehyde -- rn:R01419 <-- Benzoate --> rn:R01295

rn:R01420 { Benzaldehyde } <-- Benzaldehyde -- rn:R01419 <-- Benzoate --> rn:R01295

rn:R01419 { Benzaldehyde } <-- Benzaldehyde -- rn:R01419 <-- Benzoate --> rn:R01422

rn:R01420 { Benzaldehyde } <-- Benzaldehyde -- rn:R01419 <-- Benzoate --> rn:R01422

rn:R01419 { Benzaldehyde } <-- Benzaldehyde -- rn:R01419 <-- Benzoate --> rn:R01426

rn:R01420 { Benzaldehyde } <-- Benzaldehyde -- rn:R01419 <-- Benzoate --> rn:R01426

rn:R01419 { Benzaldehyde } <-- Benzaldehyde -- rn:R01419 <-- Benzoate --> rn:R01427

rn:R01420 { Benzaldehyde } <-- Benzaldehyde -- rn:R01419 <-- Benzoate --> rn:R01427

rn:R01419 { Benzaldehyde } <-- Benzaldehyde -- rn:R01419 <-- Benzoate --> rn:R05621

rn:R01420 { Benzaldehyde } <-- Benzaldehyde -- rn:R01419 <-- Benzoate --> rn:R05621

rn:R01419 { Benzaldehyde } <-- Benzaldehyde -- rn:R01419 <-- Benzoate --> rn:R05622

rn:R01420 { Benzaldehyde } <-- Benzaldehyde -- rn:R01419 <-- Benzoate --> rn:R05622

rn:R01419 { Benzaldehyde } <-- Benzaldehyde -- rn:R01419 <-- Benzoate --> rn:R06727

rn:R01420 { Benzaldehyde } <-- Benzaldehyde -- rn:R01419 <-- Benzoate --> rn:R06727

rn:R01419 { Benzaldehyde } <-- Benzaldehyde -- rn:R01419 <-- Benzoate --> rn:R07188

rn:R01420 { Benzaldehyde } <-- Benzaldehyde -- rn:R01419 <-- Benzoate --> rn:R07188

rn:R01419 { Benzaldehyde } <-- Benzaldehyde -- rn:R01419 <-- Benzoate --> rn:R08838

rn:R01420 { Benzaldehyde } <-- Benzaldehyde -- rn:R01419 <-- Benzoate --> rn:R08838

rn:R01419 { Benzaldehyde } <-- Benzaldehyde -- rn:R01419 <-- Benzoate --> rn:R09088

rn:R01420 { Benzaldehyde } <-- Benzaldehyde -- rn:R01419 <-- Benzoate --> rn:R09088

rn:R01419 { Benzaldehyde } <-- Benzaldehyde -- rn:R01419 <-- Benzoate --> rn:R10447

rn:R01420 { Benzaldehyde } <-- Benzaldehyde -- rn:R01419 <-- Benzoate --> rn:R10447

rn:R01419 { Benzaldehyde } <-- Benzaldehyde -- rn:R01420 <-- Benzoate --> rn:R01295

rn:R01420 { Benzaldehyde } <-- Benzaldehyde -- rn:R01420 <-- Benzoate --> rn:R01295

rn:R01419 { Benzaldehyde } <-- Benzaldehyde -- rn:R01420 <-- Benzoate --> rn:R01422

rn:R01420 { Benzaldehyde } <-- Benzaldehyde -- rn:R01420 <-- Benzoate --> rn:R01422

rn:R01419 { Benzaldehyde } <-- Benzaldehyde -- rn:R01420 <-- Benzoate --> rn:R01426

rn:R01420 { Benzaldehyde } <-- Benzaldehyde -- rn:R01420 <-- Benzoate --> rn:R01426

rn:R01419 { Benzaldehyde } <-- Benzaldehyde -- rn:R01420 <-- Benzoate --> rn:R01427

rn:R01420 { Benzaldehyde } <-- Benzaldehyde -- rn:R01420 <-- Benzoate --> rn:R01427

rn:R01419 { Benzaldehyde } <-- Benzaldehyde -- rn:R01420 <-- Benzoate --> rn:R05621

rn:R01420 { Benzaldehyde } <-- Benzaldehyde -- rn:R01420 <-- Benzoate --> rn:R05621

rn:R01419 { Benzaldehyde } <-- Benzaldehyde -- rn:R01420 <-- Benzoate --> rn:R05622

rn:R01420 { Benzaldehyde } <-- Benzaldehyde -- rn:R01420 <-- Benzoate --> rn:R05622

rn:R01419 { Benzaldehyde } <-- Benzaldehyde -- rn:R01420 <-- Benzoate --> rn:R06727

rn:R01420 { Benzaldehyde } <-- Benzaldehyde -- rn:R01420 <-- Benzoate --> rn:R06727

rn:R01419 { Benzaldehyde } <-- Benzaldehyde -- rn:R01420 <-- Benzoate --> rn:R07188

rn:R01420 { Benzaldehyde } <-- Benzaldehyde -- rn:R01420 <-- Benzoate --> rn:R07188

rn:R01419 { Benzaldehyde } <-- Benzaldehyde -- rn:R01420 <-- Benzoate --> rn:R08838

rn:R01420 { Benzaldehyde } <-- Benzaldehyde -- rn:R01420 <-- Benzoate --> rn:R08838

rn:R01419 { Benzaldehyde } <-- Benzaldehyde -- rn:R01420 <-- Benzoate --> rn:R09088

rn:R01420 { Benzaldehyde } <-- Benzaldehyde -- rn:R01420 <-- Benzoate --> rn:R09088

rn:R01419 { Benzaldehyde } <-- Benzaldehyde -- rn:R01420 <-- Benzoate --> rn:R10447

rn:R01420 { Benzaldehyde } <-- Benzaldehyde -- rn:R01420 <-- Benzoate --> rn:R10447

rn:R05136 { Salicylaldehyde } <-- Pyruvate -- rn:R01712 <-- Pyridoxal --> rn:R00174

rn:R05136 { Salicylaldehyde } <-- Pyruvate -- rn:R01712 <-- Pyridoxal --> rn:R01707

rn:R05136 { Salicylaldehyde } <-- Pyruvate -- rn:R01712 <-- Pyridoxal --> rn:R01709

rn:R05136 { Salicylaldehyde } <-- Pyruvate -- rn:R01712 <-- L-Alanine --> rn:R04187

rn:R05136 { Salicylaldehyde } <-- Pyruvate -- rn:R01712 <-- L-Alanine --> rn:R08872

rn:R05136 { Salicylaldehyde } <-- Pyruvate -- rn:R02050 <-- 2-Methyl-3-oxopropanoate --> rn:R00922

rn:R05136 { Salicylaldehyde } <-- Pyruvate -- rn:R02050 <-- L-Alanine --> rn:R04187

rn:R05136 { Salicylaldehyde } <-- Pyruvate -- rn:R02050 <-- L-Alanine --> rn:R08872

rn:R02177 { (-)-Menthol } <-- (-)-Menthol -- rn:R02177 <-- (-)-Menthone --> rn:R08925

rn:R02178 { (-)-Menthol } <-- (-)-Menthol -- rn:R02177 <-- (-)-Menthone --> rn:R08925

rn:R02179 { (-)-Menthol } <-- (-)-Menthol -- rn:R02177 <-- (-)-Menthone --> rn:R08925

rn:R08530 { (-)-Menthol } <-- (-)-Menthol -- rn:R02177 <-- (-)-Menthone --> rn:R08925

rn:R02468 { (-)-Limonene } <-- [Reduced NADPH---hemoprotein reductase] -- rn:R02253 <-- [Oxidized NADPH---hemoprotein reductase] --> rn:R08551

rn:R02469 { (-)-Limonene } <-- [Reduced NADPH---hemoprotein reductase] -- rn:R02253 <-- [Oxidized NADPH---hemoprotein reductase] --> rn:R08551

rn:R02470 { (-)-Limonene } <-- [Reduced NADPH---hemoprotein reductase] -- rn:R02253 <-- [Oxidized NADPH---hemoprotein reductase] --> rn:R08551

rn:R04366 { Linalool } <-- [Reduced NADPH---hemoprotein reductase] -- rn:R02253 <-- [Oxidized NADPH---hemoprotein reductase] --> rn:R08551

rn:R06119 { d-Limonene } <-- [Reduced NADPH---hemoprotein reductase] -- rn:R02253 <-- [Oxidized NADPH---hemoprotein reductase] --> rn:R08551

rn:R09451 { Hexadecanoic acid } <-- [Reduced NADPH---hemoprotein reductase] -- rn:R02253 <-- [Oxidized NADPH---hemoprotein reductase] --> rn:R08551

rn:R09452 { (9Z)-Octadecenoic acid } <-- [Reduced NADPH---hemoprotein reductase] -- rn:R02253 <-- [Oxidized NADPH---hemoprotein reductase] --> rn:R08551

rn:R09922 { (+)-Linalool } <-- [Reduced NADPH---hemoprotein reductase] -- rn:R02253 <-- [Oxidized NADPH---hemoprotein reductase] --> rn:R08551

rn:R09923 { (-)-Linalool } <-- [Reduced NADPH---hemoprotein reductase] -- rn:R02253 <-- [Oxidized NADPH---hemoprotein reductase] --> rn:R08551

rn:R09925 { Linalool } <-- [Reduced NADPH---hemoprotein reductase] -- rn:R02253 <-- [Oxidized NADPH---hemoprotein reductase] --> rn:R08551

rn:R09934 { Humulene } <-- [Reduced NADPH---hemoprotein reductase] -- rn:R02253 <-- [Oxidized NADPH---hemoprotein reductase] --> rn:R08551

rn:R10562 { (E,E)-Geranyllinalool } <-- [Reduced NADPH---hemoprotein reductase] -- rn:R02253 <-- [Oxidized NADPH---hemoprotein reductase] --> rn:R08551

rn:R11055 { Myrcene } <-- [Reduced NADPH---hemoprotein reductase] -- rn:R02253 <-- [Oxidized NADPH---hemoprotein reductase] --> rn:R08551

rn:R05136 { Salicylaldehyde } <-- Pyruvate -- rn:R02271 <-- L-Alanine --> rn:R04187

rn:R05136 { Salicylaldehyde } <-- Pyruvate -- rn:R02271 <-- L-Alanine --> rn:R08872

rn:R08436 { 3,4-Dihydroxybenzaldehyde } <-- Tyramine -- rn:R02382 <-- Hydrogen peroxide --> rn:R00069

rn:R08436 { 3,4-Dihydroxybenzaldehyde } <-- Tyramine -- rn:R02382 <-- Hydrogen peroxide --> rn:R00113

rn:R08436 { 3,4-Dihydroxybenzaldehyde } <-- Tyramine -- rn:R02382 <-- Hydrogen peroxide --> rn:R00274

rn:R08436 { 3,4-Dihydroxybenzaldehyde } <-- Tyramine -- rn:R02382 <-- Hydrogen peroxide --> rn:R00644

rn:R08436 { 3,4-Dihydroxybenzaldehyde } <-- Tyramine -- rn:R02382 <-- Hydrogen peroxide --> rn:R02657

rn:R08436 { 3,4-Dihydroxybenzaldehyde } <-- Tyramine -- rn:R02382 <-- 4-Hydroxyphenylacetaldehyde --> rn:R02695

rn:R08436 { 3,4-Dihydroxybenzaldehyde } <-- Tyramine -- rn:R02382 <-- 4-Hydroxyphenylacetaldehyde --> rn:R02697

rn:R08436 { 3,4-Dihydroxybenzaldehyde } <-- Tyramine -- rn:R02382 <-- Hydrogen peroxide --> rn:R03208

rn:R08436 { 3,4-Dihydroxybenzaldehyde } <-- Tyramine -- rn:R02382 <-- Hydrogen peroxide --> rn:R03953

rn:R08436 { 3,4-Dihydroxybenzaldehyde } <-- Tyramine -- rn:R02382 <-- 4-Hydroxyphenylacetaldehyde --> rn:R04305

rn:R08436 { 3,4-Dihydroxybenzaldehyde } <-- Tyramine -- rn:R02382 <-- Hydrogen peroxide --> rn:R08863

rn:R08436 { 3,4-Dihydroxybenzaldehyde } <-- Tyramine -- rn:R02382 <-- Hydrogen peroxide --> rn:R11503

rn:R08436 { 3,4-Dihydroxybenzaldehyde } <-- Tyramine -- rn:R02382 <-- Hydrogen peroxide --> rn:R11522

rn:R08436 { 3,4-Dihydroxybenzaldehyde } <-- Tyramine -- rn:R02383 <-- Dopamine --> rn:R02535

rn:R08436 { 3,4-Dihydroxybenzaldehyde } <-- Tyramine -- rn:R02383 <-- Dopamine --> rn:R04084

rn:R08436 { 3,4-Dihydroxybenzaldehyde } <-- Tyramine -- rn:R02383 <-- Dopamine --> rn:R04300

rn:R08436 { 3,4-Dihydroxybenzaldehyde } <-- Tyramine -- rn:R02383 <-- Dopamine --> rn:R04301

rn:R08436 { 3,4-Dihydroxybenzaldehyde } <-- Tyramine -- rn:R02383 <-- Dopamine --> rn:R04305

rn:R08436 { 3,4-Dihydroxybenzaldehyde } <-- Tyramine -- rn:R02383 <-- Dopamine --> rn:R05749

rn:R08436 { 3,4-Dihydroxybenzaldehyde } <-- Tyramine -- rn:R02383 <-- Dopamine --> rn:R05750

rn:R08436 { 3,4-Dihydroxybenzaldehyde } <-- Tyramine -- rn:R02383 <-- Dopamine --> rn:R08444

rn:R08436 { 3,4-Dihydroxybenzaldehyde } <-- Tyramine -- rn:R02383 <-- Dopamine --> rn:R08445

rn:R08436 { 3,4-Dihydroxybenzaldehyde } <-- Tyramine -- rn:R02383 <-- Dopamine --> rn:R08447

rn:R08436 { 3,4-Dihydroxybenzaldehyde } <-- Tyramine -- rn:R02383 <-- Dopamine --> rn:R08513

rn:R08436 { 3,4-Dihydroxybenzaldehyde } <-- Tyramine -- rn:R02383 <-- Dopamine --> rn:R08832

rn:R08436 { 3,4-Dihydroxybenzaldehyde } <-- Tyramine -- rn:R02383 <-- Dopamine --> rn:R08835

rn:R08436 { 3,4-Dihydroxybenzaldehyde } <-- Tyramine -- rn:R02383 <-- Dopamine --> rn:R11684

rn:R08436 { 3,4-Dihydroxybenzaldehyde } <-- Tyramine -- rn:R02384 <-- N-Methyltyramine --> rn:R03943

rn:R05537 { 3-(2-Hydroxyphenyl)propanoate } <-- FAD -- rn:R02487 <-- FADH2 --> rn:R03978

rn:R05537 { 3-(2-Hydroxyphenyl)propanoate } <-- FAD -- rn:R02487 <-- FADH2 --> rn:R05488

rn:R05537 { 3-(2-Hydroxyphenyl)propanoate } <-- FAD -- rn:R02487 <-- FADH2 --> rn:R09517

rn:R05537 { 3-(2-Hydroxyphenyl)propanoate } <-- FAD -- rn:R02487 <-- FADH2 --> rn:R11653

rn:R05537 { 3-(2-Hydroxyphenyl)propanoate } <-- FAD -- rn:R02487 <-- FADH2 --> rn:R12021

rn:R05537 { 3-(2-Hydroxyphenyl)propanoate } <-- FAD -- rn:R02487 <-- FADH2 --> rn:R12023

rn:R05537 { 3-(2-Hydroxyphenyl)propanoate } <-- FAD -- rn:R02487 <-- FADH2 --> rn:R12027

rn:R05537 { 3-(2-Hydroxyphenyl)propanoate } <-- FAD -- rn:R02487 <-- FADH2 --> rn:R12030

rn:R08447 { 4-Hydroxydihydrocinnamaldehyde } <-- Dopamine -- rn:R02535 <-- Dehydroascorbate --> rn:R01108

rn:R08447 { 4-Hydroxydihydrocinnamaldehyde } <-- Dopamine -- rn:R02535 <-- L-Noradrenaline --> rn:R02532

rn:R08447 { 4-Hydroxydihydrocinnamaldehyde } <-- Dopamine -- rn:R02535 <-- L-Noradrenaline --> rn:R02534

rn:R08447 { 4-Hydroxydihydrocinnamaldehyde } <-- Dopamine -- rn:R02535 <-- Dehydroascorbate --> rn:R04785

rn:R08447 { 4-Hydroxydihydrocinnamaldehyde } <-- Dopamine -- rn:R02535 <-- Dehydroascorbate --> rn:R08358

rn:R02234 { Cyclohexanone } <-- Acceptor -- rn:R02612 <-- Phenylacetaldehyde --> rn:R02536

rn:R03212 { 3-Hydroxycyclohexanone } <-- Acceptor -- rn:R02612 <-- Phenylacetaldehyde --> rn:R02536

rn:R02234 { Cyclohexanone } <-- Acceptor -- rn:R02612 <-- Phenylacetaldehyde --> rn:R02537

rn:R03212 { 3-Hydroxycyclohexanone } <-- Acceptor -- rn:R02612 <-- Phenylacetaldehyde --> rn:R02537

rn:R02615 { Styrene oxide } <-- Styrene oxide -- rn:R02615 <-- Phenylacetaldehyde --> rn:R02536

rn:R02615 { Styrene oxide } <-- Styrene oxide -- rn:R02615 <-- Phenylacetaldehyde --> rn:R02537

rn:R01703 { Hexadecanoic acid } <-- Hydrogen peroxide -- rn:R02657 <-- Gentisate aldehyde --> rn:R02655

rn:R02234 { Cyclohexanone } <-- Acceptor -- rn:R02661 <-- 2-Methylprop-2-enoyl-CoA --> rn:R04224

rn:R03212 { 3-Hydroxycyclohexanone } <-- Acceptor -- rn:R02661 <-- 2-Methylprop-2-enoyl-CoA --> rn:R04224

rn:R02468 { (-)-Limonene } <-- [Reduced NADPH---hemoprotein reductase] -- rn:R02708 <-- (S)-4-Hydroxymandelonitrile --> rn:R02676

rn:R02469 { (-)-Limonene } <-- [Reduced NADPH---hemoprotein reductase] -- rn:R02708 <-- (S)-4-Hydroxymandelonitrile --> rn:R02676

rn:R02470 { (-)-Limonene } <-- [Reduced NADPH---hemoprotein reductase] -- rn:R02708 <-- (S)-4-Hydroxymandelonitrile --> rn:R02676

rn:R04366 { Linalool } <-- [Reduced NADPH---hemoprotein reductase] -- rn:R02708 <-- (S)-4-Hydroxymandelonitrile --> rn:R02676

rn:R06119 { d-Limonene } <-- [Reduced NADPH---hemoprotein reductase] -- rn:R02708 <-- (S)-4-Hydroxymandelonitrile --> rn:R02676

rn:R09451 { Hexadecanoic acid } <-- [Reduced NADPH---hemoprotein reductase] -- rn:R02708 <-- (S)-4-Hydroxymandelonitrile --> rn:R02676

rn:R09452 { (9Z)-Octadecenoic acid } <-- [Reduced NADPH---hemoprotein reductase] -- rn:R02708 <-- (S)-4-Hydroxymandelonitrile --> rn:R02676

rn:R09922 { (+)-Linalool } <-- [Reduced NADPH---hemoprotein reductase] -- rn:R02708 <-- (S)-4-Hydroxymandelonitrile --> rn:R02676

rn:R09923 { (-)-Linalool } <-- [Reduced NADPH---hemoprotein reductase] -- rn:R02708 <-- (S)-4-Hydroxymandelonitrile --> rn:R02676

rn:R09925 { Linalool } <-- [Reduced NADPH---hemoprotein reductase] -- rn:R02708 <-- (S)-4-Hydroxymandelonitrile --> rn:R02676

rn:R09934 { Humulene } <-- [Reduced NADPH---hemoprotein reductase] -- rn:R02708 <-- (S)-4-Hydroxymandelonitrile --> rn:R02676

rn:R10562 { (E,E)-Geranyllinalool } <-- [Reduced NADPH---hemoprotein reductase] -- rn:R02708 <-- (S)-4-Hydroxymandelonitrile --> rn:R02676

rn:R11055 { Myrcene } <-- [Reduced NADPH---hemoprotein reductase] -- rn:R02708 <-- (S)-4-Hydroxymandelonitrile --> rn:R02676

rn:R02468 { (-)-Limonene } <-- [Reduced NADPH---hemoprotein reductase] -- rn:R02708 <-- (S)-4-Hydroxymandelonitrile --> rn:R04296

rn:R02469 { (-)-Limonene } <-- [Reduced NADPH---hemoprotein reductase] -- rn:R02708 <-- (S)-4-Hydroxymandelonitrile --> rn:R04296

rn:R02470 { (-)-Limonene } <-- [Reduced NADPH---hemoprotein reductase] -- rn:R02708 <-- (S)-4-Hydroxymandelonitrile --> rn:R04296

rn:R04366 { Linalool } <-- [Reduced NADPH---hemoprotein reductase] -- rn:R02708 <-- (S)-4-Hydroxymandelonitrile --> rn:R04296

rn:R06119 { d-Limonene } <-- [Reduced NADPH---hemoprotein reductase] -- rn:R02708 <-- (S)-4-Hydroxymandelonitrile --> rn:R04296

rn:R09451 { Hexadecanoic acid } <-- [Reduced NADPH---hemoprotein reductase] -- rn:R02708 <-- (S)-4-Hydroxymandelonitrile --> rn:R04296

rn:R09452 { (9Z)-Octadecenoic acid } <-- [Reduced NADPH---hemoprotein reductase] -- rn:R02708 <-- (S)-4-Hydroxymandelonitrile --> rn:R04296

rn:R09922 { (+)-Linalool } <-- [Reduced NADPH---hemoprotein reductase] -- rn:R02708 <-- (S)-4-Hydroxymandelonitrile --> rn:R04296

rn:R09923 { (-)-Linalool } <-- [Reduced NADPH---hemoprotein reductase] -- rn:R02708 <-- (S)-4-Hydroxymandelonitrile --> rn:R04296

rn:R09925 { Linalool } <-- [Reduced NADPH---hemoprotein reductase] -- rn:R02708 <-- (S)-4-Hydroxymandelonitrile --> rn:R04296

rn:R09934 { Humulene } <-- [Reduced NADPH---hemoprotein reductase] -- rn:R02708 <-- (S)-4-Hydroxymandelonitrile --> rn:R04296

rn:R10562 { (E,E)-Geranyllinalool } <-- [Reduced NADPH---hemoprotein reductase] -- rn:R02708 <-- (S)-4-Hydroxymandelonitrile --> rn:R04296

rn:R11055 { Myrcene } <-- [Reduced NADPH---hemoprotein reductase] -- rn:R02708 <-- (S)-4-Hydroxymandelonitrile --> rn:R04296

rn:R02468 { (-)-Limonene } <-- [Reduced NADPH---hemoprotein reductase] -- rn:R02708 <-- [Oxidized NADPH---hemoprotein reductase] --> rn:R08551

rn:R02469 { (-)-Limonene } <-- [Reduced NADPH---hemoprotein reductase] -- rn:R02708 <-- [Oxidized NADPH---hemoprotein reductase] --> rn:R08551

rn:R02470 { (-)-Limonene } <-- [Reduced NADPH---hemoprotein reductase] -- rn:R02708 <-- [Oxidized NADPH---hemoprotein reductase] --> rn:R08551

rn:R04366 { Linalool } <-- [Reduced NADPH---hemoprotein reductase] -- rn:R02708 <-- [Oxidized NADPH---hemoprotein reductase] --> rn:R08551

rn:R06119 { d-Limonene } <-- [Reduced NADPH---hemoprotein reductase] -- rn:R02708 <-- [Oxidized NADPH---hemoprotein reductase] --> rn:R08551

rn:R09451 { Hexadecanoic acid } <-- [Reduced NADPH---hemoprotein reductase] -- rn:R02708 <-- [Oxidized NADPH---hemoprotein reductase] --> rn:R08551

rn:R09452 { (9Z)-Octadecenoic acid } <-- [Reduced NADPH---hemoprotein reductase] -- rn:R02708 <-- [Oxidized NADPH---hemoprotein reductase] --> rn:R08551

rn:R09922 { (+)-Linalool } <-- [Reduced NADPH---hemoprotein reductase] -- rn:R02708 <-- [Oxidized NADPH---hemoprotein reductase] --> rn:R08551

rn:R09923 { (-)-Linalool } <-- [Reduced NADPH---hemoprotein reductase] -- rn:R02708 <-- [Oxidized NADPH---hemoprotein reductase] --> rn:R08551

rn:R09925 { Linalool } <-- [Reduced NADPH---hemoprotein reductase] -- rn:R02708 <-- [Oxidized NADPH---hemoprotein reductase] --> rn:R08551

rn:R09934 { Humulene } <-- [Reduced NADPH---hemoprotein reductase] -- rn:R02708 <-- [Oxidized NADPH---hemoprotein reductase] --> rn:R08551

rn:R10562 { (E,E)-Geranyllinalool } <-- [Reduced NADPH---hemoprotein reductase] -- rn:R02708 <-- [Oxidized NADPH---hemoprotein reductase] --> rn:R08551

rn:R11055 { Myrcene } <-- [Reduced NADPH---hemoprotein reductase] -- rn:R02708 <-- [Oxidized NADPH---hemoprotein reductase] --> rn:R08551

rn:R02941 { Salicylaldehyde } <-- Salicylaldehyde -- rn:R02941 <-- Salicylate --> rn:R00818

rn:R05136 { Salicylaldehyde } <-- Salicylaldehyde -- rn:R02941 <-- Salicylate --> rn:R00818

rn:R02941 { Salicylaldehyde } <-- Salicylaldehyde -- rn:R02941 <-- Salicylate --> rn:R07709

rn:R05136 { Salicylaldehyde } <-- Salicylaldehyde -- rn:R02941 <-- Salicylate --> rn:R07709

rn:R02941 { Salicylaldehyde } <-- Salicylaldehyde -- rn:R02941 <-- Salicylate --> rn:R07710

rn:R05136 { Salicylaldehyde } <-- Salicylaldehyde -- rn:R02941 <-- Salicylate --> rn:R07710

rn:R02941 { Salicylaldehyde } <-- Salicylaldehyde -- rn:R02941 <-- Salicylate --> rn:R09539

rn:R05136 { Salicylaldehyde } <-- Salicylaldehyde -- rn:R02941 <-- Salicylate --> rn:R09539

rn:R02941 { Salicylaldehyde } <-- Salicylaldehyde -- rn:R02941 <-- Salicylate --> rn:R10448

rn:R05136 { Salicylaldehyde } <-- Salicylaldehyde -- rn:R02941 <-- Salicylate --> rn:R10448

rn:R08530 { (-)-Menthol } <-- Acetyl-CoA -- rn:R02955 <-- (3S)-Citramalyl-CoA --> rn:R00237

rn:R08531 { (+)-Neomenthol } <-- Acetyl-CoA -- rn:R02955 <-- (3S)-Citramalyl-CoA --> rn:R00237

rn:R08532 { (+)-Borneol } <-- Acetyl-CoA -- rn:R02955 <-- (3S)-Citramalyl-CoA --> rn:R00237

rn:R10474 { Cinnamyl alcohol } <-- Acetyl-CoA -- rn:R02955 <-- (3S)-Citramalyl-CoA --> rn:R00237

rn:R08530 { (-)-Menthol } <-- Acetyl-CoA -- rn:R02955 <-- Acetate --> rn:R01241

rn:R08531 { (+)-Neomenthol } <-- Acetyl-CoA -- rn:R02955 <-- Acetate --> rn:R01241

rn:R08532 { (+)-Borneol } <-- Acetyl-CoA -- rn:R02955 <-- Acetate --> rn:R01241

rn:R10474 { Cinnamyl alcohol } <-- Acetyl-CoA -- rn:R02955 <-- Acetate --> rn:R01241

rn:R08530 { (-)-Menthol } <-- Acetyl-CoA -- rn:R02955 <-- Acetate --> rn:R01308

rn:R08531 { (+)-Neomenthol } <-- Acetyl-CoA -- rn:R02955 <-- Acetate --> rn:R01308

rn:R08532 { (+)-Borneol } <-- Acetyl-CoA -- rn:R02955 <-- Acetate --> rn:R01308

rn:R10474 { Cinnamyl alcohol } <-- Acetyl-CoA -- rn:R02955 <-- Acetate --> rn:R01308

rn:R08530 { (-)-Menthol } <-- Acetyl-CoA -- rn:R02955 <-- Acetate --> rn:R01426

rn:R08531 { (+)-Neomenthol } <-- Acetyl-CoA -- rn:R02955 <-- Acetate --> rn:R01426

rn:R08532 { (+)-Borneol } <-- Acetyl-CoA -- rn:R02955 <-- Acetate --> rn:R01426

rn:R10474 { Cinnamyl alcohol } <-- Acetyl-CoA -- rn:R02955 <-- Acetate --> rn:R01426

rn:R08530 { (-)-Menthol } <-- Acetyl-CoA -- rn:R02955 <-- (3S)-Citramalyl-CoA --> rn:R02491

rn:R08531 { (+)-Neomenthol } <-- Acetyl-CoA -- rn:R02955 <-- (3S)-Citramalyl-CoA --> rn:R02491

rn:R08532 { (+)-Borneol } <-- Acetyl-CoA -- rn:R02955 <-- (3S)-Citramalyl-CoA --> rn:R02491

rn:R10474 { Cinnamyl alcohol } <-- Acetyl-CoA -- rn:R02955 <-- (3S)-Citramalyl-CoA --> rn:R02491

rn:R08530 { (-)-Menthol } <-- Acetyl-CoA -- rn:R02955 <-- Acetate --> rn:R05138

rn:R08531 { (+)-Neomenthol } <-- Acetyl-CoA -- rn:R02955 <-- Acetate --> rn:R05138

rn:R08532 { (+)-Borneol } <-- Acetyl-CoA -- rn:R02955 <-- Acetate --> rn:R05138

rn:R10474 { Cinnamyl alcohol } <-- Acetyl-CoA -- rn:R02955 <-- Acetate --> rn:R05138

rn:R08530 { (-)-Menthol } <-- Acetyl-CoA -- rn:R02955 <-- Acetate --> rn:R05219

rn:R08531 { (+)-Neomenthol } <-- Acetyl-CoA -- rn:R02955 <-- Acetate --> rn:R05219

rn:R08532 { (+)-Borneol } <-- Acetyl-CoA -- rn:R02955 <-- Acetate --> rn:R05219

rn:R10474 { Cinnamyl alcohol } <-- Acetyl-CoA -- rn:R02955 <-- Acetate --> rn:R05219

rn:R08530 { (-)-Menthol } <-- Acetyl-CoA -- rn:R02955 <-- Acetate --> rn:R09931

rn:R08531 { (+)-Neomenthol } <-- Acetyl-CoA -- rn:R02955 <-- Acetate --> rn:R09931

rn:R08532 { (+)-Borneol } <-- Acetyl-CoA -- rn:R02955 <-- Acetate --> rn:R09931

rn:R10474 { Cinnamyl alcohol } <-- Acetyl-CoA -- rn:R02955 <-- Acetate --> rn:R09931

rn:R05537 { 3-(2-Hydroxyphenyl)propanoate } <-- FAD -- rn:R02979 <-- FADH2 --> rn:R03978

rn:R05537 { 3-(2-Hydroxyphenyl)propanoate } <-- FAD -- rn:R02979 <-- FADH2 --> rn:R05488

rn:R05537 { 3-(2-Hydroxyphenyl)propanoate } <-- FAD -- rn:R02979 <-- FADH2 --> rn:R09517

rn:R05537 { 3-(2-Hydroxyphenyl)propanoate } <-- FAD -- rn:R02979 <-- FADH2 --> rn:R11653

rn:R05537 { 3-(2-Hydroxyphenyl)propanoate } <-- FAD -- rn:R02979 <-- FADH2 --> rn:R12021

rn:R05537 { 3-(2-Hydroxyphenyl)propanoate } <-- FAD -- rn:R02979 <-- FADH2 --> rn:R12023

rn:R05537 { 3-(2-Hydroxyphenyl)propanoate } <-- FAD -- rn:R02979 <-- FADH2 --> rn:R12027

rn:R05537 { 3-(2-Hydroxyphenyl)propanoate } <-- FAD -- rn:R02979 <-- FADH2 --> rn:R12030

rn:R08530 { (-)-Menthol } <-- Acetyl-CoA -- rn:R03153 <-- (3S)-Citramalyl-CoA --> rn:R00237

rn:R08531 { (+)-Neomenthol } <-- Acetyl-CoA -- rn:R03153 <-- (3S)-Citramalyl-CoA --> rn:R00237

rn:R08532 { (+)-Borneol } <-- Acetyl-CoA -- rn:R03153 <-- (3S)-Citramalyl-CoA --> rn:R00237

rn:R10474 { Cinnamyl alcohol } <-- Acetyl-CoA -- rn:R03153 <-- (3S)-Citramalyl-CoA --> rn:R00237

rn:R08530 { (-)-Menthol } <-- Acetyl-CoA -- rn:R03153 <-- Acetate --> rn:R01241

rn:R08531 { (+)-Neomenthol } <-- Acetyl-CoA -- rn:R03153 <-- Acetate --> rn:R01241

rn:R08532 { (+)-Borneol } <-- Acetyl-CoA -- rn:R03153 <-- Acetate --> rn:R01241

rn:R10474 { Cinnamyl alcohol } <-- Acetyl-CoA -- rn:R03153 <-- Acetate --> rn:R01241

rn:R08530 { (-)-Menthol } <-- Acetyl-CoA -- rn:R03153 <-- Acetate --> rn:R01308

rn:R08531 { (+)-Neomenthol } <-- Acetyl-CoA -- rn:R03153 <-- Acetate --> rn:R01308

rn:R08532 { (+)-Borneol } <-- Acetyl-CoA -- rn:R03153 <-- Acetate --> rn:R01308

rn:R10474 { Cinnamyl alcohol } <-- Acetyl-CoA -- rn:R03153 <-- Acetate --> rn:R01308

rn:R08530 { (-)-Menthol } <-- Acetyl-CoA -- rn:R03153 <-- Acetate --> rn:R01426

rn:R08531 { (+)-Neomenthol } <-- Acetyl-CoA -- rn:R03153 <-- Acetate --> rn:R01426

rn:R08532 { (+)-Borneol } <-- Acetyl-CoA -- rn:R03153 <-- Acetate --> rn:R01426

rn:R10474 { Cinnamyl alcohol } <-- Acetyl-CoA -- rn:R03153 <-- Acetate --> rn:R01426

rn:R08530 { (-)-Menthol } <-- Acetyl-CoA -- rn:R03153 <-- (3S)-Citramalyl-CoA --> rn:R02491

rn:R08531 { (+)-Neomenthol } <-- Acetyl-CoA -- rn:R03153 <-- (3S)-Citramalyl-CoA --> rn:R02491

rn:R08532 { (+)-Borneol } <-- Acetyl-CoA -- rn:R03153 <-- (3S)-Citramalyl-CoA --> rn:R02491

rn:R10474 { Cinnamyl alcohol } <-- Acetyl-CoA -- rn:R03153 <-- (3S)-Citramalyl-CoA --> rn:R02491

rn:R08530 { (-)-Menthol } <-- Acetyl-CoA -- rn:R03153 <-- Acetate --> rn:R05138

rn:R08531 { (+)-Neomenthol } <-- Acetyl-CoA -- rn:R03153 <-- Acetate --> rn:R05138

rn:R08532 { (+)-Borneol } <-- Acetyl-CoA -- rn:R03153 <-- Acetate --> rn:R05138

rn:R10474 { Cinnamyl alcohol } <-- Acetyl-CoA -- rn:R03153 <-- Acetate --> rn:R05138

rn:R08530 { (-)-Menthol } <-- Acetyl-CoA -- rn:R03153 <-- Acetate --> rn:R05219

rn:R08531 { (+)-Neomenthol } <-- Acetyl-CoA -- rn:R03153 <-- Acetate --> rn:R05219

rn:R08532 { (+)-Borneol } <-- Acetyl-CoA -- rn:R03153 <-- Acetate --> rn:R05219

rn:R10474 { Cinnamyl alcohol } <-- Acetyl-CoA -- rn:R03153 <-- Acetate --> rn:R05219

rn:R08530 { (-)-Menthol } <-- Acetyl-CoA -- rn:R03153 <-- Acetate --> rn:R09931

rn:R08531 { (+)-Neomenthol } <-- Acetyl-CoA -- rn:R03153 <-- Acetate --> rn:R09931

rn:R08532 { (+)-Borneol } <-- Acetyl-CoA -- rn:R03153 <-- Acetate --> rn:R09931

rn:R10474 { Cinnamyl alcohol } <-- Acetyl-CoA -- rn:R03153 <-- Acetate --> rn:R09931

rn:R09462 { (9Z)-Octadecenoic acid } <-- Lipid hydroperoxide -- rn:R03167 <-- Glutathione disulfide --> rn:R01111

rn:R09462 { (9Z)-Octadecenoic acid } <-- Lipid hydroperoxide -- rn:R03167 <-- Glutathione disulfide --> rn:R03915

rn:R09462 { (9Z)-Octadecenoic acid } <-- Lipid hydroperoxide -- rn:R03167 <-- Glutathione disulfide --> rn:R03984

rn:R09462 { (9Z)-Octadecenoic acid } <-- Lipid hydroperoxide -- rn:R03167 <-- Glutathione disulfide --> rn:R04039

rn:R09462 { (9Z)-Octadecenoic acid } <-- Lipid hydroperoxide -- rn:R03167 <-- Glutathione disulfide --> rn:R05267

rn:R09462 { (9Z)-Octadecenoic acid } <-- Lipid hydroperoxide -- rn:R03167 <-- Glutathione disulfide --> rn:R05717

rn:R02234 { Cyclohexanone } <-- Acceptor -- rn:R03172 <-- 2-Methylbut-2-enoyl-CoA --> rn:R04205

rn:R03212 { 3-Hydroxycyclohexanone } <-- Acceptor -- rn:R03172 <-- 2-Methylbut-2-enoyl-CoA --> rn:R04205

rn:R02234 { Cyclohexanone } <-- Acceptor -- rn:R03173 <-- 2-Methylbut-2-enoyl-CoA --> rn:R04205

rn:R03212 { 3-Hydroxycyclohexanone } <-- Acceptor -- rn:R03173 <-- 2-Methylbut-2-enoyl-CoA --> rn:R04205

rn:R02234 { Cyclohexanone } <-- Acceptor -- rn:R03185 <-- Dehydroascorbate --> rn:R01108

rn:R03212 { 3-Hydroxycyclohexanone } <-- Acceptor -- rn:R03185 <-- Dehydroascorbate --> rn:R01108

rn:R02234 { Cyclohexanone } <-- Acceptor -- rn:R03185 <-- Dehydroascorbate --> rn:R04785

rn:R03212 { 3-Hydroxycyclohexanone } <-- Acceptor -- rn:R03185 <-- Dehydroascorbate --> rn:R04785

rn:R02234 { Cyclohexanone } <-- Acceptor -- rn:R03185 <-- Dehydroascorbate --> rn:R08358

rn:R03212 { 3-Hydroxycyclohexanone } <-- Acceptor -- rn:R03185 <-- Dehydroascorbate --> rn:R08358

rn:R02234 { Cyclohexanone } <-- Acceptor -- rn:R03195 <-- Uroporphyrin III --> rn:R03950

rn:R03212 { 3-Hydroxycyclohexanone } <-- Acceptor -- rn:R03195 <-- Uroporphyrin III --> rn:R03950

rn:R01703 { Hexadecanoic acid } <-- Hydrogen peroxide -- rn:R03208 <-- Thyroxine --> rn:R03734

rn:R03369 { 3-(2-Hydroxyphenyl)propanoate } <-- 3-(2-Hydroxyphenyl)propanoate -- rn:R03369 <-- 3-(2,3-Dihydroxyphenyl)propanoate --> rn:R04376

rn:R03709 { 3-(2-Hydroxyphenyl)propanoate } <-- 3-(2-Hydroxyphenyl)propanoate -- rn:R03369 <-- 3-(2,3-Dihydroxyphenyl)propanoate --> rn:R04376

rn:R04899 { 3-(2-Hydroxyphenyl)propanoate } <-- 3-(2-Hydroxyphenyl)propanoate -- rn:R03369 <-- 3-(2,3-Dihydroxyphenyl)propanoate --> rn:R04376

rn:R05537 { 3-(2-Hydroxyphenyl)propanoate } <-- 3-(2-Hydroxyphenyl)propanoate -- rn:R03369 <-- 3-(2,3-Dihydroxyphenyl)propanoate --> rn:R04376

rn:R05136 { Salicylaldehyde } <-- Pyruvate -- rn:R03502 <-- L-Alanine --> rn:R04187

rn:R05136 { Salicylaldehyde } <-- Pyruvate -- rn:R03502 <-- L-Alanine --> rn:R08872

rn:R02179 { (-)-Menthol } <-- UDP-glucose -- rn:R03625 <-- Linamarin --> rn:R10040

rn:R03548 { 7,8-Dihydroxycoumarin } <-- UDP-glucose -- rn:R03625 <-- Linamarin --> rn:R10040

rn:R03369 { 3-(2-Hydroxyphenyl)propanoate } <-- 3-(2-Hydroxyphenyl)propanoate -- rn:R03709 <-- trans-2-Hydroxycinnamate --> rn:R03710

rn:R03709 { 3-(2-Hydroxyphenyl)propanoate } <-- 3-(2-Hydroxyphenyl)propanoate -- rn:R03709 <-- trans-2-Hydroxycinnamate --> rn:R03710

rn:R04899 { 3-(2-Hydroxyphenyl)propanoate } <-- 3-(2-Hydroxyphenyl)propanoate -- rn:R03709 <-- trans-2-Hydroxycinnamate --> rn:R03710

rn:R05537 { 3-(2-Hydroxyphenyl)propanoate } <-- 3-(2-Hydroxyphenyl)propanoate -- rn:R03709 <-- trans-2-Hydroxycinnamate --> rn:R03710

rn:R05537 { 3-(2-Hydroxyphenyl)propanoate } <-- FAD -- rn:R03777 <-- FADH2 --> rn:R03978

rn:R05537 { 3-(2-Hydroxyphenyl)propanoate } <-- FAD -- rn:R03777 <-- FADH2 --> rn:R05488

rn:R05537 { 3-(2-Hydroxyphenyl)propanoate } <-- FAD -- rn:R03777 <-- FADH2 --> rn:R09517

rn:R05537 { 3-(2-Hydroxyphenyl)propanoate } <-- FAD -- rn:R03777 <-- FADH2 --> rn:R11653

rn:R05537 { 3-(2-Hydroxyphenyl)propanoate } <-- FAD -- rn:R03777 <-- FADH2 --> rn:R12021

rn:R05537 { 3-(2-Hydroxyphenyl)propanoate } <-- FAD -- rn:R03777 <-- FADH2 --> rn:R12023

rn:R05537 { 3-(2-Hydroxyphenyl)propanoate } <-- FAD -- rn:R03777 <-- FADH2 --> rn:R12027

rn:R05537 { 3-(2-Hydroxyphenyl)propanoate } <-- FAD -- rn:R03777 <-- FADH2 --> rn:R12030

rn:R02234 { Cyclohexanone } <-- Acceptor -- rn:R03793 <-- alpha-Oxo-benzeneacetic acid --> rn:R01764

rn:R03212 { 3-Hydroxycyclohexanone } <-- Acceptor -- rn:R03793 <-- alpha-Oxo-benzeneacetic acid --> rn:R01764

rn:R02234 { Cyclohexanone } <-- Acceptor -- rn:R03793 <-- alpha-Oxo-benzeneacetic acid --> rn:R02450

rn:R03212 { 3-Hydroxycyclohexanone } <-- Acceptor -- rn:R03793 <-- alpha-Oxo-benzeneacetic acid --> rn:R02450

rn:R05537 { 3-(2-Hydroxyphenyl)propanoate } <-- FAD -- rn:R03857 <-- FADH2 --> rn:R03978

rn:R05537 { 3-(2-Hydroxyphenyl)propanoate } <-- FAD -- rn:R03857 <-- FADH2 --> rn:R05488

rn:R05537 { 3-(2-Hydroxyphenyl)propanoate } <-- FAD -- rn:R03857 <-- FADH2 --> rn:R09517

rn:R05537 { 3-(2-Hydroxyphenyl)propanoate } <-- FAD -- rn:R03857 <-- FADH2 --> rn:R11653

rn:R05537 { 3-(2-Hydroxyphenyl)propanoate } <-- FAD -- rn:R03857 <-- FADH2 --> rn:R12021

rn:R05537 { 3-(2-Hydroxyphenyl)propanoate } <-- FAD -- rn:R03857 <-- FADH2 --> rn:R12023

rn:R05537 { 3-(2-Hydroxyphenyl)propanoate } <-- FAD -- rn:R03857 <-- FADH2 --> rn:R12027

rn:R05537 { 3-(2-Hydroxyphenyl)propanoate } <-- FAD -- rn:R03857 <-- FADH2 --> rn:R12030

rn:R01703 { Hexadecanoic acid } <-- Hydrogen peroxide -- rn:R03953 <-- Triiodothyronine --> rn:R03952

rn:R05488 { Styrene } <-- FADH2 -- rn:R03978 <-- FAD --> rn:R02487

rn:R05488 { Styrene } <-- FADH2 -- rn:R03978 <-- FAD --> rn:R04095

rn:R05488 { Styrene } <-- FADH2 -- rn:R03978 <-- Lithocholic acid --> rn:R04139

rn:R05488 { Styrene } <-- FADH2 -- rn:R03978 <-- FAD --> rn:R05537

rn:R05488 { Styrene } <-- FADH2 -- rn:R03978 <-- FAD --> rn:R06943

rn:R05488 { Styrene } <-- FADH2 -- rn:R03978 <-- Lithocholic acid --> rn:R07203

rn:R05488 { Styrene } <-- FADH2 -- rn:R03978 <-- Lithocholic acid --> rn:R07206

rn:R05488 { Styrene } <-- FADH2 -- rn:R03978 <-- FAD --> rn:R07220

rn:R05488 { Styrene } <-- FADH2 -- rn:R03978 <-- FAD --> rn:R09520

rn:R05488 { Styrene } <-- FADH2 -- rn:R03978 <-- FAD --> rn:R11130

rn:R05488 { Styrene } <-- FADH2 -- rn:R03978 <-- Lithocholic acid --> rn:R11145

rn:R05537 { 3-(2-Hydroxyphenyl)propanoate } <-- FAD -- rn:R03990 <-- FADH2 --> rn:R03978

rn:R05537 { 3-(2-Hydroxyphenyl)propanoate } <-- FAD -- rn:R03990 <-- FADH2 --> rn:R05488

rn:R05537 { 3-(2-Hydroxyphenyl)propanoate } <-- FAD -- rn:R03990 <-- FADH2 --> rn:R09517

rn:R05537 { 3-(2-Hydroxyphenyl)propanoate } <-- FAD -- rn:R03990 <-- FADH2 --> rn:R11653

rn:R05537 { 3-(2-Hydroxyphenyl)propanoate } <-- FAD -- rn:R03990 <-- FADH2 --> rn:R12021

rn:R05537 { 3-(2-Hydroxyphenyl)propanoate } <-- FAD -- rn:R03990 <-- FADH2 --> rn:R12023

rn:R05537 { 3-(2-Hydroxyphenyl)propanoate } <-- FAD -- rn:R03990 <-- FADH2 --> rn:R12027

rn:R05537 { 3-(2-Hydroxyphenyl)propanoate } <-- FAD -- rn:R03990 <-- FADH2 --> rn:R12030

rn:R05537 { 3-(2-Hydroxyphenyl)propanoate } <-- FAD -- rn:R04092 <-- FADH2 --> rn:R03978

rn:R05537 { 3-(2-Hydroxyphenyl)propanoate } <-- FAD -- rn:R04092 <-- FADH2 --> rn:R05488

rn:R05537 { 3-(2-Hydroxyphenyl)propanoate } <-- FAD -- rn:R04092 <-- FADH2 --> rn:R09517

rn:R05537 { 3-(2-Hydroxyphenyl)propanoate } <-- FAD -- rn:R04092 <-- FADH2 --> rn:R11653

rn:R05537 { 3-(2-Hydroxyphenyl)propanoate } <-- FAD -- rn:R04092 <-- FADH2 --> rn:R12021

rn:R05537 { 3-(2-Hydroxyphenyl)propanoate } <-- FAD -- rn:R04092 <-- FADH2 --> rn:R12023

rn:R05537 { 3-(2-Hydroxyphenyl)propanoate } <-- FAD -- rn:R04092 <-- FADH2 --> rn:R12027

rn:R05537 { 3-(2-Hydroxyphenyl)propanoate } <-- FAD -- rn:R04092 <-- FADH2 --> rn:R12030

rn:R05537 { 3-(2-Hydroxyphenyl)propanoate } <-- FAD -- rn:R04095 <-- FADH2 --> rn:R03978

rn:R05537 { 3-(2-Hydroxyphenyl)propanoate } <-- FAD -- rn:R04095 <-- 3-Methylcrotonyl-CoA --> rn:R04138

rn:R05537 { 3-(2-Hydroxyphenyl)propanoate } <-- FAD -- rn:R04095 <-- FADH2 --> rn:R05488

rn:R05537 { 3-(2-Hydroxyphenyl)propanoate } <-- FAD -- rn:R04095 <-- FADH2 --> rn:R09517

rn:R05537 { 3-(2-Hydroxyphenyl)propanoate } <-- FAD -- rn:R04095 <-- FADH2 --> rn:R11653

rn:R05537 { 3-(2-Hydroxyphenyl)propanoate } <-- FAD -- rn:R04095 <-- FADH2 --> rn:R12021

rn:R05537 { 3-(2-Hydroxyphenyl)propanoate } <-- FAD -- rn:R04095 <-- FADH2 --> rn:R12023

rn:R05537 { 3-(2-Hydroxyphenyl)propanoate } <-- FAD -- rn:R04095 <-- FADH2 --> rn:R12027

rn:R05537 { 3-(2-Hydroxyphenyl)propanoate } <-- FAD -- rn:R04095 <-- FADH2 --> rn:R12030

rn:R02468 { (-)-Limonene } <-- [Reduced NADPH---hemoprotein reductase] -- rn:R04121 <-- [Oxidized NADPH---hemoprotein reductase] --> rn:R08551

rn:R02469 { (-)-Limonene } <-- [Reduced NADPH---hemoprotein reductase] -- rn:R04121 <-- [Oxidized NADPH---hemoprotein reductase] --> rn:R08551

rn:R02470 { (-)-Limonene } <-- [Reduced NADPH---hemoprotein reductase] -- rn:R04121 <-- [Oxidized NADPH---hemoprotein reductase] --> rn:R08551

rn:R04366 { Linalool } <-- [Reduced NADPH---hemoprotein reductase] -- rn:R04121 <-- [Oxidized NADPH---hemoprotein reductase] --> rn:R08551

rn:R06119 { d-Limonene } <-- [Reduced NADPH---hemoprotein reductase] -- rn:R04121 <-- [Oxidized NADPH---hemoprotein reductase] --> rn:R08551

rn:R09451 { Hexadecanoic acid } <-- [Reduced NADPH---hemoprotein reductase] -- rn:R04121 <-- [Oxidized NADPH---hemoprotein reductase] --> rn:R08551

rn:R09452 { (9Z)-Octadecenoic acid } <-- [Reduced NADPH---hemoprotein reductase] -- rn:R04121 <-- [Oxidized NADPH---hemoprotein reductase] --> rn:R08551

rn:R09922 { (+)-Linalool } <-- [Reduced NADPH---hemoprotein reductase] -- rn:R04121 <-- [Oxidized NADPH---hemoprotein reductase] --> rn:R08551

rn:R09923 { (-)-Linalool } <-- [Reduced NADPH---hemoprotein reductase] -- rn:R04121 <-- [Oxidized NADPH---hemoprotein reductase] --> rn:R08551

rn:R09925 { Linalool } <-- [Reduced NADPH---hemoprotein reductase] -- rn:R04121 <-- [Oxidized NADPH---hemoprotein reductase] --> rn:R08551

rn:R09934 { Humulene } <-- [Reduced NADPH---hemoprotein reductase] -- rn:R04121 <-- [Oxidized NADPH---hemoprotein reductase] --> rn:R08551

rn:R10562 { (E,E)-Geranyllinalool } <-- [Reduced NADPH---hemoprotein reductase] -- rn:R04121 <-- [Oxidized NADPH---hemoprotein reductase] --> rn:R08551

rn:R11055 { Myrcene } <-- [Reduced NADPH---hemoprotein reductase] -- rn:R04121 <-- [Oxidized NADPH---hemoprotein reductase] --> rn:R08551

rn:R02468 { (-)-Limonene } <-- [Reduced NADPH---hemoprotein reductase] -- rn:R04122 <-- [Oxidized NADPH---hemoprotein reductase] --> rn:R08551

rn:R02469 { (-)-Limonene } <-- [Reduced NADPH---hemoprotein reductase] -- rn:R04122 <-- [Oxidized NADPH---hemoprotein reductase] --> rn:R08551

rn:R02470 { (-)-Limonene } <-- [Reduced NADPH---hemoprotein reductase] -- rn:R04122 <-- [Oxidized NADPH---hemoprotein reductase] --> rn:R08551

rn:R04366 { Linalool } <-- [Reduced NADPH---hemoprotein reductase] -- rn:R04122 <-- [Oxidized NADPH---hemoprotein reductase] --> rn:R08551

rn:R06119 { d-Limonene } <-- [Reduced NADPH---hemoprotein reductase] -- rn:R04122 <-- [Oxidized NADPH---hemoprotein reductase] --> rn:R08551

rn:R09451 { Hexadecanoic acid } <-- [Reduced NADPH---hemoprotein reductase] -- rn:R04122 <-- [Oxidized NADPH---hemoprotein reductase] --> rn:R08551

rn:R09452 { (9Z)-Octadecenoic acid } <-- [Reduced NADPH---hemoprotein reductase] -- rn:R04122 <-- [Oxidized NADPH---hemoprotein reductase] --> rn:R08551

rn:R09922 { (+)-Linalool } <-- [Reduced NADPH---hemoprotein reductase] -- rn:R04122 <-- [Oxidized NADPH---hemoprotein reductase] --> rn:R08551

rn:R09923 { (-)-Linalool } <-- [Reduced NADPH---hemoprotein reductase] -- rn:R04122 <-- [Oxidized NADPH---hemoprotein reductase] --> rn:R08551

rn:R09925 { Linalool } <-- [Reduced NADPH---hemoprotein reductase] -- rn:R04122 <-- [Oxidized NADPH---hemoprotein reductase] --> rn:R08551

rn:R09934 { Humulene } <-- [Reduced NADPH---hemoprotein reductase] -- rn:R04122 <-- [Oxidized NADPH---hemoprotein reductase] --> rn:R08551

rn:R10562 { (E,E)-Geranyllinalool } <-- [Reduced NADPH---hemoprotein reductase] -- rn:R04122 <-- [Oxidized NADPH---hemoprotein reductase] --> rn:R08551

rn:R11055 { Myrcene } <-- [Reduced NADPH---hemoprotein reductase] -- rn:R04122 <-- [Oxidized NADPH---hemoprotein reductase] --> rn:R08551

rn:R05136 { Salicylaldehyde } <-- Pyruvate -- rn:R04152 <-- Phosphonoacetaldehyde --> rn:R00747

rn:R05136 { Salicylaldehyde } <-- Pyruvate -- rn:R04152 <-- L-Alanine --> rn:R04187

rn:R05136 { Salicylaldehyde } <-- Pyruvate -- rn:R04152 <-- Phosphonoacetaldehyde --> rn:R04251

rn:R05136 { Salicylaldehyde } <-- Pyruvate -- rn:R04152 <-- Phosphonoacetaldehyde --> rn:R08861

rn:R05136 { Salicylaldehyde } <-- Pyruvate -- rn:R04152 <-- L-Alanine --> rn:R08872

rn:R05136 { Salicylaldehyde } <-- Pyruvate -- rn:R04152 <-- Phosphonoacetaldehyde --> rn:R08883

rn:R05136 { Salicylaldehyde } <-- Pyruvate -- rn:R04152 <-- Phosphonoacetaldehyde --> rn:R08884

rn:R05136 { Salicylaldehyde } <-- Pyruvate -- rn:R04152 <-- Phosphonoacetaldehyde --> rn:R08885

rn:R05136 { Salicylaldehyde } <-- Pyruvate -- rn:R04152 <-- Phosphonoacetaldehyde --> rn:R08886

rn:R02234 { Cyclohexanone } <-- Acceptor -- rn:R04160 <-- 4-Hydroxyphenylglyoxylate --> rn:R02672

rn:R03212 { 3-Hydroxycyclohexanone } <-- Acceptor -- rn:R04160 <-- 4-Hydroxyphenylglyoxylate --> rn:R02672

rn:R02234 { Cyclohexanone } <-- Acceptor -- rn:R04160 <-- 4-Hydroxyphenylglyoxylate --> rn:R06626

rn:R03212 { 3-Hydroxycyclohexanone } <-- Acceptor -- rn:R04160 <-- 4-Hydroxyphenylglyoxylate --> rn:R06626

rn:R02179 { (-)-Menthol } <-- UDP-glucose -- rn:R04296 <-- Dhurrin --> rn:R10035

rn:R03548 { 7,8-Dihydroxycoumarin } <-- UDP-glucose -- rn:R04296 <-- Dhurrin --> rn:R10035

rn:R08447 { 4-Hydroxydihydrocinnamaldehyde } <-- Dopamine -- rn:R04300 <-- Hydrogen peroxide --> rn:R00069

rn:R08447 { 4-Hydroxydihydrocinnamaldehyde } <-- Dopamine -- rn:R04300 <-- Hydrogen peroxide --> rn:R00113

rn:R08447 { 4-Hydroxydihydrocinnamaldehyde } <-- Dopamine -- rn:R04300 <-- Hydrogen peroxide --> rn:R00274

rn:R08447 { 4-Hydroxydihydrocinnamaldehyde } <-- Dopamine -- rn:R04300 <-- Hydrogen peroxide --> rn:R00644

rn:R08447 { 4-Hydroxydihydrocinnamaldehyde } <-- Dopamine -- rn:R04300 <-- Hydrogen peroxide --> rn:R02657

rn:R08447 { 4-Hydroxydihydrocinnamaldehyde } <-- Dopamine -- rn:R04300 <-- Hydrogen peroxide --> rn:R03208

rn:R08447 { 4-Hydroxydihydrocinnamaldehyde } <-- Dopamine -- rn:R04300 <-- 3,4-Dihydroxyphenylacetaldehyde --> rn:R03300

rn:R08447 { 4-Hydroxydihydrocinnamaldehyde } <-- Dopamine -- rn:R04300 <-- 3,4-Dihydroxyphenylacetaldehyde --> rn:R03302

rn:R08447 { 4-Hydroxydihydrocinnamaldehyde } <-- Dopamine -- rn:R04300 <-- Hydrogen peroxide --> rn:R03953

rn:R08447 { 4-Hydroxydihydrocinnamaldehyde } <-- Dopamine -- rn:R04300 <-- 3,4-Dihydroxyphenylacetaldehyde --> rn:R04084

rn:R08447 { 4-Hydroxydihydrocinnamaldehyde } <-- Dopamine -- rn:R04300 <-- Hydrogen peroxide --> rn:R08863

rn:R08447 { 4-Hydroxydihydrocinnamaldehyde } <-- Dopamine -- rn:R04300 <-- Hydrogen peroxide --> rn:R11503

rn:R08447 { 4-Hydroxydihydrocinnamaldehyde } <-- Dopamine -- rn:R04300 <-- Hydrogen peroxide --> rn:R11522

rn:R08447 { 4-Hydroxydihydrocinnamaldehyde } <-- Dopamine -- rn:R04301 <-- 3-Methoxytyramine --> rn:R04890

rn:R08447 { 4-Hydroxydihydrocinnamaldehyde } <-- Dopamine -- rn:R04301 <-- 3-Methoxytyramine --> rn:R08831

rn:R02468 { (-)-Limonene } <-- [Reduced NADPH---hemoprotein reductase] -- rn:R04460 <-- N,N-Dihydroxy-L-tyrosine --> rn:R07190

rn:R02469 { (-)-Limonene } <-- [Reduced NADPH---hemoprotein reductase] -- rn:R04460 <-- N,N-Dihydroxy-L-tyrosine --> rn:R07190

rn:R02470 { (-)-Limonene } <-- [Reduced NADPH---hemoprotein reductase] -- rn:R04460 <-- N,N-Dihydroxy-L-tyrosine --> rn:R07190

rn:R04366 { Linalool } <-- [Reduced NADPH---hemoprotein reductase] -- rn:R04460 <-- N,N-Dihydroxy-L-tyrosine --> rn:R07190

rn:R06119 { d-Limonene } <-- [Reduced NADPH---hemoprotein reductase] -- rn:R04460 <-- N,N-Dihydroxy-L-tyrosine --> rn:R07190

rn:R09451 { Hexadecanoic acid } <-- [Reduced NADPH---hemoprotein reductase] -- rn:R04460 <-- N,N-Dihydroxy-L-tyrosine --> rn:R07190

rn:R09452 { (9Z)-Octadecenoic acid } <-- [Reduced NADPH---hemoprotein reductase] -- rn:R04460 <-- N,N-Dihydroxy-L-tyrosine --> rn:R07190

rn:R09922 { (+)-Linalool } <-- [Reduced NADPH---hemoprotein reductase] -- rn:R04460 <-- N,N-Dihydroxy-L-tyrosine --> rn:R07190

rn:R09923 { (-)-Linalool } <-- [Reduced NADPH---hemoprotein reductase] -- rn:R04460 <-- N,N-Dihydroxy-L-tyrosine --> rn:R07190

rn:R09925 { Linalool } <-- [Reduced NADPH---hemoprotein reductase] -- rn:R04460 <-- N,N-Dihydroxy-L-tyrosine --> rn:R07190

rn:R09934 { Humulene } <-- [Reduced NADPH---hemoprotein reductase] -- rn:R04460 <-- N,N-Dihydroxy-L-tyrosine --> rn:R07190

rn:R10562 { (E,E)-Geranyllinalool } <-- [Reduced NADPH---hemoprotein reductase] -- rn:R04460 <-- N,N-Dihydroxy-L-tyrosine --> rn:R07190

rn:R11055 { Myrcene } <-- [Reduced NADPH---hemoprotein reductase] -- rn:R04460 <-- N,N-Dihydroxy-L-tyrosine --> rn:R07190

rn:R02468 { (-)-Limonene } <-- [Reduced NADPH---hemoprotein reductase] -- rn:R04460 <-- [Oxidized NADPH---hemoprotein reductase] --> rn:R08551

rn:R02469 { (-)-Limonene } <-- [Reduced NADPH---hemoprotein reductase] -- rn:R04460 <-- [Oxidized NADPH---hemoprotein reductase] --> rn:R08551

rn:R02470 { (-)-Limonene } <-- [Reduced NADPH---hemoprotein reductase] -- rn:R04460 <-- [Oxidized NADPH---hemoprotein reductase] --> rn:R08551

rn:R04366 { Linalool } <-- [Reduced NADPH---hemoprotein reductase] -- rn:R04460 <-- [Oxidized NADPH---hemoprotein reductase] --> rn:R08551

rn:R06119 { d-Limonene } <-- [Reduced NADPH---hemoprotein reductase] -- rn:R04460 <-- [Oxidized NADPH---hemoprotein reductase] --> rn:R08551

rn:R09451 { Hexadecanoic acid } <-- [Reduced NADPH---hemoprotein reductase] -- rn:R04460 <-- [Oxidized NADPH---hemoprotein reductase] --> rn:R08551

rn:R09452 { (9Z)-Octadecenoic acid } <-- [Reduced NADPH---hemoprotein reductase] -- rn:R04460 <-- [Oxidized NADPH---hemoprotein reductase] --> rn:R08551

rn:R09922 { (+)-Linalool } <-- [Reduced NADPH---hemoprotein reductase] -- rn:R04460 <-- [Oxidized NADPH---hemoprotein reductase] --> rn:R08551

rn:R09923 { (-)-Linalool } <-- [Reduced NADPH---hemoprotein reductase] -- rn:R04460 <-- [Oxidized NADPH---hemoprotein reductase] --> rn:R08551

rn:R09925 { Linalool } <-- [Reduced NADPH---hemoprotein reductase] -- rn:R04460 <-- [Oxidized NADPH---hemoprotein reductase] --> rn:R08551

rn:R09934 { Humulene } <-- [Reduced NADPH---hemoprotein reductase] -- rn:R04460 <-- [Oxidized NADPH---hemoprotein reductase] --> rn:R08551

rn:R10562 { (E,E)-Geranyllinalool } <-- [Reduced NADPH---hemoprotein reductase] -- rn:R04460 <-- [Oxidized NADPH---hemoprotein reductase] --> rn:R08551

rn:R11055 { Myrcene } <-- [Reduced NADPH---hemoprotein reductase] -- rn:R04460 <-- [Oxidized NADPH---hemoprotein reductase] --> rn:R08551

rn:R05537 { 3-(2-Hydroxyphenyl)propanoate } <-- FAD -- rn:R04751 <-- FADH2 --> rn:R03978

rn:R05537 { 3-(2-Hydroxyphenyl)propanoate } <-- FAD -- rn:R04751 <-- FADH2 --> rn:R05488

rn:R05537 { 3-(2-Hydroxyphenyl)propanoate } <-- FAD -- rn:R04751 <-- trans-Hex-2-enoyl-CoA --> rn:R06985

rn:R05537 { 3-(2-Hydroxyphenyl)propanoate } <-- FAD -- rn:R04751 <-- FADH2 --> rn:R09517

rn:R05537 { 3-(2-Hydroxyphenyl)propanoate } <-- FAD -- rn:R04751 <-- FADH2 --> rn:R11653

rn:R05537 { 3-(2-Hydroxyphenyl)propanoate } <-- FAD -- rn:R04751 <-- FADH2 --> rn:R12021

rn:R05537 { 3-(2-Hydroxyphenyl)propanoate } <-- FAD -- rn:R04751 <-- FADH2 --> rn:R12023

rn:R05537 { 3-(2-Hydroxyphenyl)propanoate } <-- FAD -- rn:R04751 <-- FADH2 --> rn:R12027

rn:R05537 { 3-(2-Hydroxyphenyl)propanoate } <-- FAD -- rn:R04751 <-- FADH2 --> rn:R12030

rn:R05537 { 3-(2-Hydroxyphenyl)propanoate } <-- FAD -- rn:R04754 <-- FADH2 --> rn:R03978

rn:R05537 { 3-(2-Hydroxyphenyl)propanoate } <-- FAD -- rn:R04754 <-- FADH2 --> rn:R05488

rn:R05537 { 3-(2-Hydroxyphenyl)propanoate } <-- FAD -- rn:R04754 <-- FADH2 --> rn:R09517

rn:R05537 { 3-(2-Hydroxyphenyl)propanoate } <-- FAD -- rn:R04754 <-- FADH2 --> rn:R11653

rn:R05537 { 3-(2-Hydroxyphenyl)propanoate } <-- FAD -- rn:R04754 <-- FADH2 --> rn:R12021

rn:R05537 { 3-(2-Hydroxyphenyl)propanoate } <-- FAD -- rn:R04754 <-- FADH2 --> rn:R12023

rn:R05537 { 3-(2-Hydroxyphenyl)propanoate } <-- FAD -- rn:R04754 <-- FADH2 --> rn:R12027

rn:R05537 { 3-(2-Hydroxyphenyl)propanoate } <-- FAD -- rn:R04754 <-- FADH2 --> rn:R12030

rn:R06404 { alpha-Pinene } <-- e- -- rn:R04782 <-- Hydrazine --> rn:R00153

rn:R06404 { alpha-Pinene } <-- e- -- rn:R04782 <-- Hydrazine --> rn:R07174

rn:R06404 { alpha-Pinene } <-- e- -- rn:R04782 <-- Hydrazine --> rn:R11902

rn:R03369 { 3-(2-Hydroxyphenyl)propanoate } <-- 3-(2-Hydroxyphenyl)propanoate -- rn:R04899 <-- Phenylpropanoate --> rn:R02252

rn:R03709 { 3-(2-Hydroxyphenyl)propanoate } <-- 3-(2-Hydroxyphenyl)propanoate -- rn:R04899 <-- Phenylpropanoate --> rn:R02252

rn:R04899 { 3-(2-Hydroxyphenyl)propanoate } <-- 3-(2-Hydroxyphenyl)propanoate -- rn:R04899 <-- Phenylpropanoate --> rn:R02252

rn:R05537 { 3-(2-Hydroxyphenyl)propanoate } <-- 3-(2-Hydroxyphenyl)propanoate -- rn:R04899 <-- Phenylpropanoate --> rn:R02252

rn:R03369 { 3-(2-Hydroxyphenyl)propanoate } <-- 3-(2-Hydroxyphenyl)propanoate -- rn:R04899 <-- Phenylpropanoate --> rn:R06780

rn:R03709 { 3-(2-Hydroxyphenyl)propanoate } <-- 3-(2-Hydroxyphenyl)propanoate -- rn:R04899 <-- Phenylpropanoate --> rn:R06780

rn:R04899 { 3-(2-Hydroxyphenyl)propanoate } <-- 3-(2-Hydroxyphenyl)propanoate -- rn:R04899 <-- Phenylpropanoate --> rn:R06780

rn:R05537 { 3-(2-Hydroxyphenyl)propanoate } <-- 3-(2-Hydroxyphenyl)propanoate -- rn:R04899 <-- Phenylpropanoate --> rn:R06780

rn:R03369 { 3-(2-Hydroxyphenyl)propanoate } <-- 3-(2-Hydroxyphenyl)propanoate -- rn:R04899 <-- Phenylpropanoate --> rn:R06782

rn:R03709 { 3-(2-Hydroxyphenyl)propanoate } <-- 3-(2-Hydroxyphenyl)propanoate -- rn:R04899 <-- Phenylpropanoate --> rn:R06782

rn:R04899 { 3-(2-Hydroxyphenyl)propanoate } <-- 3-(2-Hydroxyphenyl)propanoate -- rn:R04899 <-- Phenylpropanoate --> rn:R06782

rn:R05537 { 3-(2-Hydroxyphenyl)propanoate } <-- 3-(2-Hydroxyphenyl)propanoate -- rn:R04899 <-- Phenylpropanoate --> rn:R06782

rn:R08530 { (-)-Menthol } <-- Acetyl-CoA -- rn:R04950 <-- S-Substituted N-acetyl-L-cysteine --> rn:R10553

rn:R08531 { (+)-Neomenthol } <-- Acetyl-CoA -- rn:R04950 <-- S-Substituted N-acetyl-L-cysteine --> rn:R10553

rn:R08532 { (+)-Borneol } <-- Acetyl-CoA -- rn:R04950 <-- S-Substituted N-acetyl-L-cysteine --> rn:R10553

rn:R10474 { Cinnamyl alcohol } <-- Acetyl-CoA -- rn:R04950 <-- S-Substituted N-acetyl-L-cysteine --> rn:R10553

rn:R02234 { Cyclohexanone } <-- Acceptor -- rn:R05040 <-- (3Z)-Phytochromobilin --> rn:R03678

rn:R03212 { 3-Hydroxycyclohexanone } <-- Acceptor -- rn:R05040 <-- (3Z)-Phytochromobilin --> rn:R03678

rn:R02781 { 2,4,6/3,5-Pentahydroxycyclohexanone } <-- L-Glutamine -- rn:R05224 <-- L-Glutamate --> rn:R00114

rn:R02781 { 2,4,6/3,5-Pentahydroxycyclohexanone } <-- L-Glutamine -- rn:R05224 <-- L-Glutamate --> rn:R00248

rn:R02781 { 2,4,6/3,5-Pentahydroxycyclohexanone } <-- L-Glutamine -- rn:R05224 <-- L-Glutamate --> rn:R00254

rn:R02781 { 2,4,6/3,5-Pentahydroxycyclohexanone } <-- L-Glutamine -- rn:R05224 <-- L-Glutamate --> rn:R00894

rn:R02781 { 2,4,6/3,5-Pentahydroxycyclohexanone } <-- L-Glutamine -- rn:R05224 <-- L-Glutamate --> rn:R02287

rn:R02781 { 2,4,6/3,5-Pentahydroxycyclohexanone } <-- L-Glutamine -- rn:R05224 <-- L-Glutamate --> rn:R03189

rn:R02781 { 2,4,6/3,5-Pentahydroxycyclohexanone } <-- L-Glutamine -- rn:R05224 <-- L-Glutamate --> rn:R03970

rn:R02781 { 2,4,6/3,5-Pentahydroxycyclohexanone } <-- L-Glutamine -- rn:R05224 <-- L-Glutamate --> rn:R03971

rn:R02781 { 2,4,6/3,5-Pentahydroxycyclohexanone } <-- L-Glutamine -- rn:R05224 <-- L-Glutamate --> rn:R04051

rn:R02781 { 2,4,6/3,5-Pentahydroxycyclohexanone } <-- L-Glutamine -- rn:R05224 <-- L-Glutamate --> rn:R04776

rn:R02781 { 2,4,6/3,5-Pentahydroxycyclohexanone } <-- L-Glutamine -- rn:R05224 <-- Hydrogenobyrinate a,c diamide --> rn:R05227

rn:R02781 { 2,4,6/3,5-Pentahydroxycyclohexanone } <-- L-Glutamine -- rn:R05224 <-- L-Glutamate --> rn:R07643

rn:R02781 { 2,4,6/3,5-Pentahydroxycyclohexanone } <-- L-Glutamine -- rn:R05225 <-- L-Glutamate --> rn:R00114

rn:R02781 { 2,4,6/3,5-Pentahydroxycyclohexanone } <-- L-Glutamine -- rn:R05225 <-- L-Glutamate --> rn:R00248

rn:R02781 { 2,4,6/3,5-Pentahydroxycyclohexanone } <-- L-Glutamine -- rn:R05225 <-- L-Glutamate --> rn:R00254

rn:R02781 { 2,4,6/3,5-Pentahydroxycyclohexanone } <-- L-Glutamine -- rn:R05225 <-- L-Glutamate --> rn:R00894

rn:R02781 { 2,4,6/3,5-Pentahydroxycyclohexanone } <-- L-Glutamine -- rn:R05225 <-- L-Glutamate --> rn:R02287

rn:R02781 { 2,4,6/3,5-Pentahydroxycyclohexanone } <-- L-Glutamine -- rn:R05225 <-- L-Glutamate --> rn:R03189

rn:R02781 { 2,4,6/3,5-Pentahydroxycyclohexanone } <-- L-Glutamine -- rn:R05225 <-- L-Glutamate --> rn:R03970

rn:R02781 { 2,4,6/3,5-Pentahydroxycyclohexanone } <-- L-Glutamine -- rn:R05225 <-- L-Glutamate --> rn:R03971

rn:R02781 { 2,4,6/3,5-Pentahydroxycyclohexanone } <-- L-Glutamine -- rn:R05225 <-- L-Glutamate --> rn:R04051

rn:R02781 { 2,4,6/3,5-Pentahydroxycyclohexanone } <-- L-Glutamine -- rn:R05225 <-- L-Glutamate --> rn:R04776

rn:R02781 { 2,4,6/3,5-Pentahydroxycyclohexanone } <-- L-Glutamine -- rn:R05225 <-- Adenosyl cobyrinate hexaamide --> rn:R05226

rn:R02781 { 2,4,6/3,5-Pentahydroxycyclohexanone } <-- L-Glutamine -- rn:R05225 <-- Adenosyl cobyrinate hexaamide --> rn:R06529

rn:R02781 { 2,4,6/3,5-Pentahydroxycyclohexanone } <-- L-Glutamine -- rn:R05225 <-- Adenosyl cobyrinate hexaamide --> rn:R07302

rn:R02781 { 2,4,6/3,5-Pentahydroxycyclohexanone } <-- L-Glutamine -- rn:R05225 <-- L-Glutamate --> rn:R07643

rn:R02468 { (-)-Limonene } <-- [Reduced NADPH---hemoprotein reductase] -- rn:R05259 <-- Sulfur --> rn:R00864

rn:R02469 { (-)-Limonene } <-- [Reduced NADPH---hemoprotein reductase] -- rn:R05259 <-- Sulfur --> rn:R00864

rn:R02470 { (-)-Limonene } <-- [Reduced NADPH---hemoprotein reductase] -- rn:R05259 <-- Sulfur --> rn:R00864

rn:R04366 { Linalool } <-- [Reduced NADPH---hemoprotein reductase] -- rn:R05259 <-- Sulfur --> rn:R00864

rn:R06119 { d-Limonene } <-- [Reduced NADPH---hemoprotein reductase] -- rn:R05259 <-- Sulfur --> rn:R00864

rn:R09451 { Hexadecanoic acid } <-- [Reduced NADPH---hemoprotein reductase] -- rn:R05259 <-- Sulfur --> rn:R00864

rn:R09452 { (9Z)-Octadecenoic acid } <-- [Reduced NADPH---hemoprotein reductase] -- rn:R05259 <-- Sulfur --> rn:R00864

rn:R09922 { (+)-Linalool } <-- [Reduced NADPH---hemoprotein reductase] -- rn:R05259 <-- Sulfur --> rn:R00864

rn:R09923 { (-)-Linalool } <-- [Reduced NADPH---hemoprotein reductase] -- rn:R05259 <-- Sulfur --> rn:R00864

rn:R09925 { Linalool } <-- [Reduced NADPH---hemoprotein reductase] -- rn:R05259 <-- Sulfur --> rn:R00864

rn:R09934 { Humulene } <-- [Reduced NADPH---hemoprotein reductase] -- rn:R05259 <-- Sulfur --> rn:R00864

rn:R10562 { (E,E)-Geranyllinalool } <-- [Reduced NADPH---hemoprotein reductase] -- rn:R05259 <-- Sulfur --> rn:R00864

rn:R11055 { Myrcene } <-- [Reduced NADPH---hemoprotein reductase] -- rn:R05259 <-- Sulfur --> rn:R00864

rn:R02468 { (-)-Limonene } <-- [Reduced NADPH---hemoprotein reductase] -- rn:R05259 <-- Sulfur --> rn:R03533

rn:R02469 { (-)-Limonene } <-- [Reduced NADPH---hemoprotein reductase] -- rn:R05259 <-- Sulfur --> rn:R03533

rn:R02470 { (-)-Limonene } <-- [Reduced NADPH---hemoprotein reductase] -- rn:R05259 <-- Sulfur --> rn:R03533

rn:R04366 { Linalool } <-- [Reduced NADPH---hemoprotein reductase] -- rn:R05259 <-- Sulfur --> rn:R03533

rn:R06119 { d-Limonene } <-- [Reduced NADPH---hemoprotein reductase] -- rn:R05259 <-- Sulfur --> rn:R03533

rn:R09451 { Hexadecanoic acid } <-- [Reduced NADPH---hemoprotein reductase] -- rn:R05259 <-- Sulfur --> rn:R03533

rn:R09452 { (9Z)-Octadecenoic acid } <-- [Reduced NADPH---hemoprotein reductase] -- rn:R05259 <-- Sulfur --> rn:R03533

rn:R09922 { (+)-Linalool } <-- [Reduced NADPH---hemoprotein reductase] -- rn:R05259 <-- Sulfur --> rn:R03533

rn:R09923 { (-)-Linalool } <-- [Reduced NADPH---hemoprotein reductase] -- rn:R05259 <-- Sulfur --> rn:R03533

rn:R09925 { Linalool } <-- [Reduced NADPH---hemoprotein reductase] -- rn:R05259 <-- Sulfur --> rn:R03533

rn:R09934 { Humulene } <-- [Reduced NADPH---hemoprotein reductase] -- rn:R05259 <-- Sulfur --> rn:R03533

rn:R10562 { (E,E)-Geranyllinalool } <-- [Reduced NADPH---hemoprotein reductase] -- rn:R05259 <-- Sulfur --> rn:R03533

rn:R11055 { Myrcene } <-- [Reduced NADPH---hemoprotein reductase] -- rn:R05259 <-- Sulfur --> rn:R03533

rn:R02468 { (-)-Limonene } <-- [Reduced NADPH---hemoprotein reductase] -- rn:R05259 <-- Sulfur --> rn:R07365

rn:R02469 { (-)-Limonene } <-- [Reduced NADPH---hemoprotein reductase] -- rn:R05259 <-- Sulfur --> rn:R07365

rn:R02470 { (-)-Limonene } <-- [Reduced NADPH---hemoprotein reductase] -- rn:R05259 <-- Sulfur --> rn:R07365

rn:R04366 { Linalool } <-- [Reduced NADPH---hemoprotein reductase] -- rn:R05259 <-- Sulfur --> rn:R07365

rn:R06119 { d-Limonene } <-- [Reduced NADPH---hemoprotein reductase] -- rn:R05259 <-- Sulfur --> rn:R07365

rn:R09451 { Hexadecanoic acid } <-- [Reduced NADPH---hemoprotein reductase] -- rn:R05259 <-- Sulfur --> rn:R07365

rn:R09452 { (9Z)-Octadecenoic acid } <-- [Reduced NADPH---hemoprotein reductase] -- rn:R05259 <-- Sulfur --> rn:R07365

rn:R09922 { (+)-Linalool } <-- [Reduced NADPH---hemoprotein reductase] -- rn:R05259 <-- Sulfur --> rn:R07365

rn:R09923 { (-)-Linalool } <-- [Reduced NADPH---hemoprotein reductase] -- rn:R05259 <-- Sulfur --> rn:R07365

rn:R09925 { Linalool } <-- [Reduced NADPH---hemoprotein reductase] -- rn:R05259 <-- Sulfur --> rn:R07365

rn:R09934 { Humulene } <-- [Reduced NADPH---hemoprotein reductase] -- rn:R05259 <-- Sulfur --> rn:R07365

rn:R10562 { (E,E)-Geranyllinalool } <-- [Reduced NADPH---hemoprotein reductase] -- rn:R05259 <-- Sulfur --> rn:R07365

rn:R11055 { Myrcene } <-- [Reduced NADPH---hemoprotein reductase] -- rn:R05259 <-- Sulfur --> rn:R07365

rn:R02468 { (-)-Limonene } <-- [Reduced NADPH---hemoprotein reductase] -- rn:R05259 <-- [Oxidized NADPH---hemoprotein reductase] --> rn:R08551

rn:R02469 { (-)-Limonene } <-- [Reduced NADPH---hemoprotein reductase] -- rn:R05259 <-- [Oxidized NADPH---hemoprotein reductase] --> rn:R08551

rn:R02470 { (-)-Limonene } <-- [Reduced NADPH---hemoprotein reductase] -- rn:R05259 <-- [Oxidized NADPH---hemoprotein reductase] --> rn:R08551

rn:R04366 { Linalool } <-- [Reduced NADPH---hemoprotein reductase] -- rn:R05259 <-- [Oxidized NADPH---hemoprotein reductase] --> rn:R08551

rn:R06119 { d-Limonene } <-- [Reduced NADPH---hemoprotein reductase] -- rn:R05259 <-- [Oxidized NADPH---hemoprotein reductase] --> rn:R08551

rn:R09451 { Hexadecanoic acid } <-- [Reduced NADPH---hemoprotein reductase] -- rn:R05259 <-- [Oxidized NADPH---hemoprotein reductase] --> rn:R08551

rn:R09452 { (9Z)-Octadecenoic acid } <-- [Reduced NADPH---hemoprotein reductase] -- rn:R05259 <-- [Oxidized NADPH---hemoprotein reductase] --> rn:R08551

rn:R09922 { (+)-Linalool } <-- [Reduced NADPH---hemoprotein reductase] -- rn:R05259 <-- [Oxidized NADPH---hemoprotein reductase] --> rn:R08551

rn:R09923 { (-)-Linalool } <-- [Reduced NADPH---hemoprotein reductase] -- rn:R05259 <-- [Oxidized NADPH---hemoprotein reductase] --> rn:R08551

rn:R09925 { Linalool } <-- [Reduced NADPH---hemoprotein reductase] -- rn:R05259 <-- [Oxidized NADPH---hemoprotein reductase] --> rn:R08551

rn:R09934 { Humulene } <-- [Reduced NADPH---hemoprotein reductase] -- rn:R05259 <-- [Oxidized NADPH---hemoprotein reductase] --> rn:R08551

rn:R10562 { (E,E)-Geranyllinalool } <-- [Reduced NADPH---hemoprotein reductase] -- rn:R05259 <-- [Oxidized NADPH---hemoprotein reductase] --> rn:R08551

rn:R11055 { Myrcene } <-- [Reduced NADPH---hemoprotein reductase] -- rn:R05259 <-- [Oxidized NADPH---hemoprotein reductase] --> rn:R08551

rn:R05001 { 2-Hydroxyphenylacetate } <-- 2-Hydroxyphenylacetate -- rn:R05450 <-- Homogentisate --> rn:R02518

rn:R05450 { 2-Hydroxyphenylacetate } <-- 2-Hydroxyphenylacetate -- rn:R05450 <-- Homogentisate --> rn:R02518

rn:R05001 { 2-Hydroxyphenylacetate } <-- 2-Hydroxyphenylacetate -- rn:R05450 <-- Homogentisate --> rn:R02519

rn:R05450 { 2-Hydroxyphenylacetate } <-- 2-Hydroxyphenylacetate -- rn:R05450 <-- Homogentisate --> rn:R02519

rn:R05001 { 2-Hydroxyphenylacetate } <-- 2-Hydroxyphenylacetate -- rn:R05450 <-- Homogentisate --> rn:R02520

rn:R05450 { 2-Hydroxyphenylacetate } <-- 2-Hydroxyphenylacetate -- rn:R05450 <-- Homogentisate --> rn:R02520

rn:R05001 { 2-Hydroxyphenylacetate } <-- 2-Hydroxyphenylacetate -- rn:R05450 <-- Homogentisate --> rn:R07500

rn:R05450 { 2-Hydroxyphenylacetate } <-- 2-Hydroxyphenylacetate -- rn:R05450 <-- Homogentisate --> rn:R07500

rn:R05001 { 2-Hydroxyphenylacetate } <-- 2-Hydroxyphenylacetate -- rn:R05450 <-- Homogentisate --> rn:R08782

rn:R05450 { 2-Hydroxyphenylacetate } <-- 2-Hydroxyphenylacetate -- rn:R05450 <-- Homogentisate --> rn:R08782

rn:R05001 { 2-Hydroxyphenylacetate } <-- 2-Hydroxyphenylacetate -- rn:R05450 <-- Homogentisate --> rn:R10708

rn:R05450 { 2-Hydroxyphenylacetate } <-- 2-Hydroxyphenylacetate -- rn:R05450 <-- Homogentisate --> rn:R10708

rn:R02468 { (-)-Limonene } <-- [Reduced NADPH---hemoprotein reductase] -- rn:R05487 <-- 2-Hydroxyphenylacetate --> rn:R05001

rn:R02469 { (-)-Limonene } <-- [Reduced NADPH---hemoprotein reductase] -- rn:R05487 <-- 2-Hydroxyphenylacetate --> rn:R05001

rn:R02470 { (-)-Limonene } <-- [Reduced NADPH---hemoprotein reductase] -- rn:R05487 <-- 2-Hydroxyphenylacetate --> rn:R05001

rn:R04366 { Linalool } <-- [Reduced NADPH---hemoprotein reductase] -- rn:R05487 <-- 2-Hydroxyphenylacetate --> rn:R05001

rn:R06119 { d-Limonene } <-- [Reduced NADPH---hemoprotein reductase] -- rn:R05487 <-- 2-Hydroxyphenylacetate --> rn:R05001

rn:R09451 { Hexadecanoic acid } <-- [Reduced NADPH---hemoprotein reductase] -- rn:R05487 <-- 2-Hydroxyphenylacetate --> rn:R05001

rn:R09452 { (9Z)-Octadecenoic acid } <-- [Reduced NADPH---hemoprotein reductase] -- rn:R05487 <-- 2-Hydroxyphenylacetate --> rn:R05001

rn:R09922 { (+)-Linalool } <-- [Reduced NADPH---hemoprotein reductase] -- rn:R05487 <-- 2-Hydroxyphenylacetate --> rn:R05001

rn:R09923 { (-)-Linalool } <-- [Reduced NADPH---hemoprotein reductase] -- rn:R05487 <-- 2-Hydroxyphenylacetate --> rn:R05001

rn:R09925 { Linalool } <-- [Reduced NADPH---hemoprotein reductase] -- rn:R05487 <-- 2-Hydroxyphenylacetate --> rn:R05001

rn:R09934 { Humulene } <-- [Reduced NADPH---hemoprotein reductase] -- rn:R05487 <-- 2-Hydroxyphenylacetate --> rn:R05001

rn:R10562 { (E,E)-Geranyllinalool } <-- [Reduced NADPH---hemoprotein reductase] -- rn:R05487 <-- 2-Hydroxyphenylacetate --> rn:R05001

rn:R11055 { Myrcene } <-- [Reduced NADPH---hemoprotein reductase] -- rn:R05487 <-- 2-Hydroxyphenylacetate --> rn:R05001

rn:R02468 { (-)-Limonene } <-- [Reduced NADPH---hemoprotein reductase] -- rn:R05487 <-- 2-Hydroxyphenylacetate --> rn:R05450

rn:R02469 { (-)-Limonene } <-- [Reduced NADPH---hemoprotein reductase] -- rn:R05487 <-- 2-Hydroxyphenylacetate --> rn:R05450

rn:R02470 { (-)-Limonene } <-- [Reduced NADPH---hemoprotein reductase] -- rn:R05487 <-- 2-Hydroxyphenylacetate --> rn:R05450

rn:R04366 { Linalool } <-- [Reduced NADPH---hemoprotein reductase] -- rn:R05487 <-- 2-Hydroxyphenylacetate --> rn:R05450

rn:R06119 { d-Limonene } <-- [Reduced NADPH---hemoprotein reductase] -- rn:R05487 <-- 2-Hydroxyphenylacetate --> rn:R05450

rn:R09451 { Hexadecanoic acid } <-- [Reduced NADPH---hemoprotein reductase] -- rn:R05487 <-- 2-Hydroxyphenylacetate --> rn:R05450

rn:R09452 { (9Z)-Octadecenoic acid } <-- [Reduced NADPH---hemoprotein reductase] -- rn:R05487 <-- 2-Hydroxyphenylacetate --> rn:R05450

rn:R09922 { (+)-Linalool } <-- [Reduced NADPH---hemoprotein reductase] -- rn:R05487 <-- 2-Hydroxyphenylacetate --> rn:R05450

rn:R09923 { (-)-Linalool } <-- [Reduced NADPH---hemoprotein reductase] -- rn:R05487 <-- 2-Hydroxyphenylacetate --> rn:R05450

rn:R09925 { Linalool } <-- [Reduced NADPH---hemoprotein reductase] -- rn:R05487 <-- 2-Hydroxyphenylacetate --> rn:R05450

rn:R09934 { Humulene } <-- [Reduced NADPH---hemoprotein reductase] -- rn:R05487 <-- 2-Hydroxyphenylacetate --> rn:R05450

rn:R10562 { (E,E)-Geranyllinalool } <-- [Reduced NADPH---hemoprotein reductase] -- rn:R05487 <-- 2-Hydroxyphenylacetate --> rn:R05450

rn:R11055 { Myrcene } <-- [Reduced NADPH---hemoprotein reductase] -- rn:R05487 <-- 2-Hydroxyphenylacetate --> rn:R05450

rn:R02468 { (-)-Limonene } <-- [Reduced NADPH---hemoprotein reductase] -- rn:R05487 <-- [Oxidized NADPH---hemoprotein reductase] --> rn:R08551

rn:R02469 { (-)-Limonene } <-- [Reduced NADPH---hemoprotein reductase] -- rn:R05487 <-- [Oxidized NADPH---hemoprotein reductase] --> rn:R08551

rn:R02470 { (-)-Limonene } <-- [Reduced NADPH---hemoprotein reductase] -- rn:R05487 <-- [Oxidized NADPH---hemoprotein reductase] --> rn:R08551

rn:R04366 { Linalool } <-- [Reduced NADPH---hemoprotein reductase] -- rn:R05487 <-- [Oxidized NADPH---hemoprotein reductase] --> rn:R08551

rn:R06119 { d-Limonene } <-- [Reduced NADPH---hemoprotein reductase] -- rn:R05487 <-- [Oxidized NADPH---hemoprotein reductase] --> rn:R08551

rn:R09451 { Hexadecanoic acid } <-- [Reduced NADPH---hemoprotein reductase] -- rn:R05487 <-- [Oxidized NADPH---hemoprotein reductase] --> rn:R08551

rn:R09452 { (9Z)-Octadecenoic acid } <-- [Reduced NADPH---hemoprotein reductase] -- rn:R05487 <-- [Oxidized NADPH---hemoprotein reductase] --> rn:R08551

rn:R09922 { (+)-Linalool } <-- [Reduced NADPH---hemoprotein reductase] -- rn:R05487 <-- [Oxidized NADPH---hemoprotein reductase] --> rn:R08551

rn:R09923 { (-)-Linalool } <-- [Reduced NADPH---hemoprotein reductase] -- rn:R05487 <-- [Oxidized NADPH---hemoprotein reductase] --> rn:R08551

rn:R09925 { Linalool } <-- [Reduced NADPH---hemoprotein reductase] -- rn:R05487 <-- [Oxidized NADPH---hemoprotein reductase] --> rn:R08551

rn:R09934 { Humulene } <-- [Reduced NADPH---hemoprotein reductase] -- rn:R05487 <-- [Oxidized NADPH---hemoprotein reductase] --> rn:R08551

rn:R10562 { (E,E)-Geranyllinalool } <-- [Reduced NADPH---hemoprotein reductase] -- rn:R05487 <-- [Oxidized NADPH---hemoprotein reductase] --> rn:R08551

rn:R11055 { Myrcene } <-- [Reduced NADPH---hemoprotein reductase] -- rn:R05487 <-- [Oxidized NADPH---hemoprotein reductase] --> rn:R08551

rn:R03369 { 3-(2-Hydroxyphenyl)propanoate } <-- 3-(2-Hydroxyphenyl)propanoate -- rn:R05537 <-- Acetyl-CoA --> rn:R00693

rn:R03709 { 3-(2-Hydroxyphenyl)propanoate } <-- 3-(2-Hydroxyphenyl)propanoate -- rn:R05537 <-- Acetyl-CoA --> rn:R00693

rn:R04899 { 3-(2-Hydroxyphenyl)propanoate } <-- 3-(2-Hydroxyphenyl)propanoate -- rn:R05537 <-- Acetyl-CoA --> rn:R00693

rn:R05537 { 3-(2-Hydroxyphenyl)propanoate } <-- 3-(2-Hydroxyphenyl)propanoate -- rn:R05537 <-- Acetyl-CoA --> rn:R00693 # rn:R05537 { 3-(2-Hydroxyphenyl)propanoate } <-- FAD -- rn:R05537 <-- Acetyl-CoA --> rn:R00693

rn:R03369 { 3-(2-Hydroxyphenyl)propanoate } <-- 3-(2-Hydroxyphenyl)propanoate -- rn:R05537 <-- Salicylate --> rn:R00818

rn:R03709 { 3-(2-Hydroxyphenyl)propanoate } <-- 3-(2-Hydroxyphenyl)propanoate -- rn:R05537 <-- Salicylate --> rn:R00818

rn:R04899 { 3-(2-Hydroxyphenyl)propanoate } <-- 3-(2-Hydroxyphenyl)propanoate -- rn:R05537 <-- Salicylate --> rn:R00818

rn:R05537 { 3-(2-Hydroxyphenyl)propanoate } <-- 3-(2-Hydroxyphenyl)propanoate -- rn:R05537 <-- Salicylate --> rn:R00818 # rn:R05537 { 3-(2-Hydroxyphenyl)propanoate } <-- FAD -- rn:R05537 <-- Salicylate --> rn:R00818

rn:R03369 { 3-(2-Hydroxyphenyl)propanoate } <-- 3-(2-Hydroxyphenyl)propanoate -- rn:R05537 <-- AMP --> rn:R01490

rn:R03709 { 3-(2-Hydroxyphenyl)propanoate } <-- 3-(2-Hydroxyphenyl)propanoate -- rn:R05537 <-- AMP --> rn:R01490

rn:R04899 { 3-(2-Hydroxyphenyl)propanoate } <-- 3-(2-Hydroxyphenyl)propanoate -- rn:R05537 <-- AMP --> rn:R01490

rn:R05537 { 3-(2-Hydroxyphenyl)propanoate } <-- 3-(2-Hydroxyphenyl)propanoate -- rn:R05537 <-- AMP --> rn:R01490 # rn:R05537 { 3-(2-Hydroxyphenyl)propanoate } <-- FAD -- rn:R05537 <-- AMP --> rn:R01490

rn:R03369 { 3-(2-Hydroxyphenyl)propanoate } <-- 3-(2-Hydroxyphenyl)propanoate -- rn:R05537 <-- Acetyl-CoA --> rn:R02152

rn:R03709 { 3-(2-Hydroxyphenyl)propanoate } <-- 3-(2-Hydroxyphenyl)propanoate -- rn:R05537 <-- Acetyl-CoA --> rn:R02152

rn:R04899 { 3-(2-Hydroxyphenyl)propanoate } <-- 3-(2-Hydroxyphenyl)propanoate -- rn:R05537 <-- Acetyl-CoA --> rn:R02152

rn:R05537 { 3-(2-Hydroxyphenyl)propanoate } <-- 3-(2-Hydroxyphenyl)propanoate -- rn:R05537 <-- Acetyl-CoA --> rn:R02152 # rn:R05537 { 3-(2-Hydroxyphenyl)propanoate } <-- FAD -- rn:R05537 <-- Acetyl-CoA --> rn:R02152

rn:R03369 { 3-(2-Hydroxyphenyl)propanoate } <-- 3-(2-Hydroxyphenyl)propanoate -- rn:R05537 <-- Acetyl-CoA --> rn:R02911

rn:R03709 { 3-(2-Hydroxyphenyl)propanoate } <-- 3-(2-Hydroxyphenyl)propanoate -- rn:R05537 <-- Acetyl-CoA --> rn:R02911

rn:R04899 { 3-(2-Hydroxyphenyl)propanoate } <-- 3-(2-Hydroxyphenyl)propanoate -- rn:R05537 <-- Acetyl-CoA --> rn:R02911

rn:R05537 { 3-(2-Hydroxyphenyl)propanoate } <-- 3-(2-Hydroxyphenyl)propanoate -- rn:R05537 <-- Acetyl-CoA --> rn:R02911 # rn:R05537 { 3-(2-Hydroxyphenyl)propanoate } <-- FAD -- rn:R05537 <-- Acetyl-CoA --> rn:R02911

rn:R03369 { 3-(2-Hydroxyphenyl)propanoate } <-- 3-(2-Hydroxyphenyl)propanoate -- rn:R05537 <-- Acetyl-CoA --> rn:R02955

rn:R03709 { 3-(2-Hydroxyphenyl)propanoate } <-- 3-(2-Hydroxyphenyl)propanoate -- rn:R05537 <-- Acetyl-CoA --> rn:R02955

rn:R04899 { 3-(2-Hydroxyphenyl)propanoate } <-- 3-(2-Hydroxyphenyl)propanoate -- rn:R05537 <-- Acetyl-CoA --> rn:R02955

rn:R05537 { 3-(2-Hydroxyphenyl)propanoate } <-- 3-(2-Hydroxyphenyl)propanoate -- rn:R05537 <-- Acetyl-CoA --> rn:R02955 # rn:R05537 { 3-(2-Hydroxyphenyl)propanoate } <-- FAD -- rn:R05537 <-- Acetyl-CoA --> rn:R02955

rn:R03369 { 3-(2-Hydroxyphenyl)propanoate } <-- 3-(2-Hydroxyphenyl)propanoate -- rn:R05537 <-- Acetyl-CoA --> rn:R03153

rn:R03709 { 3-(2-Hydroxyphenyl)propanoate } <-- 3-(2-Hydroxyphenyl)propanoate -- rn:R05537 <-- Acetyl-CoA --> rn:R03153

rn:R04899 { 3-(2-Hydroxyphenyl)propanoate } <-- 3-(2-Hydroxyphenyl)propanoate -- rn:R05537 <-- Acetyl-CoA --> rn:R03153

rn:R05537 { 3-(2-Hydroxyphenyl)propanoate } <-- 3-(2-Hydroxyphenyl)propanoate -- rn:R05537 <-- Acetyl-CoA --> rn:R03153 # rn:R05537 { 3-(2-Hydroxyphenyl)propanoate } <-- FAD -- rn:R05537 <-- Acetyl-CoA --> rn:R03153

rn:R03369 { 3-(2-Hydroxyphenyl)propanoate } <-- 3-(2-Hydroxyphenyl)propanoate -- rn:R05537 <-- Acetyl-CoA --> rn:R03903

rn:R03709 { 3-(2-Hydroxyphenyl)propanoate } <-- 3-(2-Hydroxyphenyl)propanoate -- rn:R05537 <-- Acetyl-CoA --> rn:R03903

rn:R04899 { 3-(2-Hydroxyphenyl)propanoate } <-- 3-(2-Hydroxyphenyl)propanoate -- rn:R05537 <-- Acetyl-CoA --> rn:R03903

rn:R05537 { 3-(2-Hydroxyphenyl)propanoate } <-- 3-(2-Hydroxyphenyl)propanoate -- rn:R05537 <-- Acetyl-CoA --> rn:R03903 # rn:R05537 { 3-(2-Hydroxyphenyl)propanoate } <-- FAD -- rn:R05537 <-- Acetyl-CoA --> rn:R03903

rn:R03369 { 3-(2-Hydroxyphenyl)propanoate } <-- 3-(2-Hydroxyphenyl)propanoate -- rn:R05537 <-- FADH2 --> rn:R03978

rn:R03709 { 3-(2-Hydroxyphenyl)propanoate } <-- 3-(2-Hydroxyphenyl)propanoate -- rn:R05537 <-- FADH2 --> rn:R03978

rn:R04899 { 3-(2-Hydroxyphenyl)propanoate } <-- 3-(2-Hydroxyphenyl)propanoate -- rn:R05537 <-- FADH2 --> rn:R03978

rn:R05537 { 3-(2-Hydroxyphenyl)propanoate } <-- 3-(2-Hydroxyphenyl)propanoate -- rn:R05537 <-- FADH2 --> rn:R03978 # rn:R05537 { 3-(2-Hydroxyphenyl)propanoate } <-- FAD -- rn:R05537 <-- FADH2 --> rn:R03978

rn:R03369 { 3-(2-Hydroxyphenyl)propanoate } <-- 3-(2-Hydroxyphenyl)propanoate -- rn:R05537 <-- Acetyl-CoA --> rn:R04950

rn:R03709 { 3-(2-Hydroxyphenyl)propanoate } <-- 3-(2-Hydroxyphenyl)propanoate -- rn:R05537 <-- Acetyl-CoA --> rn:R04950

rn:R04899 { 3-(2-Hydroxyphenyl)propanoate } <-- 3-(2-Hydroxyphenyl)propanoate -- rn:R05537 <-- Acetyl-CoA --> rn:R04950

rn:R05537 { 3-(2-Hydroxyphenyl)propanoate } <-- 3-(2-Hydroxyphenyl)propanoate -- rn:R05537 <-- Acetyl-CoA --> rn:R04950 # rn:R05537 { 3-(2-Hydroxyphenyl)propanoate } <-- FAD -- rn:R05537 <-- Acetyl-CoA --> rn:R04950

rn:R03369 { 3-(2-Hydroxyphenyl)propanoate } <-- 3-(2-Hydroxyphenyl)propanoate -- rn:R05537 <-- FADH2 --> rn:R05488

rn:R03709 { 3-(2-Hydroxyphenyl)propanoate } <-- 3-(2-Hydroxyphenyl)propanoate -- rn:R05537 <-- FADH2 --> rn:R05488

rn:R04899 { 3-(2-Hydroxyphenyl)propanoate } <-- 3-(2-Hydroxyphenyl)propanoate -- rn:R05537 <-- FADH2 --> rn:R05488

rn:R05537 { 3-(2-Hydroxyphenyl)propanoate } <-- 3-(2-Hydroxyphenyl)propanoate -- rn:R05537 <-- FADH2 --> rn:R05488 # rn:R05537 { 3-(2-Hydroxyphenyl)propanoate } <-- FAD -- rn:R05537 <-- FADH2 --> rn:R05488

rn:R03369 { 3-(2-Hydroxyphenyl)propanoate } <-- 3-(2-Hydroxyphenyl)propanoate -- rn:R05537 <-- Acetyl-CoA --> rn:R05509

rn:R03709 { 3-(2-Hydroxyphenyl)propanoate } <-- 3-(2-Hydroxyphenyl)propanoate -- rn:R05537 <-- Acetyl-CoA --> rn:R05509

rn:R04899 { 3-(2-Hydroxyphenyl)propanoate } <-- 3-(2-Hydroxyphenyl)propanoate -- rn:R05537 <-- Acetyl-CoA --> rn:R05509

rn:R05537 { 3-(2-Hydroxyphenyl)propanoate } <-- 3-(2-Hydroxyphenyl)propanoate -- rn:R05537 <-- Acetyl-CoA --> rn:R05509 # rn:R05537 { 3-(2-Hydroxyphenyl)propanoate } <-- FAD -- rn:R05537 <-- Acetyl-CoA --> rn:R05509

rn:R03369 { 3-(2-Hydroxyphenyl)propanoate } <-- 3-(2-Hydroxyphenyl)propanoate -- rn:R05537 <-- AMP --> rn:R05717

rn:R03709 { 3-(2-Hydroxyphenyl)propanoate } <-- 3-(2-Hydroxyphenyl)propanoate -- rn:R05537 <-- AMP --> rn:R05717

rn:R04899 { 3-(2-Hydroxyphenyl)propanoate } <-- 3-(2-Hydroxyphenyl)propanoate -- rn:R05537 <-- AMP --> rn:R05717

rn:R05537 { 3-(2-Hydroxyphenyl)propanoate } <-- 3-(2-Hydroxyphenyl)propanoate -- rn:R05537 <-- AMP --> rn:R05717 # rn:R05537 { 3-(2-Hydroxyphenyl)propanoate } <-- FAD -- rn:R05537 <-- AMP --> rn:R05717

rn:R03369 { 3-(2-Hydroxyphenyl)propanoate } <-- 3-(2-Hydroxyphenyl)propanoate -- rn:R05537 <-- Acetyl-CoA --> rn:R07253

rn:R03709 { 3-(2-Hydroxyphenyl)propanoate } <-- 3-(2-Hydroxyphenyl)propanoate -- rn:R05537 <-- Acetyl-CoA --> rn:R07253

rn:R04899 { 3-(2-Hydroxyphenyl)propanoate } <-- 3-(2-Hydroxyphenyl)propanoate -- rn:R05537 <-- Acetyl-CoA --> rn:R07253

rn:R05537 { 3-(2-Hydroxyphenyl)propanoate } <-- 3-(2-Hydroxyphenyl)propanoate -- rn:R05537 <-- Acetyl-CoA --> rn:R07253 # rn:R05537 { 3-(2-Hydroxyphenyl)propanoate } <-- FAD -- rn:R05537 <-- Acetyl-CoA --> rn:R07253

rn:R03369 { 3-(2-Hydroxyphenyl)propanoate } <-- 3-(2-Hydroxyphenyl)propanoate -- rn:R05537 <-- Salicylate --> rn:R07709

rn:R03709 { 3-(2-Hydroxyphenyl)propanoate } <-- 3-(2-Hydroxyphenyl)propanoate -- rn:R05537 <-- Salicylate --> rn:R07709

rn:R04899 { 3-(2-Hydroxyphenyl)propanoate } <-- 3-(2-Hydroxyphenyl)propanoate -- rn:R05537 <-- Salicylate --> rn:R07709

rn:R05537 { 3-(2-Hydroxyphenyl)propanoate } <-- 3-(2-Hydroxyphenyl)propanoate -- rn:R05537 <-- Salicylate --> rn:R07709 # rn:R05537 { 3-(2-Hydroxyphenyl)propanoate } <-- FAD -- rn:R05537 <-- Salicylate --> rn:R07709

rn:R03369 { 3-(2-Hydroxyphenyl)propanoate } <-- 3-(2-Hydroxyphenyl)propanoate -- rn:R05537 <-- Salicylate --> rn:R07710

rn:R03709 { 3-(2-Hydroxyphenyl)propanoate } <-- 3-(2-Hydroxyphenyl)propanoate -- rn:R05537 <-- Salicylate --> rn:R07710

rn:R04899 { 3-(2-Hydroxyphenyl)propanoate } <-- 3-(2-Hydroxyphenyl)propanoate -- rn:R05537 <-- Salicylate --> rn:R07710

rn:R05537 { 3-(2-Hydroxyphenyl)propanoate } <-- 3-(2-Hydroxyphenyl)propanoate -- rn:R05537 <-- Salicylate --> rn:R07710 # rn:R05537 { 3-(2-Hydroxyphenyl)propanoate } <-- FAD -- rn:R05537 <-- Salicylate --> rn:R07710

rn:R03369 { 3-(2-Hydroxyphenyl)propanoate } <-- 3-(2-Hydroxyphenyl)propanoate -- rn:R05537 <-- Acetyl-CoA --> rn:R07937

rn:R03709 { 3-(2-Hydroxyphenyl)propanoate } <-- 3-(2-Hydroxyphenyl)propanoate -- rn:R05537 <-- Acetyl-CoA --> rn:R07937

rn:R04899 { 3-(2-Hydroxyphenyl)propanoate } <-- 3-(2-Hydroxyphenyl)propanoate -- rn:R05537 <-- Acetyl-CoA --> rn:R07937

rn:R05537 { 3-(2-Hydroxyphenyl)propanoate } <-- 3-(2-Hydroxyphenyl)propanoate -- rn:R05537 <-- Acetyl-CoA --> rn:R07937 # rn:R05537 { 3-(2-Hydroxyphenyl)propanoate } <-- FAD -- rn:R05537 <-- Acetyl-CoA --> rn:R07937

rn:R03369 { 3-(2-Hydroxyphenyl)propanoate } <-- 3-(2-Hydroxyphenyl)propanoate -- rn:R05537 <-- Acetyl-CoA --> rn:R07953

rn:R03709 { 3-(2-Hydroxyphenyl)propanoate } <-- 3-(2-Hydroxyphenyl)propanoate -- rn:R05537 <-- Acetyl-CoA --> rn:R07953

rn:R04899 { 3-(2-Hydroxyphenyl)propanoate } <-- 3-(2-Hydroxyphenyl)propanoate -- rn:R05537 <-- Acetyl-CoA --> rn:R07953

rn:R05537 { 3-(2-Hydroxyphenyl)propanoate } <-- 3-(2-Hydroxyphenyl)propanoate -- rn:R05537 <-- Acetyl-CoA --> rn:R07953 # rn:R05537 { 3-(2-Hydroxyphenyl)propanoate } <-- FAD -- rn:R05537 <-- Acetyl-CoA --> rn:R07953

rn:R03369 { 3-(2-Hydroxyphenyl)propanoate } <-- 3-(2-Hydroxyphenyl)propanoate -- rn:R05537 <-- Acetyl-CoA --> rn:R08036

rn:R03709 { 3-(2-Hydroxyphenyl)propanoate } <-- 3-(2-Hydroxyphenyl)propanoate -- rn:R05537 <-- Acetyl-CoA --> rn:R08036

rn:R04899 { 3-(2-Hydroxyphenyl)propanoate } <-- 3-(2-Hydroxyphenyl)propanoate -- rn:R05537 <-- Acetyl-CoA --> rn:R08036

rn:R05537 { 3-(2-Hydroxyphenyl)propanoate } <-- 3-(2-Hydroxyphenyl)propanoate -- rn:R05537 <-- Acetyl-CoA --> rn:R08036 # rn:R05537 { 3-(2-Hydroxyphenyl)propanoate } <-- FAD -- rn:R05537 <-- Acetyl-CoA --> rn:R08036

rn:R03369 { 3-(2-Hydroxyphenyl)propanoate } <-- 3-(2-Hydroxyphenyl)propanoate -- rn:R05537 <-- AMP --> rn:R08743

rn:R03709 { 3-(2-Hydroxyphenyl)propanoate } <-- 3-(2-Hydroxyphenyl)propanoate -- rn:R05537 <-- AMP --> rn:R08743

rn:R04899 { 3-(2-Hydroxyphenyl)propanoate } <-- 3-(2-Hydroxyphenyl)propanoate -- rn:R05537 <-- AMP --> rn:R08743

rn:R05537 { 3-(2-Hydroxyphenyl)propanoate } <-- 3-(2-Hydroxyphenyl)propanoate -- rn:R05537 <-- AMP --> rn:R08743 # rn:R05537 { 3-(2-Hydroxyphenyl)propanoate } <-- FAD -- rn:R05537 <-- AMP --> rn:R08743

rn:R03369 { 3-(2-Hydroxyphenyl)propanoate } <-- 3-(2-Hydroxyphenyl)propanoate -- rn:R05537 <-- Acetyl-CoA --> rn:R08870

rn:R03709 { 3-(2-Hydroxyphenyl)propanoate } <-- 3-(2-Hydroxyphenyl)propanoate -- rn:R05537 <-- Acetyl-CoA --> rn:R08870

rn:R04899 { 3-(2-Hydroxyphenyl)propanoate } <-- 3-(2-Hydroxyphenyl)propanoate -- rn:R05537 <-- Acetyl-CoA --> rn:R08870

rn:R05537 { 3-(2-Hydroxyphenyl)propanoate } <-- 3-(2-Hydroxyphenyl)propanoate -- rn:R05537 <-- Acetyl-CoA --> rn:R08870 # rn:R05537 { 3-(2-Hydroxyphenyl)propanoate } <-- FAD -- rn:R05537 <-- Acetyl-CoA --> rn:R08870

rn:R03369 { 3-(2-Hydroxyphenyl)propanoate } <-- 3-(2-Hydroxyphenyl)propanoate -- rn:R05537 <-- Acetyl-CoA --> rn:R08871

rn:R03709 { 3-(2-Hydroxyphenyl)propanoate } <-- 3-(2-Hydroxyphenyl)propanoate -- rn:R05537 <-- Acetyl-CoA --> rn:R08871

rn:R04899 { 3-(2-Hydroxyphenyl)propanoate } <-- 3-(2-Hydroxyphenyl)propanoate -- rn:R05537 <-- Acetyl-CoA --> rn:R08871

rn:R05537 { 3-(2-Hydroxyphenyl)propanoate } <-- 3-(2-Hydroxyphenyl)propanoate -- rn:R05537 <-- Acetyl-CoA --> rn:R08871 # rn:R05537 { 3-(2-Hydroxyphenyl)propanoate } <-- FAD -- rn:R05537 <-- Acetyl-CoA --> rn:R08871

rn:R03369 { 3-(2-Hydroxyphenyl)propanoate } <-- 3-(2-Hydroxyphenyl)propanoate -- rn:R05537 <-- Acetyl-CoA --> rn:R08938

rn:R03709 { 3-(2-Hydroxyphenyl)propanoate } <-- 3-(2-Hydroxyphenyl)propanoate -- rn:R05537 <-- Acetyl-CoA --> rn:R08938

rn:R04899 { 3-(2-Hydroxyphenyl)propanoate } <-- 3-(2-Hydroxyphenyl)propanoate -- rn:R05537 <-- Acetyl-CoA --> rn:R08938

rn:R05537 { 3-(2-Hydroxyphenyl)propanoate } <-- 3-(2-Hydroxyphenyl)propanoate -- rn:R05537 <-- Acetyl-CoA --> rn:R08938 # rn:R05537 { 3-(2-Hydroxyphenyl)propanoate } <-- FAD -- rn:R05537 <-- Acetyl-CoA --> rn:R08938

rn:R03369 { 3-(2-Hydroxyphenyl)propanoate } <-- 3-(2-Hydroxyphenyl)propanoate -- rn:R05537 <-- FADH2 --> rn:R09517

rn:R03709 { 3-(2-Hydroxyphenyl)propanoate } <-- 3-(2-Hydroxyphenyl)propanoate -- rn:R05537 <-- FADH2 --> rn:R09517

rn:R04899 { 3-(2-Hydroxyphenyl)propanoate } <-- 3-(2-Hydroxyphenyl)propanoate -- rn:R05537 <-- FADH2 --> rn:R09517

rn:R05537 { 3-(2-Hydroxyphenyl)propanoate } <-- 3-(2-Hydroxyphenyl)propanoate -- rn:R05537 <-- FADH2 --> rn:R09517 # rn:R05537 { 3-(2-Hydroxyphenyl)propanoate } <-- FAD -- rn:R05537 <-- FADH2 --> rn:R09517

rn:R03369 { 3-(2-Hydroxyphenyl)propanoate } <-- 3-(2-Hydroxyphenyl)propanoate -- rn:R05537 <-- Salicylate --> rn:R09539

rn:R03709 { 3-(2-Hydroxyphenyl)propanoate } <-- 3-(2-Hydroxyphenyl)propanoate -- rn:R05537 <-- Salicylate --> rn:R09539

rn:R04899 { 3-(2-Hydroxyphenyl)propanoate } <-- 3-(2-Hydroxyphenyl)propanoate -- rn:R05537 <-- Salicylate --> rn:R09539

rn:R05537 { 3-(2-Hydroxyphenyl)propanoate } <-- 3-(2-Hydroxyphenyl)propanoate -- rn:R05537 <-- Salicylate --> rn:R09539 # rn:R05537 { 3-(2-Hydroxyphenyl)propanoate } <-- FAD -- rn:R05537 <-- Salicylate --> rn:R09539

rn:R03369 { 3-(2-Hydroxyphenyl)propanoate } <-- 3-(2-Hydroxyphenyl)propanoate -- rn:R05537 <-- Salicylate --> rn:R10448

rn:R03709 { 3-(2-Hydroxyphenyl)propanoate } <-- 3-(2-Hydroxyphenyl)propanoate -- rn:R05537 <-- Salicylate --> rn:R10448

rn:R04899 { 3-(2-Hydroxyphenyl)propanoate } <-- 3-(2-Hydroxyphenyl)propanoate -- rn:R05537 <-- Salicylate --> rn:R10448

rn:R05537 { 3-(2-Hydroxyphenyl)propanoate } <-- 3-(2-Hydroxyphenyl)propanoate -- rn:R05537 <-- Salicylate --> rn:R10448 # rn:R05537 { 3-(2-Hydroxyphenyl)propanoate } <-- FAD -- rn:R05537 <-- Salicylate --> rn:R10448

rn:R03369 { 3-(2-Hydroxyphenyl)propanoate } <-- 3-(2-Hydroxyphenyl)propanoate -- rn:R05537 <-- Acetyl-CoA --> rn:R10500

rn:R03709 { 3-(2-Hydroxyphenyl)propanoate } <-- 3-(2-Hydroxyphenyl)propanoate -- rn:R05537 <-- Acetyl-CoA --> rn:R10500

rn:R04899 { 3-(2-Hydroxyphenyl)propanoate } <-- 3-(2-Hydroxyphenyl)propanoate -- rn:R05537 <-- Acetyl-CoA --> rn:R10500

rn:R05537 { 3-(2-Hydroxyphenyl)propanoate } <-- 3-(2-Hydroxyphenyl)propanoate -- rn:R05537 <-- Acetyl-CoA --> rn:R10500 # rn:R05537 { 3-(2-Hydroxyphenyl)propanoate } <-- FAD -- rn:R05537 <-- Acetyl-CoA --> rn:R10500

rn:R03369 { 3-(2-Hydroxyphenyl)propanoate } <-- 3-(2-Hydroxyphenyl)propanoate -- rn:R05537 <-- Acetyl-CoA --> rn:R10600

rn:R03709 { 3-(2-Hydroxyphenyl)propanoate } <-- 3-(2-Hydroxyphenyl)propanoate -- rn:R05537 <-- Acetyl-CoA --> rn:R10600

rn:R04899 { 3-(2-Hydroxyphenyl)propanoate } <-- 3-(2-Hydroxyphenyl)propanoate -- rn:R05537 <-- Acetyl-CoA --> rn:R10600

rn:R05537 { 3-(2-Hydroxyphenyl)propanoate } <-- 3-(2-Hydroxyphenyl)propanoate -- rn:R05537 <-- Acetyl-CoA --> rn:R10600 # rn:R05537 { 3-(2-Hydroxyphenyl)propanoate } <-- FAD -- rn:R05537 <-- Acetyl-CoA --> rn:R10600

rn:R03369 { 3-(2-Hydroxyphenyl)propanoate } <-- 3-(2-Hydroxyphenyl)propanoate -- rn:R05537 <-- Acetyl-CoA --> rn:R10745

rn:R03709 { 3-(2-Hydroxyphenyl)propanoate } <-- 3-(2-Hydroxyphenyl)propanoate -- rn:R05537 <-- Acetyl-CoA --> rn:R10745

rn:R04899 { 3-(2-Hydroxyphenyl)propanoate } <-- 3-(2-Hydroxyphenyl)propanoate -- rn:R05537 <-- Acetyl-CoA --> rn:R10745

rn:R05537 { 3-(2-Hydroxyphenyl)propanoate } <-- 3-(2-Hydroxyphenyl)propanoate -- rn:R05537 <-- Acetyl-CoA --> rn:R10745 # rn:R05537 { 3-(2-Hydroxyphenyl)propanoate } <-- FAD -- rn:R05537 <-- Acetyl-CoA --> rn:R10745

rn:R03369 { 3-(2-Hydroxyphenyl)propanoate } <-- 3-(2-Hydroxyphenyl)propanoate -- rn:R05537 <-- Acetyl-CoA --> rn:R10746

rn:R03709 { 3-(2-Hydroxyphenyl)propanoate } <-- 3-(2-Hydroxyphenyl)propanoate -- rn:R05537 <-- Acetyl-CoA --> rn:R10746

rn:R04899 { 3-(2-Hydroxyphenyl)propanoate } <-- 3-(2-Hydroxyphenyl)propanoate -- rn:R05537 <-- Acetyl-CoA --> rn:R10746

rn:R05537 { 3-(2-Hydroxyphenyl)propanoate } <-- 3-(2-Hydroxyphenyl)propanoate -- rn:R05537 <-- Acetyl-CoA --> rn:R10746 # rn:R05537 { 3-(2-Hydroxyphenyl)propanoate } <-- FAD -- rn:R05537 <-- Acetyl-CoA --> rn:R10746

rn:R03369 { 3-(2-Hydroxyphenyl)propanoate } <-- 3-(2-Hydroxyphenyl)propanoate -- rn:R05537 <-- Acetyl-CoA --> rn:R10893

rn:R03709 { 3-(2-Hydroxyphenyl)propanoate } <-- 3-(2-Hydroxyphenyl)propanoate -- rn:R05537 <-- Acetyl-CoA --> rn:R10893

rn:R04899 { 3-(2-Hydroxyphenyl)propanoate } <-- 3-(2-Hydroxyphenyl)propanoate -- rn:R05537 <-- Acetyl-CoA --> rn:R10893

rn:R05537 { 3-(2-Hydroxyphenyl)propanoate } <-- 3-(2-Hydroxyphenyl)propanoate -- rn:R05537 <-- Acetyl-CoA --> rn:R10893 # rn:R05537 { 3-(2-Hydroxyphenyl)propanoate } <-- FAD -- rn:R05537 <-- Acetyl-CoA --> rn:R10893

rn:R03369 { 3-(2-Hydroxyphenyl)propanoate } <-- 3-(2-Hydroxyphenyl)propanoate -- rn:R05537 <-- Acetyl-CoA --> rn:R11124

rn:R03709 { 3-(2-Hydroxyphenyl)propanoate } <-- 3-(2-Hydroxyphenyl)propanoate -- rn:R05537 <-- Acetyl-CoA --> rn:R11124

rn:R04899 { 3-(2-Hydroxyphenyl)propanoate } <-- 3-(2-Hydroxyphenyl)propanoate -- rn:R05537 <-- Acetyl-CoA --> rn:R11124

rn:R05537 { 3-(2-Hydroxyphenyl)propanoate } <-- 3-(2-Hydroxyphenyl)propanoate -- rn:R05537 <-- Acetyl-CoA --> rn:R11124 # rn:R05537 { 3-(2-Hydroxyphenyl)propanoate } <-- FAD -- rn:R05537 <-- Acetyl-CoA --> rn:R11124

rn:R03369 { 3-(2-Hydroxyphenyl)propanoate } <-- 3-(2-Hydroxyphenyl)propanoate -- rn:R05537 <-- Acetyl-CoA --> rn:R11125

rn:R03709 { 3-(2-Hydroxyphenyl)propanoate } <-- 3-(2-Hydroxyphenyl)propanoate -- rn:R05537 <-- Acetyl-CoA --> rn:R11125

rn:R04899 { 3-(2-Hydroxyphenyl)propanoate } <-- 3-(2-Hydroxyphenyl)propanoate -- rn:R05537 <-- Acetyl-CoA --> rn:R11125

rn:R05537 { 3-(2-Hydroxyphenyl)propanoate } <-- 3-(2-Hydroxyphenyl)propanoate -- rn:R05537 <-- Acetyl-CoA --> rn:R11125 # rn:R05537 { 3-(2-Hydroxyphenyl)propanoate } <-- FAD -- rn:R05537 <-- Acetyl-CoA --> rn:R11125

rn:R03369 { 3-(2-Hydroxyphenyl)propanoate } <-- 3-(2-Hydroxyphenyl)propanoate -- rn:R05537 <-- Acetyl-CoA --> rn:R11479

rn:R03709 { 3-(2-Hydroxyphenyl)propanoate } <-- 3-(2-Hydroxyphenyl)propanoate -- rn:R05537 <-- Acetyl-CoA --> rn:R11479

rn:R04899 { 3-(2-Hydroxyphenyl)propanoate } <-- 3-(2-Hydroxyphenyl)propanoate -- rn:R05537 <-- Acetyl-CoA --> rn:R11479

rn:R05537 { 3-(2-Hydroxyphenyl)propanoate } <-- 3-(2-Hydroxyphenyl)propanoate -- rn:R05537 <-- Acetyl-CoA --> rn:R11479 # rn:R05537 { 3-(2-Hydroxyphenyl)propanoate } <-- FAD -- rn:R05537 <-- Acetyl-CoA --> rn:R11479

rn:R03369 { 3-(2-Hydroxyphenyl)propanoate } <-- 3-(2-Hydroxyphenyl)propanoate -- rn:R05537 <-- FADH2 --> rn:R11653

rn:R03709 { 3-(2-Hydroxyphenyl)propanoate } <-- 3-(2-Hydroxyphenyl)propanoate -- rn:R05537 <-- FADH2 --> rn:R11653

rn:R04899 { 3-(2-Hydroxyphenyl)propanoate } <-- 3-(2-Hydroxyphenyl)propanoate -- rn:R05537 <-- FADH2 --> rn:R11653

rn:R05537 { 3-(2-Hydroxyphenyl)propanoate } <-- 3-(2-Hydroxyphenyl)propanoate -- rn:R05537 <-- FADH2 --> rn:R11653 # rn:R05537 { 3-(2-Hydroxyphenyl)propanoate } <-- FAD -- rn:R05537 <-- FADH2 --> rn:R11653

rn:R03369 { 3-(2-Hydroxyphenyl)propanoate } <-- 3-(2-Hydroxyphenyl)propanoate -- rn:R05537 <-- AMP --> rn:R11679

rn:R03709 { 3-(2-Hydroxyphenyl)propanoate } <-- 3-(2-Hydroxyphenyl)propanoate -- rn:R05537 <-- AMP --> rn:R11679

rn:R04899 { 3-(2-Hydroxyphenyl)propanoate } <-- 3-(2-Hydroxyphenyl)propanoate -- rn:R05537 <-- AMP --> rn:R11679

rn:R05537 { 3-(2-Hydroxyphenyl)propanoate } <-- 3-(2-Hydroxyphenyl)propanoate -- rn:R05537 <-- AMP --> rn:R11679 # rn:R05537 { 3-(2-Hydroxyphenyl)propanoate } <-- FAD -- rn:R05537 <-- AMP --> rn:R11679

rn:R03369 { 3-(2-Hydroxyphenyl)propanoate } <-- 3-(2-Hydroxyphenyl)propanoate -- rn:R05537 <-- Acetyl-CoA --> rn:R11708

rn:R03709 { 3-(2-Hydroxyphenyl)propanoate } <-- 3-(2-Hydroxyphenyl)propanoate -- rn:R05537 <-- Acetyl-CoA --> rn:R11708

rn:R04899 { 3-(2-Hydroxyphenyl)propanoate } <-- 3-(2-Hydroxyphenyl)propanoate -- rn:R05537 <-- Acetyl-CoA --> rn:R11708

rn:R05537 { 3-(2-Hydroxyphenyl)propanoate } <-- 3-(2-Hydroxyphenyl)propanoate -- rn:R05537 <-- Acetyl-CoA --> rn:R11708 # rn:R05537 { 3-(2-Hydroxyphenyl)propanoate } <-- FAD -- rn:R05537 <-- Acetyl-CoA --> rn:R11708

rn:R03369 { 3-(2-Hydroxyphenyl)propanoate } <-- 3-(2-Hydroxyphenyl)propanoate -- rn:R05537 <-- Acetyl-CoA --> rn:R11902

rn:R03709 { 3-(2-Hydroxyphenyl)propanoate } <-- 3-(2-Hydroxyphenyl)propanoate -- rn:R05537 <-- Acetyl-CoA --> rn:R11902

rn:R04899 { 3-(2-Hydroxyphenyl)propanoate } <-- 3-(2-Hydroxyphenyl)propanoate -- rn:R05537 <-- Acetyl-CoA --> rn:R11902

rn:R05537 { 3-(2-Hydroxyphenyl)propanoate } <-- 3-(2-Hydroxyphenyl)propanoate -- rn:R05537 <-- Acetyl-CoA --> rn:R11902 # rn:R05537 { 3-(2-Hydroxyphenyl)propanoate } <-- FAD -- rn:R05537 <-- Acetyl-CoA --> rn:R11902

rn:R03369 { 3-(2-Hydroxyphenyl)propanoate } <-- 3-(2-Hydroxyphenyl)propanoate -- rn:R05537 <-- FADH2 --> rn:R12021

rn:R03709 { 3-(2-Hydroxyphenyl)propanoate } <-- 3-(2-Hydroxyphenyl)propanoate -- rn:R05537 <-- FADH2 --> rn:R12021

rn:R04899 { 3-(2-Hydroxyphenyl)propanoate } <-- 3-(2-Hydroxyphenyl)propanoate -- rn:R05537 <-- FADH2 --> rn:R12021

rn:R05537 { 3-(2-Hydroxyphenyl)propanoate } <-- 3-(2-Hydroxyphenyl)propanoate -- rn:R05537 <-- FADH2 --> rn:R12021 # rn:R05537 { 3-(2-Hydroxyphenyl)propanoate } <-- FAD -- rn:R05537 <-- FADH2 --> rn:R12021

rn:R03369 { 3-(2-Hydroxyphenyl)propanoate } <-- 3-(2-Hydroxyphenyl)propanoate -- rn:R05537 <-- FADH2 --> rn:R12023

rn:R03709 { 3-(2-Hydroxyphenyl)propanoate } <-- 3-(2-Hydroxyphenyl)propanoate -- rn:R05537 <-- FADH2 --> rn:R12023

rn:R04899 { 3-(2-Hydroxyphenyl)propanoate } <-- 3-(2-Hydroxyphenyl)propanoate -- rn:R05537 <-- FADH2 --> rn:R12023

rn:R05537 { 3-(2-Hydroxyphenyl)propanoate } <-- 3-(2-Hydroxyphenyl)propanoate -- rn:R05537 <-- FADH2 --> rn:R12023 # rn:R05537 { 3-(2-Hydroxyphenyl)propanoate } <-- FAD -- rn:R05537 <-- FADH2 --> rn:R12023

rn:R03369 { 3-(2-Hydroxyphenyl)propanoate } <-- 3-(2-Hydroxyphenyl)propanoate -- rn:R05537 <-- FADH2 --> rn:R12027

rn:R03709 { 3-(2-Hydroxyphenyl)propanoate } <-- 3-(2-Hydroxyphenyl)propanoate -- rn:R05537 <-- FADH2 --> rn:R12027

rn:R04899 { 3-(2-Hydroxyphenyl)propanoate } <-- 3-(2-Hydroxyphenyl)propanoate -- rn:R05537 <-- FADH2 --> rn:R12027

rn:R05537 { 3-(2-Hydroxyphenyl)propanoate } <-- 3-(2-Hydroxyphenyl)propanoate -- rn:R05537 <-- FADH2 --> rn:R12027 # rn:R05537 { 3-(2-Hydroxyphenyl)propanoate } <-- FAD -- rn:R05537 <-- FADH2 --> rn:R12027

rn:R03369 { 3-(2-Hydroxyphenyl)propanoate } <-- 3-(2-Hydroxyphenyl)propanoate -- rn:R05537 <-- FADH2 --> rn:R12030

rn:R03709 { 3-(2-Hydroxyphenyl)propanoate } <-- 3-(2-Hydroxyphenyl)propanoate -- rn:R05537 <-- FADH2 --> rn:R12030

rn:R04899 { 3-(2-Hydroxyphenyl)propanoate } <-- 3-(2-Hydroxyphenyl)propanoate -- rn:R05537 <-- FADH2 --> rn:R12030

rn:R05537 { 3-(2-Hydroxyphenyl)propanoate } <-- 3-(2-Hydroxyphenyl)propanoate -- rn:R05537 <-- FADH2 --> rn:R12030 # rn:R05537 { 3-(2-Hydroxyphenyl)propanoate } <-- FAD -- rn:R05537 <-- FADH2 --> rn:R12030

rn:R02234 { Cyclohexanone } <-- Acceptor -- rn:R05704 <-- Hydrogen cyanide --> rn:R00152

rn:R03212 { 3-Hydroxycyclohexanone } <-- Acceptor -- rn:R05704 <-- Hydrogen cyanide --> rn:R00152

rn:R02234 { Cyclohexanone } <-- Acceptor -- rn:R05704 <-- Hydrogen cyanide --> rn:R01410

rn:R03212 { 3-Hydroxycyclohexanone } <-- Acceptor -- rn:R05704 <-- Hydrogen cyanide --> rn:R01410

rn:R02234 { Cyclohexanone } <-- Acceptor -- rn:R05704 <-- Hydrogen cyanide --> rn:R01650

rn:R03212 { 3-Hydroxycyclohexanone } <-- Acceptor -- rn:R05704 <-- Hydrogen cyanide --> rn:R01650

rn:R02234 { Cyclohexanone } <-- Acceptor -- rn:R05704 <-- Hydrogen cyanide --> rn:R03524

rn:R03212 { 3-Hydroxycyclohexanone } <-- Acceptor -- rn:R05704 <-- Hydrogen cyanide --> rn:R03524

rn:R02468 { (-)-Limonene } <-- [Reduced NADPH---hemoprotein reductase] -- rn:R05728 <-- (S)-4-Hydroxymandelonitrile --> rn:R02676

rn:R02469 { (-)-Limonene } <-- [Reduced NADPH---hemoprotein reductase] -- rn:R05728 <-- (S)-4-Hydroxymandelonitrile --> rn:R02676

rn:R02470 { (-)-Limonene } <-- [Reduced NADPH---hemoprotein reductase] -- rn:R05728 <-- (S)-4-Hydroxymandelonitrile --> rn:R02676

rn:R04366 { Linalool } <-- [Reduced NADPH---hemoprotein reductase] -- rn:R05728 <-- (S)-4-Hydroxymandelonitrile --> rn:R02676

rn:R06119 { d-Limonene } <-- [Reduced NADPH---hemoprotein reductase] -- rn:R05728 <-- (S)-4-Hydroxymandelonitrile --> rn:R02676

rn:R09451 { Hexadecanoic acid } <-- [Reduced NADPH---hemoprotein reductase] -- rn:R05728 <-- (S)-4-Hydroxymandelonitrile --> rn:R02676

rn:R09452 { (9Z)-Octadecenoic acid } <-- [Reduced NADPH---hemoprotein reductase] -- rn:R05728 <-- (S)-4-Hydroxymandelonitrile --> rn:R02676

rn:R09922 { (+)-Linalool } <-- [Reduced NADPH---hemoprotein reductase] -- rn:R05728 <-- (S)-4-Hydroxymandelonitrile --> rn:R02676

rn:R09923 { (-)-Linalool } <-- [Reduced NADPH---hemoprotein reductase] -- rn:R05728 <-- (S)-4-Hydroxymandelonitrile --> rn:R02676

rn:R09925 { Linalool } <-- [Reduced NADPH---hemoprotein reductase] -- rn:R05728 <-- (S)-4-Hydroxymandelonitrile --> rn:R02676

rn:R09934 { Humulene } <-- [Reduced NADPH---hemoprotein reductase] -- rn:R05728 <-- (S)-4-Hydroxymandelonitrile --> rn:R02676

rn:R10562 { (E,E)-Geranyllinalool } <-- [Reduced NADPH---hemoprotein reductase] -- rn:R05728 <-- (S)-4-Hydroxymandelonitrile --> rn:R02676

rn:R11055 { Myrcene } <-- [Reduced NADPH---hemoprotein reductase] -- rn:R05728 <-- (S)-4-Hydroxymandelonitrile --> rn:R02676

rn:R02468 { (-)-Limonene } <-- [Reduced NADPH---hemoprotein reductase] -- rn:R05728 <-- (S)-4-Hydroxymandelonitrile --> rn:R04296

rn:R02469 { (-)-Limonene } <-- [Reduced NADPH---hemoprotein reductase] -- rn:R05728 <-- (S)-4-Hydroxymandelonitrile --> rn:R04296

rn:R02470 { (-)-Limonene } <-- [Reduced NADPH---hemoprotein reductase] -- rn:R05728 <-- (S)-4-Hydroxymandelonitrile --> rn:R04296

rn:R04366 { Linalool } <-- [Reduced NADPH---hemoprotein reductase] -- rn:R05728 <-- (S)-4-Hydroxymandelonitrile --> rn:R04296

rn:R06119 { d-Limonene } <-- [Reduced NADPH---hemoprotein reductase] -- rn:R05728 <-- (S)-4-Hydroxymandelonitrile --> rn:R04296

rn:R09451 { Hexadecanoic acid } <-- [Reduced NADPH---hemoprotein reductase] -- rn:R05728 <-- (S)-4-Hydroxymandelonitrile --> rn:R04296

rn:R09452 { (9Z)-Octadecenoic acid } <-- [Reduced NADPH---hemoprotein reductase] -- rn:R05728 <-- (S)-4-Hydroxymandelonitrile --> rn:R04296

rn:R09922 { (+)-Linalool } <-- [Reduced NADPH---hemoprotein reductase] -- rn:R05728 <-- (S)-4-Hydroxymandelonitrile --> rn:R04296

rn:R09923 { (-)-Linalool } <-- [Reduced NADPH---hemoprotein reductase] -- rn:R05728 <-- (S)-4-Hydroxymandelonitrile --> rn:R04296

rn:R09925 { Linalool } <-- [Reduced NADPH---hemoprotein reductase] -- rn:R05728 <-- (S)-4-Hydroxymandelonitrile --> rn:R04296

rn:R09934 { Humulene } <-- [Reduced NADPH---hemoprotein reductase] -- rn:R05728 <-- (S)-4-Hydroxymandelonitrile --> rn:R04296

rn:R10562 { (E,E)-Geranyllinalool } <-- [Reduced NADPH---hemoprotein reductase] -- rn:R05728 <-- (S)-4-Hydroxymandelonitrile --> rn:R04296

rn:R11055 { Myrcene } <-- [Reduced NADPH---hemoprotein reductase] -- rn:R05728 <-- (S)-4-Hydroxymandelonitrile --> rn:R04296

rn:R02468 { (-)-Limonene } <-- [Reduced NADPH---hemoprotein reductase] -- rn:R05728 <-- [Oxidized NADPH---hemoprotein reductase] --> rn:R08551

rn:R02469 { (-)-Limonene } <-- [Reduced NADPH---hemoprotein reductase] -- rn:R05728 <-- [Oxidized NADPH---hemoprotein reductase] --> rn:R08551

rn:R02470 { (-)-Limonene } <-- [Reduced NADPH---hemoprotein reductase] -- rn:R05728 <-- [Oxidized NADPH---hemoprotein reductase] --> rn:R08551

rn:R04366 { Linalool } <-- [Reduced NADPH---hemoprotein reductase] -- rn:R05728 <-- [Oxidized NADPH---hemoprotein reductase] --> rn:R08551

rn:R06119 { d-Limonene } <-- [Reduced NADPH---hemoprotein reductase] -- rn:R05728 <-- [Oxidized NADPH---hemoprotein reductase] --> rn:R08551

rn:R09451 { Hexadecanoic acid } <-- [Reduced NADPH---hemoprotein reductase] -- rn:R05728 <-- [Oxidized NADPH---hemoprotein reductase] --> rn:R08551

rn:R09452 { (9Z)-Octadecenoic acid } <-- [Reduced NADPH---hemoprotein reductase] -- rn:R05728 <-- [Oxidized NADPH---hemoprotein reductase] --> rn:R08551

rn:R09922 { (+)-Linalool } <-- [Reduced NADPH---hemoprotein reductase] -- rn:R05728 <-- [Oxidized NADPH---hemoprotein reductase] --> rn:R08551

rn:R09923 { (-)-Linalool } <-- [Reduced NADPH---hemoprotein reductase] -- rn:R05728 <-- [Oxidized NADPH---hemoprotein reductase] --> rn:R08551

rn:R09925 { Linalool } <-- [Reduced NADPH---hemoprotein reductase] -- rn:R05728 <-- [Oxidized NADPH---hemoprotein reductase] --> rn:R08551

rn:R09934 { Humulene } <-- [Reduced NADPH---hemoprotein reductase] -- rn:R05728 <-- [Oxidized NADPH---hemoprotein reductase] --> rn:R08551

rn:R10562 { (E,E)-Geranyllinalool } <-- [Reduced NADPH---hemoprotein reductase] -- rn:R05728 <-- [Oxidized NADPH---hemoprotein reductase] --> rn:R08551

rn:R11055 { Myrcene } <-- [Reduced NADPH---hemoprotein reductase] -- rn:R05728 <-- [Oxidized NADPH---hemoprotein reductase] --> rn:R08551

rn:R02234 { Cyclohexanone } <-- Acceptor -- rn:R05753 <-- Tetrachloroethene --> rn:R05499

rn:R03212 { 3-Hydroxycyclohexanone } <-- Acceptor -- rn:R05753 <-- Tetrachloroethene --> rn:R05499

rn:R02781 { 2,4,6/3,5-Pentahydroxycyclohexanone } <-- L-Glutamine -- rn:R05815 <-- L-Glutamate --> rn:R00114

rn:R02781 { 2,4,6/3,5-Pentahydroxycyclohexanone } <-- L-Glutamine -- rn:R05815 <-- L-Glutamate --> rn:R00248

rn:R02781 { 2,4,6/3,5-Pentahydroxycyclohexanone } <-- L-Glutamine -- rn:R05815 <-- L-Glutamate --> rn:R00254

rn:R02781 { 2,4,6/3,5-Pentahydroxycyclohexanone } <-- L-Glutamine -- rn:R05815 <-- L-Glutamate --> rn:R00894

rn:R02781 { 2,4,6/3,5-Pentahydroxycyclohexanone } <-- L-Glutamine -- rn:R05815 <-- L-Glutamate --> rn:R02287

rn:R02781 { 2,4,6/3,5-Pentahydroxycyclohexanone } <-- L-Glutamine -- rn:R05815 <-- L-Glutamate --> rn:R03189

rn:R02781 { 2,4,6/3,5-Pentahydroxycyclohexanone } <-- L-Glutamine -- rn:R05815 <-- L-Glutamate --> rn:R03970

rn:R02781 { 2,4,6/3,5-Pentahydroxycyclohexanone } <-- L-Glutamine -- rn:R05815 <-- L-Glutamate --> rn:R03971

rn:R02781 { 2,4,6/3,5-Pentahydroxycyclohexanone } <-- L-Glutamine -- rn:R05815 <-- L-Glutamate --> rn:R04051

rn:R02781 { 2,4,6/3,5-Pentahydroxycyclohexanone } <-- L-Glutamine -- rn:R05815 <-- L-Glutamate --> rn:R04776

rn:R02781 { 2,4,6/3,5-Pentahydroxycyclohexanone } <-- L-Glutamine -- rn:R05815 <-- Cob(II)yrinate a,c diamide --> rn:R05218

rn:R02781 { 2,4,6/3,5-Pentahydroxycyclohexanone } <-- L-Glutamine -- rn:R05815 <-- L-Glutamate --> rn:R07643

rn:R02781 { 2,4,6/3,5-Pentahydroxycyclohexanone } <-- L-Glutamine -- rn:R05815 <-- Cob(II)yrinate a,c diamide --> rn:R12184

rn:R10453 { 3-Amino-4,7-dihydroxycoumarin } <-- 3-Dimethylallyl-4-hydroxybenzoate -- rn:R06776 <-- Chlorobiocic acid --> rn:R06773

rn:R05488 { Styrene } <-- FADH2 -- rn:R06902 <-- FAD --> rn:R02487

rn:R05488 { Styrene } <-- FADH2 -- rn:R06902 <-- FAD --> rn:R04095

rn:R05488 { Styrene } <-- FADH2 -- rn:R06902 <-- FAD --> rn:R05537

rn:R05488 { Styrene } <-- FADH2 -- rn:R06902 <-- FAD --> rn:R06943

rn:R05488 { Styrene } <-- FADH2 -- rn:R06902 <-- FAD --> rn:R07220

rn:R05488 { Styrene } <-- FADH2 -- rn:R06902 <-- FAD --> rn:R09520

rn:R05488 { Styrene } <-- FADH2 -- rn:R06902 <-- 1-Hydroxy-2-naphthoate --> rn:R09818

rn:R05488 { Styrene } <-- FADH2 -- rn:R06902 <-- FAD --> rn:R11130

rn:R05537 { 3-(2-Hydroxyphenyl)propanoate } <-- FAD -- rn:R06943 <-- FADH2 --> rn:R03978

rn:R05537 { 3-(2-Hydroxyphenyl)propanoate } <-- FAD -- rn:R06943 <-- FADH2 --> rn:R05488

rn:R05537 { 3-(2-Hydroxyphenyl)propanoate } <-- FAD -- rn:R06943 <-- 5-Carboxy-2-pentenoyl-CoA --> rn:R06942

rn:R05537 { 3-(2-Hydroxyphenyl)propanoate } <-- FAD -- rn:R06943 <-- FADH2 --> rn:R09517

rn:R05537 { 3-(2-Hydroxyphenyl)propanoate } <-- FAD -- rn:R06943 <-- FADH2 --> rn:R11653

rn:R05537 { 3-(2-Hydroxyphenyl)propanoate } <-- FAD -- rn:R06943 <-- FADH2 --> rn:R12021

rn:R05537 { 3-(2-Hydroxyphenyl)propanoate } <-- FAD -- rn:R06943 <-- FADH2 --> rn:R12023

rn:R05537 { 3-(2-Hydroxyphenyl)propanoate } <-- FAD -- rn:R06943 <-- FADH2 --> rn:R12027

rn:R05537 { 3-(2-Hydroxyphenyl)propanoate } <-- FAD -- rn:R06943 <-- FADH2 --> rn:R12030

rn:R02468 { (-)-Limonene } <-- [Reduced NADPH---hemoprotein reductase] -- rn:R07041 <-- [Oxidized NADPH---hemoprotein reductase] --> rn:R08551

rn:R02469 { (-)-Limonene } <-- [Reduced NADPH---hemoprotein reductase] -- rn:R07041 <-- [Oxidized NADPH---hemoprotein reductase] --> rn:R08551

rn:R02470 { (-)-Limonene } <-- [Reduced NADPH---hemoprotein reductase] -- rn:R07041 <-- [Oxidized NADPH---hemoprotein reductase] --> rn:R08551

rn:R04366 { Linalool } <-- [Reduced NADPH---hemoprotein reductase] -- rn:R07041 <-- [Oxidized NADPH---hemoprotein reductase] --> rn:R08551

rn:R06119 { d-Limonene } <-- [Reduced NADPH---hemoprotein reductase] -- rn:R07041 <-- [Oxidized NADPH---hemoprotein reductase] --> rn:R08551

rn:R09451 { Hexadecanoic acid } <-- [Reduced NADPH---hemoprotein reductase] -- rn:R07041 <-- [Oxidized NADPH---hemoprotein reductase] --> rn:R08551

rn:R09452 { (9Z)-Octadecenoic acid } <-- [Reduced NADPH---hemoprotein reductase] -- rn:R07041 <-- [Oxidized NADPH---hemoprotein reductase] --> rn:R08551

rn:R09922 { (+)-Linalool } <-- [Reduced NADPH---hemoprotein reductase] -- rn:R07041 <-- [Oxidized NADPH---hemoprotein reductase] --> rn:R08551

rn:R09923 { (-)-Linalool } <-- [Reduced NADPH---hemoprotein reductase] -- rn:R07041 <-- [Oxidized NADPH---hemoprotein reductase] --> rn:R08551

rn:R09925 { Linalool } <-- [Reduced NADPH---hemoprotein reductase] -- rn:R07041 <-- [Oxidized NADPH---hemoprotein reductase] --> rn:R08551

rn:R09934 { Humulene } <-- [Reduced NADPH---hemoprotein reductase] -- rn:R07041 <-- [Oxidized NADPH---hemoprotein reductase] --> rn:R08551

rn:R10562 { (E,E)-Geranyllinalool } <-- [Reduced NADPH---hemoprotein reductase] -- rn:R07041 <-- [Oxidized NADPH---hemoprotein reductase] --> rn:R08551

rn:R11055 { Myrcene } <-- [Reduced NADPH---hemoprotein reductase] -- rn:R07041 <-- [Oxidized NADPH---hemoprotein reductase] --> rn:R08551

rn:R02468 { (-)-Limonene } <-- [Reduced NADPH---hemoprotein reductase] -- rn:R07046 <-- [Oxidized NADPH---hemoprotein reductase] --> rn:R08551

rn:R02469 { (-)-Limonene } <-- [Reduced NADPH---hemoprotein reductase] -- rn:R07046 <-- [Oxidized NADPH---hemoprotein reductase] --> rn:R08551

rn:R02470 { (-)-Limonene } <-- [Reduced NADPH---hemoprotein reductase] -- rn:R07046 <-- [Oxidized NADPH---hemoprotein reductase] --> rn:R08551

rn:R04366 { Linalool } <-- [Reduced NADPH---hemoprotein reductase] -- rn:R07046 <-- [Oxidized NADPH---hemoprotein reductase] --> rn:R08551

rn:R06119 { d-Limonene } <-- [Reduced NADPH---hemoprotein reductase] -- rn:R07046 <-- [Oxidized NADPH---hemoprotein reductase] --> rn:R08551

rn:R09451 { Hexadecanoic acid } <-- [Reduced NADPH---hemoprotein reductase] -- rn:R07046 <-- [Oxidized NADPH---hemoprotein reductase] --> rn:R08551

rn:R09452 { (9Z)-Octadecenoic acid } <-- [Reduced NADPH---hemoprotein reductase] -- rn:R07046 <-- [Oxidized NADPH---hemoprotein reductase] --> rn:R08551

rn:R09922 { (+)-Linalool } <-- [Reduced NADPH---hemoprotein reductase] -- rn:R07046 <-- [Oxidized NADPH---hemoprotein reductase] --> rn:R08551

rn:R09923 { (-)-Linalool } <-- [Reduced NADPH---hemoprotein reductase] -- rn:R07046 <-- [Oxidized NADPH---hemoprotein reductase] --> rn:R08551

rn:R09925 { Linalool } <-- [Reduced NADPH---hemoprotein reductase] -- rn:R07046 <-- [Oxidized NADPH---hemoprotein reductase] --> rn:R08551

rn:R09934 { Humulene } <-- [Reduced NADPH---hemoprotein reductase] -- rn:R07046 <-- [Oxidized NADPH---hemoprotein reductase] --> rn:R08551

rn:R10562 { (E,E)-Geranyllinalool } <-- [Reduced NADPH---hemoprotein reductase] -- rn:R07046 <-- [Oxidized NADPH---hemoprotein reductase] --> rn:R08551

rn:R11055 { Myrcene } <-- [Reduced NADPH---hemoprotein reductase] -- rn:R07046 <-- [Oxidized NADPH---hemoprotein reductase] --> rn:R08551

rn:R02234 { Cyclohexanone } <-- Acceptor -- rn:R07154 <-- Isopyridoxal --> rn:R05084

rn:R03212 { 3-Hydroxycyclohexanone } <-- Acceptor -- rn:R07154 <-- Isopyridoxal --> rn:R05084

rn:R02468 { (-)-Limonene } <-- [Reduced NADPH---hemoprotein reductase] -- rn:R07203 <-- [Oxidized NADPH---hemoprotein reductase] --> rn:R08551

rn:R02469 { (-)-Limonene } <-- [Reduced NADPH---hemoprotein reductase] -- rn:R07203 <-- [Oxidized NADPH---hemoprotein reductase] --> rn:R08551

rn:R02470 { (-)-Limonene } <-- [Reduced NADPH---hemoprotein reductase] -- rn:R07203 <-- [Oxidized NADPH---hemoprotein reductase] --> rn:R08551

rn:R04366 { Linalool } <-- [Reduced NADPH---hemoprotein reductase] -- rn:R07203 <-- [Oxidized NADPH---hemoprotein reductase] --> rn:R08551

rn:R06119 { d-Limonene } <-- [Reduced NADPH---hemoprotein reductase] -- rn:R07203 <-- [Oxidized NADPH---hemoprotein reductase] --> rn:R08551

rn:R09451 { Hexadecanoic acid } <-- [Reduced NADPH---hemoprotein reductase] -- rn:R07203 <-- [Oxidized NADPH---hemoprotein reductase] --> rn:R08551

rn:R09452 { (9Z)-Octadecenoic acid } <-- [Reduced NADPH---hemoprotein reductase] -- rn:R07203 <-- [Oxidized NADPH---hemoprotein reductase] --> rn:R08551

rn:R09922 { (+)-Linalool } <-- [Reduced NADPH---hemoprotein reductase] -- rn:R07203 <-- [Oxidized NADPH---hemoprotein reductase] --> rn:R08551

rn:R09923 { (-)-Linalool } <-- [Reduced NADPH---hemoprotein reductase] -- rn:R07203 <-- [Oxidized NADPH---hemoprotein reductase] --> rn:R08551

rn:R09925 { Linalool } <-- [Reduced NADPH---hemoprotein reductase] -- rn:R07203 <-- [Oxidized NADPH---hemoprotein reductase] --> rn:R08551

rn:R09934 { Humulene } <-- [Reduced NADPH---hemoprotein reductase] -- rn:R07203 <-- [Oxidized NADPH---hemoprotein reductase] --> rn:R08551

rn:R10562 { (E,E)-Geranyllinalool } <-- [Reduced NADPH---hemoprotein reductase] -- rn:R07203 <-- [Oxidized NADPH---hemoprotein reductase] --> rn:R08551

rn:R11055 { Myrcene } <-- [Reduced NADPH---hemoprotein reductase] -- rn:R07203 <-- [Oxidized NADPH---hemoprotein reductase] --> rn:R08551

rn:R02468 { (-)-Limonene } <-- [Reduced NADPH---hemoprotein reductase] -- rn:R07205 <-- [Oxidized NADPH---hemoprotein reductase] --> rn:R08551

rn:R02469 { (-)-Limonene } <-- [Reduced NADPH---hemoprotein reductase] -- rn:R07205 <-- [Oxidized NADPH---hemoprotein reductase] --> rn:R08551

rn:R02470 { (-)-Limonene } <-- [Reduced NADPH---hemoprotein reductase] -- rn:R07205 <-- [Oxidized NADPH---hemoprotein reductase] --> rn:R08551

rn:R04366 { Linalool } <-- [Reduced NADPH---hemoprotein reductase] -- rn:R07205 <-- [Oxidized NADPH---hemoprotein reductase] --> rn:R08551

rn:R06119 { d-Limonene } <-- [Reduced NADPH---hemoprotein reductase] -- rn:R07205 <-- [Oxidized NADPH---hemoprotein reductase] --> rn:R08551

rn:R09451 { Hexadecanoic acid } <-- [Reduced NADPH---hemoprotein reductase] -- rn:R07205 <-- [Oxidized NADPH---hemoprotein reductase] --> rn:R08551

rn:R09452 { (9Z)-Octadecenoic acid } <-- [Reduced NADPH---hemoprotein reductase] -- rn:R07205 <-- [Oxidized NADPH---hemoprotein reductase] --> rn:R08551

rn:R09922 { (+)-Linalool } <-- [Reduced NADPH---hemoprotein reductase] -- rn:R07205 <-- [Oxidized NADPH---hemoprotein reductase] --> rn:R08551

rn:R09923 { (-)-Linalool } <-- [Reduced NADPH---hemoprotein reductase] -- rn:R07205 <-- [Oxidized NADPH---hemoprotein reductase] --> rn:R08551

rn:R09925 { Linalool } <-- [Reduced NADPH---hemoprotein reductase] -- rn:R07205 <-- [Oxidized NADPH---hemoprotein reductase] --> rn:R08551

rn:R09934 { Humulene } <-- [Reduced NADPH---hemoprotein reductase] -- rn:R07205 <-- [Oxidized NADPH---hemoprotein reductase] --> rn:R08551

rn:R10562 { (E,E)-Geranyllinalool } <-- [Reduced NADPH---hemoprotein reductase] -- rn:R07205 <-- [Oxidized NADPH---hemoprotein reductase] --> rn:R08551

rn:R11055 { Myrcene } <-- [Reduced NADPH---hemoprotein reductase] -- rn:R07205 <-- [Oxidized NADPH---hemoprotein reductase] --> rn:R08551

rn:R02468 { (-)-Limonene } <-- [Reduced NADPH---hemoprotein reductase] -- rn:R07206 <-- [Oxidized NADPH---hemoprotein reductase] --> rn:R08551

rn:R02469 { (-)-Limonene } <-- [Reduced NADPH---hemoprotein reductase] -- rn:R07206 <-- [Oxidized NADPH---hemoprotein reductase] --> rn:R08551

rn:R02470 { (-)-Limonene } <-- [Reduced NADPH---hemoprotein reductase] -- rn:R07206 <-- [Oxidized NADPH---hemoprotein reductase] --> rn:R08551

rn:R04366 { Linalool } <-- [Reduced NADPH---hemoprotein reductase] -- rn:R07206 <-- [Oxidized NADPH---hemoprotein reductase] --> rn:R08551

rn:R06119 { d-Limonene } <-- [Reduced NADPH---hemoprotein reductase] -- rn:R07206 <-- [Oxidized NADPH---hemoprotein reductase] --> rn:R08551

rn:R09451 { Hexadecanoic acid } <-- [Reduced NADPH---hemoprotein reductase] -- rn:R07206 <-- [Oxidized NADPH---hemoprotein reductase] --> rn:R08551

rn:R09452 { (9Z)-Octadecenoic acid } <-- [Reduced NADPH---hemoprotein reductase] -- rn:R07206 <-- [Oxidized NADPH---hemoprotein reductase] --> rn:R08551

rn:R09922 { (+)-Linalool } <-- [Reduced NADPH---hemoprotein reductase] -- rn:R07206 <-- [Oxidized NADPH---hemoprotein reductase] --> rn:R08551

rn:R09923 { (-)-Linalool } <-- [Reduced NADPH---hemoprotein reductase] -- rn:R07206 <-- [Oxidized NADPH---hemoprotein reductase] --> rn:R08551

rn:R09925 { Linalool } <-- [Reduced NADPH---hemoprotein reductase] -- rn:R07206 <-- [Oxidized NADPH---hemoprotein reductase] --> rn:R08551

rn:R09934 { Humulene } <-- [Reduced NADPH---hemoprotein reductase] -- rn:R07206 <-- [Oxidized NADPH---hemoprotein reductase] --> rn:R08551

rn:R10562 { (E,E)-Geranyllinalool } <-- [Reduced NADPH---hemoprotein reductase] -- rn:R07206 <-- [Oxidized NADPH---hemoprotein reductase] --> rn:R08551

rn:R11055 { Myrcene } <-- [Reduced NADPH---hemoprotein reductase] -- rn:R07206 <-- [Oxidized NADPH---hemoprotein reductase] --> rn:R08551

rn:R05537 { 3-(2-Hydroxyphenyl)propanoate } <-- FAD -- rn:R07220 <-- Cholic acid --> rn:R02792

rn:R05537 { 3-(2-Hydroxyphenyl)propanoate } <-- FAD -- rn:R07220 <-- Cholic acid --> rn:R02793

rn:R05537 { 3-(2-Hydroxyphenyl)propanoate } <-- FAD -- rn:R07220 <-- Cholic acid --> rn:R02794

rn:R05537 { 3-(2-Hydroxyphenyl)propanoate } <-- FAD -- rn:R07220 <-- Cholic acid --> rn:R02798

rn:R05537 { 3-(2-Hydroxyphenyl)propanoate } <-- FAD -- rn:R07220 <-- FADH2 --> rn:R03978

rn:R05537 { 3-(2-Hydroxyphenyl)propanoate } <-- FAD -- rn:R07220 <-- FADH2 --> rn:R05488

rn:R05537 { 3-(2-Hydroxyphenyl)propanoate } <-- FAD -- rn:R07220 <-- Cholic acid --> rn:R07295

rn:R05537 { 3-(2-Hydroxyphenyl)propanoate } <-- FAD -- rn:R07220 <-- FADH2 --> rn:R09517

rn:R05537 { 3-(2-Hydroxyphenyl)propanoate } <-- FAD -- rn:R07220 <-- Cholic acid --> rn:R11539

rn:R05537 { 3-(2-Hydroxyphenyl)propanoate } <-- FAD -- rn:R07220 <-- FADH2 --> rn:R11653

rn:R05537 { 3-(2-Hydroxyphenyl)propanoate } <-- FAD -- rn:R07220 <-- FADH2 --> rn:R12021

rn:R05537 { 3-(2-Hydroxyphenyl)propanoate } <-- FAD -- rn:R07220 <-- FADH2 --> rn:R12023

rn:R05537 { 3-(2-Hydroxyphenyl)propanoate } <-- FAD -- rn:R07220 <-- FADH2 --> rn:R12027

rn:R05537 { 3-(2-Hydroxyphenyl)propanoate } <-- FAD -- rn:R07220 <-- FADH2 --> rn:R12030

rn:R02234 { Cyclohexanone } <-- Acceptor -- rn:R07230 <-- Thyroxine --> rn:R03734

rn:R03212 { 3-Hydroxycyclohexanone } <-- Acceptor -- rn:R07230 <-- Thyroxine --> rn:R03734

rn:R07342 { Phenyl acetate } <-- Phenyl acetate -- rn:R07342 <-- Phenol --> rn:R00815

rn:R07342 { Phenyl acetate } <-- Phenyl acetate -- rn:R07342 <-- Phenol --> rn:R01239

rn:R07342 { Phenyl acetate } <-- Phenyl acetate -- rn:R07342 <-- Phenol --> rn:R01240

rn:R07342 { Phenyl acetate } <-- Phenyl acetate -- rn:R07342 <-- Phenol --> rn:R01241 # rn:R07342 { Phenyl acetate } <-- Phenyl acetate -- rn:R07342 <-- Acetate --> rn:R01241

rn:R07342 { Phenyl acetate } <-- Phenyl acetate -- rn:R07342 <-- Phenol --> rn:R01242

rn:R07342 { Phenyl acetate } <-- Phenyl acetate -- rn:R07342 <-- Acetate --> rn:R01308

rn:R07342 { Phenyl acetate } <-- Phenyl acetate -- rn:R07342 <-- Acetate --> rn:R01426

rn:R07342 { Phenyl acetate } <-- Phenyl acetate -- rn:R07342 <-- Phenol --> rn:R02996

rn:R07342 { Phenyl acetate } <-- Phenyl acetate -- rn:R07342 <-- Acetate --> rn:R05138

rn:R07342 { Phenyl acetate } <-- Phenyl acetate -- rn:R07342 <-- Acetate --> rn:R05219

rn:R07342 { Phenyl acetate } <-- Phenyl acetate -- rn:R07342 <-- Phenol --> rn:R05625

rn:R07342 { Phenyl acetate } <-- Phenyl acetate -- rn:R07342 <-- Acetate --> rn:R09931

rn:R07342 { Phenyl acetate } <-- Phenyl acetate -- rn:R07342 <-- Phenol --> rn:R10043

rn:R07342 { Phenyl acetate } <-- Phenyl acetate -- rn:R07342 <-- Phenol --> rn:R10608

rn:R07342 { Phenyl acetate } <-- Phenyl acetate -- rn:R07342 <-- Phenol --> rn:R11653

rn:R02468 { (-)-Limonene } <-- [Reduced NADPH---hemoprotein reductase] -- rn:R07403 <-- [Oxidized NADPH---hemoprotein reductase] --> rn:R08551

rn:R02469 { (-)-Limonene } <-- [Reduced NADPH---hemoprotein reductase] -- rn:R07403 <-- [Oxidized NADPH---hemoprotein reductase] --> rn:R08551

rn:R02470 { (-)-Limonene } <-- [Reduced NADPH---hemoprotein reductase] -- rn:R07403 <-- [Oxidized NADPH---hemoprotein reductase] --> rn:R08551

rn:R04366 { Linalool } <-- [Reduced NADPH---hemoprotein reductase] -- rn:R07403 <-- [Oxidized NADPH---hemoprotein reductase] --> rn:R08551

rn:R06119 { d-Limonene } <-- [Reduced NADPH---hemoprotein reductase] -- rn:R07403 <-- [Oxidized NADPH---hemoprotein reductase] --> rn:R08551

rn:R09451 { Hexadecanoic acid } <-- [Reduced NADPH---hemoprotein reductase] -- rn:R07403 <-- [Oxidized NADPH---hemoprotein reductase] --> rn:R08551

rn:R09452 { (9Z)-Octadecenoic acid } <-- [Reduced NADPH---hemoprotein reductase] -- rn:R07403 <-- [Oxidized NADPH---hemoprotein reductase] --> rn:R08551

rn:R09922 { (+)-Linalool } <-- [Reduced NADPH---hemoprotein reductase] -- rn:R07403 <-- [Oxidized NADPH---hemoprotein reductase] --> rn:R08551

rn:R09923 { (-)-Linalool } <-- [Reduced NADPH---hemoprotein reductase] -- rn:R07403 <-- [Oxidized NADPH---hemoprotein reductase] --> rn:R08551

rn:R09925 { Linalool } <-- [Reduced NADPH---hemoprotein reductase] -- rn:R07403 <-- [Oxidized NADPH---hemoprotein reductase] --> rn:R08551

rn:R09934 { Humulene } <-- [Reduced NADPH---hemoprotein reductase] -- rn:R07403 <-- [Oxidized NADPH---hemoprotein reductase] --> rn:R08551

rn:R10562 { (E,E)-Geranyllinalool } <-- [Reduced NADPH---hemoprotein reductase] -- rn:R07403 <-- [Oxidized NADPH---hemoprotein reductase] --> rn:R08551

rn:R11055 { Myrcene } <-- [Reduced NADPH---hemoprotein reductase] -- rn:R07403 <-- [Oxidized NADPH---hemoprotein reductase] --> rn:R08551

rn:R11068 { 3-[(1R,2S,5R,6S)-5-Hydroxy-7-oxabicyclo[4.1.0]heptan-2-yl]-2-oxopropanoate } <-- L-Phenylalanine -- rn:R08463 <-- D-Cathinone --> rn:R08464

rn:R11068 { 3-[(1R,2S,5R,6S)-5-Hydroxy-7-oxabicyclo[4.1.0]heptan-2-yl]-2-oxopropanoate } <-- L-Phenylalanine -- rn:R08463 <-- D-Cathinone --> rn:R08466

rn:R02468 { (-)-Limonene } <-- [Reduced NADPH---hemoprotein reductase] -- rn:R08652 <-- (Z)-Phenylacetaldehyde oxime --> rn:R07638

rn:R02469 { (-)-Limonene } <-- [Reduced NADPH---hemoprotein reductase] -- rn:R08652 <-- (Z)-Phenylacetaldehyde oxime --> rn:R07638

rn:R02470 { (-)-Limonene } <-- [Reduced NADPH---hemoprotein reductase] -- rn:R08652 <-- (Z)-Phenylacetaldehyde oxime --> rn:R07638

rn:R04366 { Linalool } <-- [Reduced NADPH---hemoprotein reductase] -- rn:R08652 <-- (Z)-Phenylacetaldehyde oxime --> rn:R07638

rn:R06119 { d-Limonene } <-- [Reduced NADPH---hemoprotein reductase] -- rn:R08652 <-- (Z)-Phenylacetaldehyde oxime --> rn:R07638

rn:R09451 { Hexadecanoic acid } <-- [Reduced NADPH---hemoprotein reductase] -- rn:R08652 <-- (Z)-Phenylacetaldehyde oxime --> rn:R07638

rn:R09452 { (9Z)-Octadecenoic acid } <-- [Reduced NADPH---hemoprotein reductase] -- rn:R08652 <-- (Z)-Phenylacetaldehyde oxime --> rn:R07638

rn:R09922 { (+)-Linalool } <-- [Reduced NADPH---hemoprotein reductase] -- rn:R08652 <-- (Z)-Phenylacetaldehyde oxime --> rn:R07638

rn:R09923 { (-)-Linalool } <-- [Reduced NADPH---hemoprotein reductase] -- rn:R08652 <-- (Z)-Phenylacetaldehyde oxime --> rn:R07638

rn:R09925 { Linalool } <-- [Reduced NADPH---hemoprotein reductase] -- rn:R08652 <-- (Z)-Phenylacetaldehyde oxime --> rn:R07638

rn:R09934 { Humulene } <-- [Reduced NADPH---hemoprotein reductase] -- rn:R08652 <-- (Z)-Phenylacetaldehyde oxime --> rn:R07638

rn:R10562 { (E,E)-Geranyllinalool } <-- [Reduced NADPH---hemoprotein reductase] -- rn:R08652 <-- (Z)-Phenylacetaldehyde oxime --> rn:R07638

rn:R11055 { Myrcene } <-- [Reduced NADPH---hemoprotein reductase] -- rn:R08652 <-- (Z)-Phenylacetaldehyde oxime --> rn:R07638

rn:R11068 { 3-[(1R,2S,5R,6S)-5-Hydroxy-7-oxabicyclo[4.1.0]heptan-2-yl]-2-oxopropanoate } <-- L-Phenylalanine -- rn:R08652 <-- (Z)-Phenylacetaldehyde oxime --> rn:R07638

rn:R02468 { (-)-Limonene } <-- [Reduced NADPH---hemoprotein reductase] -- rn:R08652 <-- (Z)-Phenylacetaldehyde oxime --> rn:R08653

rn:R02469 { (-)-Limonene } <-- [Reduced NADPH---hemoprotein reductase] -- rn:R08652 <-- (Z)-Phenylacetaldehyde oxime --> rn:R08653

rn:R02470 { (-)-Limonene } <-- [Reduced NADPH---hemoprotein reductase] -- rn:R08652 <-- (Z)-Phenylacetaldehyde oxime --> rn:R08653

rn:R04366 { Linalool } <-- [Reduced NADPH---hemoprotein reductase] -- rn:R08652 <-- (Z)-Phenylacetaldehyde oxime --> rn:R08653

rn:R06119 { d-Limonene } <-- [Reduced NADPH---hemoprotein reductase] -- rn:R08652 <-- (Z)-Phenylacetaldehyde oxime --> rn:R08653

rn:R09451 { Hexadecanoic acid } <-- [Reduced NADPH---hemoprotein reductase] -- rn:R08652 <-- (Z)-Phenylacetaldehyde oxime --> rn:R08653

rn:R09452 { (9Z)-Octadecenoic acid } <-- [Reduced NADPH---hemoprotein reductase] -- rn:R08652 <-- (Z)-Phenylacetaldehyde oxime --> rn:R08653

rn:R09922 { (+)-Linalool } <-- [Reduced NADPH---hemoprotein reductase] -- rn:R08652 <-- (Z)-Phenylacetaldehyde oxime --> rn:R08653

rn:R09923 { (-)-Linalool } <-- [Reduced NADPH---hemoprotein reductase] -- rn:R08652 <-- (Z)-Phenylacetaldehyde oxime --> rn:R08653

rn:R09925 { Linalool } <-- [Reduced NADPH---hemoprotein reductase] -- rn:R08652 <-- (Z)-Phenylacetaldehyde oxime --> rn:R08653

rn:R09934 { Humulene } <-- [Reduced NADPH---hemoprotein reductase] -- rn:R08652 <-- (Z)-Phenylacetaldehyde oxime --> rn:R08653

rn:R10562 { (E,E)-Geranyllinalool } <-- [Reduced NADPH---hemoprotein reductase] -- rn:R08652 <-- (Z)-Phenylacetaldehyde oxime --> rn:R08653

rn:R11055 { Myrcene } <-- [Reduced NADPH---hemoprotein reductase] -- rn:R08652 <-- (Z)-Phenylacetaldehyde oxime --> rn:R08653

rn:R11068 { 3-[(1R,2S,5R,6S)-5-Hydroxy-7-oxabicyclo[4.1.0]heptan-2-yl]-2-oxopropanoate } <-- L-Phenylalanine -- rn:R08652 <-- (Z)-Phenylacetaldehyde oxime --> rn:R08653

rn:R02468 { (-)-Limonene } <-- [Reduced NADPH---hemoprotein reductase] -- rn:R08652 <-- (Z)-Phenylacetaldehyde oxime --> rn:R10637

rn:R02469 { (-)-Limonene } <-- [Reduced NADPH---hemoprotein reductase] -- rn:R08652 <-- (Z)-Phenylacetaldehyde oxime --> rn:R10637

rn:R02470 { (-)-Limonene } <-- [Reduced NADPH---hemoprotein reductase] -- rn:R08652 <-- (Z)-Phenylacetaldehyde oxime --> rn:R10637

rn:R04366 { Linalool } <-- [Reduced NADPH---hemoprotein reductase] -- rn:R08652 <-- (Z)-Phenylacetaldehyde oxime --> rn:R10637

rn:R06119 { d-Limonene } <-- [Reduced NADPH---hemoprotein reductase] -- rn:R08652 <-- (Z)-Phenylacetaldehyde oxime --> rn:R10637

rn:R09451 { Hexadecanoic acid } <-- [Reduced NADPH---hemoprotein reductase] -- rn:R08652 <-- (Z)-Phenylacetaldehyde oxime --> rn:R10637

rn:R09452 { (9Z)-Octadecenoic acid } <-- [Reduced NADPH---hemoprotein reductase] -- rn:R08652 <-- (Z)-Phenylacetaldehyde oxime --> rn:R10637

rn:R09922 { (+)-Linalool } <-- [Reduced NADPH---hemoprotein reductase] -- rn:R08652 <-- (Z)-Phenylacetaldehyde oxime --> rn:R10637

rn:R09923 { (-)-Linalool } <-- [Reduced NADPH---hemoprotein reductase] -- rn:R08652 <-- (Z)-Phenylacetaldehyde oxime --> rn:R10637

rn:R09925 { Linalool } <-- [Reduced NADPH---hemoprotein reductase] -- rn:R08652 <-- (Z)-Phenylacetaldehyde oxime --> rn:R10637

rn:R09934 { Humulene } <-- [Reduced NADPH---hemoprotein reductase] -- rn:R08652 <-- (Z)-Phenylacetaldehyde oxime --> rn:R10637

rn:R10562 { (E,E)-Geranyllinalool } <-- [Reduced NADPH---hemoprotein reductase] -- rn:R08652 <-- (Z)-Phenylacetaldehyde oxime --> rn:R10637

rn:R11055 { Myrcene } <-- [Reduced NADPH---hemoprotein reductase] -- rn:R08652 <-- (Z)-Phenylacetaldehyde oxime --> rn:R10637

rn:R11068 { 3-[(1R,2S,5R,6S)-5-Hydroxy-7-oxabicyclo[4.1.0]heptan-2-yl]-2-oxopropanoate } <-- L-Phenylalanine -- rn:R08652 <-- (Z)-Phenylacetaldehyde oxime --> rn:R10637

rn:R02468 { (-)-Limonene } <-- [Reduced NADPH---hemoprotein reductase] -- rn:R08663 <-- (E)-2-Methylpropanal oxime --> rn:R04169

rn:R02469 { (-)-Limonene } <-- [Reduced NADPH---hemoprotein reductase] -- rn:R08663 <-- (E)-2-Methylpropanal oxime --> rn:R04169

rn:R02470 { (-)-Limonene } <-- [Reduced NADPH---hemoprotein reductase] -- rn:R08663 <-- (E)-2-Methylpropanal oxime --> rn:R04169

rn:R04366 { Linalool } <-- [Reduced NADPH---hemoprotein reductase] -- rn:R08663 <-- (E)-2-Methylpropanal oxime --> rn:R04169

rn:R06119 { d-Limonene } <-- [Reduced NADPH---hemoprotein reductase] -- rn:R08663 <-- (E)-2-Methylpropanal oxime --> rn:R04169

rn:R09451 { Hexadecanoic acid } <-- [Reduced NADPH---hemoprotein reductase] -- rn:R08663 <-- (E)-2-Methylpropanal oxime --> rn:R04169

rn:R09452 { (9Z)-Octadecenoic acid } <-- [Reduced NADPH---hemoprotein reductase] -- rn:R08663 <-- (E)-2-Methylpropanal oxime --> rn:R04169

rn:R09922 { (+)-Linalool } <-- [Reduced NADPH---hemoprotein reductase] -- rn:R08663 <-- (E)-2-Methylpropanal oxime --> rn:R04169

rn:R09923 { (-)-Linalool } <-- [Reduced NADPH---hemoprotein reductase] -- rn:R08663 <-- (E)-2-Methylpropanal oxime --> rn:R04169

rn:R09925 { Linalool } <-- [Reduced NADPH---hemoprotein reductase] -- rn:R08663 <-- (E)-2-Methylpropanal oxime --> rn:R04169

rn:R09934 { Humulene } <-- [Reduced NADPH---hemoprotein reductase] -- rn:R08663 <-- (E)-2-Methylpropanal oxime --> rn:R04169

rn:R10562 { (E,E)-Geranyllinalool } <-- [Reduced NADPH---hemoprotein reductase] -- rn:R08663 <-- (E)-2-Methylpropanal oxime --> rn:R04169

rn:R11055 { Myrcene } <-- [Reduced NADPH---hemoprotein reductase] -- rn:R08663 <-- (E)-2-Methylpropanal oxime --> rn:R04169

rn:R02468 { (-)-Limonene } <-- [Reduced NADPH---hemoprotein reductase] -- rn:R08663 <-- [Oxidized NADPH---hemoprotein reductase] --> rn:R08551

rn:R02469 { (-)-Limonene } <-- [Reduced NADPH---hemoprotein reductase] -- rn:R08663 <-- [Oxidized NADPH---hemoprotein reductase] --> rn:R08551

rn:R02470 { (-)-Limonene } <-- [Reduced NADPH---hemoprotein reductase] -- rn:R08663 <-- [Oxidized NADPH---hemoprotein reductase] --> rn:R08551

rn:R04366 { Linalool } <-- [Reduced NADPH---hemoprotein reductase] -- rn:R08663 <-- [Oxidized NADPH---hemoprotein reductase] --> rn:R08551

rn:R06119 { d-Limonene } <-- [Reduced NADPH---hemoprotein reductase] -- rn:R08663 <-- [Oxidized NADPH---hemoprotein reductase] --> rn:R08551

rn:R09451 { Hexadecanoic acid } <-- [Reduced NADPH---hemoprotein reductase] -- rn:R08663 <-- [Oxidized NADPH---hemoprotein reductase] --> rn:R08551

rn:R09452 { (9Z)-Octadecenoic acid } <-- [Reduced NADPH---hemoprotein reductase] -- rn:R08663 <-- [Oxidized NADPH---hemoprotein reductase] --> rn:R08551

rn:R09922 { (+)-Linalool } <-- [Reduced NADPH---hemoprotein reductase] -- rn:R08663 <-- [Oxidized NADPH---hemoprotein reductase] --> rn:R08551

rn:R09923 { (-)-Linalool } <-- [Reduced NADPH---hemoprotein reductase] -- rn:R08663 <-- [Oxidized NADPH---hemoprotein reductase] --> rn:R08551

rn:R09925 { Linalool } <-- [Reduced NADPH---hemoprotein reductase] -- rn:R08663 <-- [Oxidized NADPH---hemoprotein reductase] --> rn:R08551

rn:R09934 { Humulene } <-- [Reduced NADPH---hemoprotein reductase] -- rn:R08663 <-- [Oxidized NADPH---hemoprotein reductase] --> rn:R08551

rn:R10562 { (E,E)-Geranyllinalool } <-- [Reduced NADPH---hemoprotein reductase] -- rn:R08663 <-- [Oxidized NADPH---hemoprotein reductase] --> rn:R08551

rn:R11055 { Myrcene } <-- [Reduced NADPH---hemoprotein reductase] -- rn:R08663 <-- [Oxidized NADPH---hemoprotein reductase] --> rn:R08551

rn:R02468 { (-)-Limonene } <-- [Reduced NADPH---hemoprotein reductase] -- rn:R08663 <-- (E)-2-Methylpropanal oxime --> rn:R10034

rn:R02469 { (-)-Limonene } <-- [Reduced NADPH---hemoprotein reductase] -- rn:R08663 <-- (E)-2-Methylpropanal oxime --> rn:R10034

rn:R02470 { (-)-Limonene } <-- [Reduced NADPH---hemoprotein reductase] -- rn:R08663 <-- (E)-2-Methylpropanal oxime --> rn:R10034

rn:R04366 { Linalool } <-- [Reduced NADPH---hemoprotein reductase] -- rn:R08663 <-- (E)-2-Methylpropanal oxime --> rn:R10034

rn:R06119 { d-Limonene } <-- [Reduced NADPH---hemoprotein reductase] -- rn:R08663 <-- (E)-2-Methylpropanal oxime --> rn:R10034

rn:R09451 { Hexadecanoic acid } <-- [Reduced NADPH---hemoprotein reductase] -- rn:R08663 <-- (E)-2-Methylpropanal oxime --> rn:R10034

rn:R09452 { (9Z)-Octadecenoic acid } <-- [Reduced NADPH---hemoprotein reductase] -- rn:R08663 <-- (E)-2-Methylpropanal oxime --> rn:R10034

rn:R09922 { (+)-Linalool } <-- [Reduced NADPH---hemoprotein reductase] -- rn:R08663 <-- (E)-2-Methylpropanal oxime --> rn:R10034

rn:R09923 { (-)-Linalool } <-- [Reduced NADPH---hemoprotein reductase] -- rn:R08663 <-- (E)-2-Methylpropanal oxime --> rn:R10034

rn:R09925 { Linalool } <-- [Reduced NADPH---hemoprotein reductase] -- rn:R08663 <-- (E)-2-Methylpropanal oxime --> rn:R10034

rn:R09934 { Humulene } <-- [Reduced NADPH---hemoprotein reductase] -- rn:R08663 <-- (E)-2-Methylpropanal oxime --> rn:R10034

rn:R10562 { (E,E)-Geranyllinalool } <-- [Reduced NADPH---hemoprotein reductase] -- rn:R08663 <-- (E)-2-Methylpropanal oxime --> rn:R10034

rn:R11055 { Myrcene } <-- [Reduced NADPH---hemoprotein reductase] -- rn:R08663 <-- (E)-2-Methylpropanal oxime --> rn:R10034

rn:R02468 { (-)-Limonene } <-- [Reduced NADPH---hemoprotein reductase] -- rn:R08663 <-- (E)-2-Methylpropanal oxime --> rn:R11598

rn:R02469 { (-)-Limonene } <-- [Reduced NADPH---hemoprotein reductase] -- rn:R08663 <-- (E)-2-Methylpropanal oxime --> rn:R11598

rn:R02470 { (-)-Limonene } <-- [Reduced NADPH---hemoprotein reductase] -- rn:R08663 <-- (E)-2-Methylpropanal oxime --> rn:R11598

rn:R04366 { Linalool } <-- [Reduced NADPH---hemoprotein reductase] -- rn:R08663 <-- (E)-2-Methylpropanal oxime --> rn:R11598

rn:R06119 { d-Limonene } <-- [Reduced NADPH---hemoprotein reductase] -- rn:R08663 <-- (E)-2-Methylpropanal oxime --> rn:R11598

rn:R09451 { Hexadecanoic acid } <-- [Reduced NADPH---hemoprotein reductase] -- rn:R08663 <-- (E)-2-Methylpropanal oxime --> rn:R11598

rn:R09452 { (9Z)-Octadecenoic acid } <-- [Reduced NADPH---hemoprotein reductase] -- rn:R08663 <-- (E)-2-Methylpropanal oxime --> rn:R11598

rn:R09922 { (+)-Linalool } <-- [Reduced NADPH---hemoprotein reductase] -- rn:R08663 <-- (E)-2-Methylpropanal oxime --> rn:R11598

rn:R09923 { (-)-Linalool } <-- [Reduced NADPH---hemoprotein reductase] -- rn:R08663 <-- (E)-2-Methylpropanal oxime --> rn:R11598

rn:R09925 { Linalool } <-- [Reduced NADPH---hemoprotein reductase] -- rn:R08663 <-- (E)-2-Methylpropanal oxime --> rn:R11598

rn:R09934 { Humulene } <-- [Reduced NADPH---hemoprotein reductase] -- rn:R08663 <-- (E)-2-Methylpropanal oxime --> rn:R11598

rn:R10562 { (E,E)-Geranyllinalool } <-- [Reduced NADPH---hemoprotein reductase] -- rn:R08663 <-- (E)-2-Methylpropanal oxime --> rn:R11598

rn:R11055 { Myrcene } <-- [Reduced NADPH---hemoprotein reductase] -- rn:R08663 <-- (E)-2-Methylpropanal oxime --> rn:R11598

rn:R11672 { (S)-3-Acetyloctanal } <-- Amino acid -- rn:R08684 <-- Aldoxime --> rn:R02827

rn:R11672 { (S)-3-Acetyloctanal } <-- Amino acid -- rn:R08684 <-- Aldoxime --> rn:R08685

rn:R11068 { 3-[(1R,2S,5R,6S)-5-Hydroxy-7-oxabicyclo[4.1.0]heptan-2-yl]-2-oxopropanoate } <-- L-Phenylalanine -- rn:R08690 <-- L-Homophenylalanine --> rn:R08655

rn:R06404 { alpha-Pinene } <-- e- -- rn:R08862 <-- Cob(II)alamin --> rn:R00097

rn:R06404 { alpha-Pinene } <-- e- -- rn:R08862 <-- (S)-2-Hydroxypropylphosphonate --> rn:R08863

rn:R06404 { alpha-Pinene } <-- e- -- rn:R08862 <-- L-Methionine --> rn:R12055

rn:R06404 { alpha-Pinene } <-- e- -- rn:R08862 <-- Cob(II)alamin --> rn:R12183

rn:R08530 { (-)-Menthol } <-- Acetyl-CoA -- rn:R08871 <-- N-Acetyldemethylphosphinothricin --> rn:R08872

rn:R08531 { (+)-Neomenthol } <-- Acetyl-CoA -- rn:R08871 <-- N-Acetyldemethylphosphinothricin --> rn:R08872

rn:R08532 { (+)-Borneol } <-- Acetyl-CoA -- rn:R08871 <-- N-Acetyldemethylphosphinothricin --> rn:R08872

rn:R10474 { Cinnamyl alcohol } <-- Acetyl-CoA -- rn:R08871 <-- N-Acetyldemethylphosphinothricin --> rn:R08872

rn:R08530 { (-)-Menthol } <-- Acetyl-CoA -- rn:R08871 <-- N-Acetyldemethylphosphinothricin --> rn:R08873

rn:R08531 { (+)-Neomenthol } <-- Acetyl-CoA -- rn:R08871 <-- N-Acetyldemethylphosphinothricin --> rn:R08873

rn:R08532 { (+)-Borneol } <-- Acetyl-CoA -- rn:R08871 <-- N-Acetyldemethylphosphinothricin --> rn:R08873

rn:R10474 { Cinnamyl alcohol } <-- Acetyl-CoA -- rn:R08871 <-- N-Acetyldemethylphosphinothricin --> rn:R08873

rn:R06404 { alpha-Pinene } <-- e- -- rn:R08873 <-- Cob(II)alamin --> rn:R00097

rn:R06404 { alpha-Pinene } <-- e- -- rn:R08873 <-- N-Acetylphosphinothricin --> rn:R08874

rn:R06404 { alpha-Pinene } <-- e- -- rn:R08873 <-- L-Methionine --> rn:R12055

rn:R06404 { alpha-Pinene } <-- e- -- rn:R08873 <-- Cob(II)alamin --> rn:R12183

rn:R06404 { alpha-Pinene } <-- e- -- rn:R08875 <-- Cob(II)alamin --> rn:R00097

rn:R06404 { alpha-Pinene } <-- e- -- rn:R08875 <-- N-Acetylbialaphos --> rn:R08876

rn:R06404 { alpha-Pinene } <-- e- -- rn:R08875 <-- L-Methionine --> rn:R12055

rn:R06404 { alpha-Pinene } <-- e- -- rn:R08875 <-- Cob(II)alamin --> rn:R12183

rn:R08530 { (-)-Menthol } <-- Acetyl-CoA -- rn:R08938 <-- N-Acetylphosphinothricin --> rn:R08874

rn:R08531 { (+)-Neomenthol } <-- Acetyl-CoA -- rn:R08938 <-- N-Acetylphosphinothricin --> rn:R08874

rn:R08532 { (+)-Borneol } <-- Acetyl-CoA -- rn:R08938 <-- N-Acetylphosphinothricin --> rn:R08874

rn:R10474 { Cinnamyl alcohol } <-- Acetyl-CoA -- rn:R08938 <-- N-Acetylphosphinothricin --> rn:R08874

rn:R05136 { Salicylaldehyde } <-- Pyruvate -- rn:R09088 <-- Pyruvophenone --> rn:R09089

rn:R02468 { (-)-Limonene } <-- [Reduced NADPH---hemoprotein reductase] -- rn:R09403 <-- [Oxidized NADPH---hemoprotein reductase] --> rn:R08551

rn:R02469 { (-)-Limonene } <-- [Reduced NADPH---hemoprotein reductase] -- rn:R09403 <-- [Oxidized NADPH---hemoprotein reductase] --> rn:R08551

rn:R02470 { (-)-Limonene } <-- [Reduced NADPH---hemoprotein reductase] -- rn:R09403 <-- [Oxidized NADPH---hemoprotein reductase] --> rn:R08551

rn:R04366 { Linalool } <-- [Reduced NADPH---hemoprotein reductase] -- rn:R09403 <-- [Oxidized NADPH---hemoprotein reductase] --> rn:R08551

rn:R06119 { d-Limonene } <-- [Reduced NADPH---hemoprotein reductase] -- rn:R09403 <-- [Oxidized NADPH---hemoprotein reductase] --> rn:R08551

rn:R09451 { Hexadecanoic acid } <-- [Reduced NADPH---hemoprotein reductase] -- rn:R09403 <-- [Oxidized NADPH---hemoprotein reductase] --> rn:R08551

rn:R09452 { (9Z)-Octadecenoic acid } <-- [Reduced NADPH---hemoprotein reductase] -- rn:R09403 <-- [Oxidized NADPH---hemoprotein reductase] --> rn:R08551

rn:R09922 { (+)-Linalool } <-- [Reduced NADPH---hemoprotein reductase] -- rn:R09403 <-- [Oxidized NADPH---hemoprotein reductase] --> rn:R08551

rn:R09923 { (-)-Linalool } <-- [Reduced NADPH---hemoprotein reductase] -- rn:R09403 <-- [Oxidized NADPH---hemoprotein reductase] --> rn:R08551

rn:R09925 { Linalool } <-- [Reduced NADPH---hemoprotein reductase] -- rn:R09403 <-- [Oxidized NADPH---hemoprotein reductase] --> rn:R08551

rn:R09934 { Humulene } <-- [Reduced NADPH---hemoprotein reductase] -- rn:R09403 <-- [Oxidized NADPH---hemoprotein reductase] --> rn:R08551

rn:R10562 { (E,E)-Geranyllinalool } <-- [Reduced NADPH---hemoprotein reductase] -- rn:R09403 <-- [Oxidized NADPH---hemoprotein reductase] --> rn:R08551

rn:R11055 { Myrcene } <-- [Reduced NADPH---hemoprotein reductase] -- rn:R09403 <-- [Oxidized NADPH---hemoprotein reductase] --> rn:R08551

rn:R02468 { (-)-Limonene } <-- [Reduced NADPH---hemoprotein reductase] -- rn:R09403 <-- (E)-2-Methylbutanal oxime --> rn:R10030

rn:R02469 { (-)-Limonene } <-- [Reduced NADPH---hemoprotein reductase] -- rn:R09403 <-- (E)-2-Methylbutanal oxime --> rn:R10030

rn:R02470 { (-)-Limonene } <-- [Reduced NADPH---hemoprotein reductase] -- rn:R09403 <-- (E)-2-Methylbutanal oxime --> rn:R10030

rn:R04366 { Linalool } <-- [Reduced NADPH---hemoprotein reductase] -- rn:R09403 <-- (E)-2-Methylbutanal oxime --> rn:R10030

rn:R06119 { d-Limonene } <-- [Reduced NADPH---hemoprotein reductase] -- rn:R09403 <-- (E)-2-Methylbutanal oxime --> rn:R10030

rn:R09451 { Hexadecanoic acid } <-- [Reduced NADPH---hemoprotein reductase] -- rn:R09403 <-- (E)-2-Methylbutanal oxime --> rn:R10030

rn:R09452 { (9Z)-Octadecenoic acid } <-- [Reduced NADPH---hemoprotein reductase] -- rn:R09403 <-- (E)-2-Methylbutanal oxime --> rn:R10030

rn:R09922 { (+)-Linalool } <-- [Reduced NADPH---hemoprotein reductase] -- rn:R09403 <-- (E)-2-Methylbutanal oxime --> rn:R10030

rn:R09923 { (-)-Linalool } <-- [Reduced NADPH---hemoprotein reductase] -- rn:R09403 <-- (E)-2-Methylbutanal oxime --> rn:R10030

rn:R09925 { Linalool } <-- [Reduced NADPH---hemoprotein reductase] -- rn:R09403 <-- (E)-2-Methylbutanal oxime --> rn:R10030

rn:R09934 { Humulene } <-- [Reduced NADPH---hemoprotein reductase] -- rn:R09403 <-- (E)-2-Methylbutanal oxime --> rn:R10030

rn:R10562 { (E,E)-Geranyllinalool } <-- [Reduced NADPH---hemoprotein reductase] -- rn:R09403 <-- (E)-2-Methylbutanal oxime --> rn:R10030

rn:R11055 { Myrcene } <-- [Reduced NADPH---hemoprotein reductase] -- rn:R09403 <-- (E)-2-Methylbutanal oxime --> rn:R10030

rn:R02468 { (-)-Limonene } <-- [Reduced NADPH---hemoprotein reductase] -- rn:R09403 <-- (E)-2-Methylbutanal oxime --> rn:R11597

rn:R02469 { (-)-Limonene } <-- [Reduced NADPH---hemoprotein reductase] -- rn:R09403 <-- (E)-2-Methylbutanal oxime --> rn:R11597

rn:R02470 { (-)-Limonene } <-- [Reduced NADPH---hemoprotein reductase] -- rn:R09403 <-- (E)-2-Methylbutanal oxime --> rn:R11597

rn:R04366 { Linalool } <-- [Reduced NADPH---hemoprotein reductase] -- rn:R09403 <-- (E)-2-Methylbutanal oxime --> rn:R11597

rn:R06119 { d-Limonene } <-- [Reduced NADPH---hemoprotein reductase] -- rn:R09403 <-- (E)-2-Methylbutanal oxime --> rn:R11597

rn:R09451 { Hexadecanoic acid } <-- [Reduced NADPH---hemoprotein reductase] -- rn:R09403 <-- (E)-2-Methylbutanal oxime --> rn:R11597

rn:R09452 { (9Z)-Octadecenoic acid } <-- [Reduced NADPH---hemoprotein reductase] -- rn:R09403 <-- (E)-2-Methylbutanal oxime --> rn:R11597

rn:R09922 { (+)-Linalool } <-- [Reduced NADPH---hemoprotein reductase] -- rn:R09403 <-- (E)-2-Methylbutanal oxime --> rn:R11597

rn:R09923 { (-)-Linalool } <-- [Reduced NADPH---hemoprotein reductase] -- rn:R09403 <-- (E)-2-Methylbutanal oxime --> rn:R11597

rn:R09925 { Linalool } <-- [Reduced NADPH---hemoprotein reductase] -- rn:R09403 <-- (E)-2-Methylbutanal oxime --> rn:R11597

rn:R09934 { Humulene } <-- [Reduced NADPH---hemoprotein reductase] -- rn:R09403 <-- (E)-2-Methylbutanal oxime --> rn:R11597

rn:R10562 { (E,E)-Geranyllinalool } <-- [Reduced NADPH---hemoprotein reductase] -- rn:R09403 <-- (E)-2-Methylbutanal oxime --> rn:R11597

rn:R11055 { Myrcene } <-- [Reduced NADPH---hemoprotein reductase] -- rn:R09403 <-- (E)-2-Methylbutanal oxime --> rn:R11597

rn:R05488 { Styrene } <-- FADH2 -- rn:R09517 <-- FAD --> rn:R02487

rn:R05488 { Styrene } <-- FADH2 -- rn:R09517 <-- 3-Hydroxyanthranilate --> rn:R02665

rn:R05488 { Styrene } <-- FADH2 -- rn:R09517 <-- 3-Hydroxyanthranilate --> rn:R02666

rn:R05488 { Styrene } <-- FADH2 -- rn:R09517 <-- 3-Hydroxyanthranilate --> rn:R02667

rn:R05488 { Styrene } <-- FADH2 -- rn:R09517 <-- 3-Hydroxyanthranilate --> rn:R02669

rn:R05488 { Styrene } <-- FADH2 -- rn:R09517 <-- 3-Hydroxyanthranilate --> rn:R02670

rn:R05488 { Styrene } <-- FADH2 -- rn:R09517 <-- 3-Hydroxyanthranilate --> rn:R02671

rn:R05488 { Styrene } <-- FADH2 -- rn:R09517 <-- FAD --> rn:R04095

rn:R05488 { Styrene } <-- FADH2 -- rn:R09517 <-- FAD --> rn:R05537

rn:R05488 { Styrene } <-- FADH2 -- rn:R09517 <-- FAD --> rn:R06943

rn:R05488 { Styrene } <-- FADH2 -- rn:R09517 <-- FAD --> rn:R07220

rn:R05488 { Styrene } <-- FADH2 -- rn:R09517 <-- FAD --> rn:R09520

rn:R05488 { Styrene } <-- FADH2 -- rn:R09517 <-- FAD --> rn:R11130

rn:R05537 { 3-(2-Hydroxyphenyl)propanoate } <-- FAD -- rn:R09520 <-- FADH2 --> rn:R03978

rn:R05537 { 3-(2-Hydroxyphenyl)propanoate } <-- FAD -- rn:R09520 <-- FADH2 --> rn:R05488

rn:R05537 { 3-(2-Hydroxyphenyl)propanoate } <-- FAD -- rn:R09520 <-- FADH2 --> rn:R09517

rn:R05537 { 3-(2-Hydroxyphenyl)propanoate } <-- FAD -- rn:R09520 <-- FADH2 --> rn:R11653

rn:R05537 { 3-(2-Hydroxyphenyl)propanoate } <-- FAD -- rn:R09520 <-- FADH2 --> rn:R12021

rn:R05537 { 3-(2-Hydroxyphenyl)propanoate } <-- FAD -- rn:R09520 <-- FADH2 --> rn:R12023

rn:R05537 { 3-(2-Hydroxyphenyl)propanoate } <-- FAD -- rn:R09520 <-- FADH2 --> rn:R12027

rn:R05537 { 3-(2-Hydroxyphenyl)propanoate } <-- FAD -- rn:R09520 <-- FADH2 --> rn:R12030

rn:R02468 { (-)-Limonene } <-- [Reduced NADPH---hemoprotein reductase] -- rn:R09578 <-- [Oxidized NADPH---hemoprotein reductase] --> rn:R08551

rn:R02469 { (-)-Limonene } <-- [Reduced NADPH---hemoprotein reductase] -- rn:R09578 <-- [Oxidized NADPH---hemoprotein reductase] --> rn:R08551

rn:R02470 { (-)-Limonene } <-- [Reduced NADPH---hemoprotein reductase] -- rn:R09578 <-- [Oxidized NADPH---hemoprotein reductase] --> rn:R08551

rn:R04366 { Linalool } <-- [Reduced NADPH---hemoprotein reductase] -- rn:R09578 <-- [Oxidized NADPH---hemoprotein reductase] --> rn:R08551

rn:R06119 { d-Limonene } <-- [Reduced NADPH---hemoprotein reductase] -- rn:R09578 <-- [Oxidized NADPH---hemoprotein reductase] --> rn:R08551

rn:R09451 { Hexadecanoic acid } <-- [Reduced NADPH---hemoprotein reductase] -- rn:R09578 <-- [Oxidized NADPH---hemoprotein reductase] --> rn:R08551

rn:R09452 { (9Z)-Octadecenoic acid } <-- [Reduced NADPH---hemoprotein reductase] -- rn:R09578 <-- [Oxidized NADPH---hemoprotein reductase] --> rn:R08551

rn:R09922 { (+)-Linalool } <-- [Reduced NADPH---hemoprotein reductase] -- rn:R09578 <-- [Oxidized NADPH---hemoprotein reductase] --> rn:R08551

rn:R09923 { (-)-Linalool } <-- [Reduced NADPH---hemoprotein reductase] -- rn:R09578 <-- [Oxidized NADPH---hemoprotein reductase] --> rn:R08551

rn:R09925 { Linalool } <-- [Reduced NADPH---hemoprotein reductase] -- rn:R09578 <-- [Oxidized NADPH---hemoprotein reductase] --> rn:R08551

rn:R09934 { Humulene } <-- [Reduced NADPH---hemoprotein reductase] -- rn:R09578 <-- [Oxidized NADPH---hemoprotein reductase] --> rn:R08551

rn:R10562 { (E,E)-Geranyllinalool } <-- [Reduced NADPH---hemoprotein reductase] -- rn:R09578 <-- [Oxidized NADPH---hemoprotein reductase] --> rn:R08551

rn:R11055 { Myrcene } <-- [Reduced NADPH---hemoprotein reductase] -- rn:R09578 <-- [Oxidized NADPH---hemoprotein reductase] --> rn:R08551

rn:R11068 { 3-[(1R,2S,5R,6S)-5-Hydroxy-7-oxabicyclo[4.1.0]heptan-2-yl]-2-oxopropanoate } <-- L-Phenylalanine -- rn:R09578 <-- [Oxidized NADPH---hemoprotein reductase] --> rn:R08551

rn:R02468 { (-)-Limonene } <-- [Reduced NADPH---hemoprotein reductase] -- rn:R09578 <-- (E)-Phenylacetaldoxime --> rn:R10041

rn:R02469 { (-)-Limonene } <-- [Reduced NADPH---hemoprotein reductase] -- rn:R09578 <-- (E)-Phenylacetaldoxime --> rn:R10041

rn:R02470 { (-)-Limonene } <-- [Reduced NADPH---hemoprotein reductase] -- rn:R09578 <-- (E)-Phenylacetaldoxime --> rn:R10041

rn:R04366 { Linalool } <-- [Reduced NADPH---hemoprotein reductase] -- rn:R09578 <-- (E)-Phenylacetaldoxime --> rn:R10041

rn:R06119 { d-Limonene } <-- [Reduced NADPH---hemoprotein reductase] -- rn:R09578 <-- (E)-Phenylacetaldoxime --> rn:R10041

rn:R09451 { Hexadecanoic acid } <-- [Reduced NADPH---hemoprotein reductase] -- rn:R09578 <-- (E)-Phenylacetaldoxime --> rn:R10041

rn:R09452 { (9Z)-Octadecenoic acid } <-- [Reduced NADPH---hemoprotein reductase] -- rn:R09578 <-- (E)-Phenylacetaldoxime --> rn:R10041

rn:R09922 { (+)-Linalool } <-- [Reduced NADPH---hemoprotein reductase] -- rn:R09578 <-- (E)-Phenylacetaldoxime --> rn:R10041

rn:R09923 { (-)-Linalool } <-- [Reduced NADPH---hemoprotein reductase] -- rn:R09578 <-- (E)-Phenylacetaldoxime --> rn:R10041

rn:R09925 { Linalool } <-- [Reduced NADPH---hemoprotein reductase] -- rn:R09578 <-- (E)-Phenylacetaldoxime --> rn:R10041

rn:R09934 { Humulene } <-- [Reduced NADPH---hemoprotein reductase] -- rn:R09578 <-- (E)-Phenylacetaldoxime --> rn:R10041

rn:R10562 { (E,E)-Geranyllinalool } <-- [Reduced NADPH---hemoprotein reductase] -- rn:R09578 <-- (E)-Phenylacetaldoxime --> rn:R10041

rn:R11055 { Myrcene } <-- [Reduced NADPH---hemoprotein reductase] -- rn:R09578 <-- (E)-Phenylacetaldoxime --> rn:R10041

rn:R11068 { 3-[(1R,2S,5R,6S)-5-Hydroxy-7-oxabicyclo[4.1.0]heptan-2-yl]-2-oxopropanoate } <-- L-Phenylalanine -- rn:R09578 <-- (E)-Phenylacetaldoxime --> rn:R10041

rn:R02468 { (-)-Limonene } <-- [Reduced NADPH---hemoprotein reductase] -- rn:R09578 <-- (E)-Phenylacetaldoxime --> rn:R10670

rn:R02469 { (-)-Limonene } <-- [Reduced NADPH---hemoprotein reductase] -- rn:R09578 <-- (E)-Phenylacetaldoxime --> rn:R10670

rn:R02470 { (-)-Limonene } <-- [Reduced NADPH---hemoprotein reductase] -- rn:R09578 <-- (E)-Phenylacetaldoxime --> rn:R10670

rn:R04366 { Linalool } <-- [Reduced NADPH---hemoprotein reductase] -- rn:R09578 <-- (E)-Phenylacetaldoxime --> rn:R10670

rn:R06119 { d-Limonene } <-- [Reduced NADPH---hemoprotein reductase] -- rn:R09578 <-- (E)-Phenylacetaldoxime --> rn:R10670

rn:R09451 { Hexadecanoic acid } <-- [Reduced NADPH---hemoprotein reductase] -- rn:R09578 <-- (E)-Phenylacetaldoxime --> rn:R10670

rn:R09452 { (9Z)-Octadecenoic acid } <-- [Reduced NADPH---hemoprotein reductase] -- rn:R09578 <-- (E)-Phenylacetaldoxime --> rn:R10670

rn:R09922 { (+)-Linalool } <-- [Reduced NADPH---hemoprotein reductase] -- rn:R09578 <-- (E)-Phenylacetaldoxime --> rn:R10670

rn:R09923 { (-)-Linalool } <-- [Reduced NADPH---hemoprotein reductase] -- rn:R09578 <-- (E)-Phenylacetaldoxime --> rn:R10670

rn:R09925 { Linalool } <-- [Reduced NADPH---hemoprotein reductase] -- rn:R09578 <-- (E)-Phenylacetaldoxime --> rn:R10670

rn:R09934 { Humulene } <-- [Reduced NADPH---hemoprotein reductase] -- rn:R09578 <-- (E)-Phenylacetaldoxime --> rn:R10670

rn:R10562 { (E,E)-Geranyllinalool } <-- [Reduced NADPH---hemoprotein reductase] -- rn:R09578 <-- (E)-Phenylacetaldoxime --> rn:R10670

rn:R11055 { Myrcene } <-- [Reduced NADPH---hemoprotein reductase] -- rn:R09578 <-- (E)-Phenylacetaldoxime --> rn:R10670

rn:R11068 { 3-[(1R,2S,5R,6S)-5-Hydroxy-7-oxabicyclo[4.1.0]heptan-2-yl]-2-oxopropanoate } <-- L-Phenylalanine -- rn:R09578 <-- (E)-Phenylacetaldoxime --> rn:R10670

rn:R02468 { (-)-Limonene } <-- [Reduced NADPH---hemoprotein reductase] -- rn:R09578 <-- (E)-Phenylacetaldoxime --> rn:R11732

rn:R02469 { (-)-Limonene } <-- [Reduced NADPH---hemoprotein reductase] -- rn:R09578 <-- (E)-Phenylacetaldoxime --> rn:R11732

rn:R02470 { (-)-Limonene } <-- [Reduced NADPH---hemoprotein reductase] -- rn:R09578 <-- (E)-Phenylacetaldoxime --> rn:R11732

rn:R04366 { Linalool } <-- [Reduced NADPH---hemoprotein reductase] -- rn:R09578 <-- (E)-Phenylacetaldoxime --> rn:R11732

rn:R06119 { d-Limonene } <-- [Reduced NADPH---hemoprotein reductase] -- rn:R09578 <-- (E)-Phenylacetaldoxime --> rn:R11732

rn:R09451 { Hexadecanoic acid } <-- [Reduced NADPH---hemoprotein reductase] -- rn:R09578 <-- (E)-Phenylacetaldoxime --> rn:R11732

rn:R09452 { (9Z)-Octadecenoic acid } <-- [Reduced NADPH---hemoprotein reductase] -- rn:R09578 <-- (E)-Phenylacetaldoxime --> rn:R11732

rn:R09922 { (+)-Linalool } <-- [Reduced NADPH---hemoprotein reductase] -- rn:R09578 <-- (E)-Phenylacetaldoxime --> rn:R11732

rn:R09923 { (-)-Linalool } <-- [Reduced NADPH---hemoprotein reductase] -- rn:R09578 <-- (E)-Phenylacetaldoxime --> rn:R11732

rn:R09925 { Linalool } <-- [Reduced NADPH---hemoprotein reductase] -- rn:R09578 <-- (E)-Phenylacetaldoxime --> rn:R11732

rn:R09934 { Humulene } <-- [Reduced NADPH---hemoprotein reductase] -- rn:R09578 <-- (E)-Phenylacetaldoxime --> rn:R11732

rn:R10562 { (E,E)-Geranyllinalool } <-- [Reduced NADPH---hemoprotein reductase] -- rn:R09578 <-- (E)-Phenylacetaldoxime --> rn:R11732

rn:R11055 { Myrcene } <-- [Reduced NADPH---hemoprotein reductase] -- rn:R09578 <-- (E)-Phenylacetaldoxime --> rn:R11732

rn:R11068 { 3-[(1R,2S,5R,6S)-5-Hydroxy-7-oxabicyclo[4.1.0]heptan-2-yl]-2-oxopropanoate } <-- L-Phenylalanine -- rn:R09578 <-- (E)-Phenylacetaldoxime --> rn:R11732

rn:R02468 { (-)-Limonene } <-- [Reduced NADPH---hemoprotein reductase] -- rn:R09578 <-- (E)-Phenylacetaldoxime --> rn:R11737

rn:R02469 { (-)-Limonene } <-- [Reduced NADPH---hemoprotein reductase] -- rn:R09578 <-- (E)-Phenylacetaldoxime --> rn:R11737

rn:R02470 { (-)-Limonene } <-- [Reduced NADPH---hemoprotein reductase] -- rn:R09578 <-- (E)-Phenylacetaldoxime --> rn:R11737

rn:R04366 { Linalool } <-- [Reduced NADPH---hemoprotein reductase] -- rn:R09578 <-- (E)-Phenylacetaldoxime --> rn:R11737

rn:R06119 { d-Limonene } <-- [Reduced NADPH---hemoprotein reductase] -- rn:R09578 <-- (E)-Phenylacetaldoxime --> rn:R11737

rn:R09451 { Hexadecanoic acid } <-- [Reduced NADPH---hemoprotein reductase] -- rn:R09578 <-- (E)-Phenylacetaldoxime --> rn:R11737

rn:R09452 { (9Z)-Octadecenoic acid } <-- [Reduced NADPH---hemoprotein reductase] -- rn:R09578 <-- (E)-Phenylacetaldoxime --> rn:R11737

rn:R09922 { (+)-Linalool } <-- [Reduced NADPH---hemoprotein reductase] -- rn:R09578 <-- (E)-Phenylacetaldoxime --> rn:R11737

rn:R09923 { (-)-Linalool } <-- [Reduced NADPH---hemoprotein reductase] -- rn:R09578 <-- (E)-Phenylacetaldoxime --> rn:R11737

rn:R09925 { Linalool } <-- [Reduced NADPH---hemoprotein reductase] -- rn:R09578 <-- (E)-Phenylacetaldoxime --> rn:R11737

rn:R09934 { Humulene } <-- [Reduced NADPH---hemoprotein reductase] -- rn:R09578 <-- (E)-Phenylacetaldoxime --> rn:R11737

rn:R10562 { (E,E)-Geranyllinalool } <-- [Reduced NADPH---hemoprotein reductase] -- rn:R09578 <-- (E)-Phenylacetaldoxime --> rn:R11737

rn:R11055 { Myrcene } <-- [Reduced NADPH---hemoprotein reductase] -- rn:R09578 <-- (E)-Phenylacetaldoxime --> rn:R11737

rn:R11068 { 3-[(1R,2S,5R,6S)-5-Hydroxy-7-oxabicyclo[4.1.0]heptan-2-yl]-2-oxopropanoate } <-- L-Phenylalanine -- rn:R09578 <-- (E)-Phenylacetaldoxime --> rn:R11737

rn:R02468 { (-)-Limonene } <-- [Reduced NADPH---hemoprotein reductase] -- rn:R09578 <-- (E)-Phenylacetaldoxime --> rn:R11738

rn:R02469 { (-)-Limonene } <-- [Reduced NADPH---hemoprotein reductase] -- rn:R09578 <-- (E)-Phenylacetaldoxime --> rn:R11738

rn:R02470 { (-)-Limonene } <-- [Reduced NADPH---hemoprotein reductase] -- rn:R09578 <-- (E)-Phenylacetaldoxime --> rn:R11738

rn:R04366 { Linalool } <-- [Reduced NADPH---hemoprotein reductase] -- rn:R09578 <-- (E)-Phenylacetaldoxime --> rn:R11738

rn:R06119 { d-Limonene } <-- [Reduced NADPH---hemoprotein reductase] -- rn:R09578 <-- (E)-Phenylacetaldoxime --> rn:R11738

rn:R09451 { Hexadecanoic acid } <-- [Reduced NADPH---hemoprotein reductase] -- rn:R09578 <-- (E)-Phenylacetaldoxime --> rn:R11738

rn:R09452 { (9Z)-Octadecenoic acid } <-- [Reduced NADPH---hemoprotein reductase] -- rn:R09578 <-- (E)-Phenylacetaldoxime --> rn:R11738

rn:R09922 { (+)-Linalool } <-- [Reduced NADPH---hemoprotein reductase] -- rn:R09578 <-- (E)-Phenylacetaldoxime --> rn:R11738

rn:R09923 { (-)-Linalool } <-- [Reduced NADPH---hemoprotein reductase] -- rn:R09578 <-- (E)-Phenylacetaldoxime --> rn:R11738

rn:R09925 { Linalool } <-- [Reduced NADPH---hemoprotein reductase] -- rn:R09578 <-- (E)-Phenylacetaldoxime --> rn:R11738

rn:R09934 { Humulene } <-- [Reduced NADPH---hemoprotein reductase] -- rn:R09578 <-- (E)-Phenylacetaldoxime --> rn:R11738

rn:R10562 { (E,E)-Geranyllinalool } <-- [Reduced NADPH---hemoprotein reductase] -- rn:R09578 <-- (E)-Phenylacetaldoxime --> rn:R11738

rn:R11055 { Myrcene } <-- [Reduced NADPH---hemoprotein reductase] -- rn:R09578 <-- (E)-Phenylacetaldoxime --> rn:R11738

rn:R11068 { 3-[(1R,2S,5R,6S)-5-Hydroxy-7-oxabicyclo[4.1.0]heptan-2-yl]-2-oxopropanoate } <-- L-Phenylalanine -- rn:R09578 <-- (E)-Phenylacetaldoxime --> rn:R11738

rn:R02468 { (-)-Limonene } <-- [Reduced NADPH---hemoprotein reductase] -- rn:R09579 <-- [Oxidized NADPH---hemoprotein reductase] --> rn:R08551

rn:R02469 { (-)-Limonene } <-- [Reduced NADPH---hemoprotein reductase] -- rn:R09579 <-- [Oxidized NADPH---hemoprotein reductase] --> rn:R08551

rn:R02470 { (-)-Limonene } <-- [Reduced NADPH---hemoprotein reductase] -- rn:R09579 <-- [Oxidized NADPH---hemoprotein reductase] --> rn:R08551

rn:R04366 { Linalool } <-- [Reduced NADPH---hemoprotein reductase] -- rn:R09579 <-- [Oxidized NADPH---hemoprotein reductase] --> rn:R08551

rn:R06119 { d-Limonene } <-- [Reduced NADPH---hemoprotein reductase] -- rn:R09579 <-- [Oxidized NADPH---hemoprotein reductase] --> rn:R08551

rn:R09451 { Hexadecanoic acid } <-- [Reduced NADPH---hemoprotein reductase] -- rn:R09579 <-- [Oxidized NADPH---hemoprotein reductase] --> rn:R08551

rn:R09452 { (9Z)-Octadecenoic acid } <-- [Reduced NADPH---hemoprotein reductase] -- rn:R09579 <-- [Oxidized NADPH---hemoprotein reductase] --> rn:R08551

rn:R09922 { (+)-Linalool } <-- [Reduced NADPH---hemoprotein reductase] -- rn:R09579 <-- [Oxidized NADPH---hemoprotein reductase] --> rn:R08551

rn:R09923 { (-)-Linalool } <-- [Reduced NADPH---hemoprotein reductase] -- rn:R09579 <-- [Oxidized NADPH---hemoprotein reductase] --> rn:R08551

rn:R09925 { Linalool } <-- [Reduced NADPH---hemoprotein reductase] -- rn:R09579 <-- [Oxidized NADPH---hemoprotein reductase] --> rn:R08551

rn:R09934 { Humulene } <-- [Reduced NADPH---hemoprotein reductase] -- rn:R09579 <-- [Oxidized NADPH---hemoprotein reductase] --> rn:R08551

rn:R10562 { (E,E)-Geranyllinalool } <-- [Reduced NADPH---hemoprotein reductase] -- rn:R09579 <-- [Oxidized NADPH---hemoprotein reductase] --> rn:R08551

rn:R11055 { Myrcene } <-- [Reduced NADPH---hemoprotein reductase] -- rn:R09579 <-- [Oxidized NADPH---hemoprotein reductase] --> rn:R08551

rn:R11068 { 3-[(1R,2S,5R,6S)-5-Hydroxy-7-oxabicyclo[4.1.0]heptan-2-yl]-2-oxopropanoate } <-- L-Phenylalanine -- rn:R09579 <-- [Oxidized NADPH---hemoprotein reductase] --> rn:R08551

rn:R02468 { (-)-Limonene } <-- [Reduced NADPH---hemoprotein reductase] -- rn:R09579 <-- N-Hydroxy-L-phenylalanine --> rn:R09580

rn:R02469 { (-)-Limonene } <-- [Reduced NADPH---hemoprotein reductase] -- rn:R09579 <-- N-Hydroxy-L-phenylalanine --> rn:R09580

rn:R02470 { (-)-Limonene } <-- [Reduced NADPH---hemoprotein reductase] -- rn:R09579 <-- N-Hydroxy-L-phenylalanine --> rn:R09580

rn:R04366 { Linalool } <-- [Reduced NADPH---hemoprotein reductase] -- rn:R09579 <-- N-Hydroxy-L-phenylalanine --> rn:R09580

rn:R06119 { d-Limonene } <-- [Reduced NADPH---hemoprotein reductase] -- rn:R09579 <-- N-Hydroxy-L-phenylalanine --> rn:R09580

rn:R09451 { Hexadecanoic acid } <-- [Reduced NADPH---hemoprotein reductase] -- rn:R09579 <-- N-Hydroxy-L-phenylalanine --> rn:R09580

rn:R09452 { (9Z)-Octadecenoic acid } <-- [Reduced NADPH---hemoprotein reductase] -- rn:R09579 <-- N-Hydroxy-L-phenylalanine --> rn:R09580

rn:R09922 { (+)-Linalool } <-- [Reduced NADPH---hemoprotein reductase] -- rn:R09579 <-- N-Hydroxy-L-phenylalanine --> rn:R09580

rn:R09923 { (-)-Linalool } <-- [Reduced NADPH---hemoprotein reductase] -- rn:R09579 <-- N-Hydroxy-L-phenylalanine --> rn:R09580

rn:R09925 { Linalool } <-- [Reduced NADPH---hemoprotein reductase] -- rn:R09579 <-- N-Hydroxy-L-phenylalanine --> rn:R09580

rn:R09934 { Humulene } <-- [Reduced NADPH---hemoprotein reductase] -- rn:R09579 <-- N-Hydroxy-L-phenylalanine --> rn:R09580

rn:R10562 { (E,E)-Geranyllinalool } <-- [Reduced NADPH---hemoprotein reductase] -- rn:R09579 <-- N-Hydroxy-L-phenylalanine --> rn:R09580

rn:R11055 { Myrcene } <-- [Reduced NADPH---hemoprotein reductase] -- rn:R09579 <-- N-Hydroxy-L-phenylalanine --> rn:R09580

rn:R11068 { 3-[(1R,2S,5R,6S)-5-Hydroxy-7-oxabicyclo[4.1.0]heptan-2-yl]-2-oxopropanoate } <-- L-Phenylalanine -- rn:R09579 <-- N-Hydroxy-L-phenylalanine --> rn:R09580

rn:R02468 { (-)-Limonene } <-- [Reduced NADPH---hemoprotein reductase] -- rn:R09580 <-- [Oxidized NADPH---hemoprotein reductase] --> rn:R08551

rn:R02469 { (-)-Limonene } <-- [Reduced NADPH---hemoprotein reductase] -- rn:R09580 <-- [Oxidized NADPH---hemoprotein reductase] --> rn:R08551

rn:R02470 { (-)-Limonene } <-- [Reduced NADPH---hemoprotein reductase] -- rn:R09580 <-- [Oxidized NADPH---hemoprotein reductase] --> rn:R08551

rn:R04366 { Linalool } <-- [Reduced NADPH---hemoprotein reductase] -- rn:R09580 <-- [Oxidized NADPH---hemoprotein reductase] --> rn:R08551

rn:R06119 { d-Limonene } <-- [Reduced NADPH---hemoprotein reductase] -- rn:R09580 <-- [Oxidized NADPH---hemoprotein reductase] --> rn:R08551

rn:R09451 { Hexadecanoic acid } <-- [Reduced NADPH---hemoprotein reductase] -- rn:R09580 <-- [Oxidized NADPH---hemoprotein reductase] --> rn:R08551

rn:R09452 { (9Z)-Octadecenoic acid } <-- [Reduced NADPH---hemoprotein reductase] -- rn:R09580 <-- [Oxidized NADPH---hemoprotein reductase] --> rn:R08551

rn:R09922 { (+)-Linalool } <-- [Reduced NADPH---hemoprotein reductase] -- rn:R09580 <-- [Oxidized NADPH---hemoprotein reductase] --> rn:R08551

rn:R09923 { (-)-Linalool } <-- [Reduced NADPH---hemoprotein reductase] -- rn:R09580 <-- [Oxidized NADPH---hemoprotein reductase] --> rn:R08551

rn:R09925 { Linalool } <-- [Reduced NADPH---hemoprotein reductase] -- rn:R09580 <-- [Oxidized NADPH---hemoprotein reductase] --> rn:R08551

rn:R09934 { Humulene } <-- [Reduced NADPH---hemoprotein reductase] -- rn:R09580 <-- [Oxidized NADPH---hemoprotein reductase] --> rn:R08551

rn:R10562 { (E,E)-Geranyllinalool } <-- [Reduced NADPH---hemoprotein reductase] -- rn:R09580 <-- [Oxidized NADPH---hemoprotein reductase] --> rn:R08551

rn:R11055 { Myrcene } <-- [Reduced NADPH---hemoprotein reductase] -- rn:R09580 <-- [Oxidized NADPH---hemoprotein reductase] --> rn:R08551

rn:R02468 { (-)-Limonene } <-- [Reduced NADPH---hemoprotein reductase] -- rn:R09580 <-- N,N-Dihydroxy-L-phenylalanine --> rn:R09581

rn:R02469 { (-)-Limonene } <-- [Reduced NADPH---hemoprotein reductase] -- rn:R09580 <-- N,N-Dihydroxy-L-phenylalanine --> rn:R09581

rn:R02470 { (-)-Limonene } <-- [Reduced NADPH---hemoprotein reductase] -- rn:R09580 <-- N,N-Dihydroxy-L-phenylalanine --> rn:R09581

rn:R04366 { Linalool } <-- [Reduced NADPH---hemoprotein reductase] -- rn:R09580 <-- N,N-Dihydroxy-L-phenylalanine --> rn:R09581

rn:R06119 { d-Limonene } <-- [Reduced NADPH---hemoprotein reductase] -- rn:R09580 <-- N,N-Dihydroxy-L-phenylalanine --> rn:R09581

rn:R09451 { Hexadecanoic acid } <-- [Reduced NADPH---hemoprotein reductase] -- rn:R09580 <-- N,N-Dihydroxy-L-phenylalanine --> rn:R09581

rn:R09452 { (9Z)-Octadecenoic acid } <-- [Reduced NADPH---hemoprotein reductase] -- rn:R09580 <-- N,N-Dihydroxy-L-phenylalanine --> rn:R09581

rn:R09922 { (+)-Linalool } <-- [Reduced NADPH---hemoprotein reductase] -- rn:R09580 <-- N,N-Dihydroxy-L-phenylalanine --> rn:R09581

rn:R09923 { (-)-Linalool } <-- [Reduced NADPH---hemoprotein reductase] -- rn:R09580 <-- N,N-Dihydroxy-L-phenylalanine --> rn:R09581

rn:R09925 { Linalool } <-- [Reduced NADPH---hemoprotein reductase] -- rn:R09580 <-- N,N-Dihydroxy-L-phenylalanine --> rn:R09581

rn:R09934 { Humulene } <-- [Reduced NADPH---hemoprotein reductase] -- rn:R09580 <-- N,N-Dihydroxy-L-phenylalanine --> rn:R09581

rn:R10562 { (E,E)-Geranyllinalool } <-- [Reduced NADPH---hemoprotein reductase] -- rn:R09580 <-- N,N-Dihydroxy-L-phenylalanine --> rn:R09581

rn:R11055 { Myrcene } <-- [Reduced NADPH---hemoprotein reductase] -- rn:R09580 <-- N,N-Dihydroxy-L-phenylalanine --> rn:R09581

rn:R02781 { 2,4,6/3,5-Pentahydroxycyclohexanone } <-- L-Glutamine -- rn:R09598 <-- L-Glutamate --> rn:R00114

rn:R02781 { 2,4,6/3,5-Pentahydroxycyclohexanone } <-- L-Glutamine -- rn:R09598 <-- L-Glutamate --> rn:R00248

rn:R02781 { 2,4,6/3,5-Pentahydroxycyclohexanone } <-- L-Glutamine -- rn:R09598 <-- L-Glutamate --> rn:R00254

rn:R02781 { 2,4,6/3,5-Pentahydroxycyclohexanone } <-- L-Glutamine -- rn:R09598 <-- L-Glutamate --> rn:R00894

rn:R02781 { 2,4,6/3,5-Pentahydroxycyclohexanone } <-- L-Glutamine -- rn:R09598 <-- L-Glutamate --> rn:R02287

rn:R02781 { 2,4,6/3,5-Pentahydroxycyclohexanone } <-- L-Glutamine -- rn:R09598 <-- L-Glutamate --> rn:R03189

rn:R02781 { 2,4,6/3,5-Pentahydroxycyclohexanone } <-- L-Glutamine -- rn:R09598 <-- L-Glutamate --> rn:R03970

rn:R02781 { 2,4,6/3,5-Pentahydroxycyclohexanone } <-- L-Glutamine -- rn:R09598 <-- L-Glutamate --> rn:R03971

rn:R02781 { 2,4,6/3,5-Pentahydroxycyclohexanone } <-- L-Glutamine -- rn:R09598 <-- L-Glutamate --> rn:R04051

rn:R02781 { 2,4,6/3,5-Pentahydroxycyclohexanone } <-- L-Glutamine -- rn:R09598 <-- L-Glutamate --> rn:R04776

rn:R02781 { 2,4,6/3,5-Pentahydroxycyclohexanone } <-- L-Glutamine -- rn:R09598 <-- L-Glutamate --> rn:R07643

rn:R02781 { 2,4,6/3,5-Pentahydroxycyclohexanone } <-- L-Glutamine -- rn:R09598 <-- Cobyrinate c-monamide --> rn:R09599

rn:R02781 { 2,4,6/3,5-Pentahydroxycyclohexanone } <-- L-Glutamine -- rn:R09599 <-- L-Glutamate --> rn:R00114

rn:R02781 { 2,4,6/3,5-Pentahydroxycyclohexanone } <-- L-Glutamine -- rn:R09599 <-- L-Glutamate --> rn:R00248

rn:R02781 { 2,4,6/3,5-Pentahydroxycyclohexanone } <-- L-Glutamine -- rn:R09599 <-- L-Glutamate --> rn:R00254

rn:R02781 { 2,4,6/3,5-Pentahydroxycyclohexanone } <-- L-Glutamine -- rn:R09599 <-- L-Glutamate --> rn:R00894

rn:R02781 { 2,4,6/3,5-Pentahydroxycyclohexanone } <-- L-Glutamine -- rn:R09599 <-- L-Glutamate --> rn:R02287

rn:R02781 { 2,4,6/3,5-Pentahydroxycyclohexanone } <-- L-Glutamine -- rn:R09599 <-- L-Glutamate --> rn:R03189

rn:R02781 { 2,4,6/3,5-Pentahydroxycyclohexanone } <-- L-Glutamine -- rn:R09599 <-- L-Glutamate --> rn:R03970

rn:R02781 { 2,4,6/3,5-Pentahydroxycyclohexanone } <-- L-Glutamine -- rn:R09599 <-- L-Glutamate --> rn:R03971

rn:R02781 { 2,4,6/3,5-Pentahydroxycyclohexanone } <-- L-Glutamine -- rn:R09599 <-- L-Glutamate --> rn:R04051

rn:R02781 { 2,4,6/3,5-Pentahydroxycyclohexanone } <-- L-Glutamine -- rn:R09599 <-- L-Glutamate --> rn:R04776

rn:R02781 { 2,4,6/3,5-Pentahydroxycyclohexanone } <-- L-Glutamine -- rn:R09599 <-- Cob(II)yrinate a,c diamide --> rn:R05218

rn:R02781 { 2,4,6/3,5-Pentahydroxycyclohexanone } <-- L-Glutamine -- rn:R09599 <-- L-Glutamate --> rn:R07643

rn:R02781 { 2,4,6/3,5-Pentahydroxycyclohexanone } <-- L-Glutamine -- rn:R09599 <-- Cob(II)yrinate a,c diamide --> rn:R12184

rn:R02468 { (-)-Limonene } <-- [Reduced NADPH---hemoprotein reductase] -- rn:R10027 <-- [Oxidized NADPH---hemoprotein reductase] --> rn:R08551

rn:R02469 { (-)-Limonene } <-- [Reduced NADPH---hemoprotein reductase] -- rn:R10027 <-- [Oxidized NADPH---hemoprotein reductase] --> rn:R08551

rn:R02470 { (-)-Limonene } <-- [Reduced NADPH---hemoprotein reductase] -- rn:R10027 <-- [Oxidized NADPH---hemoprotein reductase] --> rn:R08551

rn:R04366 { Linalool } <-- [Reduced NADPH---hemoprotein reductase] -- rn:R10027 <-- [Oxidized NADPH---hemoprotein reductase] --> rn:R08551

rn:R06119 { d-Limonene } <-- [Reduced NADPH---hemoprotein reductase] -- rn:R10027 <-- [Oxidized NADPH---hemoprotein reductase] --> rn:R08551

rn:R09451 { Hexadecanoic acid } <-- [Reduced NADPH---hemoprotein reductase] -- rn:R10027 <-- [Oxidized NADPH---hemoprotein reductase] --> rn:R08551

rn:R09452 { (9Z)-Octadecenoic acid } <-- [Reduced NADPH---hemoprotein reductase] -- rn:R10027 <-- [Oxidized NADPH---hemoprotein reductase] --> rn:R08551

rn:R09922 { (+)-Linalool } <-- [Reduced NADPH---hemoprotein reductase] -- rn:R10027 <-- [Oxidized NADPH---hemoprotein reductase] --> rn:R08551

rn:R09923 { (-)-Linalool } <-- [Reduced NADPH---hemoprotein reductase] -- rn:R10027 <-- [Oxidized NADPH---hemoprotein reductase] --> rn:R08551

rn:R09925 { Linalool } <-- [Reduced NADPH---hemoprotein reductase] -- rn:R10027 <-- [Oxidized NADPH---hemoprotein reductase] --> rn:R08551

rn:R09934 { Humulene } <-- [Reduced NADPH---hemoprotein reductase] -- rn:R10027 <-- [Oxidized NADPH---hemoprotein reductase] --> rn:R08551

rn:R10562 { (E,E)-Geranyllinalool } <-- [Reduced NADPH---hemoprotein reductase] -- rn:R10027 <-- [Oxidized NADPH---hemoprotein reductase] --> rn:R08551

rn:R11055 { Myrcene } <-- [Reduced NADPH---hemoprotein reductase] -- rn:R10027 <-- [Oxidized NADPH---hemoprotein reductase] --> rn:R08551

rn:R02468 { (-)-Limonene } <-- [Reduced NADPH---hemoprotein reductase] -- rn:R10027 <-- N-Hydroxy-L-isoleucine --> rn:R10028

rn:R02469 { (-)-Limonene } <-- [Reduced NADPH---hemoprotein reductase] -- rn:R10027 <-- N-Hydroxy-L-isoleucine --> rn:R10028

rn:R02470 { (-)-Limonene } <-- [Reduced NADPH---hemoprotein reductase] -- rn:R10027 <-- N-Hydroxy-L-isoleucine --> rn:R10028

rn:R04366 { Linalool } <-- [Reduced NADPH---hemoprotein reductase] -- rn:R10027 <-- N-Hydroxy-L-isoleucine --> rn:R10028

rn:R06119 { d-Limonene } <-- [Reduced NADPH---hemoprotein reductase] -- rn:R10027 <-- N-Hydroxy-L-isoleucine --> rn:R10028

rn:R09451 { Hexadecanoic acid } <-- [Reduced NADPH---hemoprotein reductase] -- rn:R10027 <-- N-Hydroxy-L-isoleucine --> rn:R10028

rn:R09452 { (9Z)-Octadecenoic acid } <-- [Reduced NADPH---hemoprotein reductase] -- rn:R10027 <-- N-Hydroxy-L-isoleucine --> rn:R10028

rn:R09922 { (+)-Linalool } <-- [Reduced NADPH---hemoprotein reductase] -- rn:R10027 <-- N-Hydroxy-L-isoleucine --> rn:R10028

rn:R09923 { (-)-Linalool } <-- [Reduced NADPH---hemoprotein reductase] -- rn:R10027 <-- N-Hydroxy-L-isoleucine --> rn:R10028

rn:R09925 { Linalool } <-- [Reduced NADPH---hemoprotein reductase] -- rn:R10027 <-- N-Hydroxy-L-isoleucine --> rn:R10028

rn:R09934 { Humulene } <-- [Reduced NADPH---hemoprotein reductase] -- rn:R10027 <-- N-Hydroxy-L-isoleucine --> rn:R10028

rn:R10562 { (E,E)-Geranyllinalool } <-- [Reduced NADPH---hemoprotein reductase] -- rn:R10027 <-- N-Hydroxy-L-isoleucine --> rn:R10028

rn:R11055 { Myrcene } <-- [Reduced NADPH---hemoprotein reductase] -- rn:R10027 <-- N-Hydroxy-L-isoleucine --> rn:R10028

rn:R02468 { (-)-Limonene } <-- [Reduced NADPH---hemoprotein reductase] -- rn:R10028 <-- [Oxidized NADPH---hemoprotein reductase] --> rn:R08551

rn:R02469 { (-)-Limonene } <-- [Reduced NADPH---hemoprotein reductase] -- rn:R10028 <-- [Oxidized NADPH---hemoprotein reductase] --> rn:R08551

rn:R02470 { (-)-Limonene } <-- [Reduced NADPH---hemoprotein reductase] -- rn:R10028 <-- [Oxidized NADPH---hemoprotein reductase] --> rn:R08551

rn:R04366 { Linalool } <-- [Reduced NADPH---hemoprotein reductase] -- rn:R10028 <-- [Oxidized NADPH---hemoprotein reductase] --> rn:R08551

rn:R06119 { d-Limonene } <-- [Reduced NADPH---hemoprotein reductase] -- rn:R10028 <-- [Oxidized NADPH---hemoprotein reductase] --> rn:R08551

rn:R09451 { Hexadecanoic acid } <-- [Reduced NADPH---hemoprotein reductase] -- rn:R10028 <-- [Oxidized NADPH---hemoprotein reductase] --> rn:R08551

rn:R09452 { (9Z)-Octadecenoic acid } <-- [Reduced NADPH---hemoprotein reductase] -- rn:R10028 <-- [Oxidized NADPH---hemoprotein reductase] --> rn:R08551

rn:R09922 { (+)-Linalool } <-- [Reduced NADPH---hemoprotein reductase] -- rn:R10028 <-- [Oxidized NADPH---hemoprotein reductase] --> rn:R08551

rn:R09923 { (-)-Linalool } <-- [Reduced NADPH---hemoprotein reductase] -- rn:R10028 <-- [Oxidized NADPH---hemoprotein reductase] --> rn:R08551

rn:R09925 { Linalool } <-- [Reduced NADPH---hemoprotein reductase] -- rn:R10028 <-- [Oxidized NADPH---hemoprotein reductase] --> rn:R08551

rn:R09934 { Humulene } <-- [Reduced NADPH---hemoprotein reductase] -- rn:R10028 <-- [Oxidized NADPH---hemoprotein reductase] --> rn:R08551

rn:R10562 { (E,E)-Geranyllinalool } <-- [Reduced NADPH---hemoprotein reductase] -- rn:R10028 <-- [Oxidized NADPH---hemoprotein reductase] --> rn:R08551

rn:R11055 { Myrcene } <-- [Reduced NADPH---hemoprotein reductase] -- rn:R10028 <-- [Oxidized NADPH---hemoprotein reductase] --> rn:R08551

rn:R02468 { (-)-Limonene } <-- [Reduced NADPH---hemoprotein reductase] -- rn:R10028 <-- N,N-Dihydroxy-L-isoleucine --> rn:R10029

rn:R02469 { (-)-Limonene } <-- [Reduced NADPH---hemoprotein reductase] -- rn:R10028 <-- N,N-Dihydroxy-L-isoleucine --> rn:R10029

rn:R02470 { (-)-Limonene } <-- [Reduced NADPH---hemoprotein reductase] -- rn:R10028 <-- N,N-Dihydroxy-L-isoleucine --> rn:R10029

rn:R04366 { Linalool } <-- [Reduced NADPH---hemoprotein reductase] -- rn:R10028 <-- N,N-Dihydroxy-L-isoleucine --> rn:R10029

rn:R06119 { d-Limonene } <-- [Reduced NADPH---hemoprotein reductase] -- rn:R10028 <-- N,N-Dihydroxy-L-isoleucine --> rn:R10029

rn:R09451 { Hexadecanoic acid } <-- [Reduced NADPH---hemoprotein reductase] -- rn:R10028 <-- N,N-Dihydroxy-L-isoleucine --> rn:R10029

rn:R09452 { (9Z)-Octadecenoic acid } <-- [Reduced NADPH---hemoprotein reductase] -- rn:R10028 <-- N,N-Dihydroxy-L-isoleucine --> rn:R10029

rn:R09922 { (+)-Linalool } <-- [Reduced NADPH---hemoprotein reductase] -- rn:R10028 <-- N,N-Dihydroxy-L-isoleucine --> rn:R10029

rn:R09923 { (-)-Linalool } <-- [Reduced NADPH---hemoprotein reductase] -- rn:R10028 <-- N,N-Dihydroxy-L-isoleucine --> rn:R10029

rn:R09925 { Linalool } <-- [Reduced NADPH---hemoprotein reductase] -- rn:R10028 <-- N,N-Dihydroxy-L-isoleucine --> rn:R10029

rn:R09934 { Humulene } <-- [Reduced NADPH---hemoprotein reductase] -- rn:R10028 <-- N,N-Dihydroxy-L-isoleucine --> rn:R10029

rn:R10562 { (E,E)-Geranyllinalool } <-- [Reduced NADPH---hemoprotein reductase] -- rn:R10028 <-- N,N-Dihydroxy-L-isoleucine --> rn:R10029

rn:R11055 { Myrcene } <-- [Reduced NADPH---hemoprotein reductase] -- rn:R10028 <-- N,N-Dihydroxy-L-isoleucine --> rn:R10029

rn:R02468 { (-)-Limonene } <-- [Reduced NADPH---hemoprotein reductase] -- rn:R10031 <-- [Oxidized NADPH---hemoprotein reductase] --> rn:R08551

rn:R02469 { (-)-Limonene } <-- [Reduced NADPH---hemoprotein reductase] -- rn:R10031 <-- [Oxidized NADPH---hemoprotein reductase] --> rn:R08551

rn:R02470 { (-)-Limonene } <-- [Reduced NADPH---hemoprotein reductase] -- rn:R10031 <-- [Oxidized NADPH---hemoprotein reductase] --> rn:R08551

rn:R04366 { Linalool } <-- [Reduced NADPH---hemoprotein reductase] -- rn:R10031 <-- [Oxidized NADPH---hemoprotein reductase] --> rn:R08551

rn:R06119 { d-Limonene } <-- [Reduced NADPH---hemoprotein reductase] -- rn:R10031 <-- [Oxidized NADPH---hemoprotein reductase] --> rn:R08551

rn:R09451 { Hexadecanoic acid } <-- [Reduced NADPH---hemoprotein reductase] -- rn:R10031 <-- [Oxidized NADPH---hemoprotein reductase] --> rn:R08551

rn:R09452 { (9Z)-Octadecenoic acid } <-- [Reduced NADPH---hemoprotein reductase] -- rn:R10031 <-- [Oxidized NADPH---hemoprotein reductase] --> rn:R08551

rn:R09922 { (+)-Linalool } <-- [Reduced NADPH---hemoprotein reductase] -- rn:R10031 <-- [Oxidized NADPH---hemoprotein reductase] --> rn:R08551

rn:R09923 { (-)-Linalool } <-- [Reduced NADPH---hemoprotein reductase] -- rn:R10031 <-- [Oxidized NADPH---hemoprotein reductase] --> rn:R08551

rn:R09925 { Linalool } <-- [Reduced NADPH---hemoprotein reductase] -- rn:R10031 <-- [Oxidized NADPH---hemoprotein reductase] --> rn:R08551

rn:R09934 { Humulene } <-- [Reduced NADPH---hemoprotein reductase] -- rn:R10031 <-- [Oxidized NADPH---hemoprotein reductase] --> rn:R08551

rn:R10562 { (E,E)-Geranyllinalool } <-- [Reduced NADPH---hemoprotein reductase] -- rn:R10031 <-- [Oxidized NADPH---hemoprotein reductase] --> rn:R08551

rn:R11055 { Myrcene } <-- [Reduced NADPH---hemoprotein reductase] -- rn:R10031 <-- [Oxidized NADPH---hemoprotein reductase] --> rn:R08551

rn:R02468 { (-)-Limonene } <-- [Reduced NADPH---hemoprotein reductase] -- rn:R10031 <-- N-Hydroxy-L-valine --> rn:R10032

rn:R02469 { (-)-Limonene } <-- [Reduced NADPH---hemoprotein reductase] -- rn:R10031 <-- N-Hydroxy-L-valine --> rn:R10032

rn:R02470 { (-)-Limonene } <-- [Reduced NADPH---hemoprotein reductase] -- rn:R10031 <-- N-Hydroxy-L-valine --> rn:R10032

rn:R04366 { Linalool } <-- [Reduced NADPH---hemoprotein reductase] -- rn:R10031 <-- N-Hydroxy-L-valine --> rn:R10032

rn:R06119 { d-Limonene } <-- [Reduced NADPH---hemoprotein reductase] -- rn:R10031 <-- N-Hydroxy-L-valine --> rn:R10032

rn:R09451 { Hexadecanoic acid } <-- [Reduced NADPH---hemoprotein reductase] -- rn:R10031 <-- N-Hydroxy-L-valine --> rn:R10032

rn:R09452 { (9Z)-Octadecenoic acid } <-- [Reduced NADPH---hemoprotein reductase] -- rn:R10031 <-- N-Hydroxy-L-valine --> rn:R10032

rn:R09922 { (+)-Linalool } <-- [Reduced NADPH---hemoprotein reductase] -- rn:R10031 <-- N-Hydroxy-L-valine --> rn:R10032

rn:R09923 { (-)-Linalool } <-- [Reduced NADPH---hemoprotein reductase] -- rn:R10031 <-- N-Hydroxy-L-valine --> rn:R10032

rn:R09925 { Linalool } <-- [Reduced NADPH---hemoprotein reductase] -- rn:R10031 <-- N-Hydroxy-L-valine --> rn:R10032

rn:R09934 { Humulene } <-- [Reduced NADPH---hemoprotein reductase] -- rn:R10031 <-- N-Hydroxy-L-valine --> rn:R10032

rn:R10562 { (E,E)-Geranyllinalool } <-- [Reduced NADPH---hemoprotein reductase] -- rn:R10031 <-- N-Hydroxy-L-valine --> rn:R10032

rn:R11055 { Myrcene } <-- [Reduced NADPH---hemoprotein reductase] -- rn:R10031 <-- N-Hydroxy-L-valine --> rn:R10032

rn:R02468 { (-)-Limonene } <-- [Reduced NADPH---hemoprotein reductase] -- rn:R10032 <-- [Oxidized NADPH---hemoprotein reductase] --> rn:R08551

rn:R02469 { (-)-Limonene } <-- [Reduced NADPH---hemoprotein reductase] -- rn:R10032 <-- [Oxidized NADPH---hemoprotein reductase] --> rn:R08551

rn:R02470 { (-)-Limonene } <-- [Reduced NADPH---hemoprotein reductase] -- rn:R10032 <-- [Oxidized NADPH---hemoprotein reductase] --> rn:R08551

rn:R04366 { Linalool } <-- [Reduced NADPH---hemoprotein reductase] -- rn:R10032 <-- [Oxidized NADPH---hemoprotein reductase] --> rn:R08551

rn:R06119 { d-Limonene } <-- [Reduced NADPH---hemoprotein reductase] -- rn:R10032 <-- [Oxidized NADPH---hemoprotein reductase] --> rn:R08551

rn:R09451 { Hexadecanoic acid } <-- [Reduced NADPH---hemoprotein reductase] -- rn:R10032 <-- [Oxidized NADPH---hemoprotein reductase] --> rn:R08551

rn:R09452 { (9Z)-Octadecenoic acid } <-- [Reduced NADPH---hemoprotein reductase] -- rn:R10032 <-- [Oxidized NADPH---hemoprotein reductase] --> rn:R08551

rn:R09922 { (+)-Linalool } <-- [Reduced NADPH---hemoprotein reductase] -- rn:R10032 <-- [Oxidized NADPH---hemoprotein reductase] --> rn:R08551

rn:R09923 { (-)-Linalool } <-- [Reduced NADPH---hemoprotein reductase] -- rn:R10032 <-- [Oxidized NADPH---hemoprotein reductase] --> rn:R08551

rn:R09925 { Linalool } <-- [Reduced NADPH---hemoprotein reductase] -- rn:R10032 <-- [Oxidized NADPH---hemoprotein reductase] --> rn:R08551

rn:R09934 { Humulene } <-- [Reduced NADPH---hemoprotein reductase] -- rn:R10032 <-- [Oxidized NADPH---hemoprotein reductase] --> rn:R08551

rn:R10562 { (E,E)-Geranyllinalool } <-- [Reduced NADPH---hemoprotein reductase] -- rn:R10032 <-- [Oxidized NADPH---hemoprotein reductase] --> rn:R08551

rn:R11055 { Myrcene } <-- [Reduced NADPH---hemoprotein reductase] -- rn:R10032 <-- [Oxidized NADPH---hemoprotein reductase] --> rn:R08551

rn:R02468 { (-)-Limonene } <-- [Reduced NADPH---hemoprotein reductase] -- rn:R10032 <-- N,N-Dihydroxy-L-valine --> rn:R10033

rn:R02469 { (-)-Limonene } <-- [Reduced NADPH---hemoprotein reductase] -- rn:R10032 <-- N,N-Dihydroxy-L-valine --> rn:R10033

rn:R02470 { (-)-Limonene } <-- [Reduced NADPH---hemoprotein reductase] -- rn:R10032 <-- N,N-Dihydroxy-L-valine --> rn:R10033

rn:R04366 { Linalool } <-- [Reduced NADPH---hemoprotein reductase] -- rn:R10032 <-- N,N-Dihydroxy-L-valine --> rn:R10033

rn:R06119 { d-Limonene } <-- [Reduced NADPH---hemoprotein reductase] -- rn:R10032 <-- N,N-Dihydroxy-L-valine --> rn:R10033

rn:R09451 { Hexadecanoic acid } <-- [Reduced NADPH---hemoprotein reductase] -- rn:R10032 <-- N,N-Dihydroxy-L-valine --> rn:R10033

rn:R09452 { (9Z)-Octadecenoic acid } <-- [Reduced NADPH---hemoprotein reductase] -- rn:R10032 <-- N,N-Dihydroxy-L-valine --> rn:R10033

rn:R09922 { (+)-Linalool } <-- [Reduced NADPH---hemoprotein reductase] -- rn:R10032 <-- N,N-Dihydroxy-L-valine --> rn:R10033

rn:R09923 { (-)-Linalool } <-- [Reduced NADPH---hemoprotein reductase] -- rn:R10032 <-- N,N-Dihydroxy-L-valine --> rn:R10033

rn:R09925 { Linalool } <-- [Reduced NADPH---hemoprotein reductase] -- rn:R10032 <-- N,N-Dihydroxy-L-valine --> rn:R10033

rn:R09934 { Humulene } <-- [Reduced NADPH---hemoprotein reductase] -- rn:R10032 <-- N,N-Dihydroxy-L-valine --> rn:R10033

rn:R10562 { (E,E)-Geranyllinalool } <-- [Reduced NADPH---hemoprotein reductase] -- rn:R10032 <-- N,N-Dihydroxy-L-valine --> rn:R10033

rn:R11055 { Myrcene } <-- [Reduced NADPH---hemoprotein reductase] -- rn:R10032 <-- N,N-Dihydroxy-L-valine --> rn:R10033

rn:R02179 { (-)-Menthol } <-- UDP-glucose -- rn:R10037 <-- Lotaustralin --> rn:R10039

rn:R03548 { 7,8-Dihydroxycoumarin } <-- UDP-glucose -- rn:R10037 <-- Lotaustralin --> rn:R10039

rn:R06776 { 3-Amino-4,7-dihydroxy-8-chlorocoumarin } <-- 3-Dimethylallyl-4-hydroxybenzoate -- rn:R10453 <-- AMP --> rn:R01490

rn:R06776 { 3-Amino-4,7-dihydroxy-8-chlorocoumarin } <-- 3-Dimethylallyl-4-hydroxybenzoate -- rn:R10453 <-- AMP --> rn:R05717

rn:R06776 { 3-Amino-4,7-dihydroxy-8-chlorocoumarin } <-- 3-Dimethylallyl-4-hydroxybenzoate -- rn:R10453 <-- AMP --> rn:R08743

rn:R06776 { 3-Amino-4,7-dihydroxy-8-chlorocoumarin } <-- 3-Dimethylallyl-4-hydroxybenzoate -- rn:R10453 <-- 8-Demethylnovobiocic acid --> rn:R10454

rn:R06776 { 3-Amino-4,7-dihydroxy-8-chlorocoumarin } <-- 3-Dimethylallyl-4-hydroxybenzoate -- rn:R10453 <-- AMP --> rn:R11679

rn:R11068 { 3-[(1R,2S,5R,6S)-5-Hydroxy-7-oxabicyclo[4.1.0]heptan-2-yl]-2-oxopropanoate } <-- L-Phenylalanine -- rn:R10495 <-- AMP --> rn:R01490

rn:R11068 { 3-[(1R,2S,5R,6S)-5-Hydroxy-7-oxabicyclo[4.1.0]heptan-2-yl]-2-oxopropanoate } <-- L-Phenylalanine -- rn:R10495 <-- AMP --> rn:R05717

rn:R11068 { 3-[(1R,2S,5R,6S)-5-Hydroxy-7-oxabicyclo[4.1.0]heptan-2-yl]-2-oxopropanoate } <-- L-Phenylalanine -- rn:R10495 <-- AMP --> rn:R08743

rn:R11068 { 3-[(1R,2S,5R,6S)-5-Hydroxy-7-oxabicyclo[4.1.0]heptan-2-yl]-2-oxopropanoate } <-- L-Phenylalanine -- rn:R10495 <-- AMP --> rn:R11679

rn:R11068 { 3-[(1R,2S,5R,6S)-5-Hydroxy-7-oxabicyclo[4.1.0]heptan-2-yl]-2-oxopropanoate } <-- L-Phenylalanine -- rn:R10499 <-- Phenylpyruvate --> rn:R00695

rn:R11068 { 3-[(1R,2S,5R,6S)-5-Hydroxy-7-oxabicyclo[4.1.0]heptan-2-yl]-2-oxopropanoate } <-- L-Phenylalanine -- rn:R10499 <-- L-Amino acid --> rn:R01260

rn:R11068 { 3-[(1R,2S,5R,6S)-5-Hydroxy-7-oxabicyclo[4.1.0]heptan-2-yl]-2-oxopropanoate } <-- L-Phenylalanine -- rn:R10499 <-- L-Amino acid --> rn:R01262

rn:R11068 { 3-[(1R,2S,5R,6S)-5-Hydroxy-7-oxabicyclo[4.1.0]heptan-2-yl]-2-oxopropanoate } <-- L-Phenylalanine -- rn:R10499 <-- Phenylpyruvate --> rn:R01372

rn:R11068 { 3-[(1R,2S,5R,6S)-5-Hydroxy-7-oxabicyclo[4.1.0]heptan-2-yl]-2-oxopropanoate } <-- L-Phenylalanine -- rn:R10499 <-- Phenylpyruvate --> rn:R01375

rn:R11068 { 3-[(1R,2S,5R,6S)-5-Hydroxy-7-oxabicyclo[4.1.0]heptan-2-yl]-2-oxopropanoate } <-- L-Phenylalanine -- rn:R10499 <-- Phenylpyruvate --> rn:R01376

rn:R11068 { 3-[(1R,2S,5R,6S)-5-Hydroxy-7-oxabicyclo[4.1.0]heptan-2-yl]-2-oxopropanoate } <-- L-Phenylalanine -- rn:R10499 <-- Phenylpyruvate --> rn:R01377

rn:R11068 { 3-[(1R,2S,5R,6S)-5-Hydroxy-7-oxabicyclo[4.1.0]heptan-2-yl]-2-oxopropanoate } <-- L-Phenylalanine -- rn:R10499 <-- Phenylpyruvate --> rn:R01378

rn:R11068 { 3-[(1R,2S,5R,6S)-5-Hydroxy-7-oxabicyclo[4.1.0]heptan-2-yl]-2-oxopropanoate } <-- L-Phenylalanine -- rn:R10499 <-- Phenylpyruvate --> rn:R10431

rn:R11068 { 3-[(1R,2S,5R,6S)-5-Hydroxy-7-oxabicyclo[4.1.0]heptan-2-yl]-2-oxopropanoate } <-- L-Phenylalanine -- rn:R10499 <-- Phenylpyruvate --> rn:R10500

rn:R11068 { 3-[(1R,2S,5R,6S)-5-Hydroxy-7-oxabicyclo[4.1.0]heptan-2-yl]-2-oxopropanoate } <-- L-Phenylalanine -- rn:R10499 <-- L-Amino acid --> rn:R10503

rn:R08530 { (-)-Menthol } <-- Acetyl-CoA -- rn:R10500 <-- 2-Benzylmalic acid --> rn:R10501

rn:R08531 { (+)-Neomenthol } <-- Acetyl-CoA -- rn:R10500 <-- 2-Benzylmalic acid --> rn:R10501

rn:R08532 { (+)-Borneol } <-- Acetyl-CoA -- rn:R10500 <-- 2-Benzylmalic acid --> rn:R10501

rn:R10474 { Cinnamyl alcohol } <-- Acetyl-CoA -- rn:R10500 <-- 2-Benzylmalic acid --> rn:R10501

rn:R08530 { (-)-Menthol } <-- Acetyl-CoA -- rn:R10600 <-- Acetate --> rn:R01241

rn:R08531 { (+)-Neomenthol } <-- Acetyl-CoA -- rn:R10600 <-- Acetate --> rn:R01241

rn:R08532 { (+)-Borneol } <-- Acetyl-CoA -- rn:R10600 <-- Acetate --> rn:R01241

rn:R10474 { Cinnamyl alcohol } <-- Acetyl-CoA -- rn:R10600 <-- Acetate --> rn:R01241

rn:R08530 { (-)-Menthol } <-- Acetyl-CoA -- rn:R10600 <-- Acetate --> rn:R01308

rn:R08531 { (+)-Neomenthol } <-- Acetyl-CoA -- rn:R10600 <-- Acetate --> rn:R01308

rn:R08532 { (+)-Borneol } <-- Acetyl-CoA -- rn:R10600 <-- Acetate --> rn:R01308

rn:R10474 { Cinnamyl alcohol } <-- Acetyl-CoA -- rn:R10600 <-- Acetate --> rn:R01308

rn:R08530 { (-)-Menthol } <-- Acetyl-CoA -- rn:R10600 <-- Acetate --> rn:R01426

rn:R08531 { (+)-Neomenthol } <-- Acetyl-CoA -- rn:R10600 <-- Acetate --> rn:R01426

rn:R08532 { (+)-Borneol } <-- Acetyl-CoA -- rn:R10600 <-- Acetate --> rn:R01426

rn:R10474 { Cinnamyl alcohol } <-- Acetyl-CoA -- rn:R10600 <-- Acetate --> rn:R01426

rn:R08530 { (-)-Menthol } <-- Acetyl-CoA -- rn:R10600 <-- Acetate --> rn:R05138

rn:R08531 { (+)-Neomenthol } <-- Acetyl-CoA -- rn:R10600 <-- Acetate --> rn:R05138

rn:R08532 { (+)-Borneol } <-- Acetyl-CoA -- rn:R10600 <-- Acetate --> rn:R05138

rn:R10474 { Cinnamyl alcohol } <-- Acetyl-CoA -- rn:R10600 <-- Acetate --> rn:R05138

rn:R08530 { (-)-Menthol } <-- Acetyl-CoA -- rn:R10600 <-- Acetate --> rn:R05219

rn:R08531 { (+)-Neomenthol } <-- Acetyl-CoA -- rn:R10600 <-- Acetate --> rn:R05219

rn:R08532 { (+)-Borneol } <-- Acetyl-CoA -- rn:R10600 <-- Acetate --> rn:R05219

rn:R10474 { Cinnamyl alcohol } <-- Acetyl-CoA -- rn:R10600 <-- Acetate --> rn:R05219

rn:R08530 { (-)-Menthol } <-- Acetyl-CoA -- rn:R10600 <-- Acetate --> rn:R09931

rn:R08531 { (+)-Neomenthol } <-- Acetyl-CoA -- rn:R10600 <-- Acetate --> rn:R09931

rn:R08532 { (+)-Borneol } <-- Acetyl-CoA -- rn:R10600 <-- Acetate --> rn:R09931

rn:R10474 { Cinnamyl alcohol } <-- Acetyl-CoA -- rn:R10600 <-- Acetate --> rn:R09931

rn:R02179 { (-)-Menthol } <-- UDP-glucose -- rn:R10638 <-- Prunasin --> rn:R02558

rn:R03548 { 7,8-Dihydroxycoumarin } <-- UDP-glucose -- rn:R10638 <-- Prunasin --> rn:R02558

rn:R02179 { (-)-Menthol } <-- UDP-glucose -- rn:R10638 <-- Prunasin --> rn:R02985

rn:R03548 { 7,8-Dihydroxycoumarin } <-- UDP-glucose -- rn:R10638 <-- Prunasin --> rn:R02985

rn:R02179 { (-)-Menthol } <-- UDP-glucose -- rn:R10638 <-- Prunasin --> rn:R10639

rn:R03548 { 7,8-Dihydroxycoumarin } <-- UDP-glucose -- rn:R10638 <-- Prunasin --> rn:R10639

rn:R02468 { (-)-Limonene } <-- [Reduced NADPH---hemoprotein reductase] -- rn:R10671 <-- (E)-4-Hydroxyphenylacetaldehyde oxime --> rn:R05728

rn:R02469 { (-)-Limonene } <-- [Reduced NADPH---hemoprotein reductase] -- rn:R10671 <-- (E)-4-Hydroxyphenylacetaldehyde oxime --> rn:R05728

rn:R02470 { (-)-Limonene } <-- [Reduced NADPH---hemoprotein reductase] -- rn:R10671 <-- (E)-4-Hydroxyphenylacetaldehyde oxime --> rn:R05728

rn:R04366 { Linalool } <-- [Reduced NADPH---hemoprotein reductase] -- rn:R10671 <-- (E)-4-Hydroxyphenylacetaldehyde oxime --> rn:R05728

rn:R06119 { d-Limonene } <-- [Reduced NADPH---hemoprotein reductase] -- rn:R10671 <-- (E)-4-Hydroxyphenylacetaldehyde oxime --> rn:R05728

rn:R09451 { Hexadecanoic acid } <-- [Reduced NADPH---hemoprotein reductase] -- rn:R10671 <-- (E)-4-Hydroxyphenylacetaldehyde oxime --> rn:R05728

rn:R09452 { (9Z)-Octadecenoic acid } <-- [Reduced NADPH---hemoprotein reductase] -- rn:R10671 <-- (E)-4-Hydroxyphenylacetaldehyde oxime --> rn:R05728

rn:R09922 { (+)-Linalool } <-- [Reduced NADPH---hemoprotein reductase] -- rn:R10671 <-- (E)-4-Hydroxyphenylacetaldehyde oxime --> rn:R05728

rn:R09923 { (-)-Linalool } <-- [Reduced NADPH---hemoprotein reductase] -- rn:R10671 <-- (E)-4-Hydroxyphenylacetaldehyde oxime --> rn:R05728

rn:R09925 { Linalool } <-- [Reduced NADPH---hemoprotein reductase] -- rn:R10671 <-- (E)-4-Hydroxyphenylacetaldehyde oxime --> rn:R05728

rn:R09934 { Humulene } <-- [Reduced NADPH---hemoprotein reductase] -- rn:R10671 <-- (E)-4-Hydroxyphenylacetaldehyde oxime --> rn:R05728

rn:R10562 { (E,E)-Geranyllinalool } <-- [Reduced NADPH---hemoprotein reductase] -- rn:R10671 <-- (E)-4-Hydroxyphenylacetaldehyde oxime --> rn:R05728

rn:R11055 { Myrcene } <-- [Reduced NADPH---hemoprotein reductase] -- rn:R10671 <-- (E)-4-Hydroxyphenylacetaldehyde oxime --> rn:R05728

rn:R02468 { (-)-Limonene } <-- [Reduced NADPH---hemoprotein reductase] -- rn:R10671 <-- [Oxidized NADPH---hemoprotein reductase] --> rn:R08551

rn:R02469 { (-)-Limonene } <-- [Reduced NADPH---hemoprotein reductase] -- rn:R10671 <-- [Oxidized NADPH---hemoprotein reductase] --> rn:R08551

rn:R02470 { (-)-Limonene } <-- [Reduced NADPH---hemoprotein reductase] -- rn:R10671 <-- [Oxidized NADPH---hemoprotein reductase] --> rn:R08551

rn:R04366 { Linalool } <-- [Reduced NADPH---hemoprotein reductase] -- rn:R10671 <-- [Oxidized NADPH---hemoprotein reductase] --> rn:R08551

rn:R06119 { d-Limonene } <-- [Reduced NADPH---hemoprotein reductase] -- rn:R10671 <-- [Oxidized NADPH---hemoprotein reductase] --> rn:R08551

rn:R09451 { Hexadecanoic acid } <-- [Reduced NADPH---hemoprotein reductase] -- rn:R10671 <-- [Oxidized NADPH---hemoprotein reductase] --> rn:R08551

rn:R09452 { (9Z)-Octadecenoic acid } <-- [Reduced NADPH---hemoprotein reductase] -- rn:R10671 <-- [Oxidized NADPH---hemoprotein reductase] --> rn:R08551

rn:R09922 { (+)-Linalool } <-- [Reduced NADPH---hemoprotein reductase] -- rn:R10671 <-- [Oxidized NADPH---hemoprotein reductase] --> rn:R08551

rn:R09923 { (-)-Linalool } <-- [Reduced NADPH---hemoprotein reductase] -- rn:R10671 <-- [Oxidized NADPH---hemoprotein reductase] --> rn:R08551

rn:R09925 { Linalool } <-- [Reduced NADPH---hemoprotein reductase] -- rn:R10671 <-- [Oxidized NADPH---hemoprotein reductase] --> rn:R08551

rn:R09934 { Humulene } <-- [Reduced NADPH---hemoprotein reductase] -- rn:R10671 <-- [Oxidized NADPH---hemoprotein reductase] --> rn:R08551

rn:R10562 { (E,E)-Geranyllinalool } <-- [Reduced NADPH---hemoprotein reductase] -- rn:R10671 <-- [Oxidized NADPH---hemoprotein reductase] --> rn:R08551

rn:R11055 { Myrcene } <-- [Reduced NADPH---hemoprotein reductase] -- rn:R10671 <-- [Oxidized NADPH---hemoprotein reductase] --> rn:R08551

rn:R02468 { (-)-Limonene } <-- [Reduced NADPH---hemoprotein reductase] -- rn:R10671 <-- (E)-4-Hydroxyphenylacetaldehyde oxime --> rn:R10672

rn:R02469 { (-)-Limonene } <-- [Reduced NADPH---hemoprotein reductase] -- rn:R10671 <-- (E)-4-Hydroxyphenylacetaldehyde oxime --> rn:R10672

rn:R02470 { (-)-Limonene } <-- [Reduced NADPH---hemoprotein reductase] -- rn:R10671 <-- (E)-4-Hydroxyphenylacetaldehyde oxime --> rn:R10672

rn:R04366 { Linalool } <-- [Reduced NADPH---hemoprotein reductase] -- rn:R10671 <-- (E)-4-Hydroxyphenylacetaldehyde oxime --> rn:R10672

rn:R06119 { d-Limonene } <-- [Reduced NADPH---hemoprotein reductase] -- rn:R10671 <-- (E)-4-Hydroxyphenylacetaldehyde oxime --> rn:R10672

rn:R09451 { Hexadecanoic acid } <-- [Reduced NADPH---hemoprotein reductase] -- rn:R10671 <-- (E)-4-Hydroxyphenylacetaldehyde oxime --> rn:R10672

rn:R09452 { (9Z)-Octadecenoic acid } <-- [Reduced NADPH---hemoprotein reductase] -- rn:R10671 <-- (E)-4-Hydroxyphenylacetaldehyde oxime --> rn:R10672

rn:R09922 { (+)-Linalool } <-- [Reduced NADPH---hemoprotein reductase] -- rn:R10671 <-- (E)-4-Hydroxyphenylacetaldehyde oxime --> rn:R10672

rn:R09923 { (-)-Linalool } <-- [Reduced NADPH---hemoprotein reductase] -- rn:R10671 <-- (E)-4-Hydroxyphenylacetaldehyde oxime --> rn:R10672

rn:R09925 { Linalool } <-- [Reduced NADPH---hemoprotein reductase] -- rn:R10671 <-- (E)-4-Hydroxyphenylacetaldehyde oxime --> rn:R10672

rn:R09934 { Humulene } <-- [Reduced NADPH---hemoprotein reductase] -- rn:R10671 <-- (E)-4-Hydroxyphenylacetaldehyde oxime --> rn:R10672

rn:R10562 { (E,E)-Geranyllinalool } <-- [Reduced NADPH---hemoprotein reductase] -- rn:R10671 <-- (E)-4-Hydroxyphenylacetaldehyde oxime --> rn:R10672

rn:R11055 { Myrcene } <-- [Reduced NADPH---hemoprotein reductase] -- rn:R10671 <-- (E)-4-Hydroxyphenylacetaldehyde oxime --> rn:R10672

rn:R02468 { (-)-Limonene } <-- [Reduced NADPH---hemoprotein reductase] -- rn:R10671 <-- (E)-4-Hydroxyphenylacetaldehyde oxime --> rn:R11442

rn:R02469 { (-)-Limonene } <-- [Reduced NADPH---hemoprotein reductase] -- rn:R10671 <-- (E)-4-Hydroxyphenylacetaldehyde oxime --> rn:R11442

rn:R02470 { (-)-Limonene } <-- [Reduced NADPH---hemoprotein reductase] -- rn:R10671 <-- (E)-4-Hydroxyphenylacetaldehyde oxime --> rn:R11442

rn:R04366 { Linalool } <-- [Reduced NADPH---hemoprotein reductase] -- rn:R10671 <-- (E)-4-Hydroxyphenylacetaldehyde oxime --> rn:R11442

rn:R06119 { d-Limonene } <-- [Reduced NADPH---hemoprotein reductase] -- rn:R10671 <-- (E)-4-Hydroxyphenylacetaldehyde oxime --> rn:R11442

rn:R09451 { Hexadecanoic acid } <-- [Reduced NADPH---hemoprotein reductase] -- rn:R10671 <-- (E)-4-Hydroxyphenylacetaldehyde oxime --> rn:R11442

rn:R09452 { (9Z)-Octadecenoic acid } <-- [Reduced NADPH---hemoprotein reductase] -- rn:R10671 <-- (E)-4-Hydroxyphenylacetaldehyde oxime --> rn:R11442

rn:R09922 { (+)-Linalool } <-- [Reduced NADPH---hemoprotein reductase] -- rn:R10671 <-- (E)-4-Hydroxyphenylacetaldehyde oxime --> rn:R11442

rn:R09923 { (-)-Linalool } <-- [Reduced NADPH---hemoprotein reductase] -- rn:R10671 <-- (E)-4-Hydroxyphenylacetaldehyde oxime --> rn:R11442

rn:R09925 { Linalool } <-- [Reduced NADPH---hemoprotein reductase] -- rn:R10671 <-- (E)-4-Hydroxyphenylacetaldehyde oxime --> rn:R11442

rn:R09934 { Humulene } <-- [Reduced NADPH---hemoprotein reductase] -- rn:R10671 <-- (E)-4-Hydroxyphenylacetaldehyde oxime --> rn:R11442

rn:R10562 { (E,E)-Geranyllinalool } <-- [Reduced NADPH---hemoprotein reductase] -- rn:R10671 <-- (E)-4-Hydroxyphenylacetaldehyde oxime --> rn:R11442

rn:R11055 { Myrcene } <-- [Reduced NADPH---hemoprotein reductase] -- rn:R10671 <-- (E)-4-Hydroxyphenylacetaldehyde oxime --> rn:R11442

rn:R02468 { (-)-Limonene } <-- [Reduced NADPH---hemoprotein reductase] -- rn:R10728 <-- [Oxidized NADPH---hemoprotein reductase] --> rn:R08551

rn:R02469 { (-)-Limonene } <-- [Reduced NADPH---hemoprotein reductase] -- rn:R10728 <-- [Oxidized NADPH---hemoprotein reductase] --> rn:R08551

rn:R02470 { (-)-Limonene } <-- [Reduced NADPH---hemoprotein reductase] -- rn:R10728 <-- [Oxidized NADPH---hemoprotein reductase] --> rn:R08551

rn:R04366 { Linalool } <-- [Reduced NADPH---hemoprotein reductase] -- rn:R10728 <-- [Oxidized NADPH---hemoprotein reductase] --> rn:R08551

rn:R06119 { d-Limonene } <-- [Reduced NADPH---hemoprotein reductase] -- rn:R10728 <-- [Oxidized NADPH---hemoprotein reductase] --> rn:R08551

rn:R09451 { Hexadecanoic acid } <-- [Reduced NADPH---hemoprotein reductase] -- rn:R10728 <-- [Oxidized NADPH---hemoprotein reductase] --> rn:R08551

rn:R09452 { (9Z)-Octadecenoic acid } <-- [Reduced NADPH---hemoprotein reductase] -- rn:R10728 <-- [Oxidized NADPH---hemoprotein reductase] --> rn:R08551

rn:R09922 { (+)-Linalool } <-- [Reduced NADPH---hemoprotein reductase] -- rn:R10728 <-- [Oxidized NADPH---hemoprotein reductase] --> rn:R08551

rn:R09923 { (-)-Linalool } <-- [Reduced NADPH---hemoprotein reductase] -- rn:R10728 <-- [Oxidized NADPH---hemoprotein reductase] --> rn:R08551

rn:R09925 { Linalool } <-- [Reduced NADPH---hemoprotein reductase] -- rn:R10728 <-- [Oxidized NADPH---hemoprotein reductase] --> rn:R08551

rn:R09934 { Humulene } <-- [Reduced NADPH---hemoprotein reductase] -- rn:R10728 <-- [Oxidized NADPH---hemoprotein reductase] --> rn:R08551

rn:R10562 { (E,E)-Geranyllinalool } <-- [Reduced NADPH---hemoprotein reductase] -- rn:R10728 <-- [Oxidized NADPH---hemoprotein reductase] --> rn:R08551

rn:R11055 { Myrcene } <-- [Reduced NADPH---hemoprotein reductase] -- rn:R10728 <-- [Oxidized NADPH---hemoprotein reductase] --> rn:R08551

rn:R08530 { (-)-Menthol } <-- Acetyl-CoA -- rn:R10746 <-- Epithienamycin F --> rn:R10741

rn:R08531 { (+)-Neomenthol } <-- Acetyl-CoA -- rn:R10746 <-- Epithienamycin F --> rn:R10741

rn:R08532 { (+)-Borneol } <-- Acetyl-CoA -- rn:R10746 <-- Epithienamycin F --> rn:R10741

rn:R10474 { Cinnamyl alcohol } <-- Acetyl-CoA -- rn:R10746 <-- Epithienamycin F --> rn:R10741

rn:R02468 { (-)-Limonene } <-- [Reduced NADPH---hemoprotein reductase] -- rn:R10795 <-- [Oxidized NADPH---hemoprotein reductase] --> rn:R08551

rn:R02469 { (-)-Limonene } <-- [Reduced NADPH---hemoprotein reductase] -- rn:R10795 <-- [Oxidized NADPH---hemoprotein reductase] --> rn:R08551

rn:R02470 { (-)-Limonene } <-- [Reduced NADPH---hemoprotein reductase] -- rn:R10795 <-- [Oxidized NADPH---hemoprotein reductase] --> rn:R08551

rn:R04366 { Linalool } <-- [Reduced NADPH---hemoprotein reductase] -- rn:R10795 <-- [Oxidized NADPH---hemoprotein reductase] --> rn:R08551

rn:R06119 { d-Limonene } <-- [Reduced NADPH---hemoprotein reductase] -- rn:R10795 <-- [Oxidized NADPH---hemoprotein reductase] --> rn:R08551

rn:R09451 { Hexadecanoic acid } <-- [Reduced NADPH---hemoprotein reductase] -- rn:R10795 <-- [Oxidized NADPH---hemoprotein reductase] --> rn:R08551

rn:R09452 { (9Z)-Octadecenoic acid } <-- [Reduced NADPH---hemoprotein reductase] -- rn:R10795 <-- [Oxidized NADPH---hemoprotein reductase] --> rn:R08551

rn:R09922 { (+)-Linalool } <-- [Reduced NADPH---hemoprotein reductase] -- rn:R10795 <-- [Oxidized NADPH---hemoprotein reductase] --> rn:R08551

rn:R09923 { (-)-Linalool } <-- [Reduced NADPH---hemoprotein reductase] -- rn:R10795 <-- [Oxidized NADPH---hemoprotein reductase] --> rn:R08551

rn:R09925 { Linalool } <-- [Reduced NADPH---hemoprotein reductase] -- rn:R10795 <-- [Oxidized NADPH---hemoprotein reductase] --> rn:R08551

rn:R09934 { Humulene } <-- [Reduced NADPH---hemoprotein reductase] -- rn:R10795 <-- [Oxidized NADPH---hemoprotein reductase] --> rn:R08551

rn:R10562 { (E,E)-Geranyllinalool } <-- [Reduced NADPH---hemoprotein reductase] -- rn:R10795 <-- [Oxidized NADPH---hemoprotein reductase] --> rn:R08551

rn:R11055 { Myrcene } <-- [Reduced NADPH---hemoprotein reductase] -- rn:R10795 <-- [Oxidized NADPH---hemoprotein reductase] --> rn:R08551

rn:R02468 { (-)-Limonene } <-- [Reduced NADPH---hemoprotein reductase] -- rn:R10999 <-- [Oxidized NADPH---hemoprotein reductase] --> rn:R08551

rn:R02469 { (-)-Limonene } <-- [Reduced NADPH---hemoprotein reductase] -- rn:R10999 <-- [Oxidized NADPH---hemoprotein reductase] --> rn:R08551

rn:R02470 { (-)-Limonene } <-- [Reduced NADPH---hemoprotein reductase] -- rn:R10999 <-- [Oxidized NADPH---hemoprotein reductase] --> rn:R08551

rn:R04366 { Linalool } <-- [Reduced NADPH---hemoprotein reductase] -- rn:R10999 <-- [Oxidized NADPH---hemoprotein reductase] --> rn:R08551

rn:R06119 { d-Limonene } <-- [Reduced NADPH---hemoprotein reductase] -- rn:R10999 <-- [Oxidized NADPH---hemoprotein reductase] --> rn:R08551

rn:R09451 { Hexadecanoic acid } <-- [Reduced NADPH---hemoprotein reductase] -- rn:R10999 <-- [Oxidized NADPH---hemoprotein reductase] --> rn:R08551

rn:R09452 { (9Z)-Octadecenoic acid } <-- [Reduced NADPH---hemoprotein reductase] -- rn:R10999 <-- [Oxidized NADPH---hemoprotein reductase] --> rn:R08551

rn:R09922 { (+)-Linalool } <-- [Reduced NADPH---hemoprotein reductase] -- rn:R10999 <-- [Oxidized NADPH---hemoprotein reductase] --> rn:R08551

rn:R09923 { (-)-Linalool } <-- [Reduced NADPH---hemoprotein reductase] -- rn:R10999 <-- [Oxidized NADPH---hemoprotein reductase] --> rn:R08551

rn:R09925 { Linalool } <-- [Reduced NADPH---hemoprotein reductase] -- rn:R10999 <-- [Oxidized NADPH---hemoprotein reductase] --> rn:R08551

rn:R09934 { Humulene } <-- [Reduced NADPH---hemoprotein reductase] -- rn:R10999 <-- [Oxidized NADPH---hemoprotein reductase] --> rn:R08551

rn:R10562 { (E,E)-Geranyllinalool } <-- [Reduced NADPH---hemoprotein reductase] -- rn:R10999 <-- [Oxidized NADPH---hemoprotein reductase] --> rn:R08551

rn:R11055 { Myrcene } <-- [Reduced NADPH---hemoprotein reductase] -- rn:R10999 <-- [Oxidized NADPH---hemoprotein reductase] --> rn:R08551

rn:R02468 { (-)-Limonene } <-- [Reduced NADPH---hemoprotein reductase] -- rn:R11000 <-- [Oxidized NADPH---hemoprotein reductase] --> rn:R08551

rn:R02469 { (-)-Limonene } <-- [Reduced NADPH---hemoprotein reductase] -- rn:R11000 <-- [Oxidized NADPH---hemoprotein reductase] --> rn:R08551

rn:R02470 { (-)-Limonene } <-- [Reduced NADPH---hemoprotein reductase] -- rn:R11000 <-- [Oxidized NADPH---hemoprotein reductase] --> rn:R08551

rn:R04366 { Linalool } <-- [Reduced NADPH---hemoprotein reductase] -- rn:R11000 <-- [Oxidized NADPH---hemoprotein reductase] --> rn:R08551

rn:R06119 { d-Limonene } <-- [Reduced NADPH---hemoprotein reductase] -- rn:R11000 <-- [Oxidized NADPH---hemoprotein reductase] --> rn:R08551

rn:R09451 { Hexadecanoic acid } <-- [Reduced NADPH---hemoprotein reductase] -- rn:R11000 <-- [Oxidized NADPH---hemoprotein reductase] --> rn:R08551

rn:R09452 { (9Z)-Octadecenoic acid } <-- [Reduced NADPH---hemoprotein reductase] -- rn:R11000 <-- [Oxidized NADPH---hemoprotein reductase] --> rn:R08551

rn:R09922 { (+)-Linalool } <-- [Reduced NADPH---hemoprotein reductase] -- rn:R11000 <-- [Oxidized NADPH---hemoprotein reductase] --> rn:R08551

rn:R09923 { (-)-Linalool } <-- [Reduced NADPH---hemoprotein reductase] -- rn:R11000 <-- [Oxidized NADPH---hemoprotein reductase] --> rn:R08551

rn:R09925 { Linalool } <-- [Reduced NADPH---hemoprotein reductase] -- rn:R11000 <-- [Oxidized NADPH---hemoprotein reductase] --> rn:R08551

rn:R09934 { Humulene } <-- [Reduced NADPH---hemoprotein reductase] -- rn:R11000 <-- [Oxidized NADPH---hemoprotein reductase] --> rn:R08551

rn:R10562 { (E,E)-Geranyllinalool } <-- [Reduced NADPH---hemoprotein reductase] -- rn:R11000 <-- [Oxidized NADPH---hemoprotein reductase] --> rn:R08551

rn:R11055 { Myrcene } <-- [Reduced NADPH---hemoprotein reductase] -- rn:R11000 <-- [Oxidized NADPH---hemoprotein reductase] --> rn:R08551

rn:R08530 { (-)-Menthol } <-- Acetyl-CoA -- rn:R11124 <-- 5-Methyl-1-naphthoate --> rn:R10725

rn:R08531 { (+)-Neomenthol } <-- Acetyl-CoA -- rn:R11124 <-- 5-Methyl-1-naphthoate --> rn:R10725

rn:R08532 { (+)-Borneol } <-- Acetyl-CoA -- rn:R11124 <-- 5-Methyl-1-naphthoate --> rn:R10725

rn:R10474 { Cinnamyl alcohol } <-- Acetyl-CoA -- rn:R11124 <-- 5-Methyl-1-naphthoate --> rn:R10725

rn:R05537 { 3-(2-Hydroxyphenyl)propanoate } <-- FAD -- rn:R11130 <-- FADH2 --> rn:R03978

rn:R05537 { 3-(2-Hydroxyphenyl)propanoate } <-- FAD -- rn:R11130 <-- FADH2 --> rn:R05488

rn:R05537 { 3-(2-Hydroxyphenyl)propanoate } <-- FAD -- rn:R11130 <-- FADH2 --> rn:R09517

rn:R05537 { 3-(2-Hydroxyphenyl)propanoate } <-- FAD -- rn:R11130 <-- 3-(Methylthio)acryloyl-CoA --> rn:R10936

rn:R05537 { 3-(2-Hydroxyphenyl)propanoate } <-- FAD -- rn:R11130 <-- FADH2 --> rn:R11653

rn:R05537 { 3-(2-Hydroxyphenyl)propanoate } <-- FAD -- rn:R11130 <-- FADH2 --> rn:R12021

rn:R05537 { 3-(2-Hydroxyphenyl)propanoate } <-- FAD -- rn:R11130 <-- FADH2 --> rn:R12023

rn:R05537 { 3-(2-Hydroxyphenyl)propanoate } <-- FAD -- rn:R11130 <-- FADH2 --> rn:R12027

rn:R05537 { 3-(2-Hydroxyphenyl)propanoate } <-- FAD -- rn:R11130 <-- FADH2 --> rn:R12030

rn:R06404 { alpha-Pinene } <-- e- -- rn:R11293 <-- Cob(II)alamin --> rn:R00097

rn:R06404 { alpha-Pinene } <-- e- -- rn:R11293 <-- Gentamicin X2 --> rn:R11294

rn:R06404 { alpha-Pinene } <-- e- -- rn:R11293 <-- L-Methionine --> rn:R12055

rn:R06404 { alpha-Pinene } <-- e- -- rn:R11293 <-- Cob(II)alamin --> rn:R12183

rn:R06404 { alpha-Pinene } <-- e- -- rn:R11294 <-- Cob(II)alamin --> rn:R00097

rn:R06404 { alpha-Pinene } <-- e- -- rn:R11294 <-- L-Methionine --> rn:R12055

rn:R06404 { alpha-Pinene } <-- e- -- rn:R11294 <-- Cob(II)alamin --> rn:R12183

rn:R08530 { (-)-Menthol } <-- Acetyl-CoA -- rn:R11479 <-- 2-Acetamidoethylphosphonate --> rn:R11480

rn:R08531 { (+)-Neomenthol } <-- Acetyl-CoA -- rn:R11479 <-- 2-Acetamidoethylphosphonate --> rn:R11480

rn:R08532 { (+)-Borneol } <-- Acetyl-CoA -- rn:R11479 <-- 2-Acetamidoethylphosphonate --> rn:R11480

rn:R10474 { Cinnamyl alcohol } <-- Acetyl-CoA -- rn:R11479 <-- 2-Acetamidoethylphosphonate --> rn:R11480

rn:R01703 { Hexadecanoic acid } <-- Hydrogen peroxide -- rn:R11503 <-- 4-Hydroxy-3-methoxy-benzaldehyde --> rn:R05699

rn:R01703 { Hexadecanoic acid } <-- Hydrogen peroxide -- rn:R11503 <-- Glycolaldehyde --> rn:R05840

rn:R01703 { Hexadecanoic acid } <-- Hydrogen peroxide -- rn:R11503 <-- 4-Hydroxy-3-methoxy-benzaldehyde --> rn:R08461

rn:R01703 { Hexadecanoic acid } <-- Hydrogen peroxide -- rn:R11522 <-- Heme --> rn:R00311

rn:R01703 { Hexadecanoic acid } <-- Hydrogen peroxide -- rn:R11522 <-- Heme --> rn:R00312

rn:R01703 { Hexadecanoic acid } <-- Hydrogen peroxide -- rn:R11522 <-- Heme --> rn:R00313

rn:R01703 { Hexadecanoic acid } <-- Hydrogen peroxide -- rn:R11522 <-- Heme --> rn:R00314

rn:R01703 { Hexadecanoic acid } <-- Hydrogen peroxide -- rn:R11522 <-- Heme --> rn:R07411

rn:R01703 { Hexadecanoic acid } <-- Hydrogen peroxide -- rn:R11522 <-- Heme --> rn:R11579

rn:R02468 { (-)-Limonene } <-- [Reduced NADPH---hemoprotein reductase] -- rn:R11597 <-- [Oxidized NADPH---hemoprotein reductase] --> rn:R08551

rn:R02469 { (-)-Limonene } <-- [Reduced NADPH---hemoprotein reductase] -- rn:R11597 <-- [Oxidized NADPH---hemoprotein reductase] --> rn:R08551

rn:R02470 { (-)-Limonene } <-- [Reduced NADPH---hemoprotein reductase] -- rn:R11597 <-- [Oxidized NADPH---hemoprotein reductase] --> rn:R08551

rn:R04366 { Linalool } <-- [Reduced NADPH---hemoprotein reductase] -- rn:R11597 <-- [Oxidized NADPH---hemoprotein reductase] --> rn:R08551

rn:R06119 { d-Limonene } <-- [Reduced NADPH---hemoprotein reductase] -- rn:R11597 <-- [Oxidized NADPH---hemoprotein reductase] --> rn:R08551

rn:R09451 { Hexadecanoic acid } <-- [Reduced NADPH---hemoprotein reductase] -- rn:R11597 <-- [Oxidized NADPH---hemoprotein reductase] --> rn:R08551

rn:R09452 { (9Z)-Octadecenoic acid } <-- [Reduced NADPH---hemoprotein reductase] -- rn:R11597 <-- [Oxidized NADPH---hemoprotein reductase] --> rn:R08551

rn:R09922 { (+)-Linalool } <-- [Reduced NADPH---hemoprotein reductase] -- rn:R11597 <-- [Oxidized NADPH---hemoprotein reductase] --> rn:R08551

rn:R09923 { (-)-Linalool } <-- [Reduced NADPH---hemoprotein reductase] -- rn:R11597 <-- [Oxidized NADPH---hemoprotein reductase] --> rn:R08551

rn:R09925 { Linalool } <-- [Reduced NADPH---hemoprotein reductase] -- rn:R11597 <-- [Oxidized NADPH---hemoprotein reductase] --> rn:R08551

rn:R09934 { Humulene } <-- [Reduced NADPH---hemoprotein reductase] -- rn:R11597 <-- [Oxidized NADPH---hemoprotein reductase] --> rn:R08551

rn:R10562 { (E,E)-Geranyllinalool } <-- [Reduced NADPH---hemoprotein reductase] -- rn:R11597 <-- [Oxidized NADPH---hemoprotein reductase] --> rn:R08551

rn:R11055 { Myrcene } <-- [Reduced NADPH---hemoprotein reductase] -- rn:R11597 <-- [Oxidized NADPH---hemoprotein reductase] --> rn:R08551

rn:R02468 { (-)-Limonene } <-- [Reduced NADPH---hemoprotein reductase] -- rn:R11597 <-- (2R)-2-Hydroxy-2-methylbutanenitrile --> rn:R09358

rn:R02469 { (-)-Limonene } <-- [Reduced NADPH---hemoprotein reductase] -- rn:R11597 <-- (2R)-2-Hydroxy-2-methylbutanenitrile --> rn:R09358

rn:R02470 { (-)-Limonene } <-- [Reduced NADPH---hemoprotein reductase] -- rn:R11597 <-- (2R)-2-Hydroxy-2-methylbutanenitrile --> rn:R09358

rn:R04366 { Linalool } <-- [Reduced NADPH---hemoprotein reductase] -- rn:R11597 <-- (2R)-2-Hydroxy-2-methylbutanenitrile --> rn:R09358

rn:R06119 { d-Limonene } <-- [Reduced NADPH---hemoprotein reductase] -- rn:R11597 <-- (2R)-2-Hydroxy-2-methylbutanenitrile --> rn:R09358

rn:R09451 { Hexadecanoic acid } <-- [Reduced NADPH---hemoprotein reductase] -- rn:R11597 <-- (2R)-2-Hydroxy-2-methylbutanenitrile --> rn:R09358

rn:R09452 { (9Z)-Octadecenoic acid } <-- [Reduced NADPH---hemoprotein reductase] -- rn:R11597 <-- (2R)-2-Hydroxy-2-methylbutanenitrile --> rn:R09358

rn:R09922 { (+)-Linalool } <-- [Reduced NADPH---hemoprotein reductase] -- rn:R11597 <-- (2R)-2-Hydroxy-2-methylbutanenitrile --> rn:R09358

rn:R09923 { (-)-Linalool } <-- [Reduced NADPH---hemoprotein reductase] -- rn:R11597 <-- (2R)-2-Hydroxy-2-methylbutanenitrile --> rn:R09358

rn:R09925 { Linalool } <-- [Reduced NADPH---hemoprotein reductase] -- rn:R11597 <-- (2R)-2-Hydroxy-2-methylbutanenitrile --> rn:R09358

rn:R09934 { Humulene } <-- [Reduced NADPH---hemoprotein reductase] -- rn:R11597 <-- (2R)-2-Hydroxy-2-methylbutanenitrile --> rn:R09358

rn:R10562 { (E,E)-Geranyllinalool } <-- [Reduced NADPH---hemoprotein reductase] -- rn:R11597 <-- (2R)-2-Hydroxy-2-methylbutanenitrile --> rn:R09358

rn:R11055 { Myrcene } <-- [Reduced NADPH---hemoprotein reductase] -- rn:R11597 <-- (2R)-2-Hydroxy-2-methylbutanenitrile --> rn:R09358

rn:R02468 { (-)-Limonene } <-- [Reduced NADPH---hemoprotein reductase] -- rn:R11597 <-- (2R)-2-Hydroxy-2-methylbutanenitrile --> rn:R10037

rn:R02469 { (-)-Limonene } <-- [Reduced NADPH---hemoprotein reductase] -- rn:R11597 <-- (2R)-2-Hydroxy-2-methylbutanenitrile --> rn:R10037

rn:R02470 { (-)-Limonene } <-- [Reduced NADPH---hemoprotein reductase] -- rn:R11597 <-- (2R)-2-Hydroxy-2-methylbutanenitrile --> rn:R10037

rn:R04366 { Linalool } <-- [Reduced NADPH---hemoprotein reductase] -- rn:R11597 <-- (2R)-2-Hydroxy-2-methylbutanenitrile --> rn:R10037

rn:R06119 { d-Limonene } <-- [Reduced NADPH---hemoprotein reductase] -- rn:R11597 <-- (2R)-2-Hydroxy-2-methylbutanenitrile --> rn:R10037

rn:R09451 { Hexadecanoic acid } <-- [Reduced NADPH---hemoprotein reductase] -- rn:R11597 <-- (2R)-2-Hydroxy-2-methylbutanenitrile --> rn:R10037

rn:R09452 { (9Z)-Octadecenoic acid } <-- [Reduced NADPH---hemoprotein reductase] -- rn:R11597 <-- (2R)-2-Hydroxy-2-methylbutanenitrile --> rn:R10037

rn:R09922 { (+)-Linalool } <-- [Reduced NADPH---hemoprotein reductase] -- rn:R11597 <-- (2R)-2-Hydroxy-2-methylbutanenitrile --> rn:R10037

rn:R09923 { (-)-Linalool } <-- [Reduced NADPH---hemoprotein reductase] -- rn:R11597 <-- (2R)-2-Hydroxy-2-methylbutanenitrile --> rn:R10037

rn:R09925 { Linalool } <-- [Reduced NADPH---hemoprotein reductase] -- rn:R11597 <-- (2R)-2-Hydroxy-2-methylbutanenitrile --> rn:R10037

rn:R09934 { Humulene } <-- [Reduced NADPH---hemoprotein reductase] -- rn:R11597 <-- (2R)-2-Hydroxy-2-methylbutanenitrile --> rn:R10037

rn:R10562 { (E,E)-Geranyllinalool } <-- [Reduced NADPH---hemoprotein reductase] -- rn:R11597 <-- (2R)-2-Hydroxy-2-methylbutanenitrile --> rn:R10037

rn:R11055 { Myrcene } <-- [Reduced NADPH---hemoprotein reductase] -- rn:R11597 <-- (2R)-2-Hydroxy-2-methylbutanenitrile --> rn:R10037

rn:R02468 { (-)-Limonene } <-- [Reduced NADPH---hemoprotein reductase] -- rn:R11598 <-- Acetone cyanohydrin --> rn:R01553

rn:R02469 { (-)-Limonene } <-- [Reduced NADPH---hemoprotein reductase] -- rn:R11598 <-- Acetone cyanohydrin --> rn:R01553

rn:R02470 { (-)-Limonene } <-- [Reduced NADPH---hemoprotein reductase] -- rn:R11598 <-- Acetone cyanohydrin --> rn:R01553

rn:R04366 { Linalool } <-- [Reduced NADPH---hemoprotein reductase] -- rn:R11598 <-- Acetone cyanohydrin --> rn:R01553

rn:R06119 { d-Limonene } <-- [Reduced NADPH---hemoprotein reductase] -- rn:R11598 <-- Acetone cyanohydrin --> rn:R01553

rn:R09451 { Hexadecanoic acid } <-- [Reduced NADPH---hemoprotein reductase] -- rn:R11598 <-- Acetone cyanohydrin --> rn:R01553

rn:R09452 { (9Z)-Octadecenoic acid } <-- [Reduced NADPH---hemoprotein reductase] -- rn:R11598 <-- Acetone cyanohydrin --> rn:R01553

rn:R09922 { (+)-Linalool } <-- [Reduced NADPH---hemoprotein reductase] -- rn:R11598 <-- Acetone cyanohydrin --> rn:R01553

rn:R09923 { (-)-Linalool } <-- [Reduced NADPH---hemoprotein reductase] -- rn:R11598 <-- Acetone cyanohydrin --> rn:R01553

rn:R09925 { Linalool } <-- [Reduced NADPH---hemoprotein reductase] -- rn:R11598 <-- Acetone cyanohydrin --> rn:R01553

rn:R09934 { Humulene } <-- [Reduced NADPH---hemoprotein reductase] -- rn:R11598 <-- Acetone cyanohydrin --> rn:R01553

rn:R10562 { (E,E)-Geranyllinalool } <-- [Reduced NADPH---hemoprotein reductase] -- rn:R11598 <-- Acetone cyanohydrin --> rn:R01553

rn:R11055 { Myrcene } <-- [Reduced NADPH---hemoprotein reductase] -- rn:R11598 <-- Acetone cyanohydrin --> rn:R01553

rn:R02468 { (-)-Limonene } <-- [Reduced NADPH---hemoprotein reductase] -- rn:R11598 <-- Acetone cyanohydrin --> rn:R03625

rn:R02469 { (-)-Limonene } <-- [Reduced NADPH---hemoprotein reductase] -- rn:R11598 <-- Acetone cyanohydrin --> rn:R03625

rn:R02470 { (-)-Limonene } <-- [Reduced NADPH---hemoprotein reductase] -- rn:R11598 <-- Acetone cyanohydrin --> rn:R03625

rn:R04366 { Linalool } <-- [Reduced NADPH---hemoprotein reductase] -- rn:R11598 <-- Acetone cyanohydrin --> rn:R03625

rn:R06119 { d-Limonene } <-- [Reduced NADPH---hemoprotein reductase] -- rn:R11598 <-- Acetone cyanohydrin --> rn:R03625

rn:R09451 { Hexadecanoic acid } <-- [Reduced NADPH---hemoprotein reductase] -- rn:R11598 <-- Acetone cyanohydrin --> rn:R03625

rn:R09452 { (9Z)-Octadecenoic acid } <-- [Reduced NADPH---hemoprotein reductase] -- rn:R11598 <-- Acetone cyanohydrin --> rn:R03625

rn:R09922 { (+)-Linalool } <-- [Reduced NADPH---hemoprotein reductase] -- rn:R11598 <-- Acetone cyanohydrin --> rn:R03625

rn:R09923 { (-)-Linalool } <-- [Reduced NADPH---hemoprotein reductase] -- rn:R11598 <-- Acetone cyanohydrin --> rn:R03625

rn:R09925 { Linalool } <-- [Reduced NADPH---hemoprotein reductase] -- rn:R11598 <-- Acetone cyanohydrin --> rn:R03625

rn:R09934 { Humulene } <-- [Reduced NADPH---hemoprotein reductase] -- rn:R11598 <-- Acetone cyanohydrin --> rn:R03625

rn:R10562 { (E,E)-Geranyllinalool } <-- [Reduced NADPH---hemoprotein reductase] -- rn:R11598 <-- Acetone cyanohydrin --> rn:R03625

rn:R11055 { Myrcene } <-- [Reduced NADPH---hemoprotein reductase] -- rn:R11598 <-- Acetone cyanohydrin --> rn:R03625

rn:R02468 { (-)-Limonene } <-- [Reduced NADPH---hemoprotein reductase] -- rn:R11598 <-- [Oxidized NADPH---hemoprotein reductase] --> rn:R08551

rn:R02469 { (-)-Limonene } <-- [Reduced NADPH---hemoprotein reductase] -- rn:R11598 <-- [Oxidized NADPH---hemoprotein reductase] --> rn:R08551

rn:R02470 { (-)-Limonene } <-- [Reduced NADPH---hemoprotein reductase] -- rn:R11598 <-- [Oxidized NADPH---hemoprotein reductase] --> rn:R08551

rn:R04366 { Linalool } <-- [Reduced NADPH---hemoprotein reductase] -- rn:R11598 <-- [Oxidized NADPH---hemoprotein reductase] --> rn:R08551

rn:R06119 { d-Limonene } <-- [Reduced NADPH---hemoprotein reductase] -- rn:R11598 <-- [Oxidized NADPH---hemoprotein reductase] --> rn:R08551

rn:R09451 { Hexadecanoic acid } <-- [Reduced NADPH---hemoprotein reductase] -- rn:R11598 <-- [Oxidized NADPH---hemoprotein reductase] --> rn:R08551

rn:R09452 { (9Z)-Octadecenoic acid } <-- [Reduced NADPH---hemoprotein reductase] -- rn:R11598 <-- [Oxidized NADPH---hemoprotein reductase] --> rn:R08551

rn:R09922 { (+)-Linalool } <-- [Reduced NADPH---hemoprotein reductase] -- rn:R11598 <-- [Oxidized NADPH---hemoprotein reductase] --> rn:R08551

rn:R09923 { (-)-Linalool } <-- [Reduced NADPH---hemoprotein reductase] -- rn:R11598 <-- [Oxidized NADPH---hemoprotein reductase] --> rn:R08551

rn:R09925 { Linalool } <-- [Reduced NADPH---hemoprotein reductase] -- rn:R11598 <-- [Oxidized NADPH---hemoprotein reductase] --> rn:R08551

rn:R09934 { Humulene } <-- [Reduced NADPH---hemoprotein reductase] -- rn:R11598 <-- [Oxidized NADPH---hemoprotein reductase] --> rn:R08551

rn:R10562 { (E,E)-Geranyllinalool } <-- [Reduced NADPH---hemoprotein reductase] -- rn:R11598 <-- [Oxidized NADPH---hemoprotein reductase] --> rn:R08551

rn:R11055 { Myrcene } <-- [Reduced NADPH---hemoprotein reductase] -- rn:R11598 <-- [Oxidized NADPH---hemoprotein reductase] --> rn:R08551

rn:R02468 { (-)-Limonene } <-- [Reduced NADPH---hemoprotein reductase] -- rn:R11640 <-- [Oxidized NADPH---hemoprotein reductase] --> rn:R08551

rn:R02469 { (-)-Limonene } <-- [Reduced NADPH---hemoprotein reductase] -- rn:R11640 <-- [Oxidized NADPH---hemoprotein reductase] --> rn:R08551

rn:R02470 { (-)-Limonene } <-- [Reduced NADPH---hemoprotein reductase] -- rn:R11640 <-- [Oxidized NADPH---hemoprotein reductase] --> rn:R08551

rn:R04366 { Linalool } <-- [Reduced NADPH---hemoprotein reductase] -- rn:R11640 <-- [Oxidized NADPH---hemoprotein reductase] --> rn:R08551

rn:R06119 { d-Limonene } <-- [Reduced NADPH---hemoprotein reductase] -- rn:R11640 <-- [Oxidized NADPH---hemoprotein reductase] --> rn:R08551

rn:R09451 { Hexadecanoic acid } <-- [Reduced NADPH---hemoprotein reductase] -- rn:R11640 <-- [Oxidized NADPH---hemoprotein reductase] --> rn:R08551

rn:R09452 { (9Z)-Octadecenoic acid } <-- [Reduced NADPH---hemoprotein reductase] -- rn:R11640 <-- [Oxidized NADPH---hemoprotein reductase] --> rn:R08551

rn:R09922 { (+)-Linalool } <-- [Reduced NADPH---hemoprotein reductase] -- rn:R11640 <-- [Oxidized NADPH---hemoprotein reductase] --> rn:R08551

rn:R09923 { (-)-Linalool } <-- [Reduced NADPH---hemoprotein reductase] -- rn:R11640 <-- [Oxidized NADPH---hemoprotein reductase] --> rn:R08551

rn:R09925 { Linalool } <-- [Reduced NADPH---hemoprotein reductase] -- rn:R11640 <-- [Oxidized NADPH---hemoprotein reductase] --> rn:R08551

rn:R09934 { Humulene } <-- [Reduced NADPH---hemoprotein reductase] -- rn:R11640 <-- [Oxidized NADPH---hemoprotein reductase] --> rn:R08551

rn:R10562 { (E,E)-Geranyllinalool } <-- [Reduced NADPH---hemoprotein reductase] -- rn:R11640 <-- [Oxidized NADPH---hemoprotein reductase] --> rn:R08551

rn:R11055 { Myrcene } <-- [Reduced NADPH---hemoprotein reductase] -- rn:R11640 <-- [Oxidized NADPH---hemoprotein reductase] --> rn:R08551

rn:R02468 { (-)-Limonene } <-- [Reduced NADPH---hemoprotein reductase] -- rn:R11640 <-- (2R)-2-Hydroxy-2-methylbutanenitrile --> rn:R09358

rn:R02469 { (-)-Limonene } <-- [Reduced NADPH---hemoprotein reductase] -- rn:R11640 <-- (2R)-2-Hydroxy-2-methylbutanenitrile --> rn:R09358

rn:R02470 { (-)-Limonene } <-- [Reduced NADPH---hemoprotein reductase] -- rn:R11640 <-- (2R)-2-Hydroxy-2-methylbutanenitrile --> rn:R09358

rn:R04366 { Linalool } <-- [Reduced NADPH---hemoprotein reductase] -- rn:R11640 <-- (2R)-2-Hydroxy-2-methylbutanenitrile --> rn:R09358

rn:R06119 { d-Limonene } <-- [Reduced NADPH---hemoprotein reductase] -- rn:R11640 <-- (2R)-2-Hydroxy-2-methylbutanenitrile --> rn:R09358

rn:R09451 { Hexadecanoic acid } <-- [Reduced NADPH---hemoprotein reductase] -- rn:R11640 <-- (2R)-2-Hydroxy-2-methylbutanenitrile --> rn:R09358

rn:R09452 { (9Z)-Octadecenoic acid } <-- [Reduced NADPH---hemoprotein reductase] -- rn:R11640 <-- (2R)-2-Hydroxy-2-methylbutanenitrile --> rn:R09358

rn:R09922 { (+)-Linalool } <-- [Reduced NADPH---hemoprotein reductase] -- rn:R11640 <-- (2R)-2-Hydroxy-2-methylbutanenitrile --> rn:R09358

rn:R09923 { (-)-Linalool } <-- [Reduced NADPH---hemoprotein reductase] -- rn:R11640 <-- (2R)-2-Hydroxy-2-methylbutanenitrile --> rn:R09358

rn:R09925 { Linalool } <-- [Reduced NADPH---hemoprotein reductase] -- rn:R11640 <-- (2R)-2-Hydroxy-2-methylbutanenitrile --> rn:R09358

rn:R09934 { Humulene } <-- [Reduced NADPH---hemoprotein reductase] -- rn:R11640 <-- (2R)-2-Hydroxy-2-methylbutanenitrile --> rn:R09358

rn:R10562 { (E,E)-Geranyllinalool } <-- [Reduced NADPH---hemoprotein reductase] -- rn:R11640 <-- (2R)-2-Hydroxy-2-methylbutanenitrile --> rn:R09358

rn:R11055 { Myrcene } <-- [Reduced NADPH---hemoprotein reductase] -- rn:R11640 <-- (2R)-2-Hydroxy-2-methylbutanenitrile --> rn:R09358

rn:R02468 { (-)-Limonene } <-- [Reduced NADPH---hemoprotein reductase] -- rn:R11640 <-- (2R)-2-Hydroxy-2-methylbutanenitrile --> rn:R10037

rn:R02469 { (-)-Limonene } <-- [Reduced NADPH---hemoprotein reductase] -- rn:R11640 <-- (2R)-2-Hydroxy-2-methylbutanenitrile --> rn:R10037

rn:R02470 { (-)-Limonene } <-- [Reduced NADPH---hemoprotein reductase] -- rn:R11640 <-- (2R)-2-Hydroxy-2-methylbutanenitrile --> rn:R10037

rn:R04366 { Linalool } <-- [Reduced NADPH---hemoprotein reductase] -- rn:R11640 <-- (2R)-2-Hydroxy-2-methylbutanenitrile --> rn:R10037

rn:R06119 { d-Limonene } <-- [Reduced NADPH---hemoprotein reductase] -- rn:R11640 <-- (2R)-2-Hydroxy-2-methylbutanenitrile --> rn:R10037

rn:R09451 { Hexadecanoic acid } <-- [Reduced NADPH---hemoprotein reductase] -- rn:R11640 <-- (2R)-2-Hydroxy-2-methylbutanenitrile --> rn:R10037

rn:R09452 { (9Z)-Octadecenoic acid } <-- [Reduced NADPH---hemoprotein reductase] -- rn:R11640 <-- (2R)-2-Hydroxy-2-methylbutanenitrile --> rn:R10037

rn:R09922 { (+)-Linalool } <-- [Reduced NADPH---hemoprotein reductase] -- rn:R11640 <-- (2R)-2-Hydroxy-2-methylbutanenitrile --> rn:R10037

rn:R09923 { (-)-Linalool } <-- [Reduced NADPH---hemoprotein reductase] -- rn:R11640 <-- (2R)-2-Hydroxy-2-methylbutanenitrile --> rn:R10037

rn:R09925 { Linalool } <-- [Reduced NADPH---hemoprotein reductase] -- rn:R11640 <-- (2R)-2-Hydroxy-2-methylbutanenitrile --> rn:R10037

rn:R09934 { Humulene } <-- [Reduced NADPH---hemoprotein reductase] -- rn:R11640 <-- (2R)-2-Hydroxy-2-methylbutanenitrile --> rn:R10037

rn:R10562 { (E,E)-Geranyllinalool } <-- [Reduced NADPH---hemoprotein reductase] -- rn:R11640 <-- (2R)-2-Hydroxy-2-methylbutanenitrile --> rn:R10037

rn:R11055 { Myrcene } <-- [Reduced NADPH---hemoprotein reductase] -- rn:R11640 <-- (2R)-2-Hydroxy-2-methylbutanenitrile --> rn:R10037

rn:R02468 { (-)-Limonene } <-- [Reduced NADPH---hemoprotein reductase] -- rn:R11642 <-- Acetone cyanohydrin --> rn:R01553

rn:R02469 { (-)-Limonene } <-- [Reduced NADPH---hemoprotein reductase] -- rn:R11642 <-- Acetone cyanohydrin --> rn:R01553

rn:R02470 { (-)-Limonene } <-- [Reduced NADPH---hemoprotein reductase] -- rn:R11642 <-- Acetone cyanohydrin --> rn:R01553

rn:R04366 { Linalool } <-- [Reduced NADPH---hemoprotein reductase] -- rn:R11642 <-- Acetone cyanohydrin --> rn:R01553

rn:R06119 { d-Limonene } <-- [Reduced NADPH---hemoprotein reductase] -- rn:R11642 <-- Acetone cyanohydrin --> rn:R01553

rn:R09451 { Hexadecanoic acid } <-- [Reduced NADPH---hemoprotein reductase] -- rn:R11642 <-- Acetone cyanohydrin --> rn:R01553

rn:R09452 { (9Z)-Octadecenoic acid } <-- [Reduced NADPH---hemoprotein reductase] -- rn:R11642 <-- Acetone cyanohydrin --> rn:R01553

rn:R09922 { (+)-Linalool } <-- [Reduced NADPH---hemoprotein reductase] -- rn:R11642 <-- Acetone cyanohydrin --> rn:R01553

rn:R09923 { (-)-Linalool } <-- [Reduced NADPH---hemoprotein reductase] -- rn:R11642 <-- Acetone cyanohydrin --> rn:R01553

rn:R09925 { Linalool } <-- [Reduced NADPH---hemoprotein reductase] -- rn:R11642 <-- Acetone cyanohydrin --> rn:R01553

rn:R09934 { Humulene } <-- [Reduced NADPH---hemoprotein reductase] -- rn:R11642 <-- Acetone cyanohydrin --> rn:R01553

rn:R10562 { (E,E)-Geranyllinalool } <-- [Reduced NADPH---hemoprotein reductase] -- rn:R11642 <-- Acetone cyanohydrin --> rn:R01553

rn:R11055 { Myrcene } <-- [Reduced NADPH---hemoprotein reductase] -- rn:R11642 <-- Acetone cyanohydrin --> rn:R01553

rn:R02468 { (-)-Limonene } <-- [Reduced NADPH---hemoprotein reductase] -- rn:R11642 <-- Acetone cyanohydrin --> rn:R03625

rn:R02469 { (-)-Limonene } <-- [Reduced NADPH---hemoprotein reductase] -- rn:R11642 <-- Acetone cyanohydrin --> rn:R03625

rn:R02470 { (-)-Limonene } <-- [Reduced NADPH---hemoprotein reductase] -- rn:R11642 <-- Acetone cyanohydrin --> rn:R03625

rn:R04366 { Linalool } <-- [Reduced NADPH---hemoprotein reductase] -- rn:R11642 <-- Acetone cyanohydrin --> rn:R03625

rn:R06119 { d-Limonene } <-- [Reduced NADPH---hemoprotein reductase] -- rn:R11642 <-- Acetone cyanohydrin --> rn:R03625

rn:R09451 { Hexadecanoic acid } <-- [Reduced NADPH---hemoprotein reductase] -- rn:R11642 <-- Acetone cyanohydrin --> rn:R03625

rn:R09452 { (9Z)-Octadecenoic acid } <-- [Reduced NADPH---hemoprotein reductase] -- rn:R11642 <-- Acetone cyanohydrin --> rn:R03625

rn:R09922 { (+)-Linalool } <-- [Reduced NADPH---hemoprotein reductase] -- rn:R11642 <-- Acetone cyanohydrin --> rn:R03625

rn:R09923 { (-)-Linalool } <-- [Reduced NADPH---hemoprotein reductase] -- rn:R11642 <-- Acetone cyanohydrin --> rn:R03625

rn:R09925 { Linalool } <-- [Reduced NADPH---hemoprotein reductase] -- rn:R11642 <-- Acetone cyanohydrin --> rn:R03625

rn:R09934 { Humulene } <-- [Reduced NADPH---hemoprotein reductase] -- rn:R11642 <-- Acetone cyanohydrin --> rn:R03625

rn:R10562 { (E,E)-Geranyllinalool } <-- [Reduced NADPH---hemoprotein reductase] -- rn:R11642 <-- Acetone cyanohydrin --> rn:R03625

rn:R11055 { Myrcene } <-- [Reduced NADPH---hemoprotein reductase] -- rn:R11642 <-- Acetone cyanohydrin --> rn:R03625

rn:R02468 { (-)-Limonene } <-- [Reduced NADPH---hemoprotein reductase] -- rn:R11642 <-- [Oxidized NADPH---hemoprotein reductase] --> rn:R08551

rn:R02469 { (-)-Limonene } <-- [Reduced NADPH---hemoprotein reductase] -- rn:R11642 <-- [Oxidized NADPH---hemoprotein reductase] --> rn:R08551

rn:R02470 { (-)-Limonene } <-- [Reduced NADPH---hemoprotein reductase] -- rn:R11642 <-- [Oxidized NADPH---hemoprotein reductase] --> rn:R08551

rn:R04366 { Linalool } <-- [Reduced NADPH---hemoprotein reductase] -- rn:R11642 <-- [Oxidized NADPH---hemoprotein reductase] --> rn:R08551

rn:R06119 { d-Limonene } <-- [Reduced NADPH---hemoprotein reductase] -- rn:R11642 <-- [Oxidized NADPH---hemoprotein reductase] --> rn:R08551

rn:R09451 { Hexadecanoic acid } <-- [Reduced NADPH---hemoprotein reductase] -- rn:R11642 <-- [Oxidized NADPH---hemoprotein reductase] --> rn:R08551

rn:R09452 { (9Z)-Octadecenoic acid } <-- [Reduced NADPH---hemoprotein reductase] -- rn:R11642 <-- [Oxidized NADPH---hemoprotein reductase] --> rn:R08551

rn:R09922 { (+)-Linalool } <-- [Reduced NADPH---hemoprotein reductase] -- rn:R11642 <-- [Oxidized NADPH---hemoprotein reductase] --> rn:R08551

rn:R09923 { (-)-Linalool } <-- [Reduced NADPH---hemoprotein reductase] -- rn:R11642 <-- [Oxidized NADPH---hemoprotein reductase] --> rn:R08551

rn:R09925 { Linalool } <-- [Reduced NADPH---hemoprotein reductase] -- rn:R11642 <-- [Oxidized NADPH---hemoprotein reductase] --> rn:R08551

rn:R09934 { Humulene } <-- [Reduced NADPH---hemoprotein reductase] -- rn:R11642 <-- [Oxidized NADPH---hemoprotein reductase] --> rn:R08551

rn:R10562 { (E,E)-Geranyllinalool } <-- [Reduced NADPH---hemoprotein reductase] -- rn:R11642 <-- [Oxidized NADPH---hemoprotein reductase] --> rn:R08551

rn:R11055 { Myrcene } <-- [Reduced NADPH---hemoprotein reductase] -- rn:R11642 <-- [Oxidized NADPH---hemoprotein reductase] --> rn:R08551

rn:R05488 { Styrene } <-- FADH2 -- rn:R11653 <-- FAD --> rn:R02487

rn:R05488 { Styrene } <-- FADH2 -- rn:R11653 <-- FAD --> rn:R04095

rn:R05488 { Styrene } <-- FADH2 -- rn:R11653 <-- FAD --> rn:R05537

rn:R05488 { Styrene } <-- FADH2 -- rn:R11653 <-- FAD --> rn:R06943

rn:R05488 { Styrene } <-- FADH2 -- rn:R11653 <-- FAD --> rn:R07220

rn:R05488 { Styrene } <-- FADH2 -- rn:R11653 <-- FAD --> rn:R09520

rn:R05488 { Styrene } <-- FADH2 -- rn:R11653 <-- FAD --> rn:R11130

rn:R08530 { (-)-Menthol } <-- Acetyl-CoA -- rn:R11708 <-- (R)-2-(Phosphonomethyl)malate --> rn:R08881

rn:R08531 { (+)-Neomenthol } <-- Acetyl-CoA -- rn:R11708 <-- (R)-2-(Phosphonomethyl)malate --> rn:R08881

rn:R08532 { (+)-Borneol } <-- Acetyl-CoA -- rn:R11708 <-- (R)-2-(Phosphonomethyl)malate --> rn:R08881

rn:R10474 { Cinnamyl alcohol } <-- Acetyl-CoA -- rn:R11708 <-- (R)-2-(Phosphonomethyl)malate --> rn:R08881

rn:R02468 { (-)-Limonene } <-- [Reduced NADPH---hemoprotein reductase] -- rn:R11732 <-- Mandelonitrile --> rn:R01767

rn:R02469 { (-)-Limonene } <-- [Reduced NADPH---hemoprotein reductase] -- rn:R11732 <-- Mandelonitrile --> rn:R01767

rn:R02470 { (-)-Limonene } <-- [Reduced NADPH---hemoprotein reductase] -- rn:R11732 <-- Mandelonitrile --> rn:R01767

rn:R04366 { Linalool } <-- [Reduced NADPH---hemoprotein reductase] -- rn:R11732 <-- Mandelonitrile --> rn:R01767

rn:R06119 { d-Limonene } <-- [Reduced NADPH---hemoprotein reductase] -- rn:R11732 <-- Mandelonitrile --> rn:R01767

rn:R09451 { Hexadecanoic acid } <-- [Reduced NADPH---hemoprotein reductase] -- rn:R11732 <-- Mandelonitrile --> rn:R01767

rn:R09452 { (9Z)-Octadecenoic acid } <-- [Reduced NADPH---hemoprotein reductase] -- rn:R11732 <-- Mandelonitrile --> rn:R01767

rn:R09922 { (+)-Linalool } <-- [Reduced NADPH---hemoprotein reductase] -- rn:R11732 <-- Mandelonitrile --> rn:R01767

rn:R09923 { (-)-Linalool } <-- [Reduced NADPH---hemoprotein reductase] -- rn:R11732 <-- Mandelonitrile --> rn:R01767

rn:R09925 { Linalool } <-- [Reduced NADPH---hemoprotein reductase] -- rn:R11732 <-- Mandelonitrile --> rn:R01767

rn:R09934 { Humulene } <-- [Reduced NADPH---hemoprotein reductase] -- rn:R11732 <-- Mandelonitrile --> rn:R01767

rn:R10562 { (E,E)-Geranyllinalool } <-- [Reduced NADPH---hemoprotein reductase] -- rn:R11732 <-- Mandelonitrile --> rn:R01767

rn:R11055 { Myrcene } <-- [Reduced NADPH---hemoprotein reductase] -- rn:R11732 <-- Mandelonitrile --> rn:R01767

rn:R02468 { (-)-Limonene } <-- [Reduced NADPH---hemoprotein reductase] -- rn:R11732 <-- Mandelonitrile --> rn:R03642

rn:R02469 { (-)-Limonene } <-- [Reduced NADPH---hemoprotein reductase] -- rn:R11732 <-- Mandelonitrile --> rn:R03642

rn:R02470 { (-)-Limonene } <-- [Reduced NADPH---hemoprotein reductase] -- rn:R11732 <-- Mandelonitrile --> rn:R03642

rn:R04366 { Linalool } <-- [Reduced NADPH---hemoprotein reductase] -- rn:R11732 <-- Mandelonitrile --> rn:R03642

rn:R06119 { d-Limonene } <-- [Reduced NADPH---hemoprotein reductase] -- rn:R11732 <-- Mandelonitrile --> rn:R03642

rn:R09451 { Hexadecanoic acid } <-- [Reduced NADPH---hemoprotein reductase] -- rn:R11732 <-- Mandelonitrile --> rn:R03642

rn:R09452 { (9Z)-Octadecenoic acid } <-- [Reduced NADPH---hemoprotein reductase] -- rn:R11732 <-- Mandelonitrile --> rn:R03642

rn:R09922 { (+)-Linalool } <-- [Reduced NADPH---hemoprotein reductase] -- rn:R11732 <-- Mandelonitrile --> rn:R03642

rn:R09923 { (-)-Linalool } <-- [Reduced NADPH---hemoprotein reductase] -- rn:R11732 <-- Mandelonitrile --> rn:R03642

rn:R09925 { Linalool } <-- [Reduced NADPH---hemoprotein reductase] -- rn:R11732 <-- Mandelonitrile --> rn:R03642

rn:R09934 { Humulene } <-- [Reduced NADPH---hemoprotein reductase] -- rn:R11732 <-- Mandelonitrile --> rn:R03642

rn:R10562 { (E,E)-Geranyllinalool } <-- [Reduced NADPH---hemoprotein reductase] -- rn:R11732 <-- Mandelonitrile --> rn:R03642

rn:R11055 { Myrcene } <-- [Reduced NADPH---hemoprotein reductase] -- rn:R11732 <-- Mandelonitrile --> rn:R03642

rn:R02468 { (-)-Limonene } <-- [Reduced NADPH---hemoprotein reductase] -- rn:R11732 <-- [Oxidized NADPH---hemoprotein reductase] --> rn:R08551

rn:R02469 { (-)-Limonene } <-- [Reduced NADPH---hemoprotein reductase] -- rn:R11732 <-- [Oxidized NADPH---hemoprotein reductase] --> rn:R08551

rn:R02470 { (-)-Limonene } <-- [Reduced NADPH---hemoprotein reductase] -- rn:R11732 <-- [Oxidized NADPH---hemoprotein reductase] --> rn:R08551

rn:R04366 { Linalool } <-- [Reduced NADPH---hemoprotein reductase] -- rn:R11732 <-- [Oxidized NADPH---hemoprotein reductase] --> rn:R08551

rn:R06119 { d-Limonene } <-- [Reduced NADPH---hemoprotein reductase] -- rn:R11732 <-- [Oxidized NADPH---hemoprotein reductase] --> rn:R08551

rn:R09451 { Hexadecanoic acid } <-- [Reduced NADPH---hemoprotein reductase] -- rn:R11732 <-- [Oxidized NADPH---hemoprotein reductase] --> rn:R08551

rn:R09452 { (9Z)-Octadecenoic acid } <-- [Reduced NADPH---hemoprotein reductase] -- rn:R11732 <-- [Oxidized NADPH---hemoprotein reductase] --> rn:R08551

rn:R09922 { (+)-Linalool } <-- [Reduced NADPH---hemoprotein reductase] -- rn:R11732 <-- [Oxidized NADPH---hemoprotein reductase] --> rn:R08551

rn:R09923 { (-)-Linalool } <-- [Reduced NADPH---hemoprotein reductase] -- rn:R11732 <-- [Oxidized NADPH---hemoprotein reductase] --> rn:R08551

rn:R09925 { Linalool } <-- [Reduced NADPH---hemoprotein reductase] -- rn:R11732 <-- [Oxidized NADPH---hemoprotein reductase] --> rn:R08551

rn:R09934 { Humulene } <-- [Reduced NADPH---hemoprotein reductase] -- rn:R11732 <-- [Oxidized NADPH---hemoprotein reductase] --> rn:R08551

rn:R10562 { (E,E)-Geranyllinalool } <-- [Reduced NADPH---hemoprotein reductase] -- rn:R11732 <-- [Oxidized NADPH---hemoprotein reductase] --> rn:R08551

rn:R11055 { Myrcene } <-- [Reduced NADPH---hemoprotein reductase] -- rn:R11732 <-- [Oxidized NADPH---hemoprotein reductase] --> rn:R08551

rn:R02468 { (-)-Limonene } <-- [Reduced NADPH---hemoprotein reductase] -- rn:R11732 <-- Mandelonitrile --> rn:R10638

rn:R02469 { (-)-Limonene } <-- [Reduced NADPH---hemoprotein reductase] -- rn:R11732 <-- Mandelonitrile --> rn:R10638

rn:R02470 { (-)-Limonene } <-- [Reduced NADPH---hemoprotein reductase] -- rn:R11732 <-- Mandelonitrile --> rn:R10638

rn:R04366 { Linalool } <-- [Reduced NADPH---hemoprotein reductase] -- rn:R11732 <-- Mandelonitrile --> rn:R10638

rn:R06119 { d-Limonene } <-- [Reduced NADPH---hemoprotein reductase] -- rn:R11732 <-- Mandelonitrile --> rn:R10638

rn:R09451 { Hexadecanoic acid } <-- [Reduced NADPH---hemoprotein reductase] -- rn:R11732 <-- Mandelonitrile --> rn:R10638

rn:R09452 { (9Z)-Octadecenoic acid } <-- [Reduced NADPH---hemoprotein reductase] -- rn:R11732 <-- Mandelonitrile --> rn:R10638

rn:R09922 { (+)-Linalool } <-- [Reduced NADPH---hemoprotein reductase] -- rn:R11732 <-- Mandelonitrile --> rn:R10638

rn:R09923 { (-)-Linalool } <-- [Reduced NADPH---hemoprotein reductase] -- rn:R11732 <-- Mandelonitrile --> rn:R10638

rn:R09925 { Linalool } <-- [Reduced NADPH---hemoprotein reductase] -- rn:R11732 <-- Mandelonitrile --> rn:R10638

rn:R09934 { Humulene } <-- [Reduced NADPH---hemoprotein reductase] -- rn:R11732 <-- Mandelonitrile --> rn:R10638

rn:R10562 { (E,E)-Geranyllinalool } <-- [Reduced NADPH---hemoprotein reductase] -- rn:R11732 <-- Mandelonitrile --> rn:R10638

rn:R11055 { Myrcene } <-- [Reduced NADPH---hemoprotein reductase] -- rn:R11732 <-- Mandelonitrile --> rn:R10638

rn:R02468 { (-)-Limonene } <-- [Reduced NADPH---hemoprotein reductase] -- rn:R11732 <-- Mandelonitrile --> rn:R11380

rn:R02469 { (-)-Limonene } <-- [Reduced NADPH---hemoprotein reductase] -- rn:R11732 <-- Mandelonitrile --> rn:R11380

rn:R02470 { (-)-Limonene } <-- [Reduced NADPH---hemoprotein reductase] -- rn:R11732 <-- Mandelonitrile --> rn:R11380

rn:R04366 { Linalool } <-- [Reduced NADPH---hemoprotein reductase] -- rn:R11732 <-- Mandelonitrile --> rn:R11380

rn:R06119 { d-Limonene } <-- [Reduced NADPH---hemoprotein reductase] -- rn:R11732 <-- Mandelonitrile --> rn:R11380

rn:R09451 { Hexadecanoic acid } <-- [Reduced NADPH---hemoprotein reductase] -- rn:R11732 <-- Mandelonitrile --> rn:R11380

rn:R09452 { (9Z)-Octadecenoic acid } <-- [Reduced NADPH---hemoprotein reductase] -- rn:R11732 <-- Mandelonitrile --> rn:R11380

rn:R09922 { (+)-Linalool } <-- [Reduced NADPH---hemoprotein reductase] -- rn:R11732 <-- Mandelonitrile --> rn:R11380

rn:R09923 { (-)-Linalool } <-- [Reduced NADPH---hemoprotein reductase] -- rn:R11732 <-- Mandelonitrile --> rn:R11380

rn:R09925 { Linalool } <-- [Reduced NADPH---hemoprotein reductase] -- rn:R11732 <-- Mandelonitrile --> rn:R11380

rn:R09934 { Humulene } <-- [Reduced NADPH---hemoprotein reductase] -- rn:R11732 <-- Mandelonitrile --> rn:R11380

rn:R10562 { (E,E)-Geranyllinalool } <-- [Reduced NADPH---hemoprotein reductase] -- rn:R11732 <-- Mandelonitrile --> rn:R11380

rn:R11055 { Myrcene } <-- [Reduced NADPH---hemoprotein reductase] -- rn:R11732 <-- Mandelonitrile --> rn:R11380

rn:R02468 { (-)-Limonene } <-- [Reduced NADPH---hemoprotein reductase] -- rn:R11733 <-- Mandelonitrile --> rn:R01767

rn:R02469 { (-)-Limonene } <-- [Reduced NADPH---hemoprotein reductase] -- rn:R11733 <-- Mandelonitrile --> rn:R01767

rn:R02470 { (-)-Limonene } <-- [Reduced NADPH---hemoprotein reductase] -- rn:R11733 <-- Mandelonitrile --> rn:R01767

rn:R04366 { Linalool } <-- [Reduced NADPH---hemoprotein reductase] -- rn:R11733 <-- Mandelonitrile --> rn:R01767

rn:R06119 { d-Limonene } <-- [Reduced NADPH---hemoprotein reductase] -- rn:R11733 <-- Mandelonitrile --> rn:R01767

rn:R09451 { Hexadecanoic acid } <-- [Reduced NADPH---hemoprotein reductase] -- rn:R11733 <-- Mandelonitrile --> rn:R01767

rn:R09452 { (9Z)-Octadecenoic acid } <-- [Reduced NADPH---hemoprotein reductase] -- rn:R11733 <-- Mandelonitrile --> rn:R01767

rn:R09922 { (+)-Linalool } <-- [Reduced NADPH---hemoprotein reductase] -- rn:R11733 <-- Mandelonitrile --> rn:R01767

rn:R09923 { (-)-Linalool } <-- [Reduced NADPH---hemoprotein reductase] -- rn:R11733 <-- Mandelonitrile --> rn:R01767

rn:R09925 { Linalool } <-- [Reduced NADPH---hemoprotein reductase] -- rn:R11733 <-- Mandelonitrile --> rn:R01767

rn:R09934 { Humulene } <-- [Reduced NADPH---hemoprotein reductase] -- rn:R11733 <-- Mandelonitrile --> rn:R01767

rn:R10562 { (E,E)-Geranyllinalool } <-- [Reduced NADPH---hemoprotein reductase] -- rn:R11733 <-- Mandelonitrile --> rn:R01767

rn:R11055 { Myrcene } <-- [Reduced NADPH---hemoprotein reductase] -- rn:R11733 <-- Mandelonitrile --> rn:R01767

rn:R02468 { (-)-Limonene } <-- [Reduced NADPH---hemoprotein reductase] -- rn:R11733 <-- Mandelonitrile --> rn:R03642

rn:R02469 { (-)-Limonene } <-- [Reduced NADPH---hemoprotein reductase] -- rn:R11733 <-- Mandelonitrile --> rn:R03642

rn:R02470 { (-)-Limonene } <-- [Reduced NADPH---hemoprotein reductase] -- rn:R11733 <-- Mandelonitrile --> rn:R03642

rn:R04366 { Linalool } <-- [Reduced NADPH---hemoprotein reductase] -- rn:R11733 <-- Mandelonitrile --> rn:R03642

rn:R06119 { d-Limonene } <-- [Reduced NADPH---hemoprotein reductase] -- rn:R11733 <-- Mandelonitrile --> rn:R03642

rn:R09451 { Hexadecanoic acid } <-- [Reduced NADPH---hemoprotein reductase] -- rn:R11733 <-- Mandelonitrile --> rn:R03642

rn:R09452 { (9Z)-Octadecenoic acid } <-- [Reduced NADPH---hemoprotein reductase] -- rn:R11733 <-- Mandelonitrile --> rn:R03642

rn:R09922 { (+)-Linalool } <-- [Reduced NADPH---hemoprotein reductase] -- rn:R11733 <-- Mandelonitrile --> rn:R03642

rn:R09923 { (-)-Linalool } <-- [Reduced NADPH---hemoprotein reductase] -- rn:R11733 <-- Mandelonitrile --> rn:R03642

rn:R09925 { Linalool } <-- [Reduced NADPH---hemoprotein reductase] -- rn:R11733 <-- Mandelonitrile --> rn:R03642

rn:R09934 { Humulene } <-- [Reduced NADPH---hemoprotein reductase] -- rn:R11733 <-- Mandelonitrile --> rn:R03642

rn:R10562 { (E,E)-Geranyllinalool } <-- [Reduced NADPH---hemoprotein reductase] -- rn:R11733 <-- Mandelonitrile --> rn:R03642

rn:R11055 { Myrcene } <-- [Reduced NADPH---hemoprotein reductase] -- rn:R11733 <-- Mandelonitrile --> rn:R03642

rn:R02468 { (-)-Limonene } <-- [Reduced NADPH---hemoprotein reductase] -- rn:R11733 <-- [Oxidized NADPH---hemoprotein reductase] --> rn:R08551

rn:R02469 { (-)-Limonene } <-- [Reduced NADPH---hemoprotein reductase] -- rn:R11733 <-- [Oxidized NADPH---hemoprotein reductase] --> rn:R08551

rn:R02470 { (-)-Limonene } <-- [Reduced NADPH---hemoprotein reductase] -- rn:R11733 <-- [Oxidized NADPH---hemoprotein reductase] --> rn:R08551

rn:R04366 { Linalool } <-- [Reduced NADPH---hemoprotein reductase] -- rn:R11733 <-- [Oxidized NADPH---hemoprotein reductase] --> rn:R08551

rn:R06119 { d-Limonene } <-- [Reduced NADPH---hemoprotein reductase] -- rn:R11733 <-- [Oxidized NADPH---hemoprotein reductase] --> rn:R08551

rn:R09451 { Hexadecanoic acid } <-- [Reduced NADPH---hemoprotein reductase] -- rn:R11733 <-- [Oxidized NADPH---hemoprotein reductase] --> rn:R08551

rn:R09452 { (9Z)-Octadecenoic acid } <-- [Reduced NADPH---hemoprotein reductase] -- rn:R11733 <-- [Oxidized NADPH---hemoprotein reductase] --> rn:R08551

rn:R09922 { (+)-Linalool } <-- [Reduced NADPH---hemoprotein reductase] -- rn:R11733 <-- [Oxidized NADPH---hemoprotein reductase] --> rn:R08551

rn:R09923 { (-)-Linalool } <-- [Reduced NADPH---hemoprotein reductase] -- rn:R11733 <-- [Oxidized NADPH---hemoprotein reductase] --> rn:R08551

rn:R09925 { Linalool } <-- [Reduced NADPH---hemoprotein reductase] -- rn:R11733 <-- [Oxidized NADPH---hemoprotein reductase] --> rn:R08551

rn:R09934 { Humulene } <-- [Reduced NADPH---hemoprotein reductase] -- rn:R11733 <-- [Oxidized NADPH---hemoprotein reductase] --> rn:R08551

rn:R10562 { (E,E)-Geranyllinalool } <-- [Reduced NADPH---hemoprotein reductase] -- rn:R11733 <-- [Oxidized NADPH---hemoprotein reductase] --> rn:R08551

rn:R11055 { Myrcene } <-- [Reduced NADPH---hemoprotein reductase] -- rn:R11733 <-- [Oxidized NADPH---hemoprotein reductase] --> rn:R08551

rn:R02468 { (-)-Limonene } <-- [Reduced NADPH---hemoprotein reductase] -- rn:R11733 <-- Mandelonitrile --> rn:R10638

rn:R02469 { (-)-Limonene } <-- [Reduced NADPH---hemoprotein reductase] -- rn:R11733 <-- Mandelonitrile --> rn:R10638

rn:R02470 { (-)-Limonene } <-- [Reduced NADPH---hemoprotein reductase] -- rn:R11733 <-- Mandelonitrile --> rn:R10638

rn:R04366 { Linalool } <-- [Reduced NADPH---hemoprotein reductase] -- rn:R11733 <-- Mandelonitrile --> rn:R10638

rn:R06119 { d-Limonene } <-- [Reduced NADPH---hemoprotein reductase] -- rn:R11733 <-- Mandelonitrile --> rn:R10638

rn:R09451 { Hexadecanoic acid } <-- [Reduced NADPH---hemoprotein reductase] -- rn:R11733 <-- Mandelonitrile --> rn:R10638

rn:R09452 { (9Z)-Octadecenoic acid } <-- [Reduced NADPH---hemoprotein reductase] -- rn:R11733 <-- Mandelonitrile --> rn:R10638

rn:R09922 { (+)-Linalool } <-- [Reduced NADPH---hemoprotein reductase] -- rn:R11733 <-- Mandelonitrile --> rn:R10638

rn:R09923 { (-)-Linalool } <-- [Reduced NADPH---hemoprotein reductase] -- rn:R11733 <-- Mandelonitrile --> rn:R10638

rn:R09925 { Linalool } <-- [Reduced NADPH---hemoprotein reductase] -- rn:R11733 <-- Mandelonitrile --> rn:R10638

rn:R09934 { Humulene } <-- [Reduced NADPH---hemoprotein reductase] -- rn:R11733 <-- Mandelonitrile --> rn:R10638

rn:R10562 { (E,E)-Geranyllinalool } <-- [Reduced NADPH---hemoprotein reductase] -- rn:R11733 <-- Mandelonitrile --> rn:R10638

rn:R11055 { Myrcene } <-- [Reduced NADPH---hemoprotein reductase] -- rn:R11733 <-- Mandelonitrile --> rn:R10638

rn:R02468 { (-)-Limonene } <-- [Reduced NADPH---hemoprotein reductase] -- rn:R11733 <-- Mandelonitrile --> rn:R11380

rn:R02469 { (-)-Limonene } <-- [Reduced NADPH---hemoprotein reductase] -- rn:R11733 <-- Mandelonitrile --> rn:R11380

rn:R02470 { (-)-Limonene } <-- [Reduced NADPH---hemoprotein reductase] -- rn:R11733 <-- Mandelonitrile --> rn:R11380

rn:R04366 { Linalool } <-- [Reduced NADPH---hemoprotein reductase] -- rn:R11733 <-- Mandelonitrile --> rn:R11380

rn:R06119 { d-Limonene } <-- [Reduced NADPH---hemoprotein reductase] -- rn:R11733 <-- Mandelonitrile --> rn:R11380

rn:R09451 { Hexadecanoic acid } <-- [Reduced NADPH---hemoprotein reductase] -- rn:R11733 <-- Mandelonitrile --> rn:R11380

rn:R09452 { (9Z)-Octadecenoic acid } <-- [Reduced NADPH---hemoprotein reductase] -- rn:R11733 <-- Mandelonitrile --> rn:R11380

rn:R09922 { (+)-Linalool } <-- [Reduced NADPH---hemoprotein reductase] -- rn:R11733 <-- Mandelonitrile --> rn:R11380

rn:R09923 { (-)-Linalool } <-- [Reduced NADPH---hemoprotein reductase] -- rn:R11733 <-- Mandelonitrile --> rn:R11380

rn:R09925 { Linalool } <-- [Reduced NADPH---hemoprotein reductase] -- rn:R11733 <-- Mandelonitrile --> rn:R11380

rn:R09934 { Humulene } <-- [Reduced NADPH---hemoprotein reductase] -- rn:R11733 <-- Mandelonitrile --> rn:R11380

rn:R10562 { (E,E)-Geranyllinalool } <-- [Reduced NADPH---hemoprotein reductase] -- rn:R11733 <-- Mandelonitrile --> rn:R11380

rn:R11055 { Myrcene } <-- [Reduced NADPH---hemoprotein reductase] -- rn:R11733 <-- Mandelonitrile --> rn:R11380

rn:R02468 { (-)-Limonene } <-- [Reduced NADPH---hemoprotein reductase] -- rn:R11737 <-- [Oxidized NADPH---hemoprotein reductase] --> rn:R08551

rn:R02469 { (-)-Limonene } <-- [Reduced NADPH---hemoprotein reductase] -- rn:R11737 <-- [Oxidized NADPH---hemoprotein reductase] --> rn:R08551

rn:R02470 { (-)-Limonene } <-- [Reduced NADPH---hemoprotein reductase] -- rn:R11737 <-- [Oxidized NADPH---hemoprotein reductase] --> rn:R08551

rn:R04366 { Linalool } <-- [Reduced NADPH---hemoprotein reductase] -- rn:R11737 <-- [Oxidized NADPH---hemoprotein reductase] --> rn:R08551

rn:R06119 { d-Limonene } <-- [Reduced NADPH---hemoprotein reductase] -- rn:R11737 <-- [Oxidized NADPH---hemoprotein reductase] --> rn:R08551

rn:R09451 { Hexadecanoic acid } <-- [Reduced NADPH---hemoprotein reductase] -- rn:R11737 <-- [Oxidized NADPH---hemoprotein reductase] --> rn:R08551

rn:R09452 { (9Z)-Octadecenoic acid } <-- [Reduced NADPH---hemoprotein reductase] -- rn:R11737 <-- [Oxidized NADPH---hemoprotein reductase] --> rn:R08551

rn:R09922 { (+)-Linalool } <-- [Reduced NADPH---hemoprotein reductase] -- rn:R11737 <-- [Oxidized NADPH---hemoprotein reductase] --> rn:R08551

rn:R09923 { (-)-Linalool } <-- [Reduced NADPH---hemoprotein reductase] -- rn:R11737 <-- [Oxidized NADPH---hemoprotein reductase] --> rn:R08551

rn:R09925 { Linalool } <-- [Reduced NADPH---hemoprotein reductase] -- rn:R11737 <-- [Oxidized NADPH---hemoprotein reductase] --> rn:R08551

rn:R09934 { Humulene } <-- [Reduced NADPH---hemoprotein reductase] -- rn:R11737 <-- [Oxidized NADPH---hemoprotein reductase] --> rn:R08551

rn:R10562 { (E,E)-Geranyllinalool } <-- [Reduced NADPH---hemoprotein reductase] -- rn:R11737 <-- [Oxidized NADPH---hemoprotein reductase] --> rn:R08551

rn:R11055 { Myrcene } <-- [Reduced NADPH---hemoprotein reductase] -- rn:R11737 <-- [Oxidized NADPH---hemoprotein reductase] --> rn:R08551

rn:R02468 { (-)-Limonene } <-- [Reduced NADPH---hemoprotein reductase] -- rn:R11738 <-- [Oxidized NADPH---hemoprotein reductase] --> rn:R08551

rn:R02469 { (-)-Limonene } <-- [Reduced NADPH---hemoprotein reductase] -- rn:R11738 <-- [Oxidized NADPH---hemoprotein reductase] --> rn:R08551

rn:R02470 { (-)-Limonene } <-- [Reduced NADPH---hemoprotein reductase] -- rn:R11738 <-- [Oxidized NADPH---hemoprotein reductase] --> rn:R08551

rn:R04366 { Linalool } <-- [Reduced NADPH---hemoprotein reductase] -- rn:R11738 <-- [Oxidized NADPH---hemoprotein reductase] --> rn:R08551

rn:R06119 { d-Limonene } <-- [Reduced NADPH---hemoprotein reductase] -- rn:R11738 <-- [Oxidized NADPH---hemoprotein reductase] --> rn:R08551

rn:R09451 { Hexadecanoic acid } <-- [Reduced NADPH---hemoprotein reductase] -- rn:R11738 <-- [Oxidized NADPH---hemoprotein reductase] --> rn:R08551

rn:R09452 { (9Z)-Octadecenoic acid } <-- [Reduced NADPH---hemoprotein reductase] -- rn:R11738 <-- [Oxidized NADPH---hemoprotein reductase] --> rn:R08551

rn:R09922 { (+)-Linalool } <-- [Reduced NADPH---hemoprotein reductase] -- rn:R11738 <-- [Oxidized NADPH---hemoprotein reductase] --> rn:R08551

rn:R09923 { (-)-Linalool } <-- [Reduced NADPH---hemoprotein reductase] -- rn:R11738 <-- [Oxidized NADPH---hemoprotein reductase] --> rn:R08551

rn:R09925 { Linalool } <-- [Reduced NADPH---hemoprotein reductase] -- rn:R11738 <-- [Oxidized NADPH---hemoprotein reductase] --> rn:R08551

rn:R09934 { Humulene } <-- [Reduced NADPH---hemoprotein reductase] -- rn:R11738 <-- [Oxidized NADPH---hemoprotein reductase] --> rn:R08551

rn:R10562 { (E,E)-Geranyllinalool } <-- [Reduced NADPH---hemoprotein reductase] -- rn:R11738 <-- [Oxidized NADPH---hemoprotein reductase] --> rn:R08551

rn:R11055 { Myrcene } <-- [Reduced NADPH---hemoprotein reductase] -- rn:R11738 <-- [Oxidized NADPH---hemoprotein reductase] --> rn:R08551

rn:R02468 { (-)-Limonene } <-- [Reduced NADPH---hemoprotein reductase] -- rn:R11813 <-- Hydrogen cyanide --> rn:R00152

rn:R02469 { (-)-Limonene } <-- [Reduced NADPH---hemoprotein reductase] -- rn:R11813 <-- Hydrogen cyanide --> rn:R00152

rn:R02470 { (-)-Limonene } <-- [Reduced NADPH---hemoprotein reductase] -- rn:R11813 <-- Hydrogen cyanide --> rn:R00152

rn:R04366 { Linalool } <-- [Reduced NADPH---hemoprotein reductase] -- rn:R11813 <-- Hydrogen cyanide --> rn:R00152

rn:R06119 { d-Limonene } <-- [Reduced NADPH---hemoprotein reductase] -- rn:R11813 <-- Hydrogen cyanide --> rn:R00152

rn:R09451 { Hexadecanoic acid } <-- [Reduced NADPH---hemoprotein reductase] -- rn:R11813 <-- Hydrogen cyanide --> rn:R00152

rn:R09452 { (9Z)-Octadecenoic acid } <-- [Reduced NADPH---hemoprotein reductase] -- rn:R11813 <-- Hydrogen cyanide --> rn:R00152

rn:R09922 { (+)-Linalool } <-- [Reduced NADPH---hemoprotein reductase] -- rn:R11813 <-- Hydrogen cyanide --> rn:R00152

rn:R09923 { (-)-Linalool } <-- [Reduced NADPH---hemoprotein reductase] -- rn:R11813 <-- Hydrogen cyanide --> rn:R00152

rn:R09925 { Linalool } <-- [Reduced NADPH---hemoprotein reductase] -- rn:R11813 <-- Hydrogen cyanide --> rn:R00152

rn:R09934 { Humulene } <-- [Reduced NADPH---hemoprotein reductase] -- rn:R11813 <-- Hydrogen cyanide --> rn:R00152

rn:R10562 { (E,E)-Geranyllinalool } <-- [Reduced NADPH---hemoprotein reductase] -- rn:R11813 <-- Hydrogen cyanide --> rn:R00152

rn:R11055 { Myrcene } <-- [Reduced NADPH---hemoprotein reductase] -- rn:R11813 <-- Hydrogen cyanide --> rn:R00152

rn:R02468 { (-)-Limonene } <-- [Reduced NADPH---hemoprotein reductase] -- rn:R11813 <-- Hydrogen cyanide --> rn:R01410

rn:R02469 { (-)-Limonene } <-- [Reduced NADPH---hemoprotein reductase] -- rn:R11813 <-- Hydrogen cyanide --> rn:R01410

rn:R02470 { (-)-Limonene } <-- [Reduced NADPH---hemoprotein reductase] -- rn:R11813 <-- Hydrogen cyanide --> rn:R01410

rn:R04366 { Linalool } <-- [Reduced NADPH---hemoprotein reductase] -- rn:R11813 <-- Hydrogen cyanide --> rn:R01410

rn:R06119 { d-Limonene } <-- [Reduced NADPH---hemoprotein reductase] -- rn:R11813 <-- Hydrogen cyanide --> rn:R01410

rn:R09451 { Hexadecanoic acid } <-- [Reduced NADPH---hemoprotein reductase] -- rn:R11813 <-- Hydrogen cyanide --> rn:R01410

rn:R09452 { (9Z)-Octadecenoic acid } <-- [Reduced NADPH---hemoprotein reductase] -- rn:R11813 <-- Hydrogen cyanide --> rn:R01410

rn:R09922 { (+)-Linalool } <-- [Reduced NADPH---hemoprotein reductase] -- rn:R11813 <-- Hydrogen cyanide --> rn:R01410

rn:R09923 { (-)-Linalool } <-- [Reduced NADPH---hemoprotein reductase] -- rn:R11813 <-- Hydrogen cyanide --> rn:R01410

rn:R09925 { Linalool } <-- [Reduced NADPH---hemoprotein reductase] -- rn:R11813 <-- Hydrogen cyanide --> rn:R01410

rn:R09934 { Humulene } <-- [Reduced NADPH---hemoprotein reductase] -- rn:R11813 <-- Hydrogen cyanide --> rn:R01410

rn:R10562 { (E,E)-Geranyllinalool } <-- [Reduced NADPH---hemoprotein reductase] -- rn:R11813 <-- Hydrogen cyanide --> rn:R01410

rn:R11055 { Myrcene } <-- [Reduced NADPH---hemoprotein reductase] -- rn:R11813 <-- Hydrogen cyanide --> rn:R01410

rn:R02468 { (-)-Limonene } <-- [Reduced NADPH---hemoprotein reductase] -- rn:R11813 <-- Hydrogen cyanide --> rn:R01650

rn:R02469 { (-)-Limonene } <-- [Reduced NADPH---hemoprotein reductase] -- rn:R11813 <-- Hydrogen cyanide --> rn:R01650

rn:R02470 { (-)-Limonene } <-- [Reduced NADPH---hemoprotein reductase] -- rn:R11813 <-- Hydrogen cyanide --> rn:R01650

rn:R04366 { Linalool } <-- [Reduced NADPH---hemoprotein reductase] -- rn:R11813 <-- Hydrogen cyanide --> rn:R01650

rn:R06119 { d-Limonene } <-- [Reduced NADPH---hemoprotein reductase] -- rn:R11813 <-- Hydrogen cyanide --> rn:R01650

rn:R09451 { Hexadecanoic acid } <-- [Reduced NADPH---hemoprotein reductase] -- rn:R11813 <-- Hydrogen cyanide --> rn:R01650

rn:R09452 { (9Z)-Octadecenoic acid } <-- [Reduced NADPH---hemoprotein reductase] -- rn:R11813 <-- Hydrogen cyanide --> rn:R01650

rn:R09922 { (+)-Linalool } <-- [Reduced NADPH---hemoprotein reductase] -- rn:R11813 <-- Hydrogen cyanide --> rn:R01650

rn:R09923 { (-)-Linalool } <-- [Reduced NADPH---hemoprotein reductase] -- rn:R11813 <-- Hydrogen cyanide --> rn:R01650

rn:R09925 { Linalool } <-- [Reduced NADPH---hemoprotein reductase] -- rn:R11813 <-- Hydrogen cyanide --> rn:R01650

rn:R09934 { Humulene } <-- [Reduced NADPH---hemoprotein reductase] -- rn:R11813 <-- Hydrogen cyanide --> rn:R01650

rn:R10562 { (E,E)-Geranyllinalool } <-- [Reduced NADPH---hemoprotein reductase] -- rn:R11813 <-- Hydrogen cyanide --> rn:R01650

rn:R11055 { Myrcene } <-- [Reduced NADPH---hemoprotein reductase] -- rn:R11813 <-- Hydrogen cyanide --> rn:R01650

rn:R02468 { (-)-Limonene } <-- [Reduced NADPH---hemoprotein reductase] -- rn:R11813 <-- Hydrogen cyanide --> rn:R03524

rn:R02469 { (-)-Limonene } <-- [Reduced NADPH---hemoprotein reductase] -- rn:R11813 <-- Hydrogen cyanide --> rn:R03524

rn:R02470 { (-)-Limonene } <-- [Reduced NADPH---hemoprotein reductase] -- rn:R11813 <-- Hydrogen cyanide --> rn:R03524

rn:R04366 { Linalool } <-- [Reduced NADPH---hemoprotein reductase] -- rn:R11813 <-- Hydrogen cyanide --> rn:R03524

rn:R06119 { d-Limonene } <-- [Reduced NADPH---hemoprotein reductase] -- rn:R11813 <-- Hydrogen cyanide --> rn:R03524

rn:R09451 { Hexadecanoic acid } <-- [Reduced NADPH---hemoprotein reductase] -- rn:R11813 <-- Hydrogen cyanide --> rn:R03524

rn:R09452 { (9Z)-Octadecenoic acid } <-- [Reduced NADPH---hemoprotein reductase] -- rn:R11813 <-- Hydrogen cyanide --> rn:R03524

rn:R09922 { (+)-Linalool } <-- [Reduced NADPH---hemoprotein reductase] -- rn:R11813 <-- Hydrogen cyanide --> rn:R03524

rn:R09923 { (-)-Linalool } <-- [Reduced NADPH---hemoprotein reductase] -- rn:R11813 <-- Hydrogen cyanide --> rn:R03524

rn:R09925 { Linalool } <-- [Reduced NADPH---hemoprotein reductase] -- rn:R11813 <-- Hydrogen cyanide --> rn:R03524

rn:R09934 { Humulene } <-- [Reduced NADPH---hemoprotein reductase] -- rn:R11813 <-- Hydrogen cyanide --> rn:R03524

rn:R10562 { (E,E)-Geranyllinalool } <-- [Reduced NADPH---hemoprotein reductase] -- rn:R11813 <-- Hydrogen cyanide --> rn:R03524

rn:R11055 { Myrcene } <-- [Reduced NADPH---hemoprotein reductase] -- rn:R11813 <-- Hydrogen cyanide --> rn:R03524

rn:R02468 { (-)-Limonene } <-- [Reduced NADPH---hemoprotein reductase] -- rn:R11813 <-- [Oxidized NADPH---hemoprotein reductase] --> rn:R08551

rn:R02469 { (-)-Limonene } <-- [Reduced NADPH---hemoprotein reductase] -- rn:R11813 <-- [Oxidized NADPH---hemoprotein reductase] --> rn:R08551

rn:R02470 { (-)-Limonene } <-- [Reduced NADPH---hemoprotein reductase] -- rn:R11813 <-- [Oxidized NADPH---hemoprotein reductase] --> rn:R08551

rn:R04366 { Linalool } <-- [Reduced NADPH---hemoprotein reductase] -- rn:R11813 <-- [Oxidized NADPH---hemoprotein reductase] --> rn:R08551

rn:R06119 { d-Limonene } <-- [Reduced NADPH---hemoprotein reductase] -- rn:R11813 <-- [Oxidized NADPH---hemoprotein reductase] --> rn:R08551

rn:R09451 { Hexadecanoic acid } <-- [Reduced NADPH---hemoprotein reductase] -- rn:R11813 <-- [Oxidized NADPH---hemoprotein reductase] --> rn:R08551

rn:R09452 { (9Z)-Octadecenoic acid } <-- [Reduced NADPH---hemoprotein reductase] -- rn:R11813 <-- [Oxidized NADPH---hemoprotein reductase] --> rn:R08551

rn:R09922 { (+)-Linalool } <-- [Reduced NADPH---hemoprotein reductase] -- rn:R11813 <-- [Oxidized NADPH---hemoprotein reductase] --> rn:R08551

rn:R09923 { (-)-Linalool } <-- [Reduced NADPH---hemoprotein reductase] -- rn:R11813 <-- [Oxidized NADPH---hemoprotein reductase] --> rn:R08551

rn:R09925 { Linalool } <-- [Reduced NADPH---hemoprotein reductase] -- rn:R11813 <-- [Oxidized NADPH---hemoprotein reductase] --> rn:R08551

rn:R09934 { Humulene } <-- [Reduced NADPH---hemoprotein reductase] -- rn:R11813 <-- [Oxidized NADPH---hemoprotein reductase] --> rn:R08551

rn:R10562 { (E,E)-Geranyllinalool } <-- [Reduced NADPH---hemoprotein reductase] -- rn:R11813 <-- [Oxidized NADPH---hemoprotein reductase] --> rn:R08551

rn:R11055 { Myrcene } <-- [Reduced NADPH---hemoprotein reductase] -- rn:R11813 <-- [Oxidized NADPH---hemoprotein reductase] --> rn:R08551

rn:R02468 { (-)-Limonene } <-- [Reduced NADPH---hemoprotein reductase] -- rn:R11814 <-- Hydrogen cyanide --> rn:R00152

rn:R02469 { (-)-Limonene } <-- [Reduced NADPH---hemoprotein reductase] -- rn:R11814 <-- Hydrogen cyanide --> rn:R00152

rn:R02470 { (-)-Limonene } <-- [Reduced NADPH---hemoprotein reductase] -- rn:R11814 <-- Hydrogen cyanide --> rn:R00152

rn:R04366 { Linalool } <-- [Reduced NADPH---hemoprotein reductase] -- rn:R11814 <-- Hydrogen cyanide --> rn:R00152

rn:R06119 { d-Limonene } <-- [Reduced NADPH---hemoprotein reductase] -- rn:R11814 <-- Hydrogen cyanide --> rn:R00152

rn:R09451 { Hexadecanoic acid } <-- [Reduced NADPH---hemoprotein reductase] -- rn:R11814 <-- Hydrogen cyanide --> rn:R00152

rn:R09452 { (9Z)-Octadecenoic acid } <-- [Reduced NADPH---hemoprotein reductase] -- rn:R11814 <-- Hydrogen cyanide --> rn:R00152

rn:R09922 { (+)-Linalool } <-- [Reduced NADPH---hemoprotein reductase] -- rn:R11814 <-- Hydrogen cyanide --> rn:R00152

rn:R09923 { (-)-Linalool } <-- [Reduced NADPH---hemoprotein reductase] -- rn:R11814 <-- Hydrogen cyanide --> rn:R00152

rn:R09925 { Linalool } <-- [Reduced NADPH---hemoprotein reductase] -- rn:R11814 <-- Hydrogen cyanide --> rn:R00152

rn:R09934 { Humulene } <-- [Reduced NADPH---hemoprotein reductase] -- rn:R11814 <-- Hydrogen cyanide --> rn:R00152

rn:R10562 { (E,E)-Geranyllinalool } <-- [Reduced NADPH---hemoprotein reductase] -- rn:R11814 <-- Hydrogen cyanide --> rn:R00152

rn:R11055 { Myrcene } <-- [Reduced NADPH---hemoprotein reductase] -- rn:R11814 <-- Hydrogen cyanide --> rn:R00152

rn:R02468 { (-)-Limonene } <-- [Reduced NADPH---hemoprotein reductase] -- rn:R11814 <-- Hydrogen cyanide --> rn:R01410

rn:R02469 { (-)-Limonene } <-- [Reduced NADPH---hemoprotein reductase] -- rn:R11814 <-- Hydrogen cyanide --> rn:R01410

rn:R02470 { (-)-Limonene } <-- [Reduced NADPH---hemoprotein reductase] -- rn:R11814 <-- Hydrogen cyanide --> rn:R01410

rn:R04366 { Linalool } <-- [Reduced NADPH---hemoprotein reductase] -- rn:R11814 <-- Hydrogen cyanide --> rn:R01410

rn:R06119 { d-Limonene } <-- [Reduced NADPH---hemoprotein reductase] -- rn:R11814 <-- Hydrogen cyanide --> rn:R01410

rn:R09451 { Hexadecanoic acid } <-- [Reduced NADPH---hemoprotein reductase] -- rn:R11814 <-- Hydrogen cyanide --> rn:R01410

rn:R09452 { (9Z)-Octadecenoic acid } <-- [Reduced NADPH---hemoprotein reductase] -- rn:R11814 <-- Hydrogen cyanide --> rn:R01410

rn:R09922 { (+)-Linalool } <-- [Reduced NADPH---hemoprotein reductase] -- rn:R11814 <-- Hydrogen cyanide --> rn:R01410

rn:R09923 { (-)-Linalool } <-- [Reduced NADPH---hemoprotein reductase] -- rn:R11814 <-- Hydrogen cyanide --> rn:R01410

rn:R09925 { Linalool } <-- [Reduced NADPH---hemoprotein reductase] -- rn:R11814 <-- Hydrogen cyanide --> rn:R01410

rn:R09934 { Humulene } <-- [Reduced NADPH---hemoprotein reductase] -- rn:R11814 <-- Hydrogen cyanide --> rn:R01410

rn:R10562 { (E,E)-Geranyllinalool } <-- [Reduced NADPH---hemoprotein reductase] -- rn:R11814 <-- Hydrogen cyanide --> rn:R01410

rn:R11055 { Myrcene } <-- [Reduced NADPH---hemoprotein reductase] -- rn:R11814 <-- Hydrogen cyanide --> rn:R01410

rn:R02468 { (-)-Limonene } <-- [Reduced NADPH---hemoprotein reductase] -- rn:R11814 <-- Hydrogen cyanide --> rn:R01650

rn:R02469 { (-)-Limonene } <-- [Reduced NADPH---hemoprotein reductase] -- rn:R11814 <-- Hydrogen cyanide --> rn:R01650

rn:R02470 { (-)-Limonene } <-- [Reduced NADPH---hemoprotein reductase] -- rn:R11814 <-- Hydrogen cyanide --> rn:R01650

rn:R04366 { Linalool } <-- [Reduced NADPH---hemoprotein reductase] -- rn:R11814 <-- Hydrogen cyanide --> rn:R01650

rn:R06119 { d-Limonene } <-- [Reduced NADPH---hemoprotein reductase] -- rn:R11814 <-- Hydrogen cyanide --> rn:R01650

rn:R09451 { Hexadecanoic acid } <-- [Reduced NADPH---hemoprotein reductase] -- rn:R11814 <-- Hydrogen cyanide --> rn:R01650

rn:R09452 { (9Z)-Octadecenoic acid } <-- [Reduced NADPH---hemoprotein reductase] -- rn:R11814 <-- Hydrogen cyanide --> rn:R01650

rn:R09922 { (+)-Linalool } <-- [Reduced NADPH---hemoprotein reductase] -- rn:R11814 <-- Hydrogen cyanide --> rn:R01650

rn:R09923 { (-)-Linalool } <-- [Reduced NADPH---hemoprotein reductase] -- rn:R11814 <-- Hydrogen cyanide --> rn:R01650

rn:R09925 { Linalool } <-- [Reduced NADPH---hemoprotein reductase] -- rn:R11814 <-- Hydrogen cyanide --> rn:R01650

rn:R09934 { Humulene } <-- [Reduced NADPH---hemoprotein reductase] -- rn:R11814 <-- Hydrogen cyanide --> rn:R01650

rn:R10562 { (E,E)-Geranyllinalool } <-- [Reduced NADPH---hemoprotein reductase] -- rn:R11814 <-- Hydrogen cyanide --> rn:R01650

rn:R11055 { Myrcene } <-- [Reduced NADPH---hemoprotein reductase] -- rn:R11814 <-- Hydrogen cyanide --> rn:R01650

rn:R02468 { (-)-Limonene } <-- [Reduced NADPH---hemoprotein reductase] -- rn:R11814 <-- Hydrogen cyanide --> rn:R03524

rn:R02469 { (-)-Limonene } <-- [Reduced NADPH---hemoprotein reductase] -- rn:R11814 <-- Hydrogen cyanide --> rn:R03524

rn:R02470 { (-)-Limonene } <-- [Reduced NADPH---hemoprotein reductase] -- rn:R11814 <-- Hydrogen cyanide --> rn:R03524

rn:R04366 { Linalool } <-- [Reduced NADPH---hemoprotein reductase] -- rn:R11814 <-- Hydrogen cyanide --> rn:R03524

rn:R06119 { d-Limonene } <-- [Reduced NADPH---hemoprotein reductase] -- rn:R11814 <-- Hydrogen cyanide --> rn:R03524

rn:R09451 { Hexadecanoic acid } <-- [Reduced NADPH---hemoprotein reductase] -- rn:R11814 <-- Hydrogen cyanide --> rn:R03524

rn:R09452 { (9Z)-Octadecenoic acid } <-- [Reduced NADPH---hemoprotein reductase] -- rn:R11814 <-- Hydrogen cyanide --> rn:R03524

rn:R09922 { (+)-Linalool } <-- [Reduced NADPH---hemoprotein reductase] -- rn:R11814 <-- Hydrogen cyanide --> rn:R03524

rn:R09923 { (-)-Linalool } <-- [Reduced NADPH---hemoprotein reductase] -- rn:R11814 <-- Hydrogen cyanide --> rn:R03524

rn:R09925 { Linalool } <-- [Reduced NADPH---hemoprotein reductase] -- rn:R11814 <-- Hydrogen cyanide --> rn:R03524

rn:R09934 { Humulene } <-- [Reduced NADPH---hemoprotein reductase] -- rn:R11814 <-- Hydrogen cyanide --> rn:R03524

rn:R10562 { (E,E)-Geranyllinalool } <-- [Reduced NADPH---hemoprotein reductase] -- rn:R11814 <-- Hydrogen cyanide --> rn:R03524

rn:R11055 { Myrcene } <-- [Reduced NADPH---hemoprotein reductase] -- rn:R11814 <-- Hydrogen cyanide --> rn:R03524

rn:R02468 { (-)-Limonene } <-- [Reduced NADPH---hemoprotein reductase] -- rn:R11814 <-- [Oxidized NADPH---hemoprotein reductase] --> rn:R08551

rn:R02469 { (-)-Limonene } <-- [Reduced NADPH---hemoprotein reductase] -- rn:R11814 <-- [Oxidized NADPH---hemoprotein reductase] --> rn:R08551

rn:R02470 { (-)-Limonene } <-- [Reduced NADPH---hemoprotein reductase] -- rn:R11814 <-- [Oxidized NADPH---hemoprotein reductase] --> rn:R08551

rn:R04366 { Linalool } <-- [Reduced NADPH---hemoprotein reductase] -- rn:R11814 <-- [Oxidized NADPH---hemoprotein reductase] --> rn:R08551

rn:R06119 { d-Limonene } <-- [Reduced NADPH---hemoprotein reductase] -- rn:R11814 <-- [Oxidized NADPH---hemoprotein reductase] --> rn:R08551

rn:R09451 { Hexadecanoic acid } <-- [Reduced NADPH---hemoprotein reductase] -- rn:R11814 <-- [Oxidized NADPH---hemoprotein reductase] --> rn:R08551

rn:R09452 { (9Z)-Octadecenoic acid } <-- [Reduced NADPH---hemoprotein reductase] -- rn:R11814 <-- [Oxidized NADPH---hemoprotein reductase] --> rn:R08551

rn:R09922 { (+)-Linalool } <-- [Reduced NADPH---hemoprotein reductase] -- rn:R11814 <-- [Oxidized NADPH---hemoprotein reductase] --> rn:R08551

rn:R09923 { (-)-Linalool } <-- [Reduced NADPH---hemoprotein reductase] -- rn:R11814 <-- [Oxidized NADPH---hemoprotein reductase] --> rn:R08551

rn:R09925 { Linalool } <-- [Reduced NADPH---hemoprotein reductase] -- rn:R11814 <-- [Oxidized NADPH---hemoprotein reductase] --> rn:R08551

rn:R09934 { Humulene } <-- [Reduced NADPH---hemoprotein reductase] -- rn:R11814 <-- [Oxidized NADPH---hemoprotein reductase] --> rn:R08551

rn:R10562 { (E,E)-Geranyllinalool } <-- [Reduced NADPH---hemoprotein reductase] -- rn:R11814 <-- [Oxidized NADPH---hemoprotein reductase] --> rn:R08551

rn:R11055 { Myrcene } <-- [Reduced NADPH---hemoprotein reductase] -- rn:R11814 <-- [Oxidized NADPH---hemoprotein reductase] --> rn:R08551

rn:R08530 { (-)-Menthol } <-- Acetyl-CoA -- rn:R11902 <-- Acetylhydrazine --> rn:R11903

rn:R08531 { (+)-Neomenthol } <-- Acetyl-CoA -- rn:R11902 <-- Acetylhydrazine --> rn:R11903

rn:R08532 { (+)-Borneol } <-- Acetyl-CoA -- rn:R11902 <-- Acetylhydrazine --> rn:R11903

rn:R10474 { Cinnamyl alcohol } <-- Acetyl-CoA -- rn:R11902 <-- Acetylhydrazine --> rn:R11903

rn:R08530 { (-)-Menthol } <-- Acetyl-CoA -- rn:R11902 <-- Acetylhydrazine --> rn:R11904

rn:R08531 { (+)-Neomenthol } <-- Acetyl-CoA -- rn:R11902 <-- Acetylhydrazine --> rn:R11904

rn:R08532 { (+)-Borneol } <-- Acetyl-CoA -- rn:R11902 <-- Acetylhydrazine --> rn:R11904

rn:R10474 { Cinnamyl alcohol } <-- Acetyl-CoA -- rn:R11902 <-- Acetylhydrazine --> rn:R11904

rn:R11068 { 3-[(1R,2S,5R,6S)-5-Hydroxy-7-oxabicyclo[4.1.0]heptan-2-yl]-2-oxopropanoate } <-- L-Phenylalanine -- rn:R11918 <-- Hydrogen peroxide --> rn:R00069

rn:R11068 { 3-[(1R,2S,5R,6S)-5-Hydroxy-7-oxabicyclo[4.1.0]heptan-2-yl]-2-oxopropanoate } <-- L-Phenylalanine -- rn:R11918 <-- Hydrogen peroxide --> rn:R00113

rn:R11068 { 3-[(1R,2S,5R,6S)-5-Hydroxy-7-oxabicyclo[4.1.0]heptan-2-yl]-2-oxopropanoate } <-- L-Phenylalanine -- rn:R11918 <-- Hydrogen peroxide --> rn:R00274

rn:R11068 { 3-[(1R,2S,5R,6S)-5-Hydroxy-7-oxabicyclo[4.1.0]heptan-2-yl]-2-oxopropanoate } <-- L-Phenylalanine -- rn:R11918 <-- Hydrogen peroxide --> rn:R00644

rn:R11068 { 3-[(1R,2S,5R,6S)-5-Hydroxy-7-oxabicyclo[4.1.0]heptan-2-yl]-2-oxopropanoate } <-- L-Phenylalanine -- rn:R11918 <-- Phenylacetaldehyde --> rn:R02536

rn:R11068 { 3-[(1R,2S,5R,6S)-5-Hydroxy-7-oxabicyclo[4.1.0]heptan-2-yl]-2-oxopropanoate } <-- L-Phenylalanine -- rn:R11918 <-- Phenylacetaldehyde --> rn:R02537

rn:R11068 { 3-[(1R,2S,5R,6S)-5-Hydroxy-7-oxabicyclo[4.1.0]heptan-2-yl]-2-oxopropanoate } <-- L-Phenylalanine -- rn:R11918 <-- Hydrogen peroxide --> rn:R02657

rn:R11068 { 3-[(1R,2S,5R,6S)-5-Hydroxy-7-oxabicyclo[4.1.0]heptan-2-yl]-2-oxopropanoate } <-- L-Phenylalanine -- rn:R11918 <-- Hydrogen peroxide --> rn:R03208

rn:R11068 { 3-[(1R,2S,5R,6S)-5-Hydroxy-7-oxabicyclo[4.1.0]heptan-2-yl]-2-oxopropanoate } <-- L-Phenylalanine -- rn:R11918 <-- Hydrogen peroxide --> rn:R03953

rn:R11068 { 3-[(1R,2S,5R,6S)-5-Hydroxy-7-oxabicyclo[4.1.0]heptan-2-yl]-2-oxopropanoate } <-- L-Phenylalanine -- rn:R11918 <-- Hydrogen peroxide --> rn:R08863

rn:R11068 { 3-[(1R,2S,5R,6S)-5-Hydroxy-7-oxabicyclo[4.1.0]heptan-2-yl]-2-oxopropanoate } <-- L-Phenylalanine -- rn:R11918 <-- Hydrogen peroxide --> rn:R11503

rn:R11068 { 3-[(1R,2S,5R,6S)-5-Hydroxy-7-oxabicyclo[4.1.0]heptan-2-yl]-2-oxopropanoate } <-- L-Phenylalanine -- rn:R11918 <-- Hydrogen peroxide --> rn:R11522

rn:R05488 { Styrene } <-- FADH2 -- rn:R12021 <-- FAD --> rn:R02487

rn:R05488 { Styrene } <-- FADH2 -- rn:R12021 <-- FAD --> rn:R04095

rn:R05488 { Styrene } <-- FADH2 -- rn:R12021 <-- FAD --> rn:R05537

rn:R05488 { Styrene } <-- FADH2 -- rn:R12021 <-- FAD --> rn:R06943

rn:R05488 { Styrene } <-- FADH2 -- rn:R12021 <-- FAD --> rn:R07220

rn:R05488 { Styrene } <-- FADH2 -- rn:R12021 <-- FAD --> rn:R09520

rn:R05488 { Styrene } <-- FADH2 -- rn:R12021 <-- FAD --> rn:R11130

rn:R05488 { Styrene } <-- FADH2 -- rn:R12023 <-- FAD --> rn:R02487

rn:R05488 { Styrene } <-- FADH2 -- rn:R12023 <-- FAD --> rn:R04095

rn:R05488 { Styrene } <-- FADH2 -- rn:R12023 <-- FAD --> rn:R05537

rn:R05488 { Styrene } <-- FADH2 -- rn:R12023 <-- FAD --> rn:R06943

rn:R05488 { Styrene } <-- FADH2 -- rn:R12023 <-- FAD --> rn:R07220

rn:R05488 { Styrene } <-- FADH2 -- rn:R12023 <-- FAD --> rn:R09520

rn:R05488 { Styrene } <-- FADH2 -- rn:R12023 <-- FAD --> rn:R11130

rn:R05488 { Styrene } <-- FADH2 -- rn:R12027 <-- FAD --> rn:R02487

rn:R05488 { Styrene } <-- FADH2 -- rn:R12027 <-- FAD --> rn:R04095

rn:R05488 { Styrene } <-- FADH2 -- rn:R12027 <-- FAD --> rn:R05537

rn:R05488 { Styrene } <-- FADH2 -- rn:R12027 <-- FAD --> rn:R06943

rn:R05488 { Styrene } <-- FADH2 -- rn:R12027 <-- FAD --> rn:R07220

rn:R05488 { Styrene } <-- FADH2 -- rn:R12027 <-- FAD --> rn:R09520

rn:R05488 { Styrene } <-- FADH2 -- rn:R12027 <-- FAD --> rn:R11130

rn:R05488 { Styrene } <-- FADH2 -- rn:R12030 <-- FAD --> rn:R02487

rn:R05488 { Styrene } <-- FADH2 -- rn:R12030 <-- FAD --> rn:R04095

rn:R05488 { Styrene } <-- FADH2 -- rn:R12030 <-- FAD --> rn:R05537

rn:R05488 { Styrene } <-- FADH2 -- rn:R12030 <-- FAD --> rn:R06943

rn:R05488 { Styrene } <-- FADH2 -- rn:R12030 <-- FAD --> rn:R07220

rn:R05488 { Styrene } <-- FADH2 -- rn:R12030 <-- FAD --> rn:R09520

rn:R05488 { Styrene } <-- FADH2 -- rn:R12030 <-- FAD --> rn:R11130

rn:R02468 { (-)-Limonene } <-- [Reduced NADPH---hemoprotein reductase] -- rn:R12072 <-- [Oxidized NADPH---hemoprotein reductase] --> rn:R08551

rn:R02469 { (-)-Limonene } <-- [Reduced NADPH---hemoprotein reductase] -- rn:R12072 <-- [Oxidized NADPH---hemoprotein reductase] --> rn:R08551

rn:R02470 { (-)-Limonene } <-- [Reduced NADPH---hemoprotein reductase] -- rn:R12072 <-- [Oxidized NADPH---hemoprotein reductase] --> rn:R08551

rn:R04366 { Linalool } <-- [Reduced NADPH---hemoprotein reductase] -- rn:R12072 <-- [Oxidized NADPH---hemoprotein reductase] --> rn:R08551

rn:R06119 { d-Limonene } <-- [Reduced NADPH---hemoprotein reductase] -- rn:R12072 <-- [Oxidized NADPH---hemoprotein reductase] --> rn:R08551

rn:R09451 { Hexadecanoic acid } <-- [Reduced NADPH---hemoprotein reductase] -- rn:R12072 <-- [Oxidized NADPH---hemoprotein reductase] --> rn:R08551

rn:R09452 { (9Z)-Octadecenoic acid } <-- [Reduced NADPH---hemoprotein reductase] -- rn:R12072 <-- [Oxidized NADPH---hemoprotein reductase] --> rn:R08551

rn:R09922 { (+)-Linalool } <-- [Reduced NADPH---hemoprotein reductase] -- rn:R12072 <-- [Oxidized NADPH---hemoprotein reductase] --> rn:R08551

rn:R09923 { (-)-Linalool } <-- [Reduced NADPH---hemoprotein reductase] -- rn:R12072 <-- [Oxidized NADPH---hemoprotein reductase] --> rn:R08551

rn:R09925 { Linalool } <-- [Reduced NADPH---hemoprotein reductase] -- rn:R12072 <-- [Oxidized NADPH---hemoprotein reductase] --> rn:R08551

rn:R09934 { Humulene } <-- [Reduced NADPH---hemoprotein reductase] -- rn:R12072 <-- [Oxidized NADPH---hemoprotein reductase] --> rn:R08551

rn:R10562 { (E,E)-Geranyllinalool } <-- [Reduced NADPH---hemoprotein reductase] -- rn:R12072 <-- [Oxidized NADPH---hemoprotein reductase] --> rn:R08551

rn:R11055 { Myrcene } <-- [Reduced NADPH---hemoprotein reductase] -- rn:R12072 <-- [Oxidized NADPH---hemoprotein reductase] --> rn:R08551

rn:R02468 { (-)-Limonene } <-- [Reduced NADPH---hemoprotein reductase] -- rn:R12183 <-- Cobamide coenzyme --> rn:R05223

rn:R02469 { (-)-Limonene } <-- [Reduced NADPH---hemoprotein reductase] -- rn:R12183 <-- Cobamide coenzyme --> rn:R05223

rn:R02470 { (-)-Limonene } <-- [Reduced NADPH---hemoprotein reductase] -- rn:R12183 <-- Cobamide coenzyme --> rn:R05223

rn:R04366 { Linalool } <-- [Reduced NADPH---hemoprotein reductase] -- rn:R12183 <-- Cobamide coenzyme --> rn:R05223

rn:R06119 { d-Limonene } <-- [Reduced NADPH---hemoprotein reductase] -- rn:R12183 <-- Cobamide coenzyme --> rn:R05223

rn:R09451 { Hexadecanoic acid } <-- [Reduced NADPH---hemoprotein reductase] -- rn:R12183 <-- Cobamide coenzyme --> rn:R05223

rn:R09452 { (9Z)-Octadecenoic acid } <-- [Reduced NADPH---hemoprotein reductase] -- rn:R12183 <-- Cobamide coenzyme --> rn:R05223

rn:R09922 { (+)-Linalool } <-- [Reduced NADPH---hemoprotein reductase] -- rn:R12183 <-- Cobamide coenzyme --> rn:R05223

rn:R09923 { (-)-Linalool } <-- [Reduced NADPH---hemoprotein reductase] -- rn:R12183 <-- Cobamide coenzyme --> rn:R05223

rn:R09925 { Linalool } <-- [Reduced NADPH---hemoprotein reductase] -- rn:R12183 <-- Cobamide coenzyme --> rn:R05223

rn:R09934 { Humulene } <-- [Reduced NADPH---hemoprotein reductase] -- rn:R12183 <-- Cobamide coenzyme --> rn:R05223

rn:R10562 { (E,E)-Geranyllinalool } <-- [Reduced NADPH---hemoprotein reductase] -- rn:R12183 <-- Cobamide coenzyme --> rn:R05223

rn:R11055 { Myrcene } <-- [Reduced NADPH---hemoprotein reductase] -- rn:R12183 <-- Cobamide coenzyme --> rn:R05223

rn:R02468 { (-)-Limonene } <-- [Reduced NADPH---hemoprotein reductase] -- rn:R12183 <-- [Oxidized NADPH---hemoprotein reductase] --> rn:R08551

rn:R02469 { (-)-Limonene } <-- [Reduced NADPH---hemoprotein reductase] -- rn:R12183 <-- [Oxidized NADPH---hemoprotein reductase] --> rn:R08551

rn:R02470 { (-)-Limonene } <-- [Reduced NADPH---hemoprotein reductase] -- rn:R12183 <-- [Oxidized NADPH---hemoprotein reductase] --> rn:R08551

rn:R04366 { Linalool } <-- [Reduced NADPH---hemoprotein reductase] -- rn:R12183 <-- [Oxidized NADPH---hemoprotein reductase] --> rn:R08551

rn:R06119 { d-Limonene } <-- [Reduced NADPH---hemoprotein reductase] -- rn:R12183 <-- [Oxidized NADPH---hemoprotein reductase] --> rn:R08551

rn:R09451 { Hexadecanoic acid } <-- [Reduced NADPH---hemoprotein reductase] -- rn:R12183 <-- [Oxidized NADPH---hemoprotein reductase] --> rn:R08551

rn:R09452 { (9Z)-Octadecenoic acid } <-- [Reduced NADPH---hemoprotein reductase] -- rn:R12183 <-- [Oxidized NADPH---hemoprotein reductase] --> rn:R08551

rn:R09922 { (+)-Linalool } <-- [Reduced NADPH---hemoprotein reductase] -- rn:R12183 <-- [Oxidized NADPH---hemoprotein reductase] --> rn:R08551

rn:R09923 { (-)-Linalool } <-- [Reduced NADPH---hemoprotein reductase] -- rn:R12183 <-- [Oxidized NADPH---hemoprotein reductase] --> rn:R08551

rn:R09925 { Linalool } <-- [Reduced NADPH---hemoprotein reductase] -- rn:R12183 <-- [Oxidized NADPH---hemoprotein reductase] --> rn:R08551

rn:R09934 { Humulene } <-- [Reduced NADPH---hemoprotein reductase] -- rn:R12183 <-- [Oxidized NADPH---hemoprotein reductase] --> rn:R08551

rn:R10562 { (E,E)-Geranyllinalool } <-- [Reduced NADPH---hemoprotein reductase] -- rn:R12183 <-- [Oxidized NADPH---hemoprotein reductase] --> rn:R08551

rn:R11055 { Myrcene } <-- [Reduced NADPH---hemoprotein reductase] -- rn:R12183 <-- [Oxidized NADPH---hemoprotein reductase] --> rn:R08551

rn:R02468 { (-)-Limonene } <-- [Reduced NADPH---hemoprotein reductase] -- rn:R12184 <-- Adenosyl cobyrinate a,c diamide --> rn:R05225

rn:R02469 { (-)-Limonene } <-- [Reduced NADPH---hemoprotein reductase] -- rn:R12184 <-- Adenosyl cobyrinate a,c diamide --> rn:R05225

rn:R02470 { (-)-Limonene } <-- [Reduced NADPH---hemoprotein reductase] -- rn:R12184 <-- Adenosyl cobyrinate a,c diamide --> rn:R05225

rn:R04366 { Linalool } <-- [Reduced NADPH---hemoprotein reductase] -- rn:R12184 <-- Adenosyl cobyrinate a,c diamide --> rn:R05225

rn:R06119 { d-Limonene } <-- [Reduced NADPH---hemoprotein reductase] -- rn:R12184 <-- Adenosyl cobyrinate a,c diamide --> rn:R05225

rn:R09451 { Hexadecanoic acid } <-- [Reduced NADPH---hemoprotein reductase] -- rn:R12184 <-- Adenosyl cobyrinate a,c diamide --> rn:R05225

rn:R09452 { (9Z)-Octadecenoic acid } <-- [Reduced NADPH---hemoprotein reductase] -- rn:R12184 <-- Adenosyl cobyrinate a,c diamide --> rn:R05225

rn:R09922 { (+)-Linalool } <-- [Reduced NADPH---hemoprotein reductase] -- rn:R12184 <-- Adenosyl cobyrinate a,c diamide --> rn:R05225

rn:R09923 { (-)-Linalool } <-- [Reduced NADPH---hemoprotein reductase] -- rn:R12184 <-- Adenosyl cobyrinate a,c diamide --> rn:R05225

rn:R09925 { Linalool } <-- [Reduced NADPH---hemoprotein reductase] -- rn:R12184 <-- Adenosyl cobyrinate a,c diamide --> rn:R05225

rn:R09934 { Humulene } <-- [Reduced NADPH---hemoprotein reductase] -- rn:R12184 <-- Adenosyl cobyrinate a,c diamide --> rn:R05225

rn:R10562 { (E,E)-Geranyllinalool } <-- [Reduced NADPH---hemoprotein reductase] -- rn:R12184 <-- Adenosyl cobyrinate a,c diamide --> rn:R05225

rn:R11055 { Myrcene } <-- [Reduced NADPH---hemoprotein reductase] -- rn:R12184 <-- Adenosyl cobyrinate a,c diamide --> rn:R05225

rn:R02468 { (-)-Limonene } <-- [Reduced NADPH---hemoprotein reductase] -- rn:R12184 <-- [Oxidized NADPH---hemoprotein reductase] --> rn:R08551

rn:R02469 { (-)-Limonene } <-- [Reduced NADPH---hemoprotein reductase] -- rn:R12184 <-- [Oxidized NADPH---hemoprotein reductase] --> rn:R08551

rn:R02470 { (-)-Limonene } <-- [Reduced NADPH---hemoprotein reductase] -- rn:R12184 <-- [Oxidized NADPH---hemoprotein reductase] --> rn:R08551

rn:R04366 { Linalool } <-- [Reduced NADPH---hemoprotein reductase] -- rn:R12184 <-- [Oxidized NADPH---hemoprotein reductase] --> rn:R08551

rn:R06119 { d-Limonene } <-- [Reduced NADPH---hemoprotein reductase] -- rn:R12184 <-- [Oxidized NADPH---hemoprotein reductase] --> rn:R08551

rn:R09451 { Hexadecanoic acid } <-- [Reduced NADPH---hemoprotein reductase] -- rn:R12184 <-- [Oxidized NADPH---hemoprotein reductase] --> rn:R08551

rn:R09452 { (9Z)-Octadecenoic acid } <-- [Reduced NADPH---hemoprotein reductase] -- rn:R12184 <-- [Oxidized NADPH---hemoprotein reductase] --> rn:R08551

rn:R09922 { (+)-Linalool } <-- [Reduced NADPH---hemoprotein reductase] -- rn:R12184 <-- [Oxidized NADPH---hemoprotein reductase] --> rn:R08551

rn:R09923 { (-)-Linalool } <-- [Reduced NADPH---hemoprotein reductase] -- rn:R12184 <-- [Oxidized NADPH---hemoprotein reductase] --> rn:R08551

rn:R09925 { Linalool } <-- [Reduced NADPH---hemoprotein reductase] -- rn:R12184 <-- [Oxidized NADPH---hemoprotein reductase] --> rn:R08551

rn:R09934 { Humulene } <-- [Reduced NADPH---hemoprotein reductase] -- rn:R12184 <-- [Oxidized NADPH---hemoprotein reductase] --> rn:R08551

rn:R10562 { (E,E)-Geranyllinalool } <-- [Reduced NADPH---hemoprotein reductase] -- rn:R12184 <-- [Oxidized NADPH---hemoprotein reductase] --> rn:R08551

rn:R11055 { Myrcene } <-- [Reduced NADPH---hemoprotein reductase] -- rn:R12184 <-- [Oxidized NADPH---hemoprotein reductase] --> rn:R08551
