# Supplementary material for: The impact of Traditional Chinese Medicine on mouse gut microbiota abundances and interactions based on Granger causality and pathway analysis
Source: Front Microbiol. 2022 Nov 11;13:980082. doi: 10.3389/fmicb.2022.980082 (PMC9692106; doi:10.3389/fmicb.2022.980082)
Supplement: Supplementary file 4 [file Table_4.doc]

rn:R00008 <-- Pyruvate --> rn:R00210 -- Acetyl-CoA --> rn:R08530 { (-)-Menthol }

rn:R00008 <-- Pyruvate --> rn:R00210 -- Acetyl-CoA --> rn:R08531 { (+)-Neomenthol }

rn:R00008 <-- Pyruvate --> rn:R00210 -- Acetyl-CoA --> rn:R08532 { (+)-Borneol }

rn:R00008 <-- Pyruvate --> rn:R00210 -- Acetyl-CoA --> rn:R10474 { Cinnamyl alcohol }

rn:R00031 <-- 3,4-Dihydroxy-L-phenylalanine --> rn:R02080 -- Dopamine --> rn:R08447 { 4-Hydroxydihydrocinnamaldehyde }

rn:R00031 <-- 3,4-Dihydroxy-L-phenylalanine --> rn:R11916 -- Hydrogen peroxide --> rn:R01703 { Hexadecanoic acid }

rn:R00062 <-- Bilirubin beta-diglucuronide --> rn:R04979 -- Acceptor --> rn:R02234 { Cyclohexanone }

rn:R00062 <-- Bilirubin beta-diglucuronide --> rn:R04979 -- Acceptor --> rn:R03212 { 3-Hydroxycyclohexanone }

rn:R00114 <-- L-Glutamine --> rn:R00986 -- Pyruvate --> rn:R05136 { Salicylaldehyde }

rn:R00114 <-- L-Glutamine --> rn:R01375 -- L-Phenylalanine --> rn:R11068 { 3-[(1R,2S,5R,6S)-5-Hydroxy-7-oxabicyclo[4.1.0]heptan-2-yl]-2-oxopropanoate }

rn:R00146 <-- 2-Oxo acid --> rn:R04913 -- Amino acid --> rn:R11672 { (S)-3-Acetyloctanal }

rn:R00173 <-- Pyridoxal --> rn:R01709 -- Hydrogen peroxide --> rn:R01703 { Hexadecanoic acid }

rn:R00205 <-- Pyruvate --> rn:R00210 -- Acetyl-CoA --> rn:R08530 { (-)-Menthol }

rn:R00205 <-- Pyruvate --> rn:R00210 -- Acetyl-CoA --> rn:R08531 { (+)-Neomenthol }

rn:R00205 <-- Pyruvate --> rn:R00210 -- Acetyl-CoA --> rn:R08532 { (+)-Borneol }

rn:R00205 <-- Pyruvate --> rn:R00210 -- Acetyl-CoA --> rn:R10474 { Cinnamyl alcohol }

rn:R00216 <-- Pyruvate --> rn:R00210 -- Acetyl-CoA --> rn:R08530 { (-)-Menthol }

rn:R00216 <-- Pyruvate --> rn:R00210 -- Acetyl-CoA --> rn:R08531 { (+)-Neomenthol }

rn:R00216 <-- Pyruvate --> rn:R00210 -- Acetyl-CoA --> rn:R08532 { (+)-Borneol }

rn:R00216 <-- Pyruvate --> rn:R00210 -- Acetyl-CoA --> rn:R10474 { Cinnamyl alcohol }

rn:R00237 <-- Pyruvate --> rn:R00210 -- Acetyl-CoA --> rn:R08530 { (-)-Menthol }

rn:R00237 <-- Pyruvate --> rn:R00210 -- Acetyl-CoA --> rn:R08531 { (+)-Neomenthol }

rn:R00237 <-- Pyruvate --> rn:R00210 -- Acetyl-CoA --> rn:R08532 { (+)-Borneol }

rn:R00237 <-- Pyruvate --> rn:R00210 -- Acetyl-CoA --> rn:R10474 { Cinnamyl alcohol }

rn:R00311 <-- [Oxidized NADPH---hemoprotein reductase] --> rn:R08551 -- [Reduced NADPH---hemoprotein reductase] --> rn:R02468 { (-)-Limonene }

rn:R00311 <-- [Oxidized NADPH---hemoprotein reductase] --> rn:R08551 -- [Reduced NADPH---hemoprotein reductase] --> rn:R02469 { (-)-Limonene }

rn:R00311 <-- [Oxidized NADPH---hemoprotein reductase] --> rn:R08551 -- [Reduced NADPH---hemoprotein reductase] --> rn:R02470 { (-)-Limonene }

rn:R00311 <-- [Oxidized NADPH---hemoprotein reductase] --> rn:R08551 -- [Reduced NADPH---hemoprotein reductase] --> rn:R04366 { Linalool }

rn:R00311 <-- [Oxidized NADPH---hemoprotein reductase] --> rn:R08551 -- [Reduced NADPH---hemoprotein reductase] --> rn:R06119 { d-Limonene }

rn:R00311 <-- [Oxidized NADPH---hemoprotein reductase] --> rn:R08551 -- [Reduced NADPH---hemoprotein reductase] --> rn:R09451 { Hexadecanoic acid }

rn:R00311 <-- [Oxidized NADPH---hemoprotein reductase] --> rn:R08551 -- [Reduced NADPH---hemoprotein reductase] --> rn:R09452 { (9Z)-Octadecenoic acid }

rn:R00311 <-- [Oxidized NADPH---hemoprotein reductase] --> rn:R08551 -- [Reduced NADPH---hemoprotein reductase] --> rn:R09922 { (+)-Linalool }

rn:R00311 <-- [Oxidized NADPH---hemoprotein reductase] --> rn:R08551 -- [Reduced NADPH---hemoprotein reductase] --> rn:R09923 { (-)-Linalool }

rn:R00311 <-- [Oxidized NADPH---hemoprotein reductase] --> rn:R08551 -- [Reduced NADPH---hemoprotein reductase] --> rn:R09925 { Linalool }

rn:R00311 <-- [Oxidized NADPH---hemoprotein reductase] --> rn:R08551 -- [Reduced NADPH---hemoprotein reductase] --> rn:R09934 { Humulene }

rn:R00311 <-- [Oxidized NADPH---hemoprotein reductase] --> rn:R08551 -- [Reduced NADPH---hemoprotein reductase] --> rn:R10562 { (E,E)-Geranyllinalool }

rn:R00311 <-- [Oxidized NADPH---hemoprotein reductase] --> rn:R08551 -- [Reduced NADPH---hemoprotein reductase] --> rn:R11055 { Myrcene }

rn:R00318 <-- Acetate --> rn:R01241 -- Phenyl acetate --> rn:R07342 { Phenyl acetate }

rn:R00452 <-- Pyruvate --> rn:R00210 -- Acetyl-CoA --> rn:R08530 { (-)-Menthol }

rn:R00452 <-- Pyruvate --> rn:R00210 -- Acetyl-CoA --> rn:R08531 { (+)-Neomenthol }

rn:R00452 <-- Pyruvate --> rn:R00210 -- Acetyl-CoA --> rn:R08532 { (+)-Borneol }

rn:R00452 <-- Pyruvate --> rn:R00210 -- Acetyl-CoA --> rn:R10474 { Cinnamyl alcohol }

rn:R00486 <-- L-Aspartate --> rn:R00695 -- L-Phenylalanine --> rn:R11068 { 3-[(1R,2S,5R,6S)-5-Hydroxy-7-oxabicyclo[4.1.0]heptan-2-yl]-2-oxopropanoate }

rn:R00494 <-- L-Glutamate --> rn:R00114 -- L-Glutamine --> rn:R02781 { 2,4,6/3,5-Pentahydroxycyclohexanone }

rn:R00497 <-- Glutathione --> rn:R00120 -- Hydrogen peroxide --> rn:R01703 { Hexadecanoic acid }

rn:R00525 <-- L-Glutamate --> rn:R00114 -- L-Glutamine --> rn:R02781 { 2,4,6/3,5-Pentahydroxycyclohexanone }

rn:R00526 <-- L-Aspartate --> rn:R00695 -- L-Phenylalanine --> rn:R11068 { 3-[(1R,2S,5R,6S)-5-Hydroxy-7-oxabicyclo[4.1.0]heptan-2-yl]-2-oxopropanoate }

rn:R00661 <-- 3-Phosphonopyruvate --> rn:R08200 -- Pyruvate --> rn:R05136 { Salicylaldehyde }

rn:R00666 <-- Pyruvate --> rn:R00210 -- Acetyl-CoA --> rn:R08530 { (-)-Menthol }

rn:R00666 <-- Pyruvate --> rn:R00210 -- Acetyl-CoA --> rn:R08531 { (+)-Neomenthol }

rn:R00666 <-- Pyruvate --> rn:R00210 -- Acetyl-CoA --> rn:R08532 { (+)-Borneol }

rn:R00666 <-- Pyruvate --> rn:R00210 -- Acetyl-CoA --> rn:R10474 { Cinnamyl alcohol }

rn:R00688 <-- Phenylpyruvate --> rn:R00695 -- L-Phenylalanine --> rn:R11068 { 3-[(1R,2S,5R,6S)-5-Hydroxy-7-oxabicyclo[4.1.0]heptan-2-yl]-2-oxopropanoate }

rn:R00688 <-- Phenylpyruvate --> rn:R01372 -- 2-Hydroxyphenylacetate --> rn:R05001 { 2-Hydroxyphenylacetate }

rn:R00688 <-- Phenylpyruvate --> rn:R01372 -- 2-Hydroxyphenylacetate --> rn:R05450 { 2-Hydroxyphenylacetate }

rn:R00688 <-- Phenylpyruvate --> rn:R01375 -- L-Phenylalanine --> rn:R11068 { 3-[(1R,2S,5R,6S)-5-Hydroxy-7-oxabicyclo[4.1.0]heptan-2-yl]-2-oxopropanoate }

rn:R00688 <-- Phenylpyruvate --> rn:R01376 -- L-Phenylalanine --> rn:R11068 { 3-[(1R,2S,5R,6S)-5-Hydroxy-7-oxabicyclo[4.1.0]heptan-2-yl]-2-oxopropanoate }

rn:R00689 <-- Phenylpyruvate --> rn:R00695 -- L-Phenylalanine --> rn:R11068 { 3-[(1R,2S,5R,6S)-5-Hydroxy-7-oxabicyclo[4.1.0]heptan-2-yl]-2-oxopropanoate }

rn:R00689 <-- Phenylpyruvate --> rn:R01372 -- 2-Hydroxyphenylacetate --> rn:R05001 { 2-Hydroxyphenylacetate }

rn:R00689 <-- Phenylpyruvate --> rn:R01372 -- 2-Hydroxyphenylacetate --> rn:R05450 { 2-Hydroxyphenylacetate }

rn:R00689 <-- Phenylpyruvate --> rn:R01375 -- L-Phenylalanine --> rn:R11068 { 3-[(1R,2S,5R,6S)-5-Hydroxy-7-oxabicyclo[4.1.0]heptan-2-yl]-2-oxopropanoate }

rn:R00689 <-- Phenylpyruvate --> rn:R01376 -- L-Phenylalanine --> rn:R11068 { 3-[(1R,2S,5R,6S)-5-Hydroxy-7-oxabicyclo[4.1.0]heptan-2-yl]-2-oxopropanoate }

rn:R00692 <-- Phenylpyruvate --> rn:R00695 -- L-Phenylalanine --> rn:R11068 { 3-[(1R,2S,5R,6S)-5-Hydroxy-7-oxabicyclo[4.1.0]heptan-2-yl]-2-oxopropanoate }

rn:R00692 <-- Phenylpyruvate --> rn:R01372 -- 2-Hydroxyphenylacetate --> rn:R05001 { 2-Hydroxyphenylacetate }

rn:R00692 <-- Phenylpyruvate --> rn:R01372 -- 2-Hydroxyphenylacetate --> rn:R05450 { 2-Hydroxyphenylacetate }

rn:R00692 <-- Phenylpyruvate --> rn:R01375 -- L-Phenylalanine --> rn:R11068 { 3-[(1R,2S,5R,6S)-5-Hydroxy-7-oxabicyclo[4.1.0]heptan-2-yl]-2-oxopropanoate }

rn:R00692 <-- Phenylpyruvate --> rn:R01376 -- L-Phenylalanine --> rn:R11068 { 3-[(1R,2S,5R,6S)-5-Hydroxy-7-oxabicyclo[4.1.0]heptan-2-yl]-2-oxopropanoate }

rn:R00692 <-- L-Alanine --> rn:R04187 -- Pyruvate --> rn:R05136 { Salicylaldehyde }

rn:R00694 <-- L-Glutamate --> rn:R00114 -- L-Glutamine --> rn:R02781 { 2,4,6/3,5-Pentahydroxycyclohexanone }

rn:R00694 <-- Phenylpyruvate --> rn:R00695 -- L-Phenylalanine --> rn:R11068 { 3-[(1R,2S,5R,6S)-5-Hydroxy-7-oxabicyclo[4.1.0]heptan-2-yl]-2-oxopropanoate }

rn:R00694 <-- Phenylpyruvate --> rn:R01372 -- 2-Hydroxyphenylacetate --> rn:R05001 { 2-Hydroxyphenylacetate }

rn:R00694 <-- Phenylpyruvate --> rn:R01372 -- 2-Hydroxyphenylacetate --> rn:R05450 { 2-Hydroxyphenylacetate }

rn:R00694 <-- Phenylpyruvate --> rn:R01375 -- L-Phenylalanine --> rn:R11068 { 3-[(1R,2S,5R,6S)-5-Hydroxy-7-oxabicyclo[4.1.0]heptan-2-yl]-2-oxopropanoate }

rn:R00694 <-- Phenylpyruvate --> rn:R01376 -- L-Phenylalanine --> rn:R11068 { 3-[(1R,2S,5R,6S)-5-Hydroxy-7-oxabicyclo[4.1.0]heptan-2-yl]-2-oxopropanoate }

rn:R00695 <-- L-Phenylalanine --> rn:R00689 -- Hydrogen peroxide --> rn:R01703 { Hexadecanoic acid }

rn:R00695 <-- L-Phenylalanine --> rn:R11918 -- Hydrogen peroxide --> rn:R01703 { Hexadecanoic acid }

rn:R00697 <-- trans-Cinnamate --> rn:R11070 -- Styrene --> rn:R05417 { Styrene }

rn:R00697 <-- trans-Cinnamate --> rn:R11070 -- Styrene --> rn:R05488 { Styrene }

rn:R00699 <-- Phenethylamine --> rn:R02613 -- Hydrogen peroxide --> rn:R01703 { Hexadecanoic acid }

rn:R00708 <-- L-Glutamate --> rn:R00114 -- L-Glutamine --> rn:R02781 { 2,4,6/3,5-Pentahydroxycyclohexanone }

rn:R00711 <-- Acetate --> rn:R01241 -- Phenyl acetate --> rn:R07342 { Phenyl acetate }

rn:R00730 <-- [Oxidized NADPH---hemoprotein reductase] --> rn:R08551 -- [Reduced NADPH---hemoprotein reductase] --> rn:R02468 { (-)-Limonene }

rn:R00730 <-- [Oxidized NADPH---hemoprotein reductase] --> rn:R08551 -- [Reduced NADPH---hemoprotein reductase] --> rn:R02469 { (-)-Limonene }

rn:R00730 <-- [Oxidized NADPH---hemoprotein reductase] --> rn:R08551 -- [Reduced NADPH---hemoprotein reductase] --> rn:R02470 { (-)-Limonene }

rn:R00730 <-- [Oxidized NADPH---hemoprotein reductase] --> rn:R08551 -- [Reduced NADPH---hemoprotein reductase] --> rn:R04366 { Linalool }

rn:R00730 <-- [Oxidized NADPH---hemoprotein reductase] --> rn:R08551 -- [Reduced NADPH---hemoprotein reductase] --> rn:R06119 { d-Limonene }

rn:R00730 <-- [Oxidized NADPH---hemoprotein reductase] --> rn:R08551 -- [Reduced NADPH---hemoprotein reductase] --> rn:R09451 { Hexadecanoic acid }

rn:R00730 <-- [Oxidized NADPH---hemoprotein reductase] --> rn:R08551 -- [Reduced NADPH---hemoprotein reductase] --> rn:R09452 { (9Z)-Octadecenoic acid }

rn:R00730 <-- [Oxidized NADPH---hemoprotein reductase] --> rn:R08551 -- [Reduced NADPH---hemoprotein reductase] --> rn:R09922 { (+)-Linalool }

rn:R00730 <-- [Oxidized NADPH---hemoprotein reductase] --> rn:R08551 -- [Reduced NADPH---hemoprotein reductase] --> rn:R09923 { (-)-Linalool }

rn:R00730 <-- [Oxidized NADPH---hemoprotein reductase] --> rn:R08551 -- [Reduced NADPH---hemoprotein reductase] --> rn:R09925 { Linalool }

rn:R00730 <-- [Oxidized NADPH---hemoprotein reductase] --> rn:R08551 -- [Reduced NADPH---hemoprotein reductase] --> rn:R09934 { Humulene }

rn:R00730 <-- [Oxidized NADPH---hemoprotein reductase] --> rn:R08551 -- [Reduced NADPH---hemoprotein reductase] --> rn:R10562 { (E,E)-Geranyllinalool }

rn:R00730 <-- [Oxidized NADPH---hemoprotein reductase] --> rn:R08551 -- [Reduced NADPH---hemoprotein reductase] --> rn:R11055 { Myrcene }

rn:R00731 <-- 3,4-Dihydroxy-L-phenylalanine --> rn:R02080 -- Dopamine --> rn:R08447 { 4-Hydroxydihydrocinnamaldehyde }

rn:R00731 <-- 3,4-Dihydroxy-L-phenylalanine --> rn:R11916 -- Hydrogen peroxide --> rn:R01703 { Hexadecanoic acid }

rn:R00732 <-- L-Tyrosine --> rn:R10246 -- Acceptor --> rn:R02234 { Cyclohexanone }

rn:R00732 <-- L-Tyrosine --> rn:R10246 -- Acceptor --> rn:R03212 { 3-Hydroxycyclohexanone }

rn:R00732 <-- L-Tyrosine --> rn:R11917 -- Hydrogen peroxide --> rn:R01703 { Hexadecanoic acid }

rn:R00733 <-- L-Tyrosine --> rn:R10246 -- Acceptor --> rn:R02234 { Cyclohexanone }

rn:R00733 <-- L-Tyrosine --> rn:R10246 -- Acceptor --> rn:R03212 { 3-Hydroxycyclohexanone }

rn:R00733 <-- L-Tyrosine --> rn:R11917 -- Hydrogen peroxide --> rn:R01703 { Hexadecanoic acid }

rn:R00899 <-- Glycine --> rn:R08701 -- Acceptor --> rn:R02234 { Cyclohexanone }

rn:R00899 <-- Glycine --> rn:R08701 -- Acceptor --> rn:R03212 { 3-Hydroxycyclohexanone }

rn:R00983 <-- Anthranilate --> rn:R09517 -- FAD --> rn:R05537 { 3-(2-Hydroxyphenyl)propanoate }

rn:R00986 <-- L-Glutamate --> rn:R00114 -- L-Glutamine --> rn:R02781 { 2,4,6/3,5-Pentahydroxycyclohexanone }

rn:R00986 <-- Pyruvate --> rn:R00210 -- Acetyl-CoA --> rn:R08530 { (-)-Menthol }

rn:R00986 <-- Pyruvate --> rn:R00210 -- Acetyl-CoA --> rn:R08531 { (+)-Neomenthol }

rn:R00986 <-- Pyruvate --> rn:R00210 -- Acetyl-CoA --> rn:R08532 { (+)-Borneol }

rn:R00986 <-- Pyruvate --> rn:R00210 -- Acetyl-CoA --> rn:R10474 { Cinnamyl alcohol }

rn:R00986 <-- Anthranilate --> rn:R09517 -- FAD --> rn:R05537 { 3-(2-Hydroxyphenyl)propanoate }

rn:R00987 <-- L-Alanine --> rn:R04187 -- Pyruvate --> rn:R05136 { Salicylaldehyde }

rn:R00987 <-- Anthranilate --> rn:R09517 -- FAD --> rn:R05537 { 3-(2-Hydroxyphenyl)propanoate }

rn:R00988 <-- Anthranilate --> rn:R09517 -- FAD --> rn:R05537 { 3-(2-Hydroxyphenyl)propanoate }

rn:R01033 <-- Cl- --> rn:R12021 -- FAD --> rn:R05537 { 3-(2-Hydroxyphenyl)propanoate }

rn:R01033 <-- Cl- --> rn:R12023 -- FAD --> rn:R05537 { 3-(2-Hydroxyphenyl)propanoate }

rn:R01073 <-- Anthranilate --> rn:R09517 -- FAD --> rn:R05537 { 3-(2-Hydroxyphenyl)propanoate }

rn:R01085 <-- Pyruvate --> rn:R00210 -- Acetyl-CoA --> rn:R08530 { (-)-Menthol }

rn:R01085 <-- Pyruvate --> rn:R00210 -- Acetyl-CoA --> rn:R08531 { (+)-Neomenthol }

rn:R01085 <-- Pyruvate --> rn:R00210 -- Acetyl-CoA --> rn:R08532 { (+)-Borneol }

rn:R01085 <-- Pyruvate --> rn:R00210 -- Acetyl-CoA --> rn:R10474 { Cinnamyl alcohol }

rn:R01111 <-- Glutathione --> rn:R00120 -- Hydrogen peroxide --> rn:R01703 { Hexadecanoic acid }

rn:R01161 <-- L-Glutamate --> rn:R00114 -- L-Glutamine --> rn:R02781 { 2,4,6/3,5-Pentahydroxycyclohexanone }

rn:R01167 <-- Histamine --> rn:R02150 -- Hydrogen peroxide --> rn:R01703 { Hexadecanoic acid }

rn:R01238 <-- Phenol --> rn:R01241 -- Phenyl acetate --> rn:R07342 { Phenyl acetate }

rn:R01238 <-- Phenol --> rn:R11653 -- FAD --> rn:R05537 { 3-(2-Hydroxyphenyl)propanoate }

rn:R01243 <-- Phenol --> rn:R01241 -- Phenyl acetate --> rn:R07342 { Phenyl acetate }

rn:R01243 <-- Phenol --> rn:R11653 -- FAD --> rn:R05537 { 3-(2-Hydroxyphenyl)propanoate }

rn:R01279 <-- FADH2 --> rn:R03978 -- FAD --> rn:R05537 { 3-(2-Hydroxyphenyl)propanoate }

rn:R01279 <-- FADH2 --> rn:R05488 -- FAD --> rn:R05537 { 3-(2-Hydroxyphenyl)propanoate }

rn:R01279 <-- FADH2 --> rn:R09517 -- FAD --> rn:R05537 { 3-(2-Hydroxyphenyl)propanoate }

rn:R01279 <-- FADH2 --> rn:R11653 -- FAD --> rn:R05537 { 3-(2-Hydroxyphenyl)propanoate }

rn:R01279 <-- FADH2 --> rn:R12021 -- FAD --> rn:R05537 { 3-(2-Hydroxyphenyl)propanoate }

rn:R01279 <-- FADH2 --> rn:R12023 -- FAD --> rn:R05537 { 3-(2-Hydroxyphenyl)propanoate }

rn:R01279 <-- FADH2 --> rn:R12027 -- FAD --> rn:R05537 { 3-(2-Hydroxyphenyl)propanoate }

rn:R01279 <-- FADH2 --> rn:R12030 -- FAD --> rn:R05537 { 3-(2-Hydroxyphenyl)propanoate }

rn:R01295 <-- [Oxidized NADPH---hemoprotein reductase] --> rn:R08551 -- [Reduced NADPH---hemoprotein reductase] --> rn:R02468 { (-)-Limonene }

rn:R01295 <-- [Oxidized NADPH---hemoprotein reductase] --> rn:R08551 -- [Reduced NADPH---hemoprotein reductase] --> rn:R02469 { (-)-Limonene }

rn:R01295 <-- [Oxidized NADPH---hemoprotein reductase] --> rn:R08551 -- [Reduced NADPH---hemoprotein reductase] --> rn:R02470 { (-)-Limonene }

rn:R01295 <-- [Oxidized NADPH---hemoprotein reductase] --> rn:R08551 -- [Reduced NADPH---hemoprotein reductase] --> rn:R04366 { Linalool }

rn:R01295 <-- [Oxidized NADPH---hemoprotein reductase] --> rn:R08551 -- [Reduced NADPH---hemoprotein reductase] --> rn:R06119 { d-Limonene }

rn:R01295 <-- [Oxidized NADPH---hemoprotein reductase] --> rn:R08551 -- [Reduced NADPH---hemoprotein reductase] --> rn:R09451 { Hexadecanoic acid }

rn:R01295 <-- [Oxidized NADPH---hemoprotein reductase] --> rn:R08551 -- [Reduced NADPH---hemoprotein reductase] --> rn:R09452 { (9Z)-Octadecenoic acid }

rn:R01295 <-- [Oxidized NADPH---hemoprotein reductase] --> rn:R08551 -- [Reduced NADPH---hemoprotein reductase] --> rn:R09922 { (+)-Linalool }

rn:R01295 <-- [Oxidized NADPH---hemoprotein reductase] --> rn:R08551 -- [Reduced NADPH---hemoprotein reductase] --> rn:R09923 { (-)-Linalool }

rn:R01295 <-- [Oxidized NADPH---hemoprotein reductase] --> rn:R08551 -- [Reduced NADPH---hemoprotein reductase] --> rn:R09925 { Linalool }

rn:R01295 <-- [Oxidized NADPH---hemoprotein reductase] --> rn:R08551 -- [Reduced NADPH---hemoprotein reductase] --> rn:R09934 { Humulene }

rn:R01295 <-- [Oxidized NADPH---hemoprotein reductase] --> rn:R08551 -- [Reduced NADPH---hemoprotein reductase] --> rn:R10562 { (E,E)-Geranyllinalool }

rn:R01295 <-- [Oxidized NADPH---hemoprotein reductase] --> rn:R08551 -- [Reduced NADPH---hemoprotein reductase] --> rn:R11055 { Myrcene }

rn:R01302 <-- Chorismate --> rn:R00986 -- Pyruvate --> rn:R05136 { Salicylaldehyde }

rn:R01302 <-- Chorismate --> rn:R06603 -- Pyruvate --> rn:R05136 { Salicylaldehyde }

rn:R01302 <-- Chorismate --> rn:R10583 -- Pyruvate --> rn:R05136 { Salicylaldehyde }

rn:R01307 <-- Cl- --> rn:R12021 -- FAD --> rn:R05537 { 3-(2-Hydroxyphenyl)propanoate }

rn:R01307 <-- Cl- --> rn:R12023 -- FAD --> rn:R05537 { 3-(2-Hydroxyphenyl)propanoate }

rn:R01317 <-- Arachidonate --> rn:R01599 -- Acceptor --> rn:R02234 { Cyclohexanone }

rn:R01317 <-- Arachidonate --> rn:R01599 -- Acceptor --> rn:R03212 { 3-Hydroxycyclohexanone }

rn:R01338 <-- 2-Oxo acid --> rn:R04913 -- Amino acid --> rn:R11672 { (S)-3-Acetyloctanal }

rn:R01348 <-- [Oxidized NADPH---hemoprotein reductase] --> rn:R08551 -- [Reduced NADPH---hemoprotein reductase] --> rn:R02468 { (-)-Limonene }

rn:R01348 <-- [Oxidized NADPH---hemoprotein reductase] --> rn:R08551 -- [Reduced NADPH---hemoprotein reductase] --> rn:R02469 { (-)-Limonene }

rn:R01348 <-- [Oxidized NADPH---hemoprotein reductase] --> rn:R08551 -- [Reduced NADPH---hemoprotein reductase] --> rn:R02470 { (-)-Limonene }

rn:R01348 <-- [Oxidized NADPH---hemoprotein reductase] --> rn:R08551 -- [Reduced NADPH---hemoprotein reductase] --> rn:R04366 { Linalool }

rn:R01348 <-- [Oxidized NADPH---hemoprotein reductase] --> rn:R08551 -- [Reduced NADPH---hemoprotein reductase] --> rn:R06119 { d-Limonene }

rn:R01348 <-- [Oxidized NADPH---hemoprotein reductase] --> rn:R08551 -- [Reduced NADPH---hemoprotein reductase] --> rn:R09451 { Hexadecanoic acid }

rn:R01348 <-- [Oxidized NADPH---hemoprotein reductase] --> rn:R08551 -- [Reduced NADPH---hemoprotein reductase] --> rn:R09452 { (9Z)-Octadecenoic acid }

rn:R01348 <-- [Oxidized NADPH---hemoprotein reductase] --> rn:R08551 -- [Reduced NADPH---hemoprotein reductase] --> rn:R09922 { (+)-Linalool }

rn:R01348 <-- [Oxidized NADPH---hemoprotein reductase] --> rn:R08551 -- [Reduced NADPH---hemoprotein reductase] --> rn:R09923 { (-)-Linalool }

rn:R01348 <-- [Oxidized NADPH---hemoprotein reductase] --> rn:R08551 -- [Reduced NADPH---hemoprotein reductase] --> rn:R09925 { Linalool }

rn:R01348 <-- [Oxidized NADPH---hemoprotein reductase] --> rn:R08551 -- [Reduced NADPH---hemoprotein reductase] --> rn:R09934 { Humulene }

rn:R01348 <-- [Oxidized NADPH---hemoprotein reductase] --> rn:R08551 -- [Reduced NADPH---hemoprotein reductase] --> rn:R10562 { (E,E)-Geranyllinalool }

rn:R01348 <-- [Oxidized NADPH---hemoprotein reductase] --> rn:R08551 -- [Reduced NADPH---hemoprotein reductase] --> rn:R11055 { Myrcene }

rn:R01370 <-- Phenylpyruvate --> rn:R00695 -- L-Phenylalanine --> rn:R11068 { 3-[(1R,2S,5R,6S)-5-Hydroxy-7-oxabicyclo[4.1.0]heptan-2-yl]-2-oxopropanoate }

rn:R01370 <-- Phenylpyruvate --> rn:R01372 -- 2-Hydroxyphenylacetate --> rn:R05001 { 2-Hydroxyphenylacetate }

rn:R01370 <-- Phenylpyruvate --> rn:R01372 -- 2-Hydroxyphenylacetate --> rn:R05450 { 2-Hydroxyphenylacetate }

rn:R01370 <-- Phenylpyruvate --> rn:R01375 -- L-Phenylalanine --> rn:R11068 { 3-[(1R,2S,5R,6S)-5-Hydroxy-7-oxabicyclo[4.1.0]heptan-2-yl]-2-oxopropanoate }

rn:R01370 <-- Phenylpyruvate --> rn:R01376 -- L-Phenylalanine --> rn:R11068 { 3-[(1R,2S,5R,6S)-5-Hydroxy-7-oxabicyclo[4.1.0]heptan-2-yl]-2-oxopropanoate }

rn:R01371 <-- Phenylpyruvate --> rn:R00695 -- L-Phenylalanine --> rn:R11068 { 3-[(1R,2S,5R,6S)-5-Hydroxy-7-oxabicyclo[4.1.0]heptan-2-yl]-2-oxopropanoate }

rn:R01371 <-- Phenylpyruvate --> rn:R01372 -- 2-Hydroxyphenylacetate --> rn:R05001 { 2-Hydroxyphenylacetate }

rn:R01371 <-- Phenylpyruvate --> rn:R01372 -- 2-Hydroxyphenylacetate --> rn:R05450 { 2-Hydroxyphenylacetate }

rn:R01371 <-- Phenylpyruvate --> rn:R01375 -- L-Phenylalanine --> rn:R11068 { 3-[(1R,2S,5R,6S)-5-Hydroxy-7-oxabicyclo[4.1.0]heptan-2-yl]-2-oxopropanoate }

rn:R01371 <-- Phenylpyruvate --> rn:R01376 -- L-Phenylalanine --> rn:R11068 { 3-[(1R,2S,5R,6S)-5-Hydroxy-7-oxabicyclo[4.1.0]heptan-2-yl]-2-oxopropanoate }

rn:R01374 <-- Phenylpyruvate --> rn:R00695 -- L-Phenylalanine --> rn:R11068 { 3-[(1R,2S,5R,6S)-5-Hydroxy-7-oxabicyclo[4.1.0]heptan-2-yl]-2-oxopropanoate }

rn:R01374 <-- Phenylpyruvate --> rn:R01372 -- 2-Hydroxyphenylacetate --> rn:R05001 { 2-Hydroxyphenylacetate }

rn:R01374 <-- Phenylpyruvate --> rn:R01372 -- 2-Hydroxyphenylacetate --> rn:R05450 { 2-Hydroxyphenylacetate }

rn:R01374 <-- Phenylpyruvate --> rn:R01375 -- L-Phenylalanine --> rn:R11068 { 3-[(1R,2S,5R,6S)-5-Hydroxy-7-oxabicyclo[4.1.0]heptan-2-yl]-2-oxopropanoate }

rn:R01374 <-- Phenylpyruvate --> rn:R01376 -- L-Phenylalanine --> rn:R11068 { 3-[(1R,2S,5R,6S)-5-Hydroxy-7-oxabicyclo[4.1.0]heptan-2-yl]-2-oxopropanoate }

rn:R01375 <-- L-Phenylalanine --> rn:R00689 -- Hydrogen peroxide --> rn:R01703 { Hexadecanoic acid }

rn:R01375 <-- L-Phenylalanine --> rn:R11918 -- Hydrogen peroxide --> rn:R01703 { Hexadecanoic acid }

rn:R01376 <-- L-Phenylalanine --> rn:R00689 -- Hydrogen peroxide --> rn:R01703 { Hexadecanoic acid }

rn:R01376 <-- L-Phenylalanine --> rn:R11918 -- Hydrogen peroxide --> rn:R01703 { Hexadecanoic acid }

rn:R01422 <-- Benzoyl-CoA --> rn:R10961 -- Acceptor --> rn:R02234 { Cyclohexanone }

rn:R01422 <-- Benzoyl-CoA --> rn:R10961 -- Acceptor --> rn:R03212 { 3-Hydroxycyclohexanone }

rn:R01424 <-- Glycine --> rn:R08701 -- Acceptor --> rn:R02234 { Cyclohexanone }

rn:R01424 <-- Glycine --> rn:R08701 -- Acceptor --> rn:R03212 { 3-Hydroxycyclohexanone }

rn:R01426 <-- trans-Cinnamate --> rn:R11070 -- Styrene --> rn:R05417 { Styrene }

rn:R01426 <-- trans-Cinnamate --> rn:R11070 -- Styrene --> rn:R05488 { Styrene }

rn:R01427 <-- 3-Hydroxybenzoate --> rn:R01508 -- Acceptor --> rn:R02234 { Cyclohexanone }

rn:R01427 <-- 3-Hydroxybenzoate --> rn:R01508 -- Acceptor --> rn:R03212 { 3-Hydroxycyclohexanone }

rn:R01582 <-- Phenylpyruvate --> rn:R00695 -- L-Phenylalanine --> rn:R11068 { 3-[(1R,2S,5R,6S)-5-Hydroxy-7-oxabicyclo[4.1.0]heptan-2-yl]-2-oxopropanoate }

rn:R01582 <-- Phenylpyruvate --> rn:R01372 -- 2-Hydroxyphenylacetate --> rn:R05001 { 2-Hydroxyphenylacetate }

rn:R01582 <-- Phenylpyruvate --> rn:R01372 -- 2-Hydroxyphenylacetate --> rn:R05450 { 2-Hydroxyphenylacetate }

rn:R01582 <-- Phenylpyruvate --> rn:R01375 -- L-Phenylalanine --> rn:R11068 { 3-[(1R,2S,5R,6S)-5-Hydroxy-7-oxabicyclo[4.1.0]heptan-2-yl]-2-oxopropanoate }

rn:R01582 <-- Phenylpyruvate --> rn:R01376 -- L-Phenylalanine --> rn:R11068 { 3-[(1R,2S,5R,6S)-5-Hydroxy-7-oxabicyclo[4.1.0]heptan-2-yl]-2-oxopropanoate }

rn:R01584 <-- Pyruvate --> rn:R00210 -- Acetyl-CoA --> rn:R08530 { (-)-Menthol }

rn:R01584 <-- Pyruvate --> rn:R00210 -- Acetyl-CoA --> rn:R08531 { (+)-Neomenthol }

rn:R01584 <-- Pyruvate --> rn:R00210 -- Acetyl-CoA --> rn:R08532 { (+)-Borneol }

rn:R01584 <-- Pyruvate --> rn:R00210 -- Acetyl-CoA --> rn:R10474 { Cinnamyl alcohol }

rn:R01615 <-- p-Coumaroyl-CoA --> rn:R08767 -- Acetyl-CoA --> rn:R08530 { (-)-Menthol }

rn:R01615 <-- p-Coumaroyl-CoA --> rn:R08767 -- Acetyl-CoA --> rn:R08531 { (+)-Neomenthol }

rn:R01615 <-- p-Coumaroyl-CoA --> rn:R08767 -- Acetyl-CoA --> rn:R08532 { (+)-Borneol }

rn:R01615 <-- p-Coumaroyl-CoA --> rn:R08767 -- Acetyl-CoA --> rn:R10474 { Cinnamyl alcohol }

rn:R01649 <-- Acetate --> rn:R01241 -- Phenyl acetate --> rn:R07342 { Phenyl acetate }

rn:R01651 <-- 3-Methylbutanoyl-CoA --> rn:R04095 -- FADH2 --> rn:R05488 { Styrene }

rn:R01708 <-- Pyridoxal --> rn:R01709 -- Hydrogen peroxide --> rn:R01703 { Hexadecanoic acid }

rn:R01710 <-- Pyridoxal --> rn:R01709 -- Hydrogen peroxide --> rn:R01703 { Hexadecanoic acid }

rn:R01711 <-- Pyridoxal --> rn:R01709 -- Hydrogen peroxide --> rn:R01703 { Hexadecanoic acid }

rn:R01712 <-- Pyridoxal --> rn:R01709 -- Hydrogen peroxide --> rn:R01703 { Hexadecanoic acid }

rn:R01712 <-- L-Alanine --> rn:R04187 -- Pyruvate --> rn:R05136 { Salicylaldehyde }

rn:R01713 <-- L-Aspartate --> rn:R00695 -- L-Phenylalanine --> rn:R11068 { 3-[(1R,2S,5R,6S)-5-Hydroxy-7-oxabicyclo[4.1.0]heptan-2-yl]-2-oxopropanoate }

rn:R01713 <-- Pyridoxal --> rn:R01709 -- Hydrogen peroxide --> rn:R01703 { Hexadecanoic acid }

rn:R01714 <-- Chorismate --> rn:R00986 -- Pyruvate --> rn:R05136 { Salicylaldehyde }

rn:R01714 <-- Chorismate --> rn:R06603 -- Pyruvate --> rn:R05136 { Salicylaldehyde }

rn:R01714 <-- Chorismate --> rn:R10583 -- Pyruvate --> rn:R05136 { Salicylaldehyde }

rn:R01717 <-- Isochorismate --> rn:R03037 -- Pyruvate --> rn:R05136 { Salicylaldehyde }

rn:R01717 <-- Isochorismate --> rn:R06602 -- Pyruvate --> rn:R05136 { Salicylaldehyde }

rn:R01731 <-- L-Aspartate --> rn:R00695 -- L-Phenylalanine --> rn:R11068 { 3-[(1R,2S,5R,6S)-5-Hydroxy-7-oxabicyclo[4.1.0]heptan-2-yl]-2-oxopropanoate }

rn:R01815 <-- 3,4-Dihydroxy-L-phenylalanine --> rn:R02080 -- Dopamine --> rn:R08447 { 4-Hydroxydihydrocinnamaldehyde }

rn:R01815 <-- 3,4-Dihydroxy-L-phenylalanine --> rn:R11916 -- Hydrogen peroxide --> rn:R01703 { Hexadecanoic acid }

rn:R01816 <-- 3-Hydroxyanthranilate --> rn:R02666 -- Hydrogen peroxide --> rn:R01703 { Hexadecanoic acid }

rn:R01816 <-- 3-Hydroxyanthranilate --> rn:R02670 -- Hydrogen peroxide --> rn:R01703 { Hexadecanoic acid }

rn:R01909 <-- Pyridoxine phosphate --> rn:R00278 -- Hydrogen peroxide --> rn:R01703 { Hexadecanoic acid }

rn:R01911 <-- Pyridoxine phosphate --> rn:R00278 -- Hydrogen peroxide --> rn:R01703 { Hexadecanoic acid }

rn:R01918 <-- Glutathione --> rn:R00120 -- Hydrogen peroxide --> rn:R01703 { Hexadecanoic acid }

rn:R01918 <-- Spermidine --> rn:R09077 -- Hydrogen peroxide --> rn:R01703 { Hexadecanoic acid }

rn:R01933 <-- Glutaryl-CoA --> rn:R02487 -- FADH2 --> rn:R05488 { Styrene }

rn:R02050 <-- L-Alanine --> rn:R04187 -- Pyruvate --> rn:R05136 { Salicylaldehyde }

rn:R02077 <-- L-Glutamate --> rn:R00114 -- L-Glutamine --> rn:R02781 { 2,4,6/3,5-Pentahydroxycyclohexanone }

rn:R02078 <-- 3,4-Dihydroxy-L-phenylalanine --> rn:R02080 -- Dopamine --> rn:R08447 { 4-Hydroxydihydrocinnamaldehyde }

rn:R02078 <-- 3,4-Dihydroxy-L-phenylalanine --> rn:R11916 -- Hydrogen peroxide --> rn:R01703 { Hexadecanoic acid }

rn:R02080 <-- Dopamine --> rn:R04300 -- Hydrogen peroxide --> rn:R01703 { Hexadecanoic acid }

rn:R02155 <-- N-Methylhistamine --> rn:R04674 -- Hydrogen peroxide --> rn:R01703 { Hexadecanoic acid }

rn:R02222 <-- Oleoyl-CoA --> rn:R08176 -- (9Z)-Octadecenoic acid --> rn:R09452 { (9Z)-Octadecenoic acid }

rn:R02222 <-- Oleoyl-CoA --> rn:R08176 -- (9Z)-Octadecenoic acid --> rn:R09462 { (9Z)-Octadecenoic acid }

rn:R02222 <-- Oleoyl-CoA --> rn:R08176 -- (9Z)-Octadecenoic acid --> rn:R10576 { (9Z)-Octadecenoic acid }

rn:R02252 <-- trans-Cinnamate --> rn:R11070 -- Styrene --> rn:R05417 { Styrene }

rn:R02252 <-- trans-Cinnamate --> rn:R11070 -- Styrene --> rn:R05488 { Styrene }

rn:R02253 <-- [Oxidized NADPH---hemoprotein reductase] --> rn:R08551 -- [Reduced NADPH---hemoprotein reductase] --> rn:R02468 { (-)-Limonene }

rn:R02253 <-- [Oxidized NADPH---hemoprotein reductase] --> rn:R08551 -- [Reduced NADPH---hemoprotein reductase] --> rn:R02469 { (-)-Limonene }

rn:R02253 <-- [Oxidized NADPH---hemoprotein reductase] --> rn:R08551 -- [Reduced NADPH---hemoprotein reductase] --> rn:R02470 { (-)-Limonene }

rn:R02253 <-- [Oxidized NADPH---hemoprotein reductase] --> rn:R08551 -- [Reduced NADPH---hemoprotein reductase] --> rn:R04366 { Linalool }

rn:R02253 <-- [Oxidized NADPH---hemoprotein reductase] --> rn:R08551 -- [Reduced NADPH---hemoprotein reductase] --> rn:R06119 { d-Limonene }

rn:R02253 <-- [Oxidized NADPH---hemoprotein reductase] --> rn:R08551 -- [Reduced NADPH---hemoprotein reductase] --> rn:R09451 { Hexadecanoic acid }

rn:R02253 <-- [Oxidized NADPH---hemoprotein reductase] --> rn:R08551 -- [Reduced NADPH---hemoprotein reductase] --> rn:R09452 { (9Z)-Octadecenoic acid }

rn:R02253 <-- [Oxidized NADPH---hemoprotein reductase] --> rn:R08551 -- [Reduced NADPH---hemoprotein reductase] --> rn:R09922 { (+)-Linalool }

rn:R02253 <-- [Oxidized NADPH---hemoprotein reductase] --> rn:R08551 -- [Reduced NADPH---hemoprotein reductase] --> rn:R09923 { (-)-Linalool }

rn:R02253 <-- [Oxidized NADPH---hemoprotein reductase] --> rn:R08551 -- [Reduced NADPH---hemoprotein reductase] --> rn:R09925 { Linalool }

rn:R02253 <-- [Oxidized NADPH---hemoprotein reductase] --> rn:R08551 -- [Reduced NADPH---hemoprotein reductase] --> rn:R09934 { Humulene }

rn:R02253 <-- [Oxidized NADPH---hemoprotein reductase] --> rn:R08551 -- [Reduced NADPH---hemoprotein reductase] --> rn:R10562 { (E,E)-Geranyllinalool }

rn:R02253 <-- [Oxidized NADPH---hemoprotein reductase] --> rn:R08551 -- [Reduced NADPH---hemoprotein reductase] --> rn:R11055 { Myrcene }

rn:R02260 <-- Methylglyoxal --> rn:R00205 -- Pyruvate --> rn:R05136 { Salicylaldehyde }

rn:R02271 <-- L-Alanine --> rn:R04187 -- Pyruvate --> rn:R05136 { Salicylaldehyde }

rn:R02285 <-- L-Glutamate --> rn:R00114 -- L-Glutamine --> rn:R02781 { 2,4,6/3,5-Pentahydroxycyclohexanone }

rn:R02315 <-- L-Glutamate --> rn:R00114 -- L-Glutamine --> rn:R02781 { 2,4,6/3,5-Pentahydroxycyclohexanone }

rn:R02383 <-- Dopamine --> rn:R04300 -- Hydrogen peroxide --> rn:R01703 { Hexadecanoic acid }

rn:R02389 <-- Bilirubin beta-diglucuronide --> rn:R04979 -- Acceptor --> rn:R02234 { Cyclohexanone }

rn:R02389 <-- Bilirubin beta-diglucuronide --> rn:R04979 -- Acceptor --> rn:R03212 { 3-Hydroxycyclohexanone }

rn:R02402 <-- Glutaryl-CoA --> rn:R02487 -- FADH2 --> rn:R05488 { Styrene }

rn:R02450 <-- Benzoyl-CoA --> rn:R10961 -- Acceptor --> rn:R02234 { Cyclohexanone }

rn:R02450 <-- Benzoyl-CoA --> rn:R10961 -- Acceptor --> rn:R03212 { 3-Hydroxycyclohexanone }

rn:R02487 <-- FADH2 --> rn:R03978 -- FAD --> rn:R05537 { 3-(2-Hydroxyphenyl)propanoate }

rn:R02487 <-- FADH2 --> rn:R05488 -- FAD --> rn:R05537 { 3-(2-Hydroxyphenyl)propanoate }

rn:R02487 <-- FADH2 --> rn:R09517 -- FAD --> rn:R05537 { 3-(2-Hydroxyphenyl)propanoate }

rn:R02487 <-- FADH2 --> rn:R11653 -- FAD --> rn:R05537 { 3-(2-Hydroxyphenyl)propanoate }

rn:R02487 <-- FADH2 --> rn:R12021 -- FAD --> rn:R05537 { 3-(2-Hydroxyphenyl)propanoate }

rn:R02487 <-- FADH2 --> rn:R12023 -- FAD --> rn:R05537 { 3-(2-Hydroxyphenyl)propanoate }

rn:R02487 <-- FADH2 --> rn:R12027 -- FAD --> rn:R05537 { 3-(2-Hydroxyphenyl)propanoate }

rn:R02487 <-- FADH2 --> rn:R12030 -- FAD --> rn:R05537 { 3-(2-Hydroxyphenyl)propanoate }

rn:R02493 <-- Pyridoxamine phosphate --> rn:R00277 -- Hydrogen peroxide --> rn:R01703 { Hexadecanoic acid }

rn:R02494 <-- Pyridoxamine phosphate --> rn:R00277 -- Hydrogen peroxide --> rn:R01703 { Hexadecanoic acid }

rn:R02534 <-- L-Normetanephrine --> rn:R04893 -- Hydrogen peroxide --> rn:R01703 { Hexadecanoic acid }

rn:R02535 <-- L-Noradrenaline --> rn:R02532 -- Hydrogen peroxide --> rn:R01703 { Hexadecanoic acid }

rn:R02536 <-- Phenylacetic acid --> rn:R05487 -- 2-Hydroxyphenylacetate --> rn:R05001 { 2-Hydroxyphenylacetate }

rn:R02536 <-- Phenylacetic acid --> rn:R05487 -- 2-Hydroxyphenylacetate --> rn:R05450 { 2-Hydroxyphenylacetate }

rn:R02537 <-- Phenylacetic acid --> rn:R05487 -- 2-Hydroxyphenylacetate --> rn:R05001 { 2-Hydroxyphenylacetate }

rn:R02537 <-- Phenylacetic acid --> rn:R05487 -- 2-Hydroxyphenylacetate --> rn:R05450 { 2-Hydroxyphenylacetate }

rn:R02540 <-- Phenylacetic acid --> rn:R05487 -- 2-Hydroxyphenylacetate --> rn:R05001 { 2-Hydroxyphenylacetate }

rn:R02540 <-- Phenylacetic acid --> rn:R05487 -- 2-Hydroxyphenylacetate --> rn:R05450 { 2-Hydroxyphenylacetate }

rn:R02558 <-- Mandelonitrile --> rn:R01767 -- Benzaldehyde --> rn:R01419 { Benzaldehyde }

rn:R02558 <-- Mandelonitrile --> rn:R01767 -- Benzaldehyde --> rn:R01420 { Benzaldehyde }

rn:R02558 <-- Mandelonitrile --> rn:R11380 -- Hydrogen peroxide --> rn:R01703 { Hexadecanoic acid }

rn:R02605 <-- Cl- --> rn:R12021 -- FAD --> rn:R05537 { 3-(2-Hydroxyphenyl)propanoate }

rn:R02605 <-- Cl- --> rn:R12023 -- FAD --> rn:R05537 { 3-(2-Hydroxyphenyl)propanoate }

rn:R02656 <-- Maleylpyruvate --> rn:R11257 -- Pyruvate --> rn:R05136 { Salicylaldehyde }

rn:R02680 <-- Indole-3-acetaldehyde --> rn:R02681 -- Hydrogen peroxide --> rn:R01703 { Hexadecanoic acid }

rn:R02708 <-- [Oxidized NADPH---hemoprotein reductase] --> rn:R08551 -- [Reduced NADPH---hemoprotein reductase] --> rn:R02468 { (-)-Limonene }

rn:R02708 <-- [Oxidized NADPH---hemoprotein reductase] --> rn:R08551 -- [Reduced NADPH---hemoprotein reductase] --> rn:R02469 { (-)-Limonene }

rn:R02708 <-- [Oxidized NADPH---hemoprotein reductase] --> rn:R08551 -- [Reduced NADPH---hemoprotein reductase] --> rn:R02470 { (-)-Limonene }

rn:R02708 <-- [Oxidized NADPH---hemoprotein reductase] --> rn:R08551 -- [Reduced NADPH---hemoprotein reductase] --> rn:R04366 { Linalool }

rn:R02708 <-- [Oxidized NADPH---hemoprotein reductase] --> rn:R08551 -- [Reduced NADPH---hemoprotein reductase] --> rn:R06119 { d-Limonene }

rn:R02708 <-- [Oxidized NADPH---hemoprotein reductase] --> rn:R08551 -- [Reduced NADPH---hemoprotein reductase] --> rn:R09451 { Hexadecanoic acid }

rn:R02708 <-- [Oxidized NADPH---hemoprotein reductase] --> rn:R08551 -- [Reduced NADPH---hemoprotein reductase] --> rn:R09452 { (9Z)-Octadecenoic acid }

rn:R02708 <-- [Oxidized NADPH---hemoprotein reductase] --> rn:R08551 -- [Reduced NADPH---hemoprotein reductase] --> rn:R09922 { (+)-Linalool }

rn:R02708 <-- [Oxidized NADPH---hemoprotein reductase] --> rn:R08551 -- [Reduced NADPH---hemoprotein reductase] --> rn:R09923 { (-)-Linalool }

rn:R02708 <-- [Oxidized NADPH---hemoprotein reductase] --> rn:R08551 -- [Reduced NADPH---hemoprotein reductase] --> rn:R09925 { Linalool }

rn:R02708 <-- [Oxidized NADPH---hemoprotein reductase] --> rn:R08551 -- [Reduced NADPH---hemoprotein reductase] --> rn:R09934 { Humulene }

rn:R02708 <-- [Oxidized NADPH---hemoprotein reductase] --> rn:R08551 -- [Reduced NADPH---hemoprotein reductase] --> rn:R10562 { (E,E)-Geranyllinalool }

rn:R02708 <-- [Oxidized NADPH---hemoprotein reductase] --> rn:R08551 -- [Reduced NADPH---hemoprotein reductase] --> rn:R11055 { Myrcene }

rn:R02772 <-- L-Glutamate --> rn:R00114 -- L-Glutamine --> rn:R02781 { 2,4,6/3,5-Pentahydroxycyclohexanone }

rn:R02773 <-- L-Glutamate --> rn:R00114 -- L-Glutamine --> rn:R02781 { 2,4,6/3,5-Pentahydroxycyclohexanone }

rn:R02920 <-- L-Metanephrine --> rn:R04894 -- Hydrogen peroxide --> rn:R01703 { Hexadecanoic acid }

rn:R02942 <-- Acetate --> rn:R01241 -- Phenyl acetate --> rn:R07342 { Phenyl acetate }

rn:R02955 <-- (3S)-Citramalyl-CoA --> rn:R00237 -- Pyruvate --> rn:R05136 { Salicylaldehyde }

rn:R02955 <-- (3S)-Citramalyl-CoA --> rn:R00237 -- Acetyl-CoA --> rn:R08530 { (-)-Menthol }

rn:R02955 <-- (3S)-Citramalyl-CoA --> rn:R00237 -- Acetyl-CoA --> rn:R08531 { (+)-Neomenthol }

rn:R02955 <-- (3S)-Citramalyl-CoA --> rn:R00237 -- Acetyl-CoA --> rn:R08532 { (+)-Borneol }

rn:R02955 <-- (3S)-Citramalyl-CoA --> rn:R00237 -- Acetyl-CoA --> rn:R10474 { Cinnamyl alcohol }

rn:R02955 <-- Acetate --> rn:R01241 -- Phenyl acetate --> rn:R07342 { Phenyl acetate }

rn:R02979 <-- FADH2 --> rn:R03978 -- FAD --> rn:R05537 { 3-(2-Hydroxyphenyl)propanoate }

rn:R02979 <-- FADH2 --> rn:R05488 -- FAD --> rn:R05537 { 3-(2-Hydroxyphenyl)propanoate }

rn:R02979 <-- FADH2 --> rn:R09517 -- FAD --> rn:R05537 { 3-(2-Hydroxyphenyl)propanoate }

rn:R02979 <-- FADH2 --> rn:R11653 -- FAD --> rn:R05537 { 3-(2-Hydroxyphenyl)propanoate }

rn:R02979 <-- FADH2 --> rn:R12021 -- FAD --> rn:R05537 { 3-(2-Hydroxyphenyl)propanoate }

rn:R02979 <-- FADH2 --> rn:R12023 -- FAD --> rn:R05537 { 3-(2-Hydroxyphenyl)propanoate }

rn:R02979 <-- FADH2 --> rn:R12027 -- FAD --> rn:R05537 { 3-(2-Hydroxyphenyl)propanoate }

rn:R02979 <-- FADH2 --> rn:R12030 -- FAD --> rn:R05537 { 3-(2-Hydroxyphenyl)propanoate }

rn:R02996 <-- Phenol --> rn:R01241 -- Phenyl acetate --> rn:R07342 { Phenyl acetate }

rn:R02996 <-- Phenol --> rn:R11653 -- FAD --> rn:R05537 { 3-(2-Hydroxyphenyl)propanoate }

rn:R03037 <-- Pyruvate --> rn:R00210 -- Acetyl-CoA --> rn:R08530 { (-)-Menthol }

rn:R03037 <-- Pyruvate --> rn:R00210 -- Acetyl-CoA --> rn:R08531 { (+)-Neomenthol }

rn:R03037 <-- Pyruvate --> rn:R00210 -- Acetyl-CoA --> rn:R08532 { (+)-Borneol }

rn:R03037 <-- Pyruvate --> rn:R00210 -- Acetyl-CoA --> rn:R10474 { Cinnamyl alcohol }

rn:R03153 <-- (3S)-Citramalyl-CoA --> rn:R00237 -- Pyruvate --> rn:R05136 { Salicylaldehyde }

rn:R03153 <-- (3S)-Citramalyl-CoA --> rn:R00237 -- Acetyl-CoA --> rn:R08530 { (-)-Menthol }

rn:R03153 <-- (3S)-Citramalyl-CoA --> rn:R00237 -- Acetyl-CoA --> rn:R08531 { (+)-Neomenthol }

rn:R03153 <-- (3S)-Citramalyl-CoA --> rn:R00237 -- Acetyl-CoA --> rn:R08532 { (+)-Borneol }

rn:R03153 <-- (3S)-Citramalyl-CoA --> rn:R00237 -- Acetyl-CoA --> rn:R10474 { Cinnamyl alcohol }

rn:R03153 <-- Acetate --> rn:R01241 -- Phenyl acetate --> rn:R07342 { Phenyl acetate }

rn:R03154 <-- (3S)-Citramalyl-CoA --> rn:R00237 -- Pyruvate --> rn:R05136 { Salicylaldehyde }

rn:R03154 <-- (3S)-Citramalyl-CoA --> rn:R00237 -- Acetyl-CoA --> rn:R08530 { (-)-Menthol }

rn:R03154 <-- (3S)-Citramalyl-CoA --> rn:R00237 -- Acetyl-CoA --> rn:R08531 { (+)-Neomenthol }

rn:R03154 <-- (3S)-Citramalyl-CoA --> rn:R00237 -- Acetyl-CoA --> rn:R08532 { (+)-Borneol }

rn:R03154 <-- (3S)-Citramalyl-CoA --> rn:R00237 -- Acetyl-CoA --> rn:R10474 { Cinnamyl alcohol }

rn:R03166 <-- Uroporphyrinogen I --> rn:R04971 -- Acceptor --> rn:R02234 { Cyclohexanone }

rn:R03166 <-- Uroporphyrinogen I --> rn:R04971 -- Acceptor --> rn:R03212 { 3-Hydroxycyclohexanone }

rn:R03197 <-- Coproporphyrinogen III --> rn:R04178 -- Hydrogen peroxide --> rn:R01703 { Hexadecanoic acid }

rn:R03207 <-- L-Glutamate --> rn:R00114 -- L-Glutamine --> rn:R02781 { 2,4,6/3,5-Pentahydroxycyclohexanone }

rn:R03208 <-- Thyroxine --> rn:R03734 -- Acceptor --> rn:R02234 { Cyclohexanone }

rn:R03208 <-- Thyroxine --> rn:R03734 -- Acceptor --> rn:R03212 { 3-Hydroxycyclohexanone }

rn:R03220 <-- Protoporphyrinogen IX --> rn:R03222 -- Hydrogen peroxide --> rn:R01703 { Hexadecanoic acid }

rn:R03243 <-- L-Glutamate --> rn:R00114 -- L-Glutamine --> rn:R02781 { 2,4,6/3,5-Pentahydroxycyclohexanone }

rn:R03502 <-- L-Alanine --> rn:R04187 -- Pyruvate --> rn:R05136 { Salicylaldehyde }

rn:R03539 <-- L-Tyrosine --> rn:R10246 -- Acceptor --> rn:R02234 { Cyclohexanone }

rn:R03539 <-- L-Tyrosine --> rn:R10246 -- Acceptor --> rn:R03212 { 3-Hydroxycyclohexanone }

rn:R03539 <-- L-Tyrosine --> rn:R11917 -- Hydrogen peroxide --> rn:R01703 { Hexadecanoic acid }

rn:R03692 <-- 3-(2-Hydroxyphenyl)propanoate --> rn:R05537 -- FADH2 --> rn:R05488 { Styrene }

rn:R03692 <-- 3-(2-Hydroxyphenyl)propanoate --> rn:R05537 -- Acetyl-CoA --> rn:R08530 { (-)-Menthol }

rn:R03692 <-- 3-(2-Hydroxyphenyl)propanoate --> rn:R05537 -- Acetyl-CoA --> rn:R08531 { (+)-Neomenthol }

rn:R03692 <-- 3-(2-Hydroxyphenyl)propanoate --> rn:R05537 -- Acetyl-CoA --> rn:R08532 { (+)-Borneol }

rn:R03692 <-- 3-(2-Hydroxyphenyl)propanoate --> rn:R05537 -- Acetyl-CoA --> rn:R10474 { Cinnamyl alcohol }

rn:R03777 <-- FADH2 --> rn:R03978 -- FAD --> rn:R05537 { 3-(2-Hydroxyphenyl)propanoate }

rn:R03777 <-- FADH2 --> rn:R05488 -- FAD --> rn:R05537 { 3-(2-Hydroxyphenyl)propanoate }

rn:R03777 <-- FADH2 --> rn:R09517 -- FAD --> rn:R05537 { 3-(2-Hydroxyphenyl)propanoate }

rn:R03777 <-- FADH2 --> rn:R11653 -- FAD --> rn:R05537 { 3-(2-Hydroxyphenyl)propanoate }

rn:R03777 <-- FADH2 --> rn:R12021 -- FAD --> rn:R05537 { 3-(2-Hydroxyphenyl)propanoate }

rn:R03777 <-- FADH2 --> rn:R12023 -- FAD --> rn:R05537 { 3-(2-Hydroxyphenyl)propanoate }

rn:R03777 <-- FADH2 --> rn:R12027 -- FAD --> rn:R05537 { 3-(2-Hydroxyphenyl)propanoate }

rn:R03777 <-- FADH2 --> rn:R12030 -- FAD --> rn:R05537 { 3-(2-Hydroxyphenyl)propanoate }

rn:R03790 <-- alpha-Oxo-benzeneacetic acid --> rn:R01764 -- Benzaldehyde --> rn:R01419 { Benzaldehyde }

rn:R03790 <-- alpha-Oxo-benzeneacetic acid --> rn:R01764 -- Benzaldehyde --> rn:R01420 { Benzaldehyde }

rn:R03792 <-- alpha-Oxo-benzeneacetic acid --> rn:R01764 -- Benzaldehyde --> rn:R01419 { Benzaldehyde }

rn:R03792 <-- alpha-Oxo-benzeneacetic acid --> rn:R01764 -- Benzaldehyde --> rn:R01420 { Benzaldehyde }

rn:R03793 <-- alpha-Oxo-benzeneacetic acid --> rn:R01764 -- Benzaldehyde --> rn:R01419 { Benzaldehyde }

rn:R03793 <-- alpha-Oxo-benzeneacetic acid --> rn:R01764 -- Benzaldehyde --> rn:R01420 { Benzaldehyde }

rn:R03794 <-- (S)-4-Hydroxymandelate --> rn:R02673 -- Hydrogen peroxide --> rn:R01703 { Hexadecanoic acid }

rn:R03794 <-- (S)-4-Hydroxymandelate --> rn:R06633 -- Hydrogen peroxide --> rn:R01703 { Hexadecanoic acid }

rn:R03857 <-- FADH2 --> rn:R03978 -- FAD --> rn:R05537 { 3-(2-Hydroxyphenyl)propanoate }

rn:R03857 <-- FADH2 --> rn:R05488 -- FAD --> rn:R05537 { 3-(2-Hydroxyphenyl)propanoate }

rn:R03857 <-- FADH2 --> rn:R09517 -- FAD --> rn:R05537 { 3-(2-Hydroxyphenyl)propanoate }

rn:R03857 <-- FADH2 --> rn:R11653 -- FAD --> rn:R05537 { 3-(2-Hydroxyphenyl)propanoate }

rn:R03857 <-- FADH2 --> rn:R12021 -- FAD --> rn:R05537 { 3-(2-Hydroxyphenyl)propanoate }

rn:R03857 <-- FADH2 --> rn:R12023 -- FAD --> rn:R05537 { 3-(2-Hydroxyphenyl)propanoate }

rn:R03857 <-- FADH2 --> rn:R12027 -- FAD --> rn:R05537 { 3-(2-Hydroxyphenyl)propanoate }

rn:R03857 <-- FADH2 --> rn:R12030 -- FAD --> rn:R05537 { 3-(2-Hydroxyphenyl)propanoate }

rn:R03868 <-- 3-Fumarylpyruvate --> rn:R01085 -- Pyruvate --> rn:R05136 { Salicylaldehyde }

rn:R03915 <-- Glutathione --> rn:R00120 -- Hydrogen peroxide --> rn:R01703 { Hexadecanoic acid }

rn:R03916 <-- L-Glutamate --> rn:R00114 -- L-Glutamine --> rn:R02781 { 2,4,6/3,5-Pentahydroxycyclohexanone }

rn:R03952 <-- L-Glutamate --> rn:R00114 -- L-Glutamine --> rn:R02781 { 2,4,6/3,5-Pentahydroxycyclohexanone }

rn:R03975 <-- Chenodeoxycholate --> rn:R03978 -- FAD --> rn:R05537 { 3-(2-Hydroxyphenyl)propanoate }

rn:R03975 <-- Glycine --> rn:R08701 -- Acceptor --> rn:R02234 { Cyclohexanone }

rn:R03975 <-- Glycine --> rn:R08701 -- Acceptor --> rn:R03212 { 3-Hydroxycyclohexanone }

rn:R03977 <-- Chenodeoxycholate --> rn:R03978 -- FAD --> rn:R05537 { 3-(2-Hydroxyphenyl)propanoate }

rn:R03978 <-- FAD --> rn:R02487 -- FADH2 --> rn:R05488 { Styrene }

rn:R03978 <-- FAD --> rn:R04095 -- FADH2 --> rn:R05488 { Styrene }

rn:R03978 <-- FAD --> rn:R05537 -- FADH2 --> rn:R05488 { Styrene }

rn:R03978 <-- FAD --> rn:R05537 -- Acetyl-CoA --> rn:R08530 { (-)-Menthol }

rn:R03978 <-- FAD --> rn:R05537 -- Acetyl-CoA --> rn:R08531 { (+)-Neomenthol }

rn:R03978 <-- FAD --> rn:R05537 -- Acetyl-CoA --> rn:R08532 { (+)-Borneol }

rn:R03978 <-- FAD --> rn:R05537 -- Acetyl-CoA --> rn:R10474 { Cinnamyl alcohol }

rn:R03978 <-- FAD --> rn:R06943 -- FADH2 --> rn:R05488 { Styrene }

rn:R03978 <-- FAD --> rn:R07220 -- FADH2 --> rn:R05488 { Styrene }

rn:R03978 <-- FAD --> rn:R09520 -- FADH2 --> rn:R05488 { Styrene }

rn:R03978 <-- FAD --> rn:R11130 -- FADH2 --> rn:R05488 { Styrene }

rn:R03979 <-- Phenol --> rn:R01241 -- Phenyl acetate --> rn:R07342 { Phenyl acetate }

rn:R03979 <-- Phenol --> rn:R11653 -- FAD --> rn:R05537 { 3-(2-Hydroxyphenyl)propanoate }

rn:R03982 <-- Cl- --> rn:R12021 -- FAD --> rn:R05537 { 3-(2-Hydroxyphenyl)propanoate }

rn:R03982 <-- Cl- --> rn:R12023 -- FAD --> rn:R05537 { 3-(2-Hydroxyphenyl)propanoate }

rn:R03984 <-- Glutathione --> rn:R00120 -- Hydrogen peroxide --> rn:R01703 { Hexadecanoic acid }

rn:R03990 <-- FADH2 --> rn:R03978 -- FAD --> rn:R05537 { 3-(2-Hydroxyphenyl)propanoate }

rn:R03990 <-- FADH2 --> rn:R05488 -- FAD --> rn:R05537 { 3-(2-Hydroxyphenyl)propanoate }

rn:R03990 <-- FADH2 --> rn:R09517 -- FAD --> rn:R05537 { 3-(2-Hydroxyphenyl)propanoate }

rn:R03990 <-- FADH2 --> rn:R11653 -- FAD --> rn:R05537 { 3-(2-Hydroxyphenyl)propanoate }

rn:R03990 <-- FADH2 --> rn:R12021 -- FAD --> rn:R05537 { 3-(2-Hydroxyphenyl)propanoate }

rn:R03990 <-- FADH2 --> rn:R12023 -- FAD --> rn:R05537 { 3-(2-Hydroxyphenyl)propanoate }

rn:R03990 <-- FADH2 --> rn:R12027 -- FAD --> rn:R05537 { 3-(2-Hydroxyphenyl)propanoate }

rn:R03990 <-- FADH2 --> rn:R12030 -- FAD --> rn:R05537 { 3-(2-Hydroxyphenyl)propanoate }

rn:R03995 <-- Parapyruvate --> rn:R00008 -- Pyruvate --> rn:R05136 { Salicylaldehyde }

rn:R04039 <-- Glutathione --> rn:R00120 -- Hydrogen peroxide --> rn:R01703 { Hexadecanoic acid }

rn:R04092 <-- FADH2 --> rn:R03978 -- FAD --> rn:R05537 { 3-(2-Hydroxyphenyl)propanoate }

rn:R04092 <-- FADH2 --> rn:R05488 -- FAD --> rn:R05537 { 3-(2-Hydroxyphenyl)propanoate }

rn:R04092 <-- FADH2 --> rn:R09517 -- FAD --> rn:R05537 { 3-(2-Hydroxyphenyl)propanoate }

rn:R04092 <-- FADH2 --> rn:R11653 -- FAD --> rn:R05537 { 3-(2-Hydroxyphenyl)propanoate }

rn:R04092 <-- FADH2 --> rn:R12021 -- FAD --> rn:R05537 { 3-(2-Hydroxyphenyl)propanoate }

rn:R04092 <-- FADH2 --> rn:R12023 -- FAD --> rn:R05537 { 3-(2-Hydroxyphenyl)propanoate }

rn:R04092 <-- FADH2 --> rn:R12027 -- FAD --> rn:R05537 { 3-(2-Hydroxyphenyl)propanoate }

rn:R04092 <-- FADH2 --> rn:R12030 -- FAD --> rn:R05537 { 3-(2-Hydroxyphenyl)propanoate }

rn:R04094 <-- Sulfur --> rn:R03533 -- Acceptor --> rn:R02234 { Cyclohexanone }

rn:R04094 <-- Sulfur --> rn:R03533 -- Acceptor --> rn:R03212 { 3-Hydroxycyclohexanone }

rn:R04095 <-- FADH2 --> rn:R03978 -- FAD --> rn:R05537 { 3-(2-Hydroxyphenyl)propanoate }

rn:R04095 <-- FADH2 --> rn:R05488 -- FAD --> rn:R05537 { 3-(2-Hydroxyphenyl)propanoate }

rn:R04095 <-- FADH2 --> rn:R09517 -- FAD --> rn:R05537 { 3-(2-Hydroxyphenyl)propanoate }

rn:R04095 <-- FADH2 --> rn:R11653 -- FAD --> rn:R05537 { 3-(2-Hydroxyphenyl)propanoate }

rn:R04095 <-- FADH2 --> rn:R12021 -- FAD --> rn:R05537 { 3-(2-Hydroxyphenyl)propanoate }

rn:R04095 <-- FADH2 --> rn:R12023 -- FAD --> rn:R05537 { 3-(2-Hydroxyphenyl)propanoate }

rn:R04095 <-- FADH2 --> rn:R12027 -- FAD --> rn:R05537 { 3-(2-Hydroxyphenyl)propanoate }

rn:R04095 <-- FADH2 --> rn:R12030 -- FAD --> rn:R05537 { 3-(2-Hydroxyphenyl)propanoate }

rn:R04101 <-- Cl- --> rn:R12021 -- FAD --> rn:R05537 { 3-(2-Hydroxyphenyl)propanoate }

rn:R04101 <-- Cl- --> rn:R12023 -- FAD --> rn:R05537 { 3-(2-Hydroxyphenyl)propanoate }

rn:R04103 <-- Phenylacetic acid --> rn:R05487 -- 2-Hydroxyphenylacetate --> rn:R05001 { 2-Hydroxyphenylacetate }

rn:R04103 <-- Phenylacetic acid --> rn:R05487 -- 2-Hydroxyphenylacetate --> rn:R05450 { 2-Hydroxyphenylacetate }

rn:R04121 <-- [Oxidized NADPH---hemoprotein reductase] --> rn:R08551 -- [Reduced NADPH---hemoprotein reductase] --> rn:R02468 { (-)-Limonene }

rn:R04121 <-- [Oxidized NADPH---hemoprotein reductase] --> rn:R08551 -- [Reduced NADPH---hemoprotein reductase] --> rn:R02469 { (-)-Limonene }

rn:R04121 <-- [Oxidized NADPH---hemoprotein reductase] --> rn:R08551 -- [Reduced NADPH---hemoprotein reductase] --> rn:R02470 { (-)-Limonene }

rn:R04121 <-- [Oxidized NADPH---hemoprotein reductase] --> rn:R08551 -- [Reduced NADPH---hemoprotein reductase] --> rn:R04366 { Linalool }

rn:R04121 <-- [Oxidized NADPH---hemoprotein reductase] --> rn:R08551 -- [Reduced NADPH---hemoprotein reductase] --> rn:R06119 { d-Limonene }

rn:R04121 <-- [Oxidized NADPH---hemoprotein reductase] --> rn:R08551 -- [Reduced NADPH---hemoprotein reductase] --> rn:R09451 { Hexadecanoic acid }

rn:R04121 <-- [Oxidized NADPH---hemoprotein reductase] --> rn:R08551 -- [Reduced NADPH---hemoprotein reductase] --> rn:R09452 { (9Z)-Octadecenoic acid }

rn:R04121 <-- [Oxidized NADPH---hemoprotein reductase] --> rn:R08551 -- [Reduced NADPH---hemoprotein reductase] --> rn:R09922 { (+)-Linalool }

rn:R04121 <-- [Oxidized NADPH---hemoprotein reductase] --> rn:R08551 -- [Reduced NADPH---hemoprotein reductase] --> rn:R09923 { (-)-Linalool }

rn:R04121 <-- [Oxidized NADPH---hemoprotein reductase] --> rn:R08551 -- [Reduced NADPH---hemoprotein reductase] --> rn:R09925 { Linalool }

rn:R04121 <-- [Oxidized NADPH---hemoprotein reductase] --> rn:R08551 -- [Reduced NADPH---hemoprotein reductase] --> rn:R09934 { Humulene }

rn:R04121 <-- [Oxidized NADPH---hemoprotein reductase] --> rn:R08551 -- [Reduced NADPH---hemoprotein reductase] --> rn:R10562 { (E,E)-Geranyllinalool }

rn:R04121 <-- [Oxidized NADPH---hemoprotein reductase] --> rn:R08551 -- [Reduced NADPH---hemoprotein reductase] --> rn:R11055 { Myrcene }

rn:R04122 <-- [Oxidized NADPH---hemoprotein reductase] --> rn:R08551 -- [Reduced NADPH---hemoprotein reductase] --> rn:R02468 { (-)-Limonene }

rn:R04122 <-- [Oxidized NADPH---hemoprotein reductase] --> rn:R08551 -- [Reduced NADPH---hemoprotein reductase] --> rn:R02469 { (-)-Limonene }

rn:R04122 <-- [Oxidized NADPH---hemoprotein reductase] --> rn:R08551 -- [Reduced NADPH---hemoprotein reductase] --> rn:R02470 { (-)-Limonene }

rn:R04122 <-- [Oxidized NADPH---hemoprotein reductase] --> rn:R08551 -- [Reduced NADPH---hemoprotein reductase] --> rn:R04366 { Linalool }

rn:R04122 <-- [Oxidized NADPH---hemoprotein reductase] --> rn:R08551 -- [Reduced NADPH---hemoprotein reductase] --> rn:R06119 { d-Limonene }

rn:R04122 <-- [Oxidized NADPH---hemoprotein reductase] --> rn:R08551 -- [Reduced NADPH---hemoprotein reductase] --> rn:R09451 { Hexadecanoic acid }

rn:R04122 <-- [Oxidized NADPH---hemoprotein reductase] --> rn:R08551 -- [Reduced NADPH---hemoprotein reductase] --> rn:R09452 { (9Z)-Octadecenoic acid }

rn:R04122 <-- [Oxidized NADPH---hemoprotein reductase] --> rn:R08551 -- [Reduced NADPH---hemoprotein reductase] --> rn:R09922 { (+)-Linalool }

rn:R04122 <-- [Oxidized NADPH---hemoprotein reductase] --> rn:R08551 -- [Reduced NADPH---hemoprotein reductase] --> rn:R09923 { (-)-Linalool }

rn:R04122 <-- [Oxidized NADPH---hemoprotein reductase] --> rn:R08551 -- [Reduced NADPH---hemoprotein reductase] --> rn:R09925 { Linalool }

rn:R04122 <-- [Oxidized NADPH---hemoprotein reductase] --> rn:R08551 -- [Reduced NADPH---hemoprotein reductase] --> rn:R09934 { Humulene }

rn:R04122 <-- [Oxidized NADPH---hemoprotein reductase] --> rn:R08551 -- [Reduced NADPH---hemoprotein reductase] --> rn:R10562 { (E,E)-Geranyllinalool }

rn:R04122 <-- [Oxidized NADPH---hemoprotein reductase] --> rn:R08551 -- [Reduced NADPH---hemoprotein reductase] --> rn:R11055 { Myrcene }

rn:R04152 <-- L-Alanine --> rn:R04187 -- Pyruvate --> rn:R05136 { Salicylaldehyde }

rn:R04187 <-- Pyruvate --> rn:R00210 -- Acetyl-CoA --> rn:R08530 { (-)-Menthol }

rn:R04187 <-- Pyruvate --> rn:R00210 -- Acetyl-CoA --> rn:R08531 { (+)-Neomenthol }

rn:R04187 <-- Pyruvate --> rn:R00210 -- Acetyl-CoA --> rn:R08532 { (+)-Borneol }

rn:R04187 <-- Pyruvate --> rn:R00210 -- Acetyl-CoA --> rn:R10474 { Cinnamyl alcohol }

rn:R04188 <-- L-Glutamate --> rn:R00114 -- L-Glutamine --> rn:R02781 { 2,4,6/3,5-Pentahydroxycyclohexanone }

rn:R04188 <-- (S)-Methylmalonate semialdehyde --> rn:R04187 -- Pyruvate --> rn:R05136 { Salicylaldehyde }

rn:R04234 <-- L-Glutamate --> rn:R00114 -- L-Glutamine --> rn:R02781 { 2,4,6/3,5-Pentahydroxycyclohexanone }

rn:R04269 <-- L-Glutamate --> rn:R00114 -- L-Glutamine --> rn:R02781 { 2,4,6/3,5-Pentahydroxycyclohexanone }

rn:R04301 <-- 3-Methoxytyramine --> rn:R04890 -- Hydrogen peroxide --> rn:R01703 { Hexadecanoic acid }

rn:R04397 <-- Acetate --> rn:R01241 -- Phenyl acetate --> rn:R07342 { Phenyl acetate }

rn:R04438 <-- L-Glutamate --> rn:R00114 -- L-Glutamine --> rn:R02781 { 2,4,6/3,5-Pentahydroxycyclohexanone }

rn:R04460 <-- [Oxidized NADPH---hemoprotein reductase] --> rn:R08551 -- [Reduced NADPH---hemoprotein reductase] --> rn:R02468 { (-)-Limonene }

rn:R04460 <-- [Oxidized NADPH---hemoprotein reductase] --> rn:R08551 -- [Reduced NADPH---hemoprotein reductase] --> rn:R02469 { (-)-Limonene }

rn:R04460 <-- [Oxidized NADPH---hemoprotein reductase] --> rn:R08551 -- [Reduced NADPH---hemoprotein reductase] --> rn:R02470 { (-)-Limonene }

rn:R04460 <-- [Oxidized NADPH---hemoprotein reductase] --> rn:R08551 -- [Reduced NADPH---hemoprotein reductase] --> rn:R04366 { Linalool }

rn:R04460 <-- [Oxidized NADPH---hemoprotein reductase] --> rn:R08551 -- [Reduced NADPH---hemoprotein reductase] --> rn:R06119 { d-Limonene }

rn:R04460 <-- [Oxidized NADPH---hemoprotein reductase] --> rn:R08551 -- [Reduced NADPH---hemoprotein reductase] --> rn:R09451 { Hexadecanoic acid }

rn:R04460 <-- [Oxidized NADPH---hemoprotein reductase] --> rn:R08551 -- [Reduced NADPH---hemoprotein reductase] --> rn:R09452 { (9Z)-Octadecenoic acid }

rn:R04460 <-- [Oxidized NADPH---hemoprotein reductase] --> rn:R08551 -- [Reduced NADPH---hemoprotein reductase] --> rn:R09922 { (+)-Linalool }

rn:R04460 <-- [Oxidized NADPH---hemoprotein reductase] --> rn:R08551 -- [Reduced NADPH---hemoprotein reductase] --> rn:R09923 { (-)-Linalool }

rn:R04460 <-- [Oxidized NADPH---hemoprotein reductase] --> rn:R08551 -- [Reduced NADPH---hemoprotein reductase] --> rn:R09925 { Linalool }

rn:R04460 <-- [Oxidized NADPH---hemoprotein reductase] --> rn:R08551 -- [Reduced NADPH---hemoprotein reductase] --> rn:R09934 { Humulene }

rn:R04460 <-- [Oxidized NADPH---hemoprotein reductase] --> rn:R08551 -- [Reduced NADPH---hemoprotein reductase] --> rn:R10562 { (E,E)-Geranyllinalool }

rn:R04460 <-- [Oxidized NADPH---hemoprotein reductase] --> rn:R08551 -- [Reduced NADPH---hemoprotein reductase] --> rn:R11055 { Myrcene }

rn:R04486 <-- Deoxycholic acid --> rn:R07220 -- FADH2 --> rn:R05488 { Styrene }

rn:R04486 <-- Glycine --> rn:R08701 -- Acceptor --> rn:R02234 { Cyclohexanone }

rn:R04486 <-- Glycine --> rn:R08701 -- Acceptor --> rn:R03212 { 3-Hydroxycyclohexanone }

rn:R04487 <-- Deoxycholic acid --> rn:R07220 -- FADH2 --> rn:R05488 { Styrene }

rn:R04751 <-- FADH2 --> rn:R03978 -- FAD --> rn:R05537 { 3-(2-Hydroxyphenyl)propanoate }

rn:R04751 <-- FADH2 --> rn:R05488 -- FAD --> rn:R05537 { 3-(2-Hydroxyphenyl)propanoate }

rn:R04751 <-- FADH2 --> rn:R09517 -- FAD --> rn:R05537 { 3-(2-Hydroxyphenyl)propanoate }

rn:R04751 <-- FADH2 --> rn:R11653 -- FAD --> rn:R05537 { 3-(2-Hydroxyphenyl)propanoate }

rn:R04751 <-- FADH2 --> rn:R12021 -- FAD --> rn:R05537 { 3-(2-Hydroxyphenyl)propanoate }

rn:R04751 <-- FADH2 --> rn:R12023 -- FAD --> rn:R05537 { 3-(2-Hydroxyphenyl)propanoate }

rn:R04751 <-- FADH2 --> rn:R12027 -- FAD --> rn:R05537 { 3-(2-Hydroxyphenyl)propanoate }

rn:R04751 <-- FADH2 --> rn:R12030 -- FAD --> rn:R05537 { 3-(2-Hydroxyphenyl)propanoate }

rn:R04754 <-- FADH2 --> rn:R03978 -- FAD --> rn:R05537 { 3-(2-Hydroxyphenyl)propanoate }

rn:R04754 <-- FADH2 --> rn:R05488 -- FAD --> rn:R05537 { 3-(2-Hydroxyphenyl)propanoate }

rn:R04754 <-- FADH2 --> rn:R09517 -- FAD --> rn:R05537 { 3-(2-Hydroxyphenyl)propanoate }

rn:R04754 <-- FADH2 --> rn:R11653 -- FAD --> rn:R05537 { 3-(2-Hydroxyphenyl)propanoate }

rn:R04754 <-- FADH2 --> rn:R12021 -- FAD --> rn:R05537 { 3-(2-Hydroxyphenyl)propanoate }

rn:R04754 <-- FADH2 --> rn:R12023 -- FAD --> rn:R05537 { 3-(2-Hydroxyphenyl)propanoate }

rn:R04754 <-- FADH2 --> rn:R12027 -- FAD --> rn:R05537 { 3-(2-Hydroxyphenyl)propanoate }

rn:R04754 <-- FADH2 --> rn:R12030 -- FAD --> rn:R05537 { 3-(2-Hydroxyphenyl)propanoate }

rn:R04951 <-- Glycine --> rn:R08701 -- Acceptor --> rn:R02234 { Cyclohexanone }

rn:R04951 <-- Glycine --> rn:R08701 -- Acceptor --> rn:R03212 { 3-Hydroxycyclohexanone }

rn:R04983 <-- 2-Hexaprenyl-3-methyl-6-methoxy-1,4-benzoquinone --> rn:R04984 -- Acceptor --> rn:R02234 { Cyclohexanone }

rn:R04983 <-- 2-Hexaprenyl-3-methyl-6-methoxy-1,4-benzoquinone --> rn:R04984 -- Acceptor --> rn:R03212 { 3-Hydroxycyclohexanone }

rn:R04990 <-- 2-Octaprenyl-3-methyl-6-methoxy-1,4-benzoquinone --> rn:R06146 -- Acceptor --> rn:R02234 { Cyclohexanone }

rn:R04990 <-- 2-Octaprenyl-3-methyl-6-methoxy-1,4-benzoquinone --> rn:R06146 -- Acceptor --> rn:R03212 { 3-Hydroxycyclohexanone }

rn:R05066 <-- (S)-Methylmalonate semialdehyde --> rn:R04187 -- Pyruvate --> rn:R05136 { Salicylaldehyde }

rn:R05085 <-- L-Glutamate --> rn:R00114 -- L-Glutamine --> rn:R02781 { 2,4,6/3,5-Pentahydroxycyclohexanone }

rn:R05224 <-- L-Glutamate --> rn:R00114 -- L-Glutamine --> rn:R02781 { 2,4,6/3,5-Pentahydroxycyclohexanone }

rn:R05225 <-- L-Glutamate --> rn:R00114 -- L-Glutamine --> rn:R02781 { 2,4,6/3,5-Pentahydroxycyclohexanone }

rn:R05259 <-- Sulfur --> rn:R03533 -- Acceptor --> rn:R02234 { Cyclohexanone }

rn:R05259 <-- Sulfur --> rn:R03533 -- Acceptor --> rn:R03212 { 3-Hydroxycyclohexanone }

rn:R05259 <-- [Oxidized NADPH---hemoprotein reductase] --> rn:R08551 -- [Reduced NADPH---hemoprotein reductase] --> rn:R02468 { (-)-Limonene }

rn:R05259 <-- [Oxidized NADPH---hemoprotein reductase] --> rn:R08551 -- [Reduced NADPH---hemoprotein reductase] --> rn:R02469 { (-)-Limonene }

rn:R05259 <-- [Oxidized NADPH---hemoprotein reductase] --> rn:R08551 -- [Reduced NADPH---hemoprotein reductase] --> rn:R02470 { (-)-Limonene }

rn:R05259 <-- [Oxidized NADPH---hemoprotein reductase] --> rn:R08551 -- [Reduced NADPH---hemoprotein reductase] --> rn:R04366 { Linalool }

rn:R05259 <-- [Oxidized NADPH---hemoprotein reductase] --> rn:R08551 -- [Reduced NADPH---hemoprotein reductase] --> rn:R06119 { d-Limonene }

rn:R05259 <-- [Oxidized NADPH---hemoprotein reductase] --> rn:R08551 -- [Reduced NADPH---hemoprotein reductase] --> rn:R09451 { Hexadecanoic acid }

rn:R05259 <-- [Oxidized NADPH---hemoprotein reductase] --> rn:R08551 -- [Reduced NADPH---hemoprotein reductase] --> rn:R09452 { (9Z)-Octadecenoic acid }

rn:R05259 <-- [Oxidized NADPH---hemoprotein reductase] --> rn:R08551 -- [Reduced NADPH---hemoprotein reductase] --> rn:R09922 { (+)-Linalool }

rn:R05259 <-- [Oxidized NADPH---hemoprotein reductase] --> rn:R08551 -- [Reduced NADPH---hemoprotein reductase] --> rn:R09923 { (-)-Linalool }

rn:R05259 <-- [Oxidized NADPH---hemoprotein reductase] --> rn:R08551 -- [Reduced NADPH---hemoprotein reductase] --> rn:R09925 { Linalool }

rn:R05259 <-- [Oxidized NADPH---hemoprotein reductase] --> rn:R08551 -- [Reduced NADPH---hemoprotein reductase] --> rn:R09934 { Humulene }

rn:R05259 <-- [Oxidized NADPH---hemoprotein reductase] --> rn:R08551 -- [Reduced NADPH---hemoprotein reductase] --> rn:R10562 { (E,E)-Geranyllinalool }

rn:R05259 <-- [Oxidized NADPH---hemoprotein reductase] --> rn:R08551 -- [Reduced NADPH---hemoprotein reductase] --> rn:R11055 { Myrcene }

rn:R05267 <-- Glutathione --> rn:R00120 -- Hydrogen peroxide --> rn:R01703 { Hexadecanoic acid }

rn:R05316 <-- Benzoyl-CoA --> rn:R10961 -- Acceptor --> rn:R02234 { Cyclohexanone }

rn:R05316 <-- Benzoyl-CoA --> rn:R10961 -- Acceptor --> rn:R03212 { 3-Hydroxycyclohexanone }

rn:R05365 <-- Anthranilate --> rn:R09517 -- FAD --> rn:R05537 { 3-(2-Hydroxyphenyl)propanoate }

rn:R05371 <-- 3-Oxopropanoate --> rn:R00706 -- Acetyl-CoA --> rn:R08530 { (-)-Menthol }

rn:R05371 <-- 3-Oxopropanoate --> rn:R00706 -- Acetyl-CoA --> rn:R08531 { (+)-Neomenthol }

rn:R05371 <-- 3-Oxopropanoate --> rn:R00706 -- Acetyl-CoA --> rn:R08532 { (+)-Borneol }

rn:R05371 <-- 3-Oxopropanoate --> rn:R00706 -- Acetyl-CoA --> rn:R10474 { Cinnamyl alcohol }

rn:R05372 <-- 3-Oxopropanoate --> rn:R00706 -- Acetyl-CoA --> rn:R08530 { (-)-Menthol }

rn:R05372 <-- 3-Oxopropanoate --> rn:R00706 -- Acetyl-CoA --> rn:R08531 { (+)-Neomenthol }

rn:R05372 <-- 3-Oxopropanoate --> rn:R00706 -- Acetyl-CoA --> rn:R08532 { (+)-Borneol }

rn:R05372 <-- 3-Oxopropanoate --> rn:R00706 -- Acetyl-CoA --> rn:R10474 { Cinnamyl alcohol }

rn:R05378 <-- 3-Oxopropanoate --> rn:R00706 -- Acetyl-CoA --> rn:R08530 { (-)-Menthol }

rn:R05378 <-- 3-Oxopropanoate --> rn:R00706 -- Acetyl-CoA --> rn:R08531 { (+)-Neomenthol }

rn:R05378 <-- 3-Oxopropanoate --> rn:R00706 -- Acetyl-CoA --> rn:R08532 { (+)-Borneol }

rn:R05378 <-- 3-Oxopropanoate --> rn:R00706 -- Acetyl-CoA --> rn:R10474 { Cinnamyl alcohol }

rn:R05432 <-- Dihydrocoumarin --> rn:R03692 -- 3-(2-Hydroxyphenyl)propanoate --> rn:R03369 { 3-(2-Hydroxyphenyl)propanoate }

rn:R05432 <-- Dihydrocoumarin --> rn:R03692 -- 3-(2-Hydroxyphenyl)propanoate --> rn:R03709 { 3-(2-Hydroxyphenyl)propanoate }

rn:R05432 <-- Dihydrocoumarin --> rn:R03692 -- 3-(2-Hydroxyphenyl)propanoate --> rn:R04899 { 3-(2-Hydroxyphenyl)propanoate }

rn:R05432 <-- Dihydrocoumarin --> rn:R03692 -- 3-(2-Hydroxyphenyl)propanoate --> rn:R05537 { 3-(2-Hydroxyphenyl)propanoate }

rn:R05444 <-- Ethylene oxide --> rn:R05351 -- Acetyl-CoA --> rn:R08530 { (-)-Menthol }

rn:R05444 <-- Ethylene oxide --> rn:R05351 -- Acetyl-CoA --> rn:R08531 { (+)-Neomenthol }

rn:R05444 <-- Ethylene oxide --> rn:R05351 -- Acetyl-CoA --> rn:R08532 { (+)-Borneol }

rn:R05444 <-- Ethylene oxide --> rn:R05351 -- Acetyl-CoA --> rn:R10474 { Cinnamyl alcohol }

rn:R05487 <-- [Oxidized NADPH---hemoprotein reductase] --> rn:R08551 -- [Reduced NADPH---hemoprotein reductase] --> rn:R02468 { (-)-Limonene }

rn:R05487 <-- [Oxidized NADPH---hemoprotein reductase] --> rn:R08551 -- [Reduced NADPH---hemoprotein reductase] --> rn:R02469 { (-)-Limonene }

rn:R05487 <-- [Oxidized NADPH---hemoprotein reductase] --> rn:R08551 -- [Reduced NADPH---hemoprotein reductase] --> rn:R02470 { (-)-Limonene }

rn:R05487 <-- [Oxidized NADPH---hemoprotein reductase] --> rn:R08551 -- [Reduced NADPH---hemoprotein reductase] --> rn:R04366 { Linalool }

rn:R05487 <-- [Oxidized NADPH---hemoprotein reductase] --> rn:R08551 -- [Reduced NADPH---hemoprotein reductase] --> rn:R06119 { d-Limonene }

rn:R05487 <-- [Oxidized NADPH---hemoprotein reductase] --> rn:R08551 -- [Reduced NADPH---hemoprotein reductase] --> rn:R09451 { Hexadecanoic acid }

rn:R05487 <-- [Oxidized NADPH---hemoprotein reductase] --> rn:R08551 -- [Reduced NADPH---hemoprotein reductase] --> rn:R09452 { (9Z)-Octadecenoic acid }

rn:R05487 <-- [Oxidized NADPH---hemoprotein reductase] --> rn:R08551 -- [Reduced NADPH---hemoprotein reductase] --> rn:R09922 { (+)-Linalool }

rn:R05487 <-- [Oxidized NADPH---hemoprotein reductase] --> rn:R08551 -- [Reduced NADPH---hemoprotein reductase] --> rn:R09923 { (-)-Linalool }

rn:R05487 <-- [Oxidized NADPH---hemoprotein reductase] --> rn:R08551 -- [Reduced NADPH---hemoprotein reductase] --> rn:R09925 { Linalool }

rn:R05487 <-- [Oxidized NADPH---hemoprotein reductase] --> rn:R08551 -- [Reduced NADPH---hemoprotein reductase] --> rn:R09934 { Humulene }

rn:R05487 <-- [Oxidized NADPH---hemoprotein reductase] --> rn:R08551 -- [Reduced NADPH---hemoprotein reductase] --> rn:R10562 { (E,E)-Geranyllinalool }

rn:R05487 <-- [Oxidized NADPH---hemoprotein reductase] --> rn:R08551 -- [Reduced NADPH---hemoprotein reductase] --> rn:R11055 { Myrcene }

rn:R05506 <-- Benzoyl-CoA --> rn:R10961 -- Acceptor --> rn:R02234 { Cyclohexanone }

rn:R05506 <-- Benzoyl-CoA --> rn:R10961 -- Acceptor --> rn:R03212 { 3-Hydroxycyclohexanone }

rn:R05537 <-- FADH2 --> rn:R03978 -- FAD --> rn:R05537 { 3-(2-Hydroxyphenyl)propanoate }

rn:R05537 <-- FADH2 --> rn:R05488 -- FAD --> rn:R05537 { 3-(2-Hydroxyphenyl)propanoate }

rn:R05537 <-- FADH2 --> rn:R09517 -- FAD --> rn:R05537 { 3-(2-Hydroxyphenyl)propanoate }

rn:R05537 <-- FADH2 --> rn:R11653 -- FAD --> rn:R05537 { 3-(2-Hydroxyphenyl)propanoate }

rn:R05537 <-- FADH2 --> rn:R12021 -- FAD --> rn:R05537 { 3-(2-Hydroxyphenyl)propanoate }

rn:R05537 <-- FADH2 --> rn:R12023 -- FAD --> rn:R05537 { 3-(2-Hydroxyphenyl)propanoate }

rn:R05537 <-- FADH2 --> rn:R12027 -- FAD --> rn:R05537 { 3-(2-Hydroxyphenyl)propanoate }

rn:R05537 <-- FADH2 --> rn:R12030 -- FAD --> rn:R05537 { 3-(2-Hydroxyphenyl)propanoate }

rn:R05585 <-- Benzoyl-CoA --> rn:R10961 -- Acceptor --> rn:R02234 { Cyclohexanone }

rn:R05585 <-- Benzoyl-CoA --> rn:R10961 -- Acceptor --> rn:R03212 { 3-Hydroxycyclohexanone }

rn:R05586 <-- Glutaryl-CoA --> rn:R02487 -- FADH2 --> rn:R05488 { Styrene }

rn:R05587 <-- Benzoyl-CoA --> rn:R10961 -- Acceptor --> rn:R02234 { Cyclohexanone }

rn:R05587 <-- Benzoyl-CoA --> rn:R10961 -- Acceptor --> rn:R03212 { 3-Hydroxycyclohexanone }

rn:R05717 <-- Glutathione --> rn:R00120 -- Hydrogen peroxide --> rn:R01703 { Hexadecanoic acid }

rn:R05728 <-- [Oxidized NADPH---hemoprotein reductase] --> rn:R08551 -- [Reduced NADPH---hemoprotein reductase] --> rn:R02468 { (-)-Limonene }

rn:R05728 <-- [Oxidized NADPH---hemoprotein reductase] --> rn:R08551 -- [Reduced NADPH---hemoprotein reductase] --> rn:R02469 { (-)-Limonene }

rn:R05728 <-- [Oxidized NADPH---hemoprotein reductase] --> rn:R08551 -- [Reduced NADPH---hemoprotein reductase] --> rn:R02470 { (-)-Limonene }

rn:R05728 <-- [Oxidized NADPH---hemoprotein reductase] --> rn:R08551 -- [Reduced NADPH---hemoprotein reductase] --> rn:R04366 { Linalool }

rn:R05728 <-- [Oxidized NADPH---hemoprotein reductase] --> rn:R08551 -- [Reduced NADPH---hemoprotein reductase] --> rn:R06119 { d-Limonene }

rn:R05728 <-- [Oxidized NADPH---hemoprotein reductase] --> rn:R08551 -- [Reduced NADPH---hemoprotein reductase] --> rn:R09451 { Hexadecanoic acid }

rn:R05728 <-- [Oxidized NADPH---hemoprotein reductase] --> rn:R08551 -- [Reduced NADPH---hemoprotein reductase] --> rn:R09452 { (9Z)-Octadecenoic acid }

rn:R05728 <-- [Oxidized NADPH---hemoprotein reductase] --> rn:R08551 -- [Reduced NADPH---hemoprotein reductase] --> rn:R09922 { (+)-Linalool }

rn:R05728 <-- [Oxidized NADPH---hemoprotein reductase] --> rn:R08551 -- [Reduced NADPH---hemoprotein reductase] --> rn:R09923 { (-)-Linalool }

rn:R05728 <-- [Oxidized NADPH---hemoprotein reductase] --> rn:R08551 -- [Reduced NADPH---hemoprotein reductase] --> rn:R09925 { Linalool }

rn:R05728 <-- [Oxidized NADPH---hemoprotein reductase] --> rn:R08551 -- [Reduced NADPH---hemoprotein reductase] --> rn:R09934 { Humulene }

rn:R05728 <-- [Oxidized NADPH---hemoprotein reductase] --> rn:R08551 -- [Reduced NADPH---hemoprotein reductase] --> rn:R10562 { (E,E)-Geranyllinalool }

rn:R05728 <-- [Oxidized NADPH---hemoprotein reductase] --> rn:R08551 -- [Reduced NADPH---hemoprotein reductase] --> rn:R11055 { Myrcene }

rn:R05808 <-- Cobalt-factor III --> rn:R11580 -- Acceptor --> rn:R02234 { Cyclohexanone }

rn:R05808 <-- Cobalt-factor III --> rn:R11580 -- Acceptor --> rn:R03212 { 3-Hydroxycyclohexanone }

rn:R05815 <-- L-Glutamate --> rn:R00114 -- L-Glutamine --> rn:R02781 { 2,4,6/3,5-Pentahydroxycyclohexanone }

rn:R05835 <-- Glycine --> rn:R08701 -- Acceptor --> rn:R02234 { Cyclohexanone }

rn:R05835 <-- Glycine --> rn:R08701 -- Acceptor --> rn:R03212 { 3-Hydroxycyclohexanone }

rn:R05836 <-- Deoxycholic acid --> rn:R07220 -- FADH2 --> rn:R05488 { Styrene }

rn:R05838 <-- Pyridoxine phosphate --> rn:R00278 -- Hydrogen peroxide --> rn:R01703 { Hexadecanoic acid }

rn:R05839 <-- Pyridoxal --> rn:R01709 -- Hydrogen peroxide --> rn:R01703 { Hexadecanoic acid }

rn:R05840 <-- Pyridoxine --> rn:R01711 -- Hydrogen peroxide --> rn:R01703 { Hexadecanoic acid }

rn:R05840 <-- Pyridoxine --> rn:R01910 -- Hydrogen peroxide --> rn:R01703 { Hexadecanoic acid }

rn:R06602 <-- Pyruvate --> rn:R00210 -- Acetyl-CoA --> rn:R08530 { (-)-Menthol }

rn:R06602 <-- Pyruvate --> rn:R00210 -- Acetyl-CoA --> rn:R08531 { (+)-Neomenthol }

rn:R06602 <-- Pyruvate --> rn:R00210 -- Acetyl-CoA --> rn:R08532 { (+)-Borneol }

rn:R06602 <-- Pyruvate --> rn:R00210 -- Acetyl-CoA --> rn:R10474 { Cinnamyl alcohol }

rn:R06603 <-- Pyruvate --> rn:R00210 -- Acetyl-CoA --> rn:R08530 { (-)-Menthol }

rn:R06603 <-- Pyruvate --> rn:R00210 -- Acetyl-CoA --> rn:R08531 { (+)-Neomenthol }

rn:R06603 <-- Pyruvate --> rn:R00210 -- Acetyl-CoA --> rn:R08532 { (+)-Borneol }

rn:R06603 <-- Pyruvate --> rn:R00210 -- Acetyl-CoA --> rn:R10474 { Cinnamyl alcohol }

rn:R06632 <-- (S)-4-Hydroxymandelate --> rn:R02673 -- Hydrogen peroxide --> rn:R01703 { Hexadecanoic acid }

rn:R06632 <-- (S)-4-Hydroxymandelate --> rn:R06633 -- Hydrogen peroxide --> rn:R01703 { Hexadecanoic acid }

rn:R06747 <-- Pyrrole-2-carbonyl-[pcp] --> rn:R12021 -- FAD --> rn:R05537 { 3-(2-Hydroxyphenyl)propanoate }

rn:R06747 <-- Pyrrole-2-carbonyl-[pcp] --> rn:R12023 -- FAD --> rn:R05537 { 3-(2-Hydroxyphenyl)propanoate }

rn:R06747 <-- Pyrrole-2-carbonyl-[pcp] --> rn:R12027 -- FAD --> rn:R05537 { 3-(2-Hydroxyphenyl)propanoate }

rn:R06747 <-- Pyrrole-2-carbonyl-[pcp] --> rn:R12030 -- FAD --> rn:R05537 { 3-(2-Hydroxyphenyl)propanoate }

rn:R06748 <-- Pyrrole-2-carbonyl-[pcp] --> rn:R12021 -- FAD --> rn:R05537 { 3-(2-Hydroxyphenyl)propanoate }

rn:R06748 <-- Pyrrole-2-carbonyl-[pcp] --> rn:R12023 -- FAD --> rn:R05537 { 3-(2-Hydroxyphenyl)propanoate }

rn:R06748 <-- Pyrrole-2-carbonyl-[pcp] --> rn:R12027 -- FAD --> rn:R05537 { 3-(2-Hydroxyphenyl)propanoate }

rn:R06748 <-- Pyrrole-2-carbonyl-[pcp] --> rn:R12030 -- FAD --> rn:R05537 { 3-(2-Hydroxyphenyl)propanoate }

rn:R06758 <-- 3-Dimethylallyl-4-hydroxymandelic acid --> rn:R06759 -- 3-Dimethylallyl-4-hydroxybenzoate --> rn:R06776 { 3-Amino-4,7-dihydroxy-8-chlorocoumarin }

rn:R06758 <-- 3-Dimethylallyl-4-hydroxymandelic acid --> rn:R06759 -- 3-Dimethylallyl-4-hydroxybenzoate --> rn:R10453 { 3-Amino-4,7-dihydroxycoumarin }

rn:R06761 <-- 3-Dimethylallyl-4-hydroxybenzaldehyde --> rn:R06763 -- 3-Dimethylallyl-4-hydroxybenzoate --> rn:R06776 { 3-Amino-4,7-dihydroxy-8-chlorocoumarin }

rn:R06761 <-- 3-Dimethylallyl-4-hydroxybenzaldehyde --> rn:R06763 -- 3-Dimethylallyl-4-hydroxybenzoate --> rn:R10453 { 3-Amino-4,7-dihydroxycoumarin }

rn:R06762 <-- 3-Dimethylallyl-4-hydroxybenzaldehyde --> rn:R06763 -- 3-Dimethylallyl-4-hydroxybenzoate --> rn:R06776 { 3-Amino-4,7-dihydroxy-8-chlorocoumarin }

rn:R06762 <-- 3-Dimethylallyl-4-hydroxybenzaldehyde --> rn:R06763 -- 3-Dimethylallyl-4-hydroxybenzoate --> rn:R10453 { 3-Amino-4,7-dihydroxycoumarin }

rn:R06765 <-- 3-Amino-4,7-dihydroxycoumarin --> rn:R06777 -- 5-[[(4,7-Dihydroxy-2-oxo-2H-1-benzopyran-3-yl)amino]carbonyl]-4-methyl-1H-pyrrole-3-carboxylate --> rn:R06778 { 3-Amino-4,7-dihydroxycoumarin }

rn:R06859 <-- Phylloquinone --> rn:R03510 -- Acceptor --> rn:R02234 { Cyclohexanone }

rn:R06859 <-- Phylloquinone --> rn:R03510 -- Acceptor --> rn:R03212 { 3-Hydroxycyclohexanone }

rn:R06893 <-- Acetate --> rn:R01241 -- Phenyl acetate --> rn:R07342 { Phenyl acetate }

rn:R06895 <-- Protoporphyrinogen IX --> rn:R03222 -- Hydrogen peroxide --> rn:R01703 { Hexadecanoic acid }

rn:R06902 <-- FAD --> rn:R02487 -- FADH2 --> rn:R05488 { Styrene }

rn:R06902 <-- FAD --> rn:R04095 -- FADH2 --> rn:R05488 { Styrene }

rn:R06902 <-- FAD --> rn:R05537 -- FADH2 --> rn:R05488 { Styrene }

rn:R06902 <-- FAD --> rn:R05537 -- Acetyl-CoA --> rn:R08530 { (-)-Menthol }

rn:R06902 <-- FAD --> rn:R05537 -- Acetyl-CoA --> rn:R08531 { (+)-Neomenthol }

rn:R06902 <-- FAD --> rn:R05537 -- Acetyl-CoA --> rn:R08532 { (+)-Borneol }

rn:R06902 <-- FAD --> rn:R05537 -- Acetyl-CoA --> rn:R10474 { Cinnamyl alcohol }

rn:R06902 <-- FAD --> rn:R06943 -- FADH2 --> rn:R05488 { Styrene }

rn:R06902 <-- FAD --> rn:R07220 -- FADH2 --> rn:R05488 { Styrene }

rn:R06902 <-- FAD --> rn:R09520 -- FADH2 --> rn:R05488 { Styrene }

rn:R06902 <-- FAD --> rn:R11130 -- FADH2 --> rn:R05488 { Styrene }

rn:R06913 <-- Pyruvate --> rn:R00210 -- Acetyl-CoA --> rn:R08530 { (-)-Menthol }

rn:R06913 <-- Pyruvate --> rn:R00210 -- Acetyl-CoA --> rn:R08531 { (+)-Neomenthol }

rn:R06913 <-- Pyruvate --> rn:R00210 -- Acetyl-CoA --> rn:R08532 { (+)-Borneol }

rn:R06913 <-- Pyruvate --> rn:R00210 -- Acetyl-CoA --> rn:R10474 { Cinnamyl alcohol }

rn:R06923 <-- Pyruvate --> rn:R00210 -- Acetyl-CoA --> rn:R08530 { (-)-Menthol }

rn:R06923 <-- Pyruvate --> rn:R00210 -- Acetyl-CoA --> rn:R08531 { (+)-Neomenthol }

rn:R06923 <-- Pyruvate --> rn:R00210 -- Acetyl-CoA --> rn:R08532 { (+)-Borneol }

rn:R06923 <-- Pyruvate --> rn:R00210 -- Acetyl-CoA --> rn:R10474 { Cinnamyl alcohol }

rn:R06934 <-- Pyruvate --> rn:R00210 -- Acetyl-CoA --> rn:R08530 { (-)-Menthol }

rn:R06934 <-- Pyruvate --> rn:R00210 -- Acetyl-CoA --> rn:R08531 { (+)-Neomenthol }

rn:R06934 <-- Pyruvate --> rn:R00210 -- Acetyl-CoA --> rn:R08532 { (+)-Borneol }

rn:R06934 <-- Pyruvate --> rn:R00210 -- Acetyl-CoA --> rn:R10474 { Cinnamyl alcohol }

rn:R06943 <-- FADH2 --> rn:R03978 -- FAD --> rn:R05537 { 3-(2-Hydroxyphenyl)propanoate }

rn:R06943 <-- FADH2 --> rn:R05488 -- FAD --> rn:R05537 { 3-(2-Hydroxyphenyl)propanoate }

rn:R06943 <-- FADH2 --> rn:R09517 -- FAD --> rn:R05537 { 3-(2-Hydroxyphenyl)propanoate }

rn:R06943 <-- FADH2 --> rn:R11653 -- FAD --> rn:R05537 { 3-(2-Hydroxyphenyl)propanoate }

rn:R06943 <-- FADH2 --> rn:R12021 -- FAD --> rn:R05537 { 3-(2-Hydroxyphenyl)propanoate }

rn:R06943 <-- FADH2 --> rn:R12023 -- FAD --> rn:R05537 { 3-(2-Hydroxyphenyl)propanoate }

rn:R06943 <-- FADH2 --> rn:R12027 -- FAD --> rn:R05537 { 3-(2-Hydroxyphenyl)propanoate }

rn:R06943 <-- FADH2 --> rn:R12030 -- FAD --> rn:R05537 { 3-(2-Hydroxyphenyl)propanoate }

rn:R07041 <-- [Oxidized NADPH---hemoprotein reductase] --> rn:R08551 -- [Reduced NADPH---hemoprotein reductase] --> rn:R02468 { (-)-Limonene }

rn:R07041 <-- [Oxidized NADPH---hemoprotein reductase] --> rn:R08551 -- [Reduced NADPH---hemoprotein reductase] --> rn:R02469 { (-)-Limonene }

rn:R07041 <-- [Oxidized NADPH---hemoprotein reductase] --> rn:R08551 -- [Reduced NADPH---hemoprotein reductase] --> rn:R02470 { (-)-Limonene }

rn:R07041 <-- [Oxidized NADPH---hemoprotein reductase] --> rn:R08551 -- [Reduced NADPH---hemoprotein reductase] --> rn:R04366 { Linalool }

rn:R07041 <-- [Oxidized NADPH---hemoprotein reductase] --> rn:R08551 -- [Reduced NADPH---hemoprotein reductase] --> rn:R06119 { d-Limonene }

rn:R07041 <-- [Oxidized NADPH---hemoprotein reductase] --> rn:R08551 -- [Reduced NADPH---hemoprotein reductase] --> rn:R09451 { Hexadecanoic acid }

rn:R07041 <-- [Oxidized NADPH---hemoprotein reductase] --> rn:R08551 -- [Reduced NADPH---hemoprotein reductase] --> rn:R09452 { (9Z)-Octadecenoic acid }

rn:R07041 <-- [Oxidized NADPH---hemoprotein reductase] --> rn:R08551 -- [Reduced NADPH---hemoprotein reductase] --> rn:R09922 { (+)-Linalool }

rn:R07041 <-- [Oxidized NADPH---hemoprotein reductase] --> rn:R08551 -- [Reduced NADPH---hemoprotein reductase] --> rn:R09923 { (-)-Linalool }

rn:R07041 <-- [Oxidized NADPH---hemoprotein reductase] --> rn:R08551 -- [Reduced NADPH---hemoprotein reductase] --> rn:R09925 { Linalool }

rn:R07041 <-- [Oxidized NADPH---hemoprotein reductase] --> rn:R08551 -- [Reduced NADPH---hemoprotein reductase] --> rn:R09934 { Humulene }

rn:R07041 <-- [Oxidized NADPH---hemoprotein reductase] --> rn:R08551 -- [Reduced NADPH---hemoprotein reductase] --> rn:R10562 { (E,E)-Geranyllinalool }

rn:R07041 <-- [Oxidized NADPH---hemoprotein reductase] --> rn:R08551 -- [Reduced NADPH---hemoprotein reductase] --> rn:R11055 { Myrcene }

rn:R07046 <-- [Oxidized NADPH---hemoprotein reductase] --> rn:R08551 -- [Reduced NADPH---hemoprotein reductase] --> rn:R02468 { (-)-Limonene }

rn:R07046 <-- [Oxidized NADPH---hemoprotein reductase] --> rn:R08551 -- [Reduced NADPH---hemoprotein reductase] --> rn:R02469 { (-)-Limonene }

rn:R07046 <-- [Oxidized NADPH---hemoprotein reductase] --> rn:R08551 -- [Reduced NADPH---hemoprotein reductase] --> rn:R02470 { (-)-Limonene }

rn:R07046 <-- [Oxidized NADPH---hemoprotein reductase] --> rn:R08551 -- [Reduced NADPH---hemoprotein reductase] --> rn:R04366 { Linalool }

rn:R07046 <-- [Oxidized NADPH---hemoprotein reductase] --> rn:R08551 -- [Reduced NADPH---hemoprotein reductase] --> rn:R06119 { d-Limonene }

rn:R07046 <-- [Oxidized NADPH---hemoprotein reductase] --> rn:R08551 -- [Reduced NADPH---hemoprotein reductase] --> rn:R09451 { Hexadecanoic acid }

rn:R07046 <-- [Oxidized NADPH---hemoprotein reductase] --> rn:R08551 -- [Reduced NADPH---hemoprotein reductase] --> rn:R09452 { (9Z)-Octadecenoic acid }

rn:R07046 <-- [Oxidized NADPH---hemoprotein reductase] --> rn:R08551 -- [Reduced NADPH---hemoprotein reductase] --> rn:R09922 { (+)-Linalool }

rn:R07046 <-- [Oxidized NADPH---hemoprotein reductase] --> rn:R08551 -- [Reduced NADPH---hemoprotein reductase] --> rn:R09923 { (-)-Linalool }

rn:R07046 <-- [Oxidized NADPH---hemoprotein reductase] --> rn:R08551 -- [Reduced NADPH---hemoprotein reductase] --> rn:R09925 { Linalool }

rn:R07046 <-- [Oxidized NADPH---hemoprotein reductase] --> rn:R08551 -- [Reduced NADPH---hemoprotein reductase] --> rn:R09934 { Humulene }

rn:R07046 <-- [Oxidized NADPH---hemoprotein reductase] --> rn:R08551 -- [Reduced NADPH---hemoprotein reductase] --> rn:R10562 { (E,E)-Geranyllinalool }

rn:R07046 <-- [Oxidized NADPH---hemoprotein reductase] --> rn:R08551 -- [Reduced NADPH---hemoprotein reductase] --> rn:R11055 { Myrcene }

rn:R07064 <-- Linoleate --> rn:R07063 -- Acceptor --> rn:R02234 { Cyclohexanone }

rn:R07064 <-- Linoleate --> rn:R07063 -- Acceptor --> rn:R03212 { 3-Hydroxycyclohexanone }

rn:R07203 <-- [Oxidized NADPH---hemoprotein reductase] --> rn:R08551 -- [Reduced NADPH---hemoprotein reductase] --> rn:R02468 { (-)-Limonene }

rn:R07203 <-- [Oxidized NADPH---hemoprotein reductase] --> rn:R08551 -- [Reduced NADPH---hemoprotein reductase] --> rn:R02469 { (-)-Limonene }

rn:R07203 <-- [Oxidized NADPH---hemoprotein reductase] --> rn:R08551 -- [Reduced NADPH---hemoprotein reductase] --> rn:R02470 { (-)-Limonene }

rn:R07203 <-- [Oxidized NADPH---hemoprotein reductase] --> rn:R08551 -- [Reduced NADPH---hemoprotein reductase] --> rn:R04366 { Linalool }

rn:R07203 <-- [Oxidized NADPH---hemoprotein reductase] --> rn:R08551 -- [Reduced NADPH---hemoprotein reductase] --> rn:R06119 { d-Limonene }

rn:R07203 <-- [Oxidized NADPH---hemoprotein reductase] --> rn:R08551 -- [Reduced NADPH---hemoprotein reductase] --> rn:R09451 { Hexadecanoic acid }

rn:R07203 <-- [Oxidized NADPH---hemoprotein reductase] --> rn:R08551 -- [Reduced NADPH---hemoprotein reductase] --> rn:R09452 { (9Z)-Octadecenoic acid }

rn:R07203 <-- [Oxidized NADPH---hemoprotein reductase] --> rn:R08551 -- [Reduced NADPH---hemoprotein reductase] --> rn:R09922 { (+)-Linalool }

rn:R07203 <-- [Oxidized NADPH---hemoprotein reductase] --> rn:R08551 -- [Reduced NADPH---hemoprotein reductase] --> rn:R09923 { (-)-Linalool }

rn:R07203 <-- [Oxidized NADPH---hemoprotein reductase] --> rn:R08551 -- [Reduced NADPH---hemoprotein reductase] --> rn:R09925 { Linalool }

rn:R07203 <-- [Oxidized NADPH---hemoprotein reductase] --> rn:R08551 -- [Reduced NADPH---hemoprotein reductase] --> rn:R09934 { Humulene }

rn:R07203 <-- [Oxidized NADPH---hemoprotein reductase] --> rn:R08551 -- [Reduced NADPH---hemoprotein reductase] --> rn:R10562 { (E,E)-Geranyllinalool }

rn:R07203 <-- [Oxidized NADPH---hemoprotein reductase] --> rn:R08551 -- [Reduced NADPH---hemoprotein reductase] --> rn:R11055 { Myrcene }

rn:R07205 <-- [Oxidized NADPH---hemoprotein reductase] --> rn:R08551 -- [Reduced NADPH---hemoprotein reductase] --> rn:R02468 { (-)-Limonene }

rn:R07205 <-- [Oxidized NADPH---hemoprotein reductase] --> rn:R08551 -- [Reduced NADPH---hemoprotein reductase] --> rn:R02469 { (-)-Limonene }

rn:R07205 <-- [Oxidized NADPH---hemoprotein reductase] --> rn:R08551 -- [Reduced NADPH---hemoprotein reductase] --> rn:R02470 { (-)-Limonene }

rn:R07205 <-- [Oxidized NADPH---hemoprotein reductase] --> rn:R08551 -- [Reduced NADPH---hemoprotein reductase] --> rn:R04366 { Linalool }

rn:R07205 <-- [Oxidized NADPH---hemoprotein reductase] --> rn:R08551 -- [Reduced NADPH---hemoprotein reductase] --> rn:R06119 { d-Limonene }

rn:R07205 <-- [Oxidized NADPH---hemoprotein reductase] --> rn:R08551 -- [Reduced NADPH---hemoprotein reductase] --> rn:R09451 { Hexadecanoic acid }

rn:R07205 <-- [Oxidized NADPH---hemoprotein reductase] --> rn:R08551 -- [Reduced NADPH---hemoprotein reductase] --> rn:R09452 { (9Z)-Octadecenoic acid }

rn:R07205 <-- [Oxidized NADPH---hemoprotein reductase] --> rn:R08551 -- [Reduced NADPH---hemoprotein reductase] --> rn:R09922 { (+)-Linalool }

rn:R07205 <-- [Oxidized NADPH---hemoprotein reductase] --> rn:R08551 -- [Reduced NADPH---hemoprotein reductase] --> rn:R09923 { (-)-Linalool }

rn:R07205 <-- [Oxidized NADPH---hemoprotein reductase] --> rn:R08551 -- [Reduced NADPH---hemoprotein reductase] --> rn:R09925 { Linalool }

rn:R07205 <-- [Oxidized NADPH---hemoprotein reductase] --> rn:R08551 -- [Reduced NADPH---hemoprotein reductase] --> rn:R09934 { Humulene }

rn:R07205 <-- [Oxidized NADPH---hemoprotein reductase] --> rn:R08551 -- [Reduced NADPH---hemoprotein reductase] --> rn:R10562 { (E,E)-Geranyllinalool }

rn:R07205 <-- [Oxidized NADPH---hemoprotein reductase] --> rn:R08551 -- [Reduced NADPH---hemoprotein reductase] --> rn:R11055 { Myrcene }

rn:R07206 <-- [Oxidized NADPH---hemoprotein reductase] --> rn:R08551 -- [Reduced NADPH---hemoprotein reductase] --> rn:R02468 { (-)-Limonene }

rn:R07206 <-- [Oxidized NADPH---hemoprotein reductase] --> rn:R08551 -- [Reduced NADPH---hemoprotein reductase] --> rn:R02469 { (-)-Limonene }

rn:R07206 <-- [Oxidized NADPH---hemoprotein reductase] --> rn:R08551 -- [Reduced NADPH---hemoprotein reductase] --> rn:R02470 { (-)-Limonene }

rn:R07206 <-- [Oxidized NADPH---hemoprotein reductase] --> rn:R08551 -- [Reduced NADPH---hemoprotein reductase] --> rn:R04366 { Linalool }

rn:R07206 <-- [Oxidized NADPH---hemoprotein reductase] --> rn:R08551 -- [Reduced NADPH---hemoprotein reductase] --> rn:R06119 { d-Limonene }

rn:R07206 <-- [Oxidized NADPH---hemoprotein reductase] --> rn:R08551 -- [Reduced NADPH---hemoprotein reductase] --> rn:R09451 { Hexadecanoic acid }

rn:R07206 <-- [Oxidized NADPH---hemoprotein reductase] --> rn:R08551 -- [Reduced NADPH---hemoprotein reductase] --> rn:R09452 { (9Z)-Octadecenoic acid }

rn:R07206 <-- [Oxidized NADPH---hemoprotein reductase] --> rn:R08551 -- [Reduced NADPH---hemoprotein reductase] --> rn:R09922 { (+)-Linalool }

rn:R07206 <-- [Oxidized NADPH---hemoprotein reductase] --> rn:R08551 -- [Reduced NADPH---hemoprotein reductase] --> rn:R09923 { (-)-Linalool }

rn:R07206 <-- [Oxidized NADPH---hemoprotein reductase] --> rn:R08551 -- [Reduced NADPH---hemoprotein reductase] --> rn:R09925 { Linalool }

rn:R07206 <-- [Oxidized NADPH---hemoprotein reductase] --> rn:R08551 -- [Reduced NADPH---hemoprotein reductase] --> rn:R09934 { Humulene }

rn:R07206 <-- [Oxidized NADPH---hemoprotein reductase] --> rn:R08551 -- [Reduced NADPH---hemoprotein reductase] --> rn:R10562 { (E,E)-Geranyllinalool }

rn:R07206 <-- [Oxidized NADPH---hemoprotein reductase] --> rn:R08551 -- [Reduced NADPH---hemoprotein reductase] --> rn:R11055 { Myrcene }

rn:R07212 <-- 3,4-Dihydroxy-L-phenylalanine --> rn:R02080 -- Dopamine --> rn:R08447 { 4-Hydroxydihydrocinnamaldehyde }

rn:R07212 <-- 3,4-Dihydroxy-L-phenylalanine --> rn:R11916 -- Hydrogen peroxide --> rn:R01703 { Hexadecanoic acid }

rn:R07220 <-- FADH2 --> rn:R03978 -- FAD --> rn:R05537 { 3-(2-Hydroxyphenyl)propanoate }

rn:R07220 <-- FADH2 --> rn:R05488 -- FAD --> rn:R05537 { 3-(2-Hydroxyphenyl)propanoate }

rn:R07220 <-- FADH2 --> rn:R09517 -- FAD --> rn:R05537 { 3-(2-Hydroxyphenyl)propanoate }

rn:R07220 <-- FADH2 --> rn:R11653 -- FAD --> rn:R05537 { 3-(2-Hydroxyphenyl)propanoate }

rn:R07220 <-- FADH2 --> rn:R12021 -- FAD --> rn:R05537 { 3-(2-Hydroxyphenyl)propanoate }

rn:R07220 <-- FADH2 --> rn:R12023 -- FAD --> rn:R05537 { 3-(2-Hydroxyphenyl)propanoate }

rn:R07220 <-- FADH2 --> rn:R12027 -- FAD --> rn:R05537 { 3-(2-Hydroxyphenyl)propanoate }

rn:R07220 <-- FADH2 --> rn:R12030 -- FAD --> rn:R05537 { 3-(2-Hydroxyphenyl)propanoate }

rn:R07230 <-- Thyroxine --> rn:R03734 -- Acceptor --> rn:R02234 { Cyclohexanone }

rn:R07230 <-- Thyroxine --> rn:R03734 -- Acceptor --> rn:R03212 { 3-Hydroxycyclohexanone }

rn:R07276 <-- L-Glutamate --> rn:R00114 -- L-Glutamine --> rn:R02781 { 2,4,6/3,5-Pentahydroxycyclohexanone }

rn:R07294 <-- alpha-Oxo-benzeneacetic acid --> rn:R01764 -- Benzaldehyde --> rn:R01419 { Benzaldehyde }

rn:R07294 <-- alpha-Oxo-benzeneacetic acid --> rn:R01764 -- Benzaldehyde --> rn:R01420 { Benzaldehyde }

rn:R07295 <-- Deoxycholic acid --> rn:R07220 -- FADH2 --> rn:R05488 { Styrene }

rn:R07301 <-- Acetate --> rn:R01241 -- Phenyl acetate --> rn:R07342 { Phenyl acetate }

rn:R07301 <-- Phenethylamine --> rn:R02613 -- Hydrogen peroxide --> rn:R01703 { Hexadecanoic acid }

rn:R07342 <-- Phenol --> rn:R01241 -- Phenyl acetate --> rn:R07342 { Phenyl acetate } # rn:R07342 <-- Acetate --> rn:R01241 -- Phenyl acetate --> rn:R07342 { Phenyl acetate }

rn:R07342 <-- Phenol --> rn:R11653 -- FAD --> rn:R05537 { 3-(2-Hydroxyphenyl)propanoate }

rn:R07403 <-- [Oxidized NADPH---hemoprotein reductase] --> rn:R08551 -- [Reduced NADPH---hemoprotein reductase] --> rn:R02468 { (-)-Limonene }

rn:R07403 <-- [Oxidized NADPH---hemoprotein reductase] --> rn:R08551 -- [Reduced NADPH---hemoprotein reductase] --> rn:R02469 { (-)-Limonene }

rn:R07403 <-- [Oxidized NADPH---hemoprotein reductase] --> rn:R08551 -- [Reduced NADPH---hemoprotein reductase] --> rn:R02470 { (-)-Limonene }

rn:R07403 <-- [Oxidized NADPH---hemoprotein reductase] --> rn:R08551 -- [Reduced NADPH---hemoprotein reductase] --> rn:R04366 { Linalool }

rn:R07403 <-- [Oxidized NADPH---hemoprotein reductase] --> rn:R08551 -- [Reduced NADPH---hemoprotein reductase] --> rn:R06119 { d-Limonene }

rn:R07403 <-- [Oxidized NADPH---hemoprotein reductase] --> rn:R08551 -- [Reduced NADPH---hemoprotein reductase] --> rn:R09451 { Hexadecanoic acid }

rn:R07403 <-- [Oxidized NADPH---hemoprotein reductase] --> rn:R08551 -- [Reduced NADPH---hemoprotein reductase] --> rn:R09452 { (9Z)-Octadecenoic acid }

rn:R07403 <-- [Oxidized NADPH---hemoprotein reductase] --> rn:R08551 -- [Reduced NADPH---hemoprotein reductase] --> rn:R09922 { (+)-Linalool }

rn:R07403 <-- [Oxidized NADPH---hemoprotein reductase] --> rn:R08551 -- [Reduced NADPH---hemoprotein reductase] --> rn:R09923 { (-)-Linalool }

rn:R07403 <-- [Oxidized NADPH---hemoprotein reductase] --> rn:R08551 -- [Reduced NADPH---hemoprotein reductase] --> rn:R09925 { Linalool }

rn:R07403 <-- [Oxidized NADPH---hemoprotein reductase] --> rn:R08551 -- [Reduced NADPH---hemoprotein reductase] --> rn:R09934 { Humulene }

rn:R07403 <-- [Oxidized NADPH---hemoprotein reductase] --> rn:R08551 -- [Reduced NADPH---hemoprotein reductase] --> rn:R10562 { (E,E)-Geranyllinalool }

rn:R07403 <-- [Oxidized NADPH---hemoprotein reductase] --> rn:R08551 -- [Reduced NADPH---hemoprotein reductase] --> rn:R11055 { Myrcene }

rn:R07664 <-- alpha-Oxo-benzeneacetic acid --> rn:R01764 -- Benzaldehyde --> rn:R01419 { Benzaldehyde }

rn:R07664 <-- alpha-Oxo-benzeneacetic acid --> rn:R01764 -- Benzaldehyde --> rn:R01420 { Benzaldehyde }

rn:R07666 <-- 3-Hydroxybenzoate --> rn:R01508 -- Acceptor --> rn:R02234 { Cyclohexanone }

rn:R07666 <-- 3-Hydroxybenzoate --> rn:R01508 -- Acceptor --> rn:R03212 { 3-Hydroxycyclohexanone }

rn:R07685 <-- Pyruvate --> rn:R00210 -- Acetyl-CoA --> rn:R08530 { (-)-Menthol }

rn:R07685 <-- Pyruvate --> rn:R00210 -- Acetyl-CoA --> rn:R08531 { (+)-Neomenthol }

rn:R07685 <-- Pyruvate --> rn:R00210 -- Acetyl-CoA --> rn:R08532 { (+)-Borneol }

rn:R07685 <-- Pyruvate --> rn:R00210 -- Acetyl-CoA --> rn:R10474 { Cinnamyl alcohol }

rn:R07697 <-- Phenol --> rn:R01241 -- Phenyl acetate --> rn:R07342 { Phenyl acetate }

rn:R07697 <-- Phenol --> rn:R11653 -- FAD --> rn:R05537 { 3-(2-Hydroxyphenyl)propanoate }

rn:R07734 <-- Anthranilate --> rn:R09517 -- FAD --> rn:R05537 { 3-(2-Hydroxyphenyl)propanoate }

rn:R07796 <-- trans-Cinnamate --> rn:R11070 -- Styrene --> rn:R05417 { Styrene }

rn:R07796 <-- trans-Cinnamate --> rn:R11070 -- Styrene --> rn:R05488 { Styrene }

rn:R07855 <-- Phenylacetic acid --> rn:R05487 -- 2-Hydroxyphenylacetate --> rn:R05001 { 2-Hydroxyphenylacetate }

rn:R07855 <-- Phenylacetic acid --> rn:R05487 -- 2-Hydroxyphenylacetate --> rn:R05450 { 2-Hydroxyphenylacetate }

rn:R07859 <-- (9Z,12Z,15Z)-Octadecatrienoic acid --> rn:R07861 -- Acceptor --> rn:R02234 { Cyclohexanone }

rn:R07859 <-- (9Z,12Z,15Z)-Octadecatrienoic acid --> rn:R07861 -- Acceptor --> rn:R03212 { 3-Hydroxycyclohexanone }

rn:R07860 <-- (9Z,12Z,15Z)-Octadecatrienoic acid --> rn:R07861 -- Acceptor --> rn:R02234 { Cyclohexanone }

rn:R07860 <-- (9Z,12Z,15Z)-Octadecatrienoic acid --> rn:R07861 -- Acceptor --> rn:R03212 { 3-Hydroxycyclohexanone }

rn:R08165 <-- 2-Succinyl-5-enolpyruvyl-6-hydroxy-3-cyclohexene-1-carboxylate --> rn:R08166 -- Pyruvate --> rn:R05136 { Salicylaldehyde }

rn:R08166 <-- Pyruvate --> rn:R00210 -- Acetyl-CoA --> rn:R08530 { (-)-Menthol }

rn:R08166 <-- Pyruvate --> rn:R00210 -- Acetyl-CoA --> rn:R08531 { (+)-Neomenthol }

rn:R08166 <-- Pyruvate --> rn:R00210 -- Acetyl-CoA --> rn:R08532 { (+)-Borneol }

rn:R08166 <-- Pyruvate --> rn:R00210 -- Acetyl-CoA --> rn:R10474 { Cinnamyl alcohol }

rn:R08168 <-- S-(Indolylmethylthiohydroximoyl)-L-cysteine --> rn:R08170 -- Pyruvate --> rn:R05136 { Salicylaldehyde }

rn:R08177 <-- Linoleate --> rn:R07063 -- Acceptor --> rn:R02234 { Cyclohexanone }

rn:R08177 <-- Linoleate --> rn:R07063 -- Acceptor --> rn:R03212 { 3-Hydroxycyclohexanone }

rn:R08178 <-- (9Z,12Z,15Z)-Octadecatrienoic acid --> rn:R07861 -- Acceptor --> rn:R02234 { Cyclohexanone }

rn:R08178 <-- (9Z,12Z,15Z)-Octadecatrienoic acid --> rn:R07861 -- Acceptor --> rn:R03212 { 3-Hydroxycyclohexanone }

rn:R08183 <-- Arachidonate --> rn:R01599 -- Acceptor --> rn:R02234 { Cyclohexanone }

rn:R08183 <-- Arachidonate --> rn:R01599 -- Acceptor --> rn:R03212 { 3-Hydroxycyclohexanone }

rn:R08200 <-- Pyruvate --> rn:R00210 -- Acetyl-CoA --> rn:R08530 { (-)-Menthol }

rn:R08200 <-- Pyruvate --> rn:R00210 -- Acetyl-CoA --> rn:R08531 { (+)-Neomenthol }

rn:R08200 <-- Pyruvate --> rn:R00210 -- Acetyl-CoA --> rn:R08532 { (+)-Borneol }

rn:R08200 <-- Pyruvate --> rn:R00210 -- Acetyl-CoA --> rn:R10474 { Cinnamyl alcohol }

rn:R08206 <-- Acetate --> rn:R01241 -- Phenyl acetate --> rn:R07342 { Phenyl acetate }

rn:R08350 <-- Glutathione --> rn:R00120 -- Hydrogen peroxide --> rn:R01703 { Hexadecanoic acid }

rn:R08351 <-- Glutathione --> rn:R00120 -- Hydrogen peroxide --> rn:R01703 { Hexadecanoic acid }

rn:R08525 <-- Thyroxine --> rn:R03734 -- Acceptor --> rn:R02234 { Cyclohexanone }

rn:R08525 <-- Thyroxine --> rn:R03734 -- Acceptor --> rn:R03212 { 3-Hydroxycyclohexanone }

rn:R08551 <-- [Reduced NADPH---hemoprotein reductase] --> rn:R05487 -- 2-Hydroxyphenylacetate --> rn:R05001 { 2-Hydroxyphenylacetate }

rn:R08551 <-- [Reduced NADPH---hemoprotein reductase] --> rn:R05487 -- 2-Hydroxyphenylacetate --> rn:R05450 { 2-Hydroxyphenylacetate }

rn:R08663 <-- [Oxidized NADPH---hemoprotein reductase] --> rn:R08551 -- [Reduced NADPH---hemoprotein reductase] --> rn:R02468 { (-)-Limonene }

rn:R08663 <-- [Oxidized NADPH---hemoprotein reductase] --> rn:R08551 -- [Reduced NADPH---hemoprotein reductase] --> rn:R02469 { (-)-Limonene }

rn:R08663 <-- [Oxidized NADPH---hemoprotein reductase] --> rn:R08551 -- [Reduced NADPH---hemoprotein reductase] --> rn:R02470 { (-)-Limonene }

rn:R08663 <-- [Oxidized NADPH---hemoprotein reductase] --> rn:R08551 -- [Reduced NADPH---hemoprotein reductase] --> rn:R04366 { Linalool }

rn:R08663 <-- [Oxidized NADPH---hemoprotein reductase] --> rn:R08551 -- [Reduced NADPH---hemoprotein reductase] --> rn:R06119 { d-Limonene }

rn:R08663 <-- [Oxidized NADPH---hemoprotein reductase] --> rn:R08551 -- [Reduced NADPH---hemoprotein reductase] --> rn:R09451 { Hexadecanoic acid }

rn:R08663 <-- [Oxidized NADPH---hemoprotein reductase] --> rn:R08551 -- [Reduced NADPH---hemoprotein reductase] --> rn:R09452 { (9Z)-Octadecenoic acid }

rn:R08663 <-- [Oxidized NADPH---hemoprotein reductase] --> rn:R08551 -- [Reduced NADPH---hemoprotein reductase] --> rn:R09922 { (+)-Linalool }

rn:R08663 <-- [Oxidized NADPH---hemoprotein reductase] --> rn:R08551 -- [Reduced NADPH---hemoprotein reductase] --> rn:R09923 { (-)-Linalool }

rn:R08663 <-- [Oxidized NADPH---hemoprotein reductase] --> rn:R08551 -- [Reduced NADPH---hemoprotein reductase] --> rn:R09925 { Linalool }

rn:R08663 <-- [Oxidized NADPH---hemoprotein reductase] --> rn:R08551 -- [Reduced NADPH---hemoprotein reductase] --> rn:R09934 { Humulene }

rn:R08663 <-- [Oxidized NADPH---hemoprotein reductase] --> rn:R08551 -- [Reduced NADPH---hemoprotein reductase] --> rn:R10562 { (E,E)-Geranyllinalool }

rn:R08663 <-- [Oxidized NADPH---hemoprotein reductase] --> rn:R08551 -- [Reduced NADPH---hemoprotein reductase] --> rn:R11055 { Myrcene }

rn:R08743 <-- Chenodeoxycholate --> rn:R03978 -- FAD --> rn:R05537 { 3-(2-Hydroxyphenyl)propanoate }

rn:R08774 <-- 2-Polyprenyl-3-methyl-6-methoxy-1,4-benzoquinone --> rn:R08775 -- Acceptor --> rn:R02234 { Cyclohexanone }

rn:R08774 <-- 2-Polyprenyl-3-methyl-6-methoxy-1,4-benzoquinone --> rn:R08775 -- Acceptor --> rn:R03212 { 3-Hydroxycyclohexanone }

rn:R08861 <-- 2-Hydroxyethylphosphonate --> rn:R10191 -- HCO3- --> rn:R05453 { Acetophenone }

rn:R08874 <-- Acetate --> rn:R01241 -- Phenyl acetate --> rn:R07342 { Phenyl acetate }

rn:R08876 <-- Acetate --> rn:R01241 -- Phenyl acetate --> rn:R07342 { Phenyl acetate }

rn:R09155 <-- Chloroethane --> rn:R09156 -- Acceptor --> rn:R02234 { Cyclohexanone }

rn:R09155 <-- Chloroethane --> rn:R09156 -- Acceptor --> rn:R03212 { 3-Hydroxycyclohexanone }

rn:R09289 <-- 3-Oxopropanoate --> rn:R00706 -- Acetyl-CoA --> rn:R08530 { (-)-Menthol }

rn:R09289 <-- 3-Oxopropanoate --> rn:R00706 -- Acetyl-CoA --> rn:R08531 { (+)-Neomenthol }

rn:R09289 <-- 3-Oxopropanoate --> rn:R00706 -- Acetyl-CoA --> rn:R08532 { (+)-Borneol }

rn:R09289 <-- 3-Oxopropanoate --> rn:R00706 -- Acetyl-CoA --> rn:R10474 { Cinnamyl alcohol }

rn:R09403 <-- [Oxidized NADPH---hemoprotein reductase] --> rn:R08551 -- [Reduced NADPH---hemoprotein reductase] --> rn:R02468 { (-)-Limonene }

rn:R09403 <-- [Oxidized NADPH---hemoprotein reductase] --> rn:R08551 -- [Reduced NADPH---hemoprotein reductase] --> rn:R02469 { (-)-Limonene }

rn:R09403 <-- [Oxidized NADPH---hemoprotein reductase] --> rn:R08551 -- [Reduced NADPH---hemoprotein reductase] --> rn:R02470 { (-)-Limonene }

rn:R09403 <-- [Oxidized NADPH---hemoprotein reductase] --> rn:R08551 -- [Reduced NADPH---hemoprotein reductase] --> rn:R04366 { Linalool }

rn:R09403 <-- [Oxidized NADPH---hemoprotein reductase] --> rn:R08551 -- [Reduced NADPH---hemoprotein reductase] --> rn:R06119 { d-Limonene }

rn:R09403 <-- [Oxidized NADPH---hemoprotein reductase] --> rn:R08551 -- [Reduced NADPH---hemoprotein reductase] --> rn:R09451 { Hexadecanoic acid }

rn:R09403 <-- [Oxidized NADPH---hemoprotein reductase] --> rn:R08551 -- [Reduced NADPH---hemoprotein reductase] --> rn:R09452 { (9Z)-Octadecenoic acid }

rn:R09403 <-- [Oxidized NADPH---hemoprotein reductase] --> rn:R08551 -- [Reduced NADPH---hemoprotein reductase] --> rn:R09922 { (+)-Linalool }

rn:R09403 <-- [Oxidized NADPH---hemoprotein reductase] --> rn:R08551 -- [Reduced NADPH---hemoprotein reductase] --> rn:R09923 { (-)-Linalool }

rn:R09403 <-- [Oxidized NADPH---hemoprotein reductase] --> rn:R08551 -- [Reduced NADPH---hemoprotein reductase] --> rn:R09925 { Linalool }

rn:R09403 <-- [Oxidized NADPH---hemoprotein reductase] --> rn:R08551 -- [Reduced NADPH---hemoprotein reductase] --> rn:R09934 { Humulene }

rn:R09403 <-- [Oxidized NADPH---hemoprotein reductase] --> rn:R08551 -- [Reduced NADPH---hemoprotein reductase] --> rn:R10562 { (E,E)-Geranyllinalool }

rn:R09403 <-- [Oxidized NADPH---hemoprotein reductase] --> rn:R08551 -- [Reduced NADPH---hemoprotein reductase] --> rn:R11055 { Myrcene }

rn:R09493 <-- 2-Oxo acid --> rn:R04913 -- Amino acid --> rn:R11672 { (S)-3-Acetyloctanal }

rn:R09497 <-- Quinone --> rn:R11591 -- Pyruvate --> rn:R05136 { Salicylaldehyde }

rn:R09499 <-- Sulfur --> rn:R03533 -- Acceptor --> rn:R02234 { Cyclohexanone }

rn:R09499 <-- Sulfur --> rn:R03533 -- Acceptor --> rn:R03212 { 3-Hydroxycyclohexanone }

rn:R09517 <-- FAD --> rn:R02487 -- FADH2 --> rn:R05488 { Styrene }

rn:R09517 <-- 3-Hydroxyanthranilate --> rn:R02666 -- Hydrogen peroxide --> rn:R01703 { Hexadecanoic acid }

rn:R09517 <-- 3-Hydroxyanthranilate --> rn:R02670 -- Hydrogen peroxide --> rn:R01703 { Hexadecanoic acid }

rn:R09517 <-- FAD --> rn:R04095 -- FADH2 --> rn:R05488 { Styrene }

rn:R09517 <-- FAD --> rn:R05537 -- FADH2 --> rn:R05488 { Styrene }

rn:R09517 <-- FAD --> rn:R05537 -- Acetyl-CoA --> rn:R08530 { (-)-Menthol }

rn:R09517 <-- FAD --> rn:R05537 -- Acetyl-CoA --> rn:R08531 { (+)-Neomenthol }

rn:R09517 <-- FAD --> rn:R05537 -- Acetyl-CoA --> rn:R08532 { (+)-Borneol }

rn:R09517 <-- FAD --> rn:R05537 -- Acetyl-CoA --> rn:R10474 { Cinnamyl alcohol }

rn:R09517 <-- FAD --> rn:R06943 -- FADH2 --> rn:R05488 { Styrene }

rn:R09517 <-- FAD --> rn:R07220 -- FADH2 --> rn:R05488 { Styrene }

rn:R09517 <-- FAD --> rn:R09520 -- FADH2 --> rn:R05488 { Styrene }

rn:R09517 <-- FAD --> rn:R11130 -- FADH2 --> rn:R05488 { Styrene }

rn:R09518 <-- Quinone --> rn:R11591 -- Pyruvate --> rn:R05136 { Salicylaldehyde }

rn:R09520 <-- FADH2 --> rn:R03978 -- FAD --> rn:R05537 { 3-(2-Hydroxyphenyl)propanoate }

rn:R09520 <-- FADH2 --> rn:R05488 -- FAD --> rn:R05537 { 3-(2-Hydroxyphenyl)propanoate }

rn:R09520 <-- FADH2 --> rn:R09517 -- FAD --> rn:R05537 { 3-(2-Hydroxyphenyl)propanoate }

rn:R09520 <-- FADH2 --> rn:R11653 -- FAD --> rn:R05537 { 3-(2-Hydroxyphenyl)propanoate }

rn:R09520 <-- FADH2 --> rn:R12021 -- FAD --> rn:R05537 { 3-(2-Hydroxyphenyl)propanoate }

rn:R09520 <-- FADH2 --> rn:R12023 -- FAD --> rn:R05537 { 3-(2-Hydroxyphenyl)propanoate }

rn:R09520 <-- FADH2 --> rn:R12027 -- FAD --> rn:R05537 { 3-(2-Hydroxyphenyl)propanoate }

rn:R09520 <-- FADH2 --> rn:R12030 -- FAD --> rn:R05537 { 3-(2-Hydroxyphenyl)propanoate }

rn:R09536 <-- Arachidonate --> rn:R01599 -- Acceptor --> rn:R02234 { Cyclohexanone }

rn:R09536 <-- Arachidonate --> rn:R01599 -- Acceptor --> rn:R03212 { 3-Hydroxycyclohexanone }

rn:R09539 <-- Phenol --> rn:R01241 -- Phenyl acetate --> rn:R07342 { Phenyl acetate }

rn:R09539 <-- Phenol --> rn:R11653 -- FAD --> rn:R05537 { 3-(2-Hydroxyphenyl)propanoate }

rn:R09578 <-- [Oxidized NADPH---hemoprotein reductase] --> rn:R08551 -- [Reduced NADPH---hemoprotein reductase] --> rn:R02468 { (-)-Limonene }

rn:R09578 <-- [Oxidized NADPH---hemoprotein reductase] --> rn:R08551 -- [Reduced NADPH---hemoprotein reductase] --> rn:R02469 { (-)-Limonene }

rn:R09578 <-- [Oxidized NADPH---hemoprotein reductase] --> rn:R08551 -- [Reduced NADPH---hemoprotein reductase] --> rn:R02470 { (-)-Limonene }

rn:R09578 <-- [Oxidized NADPH---hemoprotein reductase] --> rn:R08551 -- [Reduced NADPH---hemoprotein reductase] --> rn:R04366 { Linalool }

rn:R09578 <-- [Oxidized NADPH---hemoprotein reductase] --> rn:R08551 -- [Reduced NADPH---hemoprotein reductase] --> rn:R06119 { d-Limonene }

rn:R09578 <-- [Oxidized NADPH---hemoprotein reductase] --> rn:R08551 -- [Reduced NADPH---hemoprotein reductase] --> rn:R09451 { Hexadecanoic acid }

rn:R09578 <-- [Oxidized NADPH---hemoprotein reductase] --> rn:R08551 -- [Reduced NADPH---hemoprotein reductase] --> rn:R09452 { (9Z)-Octadecenoic acid }

rn:R09578 <-- [Oxidized NADPH---hemoprotein reductase] --> rn:R08551 -- [Reduced NADPH---hemoprotein reductase] --> rn:R09922 { (+)-Linalool }

rn:R09578 <-- [Oxidized NADPH---hemoprotein reductase] --> rn:R08551 -- [Reduced NADPH---hemoprotein reductase] --> rn:R09923 { (-)-Linalool }

rn:R09578 <-- [Oxidized NADPH---hemoprotein reductase] --> rn:R08551 -- [Reduced NADPH---hemoprotein reductase] --> rn:R09925 { Linalool }

rn:R09578 <-- [Oxidized NADPH---hemoprotein reductase] --> rn:R08551 -- [Reduced NADPH---hemoprotein reductase] --> rn:R09934 { Humulene }

rn:R09578 <-- [Oxidized NADPH---hemoprotein reductase] --> rn:R08551 -- [Reduced NADPH---hemoprotein reductase] --> rn:R10562 { (E,E)-Geranyllinalool }

rn:R09578 <-- [Oxidized NADPH---hemoprotein reductase] --> rn:R08551 -- [Reduced NADPH---hemoprotein reductase] --> rn:R11055 { Myrcene }

rn:R09579 <-- [Oxidized NADPH---hemoprotein reductase] --> rn:R08551 -- [Reduced NADPH---hemoprotein reductase] --> rn:R02468 { (-)-Limonene }

rn:R09579 <-- [Oxidized NADPH---hemoprotein reductase] --> rn:R08551 -- [Reduced NADPH---hemoprotein reductase] --> rn:R02469 { (-)-Limonene }

rn:R09579 <-- [Oxidized NADPH---hemoprotein reductase] --> rn:R08551 -- [Reduced NADPH---hemoprotein reductase] --> rn:R02470 { (-)-Limonene }

rn:R09579 <-- [Oxidized NADPH---hemoprotein reductase] --> rn:R08551 -- [Reduced NADPH---hemoprotein reductase] --> rn:R04366 { Linalool }

rn:R09579 <-- [Oxidized NADPH---hemoprotein reductase] --> rn:R08551 -- [Reduced NADPH---hemoprotein reductase] --> rn:R06119 { d-Limonene }

rn:R09579 <-- [Oxidized NADPH---hemoprotein reductase] --> rn:R08551 -- [Reduced NADPH---hemoprotein reductase] --> rn:R09451 { Hexadecanoic acid }

rn:R09579 <-- [Oxidized NADPH---hemoprotein reductase] --> rn:R08551 -- [Reduced NADPH---hemoprotein reductase] --> rn:R09452 { (9Z)-Octadecenoic acid }

rn:R09579 <-- [Oxidized NADPH---hemoprotein reductase] --> rn:R08551 -- [Reduced NADPH---hemoprotein reductase] --> rn:R09922 { (+)-Linalool }

rn:R09579 <-- [Oxidized NADPH---hemoprotein reductase] --> rn:R08551 -- [Reduced NADPH---hemoprotein reductase] --> rn:R09923 { (-)-Linalool }

rn:R09579 <-- [Oxidized NADPH---hemoprotein reductase] --> rn:R08551 -- [Reduced NADPH---hemoprotein reductase] --> rn:R09925 { Linalool }

rn:R09579 <-- [Oxidized NADPH---hemoprotein reductase] --> rn:R08551 -- [Reduced NADPH---hemoprotein reductase] --> rn:R09934 { Humulene }

rn:R09579 <-- [Oxidized NADPH---hemoprotein reductase] --> rn:R08551 -- [Reduced NADPH---hemoprotein reductase] --> rn:R10562 { (E,E)-Geranyllinalool }

rn:R09579 <-- [Oxidized NADPH---hemoprotein reductase] --> rn:R08551 -- [Reduced NADPH---hemoprotein reductase] --> rn:R11055 { Myrcene }

rn:R09580 <-- [Oxidized NADPH---hemoprotein reductase] --> rn:R08551 -- [Reduced NADPH---hemoprotein reductase] --> rn:R02468 { (-)-Limonene }

rn:R09580 <-- [Oxidized NADPH---hemoprotein reductase] --> rn:R08551 -- [Reduced NADPH---hemoprotein reductase] --> rn:R02469 { (-)-Limonene }

rn:R09580 <-- [Oxidized NADPH---hemoprotein reductase] --> rn:R08551 -- [Reduced NADPH---hemoprotein reductase] --> rn:R02470 { (-)-Limonene }

rn:R09580 <-- [Oxidized NADPH---hemoprotein reductase] --> rn:R08551 -- [Reduced NADPH---hemoprotein reductase] --> rn:R04366 { Linalool }

rn:R09580 <-- [Oxidized NADPH---hemoprotein reductase] --> rn:R08551 -- [Reduced NADPH---hemoprotein reductase] --> rn:R06119 { d-Limonene }

rn:R09580 <-- [Oxidized NADPH---hemoprotein reductase] --> rn:R08551 -- [Reduced NADPH---hemoprotein reductase] --> rn:R09451 { Hexadecanoic acid }

rn:R09580 <-- [Oxidized NADPH---hemoprotein reductase] --> rn:R08551 -- [Reduced NADPH---hemoprotein reductase] --> rn:R09452 { (9Z)-Octadecenoic acid }

rn:R09580 <-- [Oxidized NADPH---hemoprotein reductase] --> rn:R08551 -- [Reduced NADPH---hemoprotein reductase] --> rn:R09922 { (+)-Linalool }

rn:R09580 <-- [Oxidized NADPH---hemoprotein reductase] --> rn:R08551 -- [Reduced NADPH---hemoprotein reductase] --> rn:R09923 { (-)-Linalool }

rn:R09580 <-- [Oxidized NADPH---hemoprotein reductase] --> rn:R08551 -- [Reduced NADPH---hemoprotein reductase] --> rn:R09925 { Linalool }

rn:R09580 <-- [Oxidized NADPH---hemoprotein reductase] --> rn:R08551 -- [Reduced NADPH---hemoprotein reductase] --> rn:R09934 { Humulene }

rn:R09580 <-- [Oxidized NADPH---hemoprotein reductase] --> rn:R08551 -- [Reduced NADPH---hemoprotein reductase] --> rn:R10562 { (E,E)-Geranyllinalool }

rn:R09580 <-- [Oxidized NADPH---hemoprotein reductase] --> rn:R08551 -- [Reduced NADPH---hemoprotein reductase] --> rn:R11055 { Myrcene }

rn:R09598 <-- L-Glutamate --> rn:R00114 -- L-Glutamine --> rn:R02781 { 2,4,6/3,5-Pentahydroxycyclohexanone }

rn:R09599 <-- L-Glutamate --> rn:R00114 -- L-Glutamine --> rn:R02781 { 2,4,6/3,5-Pentahydroxycyclohexanone }

rn:R09820 <-- 3-Oxo-5,6-dehydrosuberyl-CoA --> rn:R09839 -- Acetyl-CoA --> rn:R08530 { (-)-Menthol }

rn:R09820 <-- 3-Oxo-5,6-dehydrosuberyl-CoA --> rn:R09839 -- Acetyl-CoA --> rn:R08531 { (+)-Neomenthol }

rn:R09820 <-- 3-Oxo-5,6-dehydrosuberyl-CoA --> rn:R09839 -- Acetyl-CoA --> rn:R08532 { (+)-Borneol }

rn:R09820 <-- 3-Oxo-5,6-dehydrosuberyl-CoA --> rn:R09839 -- Acetyl-CoA --> rn:R10474 { Cinnamyl alcohol }

rn:R09824 <-- L-Glutamate --> rn:R00114 -- L-Glutamine --> rn:R02781 { 2,4,6/3,5-Pentahydroxycyclohexanone }

rn:R09840 <-- Phenylacetic acid --> rn:R05487 -- 2-Hydroxyphenylacetate --> rn:R05001 { 2-Hydroxyphenylacetate }

rn:R09840 <-- Phenylacetic acid --> rn:R05487 -- 2-Hydroxyphenylacetate --> rn:R05450 { 2-Hydroxyphenylacetate }

rn:R10027 <-- [Oxidized NADPH---hemoprotein reductase] --> rn:R08551 -- [Reduced NADPH---hemoprotein reductase] --> rn:R02468 { (-)-Limonene }

rn:R10027 <-- [Oxidized NADPH---hemoprotein reductase] --> rn:R08551 -- [Reduced NADPH---hemoprotein reductase] --> rn:R02469 { (-)-Limonene }

rn:R10027 <-- [Oxidized NADPH---hemoprotein reductase] --> rn:R08551 -- [Reduced NADPH---hemoprotein reductase] --> rn:R02470 { (-)-Limonene }

rn:R10027 <-- [Oxidized NADPH---hemoprotein reductase] --> rn:R08551 -- [Reduced NADPH---hemoprotein reductase] --> rn:R04366 { Linalool }

rn:R10027 <-- [Oxidized NADPH---hemoprotein reductase] --> rn:R08551 -- [Reduced NADPH---hemoprotein reductase] --> rn:R06119 { d-Limonene }

rn:R10027 <-- [Oxidized NADPH---hemoprotein reductase] --> rn:R08551 -- [Reduced NADPH---hemoprotein reductase] --> rn:R09451 { Hexadecanoic acid }

rn:R10027 <-- [Oxidized NADPH---hemoprotein reductase] --> rn:R08551 -- [Reduced NADPH---hemoprotein reductase] --> rn:R09452 { (9Z)-Octadecenoic acid }

rn:R10027 <-- [Oxidized NADPH---hemoprotein reductase] --> rn:R08551 -- [Reduced NADPH---hemoprotein reductase] --> rn:R09922 { (+)-Linalool }

rn:R10027 <-- [Oxidized NADPH---hemoprotein reductase] --> rn:R08551 -- [Reduced NADPH---hemoprotein reductase] --> rn:R09923 { (-)-Linalool }

rn:R10027 <-- [Oxidized NADPH---hemoprotein reductase] --> rn:R08551 -- [Reduced NADPH---hemoprotein reductase] --> rn:R09925 { Linalool }

rn:R10027 <-- [Oxidized NADPH---hemoprotein reductase] --> rn:R08551 -- [Reduced NADPH---hemoprotein reductase] --> rn:R09934 { Humulene }

rn:R10027 <-- [Oxidized NADPH---hemoprotein reductase] --> rn:R08551 -- [Reduced NADPH---hemoprotein reductase] --> rn:R10562 { (E,E)-Geranyllinalool }

rn:R10027 <-- [Oxidized NADPH---hemoprotein reductase] --> rn:R08551 -- [Reduced NADPH---hemoprotein reductase] --> rn:R11055 { Myrcene }

rn:R10028 <-- [Oxidized NADPH---hemoprotein reductase] --> rn:R08551 -- [Reduced NADPH---hemoprotein reductase] --> rn:R02468 { (-)-Limonene }

rn:R10028 <-- [Oxidized NADPH---hemoprotein reductase] --> rn:R08551 -- [Reduced NADPH---hemoprotein reductase] --> rn:R02469 { (-)-Limonene }

rn:R10028 <-- [Oxidized NADPH---hemoprotein reductase] --> rn:R08551 -- [Reduced NADPH---hemoprotein reductase] --> rn:R02470 { (-)-Limonene }

rn:R10028 <-- [Oxidized NADPH---hemoprotein reductase] --> rn:R08551 -- [Reduced NADPH---hemoprotein reductase] --> rn:R04366 { Linalool }

rn:R10028 <-- [Oxidized NADPH---hemoprotein reductase] --> rn:R08551 -- [Reduced NADPH---hemoprotein reductase] --> rn:R06119 { d-Limonene }

rn:R10028 <-- [Oxidized NADPH---hemoprotein reductase] --> rn:R08551 -- [Reduced NADPH---hemoprotein reductase] --> rn:R09451 { Hexadecanoic acid }

rn:R10028 <-- [Oxidized NADPH---hemoprotein reductase] --> rn:R08551 -- [Reduced NADPH---hemoprotein reductase] --> rn:R09452 { (9Z)-Octadecenoic acid }

rn:R10028 <-- [Oxidized NADPH---hemoprotein reductase] --> rn:R08551 -- [Reduced NADPH---hemoprotein reductase] --> rn:R09922 { (+)-Linalool }

rn:R10028 <-- [Oxidized NADPH---hemoprotein reductase] --> rn:R08551 -- [Reduced NADPH---hemoprotein reductase] --> rn:R09923 { (-)-Linalool }

rn:R10028 <-- [Oxidized NADPH---hemoprotein reductase] --> rn:R08551 -- [Reduced NADPH---hemoprotein reductase] --> rn:R09925 { Linalool }

rn:R10028 <-- [Oxidized NADPH---hemoprotein reductase] --> rn:R08551 -- [Reduced NADPH---hemoprotein reductase] --> rn:R09934 { Humulene }

rn:R10028 <-- [Oxidized NADPH---hemoprotein reductase] --> rn:R08551 -- [Reduced NADPH---hemoprotein reductase] --> rn:R10562 { (E,E)-Geranyllinalool }

rn:R10028 <-- [Oxidized NADPH---hemoprotein reductase] --> rn:R08551 -- [Reduced NADPH---hemoprotein reductase] --> rn:R11055 { Myrcene }

rn:R10031 <-- [Oxidized NADPH---hemoprotein reductase] --> rn:R08551 -- [Reduced NADPH---hemoprotein reductase] --> rn:R02468 { (-)-Limonene }

rn:R10031 <-- [Oxidized NADPH---hemoprotein reductase] --> rn:R08551 -- [Reduced NADPH---hemoprotein reductase] --> rn:R02469 { (-)-Limonene }

rn:R10031 <-- [Oxidized NADPH---hemoprotein reductase] --> rn:R08551 -- [Reduced NADPH---hemoprotein reductase] --> rn:R02470 { (-)-Limonene }

rn:R10031 <-- [Oxidized NADPH---hemoprotein reductase] --> rn:R08551 -- [Reduced NADPH---hemoprotein reductase] --> rn:R04366 { Linalool }

rn:R10031 <-- [Oxidized NADPH---hemoprotein reductase] --> rn:R08551 -- [Reduced NADPH---hemoprotein reductase] --> rn:R06119 { d-Limonene }

rn:R10031 <-- [Oxidized NADPH---hemoprotein reductase] --> rn:R08551 -- [Reduced NADPH---hemoprotein reductase] --> rn:R09451 { Hexadecanoic acid }

rn:R10031 <-- [Oxidized NADPH---hemoprotein reductase] --> rn:R08551 -- [Reduced NADPH---hemoprotein reductase] --> rn:R09452 { (9Z)-Octadecenoic acid }

rn:R10031 <-- [Oxidized NADPH---hemoprotein reductase] --> rn:R08551 -- [Reduced NADPH---hemoprotein reductase] --> rn:R09922 { (+)-Linalool }

rn:R10031 <-- [Oxidized NADPH---hemoprotein reductase] --> rn:R08551 -- [Reduced NADPH---hemoprotein reductase] --> rn:R09923 { (-)-Linalool }

rn:R10031 <-- [Oxidized NADPH---hemoprotein reductase] --> rn:R08551 -- [Reduced NADPH---hemoprotein reductase] --> rn:R09925 { Linalool }

rn:R10031 <-- [Oxidized NADPH---hemoprotein reductase] --> rn:R08551 -- [Reduced NADPH---hemoprotein reductase] --> rn:R09934 { Humulene }

rn:R10031 <-- [Oxidized NADPH---hemoprotein reductase] --> rn:R08551 -- [Reduced NADPH---hemoprotein reductase] --> rn:R10562 { (E,E)-Geranyllinalool }

rn:R10031 <-- [Oxidized NADPH---hemoprotein reductase] --> rn:R08551 -- [Reduced NADPH---hemoprotein reductase] --> rn:R11055 { Myrcene }

rn:R10032 <-- [Oxidized NADPH---hemoprotein reductase] --> rn:R08551 -- [Reduced NADPH---hemoprotein reductase] --> rn:R02468 { (-)-Limonene }

rn:R10032 <-- [Oxidized NADPH---hemoprotein reductase] --> rn:R08551 -- [Reduced NADPH---hemoprotein reductase] --> rn:R02469 { (-)-Limonene }

rn:R10032 <-- [Oxidized NADPH---hemoprotein reductase] --> rn:R08551 -- [Reduced NADPH---hemoprotein reductase] --> rn:R02470 { (-)-Limonene }

rn:R10032 <-- [Oxidized NADPH---hemoprotein reductase] --> rn:R08551 -- [Reduced NADPH---hemoprotein reductase] --> rn:R04366 { Linalool }

rn:R10032 <-- [Oxidized NADPH---hemoprotein reductase] --> rn:R08551 -- [Reduced NADPH---hemoprotein reductase] --> rn:R06119 { d-Limonene }

rn:R10032 <-- [Oxidized NADPH---hemoprotein reductase] --> rn:R08551 -- [Reduced NADPH---hemoprotein reductase] --> rn:R09451 { Hexadecanoic acid }

rn:R10032 <-- [Oxidized NADPH---hemoprotein reductase] --> rn:R08551 -- [Reduced NADPH---hemoprotein reductase] --> rn:R09452 { (9Z)-Octadecenoic acid }

rn:R10032 <-- [Oxidized NADPH---hemoprotein reductase] --> rn:R08551 -- [Reduced NADPH---hemoprotein reductase] --> rn:R09922 { (+)-Linalool }

rn:R10032 <-- [Oxidized NADPH---hemoprotein reductase] --> rn:R08551 -- [Reduced NADPH---hemoprotein reductase] --> rn:R09923 { (-)-Linalool }

rn:R10032 <-- [Oxidized NADPH---hemoprotein reductase] --> rn:R08551 -- [Reduced NADPH---hemoprotein reductase] --> rn:R09925 { Linalool }

rn:R10032 <-- [Oxidized NADPH---hemoprotein reductase] --> rn:R08551 -- [Reduced NADPH---hemoprotein reductase] --> rn:R09934 { Humulene }

rn:R10032 <-- [Oxidized NADPH---hemoprotein reductase] --> rn:R08551 -- [Reduced NADPH---hemoprotein reductase] --> rn:R10562 { (E,E)-Geranyllinalool }

rn:R10032 <-- [Oxidized NADPH---hemoprotein reductase] --> rn:R08551 -- [Reduced NADPH---hemoprotein reductase] --> rn:R11055 { Myrcene }

rn:R10042 <-- Phenol --> rn:R01241 -- Phenyl acetate --> rn:R07342 { Phenyl acetate }

rn:R10042 <-- Phenol --> rn:R11653 -- FAD --> rn:R05537 { 3-(2-Hydroxyphenyl)propanoate }

rn:R10135 <-- Maleylpyruvate --> rn:R11257 -- Pyruvate --> rn:R05136 { Salicylaldehyde }

rn:R10186 <-- alpha-D-Ribose 1-methylphosphonate 5-phosphate --> rn:R10204 -- Acceptor --> rn:R02234 { Cyclohexanone }

rn:R10186 <-- alpha-D-Ribose 1-methylphosphonate 5-phosphate --> rn:R10204 -- Acceptor --> rn:R03212 { 3-Hydroxycyclohexanone }

rn:R10248 <-- Acetate --> rn:R01241 -- Phenyl acetate --> rn:R07342 { Phenyl acetate }

rn:R10249 <-- Acetate --> rn:R01241 -- Phenyl acetate --> rn:R07342 { Phenyl acetate }

rn:R10289 <-- Acetate --> rn:R01241 -- Phenyl acetate --> rn:R07342 { Phenyl acetate }

rn:R10419 <-- Sulfur --> rn:R03533 -- Acceptor --> rn:R02234 { Cyclohexanone }

rn:R10419 <-- Sulfur --> rn:R03533 -- Acceptor --> rn:R03212 { 3-Hydroxycyclohexanone }

rn:R10438 <-- Sulfur --> rn:R03533 -- Acceptor --> rn:R02234 { Cyclohexanone }

rn:R10438 <-- Sulfur --> rn:R03533 -- Acceptor --> rn:R03212 { 3-Hydroxycyclohexanone }

rn:R10499 <-- Phenylpyruvate --> rn:R00695 -- L-Phenylalanine --> rn:R11068 { 3-[(1R,2S,5R,6S)-5-Hydroxy-7-oxabicyclo[4.1.0]heptan-2-yl]-2-oxopropanoate }

rn:R10499 <-- Phenylpyruvate --> rn:R01372 -- 2-Hydroxyphenylacetate --> rn:R05001 { 2-Hydroxyphenylacetate }

rn:R10499 <-- Phenylpyruvate --> rn:R01372 -- 2-Hydroxyphenylacetate --> rn:R05450 { 2-Hydroxyphenylacetate }

rn:R10499 <-- Phenylpyruvate --> rn:R01375 -- L-Phenylalanine --> rn:R11068 { 3-[(1R,2S,5R,6S)-5-Hydroxy-7-oxabicyclo[4.1.0]heptan-2-yl]-2-oxopropanoate }

rn:R10499 <-- Phenylpyruvate --> rn:R01376 -- L-Phenylalanine --> rn:R11068 { 3-[(1R,2S,5R,6S)-5-Hydroxy-7-oxabicyclo[4.1.0]heptan-2-yl]-2-oxopropanoate }

rn:R10503 <-- 2-Oxo acid --> rn:R04913 -- Amino acid --> rn:R11672 { (S)-3-Acetyloctanal }

rn:R10528 <-- 2-Oxo acid --> rn:R04913 -- Amino acid --> rn:R11672 { (S)-3-Acetyloctanal }

rn:R10553 <-- Acetate --> rn:R01241 -- Phenyl acetate --> rn:R07342 { Phenyl acetate }

rn:R10572 <-- Quinone --> rn:R11591 -- Pyruvate --> rn:R05136 { Salicylaldehyde }

rn:R10583 <-- Pyruvate --> rn:R00210 -- Acetyl-CoA --> rn:R08530 { (-)-Menthol }

rn:R10583 <-- Pyruvate --> rn:R00210 -- Acetyl-CoA --> rn:R08531 { (+)-Neomenthol }

rn:R10583 <-- Pyruvate --> rn:R00210 -- Acetyl-CoA --> rn:R08532 { (+)-Borneol }

rn:R10583 <-- Pyruvate --> rn:R00210 -- Acetyl-CoA --> rn:R10474 { Cinnamyl alcohol }

rn:R10600 <-- Acetate --> rn:R01241 -- Phenyl acetate --> rn:R07342 { Phenyl acetate }

rn:R10625 <-- 2-Oxo acid --> rn:R04913 -- Amino acid --> rn:R11672 { (S)-3-Acetyloctanal }

rn:R10637 <-- Mandelonitrile --> rn:R01767 -- Benzaldehyde --> rn:R01419 { Benzaldehyde }

rn:R10637 <-- Mandelonitrile --> rn:R01767 -- Benzaldehyde --> rn:R01420 { Benzaldehyde }

rn:R10637 <-- Mandelonitrile --> rn:R11380 -- Hydrogen peroxide --> rn:R01703 { Hexadecanoic acid }

rn:R10666 <-- 3-[(1-Carboxyvinyl)oxy]benzoate --> rn:R10667 -- HCO3- --> rn:R05453 { Acetophenone }

rn:R10671 <-- [Oxidized NADPH---hemoprotein reductase] --> rn:R08551 -- [Reduced NADPH---hemoprotein reductase] --> rn:R02468 { (-)-Limonene }

rn:R10671 <-- [Oxidized NADPH---hemoprotein reductase] --> rn:R08551 -- [Reduced NADPH---hemoprotein reductase] --> rn:R02469 { (-)-Limonene }

rn:R10671 <-- [Oxidized NADPH---hemoprotein reductase] --> rn:R08551 -- [Reduced NADPH---hemoprotein reductase] --> rn:R02470 { (-)-Limonene }

rn:R10671 <-- [Oxidized NADPH---hemoprotein reductase] --> rn:R08551 -- [Reduced NADPH---hemoprotein reductase] --> rn:R04366 { Linalool }

rn:R10671 <-- [Oxidized NADPH---hemoprotein reductase] --> rn:R08551 -- [Reduced NADPH---hemoprotein reductase] --> rn:R06119 { d-Limonene }

rn:R10671 <-- [Oxidized NADPH---hemoprotein reductase] --> rn:R08551 -- [Reduced NADPH---hemoprotein reductase] --> rn:R09451 { Hexadecanoic acid }

rn:R10671 <-- [Oxidized NADPH---hemoprotein reductase] --> rn:R08551 -- [Reduced NADPH---hemoprotein reductase] --> rn:R09452 { (9Z)-Octadecenoic acid }

rn:R10671 <-- [Oxidized NADPH---hemoprotein reductase] --> rn:R08551 -- [Reduced NADPH---hemoprotein reductase] --> rn:R09922 { (+)-Linalool }

rn:R10671 <-- [Oxidized NADPH---hemoprotein reductase] --> rn:R08551 -- [Reduced NADPH---hemoprotein reductase] --> rn:R09923 { (-)-Linalool }

rn:R10671 <-- [Oxidized NADPH---hemoprotein reductase] --> rn:R08551 -- [Reduced NADPH---hemoprotein reductase] --> rn:R09925 { Linalool }

rn:R10671 <-- [Oxidized NADPH---hemoprotein reductase] --> rn:R08551 -- [Reduced NADPH---hemoprotein reductase] --> rn:R09934 { Humulene }

rn:R10671 <-- [Oxidized NADPH---hemoprotein reductase] --> rn:R08551 -- [Reduced NADPH---hemoprotein reductase] --> rn:R10562 { (E,E)-Geranyllinalool }

rn:R10671 <-- [Oxidized NADPH---hemoprotein reductase] --> rn:R08551 -- [Reduced NADPH---hemoprotein reductase] --> rn:R11055 { Myrcene }

rn:R10722 <-- Glycine --> rn:R08701 -- Acceptor --> rn:R02234 { Cyclohexanone }

rn:R10722 <-- Glycine --> rn:R08701 -- Acceptor --> rn:R03212 { 3-Hydroxycyclohexanone }

rn:R10728 <-- [Oxidized NADPH---hemoprotein reductase] --> rn:R08551 -- [Reduced NADPH---hemoprotein reductase] --> rn:R02468 { (-)-Limonene }

rn:R10728 <-- [Oxidized NADPH---hemoprotein reductase] --> rn:R08551 -- [Reduced NADPH---hemoprotein reductase] --> rn:R02469 { (-)-Limonene }

rn:R10728 <-- [Oxidized NADPH---hemoprotein reductase] --> rn:R08551 -- [Reduced NADPH---hemoprotein reductase] --> rn:R02470 { (-)-Limonene }

rn:R10728 <-- [Oxidized NADPH---hemoprotein reductase] --> rn:R08551 -- [Reduced NADPH---hemoprotein reductase] --> rn:R04366 { Linalool }

rn:R10728 <-- [Oxidized NADPH---hemoprotein reductase] --> rn:R08551 -- [Reduced NADPH---hemoprotein reductase] --> rn:R06119 { d-Limonene }

rn:R10728 <-- [Oxidized NADPH---hemoprotein reductase] --> rn:R08551 -- [Reduced NADPH---hemoprotein reductase] --> rn:R09451 { Hexadecanoic acid }

rn:R10728 <-- [Oxidized NADPH---hemoprotein reductase] --> rn:R08551 -- [Reduced NADPH---hemoprotein reductase] --> rn:R09452 { (9Z)-Octadecenoic acid }

rn:R10728 <-- [Oxidized NADPH---hemoprotein reductase] --> rn:R08551 -- [Reduced NADPH---hemoprotein reductase] --> rn:R09922 { (+)-Linalool }

rn:R10728 <-- [Oxidized NADPH---hemoprotein reductase] --> rn:R08551 -- [Reduced NADPH---hemoprotein reductase] --> rn:R09923 { (-)-Linalool }

rn:R10728 <-- [Oxidized NADPH---hemoprotein reductase] --> rn:R08551 -- [Reduced NADPH---hemoprotein reductase] --> rn:R09925 { Linalool }

rn:R10728 <-- [Oxidized NADPH---hemoprotein reductase] --> rn:R08551 -- [Reduced NADPH---hemoprotein reductase] --> rn:R09934 { Humulene }

rn:R10728 <-- [Oxidized NADPH---hemoprotein reductase] --> rn:R08551 -- [Reduced NADPH---hemoprotein reductase] --> rn:R10562 { (E,E)-Geranyllinalool }

rn:R10728 <-- [Oxidized NADPH---hemoprotein reductase] --> rn:R08551 -- [Reduced NADPH---hemoprotein reductase] --> rn:R11055 { Myrcene }

rn:R10786 <-- 2-Oxo acid --> rn:R04913 -- Amino acid --> rn:R11672 { (S)-3-Acetyloctanal }

rn:R10795 <-- [Oxidized NADPH---hemoprotein reductase] --> rn:R08551 -- [Reduced NADPH---hemoprotein reductase] --> rn:R02468 { (-)-Limonene }

rn:R10795 <-- [Oxidized NADPH---hemoprotein reductase] --> rn:R08551 -- [Reduced NADPH---hemoprotein reductase] --> rn:R02469 { (-)-Limonene }

rn:R10795 <-- [Oxidized NADPH---hemoprotein reductase] --> rn:R08551 -- [Reduced NADPH---hemoprotein reductase] --> rn:R02470 { (-)-Limonene }

rn:R10795 <-- [Oxidized NADPH---hemoprotein reductase] --> rn:R08551 -- [Reduced NADPH---hemoprotein reductase] --> rn:R04366 { Linalool }

rn:R10795 <-- [Oxidized NADPH---hemoprotein reductase] --> rn:R08551 -- [Reduced NADPH---hemoprotein reductase] --> rn:R06119 { d-Limonene }

rn:R10795 <-- [Oxidized NADPH---hemoprotein reductase] --> rn:R08551 -- [Reduced NADPH---hemoprotein reductase] --> rn:R09451 { Hexadecanoic acid }

rn:R10795 <-- [Oxidized NADPH---hemoprotein reductase] --> rn:R08551 -- [Reduced NADPH---hemoprotein reductase] --> rn:R09452 { (9Z)-Octadecenoic acid }

rn:R10795 <-- [Oxidized NADPH---hemoprotein reductase] --> rn:R08551 -- [Reduced NADPH---hemoprotein reductase] --> rn:R09922 { (+)-Linalool }

rn:R10795 <-- [Oxidized NADPH---hemoprotein reductase] --> rn:R08551 -- [Reduced NADPH---hemoprotein reductase] --> rn:R09923 { (-)-Linalool }

rn:R10795 <-- [Oxidized NADPH---hemoprotein reductase] --> rn:R08551 -- [Reduced NADPH---hemoprotein reductase] --> rn:R09925 { Linalool }

rn:R10795 <-- [Oxidized NADPH---hemoprotein reductase] --> rn:R08551 -- [Reduced NADPH---hemoprotein reductase] --> rn:R09934 { Humulene }

rn:R10795 <-- [Oxidized NADPH---hemoprotein reductase] --> rn:R08551 -- [Reduced NADPH---hemoprotein reductase] --> rn:R10562 { (E,E)-Geranyllinalool }

rn:R10795 <-- [Oxidized NADPH---hemoprotein reductase] --> rn:R08551 -- [Reduced NADPH---hemoprotein reductase] --> rn:R11055 { Myrcene }

rn:R10820 <-- 3-(Methylthio)propanoyl-CoA --> rn:R11130 -- FADH2 --> rn:R05488 { Styrene }

rn:R10999 <-- [Oxidized NADPH---hemoprotein reductase] --> rn:R08551 -- [Reduced NADPH---hemoprotein reductase] --> rn:R02468 { (-)-Limonene }

rn:R10999 <-- [Oxidized NADPH---hemoprotein reductase] --> rn:R08551 -- [Reduced NADPH---hemoprotein reductase] --> rn:R02469 { (-)-Limonene }

rn:R10999 <-- [Oxidized NADPH---hemoprotein reductase] --> rn:R08551 -- [Reduced NADPH---hemoprotein reductase] --> rn:R02470 { (-)-Limonene }

rn:R10999 <-- [Oxidized NADPH---hemoprotein reductase] --> rn:R08551 -- [Reduced NADPH---hemoprotein reductase] --> rn:R04366 { Linalool }

rn:R10999 <-- [Oxidized NADPH---hemoprotein reductase] --> rn:R08551 -- [Reduced NADPH---hemoprotein reductase] --> rn:R06119 { d-Limonene }

rn:R10999 <-- [Oxidized NADPH---hemoprotein reductase] --> rn:R08551 -- [Reduced NADPH---hemoprotein reductase] --> rn:R09451 { Hexadecanoic acid }

rn:R10999 <-- [Oxidized NADPH---hemoprotein reductase] --> rn:R08551 -- [Reduced NADPH---hemoprotein reductase] --> rn:R09452 { (9Z)-Octadecenoic acid }

rn:R10999 <-- [Oxidized NADPH---hemoprotein reductase] --> rn:R08551 -- [Reduced NADPH---hemoprotein reductase] --> rn:R09922 { (+)-Linalool }

rn:R10999 <-- [Oxidized NADPH---hemoprotein reductase] --> rn:R08551 -- [Reduced NADPH---hemoprotein reductase] --> rn:R09923 { (-)-Linalool }

rn:R10999 <-- [Oxidized NADPH---hemoprotein reductase] --> rn:R08551 -- [Reduced NADPH---hemoprotein reductase] --> rn:R09925 { Linalool }

rn:R10999 <-- [Oxidized NADPH---hemoprotein reductase] --> rn:R08551 -- [Reduced NADPH---hemoprotein reductase] --> rn:R09934 { Humulene }

rn:R10999 <-- [Oxidized NADPH---hemoprotein reductase] --> rn:R08551 -- [Reduced NADPH---hemoprotein reductase] --> rn:R10562 { (E,E)-Geranyllinalool }

rn:R10999 <-- [Oxidized NADPH---hemoprotein reductase] --> rn:R08551 -- [Reduced NADPH---hemoprotein reductase] --> rn:R11055 { Myrcene }

rn:R11000 <-- [Oxidized NADPH---hemoprotein reductase] --> rn:R08551 -- [Reduced NADPH---hemoprotein reductase] --> rn:R02468 { (-)-Limonene }

rn:R11000 <-- [Oxidized NADPH---hemoprotein reductase] --> rn:R08551 -- [Reduced NADPH---hemoprotein reductase] --> rn:R02469 { (-)-Limonene }

rn:R11000 <-- [Oxidized NADPH---hemoprotein reductase] --> rn:R08551 -- [Reduced NADPH---hemoprotein reductase] --> rn:R02470 { (-)-Limonene }

rn:R11000 <-- [Oxidized NADPH---hemoprotein reductase] --> rn:R08551 -- [Reduced NADPH---hemoprotein reductase] --> rn:R04366 { Linalool }

rn:R11000 <-- [Oxidized NADPH---hemoprotein reductase] --> rn:R08551 -- [Reduced NADPH---hemoprotein reductase] --> rn:R06119 { d-Limonene }

rn:R11000 <-- [Oxidized NADPH---hemoprotein reductase] --> rn:R08551 -- [Reduced NADPH---hemoprotein reductase] --> rn:R09451 { Hexadecanoic acid }

rn:R11000 <-- [Oxidized NADPH---hemoprotein reductase] --> rn:R08551 -- [Reduced NADPH---hemoprotein reductase] --> rn:R09452 { (9Z)-Octadecenoic acid }

rn:R11000 <-- [Oxidized NADPH---hemoprotein reductase] --> rn:R08551 -- [Reduced NADPH---hemoprotein reductase] --> rn:R09922 { (+)-Linalool }

rn:R11000 <-- [Oxidized NADPH---hemoprotein reductase] --> rn:R08551 -- [Reduced NADPH---hemoprotein reductase] --> rn:R09923 { (-)-Linalool }

rn:R11000 <-- [Oxidized NADPH---hemoprotein reductase] --> rn:R08551 -- [Reduced NADPH---hemoprotein reductase] --> rn:R09925 { Linalool }

rn:R11000 <-- [Oxidized NADPH---hemoprotein reductase] --> rn:R08551 -- [Reduced NADPH---hemoprotein reductase] --> rn:R09934 { Humulene }

rn:R11000 <-- [Oxidized NADPH---hemoprotein reductase] --> rn:R08551 -- [Reduced NADPH---hemoprotein reductase] --> rn:R10562 { (E,E)-Geranyllinalool }

rn:R11000 <-- [Oxidized NADPH---hemoprotein reductase] --> rn:R08551 -- [Reduced NADPH---hemoprotein reductase] --> rn:R11055 { Myrcene }

rn:R11014 <-- S-(Hercyn-2-yl)-L-cysteine S-oxide --> rn:R11864 -- Pyruvate --> rn:R05136 { Salicylaldehyde }

rn:R11021 <-- L-Glutamate --> rn:R00114 -- L-Glutamine --> rn:R02781 { 2,4,6/3,5-Pentahydroxycyclohexanone }

rn:R11021 <-- S-(Hercyn-2-yl)-L-cysteine S-oxide --> rn:R11864 -- Pyruvate --> rn:R05136 { Salicylaldehyde }

rn:R11068 <-- Phenylpyruvate --> rn:R00695 -- L-Phenylalanine --> rn:R11068 { 3-[(1R,2S,5R,6S)-5-Hydroxy-7-oxabicyclo[4.1.0]heptan-2-yl]-2-oxopropanoate }

rn:R11068 <-- Phenylpyruvate --> rn:R01372 -- 2-Hydroxyphenylacetate --> rn:R05001 { 2-Hydroxyphenylacetate }

rn:R11068 <-- Phenylpyruvate --> rn:R01372 -- 2-Hydroxyphenylacetate --> rn:R05450 { 2-Hydroxyphenylacetate }

rn:R11068 <-- Phenylpyruvate --> rn:R01375 -- L-Phenylalanine --> rn:R11068 { 3-[(1R,2S,5R,6S)-5-Hydroxy-7-oxabicyclo[4.1.0]heptan-2-yl]-2-oxopropanoate }

rn:R11068 <-- Phenylpyruvate --> rn:R01376 -- L-Phenylalanine --> rn:R11068 { 3-[(1R,2S,5R,6S)-5-Hydroxy-7-oxabicyclo[4.1.0]heptan-2-yl]-2-oxopropanoate }

rn:R11070 <-- Styrene --> rn:R05417 -- Styrene-cis-2,3-dihydrodiol --> rn:R05354 { Styrene-cis-2,3-dihydrodiol }

rn:R11070 <-- Styrene --> rn:R05488 -- FAD --> rn:R05537 { 3-(2-Hydroxyphenyl)propanoate }

rn:R11110 <-- (6Z,9Z,12Z,15Z,18Z,21Z)-Tetracosahexaenoyl-CoA --> rn:R07934 -- Hydrogen peroxide --> rn:R01703 { Hexadecanoic acid }

rn:R11111 <-- (6Z,9Z,12Z,15Z,18Z)-Tetracosapentaenoyl-CoA --> rn:R07950 -- Hydrogen peroxide --> rn:R01703 { Hexadecanoic acid }

rn:R11130 <-- FADH2 --> rn:R03978 -- FAD --> rn:R05537 { 3-(2-Hydroxyphenyl)propanoate }

rn:R11130 <-- FADH2 --> rn:R05488 -- FAD --> rn:R05537 { 3-(2-Hydroxyphenyl)propanoate }

rn:R11130 <-- FADH2 --> rn:R09517 -- FAD --> rn:R05537 { 3-(2-Hydroxyphenyl)propanoate }

rn:R11130 <-- FADH2 --> rn:R11653 -- FAD --> rn:R05537 { 3-(2-Hydroxyphenyl)propanoate }

rn:R11130 <-- FADH2 --> rn:R12021 -- FAD --> rn:R05537 { 3-(2-Hydroxyphenyl)propanoate }

rn:R11130 <-- FADH2 --> rn:R12023 -- FAD --> rn:R05537 { 3-(2-Hydroxyphenyl)propanoate }

rn:R11130 <-- FADH2 --> rn:R12027 -- FAD --> rn:R05537 { 3-(2-Hydroxyphenyl)propanoate }

rn:R11130 <-- FADH2 --> rn:R12030 -- FAD --> rn:R05537 { 3-(2-Hydroxyphenyl)propanoate }

rn:R11257 <-- Pyruvate --> rn:R00210 -- Acetyl-CoA --> rn:R08530 { (-)-Menthol }

rn:R11257 <-- Pyruvate --> rn:R00210 -- Acetyl-CoA --> rn:R08531 { (+)-Neomenthol }

rn:R11257 <-- Pyruvate --> rn:R00210 -- Acetyl-CoA --> rn:R08532 { (+)-Borneol }

rn:R11257 <-- Pyruvate --> rn:R00210 -- Acetyl-CoA --> rn:R10474 { Cinnamyl alcohol }

rn:R11591 <-- Pyruvate --> rn:R00210 -- Acetyl-CoA --> rn:R08530 { (-)-Menthol }

rn:R11591 <-- Pyruvate --> rn:R00210 -- Acetyl-CoA --> rn:R08531 { (+)-Neomenthol }

rn:R11591 <-- Pyruvate --> rn:R00210 -- Acetyl-CoA --> rn:R08532 { (+)-Borneol }

rn:R11591 <-- Pyruvate --> rn:R00210 -- Acetyl-CoA --> rn:R10474 { Cinnamyl alcohol }

rn:R11597 <-- [Oxidized NADPH---hemoprotein reductase] --> rn:R08551 -- [Reduced NADPH---hemoprotein reductase] --> rn:R02468 { (-)-Limonene }

rn:R11597 <-- [Oxidized NADPH---hemoprotein reductase] --> rn:R08551 -- [Reduced NADPH---hemoprotein reductase] --> rn:R02469 { (-)-Limonene }

rn:R11597 <-- [Oxidized NADPH---hemoprotein reductase] --> rn:R08551 -- [Reduced NADPH---hemoprotein reductase] --> rn:R02470 { (-)-Limonene }

rn:R11597 <-- [Oxidized NADPH---hemoprotein reductase] --> rn:R08551 -- [Reduced NADPH---hemoprotein reductase] --> rn:R04366 { Linalool }

rn:R11597 <-- [Oxidized NADPH---hemoprotein reductase] --> rn:R08551 -- [Reduced NADPH---hemoprotein reductase] --> rn:R06119 { d-Limonene }

rn:R11597 <-- [Oxidized NADPH---hemoprotein reductase] --> rn:R08551 -- [Reduced NADPH---hemoprotein reductase] --> rn:R09451 { Hexadecanoic acid }

rn:R11597 <-- [Oxidized NADPH---hemoprotein reductase] --> rn:R08551 -- [Reduced NADPH---hemoprotein reductase] --> rn:R09452 { (9Z)-Octadecenoic acid }

rn:R11597 <-- [Oxidized NADPH---hemoprotein reductase] --> rn:R08551 -- [Reduced NADPH---hemoprotein reductase] --> rn:R09922 { (+)-Linalool }

rn:R11597 <-- [Oxidized NADPH---hemoprotein reductase] --> rn:R08551 -- [Reduced NADPH---hemoprotein reductase] --> rn:R09923 { (-)-Linalool }

rn:R11597 <-- [Oxidized NADPH---hemoprotein reductase] --> rn:R08551 -- [Reduced NADPH---hemoprotein reductase] --> rn:R09925 { Linalool }

rn:R11597 <-- [Oxidized NADPH---hemoprotein reductase] --> rn:R08551 -- [Reduced NADPH---hemoprotein reductase] --> rn:R09934 { Humulene }

rn:R11597 <-- [Oxidized NADPH---hemoprotein reductase] --> rn:R08551 -- [Reduced NADPH---hemoprotein reductase] --> rn:R10562 { (E,E)-Geranyllinalool }

rn:R11597 <-- [Oxidized NADPH---hemoprotein reductase] --> rn:R08551 -- [Reduced NADPH---hemoprotein reductase] --> rn:R11055 { Myrcene }

rn:R11598 <-- [Oxidized NADPH---hemoprotein reductase] --> rn:R08551 -- [Reduced NADPH---hemoprotein reductase] --> rn:R02468 { (-)-Limonene }

rn:R11598 <-- [Oxidized NADPH---hemoprotein reductase] --> rn:R08551 -- [Reduced NADPH---hemoprotein reductase] --> rn:R02469 { (-)-Limonene }

rn:R11598 <-- [Oxidized NADPH---hemoprotein reductase] --> rn:R08551 -- [Reduced NADPH---hemoprotein reductase] --> rn:R02470 { (-)-Limonene }

rn:R11598 <-- [Oxidized NADPH---hemoprotein reductase] --> rn:R08551 -- [Reduced NADPH---hemoprotein reductase] --> rn:R04366 { Linalool }

rn:R11598 <-- [Oxidized NADPH---hemoprotein reductase] --> rn:R08551 -- [Reduced NADPH---hemoprotein reductase] --> rn:R06119 { d-Limonene }

rn:R11598 <-- [Oxidized NADPH---hemoprotein reductase] --> rn:R08551 -- [Reduced NADPH---hemoprotein reductase] --> rn:R09451 { Hexadecanoic acid }

rn:R11598 <-- [Oxidized NADPH---hemoprotein reductase] --> rn:R08551 -- [Reduced NADPH---hemoprotein reductase] --> rn:R09452 { (9Z)-Octadecenoic acid }

rn:R11598 <-- [Oxidized NADPH---hemoprotein reductase] --> rn:R08551 -- [Reduced NADPH---hemoprotein reductase] --> rn:R09922 { (+)-Linalool }

rn:R11598 <-- [Oxidized NADPH---hemoprotein reductase] --> rn:R08551 -- [Reduced NADPH---hemoprotein reductase] --> rn:R09923 { (-)-Linalool }

rn:R11598 <-- [Oxidized NADPH---hemoprotein reductase] --> rn:R08551 -- [Reduced NADPH---hemoprotein reductase] --> rn:R09925 { Linalool }

rn:R11598 <-- [Oxidized NADPH---hemoprotein reductase] --> rn:R08551 -- [Reduced NADPH---hemoprotein reductase] --> rn:R09934 { Humulene }

rn:R11598 <-- [Oxidized NADPH---hemoprotein reductase] --> rn:R08551 -- [Reduced NADPH---hemoprotein reductase] --> rn:R10562 { (E,E)-Geranyllinalool }

rn:R11598 <-- [Oxidized NADPH---hemoprotein reductase] --> rn:R08551 -- [Reduced NADPH---hemoprotein reductase] --> rn:R11055 { Myrcene }

rn:R11640 <-- [Oxidized NADPH---hemoprotein reductase] --> rn:R08551 -- [Reduced NADPH---hemoprotein reductase] --> rn:R02468 { (-)-Limonene }

rn:R11640 <-- [Oxidized NADPH---hemoprotein reductase] --> rn:R08551 -- [Reduced NADPH---hemoprotein reductase] --> rn:R02469 { (-)-Limonene }

rn:R11640 <-- [Oxidized NADPH---hemoprotein reductase] --> rn:R08551 -- [Reduced NADPH---hemoprotein reductase] --> rn:R02470 { (-)-Limonene }

rn:R11640 <-- [Oxidized NADPH---hemoprotein reductase] --> rn:R08551 -- [Reduced NADPH---hemoprotein reductase] --> rn:R04366 { Linalool }

rn:R11640 <-- [Oxidized NADPH---hemoprotein reductase] --> rn:R08551 -- [Reduced NADPH---hemoprotein reductase] --> rn:R06119 { d-Limonene }

rn:R11640 <-- [Oxidized NADPH---hemoprotein reductase] --> rn:R08551 -- [Reduced NADPH---hemoprotein reductase] --> rn:R09451 { Hexadecanoic acid }

rn:R11640 <-- [Oxidized NADPH---hemoprotein reductase] --> rn:R08551 -- [Reduced NADPH---hemoprotein reductase] --> rn:R09452 { (9Z)-Octadecenoic acid }

rn:R11640 <-- [Oxidized NADPH---hemoprotein reductase] --> rn:R08551 -- [Reduced NADPH---hemoprotein reductase] --> rn:R09922 { (+)-Linalool }

rn:R11640 <-- [Oxidized NADPH---hemoprotein reductase] --> rn:R08551 -- [Reduced NADPH---hemoprotein reductase] --> rn:R09923 { (-)-Linalool }

rn:R11640 <-- [Oxidized NADPH---hemoprotein reductase] --> rn:R08551 -- [Reduced NADPH---hemoprotein reductase] --> rn:R09925 { Linalool }

rn:R11640 <-- [Oxidized NADPH---hemoprotein reductase] --> rn:R08551 -- [Reduced NADPH---hemoprotein reductase] --> rn:R09934 { Humulene }

rn:R11640 <-- [Oxidized NADPH---hemoprotein reductase] --> rn:R08551 -- [Reduced NADPH---hemoprotein reductase] --> rn:R10562 { (E,E)-Geranyllinalool }

rn:R11640 <-- [Oxidized NADPH---hemoprotein reductase] --> rn:R08551 -- [Reduced NADPH---hemoprotein reductase] --> rn:R11055 { Myrcene }

rn:R11642 <-- [Oxidized NADPH---hemoprotein reductase] --> rn:R08551 -- [Reduced NADPH---hemoprotein reductase] --> rn:R02468 { (-)-Limonene }

rn:R11642 <-- [Oxidized NADPH---hemoprotein reductase] --> rn:R08551 -- [Reduced NADPH---hemoprotein reductase] --> rn:R02469 { (-)-Limonene }

rn:R11642 <-- [Oxidized NADPH---hemoprotein reductase] --> rn:R08551 -- [Reduced NADPH---hemoprotein reductase] --> rn:R02470 { (-)-Limonene }

rn:R11642 <-- [Oxidized NADPH---hemoprotein reductase] --> rn:R08551 -- [Reduced NADPH---hemoprotein reductase] --> rn:R04366 { Linalool }

rn:R11642 <-- [Oxidized NADPH---hemoprotein reductase] --> rn:R08551 -- [Reduced NADPH---hemoprotein reductase] --> rn:R06119 { d-Limonene }

rn:R11642 <-- [Oxidized NADPH---hemoprotein reductase] --> rn:R08551 -- [Reduced NADPH---hemoprotein reductase] --> rn:R09451 { Hexadecanoic acid }

rn:R11642 <-- [Oxidized NADPH---hemoprotein reductase] --> rn:R08551 -- [Reduced NADPH---hemoprotein reductase] --> rn:R09452 { (9Z)-Octadecenoic acid }

rn:R11642 <-- [Oxidized NADPH---hemoprotein reductase] --> rn:R08551 -- [Reduced NADPH---hemoprotein reductase] --> rn:R09922 { (+)-Linalool }

rn:R11642 <-- [Oxidized NADPH---hemoprotein reductase] --> rn:R08551 -- [Reduced NADPH---hemoprotein reductase] --> rn:R09923 { (-)-Linalool }

rn:R11642 <-- [Oxidized NADPH---hemoprotein reductase] --> rn:R08551 -- [Reduced NADPH---hemoprotein reductase] --> rn:R09925 { Linalool }

rn:R11642 <-- [Oxidized NADPH---hemoprotein reductase] --> rn:R08551 -- [Reduced NADPH---hemoprotein reductase] --> rn:R09934 { Humulene }

rn:R11642 <-- [Oxidized NADPH---hemoprotein reductase] --> rn:R08551 -- [Reduced NADPH---hemoprotein reductase] --> rn:R10562 { (E,E)-Geranyllinalool }

rn:R11642 <-- [Oxidized NADPH---hemoprotein reductase] --> rn:R08551 -- [Reduced NADPH---hemoprotein reductase] --> rn:R11055 { Myrcene }

rn:R11653 <-- FAD --> rn:R02487 -- FADH2 --> rn:R05488 { Styrene }

rn:R11653 <-- FAD --> rn:R04095 -- FADH2 --> rn:R05488 { Styrene }

rn:R11653 <-- FAD --> rn:R05537 -- FADH2 --> rn:R05488 { Styrene }

rn:R11653 <-- FAD --> rn:R05537 -- Acetyl-CoA --> rn:R08530 { (-)-Menthol }

rn:R11653 <-- FAD --> rn:R05537 -- Acetyl-CoA --> rn:R08531 { (+)-Neomenthol }

rn:R11653 <-- FAD --> rn:R05537 -- Acetyl-CoA --> rn:R08532 { (+)-Borneol }

rn:R11653 <-- FAD --> rn:R05537 -- Acetyl-CoA --> rn:R10474 { Cinnamyl alcohol }

rn:R11653 <-- FAD --> rn:R06943 -- FADH2 --> rn:R05488 { Styrene }

rn:R11653 <-- FAD --> rn:R07220 -- FADH2 --> rn:R05488 { Styrene }

rn:R11653 <-- FAD --> rn:R09520 -- FADH2 --> rn:R05488 { Styrene }

rn:R11653 <-- FAD --> rn:R11130 -- FADH2 --> rn:R05488 { Styrene }

rn:R11732 <-- Mandelonitrile --> rn:R01767 -- Benzaldehyde --> rn:R01419 { Benzaldehyde }

rn:R11732 <-- Mandelonitrile --> rn:R01767 -- Benzaldehyde --> rn:R01420 { Benzaldehyde }

rn:R11732 <-- [Oxidized NADPH---hemoprotein reductase] --> rn:R08551 -- [Reduced NADPH---hemoprotein reductase] --> rn:R02468 { (-)-Limonene }

rn:R11732 <-- [Oxidized NADPH---hemoprotein reductase] --> rn:R08551 -- [Reduced NADPH---hemoprotein reductase] --> rn:R02469 { (-)-Limonene }

rn:R11732 <-- [Oxidized NADPH---hemoprotein reductase] --> rn:R08551 -- [Reduced NADPH---hemoprotein reductase] --> rn:R02470 { (-)-Limonene }

rn:R11732 <-- [Oxidized NADPH---hemoprotein reductase] --> rn:R08551 -- [Reduced NADPH---hemoprotein reductase] --> rn:R04366 { Linalool }

rn:R11732 <-- [Oxidized NADPH---hemoprotein reductase] --> rn:R08551 -- [Reduced NADPH---hemoprotein reductase] --> rn:R06119 { d-Limonene }

rn:R11732 <-- [Oxidized NADPH---hemoprotein reductase] --> rn:R08551 -- [Reduced NADPH---hemoprotein reductase] --> rn:R09451 { Hexadecanoic acid }

rn:R11732 <-- [Oxidized NADPH---hemoprotein reductase] --> rn:R08551 -- [Reduced NADPH---hemoprotein reductase] --> rn:R09452 { (9Z)-Octadecenoic acid }

rn:R11732 <-- [Oxidized NADPH---hemoprotein reductase] --> rn:R08551 -- [Reduced NADPH---hemoprotein reductase] --> rn:R09922 { (+)-Linalool }

rn:R11732 <-- [Oxidized NADPH---hemoprotein reductase] --> rn:R08551 -- [Reduced NADPH---hemoprotein reductase] --> rn:R09923 { (-)-Linalool }

rn:R11732 <-- [Oxidized NADPH---hemoprotein reductase] --> rn:R08551 -- [Reduced NADPH---hemoprotein reductase] --> rn:R09925 { Linalool }

rn:R11732 <-- [Oxidized NADPH---hemoprotein reductase] --> rn:R08551 -- [Reduced NADPH---hemoprotein reductase] --> rn:R09934 { Humulene }

rn:R11732 <-- [Oxidized NADPH---hemoprotein reductase] --> rn:R08551 -- [Reduced NADPH---hemoprotein reductase] --> rn:R10562 { (E,E)-Geranyllinalool }

rn:R11732 <-- [Oxidized NADPH---hemoprotein reductase] --> rn:R08551 -- [Reduced NADPH---hemoprotein reductase] --> rn:R11055 { Myrcene }

rn:R11732 <-- Mandelonitrile --> rn:R11380 -- Hydrogen peroxide --> rn:R01703 { Hexadecanoic acid }

rn:R11733 <-- Mandelonitrile --> rn:R01767 -- Benzaldehyde --> rn:R01419 { Benzaldehyde }

rn:R11733 <-- Mandelonitrile --> rn:R01767 -- Benzaldehyde --> rn:R01420 { Benzaldehyde }

rn:R11733 <-- [Oxidized NADPH---hemoprotein reductase] --> rn:R08551 -- [Reduced NADPH---hemoprotein reductase] --> rn:R02468 { (-)-Limonene }

rn:R11733 <-- [Oxidized NADPH---hemoprotein reductase] --> rn:R08551 -- [Reduced NADPH---hemoprotein reductase] --> rn:R02469 { (-)-Limonene }

rn:R11733 <-- [Oxidized NADPH---hemoprotein reductase] --> rn:R08551 -- [Reduced NADPH---hemoprotein reductase] --> rn:R02470 { (-)-Limonene }

rn:R11733 <-- [Oxidized NADPH---hemoprotein reductase] --> rn:R08551 -- [Reduced NADPH---hemoprotein reductase] --> rn:R04366 { Linalool }

rn:R11733 <-- [Oxidized NADPH---hemoprotein reductase] --> rn:R08551 -- [Reduced NADPH---hemoprotein reductase] --> rn:R06119 { d-Limonene }

rn:R11733 <-- [Oxidized NADPH---hemoprotein reductase] --> rn:R08551 -- [Reduced NADPH---hemoprotein reductase] --> rn:R09451 { Hexadecanoic acid }

rn:R11733 <-- [Oxidized NADPH---hemoprotein reductase] --> rn:R08551 -- [Reduced NADPH---hemoprotein reductase] --> rn:R09452 { (9Z)-Octadecenoic acid }

rn:R11733 <-- [Oxidized NADPH---hemoprotein reductase] --> rn:R08551 -- [Reduced NADPH---hemoprotein reductase] --> rn:R09922 { (+)-Linalool }

rn:R11733 <-- [Oxidized NADPH---hemoprotein reductase] --> rn:R08551 -- [Reduced NADPH---hemoprotein reductase] --> rn:R09923 { (-)-Linalool }

rn:R11733 <-- [Oxidized NADPH---hemoprotein reductase] --> rn:R08551 -- [Reduced NADPH---hemoprotein reductase] --> rn:R09925 { Linalool }

rn:R11733 <-- [Oxidized NADPH---hemoprotein reductase] --> rn:R08551 -- [Reduced NADPH---hemoprotein reductase] --> rn:R09934 { Humulene }

rn:R11733 <-- [Oxidized NADPH---hemoprotein reductase] --> rn:R08551 -- [Reduced NADPH---hemoprotein reductase] --> rn:R10562 { (E,E)-Geranyllinalool }

rn:R11733 <-- [Oxidized NADPH---hemoprotein reductase] --> rn:R08551 -- [Reduced NADPH---hemoprotein reductase] --> rn:R11055 { Myrcene }

rn:R11733 <-- Mandelonitrile --> rn:R11380 -- Hydrogen peroxide --> rn:R01703 { Hexadecanoic acid }

rn:R11737 <-- [Oxidized NADPH---hemoprotein reductase] --> rn:R08551 -- [Reduced NADPH---hemoprotein reductase] --> rn:R02468 { (-)-Limonene }

rn:R11737 <-- [Oxidized NADPH---hemoprotein reductase] --> rn:R08551 -- [Reduced NADPH---hemoprotein reductase] --> rn:R02469 { (-)-Limonene }

rn:R11737 <-- [Oxidized NADPH---hemoprotein reductase] --> rn:R08551 -- [Reduced NADPH---hemoprotein reductase] --> rn:R02470 { (-)-Limonene }

rn:R11737 <-- [Oxidized NADPH---hemoprotein reductase] --> rn:R08551 -- [Reduced NADPH---hemoprotein reductase] --> rn:R04366 { Linalool }

rn:R11737 <-- [Oxidized NADPH---hemoprotein reductase] --> rn:R08551 -- [Reduced NADPH---hemoprotein reductase] --> rn:R06119 { d-Limonene }

rn:R11737 <-- [Oxidized NADPH---hemoprotein reductase] --> rn:R08551 -- [Reduced NADPH---hemoprotein reductase] --> rn:R09451 { Hexadecanoic acid }

rn:R11737 <-- [Oxidized NADPH---hemoprotein reductase] --> rn:R08551 -- [Reduced NADPH---hemoprotein reductase] --> rn:R09452 { (9Z)-Octadecenoic acid }

rn:R11737 <-- [Oxidized NADPH---hemoprotein reductase] --> rn:R08551 -- [Reduced NADPH---hemoprotein reductase] --> rn:R09922 { (+)-Linalool }

rn:R11737 <-- [Oxidized NADPH---hemoprotein reductase] --> rn:R08551 -- [Reduced NADPH---hemoprotein reductase] --> rn:R09923 { (-)-Linalool }

rn:R11737 <-- [Oxidized NADPH---hemoprotein reductase] --> rn:R08551 -- [Reduced NADPH---hemoprotein reductase] --> rn:R09925 { Linalool }

rn:R11737 <-- [Oxidized NADPH---hemoprotein reductase] --> rn:R08551 -- [Reduced NADPH---hemoprotein reductase] --> rn:R09934 { Humulene }

rn:R11737 <-- [Oxidized NADPH---hemoprotein reductase] --> rn:R08551 -- [Reduced NADPH---hemoprotein reductase] --> rn:R10562 { (E,E)-Geranyllinalool }

rn:R11737 <-- [Oxidized NADPH---hemoprotein reductase] --> rn:R08551 -- [Reduced NADPH---hemoprotein reductase] --> rn:R11055 { Myrcene }

rn:R11738 <-- [Oxidized NADPH---hemoprotein reductase] --> rn:R08551 -- [Reduced NADPH---hemoprotein reductase] --> rn:R02468 { (-)-Limonene }

rn:R11738 <-- [Oxidized NADPH---hemoprotein reductase] --> rn:R08551 -- [Reduced NADPH---hemoprotein reductase] --> rn:R02469 { (-)-Limonene }

rn:R11738 <-- [Oxidized NADPH---hemoprotein reductase] --> rn:R08551 -- [Reduced NADPH---hemoprotein reductase] --> rn:R02470 { (-)-Limonene }

rn:R11738 <-- [Oxidized NADPH---hemoprotein reductase] --> rn:R08551 -- [Reduced NADPH---hemoprotein reductase] --> rn:R04366 { Linalool }

rn:R11738 <-- [Oxidized NADPH---hemoprotein reductase] --> rn:R08551 -- [Reduced NADPH---hemoprotein reductase] --> rn:R06119 { d-Limonene }

rn:R11738 <-- [Oxidized NADPH---hemoprotein reductase] --> rn:R08551 -- [Reduced NADPH---hemoprotein reductase] --> rn:R09451 { Hexadecanoic acid }

rn:R11738 <-- [Oxidized NADPH---hemoprotein reductase] --> rn:R08551 -- [Reduced NADPH---hemoprotein reductase] --> rn:R09452 { (9Z)-Octadecenoic acid }

rn:R11738 <-- [Oxidized NADPH---hemoprotein reductase] --> rn:R08551 -- [Reduced NADPH---hemoprotein reductase] --> rn:R09922 { (+)-Linalool }

rn:R11738 <-- [Oxidized NADPH---hemoprotein reductase] --> rn:R08551 -- [Reduced NADPH---hemoprotein reductase] --> rn:R09923 { (-)-Linalool }

rn:R11738 <-- [Oxidized NADPH---hemoprotein reductase] --> rn:R08551 -- [Reduced NADPH---hemoprotein reductase] --> rn:R09925 { Linalool }

rn:R11738 <-- [Oxidized NADPH---hemoprotein reductase] --> rn:R08551 -- [Reduced NADPH---hemoprotein reductase] --> rn:R09934 { Humulene }

rn:R11738 <-- [Oxidized NADPH---hemoprotein reductase] --> rn:R08551 -- [Reduced NADPH---hemoprotein reductase] --> rn:R10562 { (E,E)-Geranyllinalool }

rn:R11738 <-- [Oxidized NADPH---hemoprotein reductase] --> rn:R08551 -- [Reduced NADPH---hemoprotein reductase] --> rn:R11055 { Myrcene }

rn:R11813 <-- [Oxidized NADPH---hemoprotein reductase] --> rn:R08551 -- [Reduced NADPH---hemoprotein reductase] --> rn:R02468 { (-)-Limonene }

rn:R11813 <-- [Oxidized NADPH---hemoprotein reductase] --> rn:R08551 -- [Reduced NADPH---hemoprotein reductase] --> rn:R02469 { (-)-Limonene }

rn:R11813 <-- [Oxidized NADPH---hemoprotein reductase] --> rn:R08551 -- [Reduced NADPH---hemoprotein reductase] --> rn:R02470 { (-)-Limonene }

rn:R11813 <-- [Oxidized NADPH---hemoprotein reductase] --> rn:R08551 -- [Reduced NADPH---hemoprotein reductase] --> rn:R04366 { Linalool }

rn:R11813 <-- [Oxidized NADPH---hemoprotein reductase] --> rn:R08551 -- [Reduced NADPH---hemoprotein reductase] --> rn:R06119 { d-Limonene }

rn:R11813 <-- [Oxidized NADPH---hemoprotein reductase] --> rn:R08551 -- [Reduced NADPH---hemoprotein reductase] --> rn:R09451 { Hexadecanoic acid }

rn:R11813 <-- [Oxidized NADPH---hemoprotein reductase] --> rn:R08551 -- [Reduced NADPH---hemoprotein reductase] --> rn:R09452 { (9Z)-Octadecenoic acid }

rn:R11813 <-- [Oxidized NADPH---hemoprotein reductase] --> rn:R08551 -- [Reduced NADPH---hemoprotein reductase] --> rn:R09922 { (+)-Linalool }

rn:R11813 <-- [Oxidized NADPH---hemoprotein reductase] --> rn:R08551 -- [Reduced NADPH---hemoprotein reductase] --> rn:R09923 { (-)-Linalool }

rn:R11813 <-- [Oxidized NADPH---hemoprotein reductase] --> rn:R08551 -- [Reduced NADPH---hemoprotein reductase] --> rn:R09925 { Linalool }

rn:R11813 <-- [Oxidized NADPH---hemoprotein reductase] --> rn:R08551 -- [Reduced NADPH---hemoprotein reductase] --> rn:R09934 { Humulene }

rn:R11813 <-- [Oxidized NADPH---hemoprotein reductase] --> rn:R08551 -- [Reduced NADPH---hemoprotein reductase] --> rn:R10562 { (E,E)-Geranyllinalool }

rn:R11813 <-- [Oxidized NADPH---hemoprotein reductase] --> rn:R08551 -- [Reduced NADPH---hemoprotein reductase] --> rn:R11055 { Myrcene }

rn:R11814 <-- [Oxidized NADPH---hemoprotein reductase] --> rn:R08551 -- [Reduced NADPH---hemoprotein reductase] --> rn:R02468 { (-)-Limonene }

rn:R11814 <-- [Oxidized NADPH---hemoprotein reductase] --> rn:R08551 -- [Reduced NADPH---hemoprotein reductase] --> rn:R02469 { (-)-Limonene }

rn:R11814 <-- [Oxidized NADPH---hemoprotein reductase] --> rn:R08551 -- [Reduced NADPH---hemoprotein reductase] --> rn:R02470 { (-)-Limonene }

rn:R11814 <-- [Oxidized NADPH---hemoprotein reductase] --> rn:R08551 -- [Reduced NADPH---hemoprotein reductase] --> rn:R04366 { Linalool }

rn:R11814 <-- [Oxidized NADPH---hemoprotein reductase] --> rn:R08551 -- [Reduced NADPH---hemoprotein reductase] --> rn:R06119 { d-Limonene }

rn:R11814 <-- [Oxidized NADPH---hemoprotein reductase] --> rn:R08551 -- [Reduced NADPH---hemoprotein reductase] --> rn:R09451 { Hexadecanoic acid }

rn:R11814 <-- [Oxidized NADPH---hemoprotein reductase] --> rn:R08551 -- [Reduced NADPH---hemoprotein reductase] --> rn:R09452 { (9Z)-Octadecenoic acid }

rn:R11814 <-- [Oxidized NADPH---hemoprotein reductase] --> rn:R08551 -- [Reduced NADPH---hemoprotein reductase] --> rn:R09922 { (+)-Linalool }

rn:R11814 <-- [Oxidized NADPH---hemoprotein reductase] --> rn:R08551 -- [Reduced NADPH---hemoprotein reductase] --> rn:R09923 { (-)-Linalool }

rn:R11814 <-- [Oxidized NADPH---hemoprotein reductase] --> rn:R08551 -- [Reduced NADPH---hemoprotein reductase] --> rn:R09925 { Linalool }

rn:R11814 <-- [Oxidized NADPH---hemoprotein reductase] --> rn:R08551 -- [Reduced NADPH---hemoprotein reductase] --> rn:R09934 { Humulene }

rn:R11814 <-- [Oxidized NADPH---hemoprotein reductase] --> rn:R08551 -- [Reduced NADPH---hemoprotein reductase] --> rn:R10562 { (E,E)-Geranyllinalool }

rn:R11814 <-- [Oxidized NADPH---hemoprotein reductase] --> rn:R08551 -- [Reduced NADPH---hemoprotein reductase] --> rn:R11055 { Myrcene }

rn:R11864 <-- Pyruvate --> rn:R00210 -- Acetyl-CoA --> rn:R08530 { (-)-Menthol }

rn:R11864 <-- Pyruvate --> rn:R00210 -- Acetyl-CoA --> rn:R08531 { (+)-Neomenthol }

rn:R11864 <-- Pyruvate --> rn:R00210 -- Acetyl-CoA --> rn:R08532 { (+)-Borneol }

rn:R11864 <-- Pyruvate --> rn:R00210 -- Acetyl-CoA --> rn:R10474 { Cinnamyl alcohol }

rn:R11864 <-- 2-(Hydroxysulfanyl)hercynine --> rn:R11865 -- Acceptor --> rn:R02234 { Cyclohexanone }

rn:R11864 <-- 2-(Hydroxysulfanyl)hercynine --> rn:R11865 -- Acceptor --> rn:R03212 { 3-Hydroxycyclohexanone }

rn:R11903 <-- Acetate --> rn:R01241 -- Phenyl acetate --> rn:R07342 { Phenyl acetate }

rn:R12021 <-- FAD --> rn:R02487 -- FADH2 --> rn:R05488 { Styrene }

rn:R12021 <-- FAD --> rn:R04095 -- FADH2 --> rn:R05488 { Styrene }

rn:R12021 <-- FAD --> rn:R05537 -- FADH2 --> rn:R05488 { Styrene }

rn:R12021 <-- FAD --> rn:R05537 -- Acetyl-CoA --> rn:R08530 { (-)-Menthol }

rn:R12021 <-- FAD --> rn:R05537 -- Acetyl-CoA --> rn:R08531 { (+)-Neomenthol }

rn:R12021 <-- FAD --> rn:R05537 -- Acetyl-CoA --> rn:R08532 { (+)-Borneol }

rn:R12021 <-- FAD --> rn:R05537 -- Acetyl-CoA --> rn:R10474 { Cinnamyl alcohol }

rn:R12021 <-- FAD --> rn:R06943 -- FADH2 --> rn:R05488 { Styrene }

rn:R12021 <-- FAD --> rn:R07220 -- FADH2 --> rn:R05488 { Styrene }

rn:R12021 <-- FAD --> rn:R09520 -- FADH2 --> rn:R05488 { Styrene }

rn:R12021 <-- FAD --> rn:R11130 -- FADH2 --> rn:R05488 { Styrene }

rn:R12023 <-- FAD --> rn:R02487 -- FADH2 --> rn:R05488 { Styrene }

rn:R12023 <-- FAD --> rn:R04095 -- FADH2 --> rn:R05488 { Styrene }

rn:R12023 <-- FAD --> rn:R05537 -- FADH2 --> rn:R05488 { Styrene }

rn:R12023 <-- FAD --> rn:R05537 -- Acetyl-CoA --> rn:R08530 { (-)-Menthol }

rn:R12023 <-- FAD --> rn:R05537 -- Acetyl-CoA --> rn:R08531 { (+)-Neomenthol }

rn:R12023 <-- FAD --> rn:R05537 -- Acetyl-CoA --> rn:R08532 { (+)-Borneol }

rn:R12023 <-- FAD --> rn:R05537 -- Acetyl-CoA --> rn:R10474 { Cinnamyl alcohol }

rn:R12023 <-- FAD --> rn:R06943 -- FADH2 --> rn:R05488 { Styrene }

rn:R12023 <-- FAD --> rn:R07220 -- FADH2 --> rn:R05488 { Styrene }

rn:R12023 <-- FAD --> rn:R09520 -- FADH2 --> rn:R05488 { Styrene }

rn:R12023 <-- FAD --> rn:R11130 -- FADH2 --> rn:R05488 { Styrene }

rn:R12027 <-- FAD --> rn:R02487 -- FADH2 --> rn:R05488 { Styrene }

rn:R12027 <-- FAD --> rn:R04095 -- FADH2 --> rn:R05488 { Styrene }

rn:R12027 <-- FAD --> rn:R05537 -- FADH2 --> rn:R05488 { Styrene }

rn:R12027 <-- FAD --> rn:R05537 -- Acetyl-CoA --> rn:R08530 { (-)-Menthol }

rn:R12027 <-- FAD --> rn:R05537 -- Acetyl-CoA --> rn:R08531 { (+)-Neomenthol }

rn:R12027 <-- FAD --> rn:R05537 -- Acetyl-CoA --> rn:R08532 { (+)-Borneol }

rn:R12027 <-- FAD --> rn:R05537 -- Acetyl-CoA --> rn:R10474 { Cinnamyl alcohol }

rn:R12027 <-- FAD --> rn:R06943 -- FADH2 --> rn:R05488 { Styrene }

rn:R12027 <-- FAD --> rn:R07220 -- FADH2 --> rn:R05488 { Styrene }

rn:R12027 <-- FAD --> rn:R09520 -- FADH2 --> rn:R05488 { Styrene }

rn:R12027 <-- FAD --> rn:R11130 -- FADH2 --> rn:R05488 { Styrene }

rn:R12030 <-- FAD --> rn:R02487 -- FADH2 --> rn:R05488 { Styrene }

rn:R12030 <-- FAD --> rn:R04095 -- FADH2 --> rn:R05488 { Styrene }

rn:R12030 <-- FAD --> rn:R05537 -- FADH2 --> rn:R05488 { Styrene }

rn:R12030 <-- FAD --> rn:R05537 -- Acetyl-CoA --> rn:R08530 { (-)-Menthol }

rn:R12030 <-- FAD --> rn:R05537 -- Acetyl-CoA --> rn:R08531 { (+)-Neomenthol }

rn:R12030 <-- FAD --> rn:R05537 -- Acetyl-CoA --> rn:R08532 { (+)-Borneol }

rn:R12030 <-- FAD --> rn:R05537 -- Acetyl-CoA --> rn:R10474 { Cinnamyl alcohol }

rn:R12030 <-- FAD --> rn:R06943 -- FADH2 --> rn:R05488 { Styrene }

rn:R12030 <-- FAD --> rn:R07220 -- FADH2 --> rn:R05488 { Styrene }

rn:R12030 <-- FAD --> rn:R09520 -- FADH2 --> rn:R05488 { Styrene }

rn:R12030 <-- FAD --> rn:R11130 -- FADH2 --> rn:R05488 { Styrene }

rn:R12048 <-- Oleoyl-CoA --> rn:R08176 -- (9Z)-Octadecenoic acid --> rn:R09452 { (9Z)-Octadecenoic acid }

rn:R12048 <-- Oleoyl-CoA --> rn:R08176 -- (9Z)-Octadecenoic acid --> rn:R09462 { (9Z)-Octadecenoic acid }

rn:R12048 <-- Oleoyl-CoA --> rn:R08176 -- (9Z)-Octadecenoic acid --> rn:R10576 { (9Z)-Octadecenoic acid }

rn:R12072 <-- [Oxidized NADPH---hemoprotein reductase] --> rn:R08551 -- [Reduced NADPH---hemoprotein reductase] --> rn:R02468 { (-)-Limonene }

rn:R12072 <-- [Oxidized NADPH---hemoprotein reductase] --> rn:R08551 -- [Reduced NADPH---hemoprotein reductase] --> rn:R02469 { (-)-Limonene }

rn:R12072 <-- [Oxidized NADPH---hemoprotein reductase] --> rn:R08551 -- [Reduced NADPH---hemoprotein reductase] --> rn:R02470 { (-)-Limonene }

rn:R12072 <-- [Oxidized NADPH---hemoprotein reductase] --> rn:R08551 -- [Reduced NADPH---hemoprotein reductase] --> rn:R04366 { Linalool }

rn:R12072 <-- [Oxidized NADPH---hemoprotein reductase] --> rn:R08551 -- [Reduced NADPH---hemoprotein reductase] --> rn:R06119 { d-Limonene }

rn:R12072 <-- [Oxidized NADPH---hemoprotein reductase] --> rn:R08551 -- [Reduced NADPH---hemoprotein reductase] --> rn:R09451 { Hexadecanoic acid }

rn:R12072 <-- [Oxidized NADPH---hemoprotein reductase] --> rn:R08551 -- [Reduced NADPH---hemoprotein reductase] --> rn:R09452 { (9Z)-Octadecenoic acid }

rn:R12072 <-- [Oxidized NADPH---hemoprotein reductase] --> rn:R08551 -- [Reduced NADPH---hemoprotein reductase] --> rn:R09922 { (+)-Linalool }

rn:R12072 <-- [Oxidized NADPH---hemoprotein reductase] --> rn:R08551 -- [Reduced NADPH---hemoprotein reductase] --> rn:R09923 { (-)-Linalool }

rn:R12072 <-- [Oxidized NADPH---hemoprotein reductase] --> rn:R08551 -- [Reduced NADPH---hemoprotein reductase] --> rn:R09925 { Linalool }

rn:R12072 <-- [Oxidized NADPH---hemoprotein reductase] --> rn:R08551 -- [Reduced NADPH---hemoprotein reductase] --> rn:R09934 { Humulene }

rn:R12072 <-- [Oxidized NADPH---hemoprotein reductase] --> rn:R08551 -- [Reduced NADPH---hemoprotein reductase] --> rn:R10562 { (E,E)-Geranyllinalool }

rn:R12072 <-- [Oxidized NADPH---hemoprotein reductase] --> rn:R08551 -- [Reduced NADPH---hemoprotein reductase] --> rn:R11055 { Myrcene }

rn:R12112 <-- p-Coumaroyl-CoA --> rn:R08767 -- Acetyl-CoA --> rn:R08530 { (-)-Menthol }

rn:R12112 <-- p-Coumaroyl-CoA --> rn:R08767 -- Acetyl-CoA --> rn:R08531 { (+)-Neomenthol }

rn:R12112 <-- p-Coumaroyl-CoA --> rn:R08767 -- Acetyl-CoA --> rn:R08532 { (+)-Borneol }

rn:R12112 <-- p-Coumaroyl-CoA --> rn:R08767 -- Acetyl-CoA --> rn:R10474 { Cinnamyl alcohol }

rn:R12183 <-- [Oxidized NADPH---hemoprotein reductase] --> rn:R08551 -- [Reduced NADPH---hemoprotein reductase] --> rn:R02468 { (-)-Limonene }

rn:R12183 <-- [Oxidized NADPH---hemoprotein reductase] --> rn:R08551 -- [Reduced NADPH---hemoprotein reductase] --> rn:R02469 { (-)-Limonene }

rn:R12183 <-- [Oxidized NADPH---hemoprotein reductase] --> rn:R08551 -- [Reduced NADPH---hemoprotein reductase] --> rn:R02470 { (-)-Limonene }

rn:R12183 <-- [Oxidized NADPH---hemoprotein reductase] --> rn:R08551 -- [Reduced NADPH---hemoprotein reductase] --> rn:R04366 { Linalool }

rn:R12183 <-- [Oxidized NADPH---hemoprotein reductase] --> rn:R08551 -- [Reduced NADPH---hemoprotein reductase] --> rn:R06119 { d-Limonene }

rn:R12183 <-- [Oxidized NADPH---hemoprotein reductase] --> rn:R08551 -- [Reduced NADPH---hemoprotein reductase] --> rn:R09451 { Hexadecanoic acid }

rn:R12183 <-- [Oxidized NADPH---hemoprotein reductase] --> rn:R08551 -- [Reduced NADPH---hemoprotein reductase] --> rn:R09452 { (9Z)-Octadecenoic acid }

rn:R12183 <-- [Oxidized NADPH---hemoprotein reductase] --> rn:R08551 -- [Reduced NADPH---hemoprotein reductase] --> rn:R09922 { (+)-Linalool }

rn:R12183 <-- [Oxidized NADPH---hemoprotein reductase] --> rn:R08551 -- [Reduced NADPH---hemoprotein reductase] --> rn:R09923 { (-)-Linalool }

rn:R12183 <-- [Oxidized NADPH---hemoprotein reductase] --> rn:R08551 -- [Reduced NADPH---hemoprotein reductase] --> rn:R09925 { Linalool }

rn:R12183 <-- [Oxidized NADPH---hemoprotein reductase] --> rn:R08551 -- [Reduced NADPH---hemoprotein reductase] --> rn:R09934 { Humulene }

rn:R12183 <-- [Oxidized NADPH---hemoprotein reductase] --> rn:R08551 -- [Reduced NADPH---hemoprotein reductase] --> rn:R10562 { (E,E)-Geranyllinalool }

rn:R12183 <-- [Oxidized NADPH---hemoprotein reductase] --> rn:R08551 -- [Reduced NADPH---hemoprotein reductase] --> rn:R11055 { Myrcene }

rn:R12184 <-- [Oxidized NADPH---hemoprotein reductase] --> rn:R08551 -- [Reduced NADPH---hemoprotein reductase] --> rn:R02468 { (-)-Limonene }

rn:R12184 <-- [Oxidized NADPH---hemoprotein reductase] --> rn:R08551 -- [Reduced NADPH---hemoprotein reductase] --> rn:R02469 { (-)-Limonene }

rn:R12184 <-- [Oxidized NADPH---hemoprotein reductase] --> rn:R08551 -- [Reduced NADPH---hemoprotein reductase] --> rn:R02470 { (-)-Limonene }

rn:R12184 <-- [Oxidized NADPH---hemoprotein reductase] --> rn:R08551 -- [Reduced NADPH---hemoprotein reductase] --> rn:R04366 { Linalool }

rn:R12184 <-- [Oxidized NADPH---hemoprotein reductase] --> rn:R08551 -- [Reduced NADPH---hemoprotein reductase] --> rn:R06119 { d-Limonene }

rn:R12184 <-- [Oxidized NADPH---hemoprotein reductase] --> rn:R08551 -- [Reduced NADPH---hemoprotein reductase] --> rn:R09451 { Hexadecanoic acid }

rn:R12184 <-- [Oxidized NADPH---hemoprotein reductase] --> rn:R08551 -- [Reduced NADPH---hemoprotein reductase] --> rn:R09452 { (9Z)-Octadecenoic acid }

rn:R12184 <-- [Oxidized NADPH---hemoprotein reductase] --> rn:R08551 -- [Reduced NADPH---hemoprotein reductase] --> rn:R09922 { (+)-Linalool }

rn:R12184 <-- [Oxidized NADPH---hemoprotein reductase] --> rn:R08551 -- [Reduced NADPH---hemoprotein reductase] --> rn:R09923 { (-)-Linalool }

rn:R12184 <-- [Oxidized NADPH---hemoprotein reductase] --> rn:R08551 -- [Reduced NADPH---hemoprotein reductase] --> rn:R09925 { Linalool }

rn:R12184 <-- [Oxidized NADPH---hemoprotein reductase] --> rn:R08551 -- [Reduced NADPH---hemoprotein reductase] --> rn:R09934 { Humulene }

rn:R12184 <-- [Oxidized NADPH---hemoprotein reductase] --> rn:R08551 -- [Reduced NADPH---hemoprotein reductase] --> rn:R10562 { (E,E)-Geranyllinalool }

rn:R12184 <-- [Oxidized NADPH---hemoprotein reductase] --> rn:R08551 -- [Reduced NADPH---hemoprotein reductase] --> rn:R11055 { Myrcene }
