# Supplementary material for: The impact of Traditional Chinese Medicine on mouse gut microbiota abundances and interactions based on Granger causality and pathway analysis
Source: Front Microbiol. 2022 Nov 11;13:980082. doi: 10.3389/fmicb.2022.980082 (PMC9692106; doi:10.3389/fmicb.2022.980082)

**Invsimpson index for alpha diversity index**

| 7.700824 4.81645 5.842102 6.979517 4.279513 |
| --- |
| 6.017031 5.715991 5.969135 5.837191 6.045832 |
| 6.548915 6.035612 5.710265 4.011883 5.366101 |
| 7.965627 5.796925 6.684149 6.824323 4.460092 |
| 5.508136 6.464553 2.667523 4.419043 6.207651 |
| 5.491767 6.213575 4.065987 5.677088 4.163429 |
| 6.858053 6.57359 6.638729 7.314943 6.466898 |
| 6.352312 6.787451 7.207396 6.89669 5.773753 |
| 8.894207 6.007613 3.86633 3.23251 4.118428 |
| 6.010573 4.821782 5.171265 6.435095 6.481111 |
| 7.627127 6.145556 4.811807 7.842954 7.873881 |

**Simpson index for alpha diversity index**

| 0.8701438 0.7923782 0.8288287 0.8567236 0.7663285 |
| --- |
| 0.8338051 0.8250522 0.8324715 0.8286847 0.8345968 |
| 0.847303 0.8343167 0.8248768 0.7507405 0.813645 |
| 0.8744606 0.8274947 0.8503923 0.8534653 0.7757894 |
| 0.8184504 0.8453103 0.6251204 0.7737066 0.8389085 |
| 0.8179092 0.839062 0.7540573 0.8238534 0.7598133 |
| 0.854186 0.8478761 0.8493688 0.8632935 0.8453663 |
| 0.842577 0.8526693 0.8612536 0.8550029 0.8268024 |
| 0.8875673 0.8335445 0.7413568 0.6906428 0.7571889 |
| 0.8336265 0.7926078 0.8066237 0.8446021 0.8457055 |
| 0.868889 0.8372808 0.7921779 0.872497 0.8729978 |

The correlation between Alpha diversity Shannon, simpson and invsimpson index


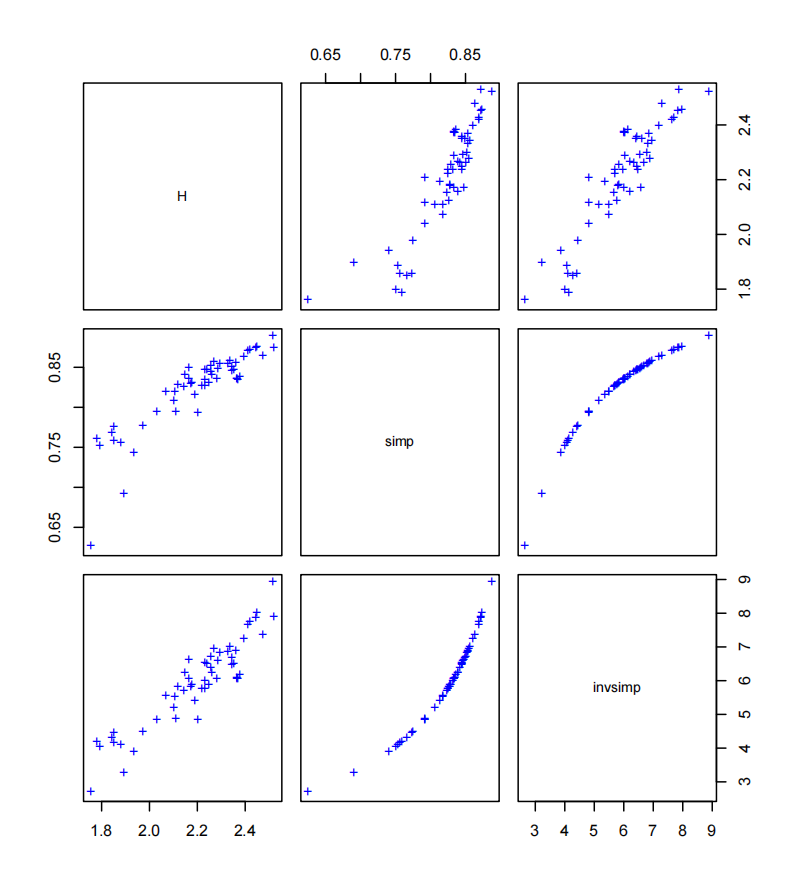

Supplement: Supplementary file 5 [file Table_5.docx]
