# Supplementary material for: The impact of Traditional Chinese Medicine on mouse gut microbiota abundances and interactions based on Granger causality and pathway analysis
Source: Front Microbiol. 2022 Nov 11;13:980082. doi: 10.3389/fmicb.2022.980082 (PMC9692106; doi:10.3389/fmicb.2022.980082)
Supplement: Supplementary file 8 [file Table_8.doc]

rn:R07712 { Liquiritigenin } <-- 2-Oxoglutarate -- rn:R00265 <-- Succinyl-CoA --> rn:R02407

rn:R07996 { Liquiritigenin } <-- 2-Oxoglutarate -- rn:R00265 <-- Succinyl-CoA --> rn:R02407

rn:R07712 { Liquiritigenin } <-- 2-Oxoglutarate -- rn:R00265 <-- Succinyl-CoA --> rn:R02990

rn:R07996 { Liquiritigenin } <-- 2-Oxoglutarate -- rn:R00265 <-- Succinyl-CoA --> rn:R02990

rn:R07712 { Liquiritigenin } <-- 2-Oxoglutarate -- rn:R00265 <-- Succinyl-CoA --> rn:R03154

rn:R07996 { Liquiritigenin } <-- 2-Oxoglutarate -- rn:R00265 <-- Succinyl-CoA --> rn:R03154

rn:R07712 { Liquiritigenin } <-- 2-Oxoglutarate -- rn:R00265 <-- Succinyl-CoA --> rn:R09280

rn:R07996 { Liquiritigenin } <-- 2-Oxoglutarate -- rn:R00265 <-- Succinyl-CoA --> rn:R09280

rn:R03006 { Formononetin } <-- [Reduced NADPH---hemoprotein reductase] -- rn:R00311 <-- Fe2+ --> rn:R00310

rn:R03615 { Flavonoid } <-- [Reduced NADPH---hemoprotein reductase] -- rn:R00311 <-- Fe2+ --> rn:R00310

rn:R06560 { Formononetin } <-- [Reduced NADPH---hemoprotein reductase] -- rn:R00311 <-- Fe2+ --> rn:R00310

rn:R07198 { Liquiritigenin } <-- [Reduced NADPH---hemoprotein reductase] -- rn:R00311 <-- Fe2+ --> rn:R00310

rn:R07745 { Calycosin } <-- [Reduced NADPH---hemoprotein reductase] -- rn:R00311 <-- Fe2+ --> rn:R00310

rn:R07777 { Liquiritigenin } <-- [Reduced NADPH---hemoprotein reductase] -- rn:R00311 <-- Fe2+ --> rn:R00310

rn:R08002 { Liquiritigenin } <-- [Reduced NADPH---hemoprotein reductase] -- rn:R00311 <-- Fe2+ --> rn:R00310

rn:R03006 { Formononetin } <-- [Reduced NADPH---hemoprotein reductase] -- rn:R00311 <-- Biliverdin --> rn:R05817

rn:R03615 { Flavonoid } <-- [Reduced NADPH---hemoprotein reductase] -- rn:R00311 <-- Biliverdin --> rn:R05817

rn:R06560 { Formononetin } <-- [Reduced NADPH---hemoprotein reductase] -- rn:R00311 <-- Biliverdin --> rn:R05817

rn:R07198 { Liquiritigenin } <-- [Reduced NADPH---hemoprotein reductase] -- rn:R00311 <-- Biliverdin --> rn:R05817

rn:R07745 { Calycosin } <-- [Reduced NADPH---hemoprotein reductase] -- rn:R00311 <-- Biliverdin --> rn:R05817

rn:R07777 { Liquiritigenin } <-- [Reduced NADPH---hemoprotein reductase] -- rn:R00311 <-- Biliverdin --> rn:R05817

rn:R08002 { Liquiritigenin } <-- [Reduced NADPH---hemoprotein reductase] -- rn:R00311 <-- Biliverdin --> rn:R05817

rn:R03006 { Formononetin } <-- [Reduced NADPH---hemoprotein reductase] -- rn:R00311 <-- Biliverdin --> rn:R05818

rn:R03615 { Flavonoid } <-- [Reduced NADPH---hemoprotein reductase] -- rn:R00311 <-- Biliverdin --> rn:R05818

rn:R06560 { Formononetin } <-- [Reduced NADPH---hemoprotein reductase] -- rn:R00311 <-- Biliverdin --> rn:R05818

rn:R07198 { Liquiritigenin } <-- [Reduced NADPH---hemoprotein reductase] -- rn:R00311 <-- Biliverdin --> rn:R05818

rn:R07745 { Calycosin } <-- [Reduced NADPH---hemoprotein reductase] -- rn:R00311 <-- Biliverdin --> rn:R05818

rn:R07777 { Liquiritigenin } <-- [Reduced NADPH---hemoprotein reductase] -- rn:R00311 <-- Biliverdin --> rn:R05818

rn:R08002 { Liquiritigenin } <-- [Reduced NADPH---hemoprotein reductase] -- rn:R00311 <-- Biliverdin --> rn:R05818

rn:R03006 { Formononetin } <-- [Reduced NADPH---hemoprotein reductase] -- rn:R00311 <-- [Oxidized NADPH---hemoprotein reductase] --> rn:R08551

rn:R03615 { Flavonoid } <-- [Reduced NADPH---hemoprotein reductase] -- rn:R00311 <-- [Oxidized NADPH---hemoprotein reductase] --> rn:R08551

rn:R06560 { Formononetin } <-- [Reduced NADPH---hemoprotein reductase] -- rn:R00311 <-- [Oxidized NADPH---hemoprotein reductase] --> rn:R08551

rn:R07198 { Liquiritigenin } <-- [Reduced NADPH---hemoprotein reductase] -- rn:R00311 <-- [Oxidized NADPH---hemoprotein reductase] --> rn:R08551

rn:R07745 { Calycosin } <-- [Reduced NADPH---hemoprotein reductase] -- rn:R00311 <-- [Oxidized NADPH---hemoprotein reductase] --> rn:R08551

rn:R07777 { Liquiritigenin } <-- [Reduced NADPH---hemoprotein reductase] -- rn:R00311 <-- [Oxidized NADPH---hemoprotein reductase] --> rn:R08551

rn:R08002 { Liquiritigenin } <-- [Reduced NADPH---hemoprotein reductase] -- rn:R00311 <-- [Oxidized NADPH---hemoprotein reductase] --> rn:R08551

rn:R03006 { Formononetin } <-- [Reduced NADPH---hemoprotein reductase] -- rn:R00311 <-- Fe2+ --> rn:R09541

rn:R03615 { Flavonoid } <-- [Reduced NADPH---hemoprotein reductase] -- rn:R00311 <-- Fe2+ --> rn:R09541

rn:R06560 { Formononetin } <-- [Reduced NADPH---hemoprotein reductase] -- rn:R00311 <-- Fe2+ --> rn:R09541

rn:R07198 { Liquiritigenin } <-- [Reduced NADPH---hemoprotein reductase] -- rn:R00311 <-- Fe2+ --> rn:R09541

rn:R07745 { Calycosin } <-- [Reduced NADPH---hemoprotein reductase] -- rn:R00311 <-- Fe2+ --> rn:R09541

rn:R07777 { Liquiritigenin } <-- [Reduced NADPH---hemoprotein reductase] -- rn:R00311 <-- Fe2+ --> rn:R09541

rn:R08002 { Liquiritigenin } <-- [Reduced NADPH---hemoprotein reductase] -- rn:R00311 <-- Fe2+ --> rn:R09541

rn:R03006 { Formononetin } <-- [Reduced NADPH---hemoprotein reductase] -- rn:R00311 <-- CO --> rn:R11168

rn:R03615 { Flavonoid } <-- [Reduced NADPH---hemoprotein reductase] -- rn:R00311 <-- CO --> rn:R11168

rn:R06560 { Formononetin } <-- [Reduced NADPH---hemoprotein reductase] -- rn:R00311 <-- CO --> rn:R11168

rn:R07198 { Liquiritigenin } <-- [Reduced NADPH---hemoprotein reductase] -- rn:R00311 <-- CO --> rn:R11168

rn:R07745 { Calycosin } <-- [Reduced NADPH---hemoprotein reductase] -- rn:R00311 <-- CO --> rn:R11168

rn:R07777 { Liquiritigenin } <-- [Reduced NADPH---hemoprotein reductase] -- rn:R00311 <-- CO --> rn:R11168

rn:R08002 { Liquiritigenin } <-- [Reduced NADPH---hemoprotein reductase] -- rn:R00311 <-- CO --> rn:R11168

rn:R07712 { Liquiritigenin } <-- 2-Oxoglutarate -- rn:R00694 <-- L-Glutamate --> rn:R00114

rn:R07996 { Liquiritigenin } <-- 2-Oxoglutarate -- rn:R00694 <-- L-Glutamate --> rn:R00114

rn:R07712 { Liquiritigenin } <-- 2-Oxoglutarate -- rn:R00694 <-- L-Glutamate --> rn:R00248

rn:R07996 { Liquiritigenin } <-- 2-Oxoglutarate -- rn:R00694 <-- L-Glutamate --> rn:R00248

rn:R07712 { Liquiritigenin } <-- 2-Oxoglutarate -- rn:R00694 <-- L-Glutamate --> rn:R00254

rn:R07996 { Liquiritigenin } <-- 2-Oxoglutarate -- rn:R00694 <-- L-Glutamate --> rn:R00254

rn:R07712 { Liquiritigenin } <-- 2-Oxoglutarate -- rn:R00694 <-- Phenylpyruvate --> rn:R00695

rn:R07996 { Liquiritigenin } <-- 2-Oxoglutarate -- rn:R00694 <-- Phenylpyruvate --> rn:R00695

rn:R07712 { Liquiritigenin } <-- 2-Oxoglutarate -- rn:R00694 <-- L-Glutamate --> rn:R00894

rn:R07996 { Liquiritigenin } <-- 2-Oxoglutarate -- rn:R00694 <-- L-Glutamate --> rn:R00894

rn:R07712 { Liquiritigenin } <-- 2-Oxoglutarate -- rn:R00694 <-- Phenylpyruvate --> rn:R01372

rn:R07996 { Liquiritigenin } <-- 2-Oxoglutarate -- rn:R00694 <-- Phenylpyruvate --> rn:R01372

rn:R07712 { Liquiritigenin } <-- 2-Oxoglutarate -- rn:R00694 <-- Phenylpyruvate --> rn:R01375

rn:R07996 { Liquiritigenin } <-- 2-Oxoglutarate -- rn:R00694 <-- Phenylpyruvate --> rn:R01375

rn:R07712 { Liquiritigenin } <-- 2-Oxoglutarate -- rn:R00694 <-- Phenylpyruvate --> rn:R01376

rn:R07996 { Liquiritigenin } <-- 2-Oxoglutarate -- rn:R00694 <-- Phenylpyruvate --> rn:R01376

rn:R07712 { Liquiritigenin } <-- 2-Oxoglutarate -- rn:R00694 <-- Phenylpyruvate --> rn:R01377

rn:R07996 { Liquiritigenin } <-- 2-Oxoglutarate -- rn:R00694 <-- Phenylpyruvate --> rn:R01377

rn:R07712 { Liquiritigenin } <-- 2-Oxoglutarate -- rn:R00694 <-- Phenylpyruvate --> rn:R01378

rn:R07996 { Liquiritigenin } <-- 2-Oxoglutarate -- rn:R00694 <-- Phenylpyruvate --> rn:R01378

rn:R07712 { Liquiritigenin } <-- 2-Oxoglutarate -- rn:R00694 <-- L-Glutamate --> rn:R02287

rn:R07996 { Liquiritigenin } <-- 2-Oxoglutarate -- rn:R00694 <-- L-Glutamate --> rn:R02287

rn:R07712 { Liquiritigenin } <-- 2-Oxoglutarate -- rn:R00694 <-- L-Glutamate --> rn:R03189

rn:R07996 { Liquiritigenin } <-- 2-Oxoglutarate -- rn:R00694 <-- L-Glutamate --> rn:R03189

rn:R07712 { Liquiritigenin } <-- 2-Oxoglutarate -- rn:R00694 <-- L-Glutamate --> rn:R03970

rn:R07996 { Liquiritigenin } <-- 2-Oxoglutarate -- rn:R00694 <-- L-Glutamate --> rn:R03970

rn:R07712 { Liquiritigenin } <-- 2-Oxoglutarate -- rn:R00694 <-- L-Glutamate --> rn:R03971

rn:R07996 { Liquiritigenin } <-- 2-Oxoglutarate -- rn:R00694 <-- L-Glutamate --> rn:R03971

rn:R07712 { Liquiritigenin } <-- 2-Oxoglutarate -- rn:R00694 <-- L-Glutamate --> rn:R04051

rn:R07996 { Liquiritigenin } <-- 2-Oxoglutarate -- rn:R00694 <-- L-Glutamate --> rn:R04051

rn:R07712 { Liquiritigenin } <-- 2-Oxoglutarate -- rn:R00694 <-- L-Glutamate --> rn:R04776

rn:R07996 { Liquiritigenin } <-- 2-Oxoglutarate -- rn:R00694 <-- L-Glutamate --> rn:R04776

rn:R07712 { Liquiritigenin } <-- 2-Oxoglutarate -- rn:R00694 <-- L-Glutamate --> rn:R07643

rn:R07996 { Liquiritigenin } <-- 2-Oxoglutarate -- rn:R00694 <-- L-Glutamate --> rn:R07643

rn:R07712 { Liquiritigenin } <-- 2-Oxoglutarate -- rn:R00694 <-- Phenylpyruvate --> rn:R10431

rn:R07996 { Liquiritigenin } <-- 2-Oxoglutarate -- rn:R00694 <-- Phenylpyruvate --> rn:R10431

rn:R07712 { Liquiritigenin } <-- 2-Oxoglutarate -- rn:R00694 <-- Phenylpyruvate --> rn:R10500

rn:R07996 { Liquiritigenin } <-- 2-Oxoglutarate -- rn:R00694 <-- Phenylpyruvate --> rn:R10500

rn:R03006 { Formononetin } <-- [Reduced NADPH---hemoprotein reductase] -- rn:R00730 <-- N-Hydroxy-L-tyrosine --> rn:R04460

rn:R03615 { Flavonoid } <-- [Reduced NADPH---hemoprotein reductase] -- rn:R00730 <-- N-Hydroxy-L-tyrosine --> rn:R04460

rn:R06560 { Formononetin } <-- [Reduced NADPH---hemoprotein reductase] -- rn:R00730 <-- N-Hydroxy-L-tyrosine --> rn:R04460

rn:R07198 { Liquiritigenin } <-- [Reduced NADPH---hemoprotein reductase] -- rn:R00730 <-- N-Hydroxy-L-tyrosine --> rn:R04460

rn:R07745 { Calycosin } <-- [Reduced NADPH---hemoprotein reductase] -- rn:R00730 <-- N-Hydroxy-L-tyrosine --> rn:R04460

rn:R07777 { Liquiritigenin } <-- [Reduced NADPH---hemoprotein reductase] -- rn:R00730 <-- N-Hydroxy-L-tyrosine --> rn:R04460

rn:R08002 { Liquiritigenin } <-- [Reduced NADPH---hemoprotein reductase] -- rn:R00730 <-- N-Hydroxy-L-tyrosine --> rn:R04460

rn:R03006 { Formononetin } <-- [Reduced NADPH---hemoprotein reductase] -- rn:R00730 <-- [Oxidized NADPH---hemoprotein reductase] --> rn:R08551

rn:R03615 { Flavonoid } <-- [Reduced NADPH---hemoprotein reductase] -- rn:R00730 <-- [Oxidized NADPH---hemoprotein reductase] --> rn:R08551

rn:R06560 { Formononetin } <-- [Reduced NADPH---hemoprotein reductase] -- rn:R00730 <-- [Oxidized NADPH---hemoprotein reductase] --> rn:R08551

rn:R07198 { Liquiritigenin } <-- [Reduced NADPH---hemoprotein reductase] -- rn:R00730 <-- [Oxidized NADPH---hemoprotein reductase] --> rn:R08551

rn:R07745 { Calycosin } <-- [Reduced NADPH---hemoprotein reductase] -- rn:R00730 <-- [Oxidized NADPH---hemoprotein reductase] --> rn:R08551

rn:R07777 { Liquiritigenin } <-- [Reduced NADPH---hemoprotein reductase] -- rn:R00730 <-- [Oxidized NADPH---hemoprotein reductase] --> rn:R08551

rn:R08002 { Liquiritigenin } <-- [Reduced NADPH---hemoprotein reductase] -- rn:R00730 <-- [Oxidized NADPH---hemoprotein reductase] --> rn:R08551

rn:R07712 { Liquiritigenin } <-- 2-Oxoglutarate -- rn:R01161 <-- L-Glutamate --> rn:R00114

rn:R07996 { Liquiritigenin } <-- 2-Oxoglutarate -- rn:R01161 <-- L-Glutamate --> rn:R00114

rn:R07712 { Liquiritigenin } <-- 2-Oxoglutarate -- rn:R01161 <-- L-Glutamate --> rn:R00248

rn:R07996 { Liquiritigenin } <-- 2-Oxoglutarate -- rn:R01161 <-- L-Glutamate --> rn:R00248

rn:R07712 { Liquiritigenin } <-- 2-Oxoglutarate -- rn:R01161 <-- L-Glutamate --> rn:R00254

rn:R07996 { Liquiritigenin } <-- 2-Oxoglutarate -- rn:R01161 <-- L-Glutamate --> rn:R00254

rn:R07712 { Liquiritigenin } <-- 2-Oxoglutarate -- rn:R01161 <-- L-Glutamate --> rn:R00894

rn:R07996 { Liquiritigenin } <-- 2-Oxoglutarate -- rn:R01161 <-- L-Glutamate --> rn:R00894

rn:R07712 { Liquiritigenin } <-- 2-Oxoglutarate -- rn:R01161 <-- L-Glutamate --> rn:R02287

rn:R07996 { Liquiritigenin } <-- 2-Oxoglutarate -- rn:R01161 <-- L-Glutamate --> rn:R02287

rn:R07712 { Liquiritigenin } <-- 2-Oxoglutarate -- rn:R01161 <-- L-Glutamate --> rn:R03189

rn:R07996 { Liquiritigenin } <-- 2-Oxoglutarate -- rn:R01161 <-- L-Glutamate --> rn:R03189

rn:R07712 { Liquiritigenin } <-- 2-Oxoglutarate -- rn:R01161 <-- L-Glutamate --> rn:R03970

rn:R07996 { Liquiritigenin } <-- 2-Oxoglutarate -- rn:R01161 <-- L-Glutamate --> rn:R03970

rn:R07712 { Liquiritigenin } <-- 2-Oxoglutarate -- rn:R01161 <-- L-Glutamate --> rn:R03971

rn:R07996 { Liquiritigenin } <-- 2-Oxoglutarate -- rn:R01161 <-- L-Glutamate --> rn:R03971

rn:R07712 { Liquiritigenin } <-- 2-Oxoglutarate -- rn:R01161 <-- L-Glutamate --> rn:R04051

rn:R07996 { Liquiritigenin } <-- 2-Oxoglutarate -- rn:R01161 <-- L-Glutamate --> rn:R04051

rn:R07712 { Liquiritigenin } <-- 2-Oxoglutarate -- rn:R01161 <-- L-Glutamate --> rn:R04776

rn:R07996 { Liquiritigenin } <-- 2-Oxoglutarate -- rn:R01161 <-- L-Glutamate --> rn:R04776

rn:R07712 { Liquiritigenin } <-- 2-Oxoglutarate -- rn:R01161 <-- Imidazol-5-yl-pyruvate --> rn:R04874

rn:R07996 { Liquiritigenin } <-- 2-Oxoglutarate -- rn:R01161 <-- Imidazol-5-yl-pyruvate --> rn:R04874

rn:R07712 { Liquiritigenin } <-- 2-Oxoglutarate -- rn:R01161 <-- Imidazol-5-yl-pyruvate --> rn:R04876

rn:R07996 { Liquiritigenin } <-- 2-Oxoglutarate -- rn:R01161 <-- Imidazol-5-yl-pyruvate --> rn:R04876

rn:R07712 { Liquiritigenin } <-- 2-Oxoglutarate -- rn:R01161 <-- L-Glutamate --> rn:R07643

rn:R07996 { Liquiritigenin } <-- 2-Oxoglutarate -- rn:R01161 <-- L-Glutamate --> rn:R07643

rn:R03006 { Formononetin } <-- [Reduced NADPH---hemoprotein reductase] -- rn:R01295 <-- 4-Hydroxybenzoate --> rn:R01238

rn:R03615 { Flavonoid } <-- [Reduced NADPH---hemoprotein reductase] -- rn:R01295 <-- 4-Hydroxybenzoate --> rn:R01238

rn:R06560 { Formononetin } <-- [Reduced NADPH---hemoprotein reductase] -- rn:R01295 <-- 4-Hydroxybenzoate --> rn:R01238

rn:R07198 { Liquiritigenin } <-- [Reduced NADPH---hemoprotein reductase] -- rn:R01295 <-- 4-Hydroxybenzoate --> rn:R01238

rn:R07745 { Calycosin } <-- [Reduced NADPH---hemoprotein reductase] -- rn:R01295 <-- 4-Hydroxybenzoate --> rn:R01238

rn:R07777 { Liquiritigenin } <-- [Reduced NADPH---hemoprotein reductase] -- rn:R01295 <-- 4-Hydroxybenzoate --> rn:R01238

rn:R08002 { Liquiritigenin } <-- [Reduced NADPH---hemoprotein reductase] -- rn:R01295 <-- 4-Hydroxybenzoate --> rn:R01238

rn:R03006 { Formononetin } <-- [Reduced NADPH---hemoprotein reductase] -- rn:R01295 <-- 4-Hydroxybenzoate --> rn:R01296

rn:R03615 { Flavonoid } <-- [Reduced NADPH---hemoprotein reductase] -- rn:R01295 <-- 4-Hydroxybenzoate --> rn:R01296

rn:R06560 { Formononetin } <-- [Reduced NADPH---hemoprotein reductase] -- rn:R01295 <-- 4-Hydroxybenzoate --> rn:R01296

rn:R07198 { Liquiritigenin } <-- [Reduced NADPH---hemoprotein reductase] -- rn:R01295 <-- 4-Hydroxybenzoate --> rn:R01296

rn:R07745 { Calycosin } <-- [Reduced NADPH---hemoprotein reductase] -- rn:R01295 <-- 4-Hydroxybenzoate --> rn:R01296

rn:R07777 { Liquiritigenin } <-- [Reduced NADPH---hemoprotein reductase] -- rn:R01295 <-- 4-Hydroxybenzoate --> rn:R01296

rn:R08002 { Liquiritigenin } <-- [Reduced NADPH---hemoprotein reductase] -- rn:R01295 <-- 4-Hydroxybenzoate --> rn:R01296

rn:R03006 { Formononetin } <-- [Reduced NADPH---hemoprotein reductase] -- rn:R01295 <-- 4-Hydroxybenzoate --> rn:R01297

rn:R03615 { Flavonoid } <-- [Reduced NADPH---hemoprotein reductase] -- rn:R01295 <-- 4-Hydroxybenzoate --> rn:R01297

rn:R06560 { Formononetin } <-- [Reduced NADPH---hemoprotein reductase] -- rn:R01295 <-- 4-Hydroxybenzoate --> rn:R01297

rn:R07198 { Liquiritigenin } <-- [Reduced NADPH---hemoprotein reductase] -- rn:R01295 <-- 4-Hydroxybenzoate --> rn:R01297

rn:R07745 { Calycosin } <-- [Reduced NADPH---hemoprotein reductase] -- rn:R01295 <-- 4-Hydroxybenzoate --> rn:R01297

rn:R07777 { Liquiritigenin } <-- [Reduced NADPH---hemoprotein reductase] -- rn:R01295 <-- 4-Hydroxybenzoate --> rn:R01297

rn:R08002 { Liquiritigenin } <-- [Reduced NADPH---hemoprotein reductase] -- rn:R01295 <-- 4-Hydroxybenzoate --> rn:R01297

rn:R03006 { Formononetin } <-- [Reduced NADPH---hemoprotein reductase] -- rn:R01295 <-- 4-Hydroxybenzoate --> rn:R01298

rn:R03615 { Flavonoid } <-- [Reduced NADPH---hemoprotein reductase] -- rn:R01295 <-- 4-Hydroxybenzoate --> rn:R01298

rn:R06560 { Formononetin } <-- [Reduced NADPH---hemoprotein reductase] -- rn:R01295 <-- 4-Hydroxybenzoate --> rn:R01298

rn:R07198 { Liquiritigenin } <-- [Reduced NADPH---hemoprotein reductase] -- rn:R01295 <-- 4-Hydroxybenzoate --> rn:R01298

rn:R07745 { Calycosin } <-- [Reduced NADPH---hemoprotein reductase] -- rn:R01295 <-- 4-Hydroxybenzoate --> rn:R01298

rn:R07777 { Liquiritigenin } <-- [Reduced NADPH---hemoprotein reductase] -- rn:R01295 <-- 4-Hydroxybenzoate --> rn:R01298

rn:R08002 { Liquiritigenin } <-- [Reduced NADPH---hemoprotein reductase] -- rn:R01295 <-- 4-Hydroxybenzoate --> rn:R01298

rn:R03006 { Formononetin } <-- [Reduced NADPH---hemoprotein reductase] -- rn:R01295 <-- 4-Hydroxybenzoate --> rn:R01299

rn:R03615 { Flavonoid } <-- [Reduced NADPH---hemoprotein reductase] -- rn:R01295 <-- 4-Hydroxybenzoate --> rn:R01299

rn:R06560 { Formononetin } <-- [Reduced NADPH---hemoprotein reductase] -- rn:R01295 <-- 4-Hydroxybenzoate --> rn:R01299

rn:R07198 { Liquiritigenin } <-- [Reduced NADPH---hemoprotein reductase] -- rn:R01295 <-- 4-Hydroxybenzoate --> rn:R01299

rn:R07745 { Calycosin } <-- [Reduced NADPH---hemoprotein reductase] -- rn:R01295 <-- 4-Hydroxybenzoate --> rn:R01299

rn:R07777 { Liquiritigenin } <-- [Reduced NADPH---hemoprotein reductase] -- rn:R01295 <-- 4-Hydroxybenzoate --> rn:R01299

rn:R08002 { Liquiritigenin } <-- [Reduced NADPH---hemoprotein reductase] -- rn:R01295 <-- 4-Hydroxybenzoate --> rn:R01299

rn:R03006 { Formononetin } <-- [Reduced NADPH---hemoprotein reductase] -- rn:R01295 <-- 4-Hydroxybenzoate --> rn:R01300

rn:R03615 { Flavonoid } <-- [Reduced NADPH---hemoprotein reductase] -- rn:R01295 <-- 4-Hydroxybenzoate --> rn:R01300

rn:R06560 { Formononetin } <-- [Reduced NADPH---hemoprotein reductase] -- rn:R01295 <-- 4-Hydroxybenzoate --> rn:R01300

rn:R07198 { Liquiritigenin } <-- [Reduced NADPH---hemoprotein reductase] -- rn:R01295 <-- 4-Hydroxybenzoate --> rn:R01300

rn:R07745 { Calycosin } <-- [Reduced NADPH---hemoprotein reductase] -- rn:R01295 <-- 4-Hydroxybenzoate --> rn:R01300

rn:R07777 { Liquiritigenin } <-- [Reduced NADPH---hemoprotein reductase] -- rn:R01295 <-- 4-Hydroxybenzoate --> rn:R01300

rn:R08002 { Liquiritigenin } <-- [Reduced NADPH---hemoprotein reductase] -- rn:R01295 <-- 4-Hydroxybenzoate --> rn:R01300

rn:R03006 { Formononetin } <-- [Reduced NADPH---hemoprotein reductase] -- rn:R01295 <-- 4-Hydroxybenzoate --> rn:R01302

rn:R03615 { Flavonoid } <-- [Reduced NADPH---hemoprotein reductase] -- rn:R01295 <-- 4-Hydroxybenzoate --> rn:R01302

rn:R06560 { Formononetin } <-- [Reduced NADPH---hemoprotein reductase] -- rn:R01295 <-- 4-Hydroxybenzoate --> rn:R01302

rn:R07198 { Liquiritigenin } <-- [Reduced NADPH---hemoprotein reductase] -- rn:R01295 <-- 4-Hydroxybenzoate --> rn:R01302

rn:R07745 { Calycosin } <-- [Reduced NADPH---hemoprotein reductase] -- rn:R01295 <-- 4-Hydroxybenzoate --> rn:R01302

rn:R07777 { Liquiritigenin } <-- [Reduced NADPH---hemoprotein reductase] -- rn:R01295 <-- 4-Hydroxybenzoate --> rn:R01302

rn:R08002 { Liquiritigenin } <-- [Reduced NADPH---hemoprotein reductase] -- rn:R01295 <-- 4-Hydroxybenzoate --> rn:R01302

rn:R03006 { Formononetin } <-- [Reduced NADPH---hemoprotein reductase] -- rn:R01295 <-- 4-Hydroxybenzoate --> rn:R01303

rn:R03615 { Flavonoid } <-- [Reduced NADPH---hemoprotein reductase] -- rn:R01295 <-- 4-Hydroxybenzoate --> rn:R01303

rn:R06560 { Formononetin } <-- [Reduced NADPH---hemoprotein reductase] -- rn:R01295 <-- 4-Hydroxybenzoate --> rn:R01303

rn:R07198 { Liquiritigenin } <-- [Reduced NADPH---hemoprotein reductase] -- rn:R01295 <-- 4-Hydroxybenzoate --> rn:R01303

rn:R07745 { Calycosin } <-- [Reduced NADPH---hemoprotein reductase] -- rn:R01295 <-- 4-Hydroxybenzoate --> rn:R01303

rn:R07777 { Liquiritigenin } <-- [Reduced NADPH---hemoprotein reductase] -- rn:R01295 <-- 4-Hydroxybenzoate --> rn:R01303

rn:R08002 { Liquiritigenin } <-- [Reduced NADPH---hemoprotein reductase] -- rn:R01295 <-- 4-Hydroxybenzoate --> rn:R01303

rn:R03006 { Formononetin } <-- [Reduced NADPH---hemoprotein reductase] -- rn:R01295 <-- 4-Hydroxybenzoate --> rn:R01304

rn:R03615 { Flavonoid } <-- [Reduced NADPH---hemoprotein reductase] -- rn:R01295 <-- 4-Hydroxybenzoate --> rn:R01304

rn:R06560 { Formononetin } <-- [Reduced NADPH---hemoprotein reductase] -- rn:R01295 <-- 4-Hydroxybenzoate --> rn:R01304

rn:R07198 { Liquiritigenin } <-- [Reduced NADPH---hemoprotein reductase] -- rn:R01295 <-- 4-Hydroxybenzoate --> rn:R01304

rn:R07745 { Calycosin } <-- [Reduced NADPH---hemoprotein reductase] -- rn:R01295 <-- 4-Hydroxybenzoate --> rn:R01304

rn:R07777 { Liquiritigenin } <-- [Reduced NADPH---hemoprotein reductase] -- rn:R01295 <-- 4-Hydroxybenzoate --> rn:R01304

rn:R08002 { Liquiritigenin } <-- [Reduced NADPH---hemoprotein reductase] -- rn:R01295 <-- 4-Hydroxybenzoate --> rn:R01304

rn:R03006 { Formononetin } <-- [Reduced NADPH---hemoprotein reductase] -- rn:R01295 <-- 4-Hydroxybenzoate --> rn:R01308

rn:R03615 { Flavonoid } <-- [Reduced NADPH---hemoprotein reductase] -- rn:R01295 <-- 4-Hydroxybenzoate --> rn:R01308

rn:R06560 { Formononetin } <-- [Reduced NADPH---hemoprotein reductase] -- rn:R01295 <-- 4-Hydroxybenzoate --> rn:R01308

rn:R07198 { Liquiritigenin } <-- [Reduced NADPH---hemoprotein reductase] -- rn:R01295 <-- 4-Hydroxybenzoate --> rn:R01308

rn:R07745 { Calycosin } <-- [Reduced NADPH---hemoprotein reductase] -- rn:R01295 <-- 4-Hydroxybenzoate --> rn:R01308

rn:R07777 { Liquiritigenin } <-- [Reduced NADPH---hemoprotein reductase] -- rn:R01295 <-- 4-Hydroxybenzoate --> rn:R01308

rn:R08002 { Liquiritigenin } <-- [Reduced NADPH---hemoprotein reductase] -- rn:R01295 <-- 4-Hydroxybenzoate --> rn:R01308

rn:R03006 { Formononetin } <-- [Reduced NADPH---hemoprotein reductase] -- rn:R01295 <-- 4-Hydroxybenzoate --> rn:R05000

rn:R03615 { Flavonoid } <-- [Reduced NADPH---hemoprotein reductase] -- rn:R01295 <-- 4-Hydroxybenzoate --> rn:R05000

rn:R06560 { Formononetin } <-- [Reduced NADPH---hemoprotein reductase] -- rn:R01295 <-- 4-Hydroxybenzoate --> rn:R05000

rn:R07198 { Liquiritigenin } <-- [Reduced NADPH---hemoprotein reductase] -- rn:R01295 <-- 4-Hydroxybenzoate --> rn:R05000

rn:R07745 { Calycosin } <-- [Reduced NADPH---hemoprotein reductase] -- rn:R01295 <-- 4-Hydroxybenzoate --> rn:R05000

rn:R07777 { Liquiritigenin } <-- [Reduced NADPH---hemoprotein reductase] -- rn:R01295 <-- 4-Hydroxybenzoate --> rn:R05000

rn:R08002 { Liquiritigenin } <-- [Reduced NADPH---hemoprotein reductase] -- rn:R01295 <-- 4-Hydroxybenzoate --> rn:R05000

rn:R03006 { Formononetin } <-- [Reduced NADPH---hemoprotein reductase] -- rn:R01295 <-- 4-Hydroxybenzoate --> rn:R05615

rn:R03615 { Flavonoid } <-- [Reduced NADPH---hemoprotein reductase] -- rn:R01295 <-- 4-Hydroxybenzoate --> rn:R05615

rn:R06560 { Formononetin } <-- [Reduced NADPH---hemoprotein reductase] -- rn:R01295 <-- 4-Hydroxybenzoate --> rn:R05615

rn:R07198 { Liquiritigenin } <-- [Reduced NADPH---hemoprotein reductase] -- rn:R01295 <-- 4-Hydroxybenzoate --> rn:R05615

rn:R07745 { Calycosin } <-- [Reduced NADPH---hemoprotein reductase] -- rn:R01295 <-- 4-Hydroxybenzoate --> rn:R05615

rn:R07777 { Liquiritigenin } <-- [Reduced NADPH---hemoprotein reductase] -- rn:R01295 <-- 4-Hydroxybenzoate --> rn:R05615

rn:R08002 { Liquiritigenin } <-- [Reduced NADPH---hemoprotein reductase] -- rn:R01295 <-- 4-Hydroxybenzoate --> rn:R05615

rn:R03006 { Formononetin } <-- [Reduced NADPH---hemoprotein reductase] -- rn:R01295 <-- 4-Hydroxybenzoate --> rn:R05616

rn:R03615 { Flavonoid } <-- [Reduced NADPH---hemoprotein reductase] -- rn:R01295 <-- 4-Hydroxybenzoate --> rn:R05616

rn:R06560 { Formononetin } <-- [Reduced NADPH---hemoprotein reductase] -- rn:R01295 <-- 4-Hydroxybenzoate --> rn:R05616

rn:R07198 { Liquiritigenin } <-- [Reduced NADPH---hemoprotein reductase] -- rn:R01295 <-- 4-Hydroxybenzoate --> rn:R05616

rn:R07745 { Calycosin } <-- [Reduced NADPH---hemoprotein reductase] -- rn:R01295 <-- 4-Hydroxybenzoate --> rn:R05616

rn:R07777 { Liquiritigenin } <-- [Reduced NADPH---hemoprotein reductase] -- rn:R01295 <-- 4-Hydroxybenzoate --> rn:R05616

rn:R08002 { Liquiritigenin } <-- [Reduced NADPH---hemoprotein reductase] -- rn:R01295 <-- 4-Hydroxybenzoate --> rn:R05616

rn:R03006 { Formononetin } <-- [Reduced NADPH---hemoprotein reductase] -- rn:R01295 <-- 4-Hydroxybenzoate --> rn:R07273

rn:R03615 { Flavonoid } <-- [Reduced NADPH---hemoprotein reductase] -- rn:R01295 <-- 4-Hydroxybenzoate --> rn:R07273

rn:R06560 { Formononetin } <-- [Reduced NADPH---hemoprotein reductase] -- rn:R01295 <-- 4-Hydroxybenzoate --> rn:R07273

rn:R07198 { Liquiritigenin } <-- [Reduced NADPH---hemoprotein reductase] -- rn:R01295 <-- 4-Hydroxybenzoate --> rn:R07273

rn:R07745 { Calycosin } <-- [Reduced NADPH---hemoprotein reductase] -- rn:R01295 <-- 4-Hydroxybenzoate --> rn:R07273

rn:R07777 { Liquiritigenin } <-- [Reduced NADPH---hemoprotein reductase] -- rn:R01295 <-- 4-Hydroxybenzoate --> rn:R07273

rn:R08002 { Liquiritigenin } <-- [Reduced NADPH---hemoprotein reductase] -- rn:R01295 <-- 4-Hydroxybenzoate --> rn:R07273

rn:R03006 { Formononetin } <-- [Reduced NADPH---hemoprotein reductase] -- rn:R01295 <-- [Oxidized NADPH---hemoprotein reductase] --> rn:R08551

rn:R03615 { Flavonoid } <-- [Reduced NADPH---hemoprotein reductase] -- rn:R01295 <-- [Oxidized NADPH---hemoprotein reductase] --> rn:R08551

rn:R06560 { Formononetin } <-- [Reduced NADPH---hemoprotein reductase] -- rn:R01295 <-- [Oxidized NADPH---hemoprotein reductase] --> rn:R08551

rn:R07198 { Liquiritigenin } <-- [Reduced NADPH---hemoprotein reductase] -- rn:R01295 <-- [Oxidized NADPH---hemoprotein reductase] --> rn:R08551

rn:R07745 { Calycosin } <-- [Reduced NADPH---hemoprotein reductase] -- rn:R01295 <-- [Oxidized NADPH---hemoprotein reductase] --> rn:R08551

rn:R07777 { Liquiritigenin } <-- [Reduced NADPH---hemoprotein reductase] -- rn:R01295 <-- [Oxidized NADPH---hemoprotein reductase] --> rn:R08551

rn:R08002 { Liquiritigenin } <-- [Reduced NADPH---hemoprotein reductase] -- rn:R01295 <-- [Oxidized NADPH---hemoprotein reductase] --> rn:R08551

rn:R03006 { Formononetin } <-- [Reduced NADPH---hemoprotein reductase] -- rn:R01295 <-- 4-Hydroxybenzoate --> rn:R09040

rn:R03615 { Flavonoid } <-- [Reduced NADPH---hemoprotein reductase] -- rn:R01295 <-- 4-Hydroxybenzoate --> rn:R09040

rn:R06560 { Formononetin } <-- [Reduced NADPH---hemoprotein reductase] -- rn:R01295 <-- 4-Hydroxybenzoate --> rn:R09040

rn:R07198 { Liquiritigenin } <-- [Reduced NADPH---hemoprotein reductase] -- rn:R01295 <-- 4-Hydroxybenzoate --> rn:R09040

rn:R07745 { Calycosin } <-- [Reduced NADPH---hemoprotein reductase] -- rn:R01295 <-- 4-Hydroxybenzoate --> rn:R09040

rn:R07777 { Liquiritigenin } <-- [Reduced NADPH---hemoprotein reductase] -- rn:R01295 <-- 4-Hydroxybenzoate --> rn:R09040

rn:R08002 { Liquiritigenin } <-- [Reduced NADPH---hemoprotein reductase] -- rn:R01295 <-- 4-Hydroxybenzoate --> rn:R09040

rn:R03006 { Formononetin } <-- [Reduced NADPH---hemoprotein reductase] -- rn:R01295 <-- 4-Hydroxybenzoate --> rn:R11102

rn:R03615 { Flavonoid } <-- [Reduced NADPH---hemoprotein reductase] -- rn:R01295 <-- 4-Hydroxybenzoate --> rn:R11102

rn:R06560 { Formononetin } <-- [Reduced NADPH---hemoprotein reductase] -- rn:R01295 <-- 4-Hydroxybenzoate --> rn:R11102

rn:R07198 { Liquiritigenin } <-- [Reduced NADPH---hemoprotein reductase] -- rn:R01295 <-- 4-Hydroxybenzoate --> rn:R11102

rn:R07745 { Calycosin } <-- [Reduced NADPH---hemoprotein reductase] -- rn:R01295 <-- 4-Hydroxybenzoate --> rn:R11102

rn:R07777 { Liquiritigenin } <-- [Reduced NADPH---hemoprotein reductase] -- rn:R01295 <-- 4-Hydroxybenzoate --> rn:R11102

rn:R08002 { Liquiritigenin } <-- [Reduced NADPH---hemoprotein reductase] -- rn:R01295 <-- 4-Hydroxybenzoate --> rn:R11102

rn:R03006 { Formononetin } <-- [Reduced NADPH---hemoprotein reductase] -- rn:R01295 <-- 4-Hydroxybenzoate --> rn:R11608

rn:R03615 { Flavonoid } <-- [Reduced NADPH---hemoprotein reductase] -- rn:R01295 <-- 4-Hydroxybenzoate --> rn:R11608

rn:R06560 { Formononetin } <-- [Reduced NADPH---hemoprotein reductase] -- rn:R01295 <-- 4-Hydroxybenzoate --> rn:R11608

rn:R07198 { Liquiritigenin } <-- [Reduced NADPH---hemoprotein reductase] -- rn:R01295 <-- 4-Hydroxybenzoate --> rn:R11608

rn:R07745 { Calycosin } <-- [Reduced NADPH---hemoprotein reductase] -- rn:R01295 <-- 4-Hydroxybenzoate --> rn:R11608

rn:R07777 { Liquiritigenin } <-- [Reduced NADPH---hemoprotein reductase] -- rn:R01295 <-- 4-Hydroxybenzoate --> rn:R11608

rn:R08002 { Liquiritigenin } <-- [Reduced NADPH---hemoprotein reductase] -- rn:R01295 <-- 4-Hydroxybenzoate --> rn:R11608

rn:R03006 { Formononetin } <-- [Reduced NADPH---hemoprotein reductase] -- rn:R01295 <-- 4-Hydroxybenzoate --> rn:R11872

rn:R03615 { Flavonoid } <-- [Reduced NADPH---hemoprotein reductase] -- rn:R01295 <-- 4-Hydroxybenzoate --> rn:R11872

rn:R06560 { Formononetin } <-- [Reduced NADPH---hemoprotein reductase] -- rn:R01295 <-- 4-Hydroxybenzoate --> rn:R11872

rn:R07198 { Liquiritigenin } <-- [Reduced NADPH---hemoprotein reductase] -- rn:R01295 <-- 4-Hydroxybenzoate --> rn:R11872

rn:R07745 { Calycosin } <-- [Reduced NADPH---hemoprotein reductase] -- rn:R01295 <-- 4-Hydroxybenzoate --> rn:R11872

rn:R07777 { Liquiritigenin } <-- [Reduced NADPH---hemoprotein reductase] -- rn:R01295 <-- 4-Hydroxybenzoate --> rn:R11872

rn:R08002 { Liquiritigenin } <-- [Reduced NADPH---hemoprotein reductase] -- rn:R01295 <-- 4-Hydroxybenzoate --> rn:R11872

rn:R03006 { Formononetin } <-- [Reduced NADPH---hemoprotein reductase] -- rn:R01295 <-- 4-Hydroxybenzoate --> rn:R12013

rn:R03615 { Flavonoid } <-- [Reduced NADPH---hemoprotein reductase] -- rn:R01295 <-- 4-Hydroxybenzoate --> rn:R12013

rn:R06560 { Formononetin } <-- [Reduced NADPH---hemoprotein reductase] -- rn:R01295 <-- 4-Hydroxybenzoate --> rn:R12013

rn:R07198 { Liquiritigenin } <-- [Reduced NADPH---hemoprotein reductase] -- rn:R01295 <-- 4-Hydroxybenzoate --> rn:R12013

rn:R07745 { Calycosin } <-- [Reduced NADPH---hemoprotein reductase] -- rn:R01295 <-- 4-Hydroxybenzoate --> rn:R12013

rn:R07777 { Liquiritigenin } <-- [Reduced NADPH---hemoprotein reductase] -- rn:R01295 <-- 4-Hydroxybenzoate --> rn:R12013

rn:R08002 { Liquiritigenin } <-- [Reduced NADPH---hemoprotein reductase] -- rn:R01295 <-- 4-Hydroxybenzoate --> rn:R12013

rn:R03006 { Formononetin } <-- [Reduced NADPH---hemoprotein reductase] -- rn:R01295 <-- 4-Hydroxybenzoate --> rn:R12015

rn:R03615 { Flavonoid } <-- [Reduced NADPH---hemoprotein reductase] -- rn:R01295 <-- 4-Hydroxybenzoate --> rn:R12015

rn:R06560 { Formononetin } <-- [Reduced NADPH---hemoprotein reductase] -- rn:R01295 <-- 4-Hydroxybenzoate --> rn:R12015

rn:R07198 { Liquiritigenin } <-- [Reduced NADPH---hemoprotein reductase] -- rn:R01295 <-- 4-Hydroxybenzoate --> rn:R12015

rn:R07745 { Calycosin } <-- [Reduced NADPH---hemoprotein reductase] -- rn:R01295 <-- 4-Hydroxybenzoate --> rn:R12015

rn:R07777 { Liquiritigenin } <-- [Reduced NADPH---hemoprotein reductase] -- rn:R01295 <-- 4-Hydroxybenzoate --> rn:R12015

rn:R08002 { Liquiritigenin } <-- [Reduced NADPH---hemoprotein reductase] -- rn:R01295 <-- 4-Hydroxybenzoate --> rn:R12015

rn:R03548 { 7,8-Dihydroxycoumarin } <-- UDP-glucose -- rn:R01304 <-- 4-(beta-D-Glucosyloxy)benzoate --> rn:R09039

rn:R07729 { Formononetin } <-- UDP-glucose -- rn:R01304 <-- 4-(beta-D-Glucosyloxy)benzoate --> rn:R09039

rn:R03006 { Formononetin } <-- [Reduced NADPH---hemoprotein reductase] -- rn:R01348 <-- [Oxidized NADPH---hemoprotein reductase] --> rn:R08551

rn:R03615 { Flavonoid } <-- [Reduced NADPH---hemoprotein reductase] -- rn:R01348 <-- [Oxidized NADPH---hemoprotein reductase] --> rn:R08551

rn:R06560 { Formononetin } <-- [Reduced NADPH---hemoprotein reductase] -- rn:R01348 <-- [Oxidized NADPH---hemoprotein reductase] --> rn:R08551

rn:R07198 { Liquiritigenin } <-- [Reduced NADPH---hemoprotein reductase] -- rn:R01348 <-- [Oxidized NADPH---hemoprotein reductase] --> rn:R08551

rn:R07745 { Calycosin } <-- [Reduced NADPH---hemoprotein reductase] -- rn:R01348 <-- [Oxidized NADPH---hemoprotein reductase] --> rn:R08551

rn:R07777 { Liquiritigenin } <-- [Reduced NADPH---hemoprotein reductase] -- rn:R01348 <-- [Oxidized NADPH---hemoprotein reductase] --> rn:R08551

rn:R08002 { Liquiritigenin } <-- [Reduced NADPH---hemoprotein reductase] -- rn:R01348 <-- [Oxidized NADPH---hemoprotein reductase] --> rn:R08551

rn:R07712 { Liquiritigenin } <-- 2-Oxoglutarate -- rn:R01580 <-- Pyridoxal phosphate --> rn:R00173

rn:R07996 { Liquiritigenin } <-- 2-Oxoglutarate -- rn:R01580 <-- Pyridoxal phosphate --> rn:R00173

rn:R07712 { Liquiritigenin } <-- 2-Oxoglutarate -- rn:R01582 <-- Phenylpyruvate --> rn:R00695

rn:R07996 { Liquiritigenin } <-- 2-Oxoglutarate -- rn:R01582 <-- Phenylpyruvate --> rn:R00695

rn:R07712 { Liquiritigenin } <-- 2-Oxoglutarate -- rn:R01582 <-- Phenylpyruvate --> rn:R01372

rn:R07996 { Liquiritigenin } <-- 2-Oxoglutarate -- rn:R01582 <-- Phenylpyruvate --> rn:R01372

rn:R07712 { Liquiritigenin } <-- 2-Oxoglutarate -- rn:R01582 <-- Phenylpyruvate --> rn:R01375

rn:R07996 { Liquiritigenin } <-- 2-Oxoglutarate -- rn:R01582 <-- Phenylpyruvate --> rn:R01375

rn:R07712 { Liquiritigenin } <-- 2-Oxoglutarate -- rn:R01582 <-- Phenylpyruvate --> rn:R01376

rn:R07996 { Liquiritigenin } <-- 2-Oxoglutarate -- rn:R01582 <-- Phenylpyruvate --> rn:R01376

rn:R07712 { Liquiritigenin } <-- 2-Oxoglutarate -- rn:R01582 <-- Phenylpyruvate --> rn:R01377

rn:R07996 { Liquiritigenin } <-- 2-Oxoglutarate -- rn:R01582 <-- Phenylpyruvate --> rn:R01377

rn:R07712 { Liquiritigenin } <-- 2-Oxoglutarate -- rn:R01582 <-- Phenylpyruvate --> rn:R01378

rn:R07996 { Liquiritigenin } <-- 2-Oxoglutarate -- rn:R01582 <-- Phenylpyruvate --> rn:R01378

rn:R07712 { Liquiritigenin } <-- 2-Oxoglutarate -- rn:R01582 <-- Phenylpyruvate --> rn:R10431

rn:R07996 { Liquiritigenin } <-- 2-Oxoglutarate -- rn:R01582 <-- Phenylpyruvate --> rn:R10431

rn:R07712 { Liquiritigenin } <-- 2-Oxoglutarate -- rn:R01582 <-- Phenylpyruvate --> rn:R10500

rn:R07996 { Liquiritigenin } <-- 2-Oxoglutarate -- rn:R01582 <-- Phenylpyruvate --> rn:R10500

rn:R07712 { Liquiritigenin } <-- 2-Oxoglutarate -- rn:R02077 <-- L-Glutamate --> rn:R00114

rn:R07996 { Liquiritigenin } <-- 2-Oxoglutarate -- rn:R02077 <-- L-Glutamate --> rn:R00114

rn:R07712 { Liquiritigenin } <-- 2-Oxoglutarate -- rn:R02077 <-- L-Glutamate --> rn:R00248

rn:R07996 { Liquiritigenin } <-- 2-Oxoglutarate -- rn:R02077 <-- L-Glutamate --> rn:R00248

rn:R07712 { Liquiritigenin } <-- 2-Oxoglutarate -- rn:R02077 <-- L-Glutamate --> rn:R00254

rn:R07996 { Liquiritigenin } <-- 2-Oxoglutarate -- rn:R02077 <-- L-Glutamate --> rn:R00254

rn:R07712 { Liquiritigenin } <-- 2-Oxoglutarate -- rn:R02077 <-- L-Glutamate --> rn:R00894

rn:R07996 { Liquiritigenin } <-- 2-Oxoglutarate -- rn:R02077 <-- L-Glutamate --> rn:R00894

rn:R07712 { Liquiritigenin } <-- 2-Oxoglutarate -- rn:R02077 <-- L-Glutamate --> rn:R02287

rn:R07996 { Liquiritigenin } <-- 2-Oxoglutarate -- rn:R02077 <-- L-Glutamate --> rn:R02287

rn:R07712 { Liquiritigenin } <-- 2-Oxoglutarate -- rn:R02077 <-- L-Glutamate --> rn:R03189

rn:R07996 { Liquiritigenin } <-- 2-Oxoglutarate -- rn:R02077 <-- L-Glutamate --> rn:R03189

rn:R07712 { Liquiritigenin } <-- 2-Oxoglutarate -- rn:R02077 <-- L-Glutamate --> rn:R03970

rn:R07996 { Liquiritigenin } <-- 2-Oxoglutarate -- rn:R02077 <-- L-Glutamate --> rn:R03970

rn:R07712 { Liquiritigenin } <-- 2-Oxoglutarate -- rn:R02077 <-- L-Glutamate --> rn:R03971

rn:R07996 { Liquiritigenin } <-- 2-Oxoglutarate -- rn:R02077 <-- L-Glutamate --> rn:R03971

rn:R07712 { Liquiritigenin } <-- 2-Oxoglutarate -- rn:R02077 <-- L-Glutamate --> rn:R04051

rn:R07996 { Liquiritigenin } <-- 2-Oxoglutarate -- rn:R02077 <-- L-Glutamate --> rn:R04051

rn:R07712 { Liquiritigenin } <-- 2-Oxoglutarate -- rn:R02077 <-- L-Glutamate --> rn:R04776

rn:R07996 { Liquiritigenin } <-- 2-Oxoglutarate -- rn:R02077 <-- L-Glutamate --> rn:R04776

rn:R07712 { Liquiritigenin } <-- 2-Oxoglutarate -- rn:R02077 <-- L-Glutamate --> rn:R07643

rn:R07996 { Liquiritigenin } <-- 2-Oxoglutarate -- rn:R02077 <-- L-Glutamate --> rn:R07643

rn:R03006 { Formononetin } <-- [Reduced NADPH---hemoprotein reductase] -- rn:R02253 <-- [Oxidized NADPH---hemoprotein reductase] --> rn:R08551

rn:R03615 { Flavonoid } <-- [Reduced NADPH---hemoprotein reductase] -- rn:R02253 <-- [Oxidized NADPH---hemoprotein reductase] --> rn:R08551

rn:R06560 { Formononetin } <-- [Reduced NADPH---hemoprotein reductase] -- rn:R02253 <-- [Oxidized NADPH---hemoprotein reductase] --> rn:R08551

rn:R07198 { Liquiritigenin } <-- [Reduced NADPH---hemoprotein reductase] -- rn:R02253 <-- [Oxidized NADPH---hemoprotein reductase] --> rn:R08551

rn:R07745 { Calycosin } <-- [Reduced NADPH---hemoprotein reductase] -- rn:R02253 <-- [Oxidized NADPH---hemoprotein reductase] --> rn:R08551

rn:R07777 { Liquiritigenin } <-- [Reduced NADPH---hemoprotein reductase] -- rn:R02253 <-- [Oxidized NADPH---hemoprotein reductase] --> rn:R08551

rn:R08002 { Liquiritigenin } <-- [Reduced NADPH---hemoprotein reductase] -- rn:R02253 <-- [Oxidized NADPH---hemoprotein reductase] --> rn:R08551

rn:R03006 { Formononetin } <-- [Reduced NADPH---hemoprotein reductase] -- rn:R02708 <-- (S)-4-Hydroxymandelonitrile --> rn:R02676

rn:R03615 { Flavonoid } <-- [Reduced NADPH---hemoprotein reductase] -- rn:R02708 <-- (S)-4-Hydroxymandelonitrile --> rn:R02676

rn:R06560 { Formononetin } <-- [Reduced NADPH---hemoprotein reductase] -- rn:R02708 <-- (S)-4-Hydroxymandelonitrile --> rn:R02676

rn:R07198 { Liquiritigenin } <-- [Reduced NADPH---hemoprotein reductase] -- rn:R02708 <-- (S)-4-Hydroxymandelonitrile --> rn:R02676

rn:R07745 { Calycosin } <-- [Reduced NADPH---hemoprotein reductase] -- rn:R02708 <-- (S)-4-Hydroxymandelonitrile --> rn:R02676

rn:R07777 { Liquiritigenin } <-- [Reduced NADPH---hemoprotein reductase] -- rn:R02708 <-- (S)-4-Hydroxymandelonitrile --> rn:R02676

rn:R08002 { Liquiritigenin } <-- [Reduced NADPH---hemoprotein reductase] -- rn:R02708 <-- (S)-4-Hydroxymandelonitrile --> rn:R02676

rn:R03006 { Formononetin } <-- [Reduced NADPH---hemoprotein reductase] -- rn:R02708 <-- (S)-4-Hydroxymandelonitrile --> rn:R04296

rn:R03615 { Flavonoid } <-- [Reduced NADPH---hemoprotein reductase] -- rn:R02708 <-- (S)-4-Hydroxymandelonitrile --> rn:R04296

rn:R06560 { Formononetin } <-- [Reduced NADPH---hemoprotein reductase] -- rn:R02708 <-- (S)-4-Hydroxymandelonitrile --> rn:R04296

rn:R07198 { Liquiritigenin } <-- [Reduced NADPH---hemoprotein reductase] -- rn:R02708 <-- (S)-4-Hydroxymandelonitrile --> rn:R04296

rn:R07745 { Calycosin } <-- [Reduced NADPH---hemoprotein reductase] -- rn:R02708 <-- (S)-4-Hydroxymandelonitrile --> rn:R04296

rn:R07777 { Liquiritigenin } <-- [Reduced NADPH---hemoprotein reductase] -- rn:R02708 <-- (S)-4-Hydroxymandelonitrile --> rn:R04296

rn:R08002 { Liquiritigenin } <-- [Reduced NADPH---hemoprotein reductase] -- rn:R02708 <-- (S)-4-Hydroxymandelonitrile --> rn:R04296

rn:R03006 { Formononetin } <-- [Reduced NADPH---hemoprotein reductase] -- rn:R02708 <-- [Oxidized NADPH---hemoprotein reductase] --> rn:R08551

rn:R03615 { Flavonoid } <-- [Reduced NADPH---hemoprotein reductase] -- rn:R02708 <-- [Oxidized NADPH---hemoprotein reductase] --> rn:R08551

rn:R06560 { Formononetin } <-- [Reduced NADPH---hemoprotein reductase] -- rn:R02708 <-- [Oxidized NADPH---hemoprotein reductase] --> rn:R08551

rn:R07198 { Liquiritigenin } <-- [Reduced NADPH---hemoprotein reductase] -- rn:R02708 <-- [Oxidized NADPH---hemoprotein reductase] --> rn:R08551

rn:R07745 { Calycosin } <-- [Reduced NADPH---hemoprotein reductase] -- rn:R02708 <-- [Oxidized NADPH---hemoprotein reductase] --> rn:R08551

rn:R07777 { Liquiritigenin } <-- [Reduced NADPH---hemoprotein reductase] -- rn:R02708 <-- [Oxidized NADPH---hemoprotein reductase] --> rn:R08551

rn:R08002 { Liquiritigenin } <-- [Reduced NADPH---hemoprotein reductase] -- rn:R02708 <-- [Oxidized NADPH---hemoprotein reductase] --> rn:R08551

rn:R07712 { Liquiritigenin } <-- 2-Oxoglutarate -- rn:R02772 <-- L-Glutamate --> rn:R00114

rn:R07996 { Liquiritigenin } <-- 2-Oxoglutarate -- rn:R02772 <-- L-Glutamate --> rn:R00114

rn:R07712 { Liquiritigenin } <-- 2-Oxoglutarate -- rn:R02772 <-- L-Glutamate --> rn:R00248

rn:R07996 { Liquiritigenin } <-- 2-Oxoglutarate -- rn:R02772 <-- L-Glutamate --> rn:R00248

rn:R07712 { Liquiritigenin } <-- 2-Oxoglutarate -- rn:R02772 <-- L-Glutamate --> rn:R00254

rn:R07996 { Liquiritigenin } <-- 2-Oxoglutarate -- rn:R02772 <-- L-Glutamate --> rn:R00254

rn:R07712 { Liquiritigenin } <-- 2-Oxoglutarate -- rn:R02772 <-- Acetyl-CoA --> rn:R00693

rn:R07996 { Liquiritigenin } <-- 2-Oxoglutarate -- rn:R02772 <-- Acetyl-CoA --> rn:R00693

rn:R07712 { Liquiritigenin } <-- 2-Oxoglutarate -- rn:R02772 <-- L-Glutamate --> rn:R00894

rn:R07996 { Liquiritigenin } <-- 2-Oxoglutarate -- rn:R02772 <-- L-Glutamate --> rn:R00894

rn:R07712 { Liquiritigenin } <-- 2-Oxoglutarate -- rn:R02772 <-- Acetyl-CoA --> rn:R02152

rn:R07996 { Liquiritigenin } <-- 2-Oxoglutarate -- rn:R02772 <-- Acetyl-CoA --> rn:R02152

rn:R07712 { Liquiritigenin } <-- 2-Oxoglutarate -- rn:R02772 <-- L-Glutamate --> rn:R02287

rn:R07996 { Liquiritigenin } <-- 2-Oxoglutarate -- rn:R02772 <-- L-Glutamate --> rn:R02287

rn:R07712 { Liquiritigenin } <-- 2-Oxoglutarate -- rn:R02772 <-- Acetyl-CoA --> rn:R02911

rn:R07996 { Liquiritigenin } <-- 2-Oxoglutarate -- rn:R02772 <-- Acetyl-CoA --> rn:R02911

rn:R07712 { Liquiritigenin } <-- 2-Oxoglutarate -- rn:R02772 <-- Acetyl-CoA --> rn:R02955

rn:R07996 { Liquiritigenin } <-- 2-Oxoglutarate -- rn:R02772 <-- Acetyl-CoA --> rn:R02955

rn:R07712 { Liquiritigenin } <-- 2-Oxoglutarate -- rn:R02772 <-- Acetyl-CoA --> rn:R03153

rn:R07996 { Liquiritigenin } <-- 2-Oxoglutarate -- rn:R02772 <-- Acetyl-CoA --> rn:R03153

rn:R07712 { Liquiritigenin } <-- 2-Oxoglutarate -- rn:R02772 <-- L-Glutamate --> rn:R03189

rn:R07996 { Liquiritigenin } <-- 2-Oxoglutarate -- rn:R02772 <-- L-Glutamate --> rn:R03189

rn:R07712 { Liquiritigenin } <-- 2-Oxoglutarate -- rn:R02772 <-- Acetyl-CoA --> rn:R03903

rn:R07996 { Liquiritigenin } <-- 2-Oxoglutarate -- rn:R02772 <-- Acetyl-CoA --> rn:R03903

rn:R07712 { Liquiritigenin } <-- 2-Oxoglutarate -- rn:R02772 <-- L-Glutamate --> rn:R03970

rn:R07996 { Liquiritigenin } <-- 2-Oxoglutarate -- rn:R02772 <-- L-Glutamate --> rn:R03970

rn:R07712 { Liquiritigenin } <-- 2-Oxoglutarate -- rn:R02772 <-- L-Glutamate --> rn:R03971

rn:R07996 { Liquiritigenin } <-- 2-Oxoglutarate -- rn:R02772 <-- L-Glutamate --> rn:R03971

rn:R07712 { Liquiritigenin } <-- 2-Oxoglutarate -- rn:R02772 <-- L-Glutamate --> rn:R04051

rn:R07996 { Liquiritigenin } <-- 2-Oxoglutarate -- rn:R02772 <-- L-Glutamate --> rn:R04051

rn:R07712 { Liquiritigenin } <-- 2-Oxoglutarate -- rn:R02772 <-- L-Glutamate --> rn:R04776

rn:R07996 { Liquiritigenin } <-- 2-Oxoglutarate -- rn:R02772 <-- L-Glutamate --> rn:R04776

rn:R07712 { Liquiritigenin } <-- 2-Oxoglutarate -- rn:R02772 <-- Acetyl-CoA --> rn:R04950

rn:R07996 { Liquiritigenin } <-- 2-Oxoglutarate -- rn:R02772 <-- Acetyl-CoA --> rn:R04950

rn:R07712 { Liquiritigenin } <-- 2-Oxoglutarate -- rn:R02772 <-- Acetyl-CoA --> rn:R05509

rn:R07996 { Liquiritigenin } <-- 2-Oxoglutarate -- rn:R02772 <-- Acetyl-CoA --> rn:R05509

rn:R07712 { Liquiritigenin } <-- 2-Oxoglutarate -- rn:R02772 <-- Acetyl-CoA --> rn:R07253

rn:R07996 { Liquiritigenin } <-- 2-Oxoglutarate -- rn:R02772 <-- Acetyl-CoA --> rn:R07253

rn:R07712 { Liquiritigenin } <-- 2-Oxoglutarate -- rn:R02772 <-- L-Glutamate --> rn:R07643

rn:R07996 { Liquiritigenin } <-- 2-Oxoglutarate -- rn:R02772 <-- L-Glutamate --> rn:R07643

rn:R07712 { Liquiritigenin } <-- 2-Oxoglutarate -- rn:R02772 <-- Acetyl-CoA --> rn:R07937

rn:R07996 { Liquiritigenin } <-- 2-Oxoglutarate -- rn:R02772 <-- Acetyl-CoA --> rn:R07937

rn:R07712 { Liquiritigenin } <-- 2-Oxoglutarate -- rn:R02772 <-- Acetyl-CoA --> rn:R07953

rn:R07996 { Liquiritigenin } <-- 2-Oxoglutarate -- rn:R02772 <-- Acetyl-CoA --> rn:R07953

rn:R07712 { Liquiritigenin } <-- 2-Oxoglutarate -- rn:R02772 <-- Acetyl-CoA --> rn:R08036

rn:R07996 { Liquiritigenin } <-- 2-Oxoglutarate -- rn:R02772 <-- Acetyl-CoA --> rn:R08036

rn:R07712 { Liquiritigenin } <-- 2-Oxoglutarate -- rn:R02772 <-- Acetyl-CoA --> rn:R08870

rn:R07996 { Liquiritigenin } <-- 2-Oxoglutarate -- rn:R02772 <-- Acetyl-CoA --> rn:R08870

rn:R07712 { Liquiritigenin } <-- 2-Oxoglutarate -- rn:R02772 <-- Acetyl-CoA --> rn:R08871

rn:R07996 { Liquiritigenin } <-- 2-Oxoglutarate -- rn:R02772 <-- Acetyl-CoA --> rn:R08871

rn:R07712 { Liquiritigenin } <-- 2-Oxoglutarate -- rn:R02772 <-- Acetyl-CoA --> rn:R08938

rn:R07996 { Liquiritigenin } <-- 2-Oxoglutarate -- rn:R02772 <-- Acetyl-CoA --> rn:R08938

rn:R07712 { Liquiritigenin } <-- 2-Oxoglutarate -- rn:R02772 <-- Acetyl-CoA --> rn:R10500

rn:R07996 { Liquiritigenin } <-- 2-Oxoglutarate -- rn:R02772 <-- Acetyl-CoA --> rn:R10500

rn:R07712 { Liquiritigenin } <-- 2-Oxoglutarate -- rn:R02772 <-- Acetyl-CoA --> rn:R10600

rn:R07996 { Liquiritigenin } <-- 2-Oxoglutarate -- rn:R02772 <-- Acetyl-CoA --> rn:R10600

rn:R07712 { Liquiritigenin } <-- 2-Oxoglutarate -- rn:R02772 <-- Acetyl-CoA --> rn:R10745

rn:R07996 { Liquiritigenin } <-- 2-Oxoglutarate -- rn:R02772 <-- Acetyl-CoA --> rn:R10745

rn:R07712 { Liquiritigenin } <-- 2-Oxoglutarate -- rn:R02772 <-- Acetyl-CoA --> rn:R10746

rn:R07996 { Liquiritigenin } <-- 2-Oxoglutarate -- rn:R02772 <-- Acetyl-CoA --> rn:R10746

rn:R07712 { Liquiritigenin } <-- 2-Oxoglutarate -- rn:R02772 <-- Acetyl-CoA --> rn:R10893

rn:R07996 { Liquiritigenin } <-- 2-Oxoglutarate -- rn:R02772 <-- Acetyl-CoA --> rn:R10893

rn:R07712 { Liquiritigenin } <-- 2-Oxoglutarate -- rn:R02772 <-- Acetyl-CoA --> rn:R11124

rn:R07996 { Liquiritigenin } <-- 2-Oxoglutarate -- rn:R02772 <-- Acetyl-CoA --> rn:R11124

rn:R07712 { Liquiritigenin } <-- 2-Oxoglutarate -- rn:R02772 <-- Acetyl-CoA --> rn:R11125

rn:R07996 { Liquiritigenin } <-- 2-Oxoglutarate -- rn:R02772 <-- Acetyl-CoA --> rn:R11125

rn:R07712 { Liquiritigenin } <-- 2-Oxoglutarate -- rn:R02772 <-- Acetyl-CoA --> rn:R11479

rn:R07996 { Liquiritigenin } <-- 2-Oxoglutarate -- rn:R02772 <-- Acetyl-CoA --> rn:R11479

rn:R07712 { Liquiritigenin } <-- 2-Oxoglutarate -- rn:R02772 <-- Acetyl-CoA --> rn:R11708

rn:R07996 { Liquiritigenin } <-- 2-Oxoglutarate -- rn:R02772 <-- Acetyl-CoA --> rn:R11708

rn:R07712 { Liquiritigenin } <-- 2-Oxoglutarate -- rn:R02772 <-- Acetyl-CoA --> rn:R11902

rn:R07996 { Liquiritigenin } <-- 2-Oxoglutarate -- rn:R02772 <-- Acetyl-CoA --> rn:R11902

rn:R07712 { Liquiritigenin } <-- 2-Oxoglutarate -- rn:R02773 <-- L-Glutamate --> rn:R00114

rn:R07996 { Liquiritigenin } <-- 2-Oxoglutarate -- rn:R02773 <-- L-Glutamate --> rn:R00114

rn:R07712 { Liquiritigenin } <-- 2-Oxoglutarate -- rn:R02773 <-- L-Glutamate --> rn:R00248

rn:R07996 { Liquiritigenin } <-- 2-Oxoglutarate -- rn:R02773 <-- L-Glutamate --> rn:R00248

rn:R07712 { Liquiritigenin } <-- 2-Oxoglutarate -- rn:R02773 <-- L-Glutamate --> rn:R00254

rn:R07996 { Liquiritigenin } <-- 2-Oxoglutarate -- rn:R02773 <-- L-Glutamate --> rn:R00254

rn:R07712 { Liquiritigenin } <-- 2-Oxoglutarate -- rn:R02773 <-- L-Glutamate --> rn:R00894

rn:R07996 { Liquiritigenin } <-- 2-Oxoglutarate -- rn:R02773 <-- L-Glutamate --> rn:R00894

rn:R07712 { Liquiritigenin } <-- 2-Oxoglutarate -- rn:R02773 <-- L-Glutamate --> rn:R02287

rn:R07996 { Liquiritigenin } <-- 2-Oxoglutarate -- rn:R02773 <-- L-Glutamate --> rn:R02287

rn:R07712 { Liquiritigenin } <-- 2-Oxoglutarate -- rn:R02773 <-- L-Glutamate --> rn:R03189

rn:R07996 { Liquiritigenin } <-- 2-Oxoglutarate -- rn:R02773 <-- L-Glutamate --> rn:R03189

rn:R07712 { Liquiritigenin } <-- 2-Oxoglutarate -- rn:R02773 <-- L-Glutamate --> rn:R03970

rn:R07996 { Liquiritigenin } <-- 2-Oxoglutarate -- rn:R02773 <-- L-Glutamate --> rn:R03970

rn:R07712 { Liquiritigenin } <-- 2-Oxoglutarate -- rn:R02773 <-- L-Glutamate --> rn:R03971

rn:R07996 { Liquiritigenin } <-- 2-Oxoglutarate -- rn:R02773 <-- L-Glutamate --> rn:R03971

rn:R07712 { Liquiritigenin } <-- 2-Oxoglutarate -- rn:R02773 <-- L-Glutamate --> rn:R04051

rn:R07996 { Liquiritigenin } <-- 2-Oxoglutarate -- rn:R02773 <-- L-Glutamate --> rn:R04051

rn:R07712 { Liquiritigenin } <-- 2-Oxoglutarate -- rn:R02773 <-- L-Glutamate --> rn:R04776

rn:R07996 { Liquiritigenin } <-- 2-Oxoglutarate -- rn:R02773 <-- L-Glutamate --> rn:R04776

rn:R07712 { Liquiritigenin } <-- 2-Oxoglutarate -- rn:R02773 <-- L-Glutamate --> rn:R07643

rn:R07996 { Liquiritigenin } <-- 2-Oxoglutarate -- rn:R02773 <-- L-Glutamate --> rn:R07643

rn:R07712 { Liquiritigenin } <-- 2-Oxoglutarate -- rn:R03207 <-- L-Glutamate --> rn:R00114

rn:R07996 { Liquiritigenin } <-- 2-Oxoglutarate -- rn:R03207 <-- L-Glutamate --> rn:R00114

rn:R07712 { Liquiritigenin } <-- 2-Oxoglutarate -- rn:R03207 <-- L-Glutamate --> rn:R00248

rn:R07996 { Liquiritigenin } <-- 2-Oxoglutarate -- rn:R03207 <-- L-Glutamate --> rn:R00248

rn:R07712 { Liquiritigenin } <-- 2-Oxoglutarate -- rn:R03207 <-- L-Glutamate --> rn:R00254

rn:R07996 { Liquiritigenin } <-- 2-Oxoglutarate -- rn:R03207 <-- L-Glutamate --> rn:R00254

rn:R07712 { Liquiritigenin } <-- 2-Oxoglutarate -- rn:R03207 <-- L-Glutamate --> rn:R00894

rn:R07996 { Liquiritigenin } <-- 2-Oxoglutarate -- rn:R03207 <-- L-Glutamate --> rn:R00894

rn:R07712 { Liquiritigenin } <-- 2-Oxoglutarate -- rn:R03207 <-- L-Glutamate --> rn:R02287

rn:R07996 { Liquiritigenin } <-- 2-Oxoglutarate -- rn:R03207 <-- L-Glutamate --> rn:R02287

rn:R07712 { Liquiritigenin } <-- 2-Oxoglutarate -- rn:R03207 <-- L-Glutamate --> rn:R03189

rn:R07996 { Liquiritigenin } <-- 2-Oxoglutarate -- rn:R03207 <-- L-Glutamate --> rn:R03189

rn:R07712 { Liquiritigenin } <-- 2-Oxoglutarate -- rn:R03207 <-- L-Glutamate --> rn:R03970

rn:R07996 { Liquiritigenin } <-- 2-Oxoglutarate -- rn:R03207 <-- L-Glutamate --> rn:R03970

rn:R07712 { Liquiritigenin } <-- 2-Oxoglutarate -- rn:R03207 <-- L-Glutamate --> rn:R03971

rn:R07996 { Liquiritigenin } <-- 2-Oxoglutarate -- rn:R03207 <-- L-Glutamate --> rn:R03971

rn:R07712 { Liquiritigenin } <-- 2-Oxoglutarate -- rn:R03207 <-- L-Glutamate --> rn:R04051

rn:R07996 { Liquiritigenin } <-- 2-Oxoglutarate -- rn:R03207 <-- L-Glutamate --> rn:R04051

rn:R07712 { Liquiritigenin } <-- 2-Oxoglutarate -- rn:R03207 <-- L-Glutamate --> rn:R04776

rn:R07996 { Liquiritigenin } <-- 2-Oxoglutarate -- rn:R03207 <-- L-Glutamate --> rn:R04776

rn:R07712 { Liquiritigenin } <-- 2-Oxoglutarate -- rn:R03207 <-- L-Glutamate --> rn:R07643

rn:R07996 { Liquiritigenin } <-- 2-Oxoglutarate -- rn:R03207 <-- L-Glutamate --> rn:R07643

rn:R07712 { Liquiritigenin } <-- 2-Oxoglutarate -- rn:R03243 <-- L-Glutamate --> rn:R00114

rn:R07996 { Liquiritigenin } <-- 2-Oxoglutarate -- rn:R03243 <-- L-Glutamate --> rn:R00114

rn:R07712 { Liquiritigenin } <-- 2-Oxoglutarate -- rn:R03243 <-- L-Glutamate --> rn:R00248

rn:R07996 { Liquiritigenin } <-- 2-Oxoglutarate -- rn:R03243 <-- L-Glutamate --> rn:R00248

rn:R07712 { Liquiritigenin } <-- 2-Oxoglutarate -- rn:R03243 <-- L-Glutamate --> rn:R00254

rn:R07996 { Liquiritigenin } <-- 2-Oxoglutarate -- rn:R03243 <-- L-Glutamate --> rn:R00254

rn:R07712 { Liquiritigenin } <-- 2-Oxoglutarate -- rn:R03243 <-- L-Glutamate --> rn:R00894

rn:R07996 { Liquiritigenin } <-- 2-Oxoglutarate -- rn:R03243 <-- L-Glutamate --> rn:R00894

rn:R07712 { Liquiritigenin } <-- 2-Oxoglutarate -- rn:R03243 <-- L-Glutamate --> rn:R02287

rn:R07996 { Liquiritigenin } <-- 2-Oxoglutarate -- rn:R03243 <-- L-Glutamate --> rn:R02287

rn:R07712 { Liquiritigenin } <-- 2-Oxoglutarate -- rn:R03243 <-- L-Glutamate --> rn:R03189

rn:R07996 { Liquiritigenin } <-- 2-Oxoglutarate -- rn:R03243 <-- L-Glutamate --> rn:R03189

rn:R07712 { Liquiritigenin } <-- 2-Oxoglutarate -- rn:R03243 <-- L-Glutamate --> rn:R03970

rn:R07996 { Liquiritigenin } <-- 2-Oxoglutarate -- rn:R03243 <-- L-Glutamate --> rn:R03970

rn:R07712 { Liquiritigenin } <-- 2-Oxoglutarate -- rn:R03243 <-- L-Glutamate --> rn:R03971

rn:R07996 { Liquiritigenin } <-- 2-Oxoglutarate -- rn:R03243 <-- L-Glutamate --> rn:R03971

rn:R07712 { Liquiritigenin } <-- 2-Oxoglutarate -- rn:R03243 <-- L-Glutamate --> rn:R04051

rn:R07996 { Liquiritigenin } <-- 2-Oxoglutarate -- rn:R03243 <-- L-Glutamate --> rn:R04051

rn:R07712 { Liquiritigenin } <-- 2-Oxoglutarate -- rn:R03243 <-- L-Glutamate --> rn:R04776

rn:R07996 { Liquiritigenin } <-- 2-Oxoglutarate -- rn:R03243 <-- L-Glutamate --> rn:R04776

rn:R07712 { Liquiritigenin } <-- 2-Oxoglutarate -- rn:R03243 <-- L-Glutamate --> rn:R07643

rn:R07996 { Liquiritigenin } <-- 2-Oxoglutarate -- rn:R03243 <-- L-Glutamate --> rn:R07643

rn:R03548 { 7,8-Dihydroxycoumarin } <-- UDP-glucose -- rn:R03625 <-- Linamarin --> rn:R10040

rn:R07729 { Formononetin } <-- UDP-glucose -- rn:R03625 <-- Linamarin --> rn:R10040

rn:R07712 { Liquiritigenin } <-- 2-Oxoglutarate -- rn:R03952 <-- L-Glutamate --> rn:R00114

rn:R07996 { Liquiritigenin } <-- 2-Oxoglutarate -- rn:R03952 <-- L-Glutamate --> rn:R00114

rn:R07712 { Liquiritigenin } <-- 2-Oxoglutarate -- rn:R03952 <-- L-Glutamate --> rn:R00248

rn:R07996 { Liquiritigenin } <-- 2-Oxoglutarate -- rn:R03952 <-- L-Glutamate --> rn:R00248

rn:R07712 { Liquiritigenin } <-- 2-Oxoglutarate -- rn:R03952 <-- L-Glutamate --> rn:R00254

rn:R07996 { Liquiritigenin } <-- 2-Oxoglutarate -- rn:R03952 <-- L-Glutamate --> rn:R00254

rn:R07712 { Liquiritigenin } <-- 2-Oxoglutarate -- rn:R03952 <-- L-Glutamate --> rn:R00894

rn:R07996 { Liquiritigenin } <-- 2-Oxoglutarate -- rn:R03952 <-- L-Glutamate --> rn:R00894

rn:R07712 { Liquiritigenin } <-- 2-Oxoglutarate -- rn:R03952 <-- L-Glutamate --> rn:R02287

rn:R07996 { Liquiritigenin } <-- 2-Oxoglutarate -- rn:R03952 <-- L-Glutamate --> rn:R02287

rn:R07712 { Liquiritigenin } <-- 2-Oxoglutarate -- rn:R03952 <-- L-Glutamate --> rn:R03189

rn:R07996 { Liquiritigenin } <-- 2-Oxoglutarate -- rn:R03952 <-- L-Glutamate --> rn:R03189

rn:R07712 { Liquiritigenin } <-- 2-Oxoglutarate -- rn:R03952 <-- L-Glutamate --> rn:R03970

rn:R07996 { Liquiritigenin } <-- 2-Oxoglutarate -- rn:R03952 <-- L-Glutamate --> rn:R03970

rn:R07712 { Liquiritigenin } <-- 2-Oxoglutarate -- rn:R03952 <-- L-Glutamate --> rn:R03971

rn:R07996 { Liquiritigenin } <-- 2-Oxoglutarate -- rn:R03952 <-- L-Glutamate --> rn:R03971

rn:R07712 { Liquiritigenin } <-- 2-Oxoglutarate -- rn:R03952 <-- L-Glutamate --> rn:R04051

rn:R07996 { Liquiritigenin } <-- 2-Oxoglutarate -- rn:R03952 <-- L-Glutamate --> rn:R04051

rn:R07712 { Liquiritigenin } <-- 2-Oxoglutarate -- rn:R03952 <-- L-Glutamate --> rn:R04776

rn:R07996 { Liquiritigenin } <-- 2-Oxoglutarate -- rn:R03952 <-- L-Glutamate --> rn:R04776

rn:R07712 { Liquiritigenin } <-- 2-Oxoglutarate -- rn:R03952 <-- L-Glutamate --> rn:R07643

rn:R07996 { Liquiritigenin } <-- 2-Oxoglutarate -- rn:R03952 <-- L-Glutamate --> rn:R07643

rn:R03006 { Formononetin } <-- [Reduced NADPH---hemoprotein reductase] -- rn:R04121 <-- [Oxidized NADPH---hemoprotein reductase] --> rn:R08551

rn:R03615 { Flavonoid } <-- [Reduced NADPH---hemoprotein reductase] -- rn:R04121 <-- [Oxidized NADPH---hemoprotein reductase] --> rn:R08551

rn:R06560 { Formononetin } <-- [Reduced NADPH---hemoprotein reductase] -- rn:R04121 <-- [Oxidized NADPH---hemoprotein reductase] --> rn:R08551

rn:R07198 { Liquiritigenin } <-- [Reduced NADPH---hemoprotein reductase] -- rn:R04121 <-- [Oxidized NADPH---hemoprotein reductase] --> rn:R08551

rn:R07745 { Calycosin } <-- [Reduced NADPH---hemoprotein reductase] -- rn:R04121 <-- [Oxidized NADPH---hemoprotein reductase] --> rn:R08551

rn:R07777 { Liquiritigenin } <-- [Reduced NADPH---hemoprotein reductase] -- rn:R04121 <-- [Oxidized NADPH---hemoprotein reductase] --> rn:R08551

rn:R08002 { Liquiritigenin } <-- [Reduced NADPH---hemoprotein reductase] -- rn:R04121 <-- [Oxidized NADPH---hemoprotein reductase] --> rn:R08551

rn:R03006 { Formononetin } <-- [Reduced NADPH---hemoprotein reductase] -- rn:R04122 <-- [Oxidized NADPH---hemoprotein reductase] --> rn:R08551

rn:R03615 { Flavonoid } <-- [Reduced NADPH---hemoprotein reductase] -- rn:R04122 <-- [Oxidized NADPH---hemoprotein reductase] --> rn:R08551

rn:R06560 { Formononetin } <-- [Reduced NADPH---hemoprotein reductase] -- rn:R04122 <-- [Oxidized NADPH---hemoprotein reductase] --> rn:R08551

rn:R07198 { Liquiritigenin } <-- [Reduced NADPH---hemoprotein reductase] -- rn:R04122 <-- [Oxidized NADPH---hemoprotein reductase] --> rn:R08551

rn:R07745 { Calycosin } <-- [Reduced NADPH---hemoprotein reductase] -- rn:R04122 <-- [Oxidized NADPH---hemoprotein reductase] --> rn:R08551

rn:R07777 { Liquiritigenin } <-- [Reduced NADPH---hemoprotein reductase] -- rn:R04122 <-- [Oxidized NADPH---hemoprotein reductase] --> rn:R08551

rn:R08002 { Liquiritigenin } <-- [Reduced NADPH---hemoprotein reductase] -- rn:R04122 <-- [Oxidized NADPH---hemoprotein reductase] --> rn:R08551

rn:R07712 { Liquiritigenin } <-- 2-Oxoglutarate -- rn:R04188 <-- L-Glutamate --> rn:R00114

rn:R07996 { Liquiritigenin } <-- 2-Oxoglutarate -- rn:R04188 <-- L-Glutamate --> rn:R00114

rn:R07712 { Liquiritigenin } <-- 2-Oxoglutarate -- rn:R04188 <-- L-Glutamate --> rn:R00248

rn:R07996 { Liquiritigenin } <-- 2-Oxoglutarate -- rn:R04188 <-- L-Glutamate --> rn:R00248

rn:R07712 { Liquiritigenin } <-- 2-Oxoglutarate -- rn:R04188 <-- L-Glutamate --> rn:R00254

rn:R07996 { Liquiritigenin } <-- 2-Oxoglutarate -- rn:R04188 <-- L-Glutamate --> rn:R00254

rn:R07712 { Liquiritigenin } <-- 2-Oxoglutarate -- rn:R04188 <-- L-Glutamate --> rn:R00894

rn:R07996 { Liquiritigenin } <-- 2-Oxoglutarate -- rn:R04188 <-- L-Glutamate --> rn:R00894

rn:R07712 { Liquiritigenin } <-- 2-Oxoglutarate -- rn:R04188 <-- L-Glutamate --> rn:R02287

rn:R07996 { Liquiritigenin } <-- 2-Oxoglutarate -- rn:R04188 <-- L-Glutamate --> rn:R02287

rn:R07712 { Liquiritigenin } <-- 2-Oxoglutarate -- rn:R04188 <-- L-Glutamate --> rn:R03189

rn:R07996 { Liquiritigenin } <-- 2-Oxoglutarate -- rn:R04188 <-- L-Glutamate --> rn:R03189

rn:R07712 { Liquiritigenin } <-- 2-Oxoglutarate -- rn:R04188 <-- L-Glutamate --> rn:R03970

rn:R07996 { Liquiritigenin } <-- 2-Oxoglutarate -- rn:R04188 <-- L-Glutamate --> rn:R03970

rn:R07712 { Liquiritigenin } <-- 2-Oxoglutarate -- rn:R04188 <-- L-Glutamate --> rn:R03971

rn:R07996 { Liquiritigenin } <-- 2-Oxoglutarate -- rn:R04188 <-- L-Glutamate --> rn:R03971

rn:R07712 { Liquiritigenin } <-- 2-Oxoglutarate -- rn:R04188 <-- L-Glutamate --> rn:R04051

rn:R07996 { Liquiritigenin } <-- 2-Oxoglutarate -- rn:R04188 <-- L-Glutamate --> rn:R04051

rn:R07712 { Liquiritigenin } <-- 2-Oxoglutarate -- rn:R04188 <-- (S)-Methylmalonate semialdehyde --> rn:R04187

rn:R07996 { Liquiritigenin } <-- 2-Oxoglutarate -- rn:R04188 <-- (S)-Methylmalonate semialdehyde --> rn:R04187

rn:R07712 { Liquiritigenin } <-- 2-Oxoglutarate -- rn:R04188 <-- L-Glutamate --> rn:R04776

rn:R07996 { Liquiritigenin } <-- 2-Oxoglutarate -- rn:R04188 <-- L-Glutamate --> rn:R04776

rn:R07712 { Liquiritigenin } <-- 2-Oxoglutarate -- rn:R04188 <-- L-Glutamate --> rn:R07643

rn:R07996 { Liquiritigenin } <-- 2-Oxoglutarate -- rn:R04188 <-- L-Glutamate --> rn:R07643

rn:R07712 { Liquiritigenin } <-- 2-Oxoglutarate -- rn:R04234 <-- L-Glutamate --> rn:R00114

rn:R07996 { Liquiritigenin } <-- 2-Oxoglutarate -- rn:R04234 <-- L-Glutamate --> rn:R00114

rn:R07712 { Liquiritigenin } <-- 2-Oxoglutarate -- rn:R04234 <-- L-Glutamate --> rn:R00248

rn:R07996 { Liquiritigenin } <-- 2-Oxoglutarate -- rn:R04234 <-- L-Glutamate --> rn:R00248

rn:R07712 { Liquiritigenin } <-- 2-Oxoglutarate -- rn:R04234 <-- L-Glutamate --> rn:R00254

rn:R07996 { Liquiritigenin } <-- 2-Oxoglutarate -- rn:R04234 <-- L-Glutamate --> rn:R00254

rn:R07712 { Liquiritigenin } <-- 2-Oxoglutarate -- rn:R04234 <-- L-Glutamate --> rn:R00894

rn:R07996 { Liquiritigenin } <-- 2-Oxoglutarate -- rn:R04234 <-- L-Glutamate --> rn:R00894

rn:R07712 { Liquiritigenin } <-- 2-Oxoglutarate -- rn:R04234 <-- L-Glutamate --> rn:R02287

rn:R07996 { Liquiritigenin } <-- 2-Oxoglutarate -- rn:R04234 <-- L-Glutamate --> rn:R02287

rn:R07712 { Liquiritigenin } <-- 2-Oxoglutarate -- rn:R04234 <-- 4-Hydroxyphenylglyoxylate --> rn:R02672

rn:R07996 { Liquiritigenin } <-- 2-Oxoglutarate -- rn:R04234 <-- 4-Hydroxyphenylglyoxylate --> rn:R02672

rn:R07712 { Liquiritigenin } <-- 2-Oxoglutarate -- rn:R04234 <-- L-Glutamate --> rn:R03189

rn:R07996 { Liquiritigenin } <-- 2-Oxoglutarate -- rn:R04234 <-- L-Glutamate --> rn:R03189

rn:R07712 { Liquiritigenin } <-- 2-Oxoglutarate -- rn:R04234 <-- L-Glutamate --> rn:R03970

rn:R07996 { Liquiritigenin } <-- 2-Oxoglutarate -- rn:R04234 <-- L-Glutamate --> rn:R03970

rn:R07712 { Liquiritigenin } <-- 2-Oxoglutarate -- rn:R04234 <-- L-Glutamate --> rn:R03971

rn:R07996 { Liquiritigenin } <-- 2-Oxoglutarate -- rn:R04234 <-- L-Glutamate --> rn:R03971

rn:R07712 { Liquiritigenin } <-- 2-Oxoglutarate -- rn:R04234 <-- L-Glutamate --> rn:R04051

rn:R07996 { Liquiritigenin } <-- 2-Oxoglutarate -- rn:R04234 <-- L-Glutamate --> rn:R04051

rn:R07712 { Liquiritigenin } <-- 2-Oxoglutarate -- rn:R04234 <-- L-Glutamate --> rn:R04776

rn:R07996 { Liquiritigenin } <-- 2-Oxoglutarate -- rn:R04234 <-- L-Glutamate --> rn:R04776

rn:R07712 { Liquiritigenin } <-- 2-Oxoglutarate -- rn:R04234 <-- 4-Hydroxyphenylglyoxylate --> rn:R06626

rn:R07996 { Liquiritigenin } <-- 2-Oxoglutarate -- rn:R04234 <-- 4-Hydroxyphenylglyoxylate --> rn:R06626

rn:R07712 { Liquiritigenin } <-- 2-Oxoglutarate -- rn:R04234 <-- L-Glutamate --> rn:R07643

rn:R07996 { Liquiritigenin } <-- 2-Oxoglutarate -- rn:R04234 <-- L-Glutamate --> rn:R07643

rn:R07712 { Liquiritigenin } <-- 2-Oxoglutarate -- rn:R04269 <-- L-Glutamate --> rn:R00114

rn:R07996 { Liquiritigenin } <-- 2-Oxoglutarate -- rn:R04269 <-- L-Glutamate --> rn:R00114

rn:R07712 { Liquiritigenin } <-- 2-Oxoglutarate -- rn:R04269 <-- L-Glutamate --> rn:R00248

rn:R07996 { Liquiritigenin } <-- 2-Oxoglutarate -- rn:R04269 <-- L-Glutamate --> rn:R00248

rn:R07712 { Liquiritigenin } <-- 2-Oxoglutarate -- rn:R04269 <-- L-Glutamate --> rn:R00254

rn:R07996 { Liquiritigenin } <-- 2-Oxoglutarate -- rn:R04269 <-- L-Glutamate --> rn:R00254

rn:R07712 { Liquiritigenin } <-- 2-Oxoglutarate -- rn:R04269 <-- L-Glutamate --> rn:R00894

rn:R07996 { Liquiritigenin } <-- 2-Oxoglutarate -- rn:R04269 <-- L-Glutamate --> rn:R00894

rn:R07712 { Liquiritigenin } <-- 2-Oxoglutarate -- rn:R04269 <-- Methyloxaloacetate --> rn:R00995

rn:R07996 { Liquiritigenin } <-- 2-Oxoglutarate -- rn:R04269 <-- Methyloxaloacetate --> rn:R00995

rn:R07712 { Liquiritigenin } <-- 2-Oxoglutarate -- rn:R04269 <-- L-Glutamate --> rn:R02287

rn:R07996 { Liquiritigenin } <-- 2-Oxoglutarate -- rn:R04269 <-- L-Glutamate --> rn:R02287

rn:R07712 { Liquiritigenin } <-- 2-Oxoglutarate -- rn:R04269 <-- L-Glutamate --> rn:R03189

rn:R07996 { Liquiritigenin } <-- 2-Oxoglutarate -- rn:R04269 <-- L-Glutamate --> rn:R03189

rn:R07712 { Liquiritigenin } <-- 2-Oxoglutarate -- rn:R04269 <-- L-Glutamate --> rn:R03970

rn:R07996 { Liquiritigenin } <-- 2-Oxoglutarate -- rn:R04269 <-- L-Glutamate --> rn:R03970

rn:R07712 { Liquiritigenin } <-- 2-Oxoglutarate -- rn:R04269 <-- L-Glutamate --> rn:R03971

rn:R07996 { Liquiritigenin } <-- 2-Oxoglutarate -- rn:R04269 <-- L-Glutamate --> rn:R03971

rn:R07712 { Liquiritigenin } <-- 2-Oxoglutarate -- rn:R04269 <-- L-Glutamate --> rn:R04051

rn:R07996 { Liquiritigenin } <-- 2-Oxoglutarate -- rn:R04269 <-- L-Glutamate --> rn:R04051

rn:R07712 { Liquiritigenin } <-- 2-Oxoglutarate -- rn:R04269 <-- L-Glutamate --> rn:R04776

rn:R07996 { Liquiritigenin } <-- 2-Oxoglutarate -- rn:R04269 <-- L-Glutamate --> rn:R04776

rn:R07712 { Liquiritigenin } <-- 2-Oxoglutarate -- rn:R04269 <-- L-Glutamate --> rn:R07643

rn:R07996 { Liquiritigenin } <-- 2-Oxoglutarate -- rn:R04269 <-- L-Glutamate --> rn:R07643

rn:R03548 { 7,8-Dihydroxycoumarin } <-- UDP-glucose -- rn:R04296 <-- Dhurrin --> rn:R10035

rn:R07729 { Formononetin } <-- UDP-glucose -- rn:R04296 <-- Dhurrin --> rn:R10035

rn:R07712 { Liquiritigenin } <-- 2-Oxoglutarate -- rn:R04438 <-- L-Glutamate --> rn:R00114

rn:R07996 { Liquiritigenin } <-- 2-Oxoglutarate -- rn:R04438 <-- L-Glutamate --> rn:R00114

rn:R07712 { Liquiritigenin } <-- 2-Oxoglutarate -- rn:R04438 <-- L-Glutamate --> rn:R00248

rn:R07996 { Liquiritigenin } <-- 2-Oxoglutarate -- rn:R04438 <-- L-Glutamate --> rn:R00248

rn:R07712 { Liquiritigenin } <-- 2-Oxoglutarate -- rn:R04438 <-- L-Glutamate --> rn:R00254

rn:R07996 { Liquiritigenin } <-- 2-Oxoglutarate -- rn:R04438 <-- L-Glutamate --> rn:R00254

rn:R07712 { Liquiritigenin } <-- 2-Oxoglutarate -- rn:R04438 <-- L-Glutamate --> rn:R00894

rn:R07996 { Liquiritigenin } <-- 2-Oxoglutarate -- rn:R04438 <-- L-Glutamate --> rn:R00894

rn:R07712 { Liquiritigenin } <-- 2-Oxoglutarate -- rn:R04438 <-- L-Glutamate --> rn:R02287

rn:R07996 { Liquiritigenin } <-- 2-Oxoglutarate -- rn:R04438 <-- L-Glutamate --> rn:R02287

rn:R07712 { Liquiritigenin } <-- 2-Oxoglutarate -- rn:R04438 <-- L-Glutamate --> rn:R03189

rn:R07996 { Liquiritigenin } <-- 2-Oxoglutarate -- rn:R04438 <-- L-Glutamate --> rn:R03189

rn:R07712 { Liquiritigenin } <-- 2-Oxoglutarate -- rn:R04438 <-- L-Glutamate --> rn:R03970

rn:R07996 { Liquiritigenin } <-- 2-Oxoglutarate -- rn:R04438 <-- L-Glutamate --> rn:R03970

rn:R07712 { Liquiritigenin } <-- 2-Oxoglutarate -- rn:R04438 <-- L-Glutamate --> rn:R03971

rn:R07996 { Liquiritigenin } <-- 2-Oxoglutarate -- rn:R04438 <-- L-Glutamate --> rn:R03971

rn:R07712 { Liquiritigenin } <-- 2-Oxoglutarate -- rn:R04438 <-- L-Glutamate --> rn:R04051

rn:R07996 { Liquiritigenin } <-- 2-Oxoglutarate -- rn:R04438 <-- L-Glutamate --> rn:R04051

rn:R07712 { Liquiritigenin } <-- 2-Oxoglutarate -- rn:R04438 <-- L-Glutamate --> rn:R04776

rn:R07996 { Liquiritigenin } <-- 2-Oxoglutarate -- rn:R04438 <-- L-Glutamate --> rn:R04776

rn:R07712 { Liquiritigenin } <-- 2-Oxoglutarate -- rn:R04438 <-- L-Glutamate --> rn:R07643

rn:R07996 { Liquiritigenin } <-- 2-Oxoglutarate -- rn:R04438 <-- L-Glutamate --> rn:R07643

rn:R03006 { Formononetin } <-- [Reduced NADPH---hemoprotein reductase] -- rn:R04460 <-- N,N-Dihydroxy-L-tyrosine --> rn:R07190

rn:R03615 { Flavonoid } <-- [Reduced NADPH---hemoprotein reductase] -- rn:R04460 <-- N,N-Dihydroxy-L-tyrosine --> rn:R07190

rn:R06560 { Formononetin } <-- [Reduced NADPH---hemoprotein reductase] -- rn:R04460 <-- N,N-Dihydroxy-L-tyrosine --> rn:R07190

rn:R07198 { Liquiritigenin } <-- [Reduced NADPH---hemoprotein reductase] -- rn:R04460 <-- N,N-Dihydroxy-L-tyrosine --> rn:R07190

rn:R07745 { Calycosin } <-- [Reduced NADPH---hemoprotein reductase] -- rn:R04460 <-- N,N-Dihydroxy-L-tyrosine --> rn:R07190

rn:R07777 { Liquiritigenin } <-- [Reduced NADPH---hemoprotein reductase] -- rn:R04460 <-- N,N-Dihydroxy-L-tyrosine --> rn:R07190

rn:R08002 { Liquiritigenin } <-- [Reduced NADPH---hemoprotein reductase] -- rn:R04460 <-- N,N-Dihydroxy-L-tyrosine --> rn:R07190

rn:R03006 { Formononetin } <-- [Reduced NADPH---hemoprotein reductase] -- rn:R04460 <-- [Oxidized NADPH---hemoprotein reductase] --> rn:R08551

rn:R03615 { Flavonoid } <-- [Reduced NADPH---hemoprotein reductase] -- rn:R04460 <-- [Oxidized NADPH---hemoprotein reductase] --> rn:R08551

rn:R06560 { Formononetin } <-- [Reduced NADPH---hemoprotein reductase] -- rn:R04460 <-- [Oxidized NADPH---hemoprotein reductase] --> rn:R08551

rn:R07198 { Liquiritigenin } <-- [Reduced NADPH---hemoprotein reductase] -- rn:R04460 <-- [Oxidized NADPH---hemoprotein reductase] --> rn:R08551

rn:R07745 { Calycosin } <-- [Reduced NADPH---hemoprotein reductase] -- rn:R04460 <-- [Oxidized NADPH---hemoprotein reductase] --> rn:R08551

rn:R07777 { Liquiritigenin } <-- [Reduced NADPH---hemoprotein reductase] -- rn:R04460 <-- [Oxidized NADPH---hemoprotein reductase] --> rn:R08551

rn:R08002 { Liquiritigenin } <-- [Reduced NADPH---hemoprotein reductase] -- rn:R04460 <-- [Oxidized NADPH---hemoprotein reductase] --> rn:R08551

rn:R07712 { Liquiritigenin } <-- 2-Oxoglutarate -- rn:R05085 <-- L-Glutamate --> rn:R00114

rn:R07996 { Liquiritigenin } <-- 2-Oxoglutarate -- rn:R05085 <-- L-Glutamate --> rn:R00114

rn:R07712 { Liquiritigenin } <-- 2-Oxoglutarate -- rn:R05085 <-- L-Glutamate --> rn:R00248

rn:R07996 { Liquiritigenin } <-- 2-Oxoglutarate -- rn:R05085 <-- L-Glutamate --> rn:R00248

rn:R07712 { Liquiritigenin } <-- 2-Oxoglutarate -- rn:R05085 <-- L-Glutamate --> rn:R00254

rn:R07996 { Liquiritigenin } <-- 2-Oxoglutarate -- rn:R05085 <-- L-Glutamate --> rn:R00254

rn:R07712 { Liquiritigenin } <-- 2-Oxoglutarate -- rn:R05085 <-- L-Glutamate --> rn:R00894

rn:R07996 { Liquiritigenin } <-- 2-Oxoglutarate -- rn:R05085 <-- L-Glutamate --> rn:R00894

rn:R07712 { Liquiritigenin } <-- 2-Oxoglutarate -- rn:R05085 <-- L-Glutamate --> rn:R02287

rn:R07996 { Liquiritigenin } <-- 2-Oxoglutarate -- rn:R05085 <-- L-Glutamate --> rn:R02287

rn:R07712 { Liquiritigenin } <-- 2-Oxoglutarate -- rn:R05085 <-- L-Glutamate --> rn:R03189

rn:R07996 { Liquiritigenin } <-- 2-Oxoglutarate -- rn:R05085 <-- L-Glutamate --> rn:R03189

rn:R07712 { Liquiritigenin } <-- 2-Oxoglutarate -- rn:R05085 <-- L-Glutamate --> rn:R03970

rn:R07996 { Liquiritigenin } <-- 2-Oxoglutarate -- rn:R05085 <-- L-Glutamate --> rn:R03970

rn:R07712 { Liquiritigenin } <-- 2-Oxoglutarate -- rn:R05085 <-- L-Glutamate --> rn:R03971

rn:R07996 { Liquiritigenin } <-- 2-Oxoglutarate -- rn:R05085 <-- L-Glutamate --> rn:R03971

rn:R07712 { Liquiritigenin } <-- 2-Oxoglutarate -- rn:R05085 <-- L-Glutamate --> rn:R04051

rn:R07996 { Liquiritigenin } <-- 2-Oxoglutarate -- rn:R05085 <-- L-Glutamate --> rn:R04051

rn:R07712 { Liquiritigenin } <-- 2-Oxoglutarate -- rn:R05085 <-- L-Glutamate --> rn:R04776

rn:R07996 { Liquiritigenin } <-- 2-Oxoglutarate -- rn:R05085 <-- L-Glutamate --> rn:R04776

rn:R07712 { Liquiritigenin } <-- 2-Oxoglutarate -- rn:R05085 <-- L-Glutamate --> rn:R07643

rn:R07996 { Liquiritigenin } <-- 2-Oxoglutarate -- rn:R05085 <-- L-Glutamate --> rn:R07643

rn:R03006 { Formononetin } <-- [Reduced NADPH---hemoprotein reductase] -- rn:R05259 <-- Sulfur --> rn:R00864

rn:R03615 { Flavonoid } <-- [Reduced NADPH---hemoprotein reductase] -- rn:R05259 <-- Sulfur --> rn:R00864

rn:R06560 { Formononetin } <-- [Reduced NADPH---hemoprotein reductase] -- rn:R05259 <-- Sulfur --> rn:R00864

rn:R07198 { Liquiritigenin } <-- [Reduced NADPH---hemoprotein reductase] -- rn:R05259 <-- Sulfur --> rn:R00864

rn:R07745 { Calycosin } <-- [Reduced NADPH---hemoprotein reductase] -- rn:R05259 <-- Sulfur --> rn:R00864

rn:R07777 { Liquiritigenin } <-- [Reduced NADPH---hemoprotein reductase] -- rn:R05259 <-- Sulfur --> rn:R00864

rn:R08002 { Liquiritigenin } <-- [Reduced NADPH---hemoprotein reductase] -- rn:R05259 <-- Sulfur --> rn:R00864

rn:R03006 { Formononetin } <-- [Reduced NADPH---hemoprotein reductase] -- rn:R05259 <-- Sulfur --> rn:R03533

rn:R03615 { Flavonoid } <-- [Reduced NADPH---hemoprotein reductase] -- rn:R05259 <-- Sulfur --> rn:R03533

rn:R06560 { Formononetin } <-- [Reduced NADPH---hemoprotein reductase] -- rn:R05259 <-- Sulfur --> rn:R03533

rn:R07198 { Liquiritigenin } <-- [Reduced NADPH---hemoprotein reductase] -- rn:R05259 <-- Sulfur --> rn:R03533

rn:R07745 { Calycosin } <-- [Reduced NADPH---hemoprotein reductase] -- rn:R05259 <-- Sulfur --> rn:R03533

rn:R07777 { Liquiritigenin } <-- [Reduced NADPH---hemoprotein reductase] -- rn:R05259 <-- Sulfur --> rn:R03533

rn:R08002 { Liquiritigenin } <-- [Reduced NADPH---hemoprotein reductase] -- rn:R05259 <-- Sulfur --> rn:R03533

rn:R03006 { Formononetin } <-- [Reduced NADPH---hemoprotein reductase] -- rn:R05259 <-- Sulfur --> rn:R07365

rn:R03615 { Flavonoid } <-- [Reduced NADPH---hemoprotein reductase] -- rn:R05259 <-- Sulfur --> rn:R07365

rn:R06560 { Formononetin } <-- [Reduced NADPH---hemoprotein reductase] -- rn:R05259 <-- Sulfur --> rn:R07365

rn:R07198 { Liquiritigenin } <-- [Reduced NADPH---hemoprotein reductase] -- rn:R05259 <-- Sulfur --> rn:R07365

rn:R07745 { Calycosin } <-- [Reduced NADPH---hemoprotein reductase] -- rn:R05259 <-- Sulfur --> rn:R07365

rn:R07777 { Liquiritigenin } <-- [Reduced NADPH---hemoprotein reductase] -- rn:R05259 <-- Sulfur --> rn:R07365

rn:R08002 { Liquiritigenin } <-- [Reduced NADPH---hemoprotein reductase] -- rn:R05259 <-- Sulfur --> rn:R07365

rn:R03006 { Formononetin } <-- [Reduced NADPH---hemoprotein reductase] -- rn:R05259 <-- [Oxidized NADPH---hemoprotein reductase] --> rn:R08551

rn:R03615 { Flavonoid } <-- [Reduced NADPH---hemoprotein reductase] -- rn:R05259 <-- [Oxidized NADPH---hemoprotein reductase] --> rn:R08551

rn:R06560 { Formononetin } <-- [Reduced NADPH---hemoprotein reductase] -- rn:R05259 <-- [Oxidized NADPH---hemoprotein reductase] --> rn:R08551

rn:R07198 { Liquiritigenin } <-- [Reduced NADPH---hemoprotein reductase] -- rn:R05259 <-- [Oxidized NADPH---hemoprotein reductase] --> rn:R08551

rn:R07745 { Calycosin } <-- [Reduced NADPH---hemoprotein reductase] -- rn:R05259 <-- [Oxidized NADPH---hemoprotein reductase] --> rn:R08551

rn:R07777 { Liquiritigenin } <-- [Reduced NADPH---hemoprotein reductase] -- rn:R05259 <-- [Oxidized NADPH---hemoprotein reductase] --> rn:R08551

rn:R08002 { Liquiritigenin } <-- [Reduced NADPH---hemoprotein reductase] -- rn:R05259 <-- [Oxidized NADPH---hemoprotein reductase] --> rn:R08551

rn:R03006 { Formononetin } <-- [Reduced NADPH---hemoprotein reductase] -- rn:R05487 <-- 2-Hydroxyphenylacetate --> rn:R05001

rn:R03615 { Flavonoid } <-- [Reduced NADPH---hemoprotein reductase] -- rn:R05487 <-- 2-Hydroxyphenylacetate --> rn:R05001

rn:R06560 { Formononetin } <-- [Reduced NADPH---hemoprotein reductase] -- rn:R05487 <-- 2-Hydroxyphenylacetate --> rn:R05001

rn:R07198 { Liquiritigenin } <-- [Reduced NADPH---hemoprotein reductase] -- rn:R05487 <-- 2-Hydroxyphenylacetate --> rn:R05001

rn:R07745 { Calycosin } <-- [Reduced NADPH---hemoprotein reductase] -- rn:R05487 <-- 2-Hydroxyphenylacetate --> rn:R05001

rn:R07777 { Liquiritigenin } <-- [Reduced NADPH---hemoprotein reductase] -- rn:R05487 <-- 2-Hydroxyphenylacetate --> rn:R05001

rn:R08002 { Liquiritigenin } <-- [Reduced NADPH---hemoprotein reductase] -- rn:R05487 <-- 2-Hydroxyphenylacetate --> rn:R05001

rn:R03006 { Formononetin } <-- [Reduced NADPH---hemoprotein reductase] -- rn:R05487 <-- 2-Hydroxyphenylacetate --> rn:R05450

rn:R03615 { Flavonoid } <-- [Reduced NADPH---hemoprotein reductase] -- rn:R05487 <-- 2-Hydroxyphenylacetate --> rn:R05450

rn:R06560 { Formononetin } <-- [Reduced NADPH---hemoprotein reductase] -- rn:R05487 <-- 2-Hydroxyphenylacetate --> rn:R05450

rn:R07198 { Liquiritigenin } <-- [Reduced NADPH---hemoprotein reductase] -- rn:R05487 <-- 2-Hydroxyphenylacetate --> rn:R05450

rn:R07745 { Calycosin } <-- [Reduced NADPH---hemoprotein reductase] -- rn:R05487 <-- 2-Hydroxyphenylacetate --> rn:R05450

rn:R07777 { Liquiritigenin } <-- [Reduced NADPH---hemoprotein reductase] -- rn:R05487 <-- 2-Hydroxyphenylacetate --> rn:R05450

rn:R08002 { Liquiritigenin } <-- [Reduced NADPH---hemoprotein reductase] -- rn:R05487 <-- 2-Hydroxyphenylacetate --> rn:R05450

rn:R03006 { Formononetin } <-- [Reduced NADPH---hemoprotein reductase] -- rn:R05487 <-- [Oxidized NADPH---hemoprotein reductase] --> rn:R08551

rn:R03615 { Flavonoid } <-- [Reduced NADPH---hemoprotein reductase] -- rn:R05487 <-- [Oxidized NADPH---hemoprotein reductase] --> rn:R08551

rn:R06560 { Formononetin } <-- [Reduced NADPH---hemoprotein reductase] -- rn:R05487 <-- [Oxidized NADPH---hemoprotein reductase] --> rn:R08551

rn:R07198 { Liquiritigenin } <-- [Reduced NADPH---hemoprotein reductase] -- rn:R05487 <-- [Oxidized NADPH---hemoprotein reductase] --> rn:R08551

rn:R07745 { Calycosin } <-- [Reduced NADPH---hemoprotein reductase] -- rn:R05487 <-- [Oxidized NADPH---hemoprotein reductase] --> rn:R08551

rn:R07777 { Liquiritigenin } <-- [Reduced NADPH---hemoprotein reductase] -- rn:R05487 <-- [Oxidized NADPH---hemoprotein reductase] --> rn:R08551

rn:R08002 { Liquiritigenin } <-- [Reduced NADPH---hemoprotein reductase] -- rn:R05487 <-- [Oxidized NADPH---hemoprotein reductase] --> rn:R08551

rn:R03006 { Formononetin } <-- [Reduced NADPH---hemoprotein reductase] -- rn:R05728 <-- (S)-4-Hydroxymandelonitrile --> rn:R02676

rn:R03615 { Flavonoid } <-- [Reduced NADPH---hemoprotein reductase] -- rn:R05728 <-- (S)-4-Hydroxymandelonitrile --> rn:R02676

rn:R06560 { Formononetin } <-- [Reduced NADPH---hemoprotein reductase] -- rn:R05728 <-- (S)-4-Hydroxymandelonitrile --> rn:R02676

rn:R07198 { Liquiritigenin } <-- [Reduced NADPH---hemoprotein reductase] -- rn:R05728 <-- (S)-4-Hydroxymandelonitrile --> rn:R02676

rn:R07745 { Calycosin } <-- [Reduced NADPH---hemoprotein reductase] -- rn:R05728 <-- (S)-4-Hydroxymandelonitrile --> rn:R02676

rn:R07777 { Liquiritigenin } <-- [Reduced NADPH---hemoprotein reductase] -- rn:R05728 <-- (S)-4-Hydroxymandelonitrile --> rn:R02676

rn:R08002 { Liquiritigenin } <-- [Reduced NADPH---hemoprotein reductase] -- rn:R05728 <-- (S)-4-Hydroxymandelonitrile --> rn:R02676

rn:R03006 { Formononetin } <-- [Reduced NADPH---hemoprotein reductase] -- rn:R05728 <-- (S)-4-Hydroxymandelonitrile --> rn:R04296

rn:R03615 { Flavonoid } <-- [Reduced NADPH---hemoprotein reductase] -- rn:R05728 <-- (S)-4-Hydroxymandelonitrile --> rn:R04296

rn:R06560 { Formononetin } <-- [Reduced NADPH---hemoprotein reductase] -- rn:R05728 <-- (S)-4-Hydroxymandelonitrile --> rn:R04296

rn:R07198 { Liquiritigenin } <-- [Reduced NADPH---hemoprotein reductase] -- rn:R05728 <-- (S)-4-Hydroxymandelonitrile --> rn:R04296

rn:R07745 { Calycosin } <-- [Reduced NADPH---hemoprotein reductase] -- rn:R05728 <-- (S)-4-Hydroxymandelonitrile --> rn:R04296

rn:R07777 { Liquiritigenin } <-- [Reduced NADPH---hemoprotein reductase] -- rn:R05728 <-- (S)-4-Hydroxymandelonitrile --> rn:R04296

rn:R08002 { Liquiritigenin } <-- [Reduced NADPH---hemoprotein reductase] -- rn:R05728 <-- (S)-4-Hydroxymandelonitrile --> rn:R04296

rn:R03006 { Formononetin } <-- [Reduced NADPH---hemoprotein reductase] -- rn:R05728 <-- [Oxidized NADPH---hemoprotein reductase] --> rn:R08551

rn:R03615 { Flavonoid } <-- [Reduced NADPH---hemoprotein reductase] -- rn:R05728 <-- [Oxidized NADPH---hemoprotein reductase] --> rn:R08551

rn:R06560 { Formononetin } <-- [Reduced NADPH---hemoprotein reductase] -- rn:R05728 <-- [Oxidized NADPH---hemoprotein reductase] --> rn:R08551

rn:R07198 { Liquiritigenin } <-- [Reduced NADPH---hemoprotein reductase] -- rn:R05728 <-- [Oxidized NADPH---hemoprotein reductase] --> rn:R08551

rn:R07745 { Calycosin } <-- [Reduced NADPH---hemoprotein reductase] -- rn:R05728 <-- [Oxidized NADPH---hemoprotein reductase] --> rn:R08551

rn:R07777 { Liquiritigenin } <-- [Reduced NADPH---hemoprotein reductase] -- rn:R05728 <-- [Oxidized NADPH---hemoprotein reductase] --> rn:R08551

rn:R08002 { Liquiritigenin } <-- [Reduced NADPH---hemoprotein reductase] -- rn:R05728 <-- [Oxidized NADPH---hemoprotein reductase] --> rn:R08551

rn:R07712 { Liquiritigenin } <-- 2-Oxoglutarate -- rn:R05839 <-- Pyridoxal --> rn:R00174

rn:R07996 { Liquiritigenin } <-- 2-Oxoglutarate -- rn:R05839 <-- Pyridoxal --> rn:R00174

rn:R07712 { Liquiritigenin } <-- 2-Oxoglutarate -- rn:R05839 <-- Pyridoxal --> rn:R01707

rn:R07996 { Liquiritigenin } <-- 2-Oxoglutarate -- rn:R05839 <-- Pyridoxal --> rn:R01707

rn:R07712 { Liquiritigenin } <-- 2-Oxoglutarate -- rn:R05839 <-- Pyridoxal --> rn:R01709

rn:R07996 { Liquiritigenin } <-- 2-Oxoglutarate -- rn:R05839 <-- Pyridoxal --> rn:R01709

rn:R06562 { 2'-Hydroxyformononetin } <-- 2'-Hydroxyformononetin -- rn:R06562 <-- (-)-Vestitone --> rn:R07737

rn:R07735 { 2'-Hydroxyformononetin } <-- 2'-Hydroxyformononetin -- rn:R06562 <-- (-)-Vestitone --> rn:R07737

rn:R10453 { 3-Amino-4,7-dihydroxycoumarin } <-- 3-Dimethylallyl-4-hydroxybenzoate -- rn:R06776 <-- Chlorobiocic acid --> rn:R06773

rn:R03006 { Formononetin } <-- [Reduced NADPH---hemoprotein reductase] -- rn:R07041 <-- [Oxidized NADPH---hemoprotein reductase] --> rn:R08551

rn:R03615 { Flavonoid } <-- [Reduced NADPH---hemoprotein reductase] -- rn:R07041 <-- [Oxidized NADPH---hemoprotein reductase] --> rn:R08551

rn:R06560 { Formononetin } <-- [Reduced NADPH---hemoprotein reductase] -- rn:R07041 <-- [Oxidized NADPH---hemoprotein reductase] --> rn:R08551

rn:R07198 { Liquiritigenin } <-- [Reduced NADPH---hemoprotein reductase] -- rn:R07041 <-- [Oxidized NADPH---hemoprotein reductase] --> rn:R08551

rn:R07745 { Calycosin } <-- [Reduced NADPH---hemoprotein reductase] -- rn:R07041 <-- [Oxidized NADPH---hemoprotein reductase] --> rn:R08551

rn:R07777 { Liquiritigenin } <-- [Reduced NADPH---hemoprotein reductase] -- rn:R07041 <-- [Oxidized NADPH---hemoprotein reductase] --> rn:R08551

rn:R08002 { Liquiritigenin } <-- [Reduced NADPH---hemoprotein reductase] -- rn:R07041 <-- [Oxidized NADPH---hemoprotein reductase] --> rn:R08551

rn:R03006 { Formononetin } <-- [Reduced NADPH---hemoprotein reductase] -- rn:R07046 <-- [Oxidized NADPH---hemoprotein reductase] --> rn:R08551

rn:R03615 { Flavonoid } <-- [Reduced NADPH---hemoprotein reductase] -- rn:R07046 <-- [Oxidized NADPH---hemoprotein reductase] --> rn:R08551

rn:R06560 { Formononetin } <-- [Reduced NADPH---hemoprotein reductase] -- rn:R07046 <-- [Oxidized NADPH---hemoprotein reductase] --> rn:R08551

rn:R07198 { Liquiritigenin } <-- [Reduced NADPH---hemoprotein reductase] -- rn:R07046 <-- [Oxidized NADPH---hemoprotein reductase] --> rn:R08551

rn:R07745 { Calycosin } <-- [Reduced NADPH---hemoprotein reductase] -- rn:R07046 <-- [Oxidized NADPH---hemoprotein reductase] --> rn:R08551

rn:R07777 { Liquiritigenin } <-- [Reduced NADPH---hemoprotein reductase] -- rn:R07046 <-- [Oxidized NADPH---hemoprotein reductase] --> rn:R08551

rn:R08002 { Liquiritigenin } <-- [Reduced NADPH---hemoprotein reductase] -- rn:R07046 <-- [Oxidized NADPH---hemoprotein reductase] --> rn:R08551

rn:R03006 { Formononetin } <-- [Reduced NADPH---hemoprotein reductase] -- rn:R07203 <-- [Oxidized NADPH---hemoprotein reductase] --> rn:R08551

rn:R03615 { Flavonoid } <-- [Reduced NADPH---hemoprotein reductase] -- rn:R07203 <-- [Oxidized NADPH---hemoprotein reductase] --> rn:R08551

rn:R06560 { Formononetin } <-- [Reduced NADPH---hemoprotein reductase] -- rn:R07203 <-- [Oxidized NADPH---hemoprotein reductase] --> rn:R08551

rn:R07198 { Liquiritigenin } <-- [Reduced NADPH---hemoprotein reductase] -- rn:R07203 <-- [Oxidized NADPH---hemoprotein reductase] --> rn:R08551

rn:R07745 { Calycosin } <-- [Reduced NADPH---hemoprotein reductase] -- rn:R07203 <-- [Oxidized NADPH---hemoprotein reductase] --> rn:R08551

rn:R07777 { Liquiritigenin } <-- [Reduced NADPH---hemoprotein reductase] -- rn:R07203 <-- [Oxidized NADPH---hemoprotein reductase] --> rn:R08551

rn:R08002 { Liquiritigenin } <-- [Reduced NADPH---hemoprotein reductase] -- rn:R07203 <-- [Oxidized NADPH---hemoprotein reductase] --> rn:R08551

rn:R03006 { Formononetin } <-- [Reduced NADPH---hemoprotein reductase] -- rn:R07205 <-- [Oxidized NADPH---hemoprotein reductase] --> rn:R08551

rn:R03615 { Flavonoid } <-- [Reduced NADPH---hemoprotein reductase] -- rn:R07205 <-- [Oxidized NADPH---hemoprotein reductase] --> rn:R08551

rn:R06560 { Formononetin } <-- [Reduced NADPH---hemoprotein reductase] -- rn:R07205 <-- [Oxidized NADPH---hemoprotein reductase] --> rn:R08551

rn:R07198 { Liquiritigenin } <-- [Reduced NADPH---hemoprotein reductase] -- rn:R07205 <-- [Oxidized NADPH---hemoprotein reductase] --> rn:R08551

rn:R07745 { Calycosin } <-- [Reduced NADPH---hemoprotein reductase] -- rn:R07205 <-- [Oxidized NADPH---hemoprotein reductase] --> rn:R08551

rn:R07777 { Liquiritigenin } <-- [Reduced NADPH---hemoprotein reductase] -- rn:R07205 <-- [Oxidized NADPH---hemoprotein reductase] --> rn:R08551

rn:R08002 { Liquiritigenin } <-- [Reduced NADPH---hemoprotein reductase] -- rn:R07205 <-- [Oxidized NADPH---hemoprotein reductase] --> rn:R08551

rn:R03006 { Formononetin } <-- [Reduced NADPH---hemoprotein reductase] -- rn:R07206 <-- [Oxidized NADPH---hemoprotein reductase] --> rn:R08551

rn:R03615 { Flavonoid } <-- [Reduced NADPH---hemoprotein reductase] -- rn:R07206 <-- [Oxidized NADPH---hemoprotein reductase] --> rn:R08551

rn:R06560 { Formononetin } <-- [Reduced NADPH---hemoprotein reductase] -- rn:R07206 <-- [Oxidized NADPH---hemoprotein reductase] --> rn:R08551

rn:R07198 { Liquiritigenin } <-- [Reduced NADPH---hemoprotein reductase] -- rn:R07206 <-- [Oxidized NADPH---hemoprotein reductase] --> rn:R08551

rn:R07745 { Calycosin } <-- [Reduced NADPH---hemoprotein reductase] -- rn:R07206 <-- [Oxidized NADPH---hemoprotein reductase] --> rn:R08551

rn:R07777 { Liquiritigenin } <-- [Reduced NADPH---hemoprotein reductase] -- rn:R07206 <-- [Oxidized NADPH---hemoprotein reductase] --> rn:R08551

rn:R08002 { Liquiritigenin } <-- [Reduced NADPH---hemoprotein reductase] -- rn:R07206 <-- [Oxidized NADPH---hemoprotein reductase] --> rn:R08551

rn:R07712 { Liquiritigenin } <-- 2-Oxoglutarate -- rn:R07276 <-- L-Glutamate --> rn:R00114

rn:R07996 { Liquiritigenin } <-- 2-Oxoglutarate -- rn:R07276 <-- L-Glutamate --> rn:R00114

rn:R07712 { Liquiritigenin } <-- 2-Oxoglutarate -- rn:R07276 <-- L-Glutamate --> rn:R00248

rn:R07996 { Liquiritigenin } <-- 2-Oxoglutarate -- rn:R07276 <-- L-Glutamate --> rn:R00248

rn:R07712 { Liquiritigenin } <-- 2-Oxoglutarate -- rn:R07276 <-- L-Glutamate --> rn:R00254

rn:R07996 { Liquiritigenin } <-- 2-Oxoglutarate -- rn:R07276 <-- L-Glutamate --> rn:R00254

rn:R07712 { Liquiritigenin } <-- 2-Oxoglutarate -- rn:R07276 <-- L-Glutamate --> rn:R00894

rn:R07996 { Liquiritigenin } <-- 2-Oxoglutarate -- rn:R07276 <-- L-Glutamate --> rn:R00894

rn:R07712 { Liquiritigenin } <-- 2-Oxoglutarate -- rn:R07276 <-- Prephenate --> rn:R01728

rn:R07996 { Liquiritigenin } <-- 2-Oxoglutarate -- rn:R07276 <-- Prephenate --> rn:R01728

rn:R07712 { Liquiritigenin } <-- 2-Oxoglutarate -- rn:R07276 <-- Prephenate --> rn:R01730

rn:R07996 { Liquiritigenin } <-- 2-Oxoglutarate -- rn:R07276 <-- Prephenate --> rn:R01730

rn:R07712 { Liquiritigenin } <-- 2-Oxoglutarate -- rn:R07276 <-- L-Glutamate --> rn:R02287

rn:R07996 { Liquiritigenin } <-- 2-Oxoglutarate -- rn:R07276 <-- L-Glutamate --> rn:R02287

rn:R07712 { Liquiritigenin } <-- 2-Oxoglutarate -- rn:R07276 <-- L-Glutamate --> rn:R03189

rn:R07996 { Liquiritigenin } <-- 2-Oxoglutarate -- rn:R07276 <-- L-Glutamate --> rn:R03189

rn:R07712 { Liquiritigenin } <-- 2-Oxoglutarate -- rn:R07276 <-- L-Glutamate --> rn:R03970

rn:R07996 { Liquiritigenin } <-- 2-Oxoglutarate -- rn:R07276 <-- L-Glutamate --> rn:R03970

rn:R07712 { Liquiritigenin } <-- 2-Oxoglutarate -- rn:R07276 <-- L-Glutamate --> rn:R03971

rn:R07996 { Liquiritigenin } <-- 2-Oxoglutarate -- rn:R07276 <-- L-Glutamate --> rn:R03971

rn:R07712 { Liquiritigenin } <-- 2-Oxoglutarate -- rn:R07276 <-- L-Glutamate --> rn:R04051

rn:R07996 { Liquiritigenin } <-- 2-Oxoglutarate -- rn:R07276 <-- L-Glutamate --> rn:R04051

rn:R07712 { Liquiritigenin } <-- 2-Oxoglutarate -- rn:R07276 <-- L-Glutamate --> rn:R04776

rn:R07996 { Liquiritigenin } <-- 2-Oxoglutarate -- rn:R07276 <-- L-Glutamate --> rn:R04776

rn:R07712 { Liquiritigenin } <-- 2-Oxoglutarate -- rn:R07276 <-- L-Glutamate --> rn:R07643

rn:R07996 { Liquiritigenin } <-- 2-Oxoglutarate -- rn:R07276 <-- L-Glutamate --> rn:R07643

rn:R07712 { Liquiritigenin } <-- 2-Oxoglutarate -- rn:R07276 <-- Prephenate --> rn:R10934

rn:R07996 { Liquiritigenin } <-- 2-Oxoglutarate -- rn:R07276 <-- Prephenate --> rn:R10934

rn:R03006 { Formononetin } <-- [Reduced NADPH---hemoprotein reductase] -- rn:R07403 <-- [Oxidized NADPH---hemoprotein reductase] --> rn:R08551

rn:R03615 { Flavonoid } <-- [Reduced NADPH---hemoprotein reductase] -- rn:R07403 <-- [Oxidized NADPH---hemoprotein reductase] --> rn:R08551

rn:R06560 { Formononetin } <-- [Reduced NADPH---hemoprotein reductase] -- rn:R07403 <-- [Oxidized NADPH---hemoprotein reductase] --> rn:R08551

rn:R07198 { Liquiritigenin } <-- [Reduced NADPH---hemoprotein reductase] -- rn:R07403 <-- [Oxidized NADPH---hemoprotein reductase] --> rn:R08551

rn:R07745 { Calycosin } <-- [Reduced NADPH---hemoprotein reductase] -- rn:R07403 <-- [Oxidized NADPH---hemoprotein reductase] --> rn:R08551

rn:R07777 { Liquiritigenin } <-- [Reduced NADPH---hemoprotein reductase] -- rn:R07403 <-- [Oxidized NADPH---hemoprotein reductase] --> rn:R08551

rn:R08002 { Liquiritigenin } <-- [Reduced NADPH---hemoprotein reductase] -- rn:R07403 <-- [Oxidized NADPH---hemoprotein reductase] --> rn:R08551

rn:R07712 { Liquiritigenin } <-- 2-Oxoglutarate -- rn:R08165 <-- 2-Succinyl-5-enolpyruvyl-6-hydroxy-3-cyclohexene-1-carboxylate --> rn:R08166

rn:R07996 { Liquiritigenin } <-- 2-Oxoglutarate -- rn:R08165 <-- 2-Succinyl-5-enolpyruvyl-6-hydroxy-3-cyclohexene-1-carboxylate --> rn:R08166

rn:R03006 { Formononetin } <-- [Reduced NADPH---hemoprotein reductase] -- rn:R08652 <-- (Z)-Phenylacetaldehyde oxime --> rn:R07638

rn:R03615 { Flavonoid } <-- [Reduced NADPH---hemoprotein reductase] -- rn:R08652 <-- (Z)-Phenylacetaldehyde oxime --> rn:R07638

rn:R06560 { Formononetin } <-- [Reduced NADPH---hemoprotein reductase] -- rn:R08652 <-- (Z)-Phenylacetaldehyde oxime --> rn:R07638

rn:R07198 { Liquiritigenin } <-- [Reduced NADPH---hemoprotein reductase] -- rn:R08652 <-- (Z)-Phenylacetaldehyde oxime --> rn:R07638

rn:R07745 { Calycosin } <-- [Reduced NADPH---hemoprotein reductase] -- rn:R08652 <-- (Z)-Phenylacetaldehyde oxime --> rn:R07638

rn:R07777 { Liquiritigenin } <-- [Reduced NADPH---hemoprotein reductase] -- rn:R08652 <-- (Z)-Phenylacetaldehyde oxime --> rn:R07638

rn:R08002 { Liquiritigenin } <-- [Reduced NADPH---hemoprotein reductase] -- rn:R08652 <-- (Z)-Phenylacetaldehyde oxime --> rn:R07638

rn:R03006 { Formononetin } <-- [Reduced NADPH---hemoprotein reductase] -- rn:R08652 <-- (Z)-Phenylacetaldehyde oxime --> rn:R08653

rn:R03615 { Flavonoid } <-- [Reduced NADPH---hemoprotein reductase] -- rn:R08652 <-- (Z)-Phenylacetaldehyde oxime --> rn:R08653

rn:R06560 { Formononetin } <-- [Reduced NADPH---hemoprotein reductase] -- rn:R08652 <-- (Z)-Phenylacetaldehyde oxime --> rn:R08653

rn:R07198 { Liquiritigenin } <-- [Reduced NADPH---hemoprotein reductase] -- rn:R08652 <-- (Z)-Phenylacetaldehyde oxime --> rn:R08653

rn:R07745 { Calycosin } <-- [Reduced NADPH---hemoprotein reductase] -- rn:R08652 <-- (Z)-Phenylacetaldehyde oxime --> rn:R08653

rn:R07777 { Liquiritigenin } <-- [Reduced NADPH---hemoprotein reductase] -- rn:R08652 <-- (Z)-Phenylacetaldehyde oxime --> rn:R08653

rn:R08002 { Liquiritigenin } <-- [Reduced NADPH---hemoprotein reductase] -- rn:R08652 <-- (Z)-Phenylacetaldehyde oxime --> rn:R08653

rn:R03006 { Formononetin } <-- [Reduced NADPH---hemoprotein reductase] -- rn:R08652 <-- (Z)-Phenylacetaldehyde oxime --> rn:R10637

rn:R03615 { Flavonoid } <-- [Reduced NADPH---hemoprotein reductase] -- rn:R08652 <-- (Z)-Phenylacetaldehyde oxime --> rn:R10637

rn:R06560 { Formononetin } <-- [Reduced NADPH---hemoprotein reductase] -- rn:R08652 <-- (Z)-Phenylacetaldehyde oxime --> rn:R10637

rn:R07198 { Liquiritigenin } <-- [Reduced NADPH---hemoprotein reductase] -- rn:R08652 <-- (Z)-Phenylacetaldehyde oxime --> rn:R10637

rn:R07745 { Calycosin } <-- [Reduced NADPH---hemoprotein reductase] -- rn:R08652 <-- (Z)-Phenylacetaldehyde oxime --> rn:R10637

rn:R07777 { Liquiritigenin } <-- [Reduced NADPH---hemoprotein reductase] -- rn:R08652 <-- (Z)-Phenylacetaldehyde oxime --> rn:R10637

rn:R08002 { Liquiritigenin } <-- [Reduced NADPH---hemoprotein reductase] -- rn:R08652 <-- (Z)-Phenylacetaldehyde oxime --> rn:R10637

rn:R03006 { Formononetin } <-- [Reduced NADPH---hemoprotein reductase] -- rn:R08663 <-- (E)-2-Methylpropanal oxime --> rn:R04169

rn:R03615 { Flavonoid } <-- [Reduced NADPH---hemoprotein reductase] -- rn:R08663 <-- (E)-2-Methylpropanal oxime --> rn:R04169

rn:R06560 { Formononetin } <-- [Reduced NADPH---hemoprotein reductase] -- rn:R08663 <-- (E)-2-Methylpropanal oxime --> rn:R04169

rn:R07198 { Liquiritigenin } <-- [Reduced NADPH---hemoprotein reductase] -- rn:R08663 <-- (E)-2-Methylpropanal oxime --> rn:R04169

rn:R07745 { Calycosin } <-- [Reduced NADPH---hemoprotein reductase] -- rn:R08663 <-- (E)-2-Methylpropanal oxime --> rn:R04169

rn:R07777 { Liquiritigenin } <-- [Reduced NADPH---hemoprotein reductase] -- rn:R08663 <-- (E)-2-Methylpropanal oxime --> rn:R04169

rn:R08002 { Liquiritigenin } <-- [Reduced NADPH---hemoprotein reductase] -- rn:R08663 <-- (E)-2-Methylpropanal oxime --> rn:R04169

rn:R03006 { Formononetin } <-- [Reduced NADPH---hemoprotein reductase] -- rn:R08663 <-- [Oxidized NADPH---hemoprotein reductase] --> rn:R08551

rn:R03615 { Flavonoid } <-- [Reduced NADPH---hemoprotein reductase] -- rn:R08663 <-- [Oxidized NADPH---hemoprotein reductase] --> rn:R08551

rn:R06560 { Formononetin } <-- [Reduced NADPH---hemoprotein reductase] -- rn:R08663 <-- [Oxidized NADPH---hemoprotein reductase] --> rn:R08551

rn:R07198 { Liquiritigenin } <-- [Reduced NADPH---hemoprotein reductase] -- rn:R08663 <-- [Oxidized NADPH---hemoprotein reductase] --> rn:R08551

rn:R07745 { Calycosin } <-- [Reduced NADPH---hemoprotein reductase] -- rn:R08663 <-- [Oxidized NADPH---hemoprotein reductase] --> rn:R08551

rn:R07777 { Liquiritigenin } <-- [Reduced NADPH---hemoprotein reductase] -- rn:R08663 <-- [Oxidized NADPH---hemoprotein reductase] --> rn:R08551

rn:R08002 { Liquiritigenin } <-- [Reduced NADPH---hemoprotein reductase] -- rn:R08663 <-- [Oxidized NADPH---hemoprotein reductase] --> rn:R08551

rn:R03006 { Formononetin } <-- [Reduced NADPH---hemoprotein reductase] -- rn:R08663 <-- (E)-2-Methylpropanal oxime --> rn:R10034

rn:R03615 { Flavonoid } <-- [Reduced NADPH---hemoprotein reductase] -- rn:R08663 <-- (E)-2-Methylpropanal oxime --> rn:R10034

rn:R06560 { Formononetin } <-- [Reduced NADPH---hemoprotein reductase] -- rn:R08663 <-- (E)-2-Methylpropanal oxime --> rn:R10034

rn:R07198 { Liquiritigenin } <-- [Reduced NADPH---hemoprotein reductase] -- rn:R08663 <-- (E)-2-Methylpropanal oxime --> rn:R10034

rn:R07745 { Calycosin } <-- [Reduced NADPH---hemoprotein reductase] -- rn:R08663 <-- (E)-2-Methylpropanal oxime --> rn:R10034

rn:R07777 { Liquiritigenin } <-- [Reduced NADPH---hemoprotein reductase] -- rn:R08663 <-- (E)-2-Methylpropanal oxime --> rn:R10034

rn:R08002 { Liquiritigenin } <-- [Reduced NADPH---hemoprotein reductase] -- rn:R08663 <-- (E)-2-Methylpropanal oxime --> rn:R10034

rn:R03006 { Formononetin } <-- [Reduced NADPH---hemoprotein reductase] -- rn:R08663 <-- (E)-2-Methylpropanal oxime --> rn:R11598

rn:R03615 { Flavonoid } <-- [Reduced NADPH---hemoprotein reductase] -- rn:R08663 <-- (E)-2-Methylpropanal oxime --> rn:R11598

rn:R06560 { Formononetin } <-- [Reduced NADPH---hemoprotein reductase] -- rn:R08663 <-- (E)-2-Methylpropanal oxime --> rn:R11598

rn:R07198 { Liquiritigenin } <-- [Reduced NADPH---hemoprotein reductase] -- rn:R08663 <-- (E)-2-Methylpropanal oxime --> rn:R11598

rn:R07745 { Calycosin } <-- [Reduced NADPH---hemoprotein reductase] -- rn:R08663 <-- (E)-2-Methylpropanal oxime --> rn:R11598

rn:R07777 { Liquiritigenin } <-- [Reduced NADPH---hemoprotein reductase] -- rn:R08663 <-- (E)-2-Methylpropanal oxime --> rn:R11598

rn:R08002 { Liquiritigenin } <-- [Reduced NADPH---hemoprotein reductase] -- rn:R08663 <-- (E)-2-Methylpropanal oxime --> rn:R11598

rn:R03006 { Formononetin } <-- [Reduced NADPH---hemoprotein reductase] -- rn:R09403 <-- [Oxidized NADPH---hemoprotein reductase] --> rn:R08551

rn:R03615 { Flavonoid } <-- [Reduced NADPH---hemoprotein reductase] -- rn:R09403 <-- [Oxidized NADPH---hemoprotein reductase] --> rn:R08551

rn:R06560 { Formononetin } <-- [Reduced NADPH---hemoprotein reductase] -- rn:R09403 <-- [Oxidized NADPH---hemoprotein reductase] --> rn:R08551

rn:R07198 { Liquiritigenin } <-- [Reduced NADPH---hemoprotein reductase] -- rn:R09403 <-- [Oxidized NADPH---hemoprotein reductase] --> rn:R08551

rn:R07745 { Calycosin } <-- [Reduced NADPH---hemoprotein reductase] -- rn:R09403 <-- [Oxidized NADPH---hemoprotein reductase] --> rn:R08551

rn:R07777 { Liquiritigenin } <-- [Reduced NADPH---hemoprotein reductase] -- rn:R09403 <-- [Oxidized NADPH---hemoprotein reductase] --> rn:R08551

rn:R08002 { Liquiritigenin } <-- [Reduced NADPH---hemoprotein reductase] -- rn:R09403 <-- [Oxidized NADPH---hemoprotein reductase] --> rn:R08551

rn:R03006 { Formononetin } <-- [Reduced NADPH---hemoprotein reductase] -- rn:R09403 <-- (E)-2-Methylbutanal oxime --> rn:R10030

rn:R03615 { Flavonoid } <-- [Reduced NADPH---hemoprotein reductase] -- rn:R09403 <-- (E)-2-Methylbutanal oxime --> rn:R10030

rn:R06560 { Formononetin } <-- [Reduced NADPH---hemoprotein reductase] -- rn:R09403 <-- (E)-2-Methylbutanal oxime --> rn:R10030

rn:R07198 { Liquiritigenin } <-- [Reduced NADPH---hemoprotein reductase] -- rn:R09403 <-- (E)-2-Methylbutanal oxime --> rn:R10030

rn:R07745 { Calycosin } <-- [Reduced NADPH---hemoprotein reductase] -- rn:R09403 <-- (E)-2-Methylbutanal oxime --> rn:R10030

rn:R07777 { Liquiritigenin } <-- [Reduced NADPH---hemoprotein reductase] -- rn:R09403 <-- (E)-2-Methylbutanal oxime --> rn:R10030

rn:R08002 { Liquiritigenin } <-- [Reduced NADPH---hemoprotein reductase] -- rn:R09403 <-- (E)-2-Methylbutanal oxime --> rn:R10030

rn:R03006 { Formononetin } <-- [Reduced NADPH---hemoprotein reductase] -- rn:R09403 <-- (E)-2-Methylbutanal oxime --> rn:R11597

rn:R03615 { Flavonoid } <-- [Reduced NADPH---hemoprotein reductase] -- rn:R09403 <-- (E)-2-Methylbutanal oxime --> rn:R11597

rn:R06560 { Formononetin } <-- [Reduced NADPH---hemoprotein reductase] -- rn:R09403 <-- (E)-2-Methylbutanal oxime --> rn:R11597

rn:R07198 { Liquiritigenin } <-- [Reduced NADPH---hemoprotein reductase] -- rn:R09403 <-- (E)-2-Methylbutanal oxime --> rn:R11597

rn:R07745 { Calycosin } <-- [Reduced NADPH---hemoprotein reductase] -- rn:R09403 <-- (E)-2-Methylbutanal oxime --> rn:R11597

rn:R07777 { Liquiritigenin } <-- [Reduced NADPH---hemoprotein reductase] -- rn:R09403 <-- (E)-2-Methylbutanal oxime --> rn:R11597

rn:R08002 { Liquiritigenin } <-- [Reduced NADPH---hemoprotein reductase] -- rn:R09403 <-- (E)-2-Methylbutanal oxime --> rn:R11597

rn:R03006 { Formononetin } <-- [Reduced NADPH---hemoprotein reductase] -- rn:R09578 <-- [Oxidized NADPH---hemoprotein reductase] --> rn:R08551

rn:R03615 { Flavonoid } <-- [Reduced NADPH---hemoprotein reductase] -- rn:R09578 <-- [Oxidized NADPH---hemoprotein reductase] --> rn:R08551

rn:R06560 { Formononetin } <-- [Reduced NADPH---hemoprotein reductase] -- rn:R09578 <-- [Oxidized NADPH---hemoprotein reductase] --> rn:R08551

rn:R07198 { Liquiritigenin } <-- [Reduced NADPH---hemoprotein reductase] -- rn:R09578 <-- [Oxidized NADPH---hemoprotein reductase] --> rn:R08551

rn:R07745 { Calycosin } <-- [Reduced NADPH---hemoprotein reductase] -- rn:R09578 <-- [Oxidized NADPH---hemoprotein reductase] --> rn:R08551

rn:R07777 { Liquiritigenin } <-- [Reduced NADPH---hemoprotein reductase] -- rn:R09578 <-- [Oxidized NADPH---hemoprotein reductase] --> rn:R08551

rn:R08002 { Liquiritigenin } <-- [Reduced NADPH---hemoprotein reductase] -- rn:R09578 <-- [Oxidized NADPH---hemoprotein reductase] --> rn:R08551

rn:R03006 { Formononetin } <-- [Reduced NADPH---hemoprotein reductase] -- rn:R09578 <-- (E)-Phenylacetaldoxime --> rn:R10041

rn:R03615 { Flavonoid } <-- [Reduced NADPH---hemoprotein reductase] -- rn:R09578 <-- (E)-Phenylacetaldoxime --> rn:R10041

rn:R06560 { Formononetin } <-- [Reduced NADPH---hemoprotein reductase] -- rn:R09578 <-- (E)-Phenylacetaldoxime --> rn:R10041

rn:R07198 { Liquiritigenin } <-- [Reduced NADPH---hemoprotein reductase] -- rn:R09578 <-- (E)-Phenylacetaldoxime --> rn:R10041

rn:R07745 { Calycosin } <-- [Reduced NADPH---hemoprotein reductase] -- rn:R09578 <-- (E)-Phenylacetaldoxime --> rn:R10041

rn:R07777 { Liquiritigenin } <-- [Reduced NADPH---hemoprotein reductase] -- rn:R09578 <-- (E)-Phenylacetaldoxime --> rn:R10041

rn:R08002 { Liquiritigenin } <-- [Reduced NADPH---hemoprotein reductase] -- rn:R09578 <-- (E)-Phenylacetaldoxime --> rn:R10041

rn:R03006 { Formononetin } <-- [Reduced NADPH---hemoprotein reductase] -- rn:R09578 <-- (E)-Phenylacetaldoxime --> rn:R10670

rn:R03615 { Flavonoid } <-- [Reduced NADPH---hemoprotein reductase] -- rn:R09578 <-- (E)-Phenylacetaldoxime --> rn:R10670

rn:R06560 { Formononetin } <-- [Reduced NADPH---hemoprotein reductase] -- rn:R09578 <-- (E)-Phenylacetaldoxime --> rn:R10670

rn:R07198 { Liquiritigenin } <-- [Reduced NADPH---hemoprotein reductase] -- rn:R09578 <-- (E)-Phenylacetaldoxime --> rn:R10670

rn:R07745 { Calycosin } <-- [Reduced NADPH---hemoprotein reductase] -- rn:R09578 <-- (E)-Phenylacetaldoxime --> rn:R10670

rn:R07777 { Liquiritigenin } <-- [Reduced NADPH---hemoprotein reductase] -- rn:R09578 <-- (E)-Phenylacetaldoxime --> rn:R10670

rn:R08002 { Liquiritigenin } <-- [Reduced NADPH---hemoprotein reductase] -- rn:R09578 <-- (E)-Phenylacetaldoxime --> rn:R10670

rn:R03006 { Formononetin } <-- [Reduced NADPH---hemoprotein reductase] -- rn:R09578 <-- (E)-Phenylacetaldoxime --> rn:R11732

rn:R03615 { Flavonoid } <-- [Reduced NADPH---hemoprotein reductase] -- rn:R09578 <-- (E)-Phenylacetaldoxime --> rn:R11732

rn:R06560 { Formononetin } <-- [Reduced NADPH---hemoprotein reductase] -- rn:R09578 <-- (E)-Phenylacetaldoxime --> rn:R11732

rn:R07198 { Liquiritigenin } <-- [Reduced NADPH---hemoprotein reductase] -- rn:R09578 <-- (E)-Phenylacetaldoxime --> rn:R11732

rn:R07745 { Calycosin } <-- [Reduced NADPH---hemoprotein reductase] -- rn:R09578 <-- (E)-Phenylacetaldoxime --> rn:R11732

rn:R07777 { Liquiritigenin } <-- [Reduced NADPH---hemoprotein reductase] -- rn:R09578 <-- (E)-Phenylacetaldoxime --> rn:R11732

rn:R08002 { Liquiritigenin } <-- [Reduced NADPH---hemoprotein reductase] -- rn:R09578 <-- (E)-Phenylacetaldoxime --> rn:R11732

rn:R03006 { Formononetin } <-- [Reduced NADPH---hemoprotein reductase] -- rn:R09578 <-- (E)-Phenylacetaldoxime --> rn:R11737

rn:R03615 { Flavonoid } <-- [Reduced NADPH---hemoprotein reductase] -- rn:R09578 <-- (E)-Phenylacetaldoxime --> rn:R11737

rn:R06560 { Formononetin } <-- [Reduced NADPH---hemoprotein reductase] -- rn:R09578 <-- (E)-Phenylacetaldoxime --> rn:R11737

rn:R07198 { Liquiritigenin } <-- [Reduced NADPH---hemoprotein reductase] -- rn:R09578 <-- (E)-Phenylacetaldoxime --> rn:R11737

rn:R07745 { Calycosin } <-- [Reduced NADPH---hemoprotein reductase] -- rn:R09578 <-- (E)-Phenylacetaldoxime --> rn:R11737

rn:R07777 { Liquiritigenin } <-- [Reduced NADPH---hemoprotein reductase] -- rn:R09578 <-- (E)-Phenylacetaldoxime --> rn:R11737

rn:R08002 { Liquiritigenin } <-- [Reduced NADPH---hemoprotein reductase] -- rn:R09578 <-- (E)-Phenylacetaldoxime --> rn:R11737

rn:R03006 { Formononetin } <-- [Reduced NADPH---hemoprotein reductase] -- rn:R09578 <-- (E)-Phenylacetaldoxime --> rn:R11738

rn:R03615 { Flavonoid } <-- [Reduced NADPH---hemoprotein reductase] -- rn:R09578 <-- (E)-Phenylacetaldoxime --> rn:R11738

rn:R06560 { Formononetin } <-- [Reduced NADPH---hemoprotein reductase] -- rn:R09578 <-- (E)-Phenylacetaldoxime --> rn:R11738

rn:R07198 { Liquiritigenin } <-- [Reduced NADPH---hemoprotein reductase] -- rn:R09578 <-- (E)-Phenylacetaldoxime --> rn:R11738

rn:R07745 { Calycosin } <-- [Reduced NADPH---hemoprotein reductase] -- rn:R09578 <-- (E)-Phenylacetaldoxime --> rn:R11738

rn:R07777 { Liquiritigenin } <-- [Reduced NADPH---hemoprotein reductase] -- rn:R09578 <-- (E)-Phenylacetaldoxime --> rn:R11738

rn:R08002 { Liquiritigenin } <-- [Reduced NADPH---hemoprotein reductase] -- rn:R09578 <-- (E)-Phenylacetaldoxime --> rn:R11738

rn:R03006 { Formononetin } <-- [Reduced NADPH---hemoprotein reductase] -- rn:R09579 <-- [Oxidized NADPH---hemoprotein reductase] --> rn:R08551

rn:R03615 { Flavonoid } <-- [Reduced NADPH---hemoprotein reductase] -- rn:R09579 <-- [Oxidized NADPH---hemoprotein reductase] --> rn:R08551

rn:R06560 { Formononetin } <-- [Reduced NADPH---hemoprotein reductase] -- rn:R09579 <-- [Oxidized NADPH---hemoprotein reductase] --> rn:R08551

rn:R07198 { Liquiritigenin } <-- [Reduced NADPH---hemoprotein reductase] -- rn:R09579 <-- [Oxidized NADPH---hemoprotein reductase] --> rn:R08551

rn:R07745 { Calycosin } <-- [Reduced NADPH---hemoprotein reductase] -- rn:R09579 <-- [Oxidized NADPH---hemoprotein reductase] --> rn:R08551

rn:R07777 { Liquiritigenin } <-- [Reduced NADPH---hemoprotein reductase] -- rn:R09579 <-- [Oxidized NADPH---hemoprotein reductase] --> rn:R08551

rn:R08002 { Liquiritigenin } <-- [Reduced NADPH---hemoprotein reductase] -- rn:R09579 <-- [Oxidized NADPH---hemoprotein reductase] --> rn:R08551

rn:R03006 { Formononetin } <-- [Reduced NADPH---hemoprotein reductase] -- rn:R09579 <-- N-Hydroxy-L-phenylalanine --> rn:R09580

rn:R03615 { Flavonoid } <-- [Reduced NADPH---hemoprotein reductase] -- rn:R09579 <-- N-Hydroxy-L-phenylalanine --> rn:R09580

rn:R06560 { Formononetin } <-- [Reduced NADPH---hemoprotein reductase] -- rn:R09579 <-- N-Hydroxy-L-phenylalanine --> rn:R09580

rn:R07198 { Liquiritigenin } <-- [Reduced NADPH---hemoprotein reductase] -- rn:R09579 <-- N-Hydroxy-L-phenylalanine --> rn:R09580

rn:R07745 { Calycosin } <-- [Reduced NADPH---hemoprotein reductase] -- rn:R09579 <-- N-Hydroxy-L-phenylalanine --> rn:R09580

rn:R07777 { Liquiritigenin } <-- [Reduced NADPH---hemoprotein reductase] -- rn:R09579 <-- N-Hydroxy-L-phenylalanine --> rn:R09580

rn:R08002 { Liquiritigenin } <-- [Reduced NADPH---hemoprotein reductase] -- rn:R09579 <-- N-Hydroxy-L-phenylalanine --> rn:R09580

rn:R03006 { Formononetin } <-- [Reduced NADPH---hemoprotein reductase] -- rn:R09580 <-- [Oxidized NADPH---hemoprotein reductase] --> rn:R08551

rn:R03615 { Flavonoid } <-- [Reduced NADPH---hemoprotein reductase] -- rn:R09580 <-- [Oxidized NADPH---hemoprotein reductase] --> rn:R08551

rn:R06560 { Formononetin } <-- [Reduced NADPH---hemoprotein reductase] -- rn:R09580 <-- [Oxidized NADPH---hemoprotein reductase] --> rn:R08551

rn:R07198 { Liquiritigenin } <-- [Reduced NADPH---hemoprotein reductase] -- rn:R09580 <-- [Oxidized NADPH---hemoprotein reductase] --> rn:R08551

rn:R07745 { Calycosin } <-- [Reduced NADPH---hemoprotein reductase] -- rn:R09580 <-- [Oxidized NADPH---hemoprotein reductase] --> rn:R08551

rn:R07777 { Liquiritigenin } <-- [Reduced NADPH---hemoprotein reductase] -- rn:R09580 <-- [Oxidized NADPH---hemoprotein reductase] --> rn:R08551

rn:R08002 { Liquiritigenin } <-- [Reduced NADPH---hemoprotein reductase] -- rn:R09580 <-- [Oxidized NADPH---hemoprotein reductase] --> rn:R08551

rn:R03006 { Formononetin } <-- [Reduced NADPH---hemoprotein reductase] -- rn:R09580 <-- N,N-Dihydroxy-L-phenylalanine --> rn:R09581

rn:R03615 { Flavonoid } <-- [Reduced NADPH---hemoprotein reductase] -- rn:R09580 <-- N,N-Dihydroxy-L-phenylalanine --> rn:R09581

rn:R06560 { Formononetin } <-- [Reduced NADPH---hemoprotein reductase] -- rn:R09580 <-- N,N-Dihydroxy-L-phenylalanine --> rn:R09581

rn:R07198 { Liquiritigenin } <-- [Reduced NADPH---hemoprotein reductase] -- rn:R09580 <-- N,N-Dihydroxy-L-phenylalanine --> rn:R09581

rn:R07745 { Calycosin } <-- [Reduced NADPH---hemoprotein reductase] -- rn:R09580 <-- N,N-Dihydroxy-L-phenylalanine --> rn:R09581

rn:R07777 { Liquiritigenin } <-- [Reduced NADPH---hemoprotein reductase] -- rn:R09580 <-- N,N-Dihydroxy-L-phenylalanine --> rn:R09581

rn:R08002 { Liquiritigenin } <-- [Reduced NADPH---hemoprotein reductase] -- rn:R09580 <-- N,N-Dihydroxy-L-phenylalanine --> rn:R09581

rn:R07712 { Liquiritigenin } <-- 2-Oxoglutarate -- rn:R09824 <-- L-Glutamate --> rn:R00114

rn:R07996 { Liquiritigenin } <-- 2-Oxoglutarate -- rn:R09824 <-- L-Glutamate --> rn:R00114

rn:R07712 { Liquiritigenin } <-- 2-Oxoglutarate -- rn:R09824 <-- L-Glutamate --> rn:R00248

rn:R07996 { Liquiritigenin } <-- 2-Oxoglutarate -- rn:R09824 <-- L-Glutamate --> rn:R00248

rn:R07712 { Liquiritigenin } <-- 2-Oxoglutarate -- rn:R09824 <-- L-Glutamate --> rn:R00254

rn:R07996 { Liquiritigenin } <-- 2-Oxoglutarate -- rn:R09824 <-- L-Glutamate --> rn:R00254

rn:R07712 { Liquiritigenin } <-- 2-Oxoglutarate -- rn:R09824 <-- L-Glutamate --> rn:R00894

rn:R07996 { Liquiritigenin } <-- 2-Oxoglutarate -- rn:R09824 <-- L-Glutamate --> rn:R00894

rn:R07712 { Liquiritigenin } <-- 2-Oxoglutarate -- rn:R09824 <-- L-Glutamate --> rn:R02287

rn:R07996 { Liquiritigenin } <-- 2-Oxoglutarate -- rn:R09824 <-- L-Glutamate --> rn:R02287

rn:R07712 { Liquiritigenin } <-- 2-Oxoglutarate -- rn:R09824 <-- L-Glutamate --> rn:R03189

rn:R07996 { Liquiritigenin } <-- 2-Oxoglutarate -- rn:R09824 <-- L-Glutamate --> rn:R03189

rn:R07712 { Liquiritigenin } <-- 2-Oxoglutarate -- rn:R09824 <-- L-Glutamate --> rn:R03970

rn:R07996 { Liquiritigenin } <-- 2-Oxoglutarate -- rn:R09824 <-- L-Glutamate --> rn:R03970

rn:R07712 { Liquiritigenin } <-- 2-Oxoglutarate -- rn:R09824 <-- L-Glutamate --> rn:R03971

rn:R07996 { Liquiritigenin } <-- 2-Oxoglutarate -- rn:R09824 <-- L-Glutamate --> rn:R03971

rn:R07712 { Liquiritigenin } <-- 2-Oxoglutarate -- rn:R09824 <-- L-Glutamate --> rn:R04051

rn:R07996 { Liquiritigenin } <-- 2-Oxoglutarate -- rn:R09824 <-- L-Glutamate --> rn:R04051

rn:R07712 { Liquiritigenin } <-- 2-Oxoglutarate -- rn:R09824 <-- L-Glutamate --> rn:R04776

rn:R07996 { Liquiritigenin } <-- 2-Oxoglutarate -- rn:R09824 <-- L-Glutamate --> rn:R04776

rn:R07712 { Liquiritigenin } <-- 2-Oxoglutarate -- rn:R09824 <-- L-Glutamate --> rn:R07643

rn:R07996 { Liquiritigenin } <-- 2-Oxoglutarate -- rn:R09824 <-- L-Glutamate --> rn:R07643

rn:R03006 { Formononetin } <-- [Reduced NADPH---hemoprotein reductase] -- rn:R10027 <-- [Oxidized NADPH---hemoprotein reductase] --> rn:R08551

rn:R03615 { Flavonoid } <-- [Reduced NADPH---hemoprotein reductase] -- rn:R10027 <-- [Oxidized NADPH---hemoprotein reductase] --> rn:R08551

rn:R06560 { Formononetin } <-- [Reduced NADPH---hemoprotein reductase] -- rn:R10027 <-- [Oxidized NADPH---hemoprotein reductase] --> rn:R08551

rn:R07198 { Liquiritigenin } <-- [Reduced NADPH---hemoprotein reductase] -- rn:R10027 <-- [Oxidized NADPH---hemoprotein reductase] --> rn:R08551

rn:R07745 { Calycosin } <-- [Reduced NADPH---hemoprotein reductase] -- rn:R10027 <-- [Oxidized NADPH---hemoprotein reductase] --> rn:R08551

rn:R07777 { Liquiritigenin } <-- [Reduced NADPH---hemoprotein reductase] -- rn:R10027 <-- [Oxidized NADPH---hemoprotein reductase] --> rn:R08551

rn:R08002 { Liquiritigenin } <-- [Reduced NADPH---hemoprotein reductase] -- rn:R10027 <-- [Oxidized NADPH---hemoprotein reductase] --> rn:R08551

rn:R03006 { Formononetin } <-- [Reduced NADPH---hemoprotein reductase] -- rn:R10027 <-- N-Hydroxy-L-isoleucine --> rn:R10028

rn:R03615 { Flavonoid } <-- [Reduced NADPH---hemoprotein reductase] -- rn:R10027 <-- N-Hydroxy-L-isoleucine --> rn:R10028

rn:R06560 { Formononetin } <-- [Reduced NADPH---hemoprotein reductase] -- rn:R10027 <-- N-Hydroxy-L-isoleucine --> rn:R10028

rn:R07198 { Liquiritigenin } <-- [Reduced NADPH---hemoprotein reductase] -- rn:R10027 <-- N-Hydroxy-L-isoleucine --> rn:R10028

rn:R07745 { Calycosin } <-- [Reduced NADPH---hemoprotein reductase] -- rn:R10027 <-- N-Hydroxy-L-isoleucine --> rn:R10028

rn:R07777 { Liquiritigenin } <-- [Reduced NADPH---hemoprotein reductase] -- rn:R10027 <-- N-Hydroxy-L-isoleucine --> rn:R10028

rn:R08002 { Liquiritigenin } <-- [Reduced NADPH---hemoprotein reductase] -- rn:R10027 <-- N-Hydroxy-L-isoleucine --> rn:R10028

rn:R03006 { Formononetin } <-- [Reduced NADPH---hemoprotein reductase] -- rn:R10028 <-- [Oxidized NADPH---hemoprotein reductase] --> rn:R08551

rn:R03615 { Flavonoid } <-- [Reduced NADPH---hemoprotein reductase] -- rn:R10028 <-- [Oxidized NADPH---hemoprotein reductase] --> rn:R08551

rn:R06560 { Formononetin } <-- [Reduced NADPH---hemoprotein reductase] -- rn:R10028 <-- [Oxidized NADPH---hemoprotein reductase] --> rn:R08551

rn:R07198 { Liquiritigenin } <-- [Reduced NADPH---hemoprotein reductase] -- rn:R10028 <-- [Oxidized NADPH---hemoprotein reductase] --> rn:R08551

rn:R07745 { Calycosin } <-- [Reduced NADPH---hemoprotein reductase] -- rn:R10028 <-- [Oxidized NADPH---hemoprotein reductase] --> rn:R08551

rn:R07777 { Liquiritigenin } <-- [Reduced NADPH---hemoprotein reductase] -- rn:R10028 <-- [Oxidized NADPH---hemoprotein reductase] --> rn:R08551

rn:R08002 { Liquiritigenin } <-- [Reduced NADPH---hemoprotein reductase] -- rn:R10028 <-- [Oxidized NADPH---hemoprotein reductase] --> rn:R08551

rn:R03006 { Formononetin } <-- [Reduced NADPH---hemoprotein reductase] -- rn:R10028 <-- N,N-Dihydroxy-L-isoleucine --> rn:R10029

rn:R03615 { Flavonoid } <-- [Reduced NADPH---hemoprotein reductase] -- rn:R10028 <-- N,N-Dihydroxy-L-isoleucine --> rn:R10029

rn:R06560 { Formononetin } <-- [Reduced NADPH---hemoprotein reductase] -- rn:R10028 <-- N,N-Dihydroxy-L-isoleucine --> rn:R10029

rn:R07198 { Liquiritigenin } <-- [Reduced NADPH---hemoprotein reductase] -- rn:R10028 <-- N,N-Dihydroxy-L-isoleucine --> rn:R10029

rn:R07745 { Calycosin } <-- [Reduced NADPH---hemoprotein reductase] -- rn:R10028 <-- N,N-Dihydroxy-L-isoleucine --> rn:R10029

rn:R07777 { Liquiritigenin } <-- [Reduced NADPH---hemoprotein reductase] -- rn:R10028 <-- N,N-Dihydroxy-L-isoleucine --> rn:R10029

rn:R08002 { Liquiritigenin } <-- [Reduced NADPH---hemoprotein reductase] -- rn:R10028 <-- N,N-Dihydroxy-L-isoleucine --> rn:R10029

rn:R03006 { Formononetin } <-- [Reduced NADPH---hemoprotein reductase] -- rn:R10031 <-- [Oxidized NADPH---hemoprotein reductase] --> rn:R08551

rn:R03615 { Flavonoid } <-- [Reduced NADPH---hemoprotein reductase] -- rn:R10031 <-- [Oxidized NADPH---hemoprotein reductase] --> rn:R08551

rn:R06560 { Formononetin } <-- [Reduced NADPH---hemoprotein reductase] -- rn:R10031 <-- [Oxidized NADPH---hemoprotein reductase] --> rn:R08551

rn:R07198 { Liquiritigenin } <-- [Reduced NADPH---hemoprotein reductase] -- rn:R10031 <-- [Oxidized NADPH---hemoprotein reductase] --> rn:R08551

rn:R07745 { Calycosin } <-- [Reduced NADPH---hemoprotein reductase] -- rn:R10031 <-- [Oxidized NADPH---hemoprotein reductase] --> rn:R08551

rn:R07777 { Liquiritigenin } <-- [Reduced NADPH---hemoprotein reductase] -- rn:R10031 <-- [Oxidized NADPH---hemoprotein reductase] --> rn:R08551

rn:R08002 { Liquiritigenin } <-- [Reduced NADPH---hemoprotein reductase] -- rn:R10031 <-- [Oxidized NADPH---hemoprotein reductase] --> rn:R08551

rn:R03006 { Formononetin } <-- [Reduced NADPH---hemoprotein reductase] -- rn:R10031 <-- N-Hydroxy-L-valine --> rn:R10032

rn:R03615 { Flavonoid } <-- [Reduced NADPH---hemoprotein reductase] -- rn:R10031 <-- N-Hydroxy-L-valine --> rn:R10032

rn:R06560 { Formononetin } <-- [Reduced NADPH---hemoprotein reductase] -- rn:R10031 <-- N-Hydroxy-L-valine --> rn:R10032

rn:R07198 { Liquiritigenin } <-- [Reduced NADPH---hemoprotein reductase] -- rn:R10031 <-- N-Hydroxy-L-valine --> rn:R10032

rn:R07745 { Calycosin } <-- [Reduced NADPH---hemoprotein reductase] -- rn:R10031 <-- N-Hydroxy-L-valine --> rn:R10032

rn:R07777 { Liquiritigenin } <-- [Reduced NADPH---hemoprotein reductase] -- rn:R10031 <-- N-Hydroxy-L-valine --> rn:R10032

rn:R08002 { Liquiritigenin } <-- [Reduced NADPH---hemoprotein reductase] -- rn:R10031 <-- N-Hydroxy-L-valine --> rn:R10032

rn:R03006 { Formononetin } <-- [Reduced NADPH---hemoprotein reductase] -- rn:R10032 <-- [Oxidized NADPH---hemoprotein reductase] --> rn:R08551

rn:R03615 { Flavonoid } <-- [Reduced NADPH---hemoprotein reductase] -- rn:R10032 <-- [Oxidized NADPH---hemoprotein reductase] --> rn:R08551

rn:R06560 { Formononetin } <-- [Reduced NADPH---hemoprotein reductase] -- rn:R10032 <-- [Oxidized NADPH---hemoprotein reductase] --> rn:R08551

rn:R07198 { Liquiritigenin } <-- [Reduced NADPH---hemoprotein reductase] -- rn:R10032 <-- [Oxidized NADPH---hemoprotein reductase] --> rn:R08551

rn:R07745 { Calycosin } <-- [Reduced NADPH---hemoprotein reductase] -- rn:R10032 <-- [Oxidized NADPH---hemoprotein reductase] --> rn:R08551

rn:R07777 { Liquiritigenin } <-- [Reduced NADPH---hemoprotein reductase] -- rn:R10032 <-- [Oxidized NADPH---hemoprotein reductase] --> rn:R08551

rn:R08002 { Liquiritigenin } <-- [Reduced NADPH---hemoprotein reductase] -- rn:R10032 <-- [Oxidized NADPH---hemoprotein reductase] --> rn:R08551

rn:R03006 { Formononetin } <-- [Reduced NADPH---hemoprotein reductase] -- rn:R10032 <-- N,N-Dihydroxy-L-valine --> rn:R10033

rn:R03615 { Flavonoid } <-- [Reduced NADPH---hemoprotein reductase] -- rn:R10032 <-- N,N-Dihydroxy-L-valine --> rn:R10033

rn:R06560 { Formononetin } <-- [Reduced NADPH---hemoprotein reductase] -- rn:R10032 <-- N,N-Dihydroxy-L-valine --> rn:R10033

rn:R07198 { Liquiritigenin } <-- [Reduced NADPH---hemoprotein reductase] -- rn:R10032 <-- N,N-Dihydroxy-L-valine --> rn:R10033

rn:R07745 { Calycosin } <-- [Reduced NADPH---hemoprotein reductase] -- rn:R10032 <-- N,N-Dihydroxy-L-valine --> rn:R10033

rn:R07777 { Liquiritigenin } <-- [Reduced NADPH---hemoprotein reductase] -- rn:R10032 <-- N,N-Dihydroxy-L-valine --> rn:R10033

rn:R08002 { Liquiritigenin } <-- [Reduced NADPH---hemoprotein reductase] -- rn:R10032 <-- N,N-Dihydroxy-L-valine --> rn:R10033

rn:R03548 { 7,8-Dihydroxycoumarin } <-- UDP-glucose -- rn:R10037 <-- Lotaustralin --> rn:R10039

rn:R07729 { Formononetin } <-- UDP-glucose -- rn:R10037 <-- Lotaustralin --> rn:R10039

rn:R07712 { Liquiritigenin } <-- 2-Oxoglutarate -- rn:R10415 <-- Succinate --> rn:R02164

rn:R07996 { Liquiritigenin } <-- 2-Oxoglutarate -- rn:R10415 <-- Succinate --> rn:R02164

rn:R07712 { Liquiritigenin } <-- 2-Oxoglutarate -- rn:R10415 <-- Succinate --> rn:R02603

rn:R07996 { Liquiritigenin } <-- 2-Oxoglutarate -- rn:R10415 <-- Succinate --> rn:R02603

rn:R07712 { Liquiritigenin } <-- 2-Oxoglutarate -- rn:R10415 <-- (5R)-Carbapen-2-em-3-carboxylate --> rn:R10736

rn:R07996 { Liquiritigenin } <-- 2-Oxoglutarate -- rn:R10415 <-- (5R)-Carbapen-2-em-3-carboxylate --> rn:R10736

rn:R06776 { 3-Amino-4,7-dihydroxy-8-chlorocoumarin } <-- 3-Dimethylallyl-4-hydroxybenzoate -- rn:R10453 <-- AMP --> rn:R01490

rn:R06776 { 3-Amino-4,7-dihydroxy-8-chlorocoumarin } <-- 3-Dimethylallyl-4-hydroxybenzoate -- rn:R10453 <-- AMP --> rn:R05717

rn:R06776 { 3-Amino-4,7-dihydroxy-8-chlorocoumarin } <-- 3-Dimethylallyl-4-hydroxybenzoate -- rn:R10453 <-- AMP --> rn:R08743

rn:R06776 { 3-Amino-4,7-dihydroxy-8-chlorocoumarin } <-- 3-Dimethylallyl-4-hydroxybenzoate -- rn:R10453 <-- 8-Demethylnovobiocic acid --> rn:R10454

rn:R06776 { 3-Amino-4,7-dihydroxy-8-chlorocoumarin } <-- 3-Dimethylallyl-4-hydroxybenzoate -- rn:R10453 <-- AMP --> rn:R11679

rn:R03548 { 7,8-Dihydroxycoumarin } <-- UDP-glucose -- rn:R10638 <-- Prunasin --> rn:R02558

rn:R07729 { Formononetin } <-- UDP-glucose -- rn:R10638 <-- Prunasin --> rn:R02558

rn:R03548 { 7,8-Dihydroxycoumarin } <-- UDP-glucose -- rn:R10638 <-- Prunasin --> rn:R02985

rn:R07729 { Formononetin } <-- UDP-glucose -- rn:R10638 <-- Prunasin --> rn:R02985

rn:R03548 { 7,8-Dihydroxycoumarin } <-- UDP-glucose -- rn:R10638 <-- Prunasin --> rn:R10639

rn:R07729 { Formononetin } <-- UDP-glucose -- rn:R10638 <-- Prunasin --> rn:R10639

rn:R03006 { Formononetin } <-- [Reduced NADPH---hemoprotein reductase] -- rn:R10671 <-- (E)-4-Hydroxyphenylacetaldehyde oxime --> rn:R05728

rn:R03615 { Flavonoid } <-- [Reduced NADPH---hemoprotein reductase] -- rn:R10671 <-- (E)-4-Hydroxyphenylacetaldehyde oxime --> rn:R05728

rn:R06560 { Formononetin } <-- [Reduced NADPH---hemoprotein reductase] -- rn:R10671 <-- (E)-4-Hydroxyphenylacetaldehyde oxime --> rn:R05728

rn:R07198 { Liquiritigenin } <-- [Reduced NADPH---hemoprotein reductase] -- rn:R10671 <-- (E)-4-Hydroxyphenylacetaldehyde oxime --> rn:R05728

rn:R07745 { Calycosin } <-- [Reduced NADPH---hemoprotein reductase] -- rn:R10671 <-- (E)-4-Hydroxyphenylacetaldehyde oxime --> rn:R05728

rn:R07777 { Liquiritigenin } <-- [Reduced NADPH---hemoprotein reductase] -- rn:R10671 <-- (E)-4-Hydroxyphenylacetaldehyde oxime --> rn:R05728

rn:R08002 { Liquiritigenin } <-- [Reduced NADPH---hemoprotein reductase] -- rn:R10671 <-- (E)-4-Hydroxyphenylacetaldehyde oxime --> rn:R05728

rn:R03006 { Formononetin } <-- [Reduced NADPH---hemoprotein reductase] -- rn:R10671 <-- [Oxidized NADPH---hemoprotein reductase] --> rn:R08551

rn:R03615 { Flavonoid } <-- [Reduced NADPH---hemoprotein reductase] -- rn:R10671 <-- [Oxidized NADPH---hemoprotein reductase] --> rn:R08551

rn:R06560 { Formononetin } <-- [Reduced NADPH---hemoprotein reductase] -- rn:R10671 <-- [Oxidized NADPH---hemoprotein reductase] --> rn:R08551

rn:R07198 { Liquiritigenin } <-- [Reduced NADPH---hemoprotein reductase] -- rn:R10671 <-- [Oxidized NADPH---hemoprotein reductase] --> rn:R08551

rn:R07745 { Calycosin } <-- [Reduced NADPH---hemoprotein reductase] -- rn:R10671 <-- [Oxidized NADPH---hemoprotein reductase] --> rn:R08551

rn:R07777 { Liquiritigenin } <-- [Reduced NADPH---hemoprotein reductase] -- rn:R10671 <-- [Oxidized NADPH---hemoprotein reductase] --> rn:R08551

rn:R08002 { Liquiritigenin } <-- [Reduced NADPH---hemoprotein reductase] -- rn:R10671 <-- [Oxidized NADPH---hemoprotein reductase] --> rn:R08551

rn:R03006 { Formononetin } <-- [Reduced NADPH---hemoprotein reductase] -- rn:R10671 <-- (E)-4-Hydroxyphenylacetaldehyde oxime --> rn:R10672

rn:R03615 { Flavonoid } <-- [Reduced NADPH---hemoprotein reductase] -- rn:R10671 <-- (E)-4-Hydroxyphenylacetaldehyde oxime --> rn:R10672

rn:R06560 { Formononetin } <-- [Reduced NADPH---hemoprotein reductase] -- rn:R10671 <-- (E)-4-Hydroxyphenylacetaldehyde oxime --> rn:R10672

rn:R07198 { Liquiritigenin } <-- [Reduced NADPH---hemoprotein reductase] -- rn:R10671 <-- (E)-4-Hydroxyphenylacetaldehyde oxime --> rn:R10672

rn:R07745 { Calycosin } <-- [Reduced NADPH---hemoprotein reductase] -- rn:R10671 <-- (E)-4-Hydroxyphenylacetaldehyde oxime --> rn:R10672

rn:R07777 { Liquiritigenin } <-- [Reduced NADPH---hemoprotein reductase] -- rn:R10671 <-- (E)-4-Hydroxyphenylacetaldehyde oxime --> rn:R10672

rn:R08002 { Liquiritigenin } <-- [Reduced NADPH---hemoprotein reductase] -- rn:R10671 <-- (E)-4-Hydroxyphenylacetaldehyde oxime --> rn:R10672

rn:R03006 { Formononetin } <-- [Reduced NADPH---hemoprotein reductase] -- rn:R10671 <-- (E)-4-Hydroxyphenylacetaldehyde oxime --> rn:R11442

rn:R03615 { Flavonoid } <-- [Reduced NADPH---hemoprotein reductase] -- rn:R10671 <-- (E)-4-Hydroxyphenylacetaldehyde oxime --> rn:R11442

rn:R06560 { Formononetin } <-- [Reduced NADPH---hemoprotein reductase] -- rn:R10671 <-- (E)-4-Hydroxyphenylacetaldehyde oxime --> rn:R11442

rn:R07198 { Liquiritigenin } <-- [Reduced NADPH---hemoprotein reductase] -- rn:R10671 <-- (E)-4-Hydroxyphenylacetaldehyde oxime --> rn:R11442

rn:R07745 { Calycosin } <-- [Reduced NADPH---hemoprotein reductase] -- rn:R10671 <-- (E)-4-Hydroxyphenylacetaldehyde oxime --> rn:R11442

rn:R07777 { Liquiritigenin } <-- [Reduced NADPH---hemoprotein reductase] -- rn:R10671 <-- (E)-4-Hydroxyphenylacetaldehyde oxime --> rn:R11442

rn:R08002 { Liquiritigenin } <-- [Reduced NADPH---hemoprotein reductase] -- rn:R10671 <-- (E)-4-Hydroxyphenylacetaldehyde oxime --> rn:R11442

rn:R07712 { Liquiritigenin } <-- 2-Oxoglutarate -- rn:R10724 <-- Succinate --> rn:R02164

rn:R07996 { Liquiritigenin } <-- 2-Oxoglutarate -- rn:R10724 <-- Succinate --> rn:R02164

rn:R07712 { Liquiritigenin } <-- 2-Oxoglutarate -- rn:R10724 <-- Succinate --> rn:R02603

rn:R07996 { Liquiritigenin } <-- 2-Oxoglutarate -- rn:R10724 <-- Succinate --> rn:R02603

rn:R07712 { Liquiritigenin } <-- 2-Oxoglutarate -- rn:R10724 <-- (2-Amino-1-hydroxyethyl)phosphonate --> rn:R04924

rn:R07996 { Liquiritigenin } <-- 2-Oxoglutarate -- rn:R10724 <-- (2-Amino-1-hydroxyethyl)phosphonate --> rn:R04924

rn:R07712 { Liquiritigenin } <-- 2-Oxoglutarate -- rn:R10724 <-- (2-Amino-1-hydroxyethyl)phosphonate --> rn:R10722

rn:R07996 { Liquiritigenin } <-- 2-Oxoglutarate -- rn:R10724 <-- (2-Amino-1-hydroxyethyl)phosphonate --> rn:R10722

rn:R03006 { Formononetin } <-- [Reduced NADPH---hemoprotein reductase] -- rn:R10728 <-- [Oxidized NADPH---hemoprotein reductase] --> rn:R08551

rn:R03615 { Flavonoid } <-- [Reduced NADPH---hemoprotein reductase] -- rn:R10728 <-- [Oxidized NADPH---hemoprotein reductase] --> rn:R08551

rn:R06560 { Formononetin } <-- [Reduced NADPH---hemoprotein reductase] -- rn:R10728 <-- [Oxidized NADPH---hemoprotein reductase] --> rn:R08551

rn:R07198 { Liquiritigenin } <-- [Reduced NADPH---hemoprotein reductase] -- rn:R10728 <-- [Oxidized NADPH---hemoprotein reductase] --> rn:R08551

rn:R07745 { Calycosin } <-- [Reduced NADPH---hemoprotein reductase] -- rn:R10728 <-- [Oxidized NADPH---hemoprotein reductase] --> rn:R08551

rn:R07777 { Liquiritigenin } <-- [Reduced NADPH---hemoprotein reductase] -- rn:R10728 <-- [Oxidized NADPH---hemoprotein reductase] --> rn:R08551

rn:R08002 { Liquiritigenin } <-- [Reduced NADPH---hemoprotein reductase] -- rn:R10728 <-- [Oxidized NADPH---hemoprotein reductase] --> rn:R08551

rn:R07712 { Liquiritigenin } <-- 2-Oxoglutarate -- rn:R10735 <-- Succinate --> rn:R02164

rn:R07996 { Liquiritigenin } <-- 2-Oxoglutarate -- rn:R10735 <-- Succinate --> rn:R02164

rn:R07712 { Liquiritigenin } <-- 2-Oxoglutarate -- rn:R10735 <-- Succinate --> rn:R02603

rn:R07996 { Liquiritigenin } <-- 2-Oxoglutarate -- rn:R10735 <-- Succinate --> rn:R02603

rn:R07712 { Liquiritigenin } <-- 2-Oxoglutarate -- rn:R10735 <-- OA-6129 B2 --> rn:R10750

rn:R07996 { Liquiritigenin } <-- 2-Oxoglutarate -- rn:R10735 <-- OA-6129 B2 --> rn:R10750

rn:R07712 { Liquiritigenin } <-- 2-Oxoglutarate -- rn:R10740 <-- Succinate --> rn:R02164

rn:R07996 { Liquiritigenin } <-- 2-Oxoglutarate -- rn:R10740 <-- Succinate --> rn:R02164

rn:R07712 { Liquiritigenin } <-- 2-Oxoglutarate -- rn:R10740 <-- Succinate --> rn:R02603

rn:R07996 { Liquiritigenin } <-- 2-Oxoglutarate -- rn:R10740 <-- Succinate --> rn:R02603

rn:R07712 { Liquiritigenin } <-- 2-Oxoglutarate -- rn:R10740 <-- Thienamycin --> rn:R10745

rn:R07996 { Liquiritigenin } <-- 2-Oxoglutarate -- rn:R10740 <-- Thienamycin --> rn:R10745

rn:R07712 { Liquiritigenin } <-- 2-Oxoglutarate -- rn:R10741 <-- Succinate --> rn:R02164

rn:R07996 { Liquiritigenin } <-- 2-Oxoglutarate -- rn:R10741 <-- Succinate --> rn:R02164

rn:R07712 { Liquiritigenin } <-- 2-Oxoglutarate -- rn:R10741 <-- Succinate --> rn:R02603

rn:R07996 { Liquiritigenin } <-- 2-Oxoglutarate -- rn:R10741 <-- Succinate --> rn:R02603

rn:R07712 { Liquiritigenin } <-- 2-Oxoglutarate -- rn:R10741 <-- Epithienamycin E --> rn:R10742

rn:R07996 { Liquiritigenin } <-- 2-Oxoglutarate -- rn:R10741 <-- Epithienamycin E --> rn:R10742

rn:R07712 { Liquiritigenin } <-- 2-Oxoglutarate -- rn:R10742 <-- Succinate --> rn:R02164

rn:R07996 { Liquiritigenin } <-- 2-Oxoglutarate -- rn:R10742 <-- Succinate --> rn:R02164

rn:R07712 { Liquiritigenin } <-- 2-Oxoglutarate -- rn:R10742 <-- Succinate --> rn:R02603

rn:R07996 { Liquiritigenin } <-- 2-Oxoglutarate -- rn:R10742 <-- Succinate --> rn:R02603

rn:R03006 { Formononetin } <-- [Reduced NADPH---hemoprotein reductase] -- rn:R10795 <-- [Oxidized NADPH---hemoprotein reductase] --> rn:R08551

rn:R03615 { Flavonoid } <-- [Reduced NADPH---hemoprotein reductase] -- rn:R10795 <-- [Oxidized NADPH---hemoprotein reductase] --> rn:R08551

rn:R06560 { Formononetin } <-- [Reduced NADPH---hemoprotein reductase] -- rn:R10795 <-- [Oxidized NADPH---hemoprotein reductase] --> rn:R08551

rn:R07198 { Liquiritigenin } <-- [Reduced NADPH---hemoprotein reductase] -- rn:R10795 <-- [Oxidized NADPH---hemoprotein reductase] --> rn:R08551

rn:R07745 { Calycosin } <-- [Reduced NADPH---hemoprotein reductase] -- rn:R10795 <-- [Oxidized NADPH---hemoprotein reductase] --> rn:R08551

rn:R07777 { Liquiritigenin } <-- [Reduced NADPH---hemoprotein reductase] -- rn:R10795 <-- [Oxidized NADPH---hemoprotein reductase] --> rn:R08551

rn:R08002 { Liquiritigenin } <-- [Reduced NADPH---hemoprotein reductase] -- rn:R10795 <-- [Oxidized NADPH---hemoprotein reductase] --> rn:R08551

rn:R03006 { Formononetin } <-- [Reduced NADPH---hemoprotein reductase] -- rn:R10999 <-- [Oxidized NADPH---hemoprotein reductase] --> rn:R08551

rn:R03615 { Flavonoid } <-- [Reduced NADPH---hemoprotein reductase] -- rn:R10999 <-- [Oxidized NADPH---hemoprotein reductase] --> rn:R08551

rn:R06560 { Formononetin } <-- [Reduced NADPH---hemoprotein reductase] -- rn:R10999 <-- [Oxidized NADPH---hemoprotein reductase] --> rn:R08551

rn:R07198 { Liquiritigenin } <-- [Reduced NADPH---hemoprotein reductase] -- rn:R10999 <-- [Oxidized NADPH---hemoprotein reductase] --> rn:R08551

rn:R07745 { Calycosin } <-- [Reduced NADPH---hemoprotein reductase] -- rn:R10999 <-- [Oxidized NADPH---hemoprotein reductase] --> rn:R08551

rn:R07777 { Liquiritigenin } <-- [Reduced NADPH---hemoprotein reductase] -- rn:R10999 <-- [Oxidized NADPH---hemoprotein reductase] --> rn:R08551

rn:R08002 { Liquiritigenin } <-- [Reduced NADPH---hemoprotein reductase] -- rn:R10999 <-- [Oxidized NADPH---hemoprotein reductase] --> rn:R08551

rn:R03006 { Formononetin } <-- [Reduced NADPH---hemoprotein reductase] -- rn:R11000 <-- [Oxidized NADPH---hemoprotein reductase] --> rn:R08551

rn:R03615 { Flavonoid } <-- [Reduced NADPH---hemoprotein reductase] -- rn:R11000 <-- [Oxidized NADPH---hemoprotein reductase] --> rn:R08551

rn:R06560 { Formononetin } <-- [Reduced NADPH---hemoprotein reductase] -- rn:R11000 <-- [Oxidized NADPH---hemoprotein reductase] --> rn:R08551

rn:R07198 { Liquiritigenin } <-- [Reduced NADPH---hemoprotein reductase] -- rn:R11000 <-- [Oxidized NADPH---hemoprotein reductase] --> rn:R08551

rn:R07745 { Calycosin } <-- [Reduced NADPH---hemoprotein reductase] -- rn:R11000 <-- [Oxidized NADPH---hemoprotein reductase] --> rn:R08551

rn:R07777 { Liquiritigenin } <-- [Reduced NADPH---hemoprotein reductase] -- rn:R11000 <-- [Oxidized NADPH---hemoprotein reductase] --> rn:R08551

rn:R08002 { Liquiritigenin } <-- [Reduced NADPH---hemoprotein reductase] -- rn:R11000 <-- [Oxidized NADPH---hemoprotein reductase] --> rn:R08551

rn:R07712 { Liquiritigenin } <-- 2-Oxoglutarate -- rn:R11340 <-- Succinate --> rn:R02164

rn:R07996 { Liquiritigenin } <-- 2-Oxoglutarate -- rn:R11340 <-- Succinate --> rn:R02164

rn:R07712 { Liquiritigenin } <-- 2-Oxoglutarate -- rn:R11340 <-- Succinate --> rn:R02603

rn:R07996 { Liquiritigenin } <-- 2-Oxoglutarate -- rn:R11340 <-- Succinate --> rn:R02603

rn:R03006 { Formononetin } <-- [Reduced NADPH---hemoprotein reductase] -- rn:R11597 <-- [Oxidized NADPH---hemoprotein reductase] --> rn:R08551

rn:R03615 { Flavonoid } <-- [Reduced NADPH---hemoprotein reductase] -- rn:R11597 <-- [Oxidized NADPH---hemoprotein reductase] --> rn:R08551

rn:R06560 { Formononetin } <-- [Reduced NADPH---hemoprotein reductase] -- rn:R11597 <-- [Oxidized NADPH---hemoprotein reductase] --> rn:R08551

rn:R07198 { Liquiritigenin } <-- [Reduced NADPH---hemoprotein reductase] -- rn:R11597 <-- [Oxidized NADPH---hemoprotein reductase] --> rn:R08551

rn:R07745 { Calycosin } <-- [Reduced NADPH---hemoprotein reductase] -- rn:R11597 <-- [Oxidized NADPH---hemoprotein reductase] --> rn:R08551

rn:R07777 { Liquiritigenin } <-- [Reduced NADPH---hemoprotein reductase] -- rn:R11597 <-- [Oxidized NADPH---hemoprotein reductase] --> rn:R08551

rn:R08002 { Liquiritigenin } <-- [Reduced NADPH---hemoprotein reductase] -- rn:R11597 <-- [Oxidized NADPH---hemoprotein reductase] --> rn:R08551

rn:R03006 { Formononetin } <-- [Reduced NADPH---hemoprotein reductase] -- rn:R11597 <-- (2R)-2-Hydroxy-2-methylbutanenitrile --> rn:R09358

rn:R03615 { Flavonoid } <-- [Reduced NADPH---hemoprotein reductase] -- rn:R11597 <-- (2R)-2-Hydroxy-2-methylbutanenitrile --> rn:R09358

rn:R06560 { Formononetin } <-- [Reduced NADPH---hemoprotein reductase] -- rn:R11597 <-- (2R)-2-Hydroxy-2-methylbutanenitrile --> rn:R09358

rn:R07198 { Liquiritigenin } <-- [Reduced NADPH---hemoprotein reductase] -- rn:R11597 <-- (2R)-2-Hydroxy-2-methylbutanenitrile --> rn:R09358

rn:R07745 { Calycosin } <-- [Reduced NADPH---hemoprotein reductase] -- rn:R11597 <-- (2R)-2-Hydroxy-2-methylbutanenitrile --> rn:R09358

rn:R07777 { Liquiritigenin } <-- [Reduced NADPH---hemoprotein reductase] -- rn:R11597 <-- (2R)-2-Hydroxy-2-methylbutanenitrile --> rn:R09358

rn:R08002 { Liquiritigenin } <-- [Reduced NADPH---hemoprotein reductase] -- rn:R11597 <-- (2R)-2-Hydroxy-2-methylbutanenitrile --> rn:R09358

rn:R03006 { Formononetin } <-- [Reduced NADPH---hemoprotein reductase] -- rn:R11597 <-- (2R)-2-Hydroxy-2-methylbutanenitrile --> rn:R10037

rn:R03615 { Flavonoid } <-- [Reduced NADPH---hemoprotein reductase] -- rn:R11597 <-- (2R)-2-Hydroxy-2-methylbutanenitrile --> rn:R10037

rn:R06560 { Formononetin } <-- [Reduced NADPH---hemoprotein reductase] -- rn:R11597 <-- (2R)-2-Hydroxy-2-methylbutanenitrile --> rn:R10037

rn:R07198 { Liquiritigenin } <-- [Reduced NADPH---hemoprotein reductase] -- rn:R11597 <-- (2R)-2-Hydroxy-2-methylbutanenitrile --> rn:R10037

rn:R07745 { Calycosin } <-- [Reduced NADPH---hemoprotein reductase] -- rn:R11597 <-- (2R)-2-Hydroxy-2-methylbutanenitrile --> rn:R10037

rn:R07777 { Liquiritigenin } <-- [Reduced NADPH---hemoprotein reductase] -- rn:R11597 <-- (2R)-2-Hydroxy-2-methylbutanenitrile --> rn:R10037

rn:R08002 { Liquiritigenin } <-- [Reduced NADPH---hemoprotein reductase] -- rn:R11597 <-- (2R)-2-Hydroxy-2-methylbutanenitrile --> rn:R10037

rn:R03006 { Formononetin } <-- [Reduced NADPH---hemoprotein reductase] -- rn:R11598 <-- Acetone cyanohydrin --> rn:R01553

rn:R03615 { Flavonoid } <-- [Reduced NADPH---hemoprotein reductase] -- rn:R11598 <-- Acetone cyanohydrin --> rn:R01553

rn:R06560 { Formononetin } <-- [Reduced NADPH---hemoprotein reductase] -- rn:R11598 <-- Acetone cyanohydrin --> rn:R01553

rn:R07198 { Liquiritigenin } <-- [Reduced NADPH---hemoprotein reductase] -- rn:R11598 <-- Acetone cyanohydrin --> rn:R01553

rn:R07745 { Calycosin } <-- [Reduced NADPH---hemoprotein reductase] -- rn:R11598 <-- Acetone cyanohydrin --> rn:R01553

rn:R07777 { Liquiritigenin } <-- [Reduced NADPH---hemoprotein reductase] -- rn:R11598 <-- Acetone cyanohydrin --> rn:R01553

rn:R08002 { Liquiritigenin } <-- [Reduced NADPH---hemoprotein reductase] -- rn:R11598 <-- Acetone cyanohydrin --> rn:R01553

rn:R03006 { Formononetin } <-- [Reduced NADPH---hemoprotein reductase] -- rn:R11598 <-- Acetone cyanohydrin --> rn:R03625

rn:R03615 { Flavonoid } <-- [Reduced NADPH---hemoprotein reductase] -- rn:R11598 <-- Acetone cyanohydrin --> rn:R03625

rn:R06560 { Formononetin } <-- [Reduced NADPH---hemoprotein reductase] -- rn:R11598 <-- Acetone cyanohydrin --> rn:R03625

rn:R07198 { Liquiritigenin } <-- [Reduced NADPH---hemoprotein reductase] -- rn:R11598 <-- Acetone cyanohydrin --> rn:R03625

rn:R07745 { Calycosin } <-- [Reduced NADPH---hemoprotein reductase] -- rn:R11598 <-- Acetone cyanohydrin --> rn:R03625

rn:R07777 { Liquiritigenin } <-- [Reduced NADPH---hemoprotein reductase] -- rn:R11598 <-- Acetone cyanohydrin --> rn:R03625

rn:R08002 { Liquiritigenin } <-- [Reduced NADPH---hemoprotein reductase] -- rn:R11598 <-- Acetone cyanohydrin --> rn:R03625

rn:R03006 { Formononetin } <-- [Reduced NADPH---hemoprotein reductase] -- rn:R11598 <-- [Oxidized NADPH---hemoprotein reductase] --> rn:R08551

rn:R03615 { Flavonoid } <-- [Reduced NADPH---hemoprotein reductase] -- rn:R11598 <-- [Oxidized NADPH---hemoprotein reductase] --> rn:R08551

rn:R06560 { Formononetin } <-- [Reduced NADPH---hemoprotein reductase] -- rn:R11598 <-- [Oxidized NADPH---hemoprotein reductase] --> rn:R08551

rn:R07198 { Liquiritigenin } <-- [Reduced NADPH---hemoprotein reductase] -- rn:R11598 <-- [Oxidized NADPH---hemoprotein reductase] --> rn:R08551

rn:R07745 { Calycosin } <-- [Reduced NADPH---hemoprotein reductase] -- rn:R11598 <-- [Oxidized NADPH---hemoprotein reductase] --> rn:R08551

rn:R07777 { Liquiritigenin } <-- [Reduced NADPH---hemoprotein reductase] -- rn:R11598 <-- [Oxidized NADPH---hemoprotein reductase] --> rn:R08551

rn:R08002 { Liquiritigenin } <-- [Reduced NADPH---hemoprotein reductase] -- rn:R11598 <-- [Oxidized NADPH---hemoprotein reductase] --> rn:R08551

rn:R03006 { Formononetin } <-- [Reduced NADPH---hemoprotein reductase] -- rn:R11640 <-- [Oxidized NADPH---hemoprotein reductase] --> rn:R08551

rn:R03615 { Flavonoid } <-- [Reduced NADPH---hemoprotein reductase] -- rn:R11640 <-- [Oxidized NADPH---hemoprotein reductase] --> rn:R08551

rn:R06560 { Formononetin } <-- [Reduced NADPH---hemoprotein reductase] -- rn:R11640 <-- [Oxidized NADPH---hemoprotein reductase] --> rn:R08551

rn:R07198 { Liquiritigenin } <-- [Reduced NADPH---hemoprotein reductase] -- rn:R11640 <-- [Oxidized NADPH---hemoprotein reductase] --> rn:R08551

rn:R07745 { Calycosin } <-- [Reduced NADPH---hemoprotein reductase] -- rn:R11640 <-- [Oxidized NADPH---hemoprotein reductase] --> rn:R08551

rn:R07777 { Liquiritigenin } <-- [Reduced NADPH---hemoprotein reductase] -- rn:R11640 <-- [Oxidized NADPH---hemoprotein reductase] --> rn:R08551

rn:R08002 { Liquiritigenin } <-- [Reduced NADPH---hemoprotein reductase] -- rn:R11640 <-- [Oxidized NADPH---hemoprotein reductase] --> rn:R08551

rn:R03006 { Formononetin } <-- [Reduced NADPH---hemoprotein reductase] -- rn:R11640 <-- (2R)-2-Hydroxy-2-methylbutanenitrile --> rn:R09358

rn:R03615 { Flavonoid } <-- [Reduced NADPH---hemoprotein reductase] -- rn:R11640 <-- (2R)-2-Hydroxy-2-methylbutanenitrile --> rn:R09358

rn:R06560 { Formononetin } <-- [Reduced NADPH---hemoprotein reductase] -- rn:R11640 <-- (2R)-2-Hydroxy-2-methylbutanenitrile --> rn:R09358

rn:R07198 { Liquiritigenin } <-- [Reduced NADPH---hemoprotein reductase] -- rn:R11640 <-- (2R)-2-Hydroxy-2-methylbutanenitrile --> rn:R09358

rn:R07745 { Calycosin } <-- [Reduced NADPH---hemoprotein reductase] -- rn:R11640 <-- (2R)-2-Hydroxy-2-methylbutanenitrile --> rn:R09358

rn:R07777 { Liquiritigenin } <-- [Reduced NADPH---hemoprotein reductase] -- rn:R11640 <-- (2R)-2-Hydroxy-2-methylbutanenitrile --> rn:R09358

rn:R08002 { Liquiritigenin } <-- [Reduced NADPH---hemoprotein reductase] -- rn:R11640 <-- (2R)-2-Hydroxy-2-methylbutanenitrile --> rn:R09358

rn:R03006 { Formononetin } <-- [Reduced NADPH---hemoprotein reductase] -- rn:R11640 <-- (2R)-2-Hydroxy-2-methylbutanenitrile --> rn:R10037

rn:R03615 { Flavonoid } <-- [Reduced NADPH---hemoprotein reductase] -- rn:R11640 <-- (2R)-2-Hydroxy-2-methylbutanenitrile --> rn:R10037

rn:R06560 { Formononetin } <-- [Reduced NADPH---hemoprotein reductase] -- rn:R11640 <-- (2R)-2-Hydroxy-2-methylbutanenitrile --> rn:R10037

rn:R07198 { Liquiritigenin } <-- [Reduced NADPH---hemoprotein reductase] -- rn:R11640 <-- (2R)-2-Hydroxy-2-methylbutanenitrile --> rn:R10037

rn:R07745 { Calycosin } <-- [Reduced NADPH---hemoprotein reductase] -- rn:R11640 <-- (2R)-2-Hydroxy-2-methylbutanenitrile --> rn:R10037

rn:R07777 { Liquiritigenin } <-- [Reduced NADPH---hemoprotein reductase] -- rn:R11640 <-- (2R)-2-Hydroxy-2-methylbutanenitrile --> rn:R10037

rn:R08002 { Liquiritigenin } <-- [Reduced NADPH---hemoprotein reductase] -- rn:R11640 <-- (2R)-2-Hydroxy-2-methylbutanenitrile --> rn:R10037

rn:R03006 { Formononetin } <-- [Reduced NADPH---hemoprotein reductase] -- rn:R11642 <-- Acetone cyanohydrin --> rn:R01553

rn:R03615 { Flavonoid } <-- [Reduced NADPH---hemoprotein reductase] -- rn:R11642 <-- Acetone cyanohydrin --> rn:R01553

rn:R06560 { Formononetin } <-- [Reduced NADPH---hemoprotein reductase] -- rn:R11642 <-- Acetone cyanohydrin --> rn:R01553

rn:R07198 { Liquiritigenin } <-- [Reduced NADPH---hemoprotein reductase] -- rn:R11642 <-- Acetone cyanohydrin --> rn:R01553

rn:R07745 { Calycosin } <-- [Reduced NADPH---hemoprotein reductase] -- rn:R11642 <-- Acetone cyanohydrin --> rn:R01553

rn:R07777 { Liquiritigenin } <-- [Reduced NADPH---hemoprotein reductase] -- rn:R11642 <-- Acetone cyanohydrin --> rn:R01553

rn:R08002 { Liquiritigenin } <-- [Reduced NADPH---hemoprotein reductase] -- rn:R11642 <-- Acetone cyanohydrin --> rn:R01553

rn:R03006 { Formononetin } <-- [Reduced NADPH---hemoprotein reductase] -- rn:R11642 <-- Acetone cyanohydrin --> rn:R03625

rn:R03615 { Flavonoid } <-- [Reduced NADPH---hemoprotein reductase] -- rn:R11642 <-- Acetone cyanohydrin --> rn:R03625

rn:R06560 { Formononetin } <-- [Reduced NADPH---hemoprotein reductase] -- rn:R11642 <-- Acetone cyanohydrin --> rn:R03625

rn:R07198 { Liquiritigenin } <-- [Reduced NADPH---hemoprotein reductase] -- rn:R11642 <-- Acetone cyanohydrin --> rn:R03625

rn:R07745 { Calycosin } <-- [Reduced NADPH---hemoprotein reductase] -- rn:R11642 <-- Acetone cyanohydrin --> rn:R03625

rn:R07777 { Liquiritigenin } <-- [Reduced NADPH---hemoprotein reductase] -- rn:R11642 <-- Acetone cyanohydrin --> rn:R03625

rn:R08002 { Liquiritigenin } <-- [Reduced NADPH---hemoprotein reductase] -- rn:R11642 <-- Acetone cyanohydrin --> rn:R03625

rn:R03006 { Formononetin } <-- [Reduced NADPH---hemoprotein reductase] -- rn:R11642 <-- [Oxidized NADPH---hemoprotein reductase] --> rn:R08551

rn:R03615 { Flavonoid } <-- [Reduced NADPH---hemoprotein reductase] -- rn:R11642 <-- [Oxidized NADPH---hemoprotein reductase] --> rn:R08551

rn:R06560 { Formononetin } <-- [Reduced NADPH---hemoprotein reductase] -- rn:R11642 <-- [Oxidized NADPH---hemoprotein reductase] --> rn:R08551

rn:R07198 { Liquiritigenin } <-- [Reduced NADPH---hemoprotein reductase] -- rn:R11642 <-- [Oxidized NADPH---hemoprotein reductase] --> rn:R08551

rn:R07745 { Calycosin } <-- [Reduced NADPH---hemoprotein reductase] -- rn:R11642 <-- [Oxidized NADPH---hemoprotein reductase] --> rn:R08551

rn:R07777 { Liquiritigenin } <-- [Reduced NADPH---hemoprotein reductase] -- rn:R11642 <-- [Oxidized NADPH---hemoprotein reductase] --> rn:R08551

rn:R08002 { Liquiritigenin } <-- [Reduced NADPH---hemoprotein reductase] -- rn:R11642 <-- [Oxidized NADPH---hemoprotein reductase] --> rn:R08551

rn:R03006 { Formononetin } <-- [Reduced NADPH---hemoprotein reductase] -- rn:R11732 <-- Mandelonitrile --> rn:R01767

rn:R03615 { Flavonoid } <-- [Reduced NADPH---hemoprotein reductase] -- rn:R11732 <-- Mandelonitrile --> rn:R01767

rn:R06560 { Formononetin } <-- [Reduced NADPH---hemoprotein reductase] -- rn:R11732 <-- Mandelonitrile --> rn:R01767

rn:R07198 { Liquiritigenin } <-- [Reduced NADPH---hemoprotein reductase] -- rn:R11732 <-- Mandelonitrile --> rn:R01767

rn:R07745 { Calycosin } <-- [Reduced NADPH---hemoprotein reductase] -- rn:R11732 <-- Mandelonitrile --> rn:R01767

rn:R07777 { Liquiritigenin } <-- [Reduced NADPH---hemoprotein reductase] -- rn:R11732 <-- Mandelonitrile --> rn:R01767

rn:R08002 { Liquiritigenin } <-- [Reduced NADPH---hemoprotein reductase] -- rn:R11732 <-- Mandelonitrile --> rn:R01767

rn:R03006 { Formononetin } <-- [Reduced NADPH---hemoprotein reductase] -- rn:R11732 <-- Mandelonitrile --> rn:R03642

rn:R03615 { Flavonoid } <-- [Reduced NADPH---hemoprotein reductase] -- rn:R11732 <-- Mandelonitrile --> rn:R03642

rn:R06560 { Formononetin } <-- [Reduced NADPH---hemoprotein reductase] -- rn:R11732 <-- Mandelonitrile --> rn:R03642

rn:R07198 { Liquiritigenin } <-- [Reduced NADPH---hemoprotein reductase] -- rn:R11732 <-- Mandelonitrile --> rn:R03642

rn:R07745 { Calycosin } <-- [Reduced NADPH---hemoprotein reductase] -- rn:R11732 <-- Mandelonitrile --> rn:R03642

rn:R07777 { Liquiritigenin } <-- [Reduced NADPH---hemoprotein reductase] -- rn:R11732 <-- Mandelonitrile --> rn:R03642

rn:R08002 { Liquiritigenin } <-- [Reduced NADPH---hemoprotein reductase] -- rn:R11732 <-- Mandelonitrile --> rn:R03642

rn:R03006 { Formononetin } <-- [Reduced NADPH---hemoprotein reductase] -- rn:R11732 <-- [Oxidized NADPH---hemoprotein reductase] --> rn:R08551

rn:R03615 { Flavonoid } <-- [Reduced NADPH---hemoprotein reductase] -- rn:R11732 <-- [Oxidized NADPH---hemoprotein reductase] --> rn:R08551

rn:R06560 { Formononetin } <-- [Reduced NADPH---hemoprotein reductase] -- rn:R11732 <-- [Oxidized NADPH---hemoprotein reductase] --> rn:R08551

rn:R07198 { Liquiritigenin } <-- [Reduced NADPH---hemoprotein reductase] -- rn:R11732 <-- [Oxidized NADPH---hemoprotein reductase] --> rn:R08551

rn:R07745 { Calycosin } <-- [Reduced NADPH---hemoprotein reductase] -- rn:R11732 <-- [Oxidized NADPH---hemoprotein reductase] --> rn:R08551

rn:R07777 { Liquiritigenin } <-- [Reduced NADPH---hemoprotein reductase] -- rn:R11732 <-- [Oxidized NADPH---hemoprotein reductase] --> rn:R08551

rn:R08002 { Liquiritigenin } <-- [Reduced NADPH---hemoprotein reductase] -- rn:R11732 <-- [Oxidized NADPH---hemoprotein reductase] --> rn:R08551

rn:R03006 { Formononetin } <-- [Reduced NADPH---hemoprotein reductase] -- rn:R11732 <-- Mandelonitrile --> rn:R10638

rn:R03615 { Flavonoid } <-- [Reduced NADPH---hemoprotein reductase] -- rn:R11732 <-- Mandelonitrile --> rn:R10638

rn:R06560 { Formononetin } <-- [Reduced NADPH---hemoprotein reductase] -- rn:R11732 <-- Mandelonitrile --> rn:R10638

rn:R07198 { Liquiritigenin } <-- [Reduced NADPH---hemoprotein reductase] -- rn:R11732 <-- Mandelonitrile --> rn:R10638

rn:R07745 { Calycosin } <-- [Reduced NADPH---hemoprotein reductase] -- rn:R11732 <-- Mandelonitrile --> rn:R10638

rn:R07777 { Liquiritigenin } <-- [Reduced NADPH---hemoprotein reductase] -- rn:R11732 <-- Mandelonitrile --> rn:R10638

rn:R08002 { Liquiritigenin } <-- [Reduced NADPH---hemoprotein reductase] -- rn:R11732 <-- Mandelonitrile --> rn:R10638

rn:R03006 { Formononetin } <-- [Reduced NADPH---hemoprotein reductase] -- rn:R11732 <-- Mandelonitrile --> rn:R11380

rn:R03615 { Flavonoid } <-- [Reduced NADPH---hemoprotein reductase] -- rn:R11732 <-- Mandelonitrile --> rn:R11380

rn:R06560 { Formononetin } <-- [Reduced NADPH---hemoprotein reductase] -- rn:R11732 <-- Mandelonitrile --> rn:R11380

rn:R07198 { Liquiritigenin } <-- [Reduced NADPH---hemoprotein reductase] -- rn:R11732 <-- Mandelonitrile --> rn:R11380

rn:R07745 { Calycosin } <-- [Reduced NADPH---hemoprotein reductase] -- rn:R11732 <-- Mandelonitrile --> rn:R11380

rn:R07777 { Liquiritigenin } <-- [Reduced NADPH---hemoprotein reductase] -- rn:R11732 <-- Mandelonitrile --> rn:R11380

rn:R08002 { Liquiritigenin } <-- [Reduced NADPH---hemoprotein reductase] -- rn:R11732 <-- Mandelonitrile --> rn:R11380

rn:R03006 { Formononetin } <-- [Reduced NADPH---hemoprotein reductase] -- rn:R11733 <-- Mandelonitrile --> rn:R01767

rn:R03615 { Flavonoid } <-- [Reduced NADPH---hemoprotein reductase] -- rn:R11733 <-- Mandelonitrile --> rn:R01767

rn:R06560 { Formononetin } <-- [Reduced NADPH---hemoprotein reductase] -- rn:R11733 <-- Mandelonitrile --> rn:R01767

rn:R07198 { Liquiritigenin } <-- [Reduced NADPH---hemoprotein reductase] -- rn:R11733 <-- Mandelonitrile --> rn:R01767

rn:R07745 { Calycosin } <-- [Reduced NADPH---hemoprotein reductase] -- rn:R11733 <-- Mandelonitrile --> rn:R01767

rn:R07777 { Liquiritigenin } <-- [Reduced NADPH---hemoprotein reductase] -- rn:R11733 <-- Mandelonitrile --> rn:R01767

rn:R08002 { Liquiritigenin } <-- [Reduced NADPH---hemoprotein reductase] -- rn:R11733 <-- Mandelonitrile --> rn:R01767

rn:R03006 { Formononetin } <-- [Reduced NADPH---hemoprotein reductase] -- rn:R11733 <-- Mandelonitrile --> rn:R03642

rn:R03615 { Flavonoid } <-- [Reduced NADPH---hemoprotein reductase] -- rn:R11733 <-- Mandelonitrile --> rn:R03642

rn:R06560 { Formononetin } <-- [Reduced NADPH---hemoprotein reductase] -- rn:R11733 <-- Mandelonitrile --> rn:R03642

rn:R07198 { Liquiritigenin } <-- [Reduced NADPH---hemoprotein reductase] -- rn:R11733 <-- Mandelonitrile --> rn:R03642

rn:R07745 { Calycosin } <-- [Reduced NADPH---hemoprotein reductase] -- rn:R11733 <-- Mandelonitrile --> rn:R03642

rn:R07777 { Liquiritigenin } <-- [Reduced NADPH---hemoprotein reductase] -- rn:R11733 <-- Mandelonitrile --> rn:R03642

rn:R08002 { Liquiritigenin } <-- [Reduced NADPH---hemoprotein reductase] -- rn:R11733 <-- Mandelonitrile --> rn:R03642

rn:R03006 { Formononetin } <-- [Reduced NADPH---hemoprotein reductase] -- rn:R11733 <-- [Oxidized NADPH---hemoprotein reductase] --> rn:R08551

rn:R03615 { Flavonoid } <-- [Reduced NADPH---hemoprotein reductase] -- rn:R11733 <-- [Oxidized NADPH---hemoprotein reductase] --> rn:R08551

rn:R06560 { Formononetin } <-- [Reduced NADPH---hemoprotein reductase] -- rn:R11733 <-- [Oxidized NADPH---hemoprotein reductase] --> rn:R08551

rn:R07198 { Liquiritigenin } <-- [Reduced NADPH---hemoprotein reductase] -- rn:R11733 <-- [Oxidized NADPH---hemoprotein reductase] --> rn:R08551

rn:R07745 { Calycosin } <-- [Reduced NADPH---hemoprotein reductase] -- rn:R11733 <-- [Oxidized NADPH---hemoprotein reductase] --> rn:R08551

rn:R07777 { Liquiritigenin } <-- [Reduced NADPH---hemoprotein reductase] -- rn:R11733 <-- [Oxidized NADPH---hemoprotein reductase] --> rn:R08551

rn:R08002 { Liquiritigenin } <-- [Reduced NADPH---hemoprotein reductase] -- rn:R11733 <-- [Oxidized NADPH---hemoprotein reductase] --> rn:R08551

rn:R03006 { Formononetin } <-- [Reduced NADPH---hemoprotein reductase] -- rn:R11733 <-- Mandelonitrile --> rn:R10638

rn:R03615 { Flavonoid } <-- [Reduced NADPH---hemoprotein reductase] -- rn:R11733 <-- Mandelonitrile --> rn:R10638

rn:R06560 { Formononetin } <-- [Reduced NADPH---hemoprotein reductase] -- rn:R11733 <-- Mandelonitrile --> rn:R10638

rn:R07198 { Liquiritigenin } <-- [Reduced NADPH---hemoprotein reductase] -- rn:R11733 <-- Mandelonitrile --> rn:R10638

rn:R07745 { Calycosin } <-- [Reduced NADPH---hemoprotein reductase] -- rn:R11733 <-- Mandelonitrile --> rn:R10638

rn:R07777 { Liquiritigenin } <-- [Reduced NADPH---hemoprotein reductase] -- rn:R11733 <-- Mandelonitrile --> rn:R10638

rn:R08002 { Liquiritigenin } <-- [Reduced NADPH---hemoprotein reductase] -- rn:R11733 <-- Mandelonitrile --> rn:R10638

rn:R03006 { Formononetin } <-- [Reduced NADPH---hemoprotein reductase] -- rn:R11733 <-- Mandelonitrile --> rn:R11380

rn:R03615 { Flavonoid } <-- [Reduced NADPH---hemoprotein reductase] -- rn:R11733 <-- Mandelonitrile --> rn:R11380

rn:R06560 { Formononetin } <-- [Reduced NADPH---hemoprotein reductase] -- rn:R11733 <-- Mandelonitrile --> rn:R11380

rn:R07198 { Liquiritigenin } <-- [Reduced NADPH---hemoprotein reductase] -- rn:R11733 <-- Mandelonitrile --> rn:R11380

rn:R07745 { Calycosin } <-- [Reduced NADPH---hemoprotein reductase] -- rn:R11733 <-- Mandelonitrile --> rn:R11380

rn:R07777 { Liquiritigenin } <-- [Reduced NADPH---hemoprotein reductase] -- rn:R11733 <-- Mandelonitrile --> rn:R11380

rn:R08002 { Liquiritigenin } <-- [Reduced NADPH---hemoprotein reductase] -- rn:R11733 <-- Mandelonitrile --> rn:R11380

rn:R03006 { Formononetin } <-- [Reduced NADPH---hemoprotein reductase] -- rn:R11737 <-- [Oxidized NADPH---hemoprotein reductase] --> rn:R08551

rn:R03615 { Flavonoid } <-- [Reduced NADPH---hemoprotein reductase] -- rn:R11737 <-- [Oxidized NADPH---hemoprotein reductase] --> rn:R08551

rn:R06560 { Formononetin } <-- [Reduced NADPH---hemoprotein reductase] -- rn:R11737 <-- [Oxidized NADPH---hemoprotein reductase] --> rn:R08551

rn:R07198 { Liquiritigenin } <-- [Reduced NADPH---hemoprotein reductase] -- rn:R11737 <-- [Oxidized NADPH---hemoprotein reductase] --> rn:R08551

rn:R07745 { Calycosin } <-- [Reduced NADPH---hemoprotein reductase] -- rn:R11737 <-- [Oxidized NADPH---hemoprotein reductase] --> rn:R08551

rn:R07777 { Liquiritigenin } <-- [Reduced NADPH---hemoprotein reductase] -- rn:R11737 <-- [Oxidized NADPH---hemoprotein reductase] --> rn:R08551

rn:R08002 { Liquiritigenin } <-- [Reduced NADPH---hemoprotein reductase] -- rn:R11737 <-- [Oxidized NADPH---hemoprotein reductase] --> rn:R08551

rn:R03006 { Formononetin } <-- [Reduced NADPH---hemoprotein reductase] -- rn:R11738 <-- [Oxidized NADPH---hemoprotein reductase] --> rn:R08551

rn:R03615 { Flavonoid } <-- [Reduced NADPH---hemoprotein reductase] -- rn:R11738 <-- [Oxidized NADPH---hemoprotein reductase] --> rn:R08551

rn:R06560 { Formononetin } <-- [Reduced NADPH---hemoprotein reductase] -- rn:R11738 <-- [Oxidized NADPH---hemoprotein reductase] --> rn:R08551

rn:R07198 { Liquiritigenin } <-- [Reduced NADPH---hemoprotein reductase] -- rn:R11738 <-- [Oxidized NADPH---hemoprotein reductase] --> rn:R08551

rn:R07745 { Calycosin } <-- [Reduced NADPH---hemoprotein reductase] -- rn:R11738 <-- [Oxidized NADPH---hemoprotein reductase] --> rn:R08551

rn:R07777 { Liquiritigenin } <-- [Reduced NADPH---hemoprotein reductase] -- rn:R11738 <-- [Oxidized NADPH---hemoprotein reductase] --> rn:R08551

rn:R08002 { Liquiritigenin } <-- [Reduced NADPH---hemoprotein reductase] -- rn:R11738 <-- [Oxidized NADPH---hemoprotein reductase] --> rn:R08551

rn:R03006 { Formononetin } <-- [Reduced NADPH---hemoprotein reductase] -- rn:R11813 <-- Hydrogen cyanide --> rn:R00152

rn:R03615 { Flavonoid } <-- [Reduced NADPH---hemoprotein reductase] -- rn:R11813 <-- Hydrogen cyanide --> rn:R00152

rn:R06560 { Formononetin } <-- [Reduced NADPH---hemoprotein reductase] -- rn:R11813 <-- Hydrogen cyanide --> rn:R00152

rn:R07198 { Liquiritigenin } <-- [Reduced NADPH---hemoprotein reductase] -- rn:R11813 <-- Hydrogen cyanide --> rn:R00152

rn:R07745 { Calycosin } <-- [Reduced NADPH---hemoprotein reductase] -- rn:R11813 <-- Hydrogen cyanide --> rn:R00152

rn:R07777 { Liquiritigenin } <-- [Reduced NADPH---hemoprotein reductase] -- rn:R11813 <-- Hydrogen cyanide --> rn:R00152

rn:R08002 { Liquiritigenin } <-- [Reduced NADPH---hemoprotein reductase] -- rn:R11813 <-- Hydrogen cyanide --> rn:R00152

rn:R03006 { Formononetin } <-- [Reduced NADPH---hemoprotein reductase] -- rn:R11813 <-- Hydrogen cyanide --> rn:R01410

rn:R03615 { Flavonoid } <-- [Reduced NADPH---hemoprotein reductase] -- rn:R11813 <-- Hydrogen cyanide --> rn:R01410

rn:R06560 { Formononetin } <-- [Reduced NADPH---hemoprotein reductase] -- rn:R11813 <-- Hydrogen cyanide --> rn:R01410

rn:R07198 { Liquiritigenin } <-- [Reduced NADPH---hemoprotein reductase] -- rn:R11813 <-- Hydrogen cyanide --> rn:R01410

rn:R07745 { Calycosin } <-- [Reduced NADPH---hemoprotein reductase] -- rn:R11813 <-- Hydrogen cyanide --> rn:R01410

rn:R07777 { Liquiritigenin } <-- [Reduced NADPH---hemoprotein reductase] -- rn:R11813 <-- Hydrogen cyanide --> rn:R01410

rn:R08002 { Liquiritigenin } <-- [Reduced NADPH---hemoprotein reductase] -- rn:R11813 <-- Hydrogen cyanide --> rn:R01410

rn:R03006 { Formononetin } <-- [Reduced NADPH---hemoprotein reductase] -- rn:R11813 <-- Hydrogen cyanide --> rn:R01650

rn:R03615 { Flavonoid } <-- [Reduced NADPH---hemoprotein reductase] -- rn:R11813 <-- Hydrogen cyanide --> rn:R01650

rn:R06560 { Formononetin } <-- [Reduced NADPH---hemoprotein reductase] -- rn:R11813 <-- Hydrogen cyanide --> rn:R01650

rn:R07198 { Liquiritigenin } <-- [Reduced NADPH---hemoprotein reductase] -- rn:R11813 <-- Hydrogen cyanide --> rn:R01650

rn:R07745 { Calycosin } <-- [Reduced NADPH---hemoprotein reductase] -- rn:R11813 <-- Hydrogen cyanide --> rn:R01650

rn:R07777 { Liquiritigenin } <-- [Reduced NADPH---hemoprotein reductase] -- rn:R11813 <-- Hydrogen cyanide --> rn:R01650

rn:R08002 { Liquiritigenin } <-- [Reduced NADPH---hemoprotein reductase] -- rn:R11813 <-- Hydrogen cyanide --> rn:R01650

rn:R03006 { Formononetin } <-- [Reduced NADPH---hemoprotein reductase] -- rn:R11813 <-- Hydrogen cyanide --> rn:R03524

rn:R03615 { Flavonoid } <-- [Reduced NADPH---hemoprotein reductase] -- rn:R11813 <-- Hydrogen cyanide --> rn:R03524

rn:R06560 { Formononetin } <-- [Reduced NADPH---hemoprotein reductase] -- rn:R11813 <-- Hydrogen cyanide --> rn:R03524

rn:R07198 { Liquiritigenin } <-- [Reduced NADPH---hemoprotein reductase] -- rn:R11813 <-- Hydrogen cyanide --> rn:R03524

rn:R07745 { Calycosin } <-- [Reduced NADPH---hemoprotein reductase] -- rn:R11813 <-- Hydrogen cyanide --> rn:R03524

rn:R07777 { Liquiritigenin } <-- [Reduced NADPH---hemoprotein reductase] -- rn:R11813 <-- Hydrogen cyanide --> rn:R03524

rn:R08002 { Liquiritigenin } <-- [Reduced NADPH---hemoprotein reductase] -- rn:R11813 <-- Hydrogen cyanide --> rn:R03524

rn:R03006 { Formononetin } <-- [Reduced NADPH---hemoprotein reductase] -- rn:R11813 <-- [Oxidized NADPH---hemoprotein reductase] --> rn:R08551

rn:R03615 { Flavonoid } <-- [Reduced NADPH---hemoprotein reductase] -- rn:R11813 <-- [Oxidized NADPH---hemoprotein reductase] --> rn:R08551

rn:R06560 { Formononetin } <-- [Reduced NADPH---hemoprotein reductase] -- rn:R11813 <-- [Oxidized NADPH---hemoprotein reductase] --> rn:R08551

rn:R07198 { Liquiritigenin } <-- [Reduced NADPH---hemoprotein reductase] -- rn:R11813 <-- [Oxidized NADPH---hemoprotein reductase] --> rn:R08551

rn:R07745 { Calycosin } <-- [Reduced NADPH---hemoprotein reductase] -- rn:R11813 <-- [Oxidized NADPH---hemoprotein reductase] --> rn:R08551

rn:R07777 { Liquiritigenin } <-- [Reduced NADPH---hemoprotein reductase] -- rn:R11813 <-- [Oxidized NADPH---hemoprotein reductase] --> rn:R08551

rn:R08002 { Liquiritigenin } <-- [Reduced NADPH---hemoprotein reductase] -- rn:R11813 <-- [Oxidized NADPH---hemoprotein reductase] --> rn:R08551

rn:R03006 { Formononetin } <-- [Reduced NADPH---hemoprotein reductase] -- rn:R11814 <-- Hydrogen cyanide --> rn:R00152

rn:R03615 { Flavonoid } <-- [Reduced NADPH---hemoprotein reductase] -- rn:R11814 <-- Hydrogen cyanide --> rn:R00152

rn:R06560 { Formononetin } <-- [Reduced NADPH---hemoprotein reductase] -- rn:R11814 <-- Hydrogen cyanide --> rn:R00152

rn:R07198 { Liquiritigenin } <-- [Reduced NADPH---hemoprotein reductase] -- rn:R11814 <-- Hydrogen cyanide --> rn:R00152

rn:R07745 { Calycosin } <-- [Reduced NADPH---hemoprotein reductase] -- rn:R11814 <-- Hydrogen cyanide --> rn:R00152

rn:R07777 { Liquiritigenin } <-- [Reduced NADPH---hemoprotein reductase] -- rn:R11814 <-- Hydrogen cyanide --> rn:R00152

rn:R08002 { Liquiritigenin } <-- [Reduced NADPH---hemoprotein reductase] -- rn:R11814 <-- Hydrogen cyanide --> rn:R00152

rn:R03006 { Formononetin } <-- [Reduced NADPH---hemoprotein reductase] -- rn:R11814 <-- Hydrogen cyanide --> rn:R01410

rn:R03615 { Flavonoid } <-- [Reduced NADPH---hemoprotein reductase] -- rn:R11814 <-- Hydrogen cyanide --> rn:R01410

rn:R06560 { Formononetin } <-- [Reduced NADPH---hemoprotein reductase] -- rn:R11814 <-- Hydrogen cyanide --> rn:R01410

rn:R07198 { Liquiritigenin } <-- [Reduced NADPH---hemoprotein reductase] -- rn:R11814 <-- Hydrogen cyanide --> rn:R01410

rn:R07745 { Calycosin } <-- [Reduced NADPH---hemoprotein reductase] -- rn:R11814 <-- Hydrogen cyanide --> rn:R01410

rn:R07777 { Liquiritigenin } <-- [Reduced NADPH---hemoprotein reductase] -- rn:R11814 <-- Hydrogen cyanide --> rn:R01410

rn:R08002 { Liquiritigenin } <-- [Reduced NADPH---hemoprotein reductase] -- rn:R11814 <-- Hydrogen cyanide --> rn:R01410

rn:R03006 { Formononetin } <-- [Reduced NADPH---hemoprotein reductase] -- rn:R11814 <-- Hydrogen cyanide --> rn:R01650

rn:R03615 { Flavonoid } <-- [Reduced NADPH---hemoprotein reductase] -- rn:R11814 <-- Hydrogen cyanide --> rn:R01650

rn:R06560 { Formononetin } <-- [Reduced NADPH---hemoprotein reductase] -- rn:R11814 <-- Hydrogen cyanide --> rn:R01650

rn:R07198 { Liquiritigenin } <-- [Reduced NADPH---hemoprotein reductase] -- rn:R11814 <-- Hydrogen cyanide --> rn:R01650

rn:R07745 { Calycosin } <-- [Reduced NADPH---hemoprotein reductase] -- rn:R11814 <-- Hydrogen cyanide --> rn:R01650

rn:R07777 { Liquiritigenin } <-- [Reduced NADPH---hemoprotein reductase] -- rn:R11814 <-- Hydrogen cyanide --> rn:R01650

rn:R08002 { Liquiritigenin } <-- [Reduced NADPH---hemoprotein reductase] -- rn:R11814 <-- Hydrogen cyanide --> rn:R01650

rn:R03006 { Formononetin } <-- [Reduced NADPH---hemoprotein reductase] -- rn:R11814 <-- Hydrogen cyanide --> rn:R03524

rn:R03615 { Flavonoid } <-- [Reduced NADPH---hemoprotein reductase] -- rn:R11814 <-- Hydrogen cyanide --> rn:R03524

rn:R06560 { Formononetin } <-- [Reduced NADPH---hemoprotein reductase] -- rn:R11814 <-- Hydrogen cyanide --> rn:R03524

rn:R07198 { Liquiritigenin } <-- [Reduced NADPH---hemoprotein reductase] -- rn:R11814 <-- Hydrogen cyanide --> rn:R03524

rn:R07745 { Calycosin } <-- [Reduced NADPH---hemoprotein reductase] -- rn:R11814 <-- Hydrogen cyanide --> rn:R03524

rn:R07777 { Liquiritigenin } <-- [Reduced NADPH---hemoprotein reductase] -- rn:R11814 <-- Hydrogen cyanide --> rn:R03524

rn:R08002 { Liquiritigenin } <-- [Reduced NADPH---hemoprotein reductase] -- rn:R11814 <-- Hydrogen cyanide --> rn:R03524

rn:R03006 { Formononetin } <-- [Reduced NADPH---hemoprotein reductase] -- rn:R11814 <-- [Oxidized NADPH---hemoprotein reductase] --> rn:R08551

rn:R03615 { Flavonoid } <-- [Reduced NADPH---hemoprotein reductase] -- rn:R11814 <-- [Oxidized NADPH---hemoprotein reductase] --> rn:R08551

rn:R06560 { Formononetin } <-- [Reduced NADPH---hemoprotein reductase] -- rn:R11814 <-- [Oxidized NADPH---hemoprotein reductase] --> rn:R08551

rn:R07198 { Liquiritigenin } <-- [Reduced NADPH---hemoprotein reductase] -- rn:R11814 <-- [Oxidized NADPH---hemoprotein reductase] --> rn:R08551

rn:R07745 { Calycosin } <-- [Reduced NADPH---hemoprotein reductase] -- rn:R11814 <-- [Oxidized NADPH---hemoprotein reductase] --> rn:R08551

rn:R07777 { Liquiritigenin } <-- [Reduced NADPH---hemoprotein reductase] -- rn:R11814 <-- [Oxidized NADPH---hemoprotein reductase] --> rn:R08551

rn:R08002 { Liquiritigenin } <-- [Reduced NADPH---hemoprotein reductase] -- rn:R11814 <-- [Oxidized NADPH---hemoprotein reductase] --> rn:R08551

rn:R03006 { Formononetin } <-- [Reduced NADPH---hemoprotein reductase] -- rn:R12072 <-- [Oxidized NADPH---hemoprotein reductase] --> rn:R08551

rn:R03615 { Flavonoid } <-- [Reduced NADPH---hemoprotein reductase] -- rn:R12072 <-- [Oxidized NADPH---hemoprotein reductase] --> rn:R08551

rn:R06560 { Formononetin } <-- [Reduced NADPH---hemoprotein reductase] -- rn:R12072 <-- [Oxidized NADPH---hemoprotein reductase] --> rn:R08551

rn:R07198 { Liquiritigenin } <-- [Reduced NADPH---hemoprotein reductase] -- rn:R12072 <-- [Oxidized NADPH---hemoprotein reductase] --> rn:R08551

rn:R07745 { Calycosin } <-- [Reduced NADPH---hemoprotein reductase] -- rn:R12072 <-- [Oxidized NADPH---hemoprotein reductase] --> rn:R08551

rn:R07777 { Liquiritigenin } <-- [Reduced NADPH---hemoprotein reductase] -- rn:R12072 <-- [Oxidized NADPH---hemoprotein reductase] --> rn:R08551

rn:R08002 { Liquiritigenin } <-- [Reduced NADPH---hemoprotein reductase] -- rn:R12072 <-- [Oxidized NADPH---hemoprotein reductase] --> rn:R08551

rn:R03006 { Formononetin } <-- [Reduced NADPH---hemoprotein reductase] -- rn:R12183 <-- Cobamide coenzyme --> rn:R05223

rn:R03615 { Flavonoid } <-- [Reduced NADPH---hemoprotein reductase] -- rn:R12183 <-- Cobamide coenzyme --> rn:R05223

rn:R06560 { Formononetin } <-- [Reduced NADPH---hemoprotein reductase] -- rn:R12183 <-- Cobamide coenzyme --> rn:R05223

rn:R07198 { Liquiritigenin } <-- [Reduced NADPH---hemoprotein reductase] -- rn:R12183 <-- Cobamide coenzyme --> rn:R05223

rn:R07745 { Calycosin } <-- [Reduced NADPH---hemoprotein reductase] -- rn:R12183 <-- Cobamide coenzyme --> rn:R05223

rn:R07777 { Liquiritigenin } <-- [Reduced NADPH---hemoprotein reductase] -- rn:R12183 <-- Cobamide coenzyme --> rn:R05223

rn:R08002 { Liquiritigenin } <-- [Reduced NADPH---hemoprotein reductase] -- rn:R12183 <-- Cobamide coenzyme --> rn:R05223

rn:R03006 { Formononetin } <-- [Reduced NADPH---hemoprotein reductase] -- rn:R12183 <-- [Oxidized NADPH---hemoprotein reductase] --> rn:R08551

rn:R03615 { Flavonoid } <-- [Reduced NADPH---hemoprotein reductase] -- rn:R12183 <-- [Oxidized NADPH---hemoprotein reductase] --> rn:R08551

rn:R06560 { Formononetin } <-- [Reduced NADPH---hemoprotein reductase] -- rn:R12183 <-- [Oxidized NADPH---hemoprotein reductase] --> rn:R08551

rn:R07198 { Liquiritigenin } <-- [Reduced NADPH---hemoprotein reductase] -- rn:R12183 <-- [Oxidized NADPH---hemoprotein reductase] --> rn:R08551

rn:R07745 { Calycosin } <-- [Reduced NADPH---hemoprotein reductase] -- rn:R12183 <-- [Oxidized NADPH---hemoprotein reductase] --> rn:R08551

rn:R07777 { Liquiritigenin } <-- [Reduced NADPH---hemoprotein reductase] -- rn:R12183 <-- [Oxidized NADPH---hemoprotein reductase] --> rn:R08551

rn:R08002 { Liquiritigenin } <-- [Reduced NADPH---hemoprotein reductase] -- rn:R12183 <-- [Oxidized NADPH---hemoprotein reductase] --> rn:R08551

rn:R03006 { Formononetin } <-- [Reduced NADPH---hemoprotein reductase] -- rn:R12184 <-- Adenosyl cobyrinate a,c diamide --> rn:R05225

rn:R03615 { Flavonoid } <-- [Reduced NADPH---hemoprotein reductase] -- rn:R12184 <-- Adenosyl cobyrinate a,c diamide --> rn:R05225

rn:R06560 { Formononetin } <-- [Reduced NADPH---hemoprotein reductase] -- rn:R12184 <-- Adenosyl cobyrinate a,c diamide --> rn:R05225

rn:R07198 { Liquiritigenin } <-- [Reduced NADPH---hemoprotein reductase] -- rn:R12184 <-- Adenosyl cobyrinate a,c diamide --> rn:R05225

rn:R07745 { Calycosin } <-- [Reduced NADPH---hemoprotein reductase] -- rn:R12184 <-- Adenosyl cobyrinate a,c diamide --> rn:R05225

rn:R07777 { Liquiritigenin } <-- [Reduced NADPH---hemoprotein reductase] -- rn:R12184 <-- Adenosyl cobyrinate a,c diamide --> rn:R05225

rn:R08002 { Liquiritigenin } <-- [Reduced NADPH---hemoprotein reductase] -- rn:R12184 <-- Adenosyl cobyrinate a,c diamide --> rn:R05225

rn:R03006 { Formononetin } <-- [Reduced NADPH---hemoprotein reductase] -- rn:R12184 <-- [Oxidized NADPH---hemoprotein reductase] --> rn:R08551

rn:R03615 { Flavonoid } <-- [Reduced NADPH---hemoprotein reductase] -- rn:R12184 <-- [Oxidized NADPH---hemoprotein reductase] --> rn:R08551

rn:R06560 { Formononetin } <-- [Reduced NADPH---hemoprotein reductase] -- rn:R12184 <-- [Oxidized NADPH---hemoprotein reductase] --> rn:R08551

rn:R07198 { Liquiritigenin } <-- [Reduced NADPH---hemoprotein reductase] -- rn:R12184 <-- [Oxidized NADPH---hemoprotein reductase] --> rn:R08551

rn:R07745 { Calycosin } <-- [Reduced NADPH---hemoprotein reductase] -- rn:R12184 <-- [Oxidized NADPH---hemoprotein reductase] --> rn:R08551

rn:R07777 { Liquiritigenin } <-- [Reduced NADPH---hemoprotein reductase] -- rn:R12184 <-- [Oxidized NADPH---hemoprotein reductase] --> rn:R08551

rn:R08002 { Liquiritigenin } <-- [Reduced NADPH---hemoprotein reductase] -- rn:R12184 <-- [Oxidized NADPH---hemoprotein reductase] --> rn:R08551

rn:R07712 { Liquiritigenin } <-- 2-Oxoglutarate -- rn:R12216 <-- Succinate --> rn:R02164

rn:R07996 { Liquiritigenin } <-- 2-Oxoglutarate -- rn:R12216 <-- Succinate --> rn:R02164

rn:R07712 { Liquiritigenin } <-- 2-Oxoglutarate -- rn:R12216 <-- Succinate --> rn:R02603

rn:R07996 { Liquiritigenin } <-- 2-Oxoglutarate -- rn:R12216 <-- Succinate --> rn:R02603

rn:R07712 { Liquiritigenin } <-- 2-Oxoglutarate -- rn:R12216 <-- (S)-2-Hydroxyglutarate --> rn:R12217

rn:R07996 { Liquiritigenin } <-- 2-Oxoglutarate -- rn:R12216 <-- (S)-2-Hydroxyglutarate --> rn:R12217
