# Supplementary material for: The impact of Traditional Chinese Medicine on mouse gut microbiota abundances and interactions based on Granger causality and pathway analysis
Source: Front Microbiol. 2022 Nov 11;13:980082. doi: 10.3389/fmicb.2022.980082 (PMC9692106; doi:10.3389/fmicb.2022.980082)
Supplement: Supplementary file 9 [file Table_9.doc]

rn:R00311 <-- [Oxidized NADPH---hemoprotein reductase] --> rn:R08551 -- [Reduced NADPH---hemoprotein reductase] --> rn:R03006 { Formononetin }

rn:R00311 <-- [Oxidized NADPH---hemoprotein reductase] --> rn:R08551 -- [Reduced NADPH---hemoprotein reductase] --> rn:R03615 { Flavonoid }

rn:R00311 <-- [Oxidized NADPH---hemoprotein reductase] --> rn:R08551 -- [Reduced NADPH---hemoprotein reductase] --> rn:R06560 { Formononetin }

rn:R00311 <-- [Oxidized NADPH---hemoprotein reductase] --> rn:R08551 -- [Reduced NADPH---hemoprotein reductase] --> rn:R07198 { Liquiritigenin }

rn:R00311 <-- [Oxidized NADPH---hemoprotein reductase] --> rn:R08551 -- [Reduced NADPH---hemoprotein reductase] --> rn:R07745 { Calycosin }

rn:R00311 <-- [Oxidized NADPH---hemoprotein reductase] --> rn:R08551 -- [Reduced NADPH---hemoprotein reductase] --> rn:R07777 { Liquiritigenin }

rn:R00311 <-- [Oxidized NADPH---hemoprotein reductase] --> rn:R08551 -- [Reduced NADPH---hemoprotein reductase] --> rn:R08002 { Liquiritigenin }

rn:R00494 <-- L-Glutamate --> rn:R00114 -- 2-Oxoglutarate --> rn:R07712 { Liquiritigenin }

rn:R00494 <-- L-Glutamate --> rn:R00114 -- 2-Oxoglutarate --> rn:R07996 { Liquiritigenin }

rn:R00494 <-- L-Glutamate --> rn:R00248 -- 2-Oxoglutarate --> rn:R07712 { Liquiritigenin }

rn:R00494 <-- L-Glutamate --> rn:R00248 -- 2-Oxoglutarate --> rn:R07996 { Liquiritigenin }

rn:R00494 <-- L-Glutamate --> rn:R04051 -- 2-Oxoglutarate --> rn:R07712 { Liquiritigenin }

rn:R00494 <-- L-Glutamate --> rn:R04051 -- 2-Oxoglutarate --> rn:R07996 { Liquiritigenin }

rn:R00525 <-- L-Glutamate --> rn:R00114 -- 2-Oxoglutarate --> rn:R07712 { Liquiritigenin }

rn:R00525 <-- L-Glutamate --> rn:R00114 -- 2-Oxoglutarate --> rn:R07996 { Liquiritigenin }

rn:R00525 <-- L-Glutamate --> rn:R00248 -- 2-Oxoglutarate --> rn:R07712 { Liquiritigenin }

rn:R00525 <-- L-Glutamate --> rn:R00248 -- 2-Oxoglutarate --> rn:R07996 { Liquiritigenin }

rn:R00525 <-- L-Glutamate --> rn:R04051 -- 2-Oxoglutarate --> rn:R07712 { Liquiritigenin }

rn:R00525 <-- L-Glutamate --> rn:R04051 -- 2-Oxoglutarate --> rn:R07996 { Liquiritigenin }

rn:R00661 <-- 3-Phosphonopyruvate --> rn:R04051 -- 2-Oxoglutarate --> rn:R07712 { Liquiritigenin }

rn:R00661 <-- 3-Phosphonopyruvate --> rn:R04051 -- 2-Oxoglutarate --> rn:R07996 { Liquiritigenin }

rn:R00694 <-- L-Glutamate --> rn:R00114 -- 2-Oxoglutarate --> rn:R07712 { Liquiritigenin }

rn:R00694 <-- L-Glutamate --> rn:R00114 -- 2-Oxoglutarate --> rn:R07996 { Liquiritigenin }

rn:R00694 <-- L-Glutamate --> rn:R00248 -- 2-Oxoglutarate --> rn:R07712 { Liquiritigenin }

rn:R00694 <-- L-Glutamate --> rn:R00248 -- 2-Oxoglutarate --> rn:R07996 { Liquiritigenin }

rn:R00694 <-- L-Glutamate --> rn:R04051 -- 2-Oxoglutarate --> rn:R07712 { Liquiritigenin }

rn:R00694 <-- L-Glutamate --> rn:R04051 -- 2-Oxoglutarate --> rn:R07996 { Liquiritigenin }

rn:R00708 <-- L-Glutamate --> rn:R00114 -- 2-Oxoglutarate --> rn:R07712 { Liquiritigenin }

rn:R00708 <-- L-Glutamate --> rn:R00114 -- 2-Oxoglutarate --> rn:R07996 { Liquiritigenin }

rn:R00708 <-- L-Glutamate --> rn:R00248 -- 2-Oxoglutarate --> rn:R07712 { Liquiritigenin }

rn:R00708 <-- L-Glutamate --> rn:R00248 -- 2-Oxoglutarate --> rn:R07996 { Liquiritigenin }

rn:R00708 <-- L-Glutamate --> rn:R04051 -- 2-Oxoglutarate --> rn:R07712 { Liquiritigenin }

rn:R00708 <-- L-Glutamate --> rn:R04051 -- 2-Oxoglutarate --> rn:R07996 { Liquiritigenin }

rn:R00730 <-- [Oxidized NADPH---hemoprotein reductase] --> rn:R08551 -- [Reduced NADPH---hemoprotein reductase] --> rn:R03006 { Formononetin }

rn:R00730 <-- [Oxidized NADPH---hemoprotein reductase] --> rn:R08551 -- [Reduced NADPH---hemoprotein reductase] --> rn:R03615 { Flavonoid }

rn:R00730 <-- [Oxidized NADPH---hemoprotein reductase] --> rn:R08551 -- [Reduced NADPH---hemoprotein reductase] --> rn:R06560 { Formononetin }

rn:R00730 <-- [Oxidized NADPH---hemoprotein reductase] --> rn:R08551 -- [Reduced NADPH---hemoprotein reductase] --> rn:R07198 { Liquiritigenin }

rn:R00730 <-- [Oxidized NADPH---hemoprotein reductase] --> rn:R08551 -- [Reduced NADPH---hemoprotein reductase] --> rn:R07745 { Calycosin }

rn:R00730 <-- [Oxidized NADPH---hemoprotein reductase] --> rn:R08551 -- [Reduced NADPH---hemoprotein reductase] --> rn:R07777 { Liquiritigenin }

rn:R00730 <-- [Oxidized NADPH---hemoprotein reductase] --> rn:R08551 -- [Reduced NADPH---hemoprotein reductase] --> rn:R08002 { Liquiritigenin }

rn:R00986 <-- L-Glutamate --> rn:R00114 -- 2-Oxoglutarate --> rn:R07712 { Liquiritigenin }

rn:R00986 <-- L-Glutamate --> rn:R00114 -- 2-Oxoglutarate --> rn:R07996 { Liquiritigenin }

rn:R00986 <-- L-Glutamate --> rn:R00248 -- 2-Oxoglutarate --> rn:R07712 { Liquiritigenin }

rn:R00986 <-- L-Glutamate --> rn:R00248 -- 2-Oxoglutarate --> rn:R07996 { Liquiritigenin }

rn:R00986 <-- L-Glutamate --> rn:R04051 -- 2-Oxoglutarate --> rn:R07712 { Liquiritigenin }

rn:R00986 <-- L-Glutamate --> rn:R04051 -- 2-Oxoglutarate --> rn:R07996 { Liquiritigenin }

rn:R01161 <-- L-Glutamate --> rn:R00114 -- 2-Oxoglutarate --> rn:R07712 { Liquiritigenin }

rn:R01161 <-- L-Glutamate --> rn:R00114 -- 2-Oxoglutarate --> rn:R07996 { Liquiritigenin }

rn:R01161 <-- L-Glutamate --> rn:R00248 -- 2-Oxoglutarate --> rn:R07712 { Liquiritigenin }

rn:R01161 <-- L-Glutamate --> rn:R00248 -- 2-Oxoglutarate --> rn:R07996 { Liquiritigenin }

rn:R01161 <-- L-Glutamate --> rn:R04051 -- 2-Oxoglutarate --> rn:R07712 { Liquiritigenin }

rn:R01161 <-- L-Glutamate --> rn:R04051 -- 2-Oxoglutarate --> rn:R07996 { Liquiritigenin }

rn:R01295 <-- [Oxidized NADPH---hemoprotein reductase] --> rn:R08551 -- [Reduced NADPH---hemoprotein reductase] --> rn:R03006 { Formononetin }

rn:R01295 <-- [Oxidized NADPH---hemoprotein reductase] --> rn:R08551 -- [Reduced NADPH---hemoprotein reductase] --> rn:R03615 { Flavonoid }

rn:R01295 <-- [Oxidized NADPH---hemoprotein reductase] --> rn:R08551 -- [Reduced NADPH---hemoprotein reductase] --> rn:R06560 { Formononetin }

rn:R01295 <-- [Oxidized NADPH---hemoprotein reductase] --> rn:R08551 -- [Reduced NADPH---hemoprotein reductase] --> rn:R07198 { Liquiritigenin }

rn:R01295 <-- [Oxidized NADPH---hemoprotein reductase] --> rn:R08551 -- [Reduced NADPH---hemoprotein reductase] --> rn:R07745 { Calycosin }

rn:R01295 <-- [Oxidized NADPH---hemoprotein reductase] --> rn:R08551 -- [Reduced NADPH---hemoprotein reductase] --> rn:R07777 { Liquiritigenin }

rn:R01295 <-- [Oxidized NADPH---hemoprotein reductase] --> rn:R08551 -- [Reduced NADPH---hemoprotein reductase] --> rn:R08002 { Liquiritigenin }

rn:R01348 <-- [Oxidized NADPH---hemoprotein reductase] --> rn:R08551 -- [Reduced NADPH---hemoprotein reductase] --> rn:R03006 { Formononetin }

rn:R01348 <-- [Oxidized NADPH---hemoprotein reductase] --> rn:R08551 -- [Reduced NADPH---hemoprotein reductase] --> rn:R03615 { Flavonoid }

rn:R01348 <-- [Oxidized NADPH---hemoprotein reductase] --> rn:R08551 -- [Reduced NADPH---hemoprotein reductase] --> rn:R06560 { Formononetin }

rn:R01348 <-- [Oxidized NADPH---hemoprotein reductase] --> rn:R08551 -- [Reduced NADPH---hemoprotein reductase] --> rn:R07198 { Liquiritigenin }

rn:R01348 <-- [Oxidized NADPH---hemoprotein reductase] --> rn:R08551 -- [Reduced NADPH---hemoprotein reductase] --> rn:R07745 { Calycosin }

rn:R01348 <-- [Oxidized NADPH---hemoprotein reductase] --> rn:R08551 -- [Reduced NADPH---hemoprotein reductase] --> rn:R07777 { Liquiritigenin }

rn:R01348 <-- [Oxidized NADPH---hemoprotein reductase] --> rn:R08551 -- [Reduced NADPH---hemoprotein reductase] --> rn:R08002 { Liquiritigenin }

rn:R02077 <-- L-Glutamate --> rn:R00114 -- 2-Oxoglutarate --> rn:R07712 { Liquiritigenin }

rn:R02077 <-- L-Glutamate --> rn:R00114 -- 2-Oxoglutarate --> rn:R07996 { Liquiritigenin }

rn:R02077 <-- L-Glutamate --> rn:R00248 -- 2-Oxoglutarate --> rn:R07712 { Liquiritigenin }

rn:R02077 <-- L-Glutamate --> rn:R00248 -- 2-Oxoglutarate --> rn:R07996 { Liquiritigenin }

rn:R02077 <-- L-Glutamate --> rn:R04051 -- 2-Oxoglutarate --> rn:R07712 { Liquiritigenin }

rn:R02077 <-- L-Glutamate --> rn:R04051 -- 2-Oxoglutarate --> rn:R07996 { Liquiritigenin }

rn:R02253 <-- [Oxidized NADPH---hemoprotein reductase] --> rn:R08551 -- [Reduced NADPH---hemoprotein reductase] --> rn:R03006 { Formononetin }

rn:R02253 <-- [Oxidized NADPH---hemoprotein reductase] --> rn:R08551 -- [Reduced NADPH---hemoprotein reductase] --> rn:R03615 { Flavonoid }

rn:R02253 <-- [Oxidized NADPH---hemoprotein reductase] --> rn:R08551 -- [Reduced NADPH---hemoprotein reductase] --> rn:R06560 { Formononetin }

rn:R02253 <-- [Oxidized NADPH---hemoprotein reductase] --> rn:R08551 -- [Reduced NADPH---hemoprotein reductase] --> rn:R07198 { Liquiritigenin }

rn:R02253 <-- [Oxidized NADPH---hemoprotein reductase] --> rn:R08551 -- [Reduced NADPH---hemoprotein reductase] --> rn:R07745 { Calycosin }

rn:R02253 <-- [Oxidized NADPH---hemoprotein reductase] --> rn:R08551 -- [Reduced NADPH---hemoprotein reductase] --> rn:R07777 { Liquiritigenin }

rn:R02253 <-- [Oxidized NADPH---hemoprotein reductase] --> rn:R08551 -- [Reduced NADPH---hemoprotein reductase] --> rn:R08002 { Liquiritigenin }

rn:R02285 <-- L-Glutamate --> rn:R00114 -- 2-Oxoglutarate --> rn:R07712 { Liquiritigenin }

rn:R02285 <-- L-Glutamate --> rn:R00114 -- 2-Oxoglutarate --> rn:R07996 { Liquiritigenin }

rn:R02285 <-- L-Glutamate --> rn:R00248 -- 2-Oxoglutarate --> rn:R07712 { Liquiritigenin }

rn:R02285 <-- L-Glutamate --> rn:R00248 -- 2-Oxoglutarate --> rn:R07996 { Liquiritigenin }

rn:R02285 <-- L-Glutamate --> rn:R04051 -- 2-Oxoglutarate --> rn:R07712 { Liquiritigenin }

rn:R02285 <-- L-Glutamate --> rn:R04051 -- 2-Oxoglutarate --> rn:R07996 { Liquiritigenin }

rn:R02315 <-- L-Glutamate --> rn:R00114 -- 2-Oxoglutarate --> rn:R07712 { Liquiritigenin }

rn:R02315 <-- L-Glutamate --> rn:R00114 -- 2-Oxoglutarate --> rn:R07996 { Liquiritigenin }

rn:R02315 <-- L-Glutamate --> rn:R00248 -- 2-Oxoglutarate --> rn:R07712 { Liquiritigenin }

rn:R02315 <-- L-Glutamate --> rn:R00248 -- 2-Oxoglutarate --> rn:R07996 { Liquiritigenin }

rn:R02315 <-- L-Glutamate --> rn:R04051 -- 2-Oxoglutarate --> rn:R07712 { Liquiritigenin }

rn:R02315 <-- L-Glutamate --> rn:R04051 -- 2-Oxoglutarate --> rn:R07996 { Liquiritigenin }

rn:R02708 <-- [Oxidized NADPH---hemoprotein reductase] --> rn:R08551 -- [Reduced NADPH---hemoprotein reductase] --> rn:R03006 { Formononetin }

rn:R02708 <-- [Oxidized NADPH---hemoprotein reductase] --> rn:R08551 -- [Reduced NADPH---hemoprotein reductase] --> rn:R03615 { Flavonoid }

rn:R02708 <-- [Oxidized NADPH---hemoprotein reductase] --> rn:R08551 -- [Reduced NADPH---hemoprotein reductase] --> rn:R06560 { Formononetin }

rn:R02708 <-- [Oxidized NADPH---hemoprotein reductase] --> rn:R08551 -- [Reduced NADPH---hemoprotein reductase] --> rn:R07198 { Liquiritigenin }

rn:R02708 <-- [Oxidized NADPH---hemoprotein reductase] --> rn:R08551 -- [Reduced NADPH---hemoprotein reductase] --> rn:R07745 { Calycosin }

rn:R02708 <-- [Oxidized NADPH---hemoprotein reductase] --> rn:R08551 -- [Reduced NADPH---hemoprotein reductase] --> rn:R07777 { Liquiritigenin }

rn:R02708 <-- [Oxidized NADPH---hemoprotein reductase] --> rn:R08551 -- [Reduced NADPH---hemoprotein reductase] --> rn:R08002 { Liquiritigenin }

rn:R02772 <-- L-Glutamate --> rn:R00114 -- 2-Oxoglutarate --> rn:R07712 { Liquiritigenin }

rn:R02772 <-- L-Glutamate --> rn:R00114 -- 2-Oxoglutarate --> rn:R07996 { Liquiritigenin }

rn:R02772 <-- L-Glutamate --> rn:R00248 -- 2-Oxoglutarate --> rn:R07712 { Liquiritigenin }

rn:R02772 <-- L-Glutamate --> rn:R00248 -- 2-Oxoglutarate --> rn:R07996 { Liquiritigenin }

rn:R02772 <-- L-Glutamate --> rn:R04051 -- 2-Oxoglutarate --> rn:R07712 { Liquiritigenin }

rn:R02772 <-- L-Glutamate --> rn:R04051 -- 2-Oxoglutarate --> rn:R07996 { Liquiritigenin }

rn:R02773 <-- L-Glutamate --> rn:R00114 -- 2-Oxoglutarate --> rn:R07712 { Liquiritigenin }

rn:R02773 <-- L-Glutamate --> rn:R00114 -- 2-Oxoglutarate --> rn:R07996 { Liquiritigenin }

rn:R02773 <-- L-Glutamate --> rn:R00248 -- 2-Oxoglutarate --> rn:R07712 { Liquiritigenin }

rn:R02773 <-- L-Glutamate --> rn:R00248 -- 2-Oxoglutarate --> rn:R07996 { Liquiritigenin }

rn:R02773 <-- L-Glutamate --> rn:R04051 -- 2-Oxoglutarate --> rn:R07712 { Liquiritigenin }

rn:R02773 <-- L-Glutamate --> rn:R04051 -- 2-Oxoglutarate --> rn:R07996 { Liquiritigenin }

rn:R03207 <-- L-Glutamate --> rn:R00114 -- 2-Oxoglutarate --> rn:R07712 { Liquiritigenin }

rn:R03207 <-- L-Glutamate --> rn:R00114 -- 2-Oxoglutarate --> rn:R07996 { Liquiritigenin }

rn:R03207 <-- L-Glutamate --> rn:R00248 -- 2-Oxoglutarate --> rn:R07712 { Liquiritigenin }

rn:R03207 <-- L-Glutamate --> rn:R00248 -- 2-Oxoglutarate --> rn:R07996 { Liquiritigenin }

rn:R03207 <-- L-Glutamate --> rn:R04051 -- 2-Oxoglutarate --> rn:R07712 { Liquiritigenin }

rn:R03207 <-- L-Glutamate --> rn:R04051 -- 2-Oxoglutarate --> rn:R07996 { Liquiritigenin }

rn:R03243 <-- L-Glutamate --> rn:R00114 -- 2-Oxoglutarate --> rn:R07712 { Liquiritigenin }

rn:R03243 <-- L-Glutamate --> rn:R00114 -- 2-Oxoglutarate --> rn:R07996 { Liquiritigenin }

rn:R03243 <-- L-Glutamate --> rn:R00248 -- 2-Oxoglutarate --> rn:R07712 { Liquiritigenin }

rn:R03243 <-- L-Glutamate --> rn:R00248 -- 2-Oxoglutarate --> rn:R07996 { Liquiritigenin }

rn:R03243 <-- L-Glutamate --> rn:R04051 -- 2-Oxoglutarate --> rn:R07712 { Liquiritigenin }

rn:R03243 <-- L-Glutamate --> rn:R04051 -- 2-Oxoglutarate --> rn:R07996 { Liquiritigenin }

rn:R03916 <-- L-Glutamate --> rn:R00114 -- 2-Oxoglutarate --> rn:R07712 { Liquiritigenin }

rn:R03916 <-- L-Glutamate --> rn:R00114 -- 2-Oxoglutarate --> rn:R07996 { Liquiritigenin }

rn:R03916 <-- L-Glutamate --> rn:R00248 -- 2-Oxoglutarate --> rn:R07712 { Liquiritigenin }

rn:R03916 <-- L-Glutamate --> rn:R00248 -- 2-Oxoglutarate --> rn:R07996 { Liquiritigenin }

rn:R03916 <-- L-Glutamate --> rn:R04051 -- 2-Oxoglutarate --> rn:R07712 { Liquiritigenin }

rn:R03916 <-- L-Glutamate --> rn:R04051 -- 2-Oxoglutarate --> rn:R07996 { Liquiritigenin }

rn:R03952 <-- L-Glutamate --> rn:R00114 -- 2-Oxoglutarate --> rn:R07712 { Liquiritigenin }

rn:R03952 <-- L-Glutamate --> rn:R00114 -- 2-Oxoglutarate --> rn:R07996 { Liquiritigenin }

rn:R03952 <-- L-Glutamate --> rn:R00248 -- 2-Oxoglutarate --> rn:R07712 { Liquiritigenin }

rn:R03952 <-- L-Glutamate --> rn:R00248 -- 2-Oxoglutarate --> rn:R07996 { Liquiritigenin }

rn:R03952 <-- L-Glutamate --> rn:R04051 -- 2-Oxoglutarate --> rn:R07712 { Liquiritigenin }

rn:R03952 <-- L-Glutamate --> rn:R04051 -- 2-Oxoglutarate --> rn:R07996 { Liquiritigenin }

rn:R04121 <-- [Oxidized NADPH---hemoprotein reductase] --> rn:R08551 -- [Reduced NADPH---hemoprotein reductase] --> rn:R03006 { Formononetin }

rn:R04121 <-- [Oxidized NADPH---hemoprotein reductase] --> rn:R08551 -- [Reduced NADPH---hemoprotein reductase] --> rn:R03615 { Flavonoid }

rn:R04121 <-- [Oxidized NADPH---hemoprotein reductase] --> rn:R08551 -- [Reduced NADPH---hemoprotein reductase] --> rn:R06560 { Formononetin }

rn:R04121 <-- [Oxidized NADPH---hemoprotein reductase] --> rn:R08551 -- [Reduced NADPH---hemoprotein reductase] --> rn:R07198 { Liquiritigenin }

rn:R04121 <-- [Oxidized NADPH---hemoprotein reductase] --> rn:R08551 -- [Reduced NADPH---hemoprotein reductase] --> rn:R07745 { Calycosin }

rn:R04121 <-- [Oxidized NADPH---hemoprotein reductase] --> rn:R08551 -- [Reduced NADPH---hemoprotein reductase] --> rn:R07777 { Liquiritigenin }

rn:R04121 <-- [Oxidized NADPH---hemoprotein reductase] --> rn:R08551 -- [Reduced NADPH---hemoprotein reductase] --> rn:R08002 { Liquiritigenin }

rn:R04122 <-- [Oxidized NADPH---hemoprotein reductase] --> rn:R08551 -- [Reduced NADPH---hemoprotein reductase] --> rn:R03006 { Formononetin }

rn:R04122 <-- [Oxidized NADPH---hemoprotein reductase] --> rn:R08551 -- [Reduced NADPH---hemoprotein reductase] --> rn:R03615 { Flavonoid }

rn:R04122 <-- [Oxidized NADPH---hemoprotein reductase] --> rn:R08551 -- [Reduced NADPH---hemoprotein reductase] --> rn:R06560 { Formononetin }

rn:R04122 <-- [Oxidized NADPH---hemoprotein reductase] --> rn:R08551 -- [Reduced NADPH---hemoprotein reductase] --> rn:R07198 { Liquiritigenin }

rn:R04122 <-- [Oxidized NADPH---hemoprotein reductase] --> rn:R08551 -- [Reduced NADPH---hemoprotein reductase] --> rn:R07745 { Calycosin }

rn:R04122 <-- [Oxidized NADPH---hemoprotein reductase] --> rn:R08551 -- [Reduced NADPH---hemoprotein reductase] --> rn:R07777 { Liquiritigenin }

rn:R04122 <-- [Oxidized NADPH---hemoprotein reductase] --> rn:R08551 -- [Reduced NADPH---hemoprotein reductase] --> rn:R08002 { Liquiritigenin }

rn:R04188 <-- L-Glutamate --> rn:R00114 -- 2-Oxoglutarate --> rn:R07712 { Liquiritigenin }

rn:R04188 <-- L-Glutamate --> rn:R00114 -- 2-Oxoglutarate --> rn:R07996 { Liquiritigenin }

rn:R04188 <-- L-Glutamate --> rn:R00248 -- 2-Oxoglutarate --> rn:R07712 { Liquiritigenin }

rn:R04188 <-- L-Glutamate --> rn:R00248 -- 2-Oxoglutarate --> rn:R07996 { Liquiritigenin }

rn:R04188 <-- L-Glutamate --> rn:R04051 -- 2-Oxoglutarate --> rn:R07712 { Liquiritigenin }

rn:R04188 <-- L-Glutamate --> rn:R04051 -- 2-Oxoglutarate --> rn:R07996 { Liquiritigenin }

rn:R04234 <-- L-Glutamate --> rn:R00114 -- 2-Oxoglutarate --> rn:R07712 { Liquiritigenin }

rn:R04234 <-- L-Glutamate --> rn:R00114 -- 2-Oxoglutarate --> rn:R07996 { Liquiritigenin }

rn:R04234 <-- L-Glutamate --> rn:R00248 -- 2-Oxoglutarate --> rn:R07712 { Liquiritigenin }

rn:R04234 <-- L-Glutamate --> rn:R00248 -- 2-Oxoglutarate --> rn:R07996 { Liquiritigenin }

rn:R04234 <-- L-Glutamate --> rn:R04051 -- 2-Oxoglutarate --> rn:R07712 { Liquiritigenin }

rn:R04234 <-- L-Glutamate --> rn:R04051 -- 2-Oxoglutarate --> rn:R07996 { Liquiritigenin }

rn:R04269 <-- L-Glutamate --> rn:R00114 -- 2-Oxoglutarate --> rn:R07712 { Liquiritigenin }

rn:R04269 <-- L-Glutamate --> rn:R00114 -- 2-Oxoglutarate --> rn:R07996 { Liquiritigenin }

rn:R04269 <-- L-Glutamate --> rn:R00248 -- 2-Oxoglutarate --> rn:R07712 { Liquiritigenin }

rn:R04269 <-- L-Glutamate --> rn:R00248 -- 2-Oxoglutarate --> rn:R07996 { Liquiritigenin }

rn:R04269 <-- L-Glutamate --> rn:R04051 -- 2-Oxoglutarate --> rn:R07712 { Liquiritigenin }

rn:R04269 <-- L-Glutamate --> rn:R04051 -- 2-Oxoglutarate --> rn:R07996 { Liquiritigenin }

rn:R04438 <-- L-Glutamate --> rn:R00114 -- 2-Oxoglutarate --> rn:R07712 { Liquiritigenin }

rn:R04438 <-- L-Glutamate --> rn:R00114 -- 2-Oxoglutarate --> rn:R07996 { Liquiritigenin }

rn:R04438 <-- L-Glutamate --> rn:R00248 -- 2-Oxoglutarate --> rn:R07712 { Liquiritigenin }

rn:R04438 <-- L-Glutamate --> rn:R00248 -- 2-Oxoglutarate --> rn:R07996 { Liquiritigenin }

rn:R04438 <-- L-Glutamate --> rn:R04051 -- 2-Oxoglutarate --> rn:R07712 { Liquiritigenin }

rn:R04438 <-- L-Glutamate --> rn:R04051 -- 2-Oxoglutarate --> rn:R07996 { Liquiritigenin }

rn:R04460 <-- [Oxidized NADPH---hemoprotein reductase] --> rn:R08551 -- [Reduced NADPH---hemoprotein reductase] --> rn:R03006 { Formononetin }

rn:R04460 <-- [Oxidized NADPH---hemoprotein reductase] --> rn:R08551 -- [Reduced NADPH---hemoprotein reductase] --> rn:R03615 { Flavonoid }

rn:R04460 <-- [Oxidized NADPH---hemoprotein reductase] --> rn:R08551 -- [Reduced NADPH---hemoprotein reductase] --> rn:R06560 { Formononetin }

rn:R04460 <-- [Oxidized NADPH---hemoprotein reductase] --> rn:R08551 -- [Reduced NADPH---hemoprotein reductase] --> rn:R07198 { Liquiritigenin }

rn:R04460 <-- [Oxidized NADPH---hemoprotein reductase] --> rn:R08551 -- [Reduced NADPH---hemoprotein reductase] --> rn:R07745 { Calycosin }

rn:R04460 <-- [Oxidized NADPH---hemoprotein reductase] --> rn:R08551 -- [Reduced NADPH---hemoprotein reductase] --> rn:R07777 { Liquiritigenin }

rn:R04460 <-- [Oxidized NADPH---hemoprotein reductase] --> rn:R08551 -- [Reduced NADPH---hemoprotein reductase] --> rn:R08002 { Liquiritigenin }

rn:R05085 <-- L-Glutamate --> rn:R00114 -- 2-Oxoglutarate --> rn:R07712 { Liquiritigenin }

rn:R05085 <-- L-Glutamate --> rn:R00114 -- 2-Oxoglutarate --> rn:R07996 { Liquiritigenin }

rn:R05085 <-- L-Glutamate --> rn:R00248 -- 2-Oxoglutarate --> rn:R07712 { Liquiritigenin }

rn:R05085 <-- L-Glutamate --> rn:R00248 -- 2-Oxoglutarate --> rn:R07996 { Liquiritigenin }

rn:R05085 <-- L-Glutamate --> rn:R04051 -- 2-Oxoglutarate --> rn:R07712 { Liquiritigenin }

rn:R05085 <-- L-Glutamate --> rn:R04051 -- 2-Oxoglutarate --> rn:R07996 { Liquiritigenin }

rn:R05224 <-- L-Glutamate --> rn:R00114 -- 2-Oxoglutarate --> rn:R07712 { Liquiritigenin }

rn:R05224 <-- L-Glutamate --> rn:R00114 -- 2-Oxoglutarate --> rn:R07996 { Liquiritigenin }

rn:R05224 <-- L-Glutamate --> rn:R00248 -- 2-Oxoglutarate --> rn:R07712 { Liquiritigenin }

rn:R05224 <-- L-Glutamate --> rn:R00248 -- 2-Oxoglutarate --> rn:R07996 { Liquiritigenin }

rn:R05224 <-- L-Glutamate --> rn:R04051 -- 2-Oxoglutarate --> rn:R07712 { Liquiritigenin }

rn:R05224 <-- L-Glutamate --> rn:R04051 -- 2-Oxoglutarate --> rn:R07996 { Liquiritigenin }

rn:R05225 <-- L-Glutamate --> rn:R00114 -- 2-Oxoglutarate --> rn:R07712 { Liquiritigenin }

rn:R05225 <-- L-Glutamate --> rn:R00114 -- 2-Oxoglutarate --> rn:R07996 { Liquiritigenin }

rn:R05225 <-- L-Glutamate --> rn:R00248 -- 2-Oxoglutarate --> rn:R07712 { Liquiritigenin }

rn:R05225 <-- L-Glutamate --> rn:R00248 -- 2-Oxoglutarate --> rn:R07996 { Liquiritigenin }

rn:R05225 <-- L-Glutamate --> rn:R04051 -- 2-Oxoglutarate --> rn:R07712 { Liquiritigenin }

rn:R05225 <-- L-Glutamate --> rn:R04051 -- 2-Oxoglutarate --> rn:R07996 { Liquiritigenin }

rn:R05259 <-- [Oxidized NADPH---hemoprotein reductase] --> rn:R08551 -- [Reduced NADPH---hemoprotein reductase] --> rn:R03006 { Formononetin }

rn:R05259 <-- [Oxidized NADPH---hemoprotein reductase] --> rn:R08551 -- [Reduced NADPH---hemoprotein reductase] --> rn:R03615 { Flavonoid }

rn:R05259 <-- [Oxidized NADPH---hemoprotein reductase] --> rn:R08551 -- [Reduced NADPH---hemoprotein reductase] --> rn:R06560 { Formononetin }

rn:R05259 <-- [Oxidized NADPH---hemoprotein reductase] --> rn:R08551 -- [Reduced NADPH---hemoprotein reductase] --> rn:R07198 { Liquiritigenin }

rn:R05259 <-- [Oxidized NADPH---hemoprotein reductase] --> rn:R08551 -- [Reduced NADPH---hemoprotein reductase] --> rn:R07745 { Calycosin }

rn:R05259 <-- [Oxidized NADPH---hemoprotein reductase] --> rn:R08551 -- [Reduced NADPH---hemoprotein reductase] --> rn:R07777 { Liquiritigenin }

rn:R05259 <-- [Oxidized NADPH---hemoprotein reductase] --> rn:R08551 -- [Reduced NADPH---hemoprotein reductase] --> rn:R08002 { Liquiritigenin }

rn:R05487 <-- [Oxidized NADPH---hemoprotein reductase] --> rn:R08551 -- [Reduced NADPH---hemoprotein reductase] --> rn:R03006 { Formononetin }

rn:R05487 <-- [Oxidized NADPH---hemoprotein reductase] --> rn:R08551 -- [Reduced NADPH---hemoprotein reductase] --> rn:R03615 { Flavonoid }

rn:R05487 <-- [Oxidized NADPH---hemoprotein reductase] --> rn:R08551 -- [Reduced NADPH---hemoprotein reductase] --> rn:R06560 { Formononetin }

rn:R05487 <-- [Oxidized NADPH---hemoprotein reductase] --> rn:R08551 -- [Reduced NADPH---hemoprotein reductase] --> rn:R07198 { Liquiritigenin }

rn:R05487 <-- [Oxidized NADPH---hemoprotein reductase] --> rn:R08551 -- [Reduced NADPH---hemoprotein reductase] --> rn:R07745 { Calycosin }

rn:R05487 <-- [Oxidized NADPH---hemoprotein reductase] --> rn:R08551 -- [Reduced NADPH---hemoprotein reductase] --> rn:R07777 { Liquiritigenin }

rn:R05487 <-- [Oxidized NADPH---hemoprotein reductase] --> rn:R08551 -- [Reduced NADPH---hemoprotein reductase] --> rn:R08002 { Liquiritigenin }

rn:R05728 <-- [Oxidized NADPH---hemoprotein reductase] --> rn:R08551 -- [Reduced NADPH---hemoprotein reductase] --> rn:R03006 { Formononetin }

rn:R05728 <-- [Oxidized NADPH---hemoprotein reductase] --> rn:R08551 -- [Reduced NADPH---hemoprotein reductase] --> rn:R03615 { Flavonoid }

rn:R05728 <-- [Oxidized NADPH---hemoprotein reductase] --> rn:R08551 -- [Reduced NADPH---hemoprotein reductase] --> rn:R06560 { Formononetin }

rn:R05728 <-- [Oxidized NADPH---hemoprotein reductase] --> rn:R08551 -- [Reduced NADPH---hemoprotein reductase] --> rn:R07198 { Liquiritigenin }

rn:R05728 <-- [Oxidized NADPH---hemoprotein reductase] --> rn:R08551 -- [Reduced NADPH---hemoprotein reductase] --> rn:R07745 { Calycosin }

rn:R05728 <-- [Oxidized NADPH---hemoprotein reductase] --> rn:R08551 -- [Reduced NADPH---hemoprotein reductase] --> rn:R07777 { Liquiritigenin }

rn:R05728 <-- [Oxidized NADPH---hemoprotein reductase] --> rn:R08551 -- [Reduced NADPH---hemoprotein reductase] --> rn:R08002 { Liquiritigenin }

rn:R05815 <-- L-Glutamate --> rn:R00114 -- 2-Oxoglutarate --> rn:R07712 { Liquiritigenin }

rn:R05815 <-- L-Glutamate --> rn:R00114 -- 2-Oxoglutarate --> rn:R07996 { Liquiritigenin }

rn:R05815 <-- L-Glutamate --> rn:R00248 -- 2-Oxoglutarate --> rn:R07712 { Liquiritigenin }

rn:R05815 <-- L-Glutamate --> rn:R00248 -- 2-Oxoglutarate --> rn:R07996 { Liquiritigenin }

rn:R05815 <-- L-Glutamate --> rn:R04051 -- 2-Oxoglutarate --> rn:R07712 { Liquiritigenin }

rn:R05815 <-- L-Glutamate --> rn:R04051 -- 2-Oxoglutarate --> rn:R07996 { Liquiritigenin }

rn:R06758 <-- 3-Dimethylallyl-4-hydroxymandelic acid --> rn:R06759 -- 3-Dimethylallyl-4-hydroxybenzoate --> rn:R06776 { 3-Amino-4,7-dihydroxy-8-chlorocoumarin }

rn:R06758 <-- 3-Dimethylallyl-4-hydroxymandelic acid --> rn:R06759 -- 3-Dimethylallyl-4-hydroxybenzoate --> rn:R10453 { 3-Amino-4,7-dihydroxycoumarin }

rn:R06761 <-- 3-Dimethylallyl-4-hydroxybenzaldehyde --> rn:R06763 -- 3-Dimethylallyl-4-hydroxybenzoate --> rn:R06776 { 3-Amino-4,7-dihydroxy-8-chlorocoumarin }

rn:R06761 <-- 3-Dimethylallyl-4-hydroxybenzaldehyde --> rn:R06763 -- 3-Dimethylallyl-4-hydroxybenzoate --> rn:R10453 { 3-Amino-4,7-dihydroxycoumarin }

rn:R06762 <-- 3-Dimethylallyl-4-hydroxybenzaldehyde --> rn:R06763 -- 3-Dimethylallyl-4-hydroxybenzoate --> rn:R06776 { 3-Amino-4,7-dihydroxy-8-chlorocoumarin }

rn:R06762 <-- 3-Dimethylallyl-4-hydroxybenzaldehyde --> rn:R06763 -- 3-Dimethylallyl-4-hydroxybenzoate --> rn:R10453 { 3-Amino-4,7-dihydroxycoumarin }

rn:R06765 <-- 3-Amino-4,7-dihydroxycoumarin --> rn:R06777 -- 5-[[(4,7-Dihydroxy-2-oxo-2H-1-benzopyran-3-yl)amino]carbonyl]-4-methyl-1H-pyrrole-3-carboxylate --> rn:R06778 { 3-Amino-4,7-dihydroxycoumarin }

rn:R07041 <-- [Oxidized NADPH---hemoprotein reductase] --> rn:R08551 -- [Reduced NADPH---hemoprotein reductase] --> rn:R03006 { Formononetin }

rn:R07041 <-- [Oxidized NADPH---hemoprotein reductase] --> rn:R08551 -- [Reduced NADPH---hemoprotein reductase] --> rn:R03615 { Flavonoid }

rn:R07041 <-- [Oxidized NADPH---hemoprotein reductase] --> rn:R08551 -- [Reduced NADPH---hemoprotein reductase] --> rn:R06560 { Formononetin }

rn:R07041 <-- [Oxidized NADPH---hemoprotein reductase] --> rn:R08551 -- [Reduced NADPH---hemoprotein reductase] --> rn:R07198 { Liquiritigenin }

rn:R07041 <-- [Oxidized NADPH---hemoprotein reductase] --> rn:R08551 -- [Reduced NADPH---hemoprotein reductase] --> rn:R07745 { Calycosin }

rn:R07041 <-- [Oxidized NADPH---hemoprotein reductase] --> rn:R08551 -- [Reduced NADPH---hemoprotein reductase] --> rn:R07777 { Liquiritigenin }

rn:R07041 <-- [Oxidized NADPH---hemoprotein reductase] --> rn:R08551 -- [Reduced NADPH---hemoprotein reductase] --> rn:R08002 { Liquiritigenin }

rn:R07046 <-- [Oxidized NADPH---hemoprotein reductase] --> rn:R08551 -- [Reduced NADPH---hemoprotein reductase] --> rn:R03006 { Formononetin }

rn:R07046 <-- [Oxidized NADPH---hemoprotein reductase] --> rn:R08551 -- [Reduced NADPH---hemoprotein reductase] --> rn:R03615 { Flavonoid }

rn:R07046 <-- [Oxidized NADPH---hemoprotein reductase] --> rn:R08551 -- [Reduced NADPH---hemoprotein reductase] --> rn:R06560 { Formononetin }

rn:R07046 <-- [Oxidized NADPH---hemoprotein reductase] --> rn:R08551 -- [Reduced NADPH---hemoprotein reductase] --> rn:R07198 { Liquiritigenin }

rn:R07046 <-- [Oxidized NADPH---hemoprotein reductase] --> rn:R08551 -- [Reduced NADPH---hemoprotein reductase] --> rn:R07745 { Calycosin }

rn:R07046 <-- [Oxidized NADPH---hemoprotein reductase] --> rn:R08551 -- [Reduced NADPH---hemoprotein reductase] --> rn:R07777 { Liquiritigenin }

rn:R07046 <-- [Oxidized NADPH---hemoprotein reductase] --> rn:R08551 -- [Reduced NADPH---hemoprotein reductase] --> rn:R08002 { Liquiritigenin }

rn:R07203 <-- [Oxidized NADPH---hemoprotein reductase] --> rn:R08551 -- [Reduced NADPH---hemoprotein reductase] --> rn:R03006 { Formononetin }

rn:R07203 <-- [Oxidized NADPH---hemoprotein reductase] --> rn:R08551 -- [Reduced NADPH---hemoprotein reductase] --> rn:R03615 { Flavonoid }

rn:R07203 <-- [Oxidized NADPH---hemoprotein reductase] --> rn:R08551 -- [Reduced NADPH---hemoprotein reductase] --> rn:R06560 { Formononetin }

rn:R07203 <-- [Oxidized NADPH---hemoprotein reductase] --> rn:R08551 -- [Reduced NADPH---hemoprotein reductase] --> rn:R07198 { Liquiritigenin }

rn:R07203 <-- [Oxidized NADPH---hemoprotein reductase] --> rn:R08551 -- [Reduced NADPH---hemoprotein reductase] --> rn:R07745 { Calycosin }

rn:R07203 <-- [Oxidized NADPH---hemoprotein reductase] --> rn:R08551 -- [Reduced NADPH---hemoprotein reductase] --> rn:R07777 { Liquiritigenin }

rn:R07203 <-- [Oxidized NADPH---hemoprotein reductase] --> rn:R08551 -- [Reduced NADPH---hemoprotein reductase] --> rn:R08002 { Liquiritigenin }

rn:R07205 <-- [Oxidized NADPH---hemoprotein reductase] --> rn:R08551 -- [Reduced NADPH---hemoprotein reductase] --> rn:R03006 { Formononetin }

rn:R07205 <-- [Oxidized NADPH---hemoprotein reductase] --> rn:R08551 -- [Reduced NADPH---hemoprotein reductase] --> rn:R03615 { Flavonoid }

rn:R07205 <-- [Oxidized NADPH---hemoprotein reductase] --> rn:R08551 -- [Reduced NADPH---hemoprotein reductase] --> rn:R06560 { Formononetin }

rn:R07205 <-- [Oxidized NADPH---hemoprotein reductase] --> rn:R08551 -- [Reduced NADPH---hemoprotein reductase] --> rn:R07198 { Liquiritigenin }

rn:R07205 <-- [Oxidized NADPH---hemoprotein reductase] --> rn:R08551 -- [Reduced NADPH---hemoprotein reductase] --> rn:R07745 { Calycosin }

rn:R07205 <-- [Oxidized NADPH---hemoprotein reductase] --> rn:R08551 -- [Reduced NADPH---hemoprotein reductase] --> rn:R07777 { Liquiritigenin }

rn:R07205 <-- [Oxidized NADPH---hemoprotein reductase] --> rn:R08551 -- [Reduced NADPH---hemoprotein reductase] --> rn:R08002 { Liquiritigenin }

rn:R07206 <-- [Oxidized NADPH---hemoprotein reductase] --> rn:R08551 -- [Reduced NADPH---hemoprotein reductase] --> rn:R03006 { Formononetin }

rn:R07206 <-- [Oxidized NADPH---hemoprotein reductase] --> rn:R08551 -- [Reduced NADPH---hemoprotein reductase] --> rn:R03615 { Flavonoid }

rn:R07206 <-- [Oxidized NADPH---hemoprotein reductase] --> rn:R08551 -- [Reduced NADPH---hemoprotein reductase] --> rn:R06560 { Formononetin }

rn:R07206 <-- [Oxidized NADPH---hemoprotein reductase] --> rn:R08551 -- [Reduced NADPH---hemoprotein reductase] --> rn:R07198 { Liquiritigenin }

rn:R07206 <-- [Oxidized NADPH---hemoprotein reductase] --> rn:R08551 -- [Reduced NADPH---hemoprotein reductase] --> rn:R07745 { Calycosin }

rn:R07206 <-- [Oxidized NADPH---hemoprotein reductase] --> rn:R08551 -- [Reduced NADPH---hemoprotein reductase] --> rn:R07777 { Liquiritigenin }

rn:R07206 <-- [Oxidized NADPH---hemoprotein reductase] --> rn:R08551 -- [Reduced NADPH---hemoprotein reductase] --> rn:R08002 { Liquiritigenin }

rn:R07276 <-- L-Glutamate --> rn:R00114 -- 2-Oxoglutarate --> rn:R07712 { Liquiritigenin }

rn:R07276 <-- L-Glutamate --> rn:R00114 -- 2-Oxoglutarate --> rn:R07996 { Liquiritigenin }

rn:R07276 <-- L-Glutamate --> rn:R00248 -- 2-Oxoglutarate --> rn:R07712 { Liquiritigenin }

rn:R07276 <-- L-Glutamate --> rn:R00248 -- 2-Oxoglutarate --> rn:R07996 { Liquiritigenin }

rn:R07276 <-- L-Glutamate --> rn:R04051 -- 2-Oxoglutarate --> rn:R07712 { Liquiritigenin }

rn:R07276 <-- L-Glutamate --> rn:R04051 -- 2-Oxoglutarate --> rn:R07996 { Liquiritigenin }

rn:R07403 <-- [Oxidized NADPH---hemoprotein reductase] --> rn:R08551 -- [Reduced NADPH---hemoprotein reductase] --> rn:R03006 { Formononetin }

rn:R07403 <-- [Oxidized NADPH---hemoprotein reductase] --> rn:R08551 -- [Reduced NADPH---hemoprotein reductase] --> rn:R03615 { Flavonoid }

rn:R07403 <-- [Oxidized NADPH---hemoprotein reductase] --> rn:R08551 -- [Reduced NADPH---hemoprotein reductase] --> rn:R06560 { Formononetin }

rn:R07403 <-- [Oxidized NADPH---hemoprotein reductase] --> rn:R08551 -- [Reduced NADPH---hemoprotein reductase] --> rn:R07198 { Liquiritigenin }

rn:R07403 <-- [Oxidized NADPH---hemoprotein reductase] --> rn:R08551 -- [Reduced NADPH---hemoprotein reductase] --> rn:R07745 { Calycosin }

rn:R07403 <-- [Oxidized NADPH---hemoprotein reductase] --> rn:R08551 -- [Reduced NADPH---hemoprotein reductase] --> rn:R07777 { Liquiritigenin }

rn:R07403 <-- [Oxidized NADPH---hemoprotein reductase] --> rn:R08551 -- [Reduced NADPH---hemoprotein reductase] --> rn:R08002 { Liquiritigenin }

rn:R08663 <-- [Oxidized NADPH---hemoprotein reductase] --> rn:R08551 -- [Reduced NADPH---hemoprotein reductase] --> rn:R03006 { Formononetin }

rn:R08663 <-- [Oxidized NADPH---hemoprotein reductase] --> rn:R08551 -- [Reduced NADPH---hemoprotein reductase] --> rn:R03615 { Flavonoid }

rn:R08663 <-- [Oxidized NADPH---hemoprotein reductase] --> rn:R08551 -- [Reduced NADPH---hemoprotein reductase] --> rn:R06560 { Formononetin }

rn:R08663 <-- [Oxidized NADPH---hemoprotein reductase] --> rn:R08551 -- [Reduced NADPH---hemoprotein reductase] --> rn:R07198 { Liquiritigenin }

rn:R08663 <-- [Oxidized NADPH---hemoprotein reductase] --> rn:R08551 -- [Reduced NADPH---hemoprotein reductase] --> rn:R07745 { Calycosin }

rn:R08663 <-- [Oxidized NADPH---hemoprotein reductase] --> rn:R08551 -- [Reduced NADPH---hemoprotein reductase] --> rn:R07777 { Liquiritigenin }

rn:R08663 <-- [Oxidized NADPH---hemoprotein reductase] --> rn:R08551 -- [Reduced NADPH---hemoprotein reductase] --> rn:R08002 { Liquiritigenin }

rn:R09403 <-- [Oxidized NADPH---hemoprotein reductase] --> rn:R08551 -- [Reduced NADPH---hemoprotein reductase] --> rn:R03006 { Formononetin }

rn:R09403 <-- [Oxidized NADPH---hemoprotein reductase] --> rn:R08551 -- [Reduced NADPH---hemoprotein reductase] --> rn:R03615 { Flavonoid }

rn:R09403 <-- [Oxidized NADPH---hemoprotein reductase] --> rn:R08551 -- [Reduced NADPH---hemoprotein reductase] --> rn:R06560 { Formononetin }

rn:R09403 <-- [Oxidized NADPH---hemoprotein reductase] --> rn:R08551 -- [Reduced NADPH---hemoprotein reductase] --> rn:R07198 { Liquiritigenin }

rn:R09403 <-- [Oxidized NADPH---hemoprotein reductase] --> rn:R08551 -- [Reduced NADPH---hemoprotein reductase] --> rn:R07745 { Calycosin }

rn:R09403 <-- [Oxidized NADPH---hemoprotein reductase] --> rn:R08551 -- [Reduced NADPH---hemoprotein reductase] --> rn:R07777 { Liquiritigenin }

rn:R09403 <-- [Oxidized NADPH---hemoprotein reductase] --> rn:R08551 -- [Reduced NADPH---hemoprotein reductase] --> rn:R08002 { Liquiritigenin }

rn:R09497 <-- Quinone --> rn:R12217 -- 2-Oxoglutarate --> rn:R07712 { Liquiritigenin }

rn:R09497 <-- Quinone --> rn:R12217 -- 2-Oxoglutarate --> rn:R07996 { Liquiritigenin }

rn:R09518 <-- Quinone --> rn:R12217 -- 2-Oxoglutarate --> rn:R07712 { Liquiritigenin }

rn:R09518 <-- Quinone --> rn:R12217 -- 2-Oxoglutarate --> rn:R07996 { Liquiritigenin }

rn:R09578 <-- [Oxidized NADPH---hemoprotein reductase] --> rn:R08551 -- [Reduced NADPH---hemoprotein reductase] --> rn:R03006 { Formononetin }

rn:R09578 <-- [Oxidized NADPH---hemoprotein reductase] --> rn:R08551 -- [Reduced NADPH---hemoprotein reductase] --> rn:R03615 { Flavonoid }

rn:R09578 <-- [Oxidized NADPH---hemoprotein reductase] --> rn:R08551 -- [Reduced NADPH---hemoprotein reductase] --> rn:R06560 { Formononetin }

rn:R09578 <-- [Oxidized NADPH---hemoprotein reductase] --> rn:R08551 -- [Reduced NADPH---hemoprotein reductase] --> rn:R07198 { Liquiritigenin }

rn:R09578 <-- [Oxidized NADPH---hemoprotein reductase] --> rn:R08551 -- [Reduced NADPH---hemoprotein reductase] --> rn:R07745 { Calycosin }

rn:R09578 <-- [Oxidized NADPH---hemoprotein reductase] --> rn:R08551 -- [Reduced NADPH---hemoprotein reductase] --> rn:R07777 { Liquiritigenin }

rn:R09578 <-- [Oxidized NADPH---hemoprotein reductase] --> rn:R08551 -- [Reduced NADPH---hemoprotein reductase] --> rn:R08002 { Liquiritigenin }

rn:R09579 <-- [Oxidized NADPH---hemoprotein reductase] --> rn:R08551 -- [Reduced NADPH---hemoprotein reductase] --> rn:R03006 { Formononetin }

rn:R09579 <-- [Oxidized NADPH---hemoprotein reductase] --> rn:R08551 -- [Reduced NADPH---hemoprotein reductase] --> rn:R03615 { Flavonoid }

rn:R09579 <-- [Oxidized NADPH---hemoprotein reductase] --> rn:R08551 -- [Reduced NADPH---hemoprotein reductase] --> rn:R06560 { Formononetin }

rn:R09579 <-- [Oxidized NADPH---hemoprotein reductase] --> rn:R08551 -- [Reduced NADPH---hemoprotein reductase] --> rn:R07198 { Liquiritigenin }

rn:R09579 <-- [Oxidized NADPH---hemoprotein reductase] --> rn:R08551 -- [Reduced NADPH---hemoprotein reductase] --> rn:R07745 { Calycosin }

rn:R09579 <-- [Oxidized NADPH---hemoprotein reductase] --> rn:R08551 -- [Reduced NADPH---hemoprotein reductase] --> rn:R07777 { Liquiritigenin }

rn:R09579 <-- [Oxidized NADPH---hemoprotein reductase] --> rn:R08551 -- [Reduced NADPH---hemoprotein reductase] --> rn:R08002 { Liquiritigenin }

rn:R09580 <-- [Oxidized NADPH---hemoprotein reductase] --> rn:R08551 -- [Reduced NADPH---hemoprotein reductase] --> rn:R03006 { Formononetin }

rn:R09580 <-- [Oxidized NADPH---hemoprotein reductase] --> rn:R08551 -- [Reduced NADPH---hemoprotein reductase] --> rn:R03615 { Flavonoid }

rn:R09580 <-- [Oxidized NADPH---hemoprotein reductase] --> rn:R08551 -- [Reduced NADPH---hemoprotein reductase] --> rn:R06560 { Formononetin }

rn:R09580 <-- [Oxidized NADPH---hemoprotein reductase] --> rn:R08551 -- [Reduced NADPH---hemoprotein reductase] --> rn:R07198 { Liquiritigenin }

rn:R09580 <-- [Oxidized NADPH---hemoprotein reductase] --> rn:R08551 -- [Reduced NADPH---hemoprotein reductase] --> rn:R07745 { Calycosin }

rn:R09580 <-- [Oxidized NADPH---hemoprotein reductase] --> rn:R08551 -- [Reduced NADPH---hemoprotein reductase] --> rn:R07777 { Liquiritigenin }

rn:R09580 <-- [Oxidized NADPH---hemoprotein reductase] --> rn:R08551 -- [Reduced NADPH---hemoprotein reductase] --> rn:R08002 { Liquiritigenin }

rn:R09598 <-- L-Glutamate --> rn:R00114 -- 2-Oxoglutarate --> rn:R07712 { Liquiritigenin }

rn:R09598 <-- L-Glutamate --> rn:R00114 -- 2-Oxoglutarate --> rn:R07996 { Liquiritigenin }

rn:R09598 <-- L-Glutamate --> rn:R00248 -- 2-Oxoglutarate --> rn:R07712 { Liquiritigenin }

rn:R09598 <-- L-Glutamate --> rn:R00248 -- 2-Oxoglutarate --> rn:R07996 { Liquiritigenin }

rn:R09598 <-- L-Glutamate --> rn:R04051 -- 2-Oxoglutarate --> rn:R07712 { Liquiritigenin }

rn:R09598 <-- L-Glutamate --> rn:R04051 -- 2-Oxoglutarate --> rn:R07996 { Liquiritigenin }

rn:R09599 <-- L-Glutamate --> rn:R00114 -- 2-Oxoglutarate --> rn:R07712 { Liquiritigenin }

rn:R09599 <-- L-Glutamate --> rn:R00114 -- 2-Oxoglutarate --> rn:R07996 { Liquiritigenin }

rn:R09599 <-- L-Glutamate --> rn:R00248 -- 2-Oxoglutarate --> rn:R07712 { Liquiritigenin }

rn:R09599 <-- L-Glutamate --> rn:R00248 -- 2-Oxoglutarate --> rn:R07996 { Liquiritigenin }

rn:R09599 <-- L-Glutamate --> rn:R04051 -- 2-Oxoglutarate --> rn:R07712 { Liquiritigenin }

rn:R09599 <-- L-Glutamate --> rn:R04051 -- 2-Oxoglutarate --> rn:R07996 { Liquiritigenin }

rn:R09824 <-- L-Glutamate --> rn:R00114 -- 2-Oxoglutarate --> rn:R07712 { Liquiritigenin }

rn:R09824 <-- L-Glutamate --> rn:R00114 -- 2-Oxoglutarate --> rn:R07996 { Liquiritigenin }

rn:R09824 <-- L-Glutamate --> rn:R00248 -- 2-Oxoglutarate --> rn:R07712 { Liquiritigenin }

rn:R09824 <-- L-Glutamate --> rn:R00248 -- 2-Oxoglutarate --> rn:R07996 { Liquiritigenin }

rn:R09824 <-- L-Glutamate --> rn:R04051 -- 2-Oxoglutarate --> rn:R07712 { Liquiritigenin }

rn:R09824 <-- L-Glutamate --> rn:R04051 -- 2-Oxoglutarate --> rn:R07996 { Liquiritigenin }

rn:R10027 <-- [Oxidized NADPH---hemoprotein reductase] --> rn:R08551 -- [Reduced NADPH---hemoprotein reductase] --> rn:R03006 { Formononetin }

rn:R10027 <-- [Oxidized NADPH---hemoprotein reductase] --> rn:R08551 -- [Reduced NADPH---hemoprotein reductase] --> rn:R03615 { Flavonoid }

rn:R10027 <-- [Oxidized NADPH---hemoprotein reductase] --> rn:R08551 -- [Reduced NADPH---hemoprotein reductase] --> rn:R06560 { Formononetin }

rn:R10027 <-- [Oxidized NADPH---hemoprotein reductase] --> rn:R08551 -- [Reduced NADPH---hemoprotein reductase] --> rn:R07198 { Liquiritigenin }

rn:R10027 <-- [Oxidized NADPH---hemoprotein reductase] --> rn:R08551 -- [Reduced NADPH---hemoprotein reductase] --> rn:R07745 { Calycosin }

rn:R10027 <-- [Oxidized NADPH---hemoprotein reductase] --> rn:R08551 -- [Reduced NADPH---hemoprotein reductase] --> rn:R07777 { Liquiritigenin }

rn:R10027 <-- [Oxidized NADPH---hemoprotein reductase] --> rn:R08551 -- [Reduced NADPH---hemoprotein reductase] --> rn:R08002 { Liquiritigenin }

rn:R10028 <-- [Oxidized NADPH---hemoprotein reductase] --> rn:R08551 -- [Reduced NADPH---hemoprotein reductase] --> rn:R03006 { Formononetin }

rn:R10028 <-- [Oxidized NADPH---hemoprotein reductase] --> rn:R08551 -- [Reduced NADPH---hemoprotein reductase] --> rn:R03615 { Flavonoid }

rn:R10028 <-- [Oxidized NADPH---hemoprotein reductase] --> rn:R08551 -- [Reduced NADPH---hemoprotein reductase] --> rn:R06560 { Formononetin }

rn:R10028 <-- [Oxidized NADPH---hemoprotein reductase] --> rn:R08551 -- [Reduced NADPH---hemoprotein reductase] --> rn:R07198 { Liquiritigenin }

rn:R10028 <-- [Oxidized NADPH---hemoprotein reductase] --> rn:R08551 -- [Reduced NADPH---hemoprotein reductase] --> rn:R07745 { Calycosin }

rn:R10028 <-- [Oxidized NADPH---hemoprotein reductase] --> rn:R08551 -- [Reduced NADPH---hemoprotein reductase] --> rn:R07777 { Liquiritigenin }

rn:R10028 <-- [Oxidized NADPH---hemoprotein reductase] --> rn:R08551 -- [Reduced NADPH---hemoprotein reductase] --> rn:R08002 { Liquiritigenin }

rn:R10031 <-- [Oxidized NADPH---hemoprotein reductase] --> rn:R08551 -- [Reduced NADPH---hemoprotein reductase] --> rn:R03006 { Formononetin }

rn:R10031 <-- [Oxidized NADPH---hemoprotein reductase] --> rn:R08551 -- [Reduced NADPH---hemoprotein reductase] --> rn:R03615 { Flavonoid }

rn:R10031 <-- [Oxidized NADPH---hemoprotein reductase] --> rn:R08551 -- [Reduced NADPH---hemoprotein reductase] --> rn:R06560 { Formononetin }

rn:R10031 <-- [Oxidized NADPH---hemoprotein reductase] --> rn:R08551 -- [Reduced NADPH---hemoprotein reductase] --> rn:R07198 { Liquiritigenin }

rn:R10031 <-- [Oxidized NADPH---hemoprotein reductase] --> rn:R08551 -- [Reduced NADPH---hemoprotein reductase] --> rn:R07745 { Calycosin }

rn:R10031 <-- [Oxidized NADPH---hemoprotein reductase] --> rn:R08551 -- [Reduced NADPH---hemoprotein reductase] --> rn:R07777 { Liquiritigenin }

rn:R10031 <-- [Oxidized NADPH---hemoprotein reductase] --> rn:R08551 -- [Reduced NADPH---hemoprotein reductase] --> rn:R08002 { Liquiritigenin }

rn:R10032 <-- [Oxidized NADPH---hemoprotein reductase] --> rn:R08551 -- [Reduced NADPH---hemoprotein reductase] --> rn:R03006 { Formononetin }

rn:R10032 <-- [Oxidized NADPH---hemoprotein reductase] --> rn:R08551 -- [Reduced NADPH---hemoprotein reductase] --> rn:R03615 { Flavonoid }

rn:R10032 <-- [Oxidized NADPH---hemoprotein reductase] --> rn:R08551 -- [Reduced NADPH---hemoprotein reductase] --> rn:R06560 { Formononetin }

rn:R10032 <-- [Oxidized NADPH---hemoprotein reductase] --> rn:R08551 -- [Reduced NADPH---hemoprotein reductase] --> rn:R07198 { Liquiritigenin }

rn:R10032 <-- [Oxidized NADPH---hemoprotein reductase] --> rn:R08551 -- [Reduced NADPH---hemoprotein reductase] --> rn:R07745 { Calycosin }

rn:R10032 <-- [Oxidized NADPH---hemoprotein reductase] --> rn:R08551 -- [Reduced NADPH---hemoprotein reductase] --> rn:R07777 { Liquiritigenin }

rn:R10032 <-- [Oxidized NADPH---hemoprotein reductase] --> rn:R08551 -- [Reduced NADPH---hemoprotein reductase] --> rn:R08002 { Liquiritigenin }

rn:R10572 <-- Quinone --> rn:R12217 -- 2-Oxoglutarate --> rn:R07712 { Liquiritigenin }

rn:R10572 <-- Quinone --> rn:R12217 -- 2-Oxoglutarate --> rn:R07996 { Liquiritigenin }

rn:R10671 <-- [Oxidized NADPH---hemoprotein reductase] --> rn:R08551 -- [Reduced NADPH---hemoprotein reductase] --> rn:R03006 { Formononetin }

rn:R10671 <-- [Oxidized NADPH---hemoprotein reductase] --> rn:R08551 -- [Reduced NADPH---hemoprotein reductase] --> rn:R03615 { Flavonoid }

rn:R10671 <-- [Oxidized NADPH---hemoprotein reductase] --> rn:R08551 -- [Reduced NADPH---hemoprotein reductase] --> rn:R06560 { Formononetin }

rn:R10671 <-- [Oxidized NADPH---hemoprotein reductase] --> rn:R08551 -- [Reduced NADPH---hemoprotein reductase] --> rn:R07198 { Liquiritigenin }

rn:R10671 <-- [Oxidized NADPH---hemoprotein reductase] --> rn:R08551 -- [Reduced NADPH---hemoprotein reductase] --> rn:R07745 { Calycosin }

rn:R10671 <-- [Oxidized NADPH---hemoprotein reductase] --> rn:R08551 -- [Reduced NADPH---hemoprotein reductase] --> rn:R07777 { Liquiritigenin }

rn:R10671 <-- [Oxidized NADPH---hemoprotein reductase] --> rn:R08551 -- [Reduced NADPH---hemoprotein reductase] --> rn:R08002 { Liquiritigenin }

rn:R10728 <-- [Oxidized NADPH---hemoprotein reductase] --> rn:R08551 -- [Reduced NADPH---hemoprotein reductase] --> rn:R03006 { Formononetin }

rn:R10728 <-- [Oxidized NADPH---hemoprotein reductase] --> rn:R08551 -- [Reduced NADPH---hemoprotein reductase] --> rn:R03615 { Flavonoid }

rn:R10728 <-- [Oxidized NADPH---hemoprotein reductase] --> rn:R08551 -- [Reduced NADPH---hemoprotein reductase] --> rn:R06560 { Formononetin }

rn:R10728 <-- [Oxidized NADPH---hemoprotein reductase] --> rn:R08551 -- [Reduced NADPH---hemoprotein reductase] --> rn:R07198 { Liquiritigenin }

rn:R10728 <-- [Oxidized NADPH---hemoprotein reductase] --> rn:R08551 -- [Reduced NADPH---hemoprotein reductase] --> rn:R07745 { Calycosin }

rn:R10728 <-- [Oxidized NADPH---hemoprotein reductase] --> rn:R08551 -- [Reduced NADPH---hemoprotein reductase] --> rn:R07777 { Liquiritigenin }

rn:R10728 <-- [Oxidized NADPH---hemoprotein reductase] --> rn:R08551 -- [Reduced NADPH---hemoprotein reductase] --> rn:R08002 { Liquiritigenin }

rn:R10795 <-- [Oxidized NADPH---hemoprotein reductase] --> rn:R08551 -- [Reduced NADPH---hemoprotein reductase] --> rn:R03006 { Formononetin }

rn:R10795 <-- [Oxidized NADPH---hemoprotein reductase] --> rn:R08551 -- [Reduced NADPH---hemoprotein reductase] --> rn:R03615 { Flavonoid }

rn:R10795 <-- [Oxidized NADPH---hemoprotein reductase] --> rn:R08551 -- [Reduced NADPH---hemoprotein reductase] --> rn:R06560 { Formononetin }

rn:R10795 <-- [Oxidized NADPH---hemoprotein reductase] --> rn:R08551 -- [Reduced NADPH---hemoprotein reductase] --> rn:R07198 { Liquiritigenin }

rn:R10795 <-- [Oxidized NADPH---hemoprotein reductase] --> rn:R08551 -- [Reduced NADPH---hemoprotein reductase] --> rn:R07745 { Calycosin }

rn:R10795 <-- [Oxidized NADPH---hemoprotein reductase] --> rn:R08551 -- [Reduced NADPH---hemoprotein reductase] --> rn:R07777 { Liquiritigenin }

rn:R10795 <-- [Oxidized NADPH---hemoprotein reductase] --> rn:R08551 -- [Reduced NADPH---hemoprotein reductase] --> rn:R08002 { Liquiritigenin }

rn:R10999 <-- [Oxidized NADPH---hemoprotein reductase] --> rn:R08551 -- [Reduced NADPH---hemoprotein reductase] --> rn:R03006 { Formononetin }

rn:R10999 <-- [Oxidized NADPH---hemoprotein reductase] --> rn:R08551 -- [Reduced NADPH---hemoprotein reductase] --> rn:R03615 { Flavonoid }

rn:R10999 <-- [Oxidized NADPH---hemoprotein reductase] --> rn:R08551 -- [Reduced NADPH---hemoprotein reductase] --> rn:R06560 { Formononetin }

rn:R10999 <-- [Oxidized NADPH---hemoprotein reductase] --> rn:R08551 -- [Reduced NADPH---hemoprotein reductase] --> rn:R07198 { Liquiritigenin }

rn:R10999 <-- [Oxidized NADPH---hemoprotein reductase] --> rn:R08551 -- [Reduced NADPH---hemoprotein reductase] --> rn:R07745 { Calycosin }

rn:R10999 <-- [Oxidized NADPH---hemoprotein reductase] --> rn:R08551 -- [Reduced NADPH---hemoprotein reductase] --> rn:R07777 { Liquiritigenin }

rn:R10999 <-- [Oxidized NADPH---hemoprotein reductase] --> rn:R08551 -- [Reduced NADPH---hemoprotein reductase] --> rn:R08002 { Liquiritigenin }

rn:R11000 <-- [Oxidized NADPH---hemoprotein reductase] --> rn:R08551 -- [Reduced NADPH---hemoprotein reductase] --> rn:R03006 { Formononetin }

rn:R11000 <-- [Oxidized NADPH---hemoprotein reductase] --> rn:R08551 -- [Reduced NADPH---hemoprotein reductase] --> rn:R03615 { Flavonoid }

rn:R11000 <-- [Oxidized NADPH---hemoprotein reductase] --> rn:R08551 -- [Reduced NADPH---hemoprotein reductase] --> rn:R06560 { Formononetin }

rn:R11000 <-- [Oxidized NADPH---hemoprotein reductase] --> rn:R08551 -- [Reduced NADPH---hemoprotein reductase] --> rn:R07198 { Liquiritigenin }

rn:R11000 <-- [Oxidized NADPH---hemoprotein reductase] --> rn:R08551 -- [Reduced NADPH---hemoprotein reductase] --> rn:R07745 { Calycosin }

rn:R11000 <-- [Oxidized NADPH---hemoprotein reductase] --> rn:R08551 -- [Reduced NADPH---hemoprotein reductase] --> rn:R07777 { Liquiritigenin }

rn:R11000 <-- [Oxidized NADPH---hemoprotein reductase] --> rn:R08551 -- [Reduced NADPH---hemoprotein reductase] --> rn:R08002 { Liquiritigenin }

rn:R11021 <-- L-Glutamate --> rn:R00114 -- 2-Oxoglutarate --> rn:R07712 { Liquiritigenin }

rn:R11021 <-- L-Glutamate --> rn:R00114 -- 2-Oxoglutarate --> rn:R07996 { Liquiritigenin }

rn:R11021 <-- L-Glutamate --> rn:R00248 -- 2-Oxoglutarate --> rn:R07712 { Liquiritigenin }

rn:R11021 <-- L-Glutamate --> rn:R00248 -- 2-Oxoglutarate --> rn:R07996 { Liquiritigenin }

rn:R11021 <-- L-Glutamate --> rn:R04051 -- 2-Oxoglutarate --> rn:R07712 { Liquiritigenin }

rn:R11021 <-- L-Glutamate --> rn:R04051 -- 2-Oxoglutarate --> rn:R07996 { Liquiritigenin }

rn:R11597 <-- [Oxidized NADPH---hemoprotein reductase] --> rn:R08551 -- [Reduced NADPH---hemoprotein reductase] --> rn:R03006 { Formononetin }

rn:R11597 <-- [Oxidized NADPH---hemoprotein reductase] --> rn:R08551 -- [Reduced NADPH---hemoprotein reductase] --> rn:R03615 { Flavonoid }

rn:R11597 <-- [Oxidized NADPH---hemoprotein reductase] --> rn:R08551 -- [Reduced NADPH---hemoprotein reductase] --> rn:R06560 { Formononetin }

rn:R11597 <-- [Oxidized NADPH---hemoprotein reductase] --> rn:R08551 -- [Reduced NADPH---hemoprotein reductase] --> rn:R07198 { Liquiritigenin }

rn:R11597 <-- [Oxidized NADPH---hemoprotein reductase] --> rn:R08551 -- [Reduced NADPH---hemoprotein reductase] --> rn:R07745 { Calycosin }

rn:R11597 <-- [Oxidized NADPH---hemoprotein reductase] --> rn:R08551 -- [Reduced NADPH---hemoprotein reductase] --> rn:R07777 { Liquiritigenin }

rn:R11597 <-- [Oxidized NADPH---hemoprotein reductase] --> rn:R08551 -- [Reduced NADPH---hemoprotein reductase] --> rn:R08002 { Liquiritigenin }

rn:R11598 <-- [Oxidized NADPH---hemoprotein reductase] --> rn:R08551 -- [Reduced NADPH---hemoprotein reductase] --> rn:R03006 { Formononetin }

rn:R11598 <-- [Oxidized NADPH---hemoprotein reductase] --> rn:R08551 -- [Reduced NADPH---hemoprotein reductase] --> rn:R03615 { Flavonoid }

rn:R11598 <-- [Oxidized NADPH---hemoprotein reductase] --> rn:R08551 -- [Reduced NADPH---hemoprotein reductase] --> rn:R06560 { Formononetin }

rn:R11598 <-- [Oxidized NADPH---hemoprotein reductase] --> rn:R08551 -- [Reduced NADPH---hemoprotein reductase] --> rn:R07198 { Liquiritigenin }

rn:R11598 <-- [Oxidized NADPH---hemoprotein reductase] --> rn:R08551 -- [Reduced NADPH---hemoprotein reductase] --> rn:R07745 { Calycosin }

rn:R11598 <-- [Oxidized NADPH---hemoprotein reductase] --> rn:R08551 -- [Reduced NADPH---hemoprotein reductase] --> rn:R07777 { Liquiritigenin }

rn:R11598 <-- [Oxidized NADPH---hemoprotein reductase] --> rn:R08551 -- [Reduced NADPH---hemoprotein reductase] --> rn:R08002 { Liquiritigenin }

rn:R11640 <-- [Oxidized NADPH---hemoprotein reductase] --> rn:R08551 -- [Reduced NADPH---hemoprotein reductase] --> rn:R03006 { Formononetin }

rn:R11640 <-- [Oxidized NADPH---hemoprotein reductase] --> rn:R08551 -- [Reduced NADPH---hemoprotein reductase] --> rn:R03615 { Flavonoid }

rn:R11640 <-- [Oxidized NADPH---hemoprotein reductase] --> rn:R08551 -- [Reduced NADPH---hemoprotein reductase] --> rn:R06560 { Formononetin }

rn:R11640 <-- [Oxidized NADPH---hemoprotein reductase] --> rn:R08551 -- [Reduced NADPH---hemoprotein reductase] --> rn:R07198 { Liquiritigenin }

rn:R11640 <-- [Oxidized NADPH---hemoprotein reductase] --> rn:R08551 -- [Reduced NADPH---hemoprotein reductase] --> rn:R07745 { Calycosin }

rn:R11640 <-- [Oxidized NADPH---hemoprotein reductase] --> rn:R08551 -- [Reduced NADPH---hemoprotein reductase] --> rn:R07777 { Liquiritigenin }

rn:R11640 <-- [Oxidized NADPH---hemoprotein reductase] --> rn:R08551 -- [Reduced NADPH---hemoprotein reductase] --> rn:R08002 { Liquiritigenin }

rn:R11642 <-- [Oxidized NADPH---hemoprotein reductase] --> rn:R08551 -- [Reduced NADPH---hemoprotein reductase] --> rn:R03006 { Formononetin }

rn:R11642 <-- [Oxidized NADPH---hemoprotein reductase] --> rn:R08551 -- [Reduced NADPH---hemoprotein reductase] --> rn:R03615 { Flavonoid }

rn:R11642 <-- [Oxidized NADPH---hemoprotein reductase] --> rn:R08551 -- [Reduced NADPH---hemoprotein reductase] --> rn:R06560 { Formononetin }

rn:R11642 <-- [Oxidized NADPH---hemoprotein reductase] --> rn:R08551 -- [Reduced NADPH---hemoprotein reductase] --> rn:R07198 { Liquiritigenin }

rn:R11642 <-- [Oxidized NADPH---hemoprotein reductase] --> rn:R08551 -- [Reduced NADPH---hemoprotein reductase] --> rn:R07745 { Calycosin }

rn:R11642 <-- [Oxidized NADPH---hemoprotein reductase] --> rn:R08551 -- [Reduced NADPH---hemoprotein reductase] --> rn:R07777 { Liquiritigenin }

rn:R11642 <-- [Oxidized NADPH---hemoprotein reductase] --> rn:R08551 -- [Reduced NADPH---hemoprotein reductase] --> rn:R08002 { Liquiritigenin }

rn:R11732 <-- [Oxidized NADPH---hemoprotein reductase] --> rn:R08551 -- [Reduced NADPH---hemoprotein reductase] --> rn:R03006 { Formononetin }

rn:R11732 <-- [Oxidized NADPH---hemoprotein reductase] --> rn:R08551 -- [Reduced NADPH---hemoprotein reductase] --> rn:R03615 { Flavonoid }

rn:R11732 <-- [Oxidized NADPH---hemoprotein reductase] --> rn:R08551 -- [Reduced NADPH---hemoprotein reductase] --> rn:R06560 { Formononetin }

rn:R11732 <-- [Oxidized NADPH---hemoprotein reductase] --> rn:R08551 -- [Reduced NADPH---hemoprotein reductase] --> rn:R07198 { Liquiritigenin }

rn:R11732 <-- [Oxidized NADPH---hemoprotein reductase] --> rn:R08551 -- [Reduced NADPH---hemoprotein reductase] --> rn:R07745 { Calycosin }

rn:R11732 <-- [Oxidized NADPH---hemoprotein reductase] --> rn:R08551 -- [Reduced NADPH---hemoprotein reductase] --> rn:R07777 { Liquiritigenin }

rn:R11732 <-- [Oxidized NADPH---hemoprotein reductase] --> rn:R08551 -- [Reduced NADPH---hemoprotein reductase] --> rn:R08002 { Liquiritigenin }

rn:R11733 <-- [Oxidized NADPH---hemoprotein reductase] --> rn:R08551 -- [Reduced NADPH---hemoprotein reductase] --> rn:R03006 { Formononetin }

rn:R11733 <-- [Oxidized NADPH---hemoprotein reductase] --> rn:R08551 -- [Reduced NADPH---hemoprotein reductase] --> rn:R03615 { Flavonoid }

rn:R11733 <-- [Oxidized NADPH---hemoprotein reductase] --> rn:R08551 -- [Reduced NADPH---hemoprotein reductase] --> rn:R06560 { Formononetin }

rn:R11733 <-- [Oxidized NADPH---hemoprotein reductase] --> rn:R08551 -- [Reduced NADPH---hemoprotein reductase] --> rn:R07198 { Liquiritigenin }

rn:R11733 <-- [Oxidized NADPH---hemoprotein reductase] --> rn:R08551 -- [Reduced NADPH---hemoprotein reductase] --> rn:R07745 { Calycosin }

rn:R11733 <-- [Oxidized NADPH---hemoprotein reductase] --> rn:R08551 -- [Reduced NADPH---hemoprotein reductase] --> rn:R07777 { Liquiritigenin }

rn:R11733 <-- [Oxidized NADPH---hemoprotein reductase] --> rn:R08551 -- [Reduced NADPH---hemoprotein reductase] --> rn:R08002 { Liquiritigenin }

rn:R11737 <-- [Oxidized NADPH---hemoprotein reductase] --> rn:R08551 -- [Reduced NADPH---hemoprotein reductase] --> rn:R03006 { Formononetin }

rn:R11737 <-- [Oxidized NADPH---hemoprotein reductase] --> rn:R08551 -- [Reduced NADPH---hemoprotein reductase] --> rn:R03615 { Flavonoid }

rn:R11737 <-- [Oxidized NADPH---hemoprotein reductase] --> rn:R08551 -- [Reduced NADPH---hemoprotein reductase] --> rn:R06560 { Formononetin }

rn:R11737 <-- [Oxidized NADPH---hemoprotein reductase] --> rn:R08551 -- [Reduced NADPH---hemoprotein reductase] --> rn:R07198 { Liquiritigenin }

rn:R11737 <-- [Oxidized NADPH---hemoprotein reductase] --> rn:R08551 -- [Reduced NADPH---hemoprotein reductase] --> rn:R07745 { Calycosin }

rn:R11737 <-- [Oxidized NADPH---hemoprotein reductase] --> rn:R08551 -- [Reduced NADPH---hemoprotein reductase] --> rn:R07777 { Liquiritigenin }

rn:R11737 <-- [Oxidized NADPH---hemoprotein reductase] --> rn:R08551 -- [Reduced NADPH---hemoprotein reductase] --> rn:R08002 { Liquiritigenin }

rn:R11738 <-- [Oxidized NADPH---hemoprotein reductase] --> rn:R08551 -- [Reduced NADPH---hemoprotein reductase] --> rn:R03006 { Formononetin }

rn:R11738 <-- [Oxidized NADPH---hemoprotein reductase] --> rn:R08551 -- [Reduced NADPH---hemoprotein reductase] --> rn:R03615 { Flavonoid }

rn:R11738 <-- [Oxidized NADPH---hemoprotein reductase] --> rn:R08551 -- [Reduced NADPH---hemoprotein reductase] --> rn:R06560 { Formononetin }

rn:R11738 <-- [Oxidized NADPH---hemoprotein reductase] --> rn:R08551 -- [Reduced NADPH---hemoprotein reductase] --> rn:R07198 { Liquiritigenin }

rn:R11738 <-- [Oxidized NADPH---hemoprotein reductase] --> rn:R08551 -- [Reduced NADPH---hemoprotein reductase] --> rn:R07745 { Calycosin }

rn:R11738 <-- [Oxidized NADPH---hemoprotein reductase] --> rn:R08551 -- [Reduced NADPH---hemoprotein reductase] --> rn:R07777 { Liquiritigenin }

rn:R11738 <-- [Oxidized NADPH---hemoprotein reductase] --> rn:R08551 -- [Reduced NADPH---hemoprotein reductase] --> rn:R08002 { Liquiritigenin }

rn:R11813 <-- [Oxidized NADPH---hemoprotein reductase] --> rn:R08551 -- [Reduced NADPH---hemoprotein reductase] --> rn:R03006 { Formononetin }

rn:R11813 <-- [Oxidized NADPH---hemoprotein reductase] --> rn:R08551 -- [Reduced NADPH---hemoprotein reductase] --> rn:R03615 { Flavonoid }

rn:R11813 <-- [Oxidized NADPH---hemoprotein reductase] --> rn:R08551 -- [Reduced NADPH---hemoprotein reductase] --> rn:R06560 { Formononetin }

rn:R11813 <-- [Oxidized NADPH---hemoprotein reductase] --> rn:R08551 -- [Reduced NADPH---hemoprotein reductase] --> rn:R07198 { Liquiritigenin }

rn:R11813 <-- [Oxidized NADPH---hemoprotein reductase] --> rn:R08551 -- [Reduced NADPH---hemoprotein reductase] --> rn:R07745 { Calycosin }

rn:R11813 <-- [Oxidized NADPH---hemoprotein reductase] --> rn:R08551 -- [Reduced NADPH---hemoprotein reductase] --> rn:R07777 { Liquiritigenin }

rn:R11813 <-- [Oxidized NADPH---hemoprotein reductase] --> rn:R08551 -- [Reduced NADPH---hemoprotein reductase] --> rn:R08002 { Liquiritigenin }

rn:R11814 <-- [Oxidized NADPH---hemoprotein reductase] --> rn:R08551 -- [Reduced NADPH---hemoprotein reductase] --> rn:R03006 { Formononetin }

rn:R11814 <-- [Oxidized NADPH---hemoprotein reductase] --> rn:R08551 -- [Reduced NADPH---hemoprotein reductase] --> rn:R03615 { Flavonoid }

rn:R11814 <-- [Oxidized NADPH---hemoprotein reductase] --> rn:R08551 -- [Reduced NADPH---hemoprotein reductase] --> rn:R06560 { Formononetin }

rn:R11814 <-- [Oxidized NADPH---hemoprotein reductase] --> rn:R08551 -- [Reduced NADPH---hemoprotein reductase] --> rn:R07198 { Liquiritigenin }

rn:R11814 <-- [Oxidized NADPH---hemoprotein reductase] --> rn:R08551 -- [Reduced NADPH---hemoprotein reductase] --> rn:R07745 { Calycosin }

rn:R11814 <-- [Oxidized NADPH---hemoprotein reductase] --> rn:R08551 -- [Reduced NADPH---hemoprotein reductase] --> rn:R07777 { Liquiritigenin }

rn:R11814 <-- [Oxidized NADPH---hemoprotein reductase] --> rn:R08551 -- [Reduced NADPH---hemoprotein reductase] --> rn:R08002 { Liquiritigenin }

rn:R12072 <-- [Oxidized NADPH---hemoprotein reductase] --> rn:R08551 -- [Reduced NADPH---hemoprotein reductase] --> rn:R03006 { Formononetin }

rn:R12072 <-- [Oxidized NADPH---hemoprotein reductase] --> rn:R08551 -- [Reduced NADPH---hemoprotein reductase] --> rn:R03615 { Flavonoid }

rn:R12072 <-- [Oxidized NADPH---hemoprotein reductase] --> rn:R08551 -- [Reduced NADPH---hemoprotein reductase] --> rn:R06560 { Formononetin }

rn:R12072 <-- [Oxidized NADPH---hemoprotein reductase] --> rn:R08551 -- [Reduced NADPH---hemoprotein reductase] --> rn:R07198 { Liquiritigenin }

rn:R12072 <-- [Oxidized NADPH---hemoprotein reductase] --> rn:R08551 -- [Reduced NADPH---hemoprotein reductase] --> rn:R07745 { Calycosin }

rn:R12072 <-- [Oxidized NADPH---hemoprotein reductase] --> rn:R08551 -- [Reduced NADPH---hemoprotein reductase] --> rn:R07777 { Liquiritigenin }

rn:R12072 <-- [Oxidized NADPH---hemoprotein reductase] --> rn:R08551 -- [Reduced NADPH---hemoprotein reductase] --> rn:R08002 { Liquiritigenin }

rn:R12183 <-- [Oxidized NADPH---hemoprotein reductase] --> rn:R08551 -- [Reduced NADPH---hemoprotein reductase] --> rn:R03006 { Formononetin }

rn:R12183 <-- [Oxidized NADPH---hemoprotein reductase] --> rn:R08551 -- [Reduced NADPH---hemoprotein reductase] --> rn:R03615 { Flavonoid }

rn:R12183 <-- [Oxidized NADPH---hemoprotein reductase] --> rn:R08551 -- [Reduced NADPH---hemoprotein reductase] --> rn:R06560 { Formononetin }

rn:R12183 <-- [Oxidized NADPH---hemoprotein reductase] --> rn:R08551 -- [Reduced NADPH---hemoprotein reductase] --> rn:R07198 { Liquiritigenin }

rn:R12183 <-- [Oxidized NADPH---hemoprotein reductase] --> rn:R08551 -- [Reduced NADPH---hemoprotein reductase] --> rn:R07745 { Calycosin }

rn:R12183 <-- [Oxidized NADPH---hemoprotein reductase] --> rn:R08551 -- [Reduced NADPH---hemoprotein reductase] --> rn:R07777 { Liquiritigenin }

rn:R12183 <-- [Oxidized NADPH---hemoprotein reductase] --> rn:R08551 -- [Reduced NADPH---hemoprotein reductase] --> rn:R08002 { Liquiritigenin }

rn:R12184 <-- [Oxidized NADPH---hemoprotein reductase] --> rn:R08551 -- [Reduced NADPH---hemoprotein reductase] --> rn:R03006 { Formononetin }

rn:R12184 <-- [Oxidized NADPH---hemoprotein reductase] --> rn:R08551 -- [Reduced NADPH---hemoprotein reductase] --> rn:R03615 { Flavonoid }

rn:R12184 <-- [Oxidized NADPH---hemoprotein reductase] --> rn:R08551 -- [Reduced NADPH---hemoprotein reductase] --> rn:R06560 { Formononetin }

rn:R12184 <-- [Oxidized NADPH---hemoprotein reductase] --> rn:R08551 -- [Reduced NADPH---hemoprotein reductase] --> rn:R07198 { Liquiritigenin }

rn:R12184 <-- [Oxidized NADPH---hemoprotein reductase] --> rn:R08551 -- [Reduced NADPH---hemoprotein reductase] --> rn:R07745 { Calycosin }

rn:R12184 <-- [Oxidized NADPH---hemoprotein reductase] --> rn:R08551 -- [Reduced NADPH---hemoprotein reductase] --> rn:R07777 { Liquiritigenin }

rn:R12184 <-- [Oxidized NADPH---hemoprotein reductase] --> rn:R08551 -- [Reduced NADPH---hemoprotein reductase] --> rn:R08002 { Liquiritigenin }

rn:R12216 <-- (S)-2-Hydroxyglutarate --> rn:R12217 -- 2-Oxoglutarate --> rn:R07712 { Liquiritigenin }

rn:R12216 <-- (S)-2-Hydroxyglutarate --> rn:R12217 -- 2-Oxoglutarate --> rn:R07996 { Liquiritigenin }
