# Supplementary material for: The impact of Traditional Chinese Medicine on mouse gut microbiota abundances and interactions based on Granger causality and pathway analysis
Source: Front Microbiol. 2022 Nov 11;13:980082. doi: 10.3389/fmicb.2022.980082 (PMC9692106; doi:10.3389/fmicb.2022.980082)
Supplement: Supplementary file 10 [file Table_10.docx]

**15 Specific equations for *cassia twig* and *B.pseudolongum*：**

rn:R01183

myo-Inositol + NAD+ <=> 2,4,6/3,5-Pentahydroxycyclohexanone + NADH + H+

rn:R01704

Hexadecanal + NAD+ + H2O <=> Hexadecanoic acid + NADH + H+

rn:R02781

L-Glutamine + 2,4,6/3,5-Pentahydroxycyclohexanone <=> 2-Oxoglutaramate + 1-Amino-1-deoxy-scyllo-inositol

rn:R02782

2,4,6/3,5-Pentahydroxycyclohexanone <=> 3D-3,5/4-Trihydroxycyclohexane-1,2-dione + H2O

rn:R05136

Salicylaldehyde + Pyruvate <=> trans-o-Hydroxybenzylidenepyruvate + H2O

rn:R05659

2,4,6/3,5-Pentahydroxycyclohexanone <=> D-2,3-Diketo 4-deoxy-epi-inositol + H2O

rn:R06913

2-Hydroxy-3-methylbenzalpyruvate + H2O <=> 3-Methylsalicylaldehyde + Pyruvate

rn:R06914

3-Methylsalicylaldehyde + NAD+ + H2O <=> 3-Methylsalicylate + NADH + H+

rn:R06934

2-Hydroxy-4-hydroxymethylbenzalpyruvate + H2O <=> 4-Hydroxymethylsalicylaldehyde + Pyruvate

rn:R06935

4-Hydroxymethylsalicylaldehyde + NAD+ + H2O <=> 4-Hydroxymethylsalicylate + NADH + H+

rn:R08106

1-Fluorocyclohexadiene-cis,cis-1,2-diol <=> Catechol + Hydrofluoric acid

rn:R08122

2-Fluorocyclohexadiene-cis,cis-1,2-diol-1-carboxylate <=> Catechol

rn:R09952

1-Keto-D-chiro-inositol <=> 2,4,6/3,5-Pentahydroxycyclohexanone

rn:R09953

scyllo-Inositol + NAD+ <=> 2,4,6/3,5-Pentahydroxycyclohexanone + NADH + H+

rn:R11770

2,4,6/3,5-Pentahydroxycyclohexanone + NAD+ <=> 3-Dehydro-scyllo-inosose + NADH + H+

**5 Specific equations for *cassia twig* and *A. equolifaciens*：**

rn:R02675

4-Cresol + 4 Oxidized azurin + H2O <=> 4-Hydroxybenzaldehyde + 4 Reduced azurin + 4 H+

rn:R05354

Styrene-cis-2,3-dihydrodiol + NAD+ <=> 3-Vinylcatechol + NADH + H+

rn:R05417

Styrene + Oxygen + NADH + H+ <=> Styrene-cis-2,3-dihydrodiol + NAD+

rn:R05424

Ethylbenzene <=> Styrene + 2 H+

rn:R05488

Styrene + FADH2 + Oxygen <=> (S)-2-Phenyloxirane + FAD + H2O

**75 shared equations by *cassia twig* ~ *B.pseudolongum* and *cassia twig* ~*A. equolifaciens*：**

rn:R00043

1,2-Bis(4-hydroxy-3-methoxyphenyl)ethylene + Oxygen <=> 2 4-Hydroxy-3-methoxy-benzaldehyde

rn:R01241

Phenol + Acetate <=> Phenyl acetate + H2O

rn:R01293

4-Hydroxybenzaldehyde + NAD+ + H2O <=> 4-Hydroxybenzoate + NADH + H+

rn:R01294

4-Hydroxybenzaldehyde + NADP+ + H2O <=> 4-Hydroxybenzoate + NADPH + H+

rn:R01303

4-Hydroxybenzoate + Acceptor + H2O <=> 4-Hydroxybenzaldehyde + Reduced acceptor + Oxygen

rn:R01372

Phenylpyruvate + Oxygen <=> 2-hydroxyphenylacetate + CO2

rn:R01419

Benzaldehyde + NAD+ + H2O <=> Benzoate + NADH + H+

rn:R01420

Benzaldehyde + NADP+ + H2O <=> Benzoate + NADPH + H+

rn:R01764

alpha-Oxo-benzeneacetic acid <=> Benzaldehyde + CO2

rn:R01767

Mandelonitrile <=> Hydrogen cyanide + Benzaldehyde

rn:R02177

(-)-Menthol + NADP+ <=> (-)-Menthone + NADPH + H+

rn:R02178

(-)-Menthol + Oxygen + NADPH + H+ <=> p-Menthane-3,8-diol + NADP+ + H2O

rn:R02231

Cyclohexanone + Oxygen + NADPH + H+ <=> epsilon-Caprolactone + NADP+ + H2O

rn:R02338

Indole + Oxygen <=> 2-Formylaminobenzaldehyde

rn:R02506

Cinnamaldehyde + CoA + NADP+ <=> Cinnamoyl-CoA + NADPH + H+

rn:R02548

(+)-Neomenthol + NADP+ <=> (-)-Menthone + NADPH + H+

rn:R02615

styrene oxide <=> Phenylacetaldehyde

rn:R02672

4-Hydroxyphenylglyoxylate <=> 4-Hydroxybenzaldehyde + CO2

rn:R02673

(S)-4-Hydroxymandelate + Oxygen <=> 4-Hydroxybenzaldehyde + Hydrogen peroxide + CO2

rn:R02676

(S)-4-Hydroxymandelonitrile <=> Hydrogen cyanide + 4-Hydroxybenzaldehyde

rn:R02877

Vanillyl alcohol + Oxygen <=> 4-Hydroxy-3-methoxy-benzaldehyde + Hydrogen peroxide

rn:R02941

Salicylaldehyde + NAD+ + H2O <=> Salicylate + NADH + H+

rn:R02995

6-Oxocineole + Oxygen + NADPH + H+ <=> 1,6,6-Trimethyl-2,7-dioxabicyclo[3.2.2]nonan-3-one + NADP+ + H2O

rn:R03054

Cinnamaldehyde + NADPH + H+ <=> Cinnamyl alcohol + NADP+

rn:R03369

3-(2-hydroxyphenyl)propanoate + Oxygen + NADH + H+ <=> 3-(2,3-Dihydroxyphenyl)propanoate + NAD+ + H2O

rn:R03498

D-Pinitol + NADP+ <=> 2D-5-O-Methyl-2,3,5/4,6-pentahydroxycyclohexanone + NADPH + H+

rn:R03692

Dihydrocoumarin + H2O <=> 3-(2-hydroxyphenyl)propanoate

rn:R03709

3-(2-hydroxyphenyl)propanoate + NAD+ <=> trans-2-Hydroxycinnamate + NADH + H+

rn:R04136

3-Hydroxybenzyl alcohol + NADP+ <=> 3-Hydroxybenzaldehyde + NADPH + H+

rn:R04899

3-(2-hydroxyphenyl)propanoate <=> Phenylpropanoate

rn:R05001

2-hydroxyphenylacetate <=> 2,6-Dihydroxyphenylacetate

rn:R05273

Vanillate + NAD+ + H2O <=> 4-Hydroxy-3-methoxy-benzaldehyde + Oxygen + NADH + H+

rn:R05360

2-Hydroxy-6-oxo-6-(2-hydroxyphenyl)-hexa-2,4-dienoate + H2O <=> Salicylate + 2-Hydroxy-2,4-pentadienoate

rn:R05432

2 Indanone + Oxygen + 2 NADPH + 2 H+ <=> 2 Dihydrocoumarin + 2 NADP+

rn:R05450

2-hydroxyphenylacetate + Oxygen + NADH + H+ <=> Homogentisate + NAD+ + H2O

rn:R05487

Phenylacetic acid + [Reduced NADPH---hemoprotein reductase] + Oxygen <=> 2-hydroxyphenylacetate + [Oxidized NADPH---hemoprotein reductase] + H2O

rn:R05537

3-(2-hydroxyphenyl)propanoate + ATP + CoA + FAD + NAD+ <=> Salicylate + AMP + Diphosphate + FADH2 + Acetyl-CoA + NADH + H+

rn:R05663

2-Methylbenzaldehyde + NADP+ + H2O <=> o-Toluate + NADPH + H+

rn:R05664

3-Methylbenzaldehyde + NADP+ + H2O <=> m-Methylbenzoate + NADPH + H+

rn:R05699

4-Hydroxy-3-methoxy-benzaldehyde + NAD+ + H2O <=> Vanillate + NADH + H+

rn:R05773

4-Hydroxy-3-methoxyphenyl-beta-hydroxypropanoyl-CoA <=> 4-Hydroxy-3-methoxy-benzaldehyde + Acetyl-CoA

rn:R06400

(1S,4R)-1-Hydroxy-2-oxoLimonene + NADPH + Oxygen + H+ <=> (4R)-7-Hydroxy-4-isopropenyl-7-methyl-2-oxo-oxepanone + NADP+ + H2O

rn:R06761

3-Dimethylallyl-beta-hydroxy-L-tyrosyl-[pcp] <=> 3-Dimethylallyl-4-hydroxybenzaldehyde

rn:R06762

3-Dimethylallyl-4-hydroxyphenylpyruvate <=> 3-Dimethylallyl-4-hydroxybenzaldehyde

rn:R06763

3-Dimethylallyl-4-hydroxybenzaldehyde <=> 3-Dimethylallyl-4-hydroxybenzoate

rn:R06765

2-Hydroxy-beta-keto-L-tyrosyl-[pcp] <=> 3-Amino-4,7-dihydroxycoumarin

rn:R06766

3-Amino-4,7-dihydroxycoumarin <=> 3-Amino-4,7-dihydroxy-8-chlorocoumarin

rn:R06776

3-Amino-4,7-dihydroxy-8-chlorocoumarin + 3-Dimethylallyl-4-hydroxybenzoate <=> Chlorobiocic acid

rn:R06777

3-Methylpyrrole-2,4-dicarboxylic acid + 3-Amino-4,7-dihydroxycoumarin + ATP <=> 5-[[(4,7-Dihydroxy-2-oxo-2H-1-benzopyran-3-yl)amino]carbonyl]-4-methyl-1H-pyrrole-3-carboxylate + AMP + Diphosphate

rn:R06778

5-[[(4,7-Dihydroxy-2-oxo-2H-1-benzopyran-3-yl)amino]carbonyl]-4-methyl-1H-pyrrole-3-carboxylate + 3-Amino-4,7-dihydroxycoumarin + ATP + 2 S-Adenosyl-L-methionine <=> Coumermic acid + AMP + Diphosphate + 2 S-Adenosyl-L-homocysteine

rn:R06783

trans-Cinnamate + Oxygen + NADH + H+ <=> cis-3-(3-Carboxyethenyl)-3,5-cyclohexadiene-1,2-diol + NAD+

rn:R06785

cis-3-(3-Carboxyethenyl)-3,5-cyclohexadiene-1,2-diol + NAD+ <=> trans-2,3-Dihydroxycinnamate + NADH + H+

rn:R06892

4'-Hydroxyacetophenone + NADPH + H+ + Oxygen <=> 4-Hydroxyphenyl acetate + NADP+ + H2O

rn:R06893

4-Hydroxyphenyl acetate + H2O <=> Hydroquinone + Acetate

rn:R07342

Phenyl acetate + H2O <=> Phenol + Acetate

rn:R07666

3-Hydroxybenzaldehyde + NADP+ + H2O <=> 3-Hydroxybenzoate + NADPH + H+

rn:R08083

Nerol + NADP+ <=> cis-Citral + NADPH + H+

rn:R08165

Isochorismate + 2-Oxoglutarate <=> 2-Succinyl-5-enolpyruvyl-6-hydroxy-3-cyclohexene-1-carboxylate + CO2

rn:R08166

2-Succinyl-5-enolpyruvyl-6-hydroxy-3-cyclohexene-1-carboxylate <=> (1R,6R)-6-Hydroxy-2-succinylcyclohexa-2,4-diene-1-carboxylate + Pyruvate

rn:R08176

Oleoyl-CoA + H2O <=> CoA + (9Z)-Octadecenoic acid

rn:R08447

Dopamine + 4-Hydroxydihydrocinnamaldehyde <=> (S)-Autumnaline

rn:R08461

4-Hydroxy-3-methoxy-benzaldehyde + 4-Aminobutanoate <=> Vanillylamine + Succinate semialdehyde

rn:R09277

4-[2-(5-Carboxy-2-hydroxy-3-methoxyphenyl)-2-oxoethylidene]-2-hydroxy-2-pentenedioate + H2O <=> 5-Carboxyvanillic acid + 4-Methylene-2-oxoglutarate

rn:R09389

(-)-Limonene + NADPH + Oxygen + H+ <=> (4S)-Limonene-1,2-epoxide + NADP+ + H2O

rn:R09390

(1R,4S)-1-Hydroxy-2-oxoLimonene + NADPH + Oxygen + H+ <=> (4S)-7-Hydroxy-4-isopropenyl-7-methyl-2-oxo-oxepanone + NADP+ + H2O

rn:R09393

d-Limonene + NADPH + Oxygen + H+ <=> Limonene-1,2-epoxide + NADP+ + H2O

rn:R09453

cis-9,10-Epoxystearic acid + NADPH + Oxygen + H+ <=> 9,10-Epoxy-18-hydroxystearate + NADP+ + H2O

rn:R09460

16-Hydroxypalmitate + NADPH + Oxygen + H+ <=> 10,16-Dihydroxyhexadecanoic acid + NADP+ + H2O

rn:R09954

scyllo-Inositol + NADP+ <=> 2,4,6/3,5-Pentahydroxycyclohexanone + NADPH + H+

rn:R10453

ATP + 3-Dimethylallyl-4-hydroxybenzoate + 3-Amino-4,7-dihydroxycoumarin <=> AMP + Diphosphate + 8-Demethylnovobiocic acid

rn:R11068

3-[(1R,2S,5R,6S)-5-Hydroxy-7-oxabicyclo[4.1.0]heptan-2-yl]-2-oxopropanoate + L-Phenylalanine <=> L-Dihydroanticapsin + Phenylpyruvate

rn:R11070

trans-Cinnamate <=> styrene + CO2

rn:R11195

4-Hydroxy-3-methylbenzaldehyde + NADP+ + H2O <=> 4-Hydroxy-3-methylbenzoate + NADPH + H+

rn:R11503

1-(4-Hydroxy-3-methoxyphenyl)-2-(2-methoxyphenoxy)propane-1,3-diol + Hydrogen peroxide <=> 4-Hydroxy-3-methoxy-benzaldehyde + o-Methoxyphenol + Glycolaldehyde + H2O

rn:R12141

9-Hydroxy-12-oxo-10(E),15(Z)-octadecadienoic acid + NADPH + H+ <=> 9-Hydroxy-12-oxo-15(Z)-octadecenoic acid + NADP+

**11 shared equations by** Decoction of Four Mild Drugs **~*B. pseudolongum* and** Decoction of Four Mild Drugs **~*A. equolifaciens***

rn:R03692

Dihydrocoumarin + H2O <=> 3-(2-Hydroxyphenyl)propanoate

rn:R05432

2 Indanone + Oxygen + 2 NADPH + 2 H+ <=> 2 Dihydrocoumarin + 2 NADP+

rn:R06562

2'-HydroxyFormononetin + NADPH + H+ <=> (-)-Vestitone + NADP+

rn:R06568

p-Coumaroyl-CoA + 3 Malonyl-CoA + NADPH + H+ <=> Isoliquiritigenin + 4 CoA + 3 CO2 + NADP+ + H2O

rn:R06765

2-Hydroxy-beta-keto-L-tyrosyl-[pcp] <=> 3-Amino-4,7-dihydroxycoumarin

rn:R06766

3-Amino-4,7-dihydroxycoumarin <=> 3-Amino-4,7-dihydroxy-8-chlorocoumarin

rn:R06776

3-Amino-4,7-dihydroxy-8-chlorocoumarin + 3-Dimethylallyl-4-hydroxybenzoate <=> Chlorobiocic acid

rn:R06777

3-Methylpyrrole-2,4-dicarboxylic acid + 3-Amino-4,7-dihydroxycoumarin + ATP <=> 5-[[(4,7-Dihydroxy-2-oxo-2H-1-benzopyran-3-yl)amino]carbonyl]-4-methyl-1H-pyrrole-3-carboxylate + AMP + Diphosphate

rn:R06778

5-[[(4,7-Dihydroxy-2-oxo-2H-1-benzopyran-3-yl)amino]carbonyl]-4-methyl-1H-pyrrole-3-carboxylate + 3-Amino-4,7-dihydroxycoumarin + ATP + 2 S-Adenosyl-L-methionine <=> Coumermic acid + AMP + Diphosphate + 2 S-Adenosyl-L-homocysteine

rn:R07711

Liquiritigenin + NADPH + H+ + Oxygen <=> 6,7,4'-Trihydroxyflavanone + NADP+ + H2O

rn:R10453

ATP + 3-Dimethylallyl-4-hydroxybenzoate + 3-Amino-4,7-dihydroxycoumarin <=> AMP + Diphosphate + 8-Demethylnovobiocic acid
