# Supplementary material for: The impact of Traditional Chinese Medicine on mouse gut microbiota abundances and interactions based on Granger causality and pathway analysis
Source: Front Microbiol. 2022 Nov 11;13:980082. doi: 10.3389/fmicb.2022.980082 (PMC9692106; doi:10.3389/fmicb.2022.980082)
Supplement: Supplementary file 12 [file Data_Sheet_2.PDF]

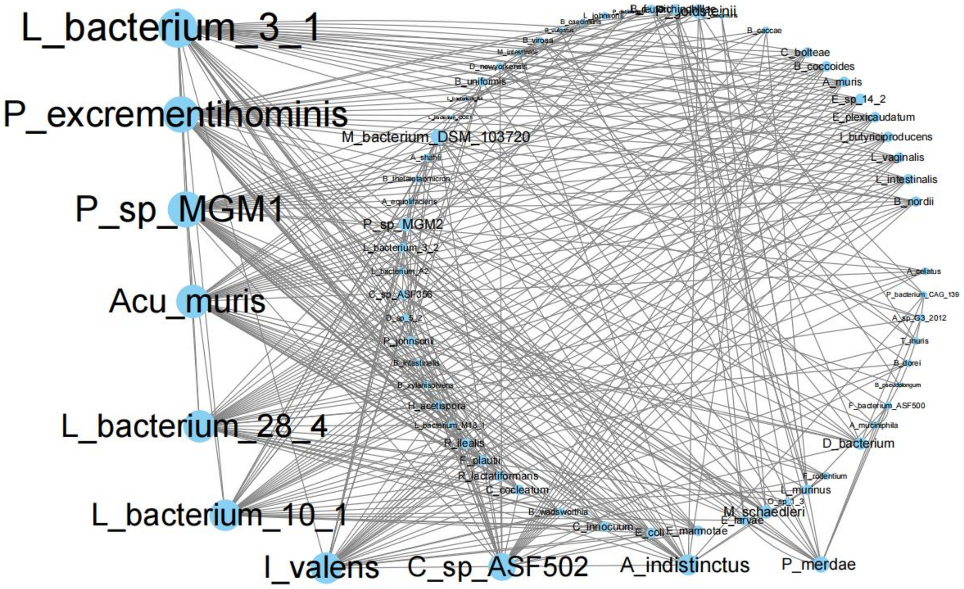

FigS1: Granger Causality network for mouse gut microorganisms in blank group with $p<10^{-4}$

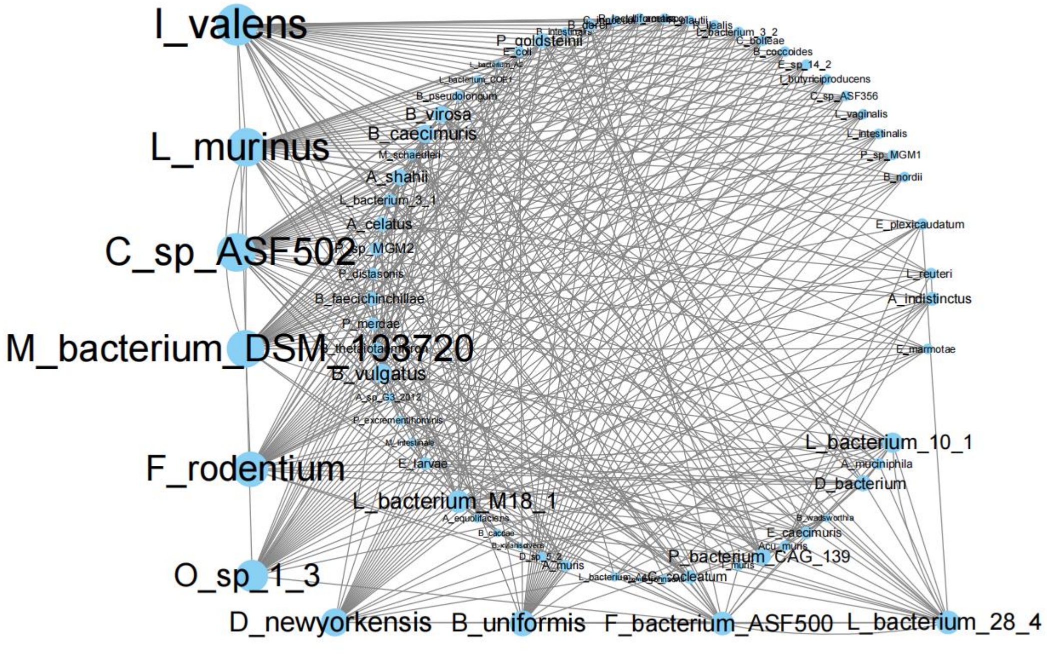

FigS2: Granger Causality network for mouse gut microorganisms treated by Dangguibuxue Decocotion with $p<10^{-4}$

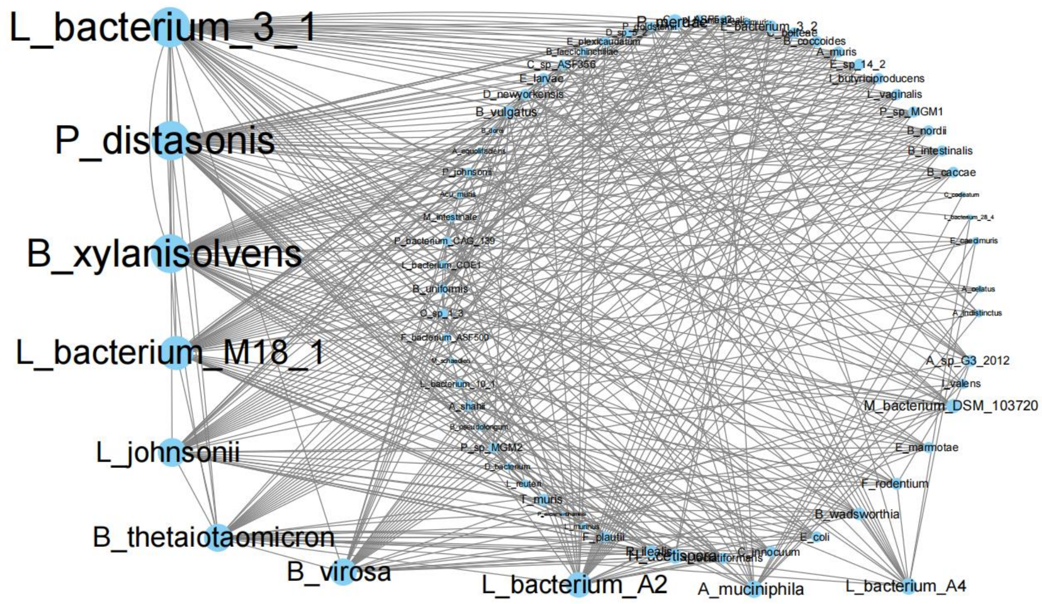

FigS3: Granger Causality network for mouse gut microorganisms treated by Codonopsis pilosula (Dang shen) with $p<10^{-4}$

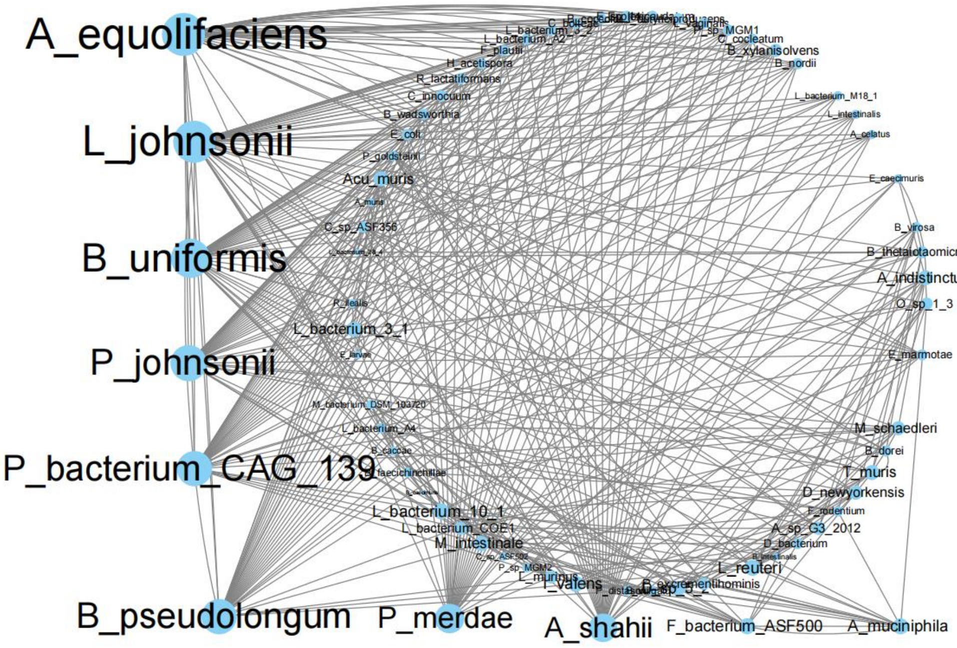

FigS4: Granger Causality network for mouse gut microorganisms treated by Poria cocos(Fu ling) with $p<10^{-4}$

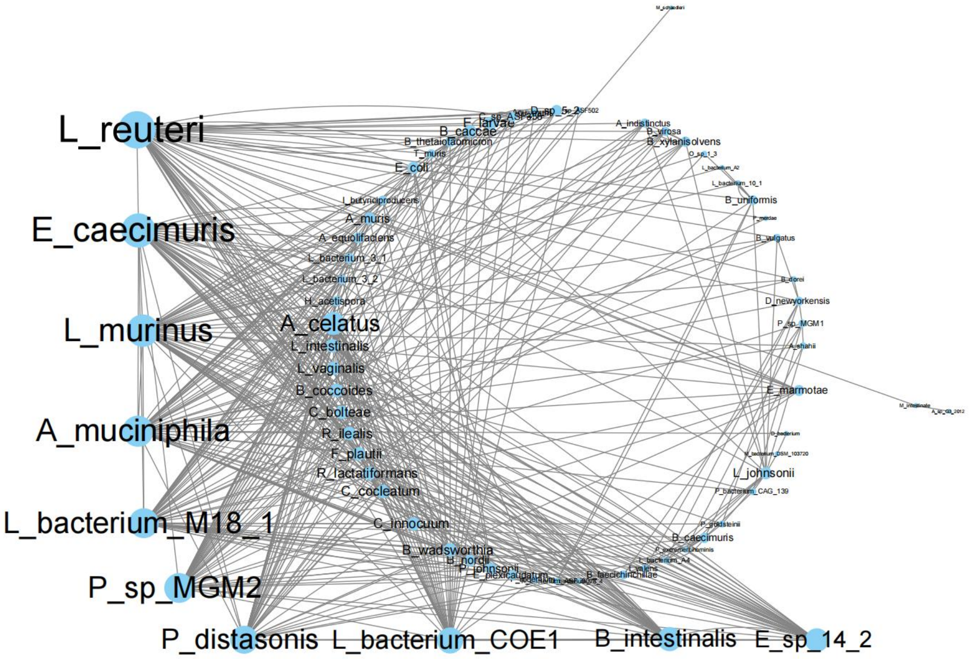

FigS5: Granger Causality network for mouse gut microorganisms treated by rhizoma zingiberis (Gan jiang) with $p<10^{-4}$

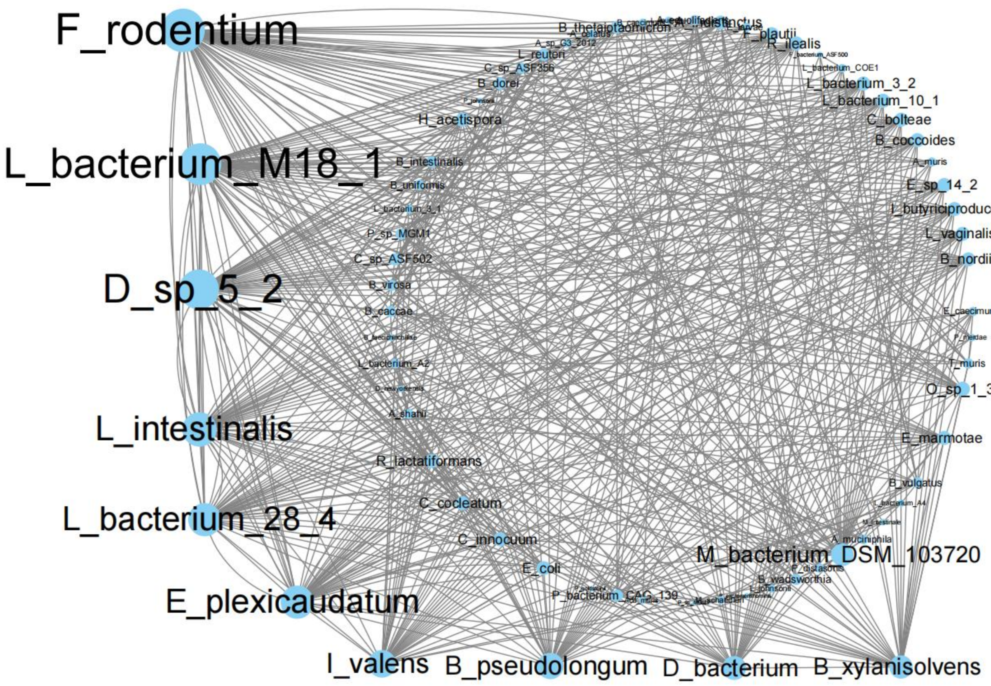

FigS6: Granger Causality network for mouse gut microorganisms treated by Cassia twig (Gui zhi) with $p<10^{-4}$

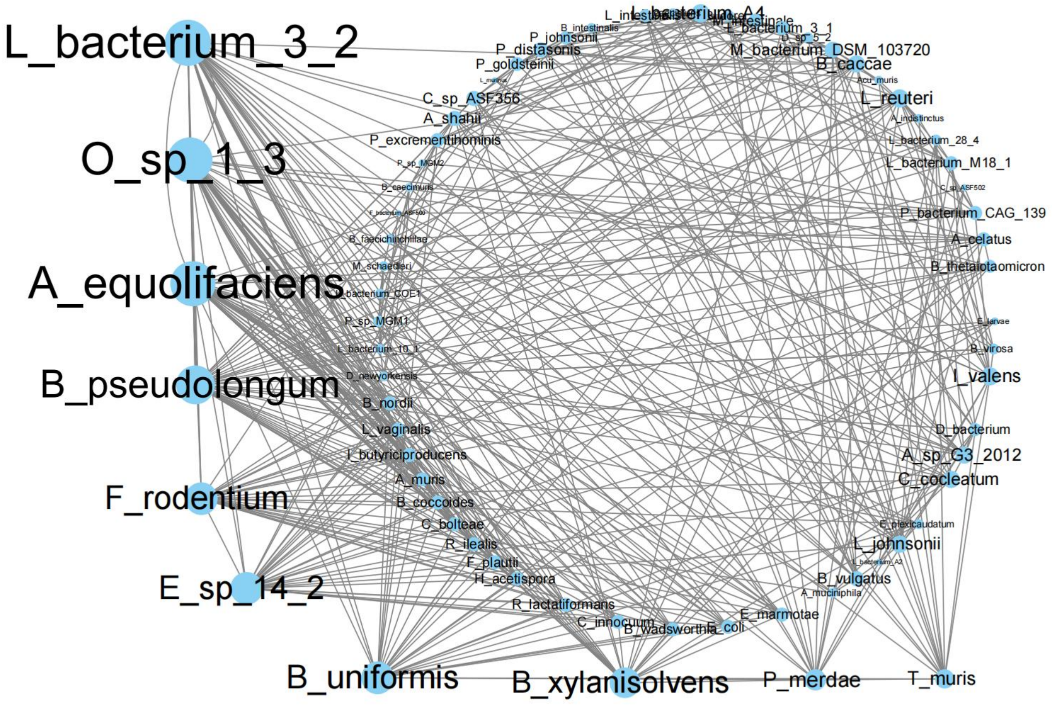

FigS7: Granger Causality network for mouse gut microorganisms treated by Mangnolia officinalis (Hou po) with $p<10^{-4}$

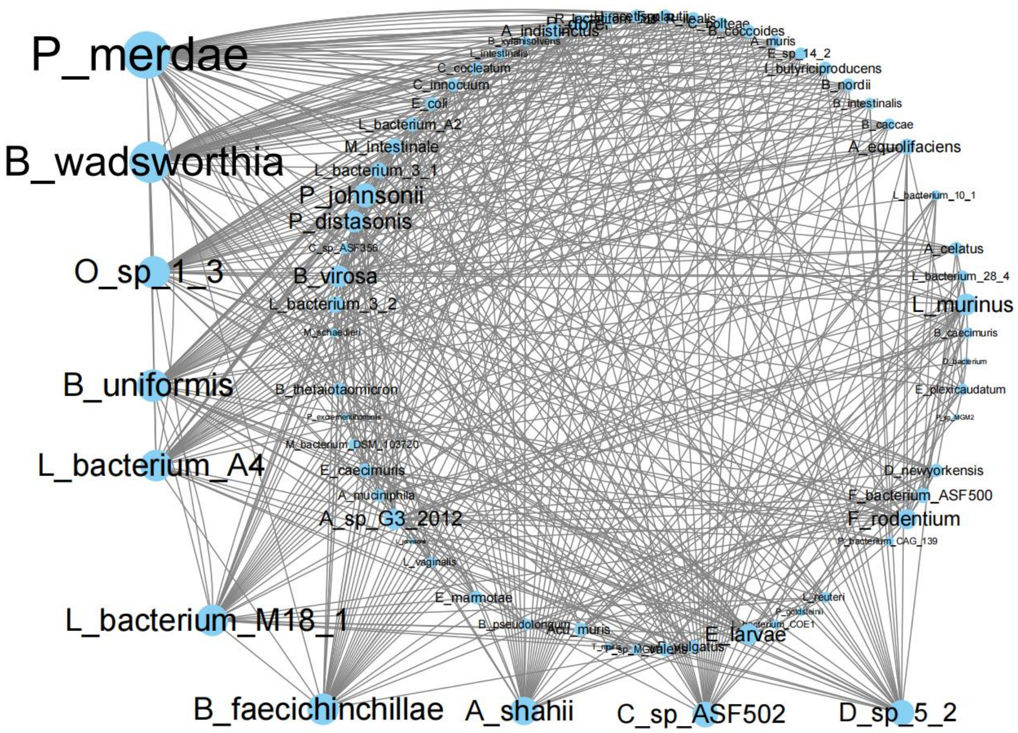

FigS8: Granger Causality network for mouse gut microorganisms treated by *Radices saussureae* (Mu xiang) with $p<10^{-4}$

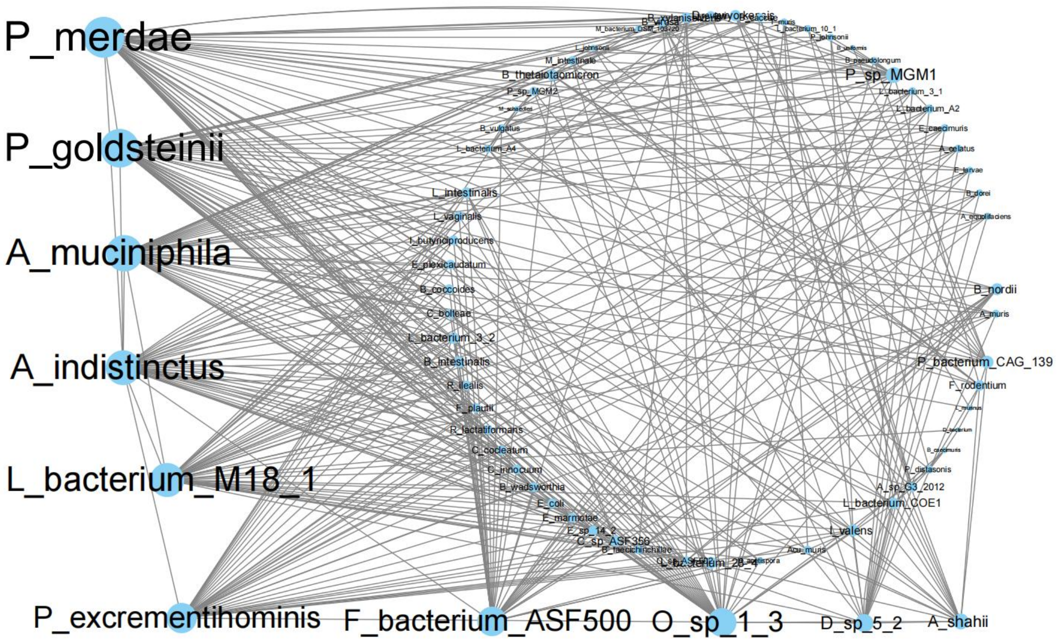

FigS9: Granger Causality network for mouse gut microorganisms in saline group with $p<10^{-4}$

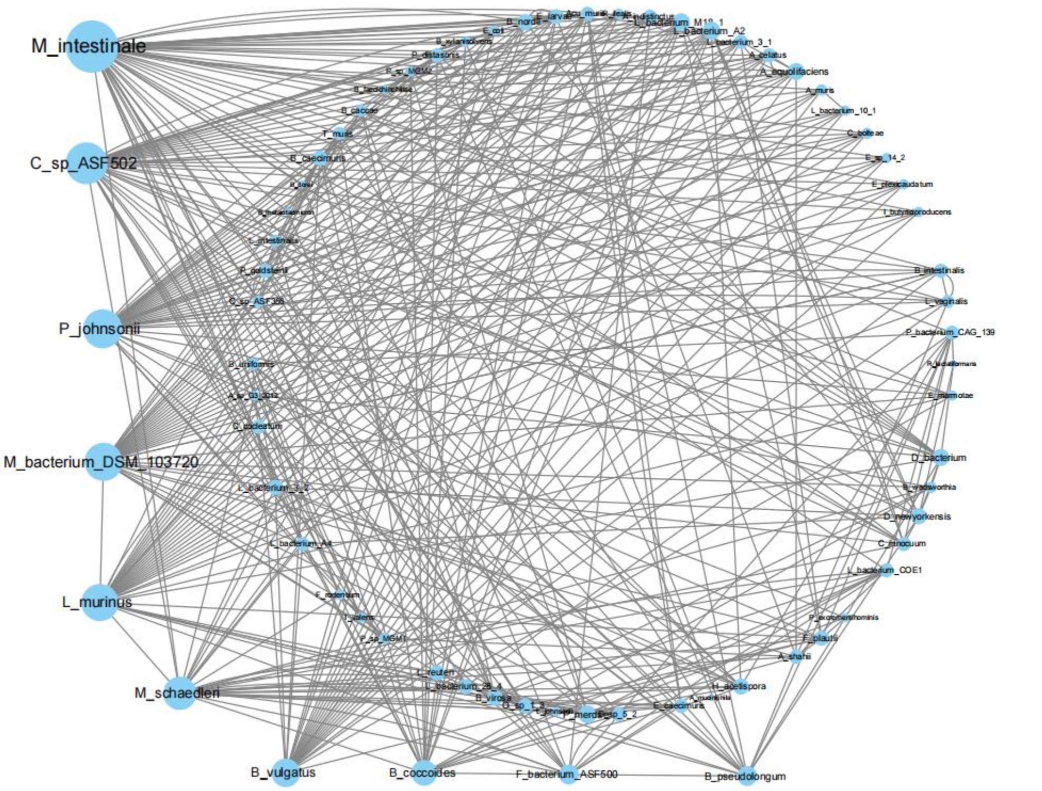

FigS10: Granger Causality network for mouse gut microorganisms treated by Rhizoma Dioscoreae (Shan yao) with $p<10^{-4}$
